# High Resolution Discovery Proteomics Reveals Candidate Disease Progression Markers of Alzheimer’s Disease in Human Cerebrospinal Fluid

**DOI:** 10.1371/journal.pone.0135365

**Published:** 2015-08-13

**Authors:** Ronald C. Hendrickson, Anita Y. H. Lee, Qinghua Song, Andy Liaw, Matt Wiener, Cloud P. Paweletz, Jeffrey L. Seeburger, Jenny Li, Fanyu Meng, Ekaterina G. Deyanova, Matthew T. Mazur, Robert E. Settlage, Xuemei Zhao, Katie Southwick, Yi Du, Dan Holder, Jeffrey R. Sachs, Omar F. Laterza, Aimee Dallob, Derek L. Chappell, Karen Snyder, Vijay Modur, Elizabeth King, Catharine Joachim, Andrey Y. Bondarenko, Mark Shearman, Keith A. Soper, A. David Smith, William Z. Potter, Ken S. Koblan, Alan B. Sachs, Nathan A. Yates

**Affiliations:** 1 Departments of Exploratory and Translational Sciences, Merck & Co., Rahway, NJ, United States of America; 2 Biometrics, Merck & Co., Rahway, NJ, United States of America; 3 Applied Computer Science and Mathematics, Merck & Co., Rahway, NJ, United States of America; 4 Clinical Neuroscience and Ophthalmology, Merck & Co., West Point, PA, United States of America; 5 Biometrics, Merck & Co., West Point, PA, United States of America; 6 Clinical Development Laboratory, Merck & Co., Rahway, NJ, United States of America; 7 Neuroscience Basic Research, Merck & Co., West Point, PA, United States of America; 8 OPTIMA, University of Oxford, Department of Pharmacology, Oxford United Kingdom; 9 Rosetta Biosoftware, Seattle, WA, United States of America; Nathan Kline Institute and New York University School of Medicine, UNITED STATES

## Abstract

Disease modifying treatments for Alzheimer’s disease (AD) constitute a major goal in medicine. Current trends suggest that biomarkers reflective of AD neuropathology and modifiable by treatment would provide supportive evidence for disease modification. Nevertheless, a lack of quantitative tools to assess disease modifying treatment effects remains a major hurdle. Cerebrospinal fluid (CSF) biochemical markers such as total tau, p-tau and Ab42 are well established markers of AD; however, global quantitative biochemical changes in CSF in AD disease progression remain largely uncharacterized. Here we applied a high resolution open discovery platform, dMS, to profile a cross-sectional cohort of lumbar CSF from post-mortem diagnosed AD patients versus those from non-AD/non-demented (control) patients. Multiple markers were identified to be statistically significant in the cohort tested. We selected two markers SME-1 (p<0.0001) and SME-2 (p = 0.0004) for evaluation in a second independent longitudinal cohort of human CSF from post-mortem diagnosed AD patients and age-matched and case-matched control patients. In cohort-2, SME-1, identified as neuronal secretory protein VGF, and SME-2, identified as neuronal pentraxin receptor-1 (NPTXR), in AD were 21% (p = 0.039) and 17% (p = 0.026) lower, at baseline, respectively, than in controls. Linear mixed model analysis in the longitudinal cohort estimate a decrease in the levels of VGF and NPTXR at the rate of 10.9% and 6.9% per year in the AD patients, whereas both markers increased in controls. Because these markers are detected by mass spectrometry without the need for antibody reagents, targeted MS based assays provide a clear translation path for evaluating selected AD disease-progression markers with high analytical precision in the clinic.

## Introduction

Alzheimer’s disease (AD) is a major neurodegenerative disease that is characterized by the progressive and selective degeneration of certain neurons in the brain, including cholinergic neurons of the basal forebrain [1]. The symptoms of cognitive impairment followed by AD include a progressive loss of memory, the loss of the ability to communicate and the loss of other cognitive functions which occur over a course of several years [2]. Although several symptomatic therapies have been approved to provide some compensation for the cholinergic deficit, for example, Aricept (donepezil), the clinical effects are modest and none are able to significantly alter the course of the disease [3]. There is a large unmet medical need for a disease-modifying therapy [4]. Improving of strategies for the treatment of AD has become a focus for the medical and scientific communities due to increases in the average age of the world population, the consequent increase in incidence and prevalence of age-related disorders such as AD, and the severe socioeconomic impact associated with supporting AD patients over the long term [5].

Requisite to improving the treatment of AD is improving the tests clinicians use to accurately diagnose the disease early in its course and to accurately monitor the progression of the disease. Following the clinical diagnosis, the progression of the disease is typically monitored through cognitive testing and assessment of everyday function. The course is often variable across patients and may be influenced by both organic and environmental elements. Hence there is a need for objective and quantitative biomarkers. AD biomarkers can be used for multiple purposes including; (1) as diagnostic markers to identify patients, (2) as disease predictive markers to forecast who is likely to develop the disease, and (3) as disease progression markers to reflect the progression of the pathophysiology.

Among the techniques that currently hold promise in this regard is the biochemical analysis of cerebrospinal fluid (CSF). The value of CSF analysis is based on the fact that the composition of this fluid may reflect brain biochemistry due to its direct contact with brain tissue. The CSF proteins that have received the most attention, CSF β-Amyloid, total tau and hyperphosphorylated tau [6] are thought to reflect key features of the disease pathogenesis, including senile plaques in the brain and intraneuronal fibrillary tangles [7–9]. For example, studies of antemortem CSF samples with post mortem verification of AD have confirmed that CSF Ab42 levels correlated inversely to amyloid load and tau levels correlated with immunohistochemical analysis of hyperphosphorylated tau and neurofibrillary tanges in brain tissue [9]. The last decade has seen an increase in efforts to identify and qualify AD-related biomarkers that might increase the sensitivity and specificity of diagnosis and provide a convenient and objective measure of disease progression [10–13].

To enable the identification of candidate disease progression biochemical markers in AD, we applied an "open" high resolution mass spectrometry-based label-free quantitative (LFQ) approach, dMS, that allows statistical analysis of the integrated ion intensity measurements in full spectrum mass spectrometry data (MS1) [14, 15]. Previously we showed that the ratio reliably detected by the dMS method was 1.5:1 [16] and the LFQ quantitation showed strong concordance with ELISA [17]. In addition, to industrialize the workflow to support large scale biomarker discovery experiments we developed a reproducible sample processing method for CSF, Elucidator, an automated end-to-end data analysis tool that includes dynamic time warping to align mass spectra across images, and demonstrated proof of concept in 108 rhesus CSF samples [18]. Here we profile archived human lumbar CSF collected in life from pathological confirmed AD patients and age-matched non-demented controls, Cohort-1, by high resolution mass spectrometry. We also constructed a random forest classifier that distinguishes between AD and Control. To further explore these biochemical markers as disease progression markers for AD, we selected two markers, SME1 and SME2, and performed mass spectrometric quantitation in a separate longitudinal cohort, Cohort-2, where CSF was drawn annually from AD patients and age-matched controls and where clinical diagnosis was confirmed post mortem. This high resolution feature based discovery proteomics approach is compatible with the analysis of many hundreds of samples and provides a direct path for translating candidate markers into multiplexed selected reaction monitoring (SRM) assays that utilize tandem mass spectrometry (MS/MS) to quantify specific analytes with high analytical precision.

## Materials and Methods

### Participants

Participants were volunteers in the OPTIMA naturalistic longitudinal study of memory and ageing. All OPTIMA's protocols were reviewed and received ethical approval from the local research ethics committee (Central Oxford Ethics Committee). OPTIMA is a convenience sample of patients with dementia and non-demented volunteers of similar age. Participants provided written informed consent to participate in the study. Original of the signed consent form was kept on file. OPTIMA's recruitment and assessment protocol has been described previously [19, 20]. We invited participants who could give valid consent to undergo a lumbar puncture (LP).

### Lumbar punctures

All LPs were taken in the supine position and used standard clinical techniques [21]. Most took place in the late morning.

### Human Cohorts (1&2) and lumbar CSF samples

Clinical diagnoses of probable Alzheimer's disease used the NINCDS criteria [22]. CSF samples collected at the bedside into polystyrene tubes. Specimens were centrifuged for 10 min at 1,000 g at 4°C to remove cellular components. The supernantent was then collected and aliquoted into polypropylene tubes that were stored at -80°C. Specimens were not thawed and re-frozen before use in this study. The handling of CSF in Corhort-1 was the same as in Cohort-2. Cohort-1 and Cohort-2 comprised controls who had been cognitively screened annually for at least two years to exclude dementia and AD cases who were severely impaired. In the longitudinal Cohort-2 samples CSF samples were collected at annual follow-up visits. The number of follow up visits post diagnosis ranged from 1 to 7 years with average follow up of 2.3 years (1.9 yrs in AD, 2.6 yrs Controls). See S3 Table for Optima coded patient ID #, time since first CSF draw ranging from 0 (first sample)–final sample (1–7 years). It is important to note the Cohort-1 group differed in age, gender distribution and occurrence of the Apo ε4 genotype. The Cohort-2 groups were similar in age and gender distribution but differed in Apo ε4 genotype. These possible co-variants were not included in the modeling.

### Differential mass Spectrometry (dMS)

Differential mass spectrometry (dMS) is a general proteomic workflow we have described earlier ([14–18]) that allows statistical analysis of the integrated ion intensity measurements in full spectrum mass spectrometry data (MS1). dMS includes the following steps: collect biological samples, perform reproducible biochemical sample processing, analyze them using liquid chromatography mass spectrometry (LCMS), align the LCMS spectra obtained for different samples, extract features from the aligned LCMS data, perform statistical tests to find features differentially expressed across experimental conditions, select features of particular interest, perform targeted analysis to obtain MS/MS spectra for those features, and determine the amino acid sequence of the analytes from the MS/MS spectra.

### Biochemical sample processing of CSF samples

Aliquots (400μL) of neat lumbar CSF were processed 4 per block using 4 individual MARS spin columns (Aglient) as per manufacturer’s instructions with minor modifications as previously described [18]. All samples were thawed at the same time, spiked with internal controls [18], and refrozen and stored for proteomic analysis. Samples were processed in an interwoven block design. Briefly, CSF flow thru was collected, desalted with a 5 kDa cutoff filter, and concentrated. The sample was reconstituted in 50 mM NH4CO3, proteins reduced with 4mM TCEP and alkylated with 10 mM iodoacetamide in the dark. Proteins were digested to peptides by an overnight incubation 1:50 with sequence grade trypsin (Promega). Peptide digests were quenched with 10μL neat acetic acid, desalted on a Michrom peptide trap column, concentrated to dryness then resuspended in 40μL 0.1M acetic acid. Cohort-1 was processed independently and data analysis completed before the initiation of experiments in Cohort-2. The time interval between initiation of proteomic experiments in Cohort-1 and initiation of proteomic experiments in Cohort-2 was 18 months. Experimental block design, run order and acquisition date for Cohort-1 is provided in S1 Table. Block design, blinded sample name and run order for the Cohort-2 longitudinal study is provided in S3 Table.

### LC-MS profiling

Each CSF sample was analyzed by a reverse phase nano-HPLC coupled to an LTQ-FTMS hybrid mass spectrometer (ThermoFisher) essentially as described earlier [16]. Full scan high resolution mass spectra were recorded at a rate of 1 spectrum per second using a 300 Da to 2000 Da scan range. OneμL (equivalent of 10 μL immunodepleted CSF) of digested sample was loaded with a Famos autosampler (LC Packings) onto a capillary sample trap column (100μm ID, 2.5 cm) and desalted on line for 3 min at 3μL/min with solvent A [100% HPLC grade water, 0.1 M acetic acid]. After 4 minutes the flow rate was reduced to 1 μl/min and peptides were eluted into an in-house packed spray tip column (100 μm i.d., 190μm o.d. × 8 cm; POROS R2; flame pulled tip ~ 5μm). Peptides were analyzed on a hybrid LTQ-FTMS (positive mode, AGC = 100000, maximum injection time = 1s, spray voltage = 2.5 kV, capillary temperature at 240 C) using a 75 min gradient run. The gradient was delivered by an Agilent 1100 capillary pump and had four distinct sections; a) 100% A at a flow rate of 3 μL/min from 0 to 3 min., b) 3.01 to 5 min binary gradient from 0% to 6% solvent B [90% acetonitrile, 10% 0.1 M acetic acid] at a flow rate of 1.0μL/min, c) 5.01 min to 39 min to 30% B at 1μL/min, and d) 30 to 60 min to 90% Solvent B, followed by equilibration 100% A at 1μL/min from 60.01 to 75 min.

### Feature extraction and filtering

Expression profiles generated from the raw LC-MS data collected for each CSF sample were analyzed using the Elucidator (Rosetta Biosoftware, Seattle, WA, Version: 3.1) proteomics data analysis suite. The PeakTeller algorithm was used to align, measure and extract, m/z, retention time, and intensity data (peak area) for the features contained in the data set [18]. A feature is specified by an m/z and a time range, and for each LCMS data set, the feature’s value is the area under the curve for the feature, that is, the sum of measured intensities for that m/z in that time range. Some signals are believed to arise as multiple charge states and/or isotopes of a single underlying analyte; these signals are said to form a group.

Expression profiles generated from the raw LC-MS data collected from each CSF sample in Cohort-2 were analyzed using Elucidator as described above. Elucidator feature extraction of the Cohort-2 data set revealed a total of 8,108 features detected and quantified in the data set. As naturally occurring isotope distributions contain both ^12^C and ^13^C, we removed features in the dataset where the isotope count < 2 (7,827 features remain), removed features with charge state = 0 (7,827 features remain), and kept the feature with maximum peak intensity (composite image) in each isotope group (2409 features remain).

### Number of positives vs. number of false positives

**Fig 1:**
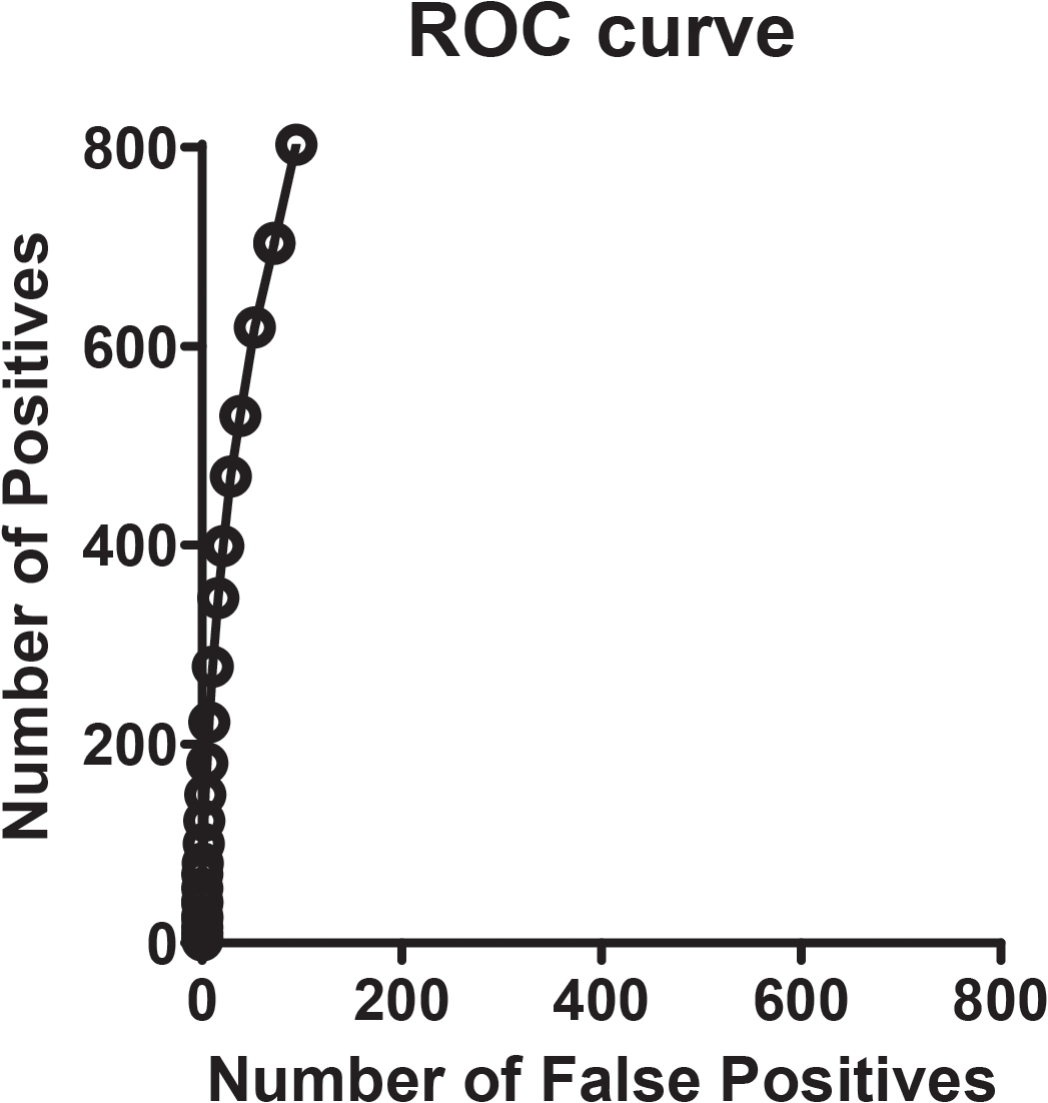
Receiver-operator curves (ROC) for cross-sectional study (Cohort-1). Comparison of the AD samples versus the control samples was used to estimate the sensitivity, while the comparisons between two groups comprised of randomly selected but equally balanced AD and control samples were used to estimate specificity.

To determine the number of features that show a statistically significant difference in abundance (total positives), the Wilcoxon Rank Sum test was performed using filtered data from two groups, 10 control and 10 disease. To establish the number of false positives that might be included in the detected differences, the labels were randomly shuffled 10 times and the same Wilcoxon Rank Sum test was performed. For a given confidence threshold (similar to a p-value), one can determine the number of differences (statistically significant features) in the original comparison and the estimated number of false positive results (from the second comparison) at that confidence threshold. Plotting the number of actual differences versus the estimated number of false positives for different values of the confidence threshold traces out a curve estimating the number of positives vs. the number of false positives (Fig 1).

### Random forest classifier

The data from Cohort-1 are also analyzed by the random forests cross-validation procedure [23]. The process involves repetitions of nested cross-validations. In the "inner" cross-validation, a random forest classifier is built using the log intensity values of all features, and the importance ranking of features generated. Then the least important half of the features are removed, and a classifier is built again. The process is repeated until no more features can be removed. Each of these classifiers are evaluated in the "outer" cross-validation to ensure accurate assessment of classifier performance. This process generates a curve of number of features vs. classifier performance, and provides guidance as to the number of features a classifier needs to retain good prediction performance.

### Linear effects mixed model analysis

For each feature in the Cohort-2 data, a linear mixed-effects model is fitted with log intensity as the response, years (since last visit with "normal" status), group (control or AD) and interaction between years and group as fixed effects, and subject and years as random effects. The model assumes that there is a linear time trend in both populations (control vs. AD), as well as allowing each subject in the group to deviate from the population trend of the group. From each fitted model, we obtain the ratio of the two groups at baseline, the percent change per year for the groups. We can test whether the time trend in the AD group is significant, as well as whether the trend is different from that in the control group. A candidate feature would have both tests showing statistical significance.

### LC-MS/MS and Peptide Identification

The LC-MS/MS spectra were acquired either by data-dependent acquisition or by targeting a specific *m/z*. In both cases a 2 *m/z* isolation width was used. Each MS/MS product ion spectra was linked to a corresponding precursor ion feature, and DTA files were created for all precursor-linked MS/MS spectra from all raw data files. The DTA files were searched against a human International Protein Index (IPI) database (version 3.75) using the SEQUEST search algorithm (version 2.7). The human IPI database contained 89,486 protein sequences, and was appended with decoy reverse sequences for all proteins to determine false discovery rate [24]. Search parameters specified a tryptic enzyme cleavage at lysine or arginine, except when followed by a proline residue. A maximum of three missed cleavages were allowed within each peptide. Additional parameters included a precursor ion tolerance of ±80 ppm, fragment ion tolerance of 0.5 Da, fixed alkylation of cysteine (+57.021 Da), variable oxidation of methionine (+15.9950 Da) and variable phosphorylation of serine, threonine and tyrosine (+79.9660 Da). All other parameters were set to default SEQUEST values. Search result out-files were submitted to a Prophets-based algorithm in Elucidator (Peptide Tellers) to create a statistical model for assigning probabilities to peptide identifications [25, 26]. Filter settings were set to a predicted false discovery error rate of 0.005, which resulted in the identification of 736 peptides from 155 proteins.

### Ab1-42, tau, and p-tau ELISA

Ab-1-42, tau and p-tau were measured using a validated ELISA assay as described previously [27]. Ab1-42 was measured with Innotest Ab1-42 ELISA kit (Innogenetics Inc., Cat. #80040, Ghent, Belgium) following the manufacturer’s recommendations with some modifications. Ab1-42 present in human CSF samples was first captured with a mouse monoclonal antibody specific for the C-terminal half of Ab The detection system employs an N-terminal specific biotinylated mouse monoclonal antibody and a secondary conjugate made of HRP labeled strepavidin. The HRP is used to convert tetramethyl benzidine to a chromophore which is quantitatively measured at 450 nm. A total of 100μL of the sample (CSF Diluted 1:3 with Sample Diluent) was used in each reaction. Ab1-42 standard was purchased from American Peptide (Sunnyvale, CA, USA) and the concentration was determined by amino-acid analysis. Standard concentrations in the assay ranged from 5.45 to 350 pg/mL. Total Tau (t-Tau) expression was measured with a human Tau (hTAU AG Innotest) ELISA kit (Innogenetics Inc., catalogue number 80226, Ghent, Belgium) following the manufacturer’s recommendations. Phosphorylated Tau-181 (pTau-181) was measured with the Phospho-TAU (181P) Innotest ELISA kit (Innogenetics Inc., catalogue number 80062, Ghent, Belgium), following the manufacturer’s recommendations.

## Results

### Biochemical Changes between AD and Control–discovery by high resolution LC-MS profiling and statistical analysis of Cohort-1 samples

High resolution mass spectrometry was used to profile the lumbar CSF from Cohort-1 (Table 1). The mass spectrometry data was acquired at a resolving power of 60,000 with a mass accuracy of 5 parts per million or less. Experimental block design including blinded sample name, date LC-MS data acquired, and LC-MS run order is provided in S1 Table. A total of 19,883 features (where a feature is defined as an isotope peak with a measured relative abundance (AUC), distinct mass-to-charge ratio (*m/z*) and retention time) were extracted from the LC-MS data, which were further grouped into 6,221 isotope groups. As naturally occurring isotope distributions contain both ^12^C and ^13^C we removed features in the data set where the isotope count <2, charge state <1, and selected the features with maximum peak intensity in each isotope group (4764 features), and removed features in the bottom 10% of abundance resulting in 3941 features in the "filtered" data set. To determine the number of features that show a statistically significant difference in abundance (total positives), a non-parametric version of the 2-sample t-test (Wilcoxon Rank Sum test) [28] was performed using filtered data from two groups, 10 control and 10 AD. To establish the number of false positives that might be included in the detected differences, the labels were randomly shuffled 10 times and the same Wilcoxon Rank Sum test was performed. As shown in Fig 1, we estimate that there are several hundred interesting features in the initial dataset of approximately 20,000 features.

**Table 1:** The demographic characteristics of the cross sectional study population, Cohort-1, at the time of CSF collection.

	CTL	AD
	(N = 10)	(N = 10)
Gender, n (%)		
Female	2 (20%)	8 (80%)
Male	8 (80%)	2 (20%)
Age, years		
Mean (SD)	30 (0)	3.1 (3.2)
Range	36–80	0–9
MMSE		
Mean (SD)	30 (0)	3.1 (3,2)
Range	30–30	0–9
ApoE Genotype, n (%)		
E4 (-)	7 (70%)	2 (20%)
E4	3 (30%)	8 (80%)

CTL = control; AD = Alzheimer's disease; N, n = number of subjects/patients; SD = standard deviation; MMSE = Mini- mental state examination; ApoE = Apolipoprotein E

To assess the ability of the Cohort-1 LC-MS data to distinguish between AD and control samples, a random forest classifier was built as described in the Methods. The 10-fold cross-validation procedure used to evaluate the performance of the classifier for the 2-class, 20-subject, data set shows that the classifier based on the 100 most highly ranked features did very well in predicting control and AD within the data set (accuracies are 99.9% and 99.8% respectively). Two major limitations, however, of cross-validation classification are the lack of an independent validation set and, in the case for 'omics' discovery platforms, the need for a multiplicity correction to reduce the likelihood of over fitting that can occur when a large number of data points are used to discriminate among a small set of outcomes [29]. To address these limitations, we chose to select two markers for further testing in an independent cohort.

### Two biochemical markers are selected for testing in an independent longitudinal cohort

**Fig 2:**
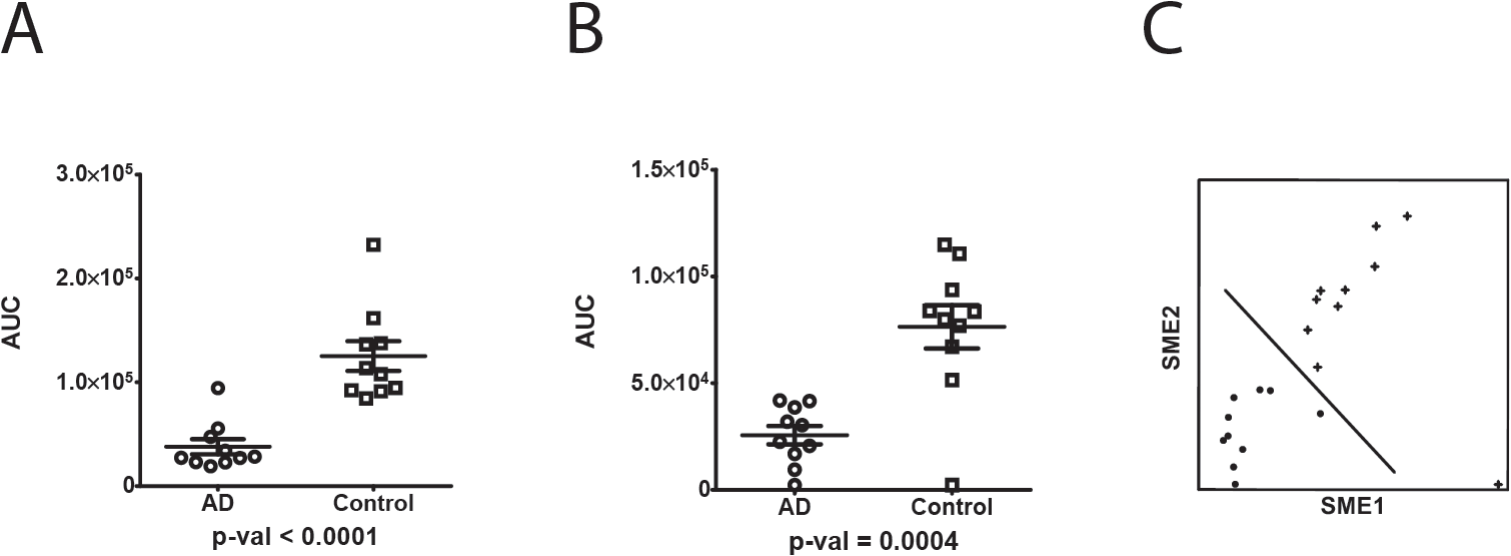
Quantitation of SME1 and SME2 by dMS (Cohort-1) and 2-way plot. SME 1 (Peptide NSEPQDEGELFQGVDPR, from neurosecretory protein VGF precursor) and SME2 (peptide VAELEHGSSAYSPPDAFK, from neuronal pentraxin receptor-1) are significantly reduced in AD patients as compared to controls. Area under the curve (AUC) intensity measurements are shown on linear scale. Horizontal bar represents the mean value, vertical error bar represents SD. (A) SME1, P < 0.0001. (B) SME2, P < 0.0005. (C) Two way plot of SME1 and SME2. The symbols (●, +) represent AD and control, respectively.

To further understand the biochemical markers in the classifier, we selected two from the top 100 ranked features for testing in a second cohort (see S2 Table for the random forest classifier, top 100 features). As the longer term goal is a clinical assay, we selected biochemical markers that are readily measurable in human CSF. We looked for features that had good chromatographic peak shape, fell at the middle of the chromatographic separation, and exhibit good signal-to-noise in the samples tested. SME1 and SME2 were selected. Both markers, SME1 (*p* < 0.0001) and SME2 (*p* = 0.0004) are significantly reduced in AD patients as compared to controls showing a 80% and 70% decrease, respectively, (Fig 2A and 2B) in the Cohort-1 tested. A two way plot of SME 1 and SME 2 shows the two markers readily distinguish AD vs. control in the samples tested (Fig 2C).

### Linear mixed-effect analysis shows SME-1 and SME-2 change with disease progression in Cohort-2

A second independent longitudinal cohort of lumbar CSF from post-mortem diagnosed AD patients and aged-matched case-matched control patients (Cohort-2) was obtained. Patient demographic data is shown in Table 2. Control subjects were screened annually to exclude any with dementia and AD patients were initially mildly to moderately impaired. In this study, N = 30 control patients (mean MMSE = 28.7 ± 1.7) and N = 30 AD patients (mean MMSE 16.3 ± 7.9) provided 176 CSF samples for dMS analysis at multiple points per patient at approximately annual intervals from initial diagnosis. As two markers, SME1 and SME2, were selected in Cohort-1 to test a pre-specified hypothesis in Cohort-2, no multiplicity correction is required. To avoid systematic error, samples were processed in an interwoven block design (S3 Table) and the laboratory personnel were blinded to sample identities. A total of 8,108 features as defined by distinct *m/z*, retention time and an intensity value were extracted from the LC-MS data, which were further grouped into 2,409 isotope groups. SME-1 and SME-2 showed lower levels in AD compared with controls at baseline -27% (p = 0.026) and -21% (p = 0.039), respectively (Table 3, S4 Table). Linear mixed model analysis in the longitudinal samples estimated the rate of decrease in the levels in AD to be -10.9% (p = 0.023) and -6.9% (p = 0.016) per year for SME-1 and SME-2, respectively, in the cohort tested (Table 3, S1 Fig, S2 Fig and S3 Fig). These results demonstrate that the phenomenon of significantly reduced SME1 and SME2 replicates in the second cohort tested and change with disease progression. Classical markers, Ab42, tau, and p-tau, measured by ELISA in the same Cohort-2 samples show an estimated decrease of -7.2% (p = 0.003), -4.9% (p<0.001), and -6.8% (p<0.001), respectively using the LME method. Total protein levels in the CSF samples were measured to exclude a simple dilution effect due to brain atrophy over time. No significant change in CSF protein concentration was observed in the AD patients as compared to control (p = 0.056) however, a trend towards reduced protein concentration in AD over time was seen (-3.4%/yr (p = 0.054)).

**Table 2:** The demographic characteristics of the longitudinal study population, Cohort-2, at the time of CSF collection.

	CTL (N = 30)	AD (N = 30)
Gender, n (%)		
Female	19 (63%)	19 (63%)
Male	11 (37%)	11 (37%)
Age, years^a^		
Mean (SD)	69.0 (9.0)	70.0 (8.0)
Range	45–84	57–84
MMSE		
Mean (SD)	28.7 (1.7)	16.3 (7.9)
Range	24–30	0–30
ApoE Genotype, n (%)		
E4 (-)	24 (80%)	8 (27%)
E4 (+)	6 (20%)	22 (73%)

a: Data at the first visit.

CTL = control; AD = Alzheimer's disease; N, n = number of subjects/patients; SD = standard deviation; MMSE = Mini- mental state examination; ApoE = Apolipoprotein E

**Table 3:** Linear mixed effect model analysis of SME1 and SME2 and tau, p-tau, and AB42 in the longitudinal cohort, Cohort-2. Serial CSF samples were taken annually. Log area under the curve (AUC) intensity values for SME1 and SME2 measured by dMS and ELISA measurements for tau, p-tau, and AB42 were analyzed in the mixed-effect model described in methods.

Analyte	Assay Type	Baseline fold change	Rate of change per year (AD)	Rate of change per year (CTL)	p-value AD slope vs. 0	p-value AD slope vs. CTL slope
SME1	dMS	↓ AD; 0.7	-10.90%	4.00%	0.026	0.023
SME2	dMS	↓ AD; 0.8	-6.90%	4.10%	0.039	0.016
tau	ELISA	↓ AD; 0.3	-4.87%	0.59%	0.003	0.009
p-tau	ELISA	↓ AD; 0.5	-6.77%	-0.02%	<0.001	<0.001
Aβ42	ELISA	↓ AD; 0.4	-7.23%	-0.69%	<0.001	0.003

**SME1:** dMS feature ID 751080736 at m/z = 639.63 Da; z = 3; Mo = 1915.85 Da; AA sequence = NSEPQDEGELFQGVDPR; unique to protein sequence IPI00289501.2 Neurosecretory protein VGF.

**SME2** = dMS feature ID751082515 at m/z = 635.98 Da, z = 3, Mo = 1903.90 Da, AA sequence = VAELEHGSSAYSPPDAFK, unique to protein sequence IPI00334238.1 Neuronal pentraxin receptor 1 (NPTXR).

### Targeted MS/MS analysis identifies neuronal pentraxin receptor 1 and neuronal secretory protein VGF

Because differential mass spectrometry (dMS) allows statistical analysis of the AUCs (integrated peak area under the curve) prior to peptide identification, we then performed targeted LC-MS/MS to determine the identity of SME1 and SME2. Collision activated dissociation (CAD) tandem mass spectra generated on the (M+3H)^3+^ ions at *m/z* = 639.6 and retention time 24.8 minutes reveals SME1 has the amino acid sequence NSEPQDEGELFQGVDPR. SME1 peptide is unique to the neuronal secretory protein VGF, a neuroendocrine specific gene product that is thought to play a role in neuronal communication. CAD tandem mass spectra generated on the (M+3H)^3+^ ions at *m/z* = 635.9 and retention time 22.3 minutes reveals SME2 has the amino acid sequence VAELEHGSSAYSPPDAFK. SME2 is a unique peptide from Neuronal pentraxin receptor-1, a 65 kDa type II transmembrane protein. Neuronal pentraxin receptor 1 (NPTXR) is primarily expressed in the central nervous system with highest expression in the neurons and moderate expression in glial cells.

### Post-hoc mixed-effect analysis of entire dataset shows additional features that change with disease progression

Although the primary hypothesis was to test VGF (SME1) and NPTXR (SME2) in cohort-2, given dMS is an open platform that measures the integrated peak area from MS-1 data, we performed an unbiased analysis of the entire Cohort-2 data set (8,108 features, S5 Table) to identify additional candidate markers of disease progression. Table 4 shows the top 25 features from the linear-mixed model analysis as described in Methods. Targeted MS/MS analysis led to the identification of 9 out of the 25 candidate disease progression markers from Table 4. Conversely, an analysis of the Cohort-2 dataset can be performed to look for a protein that is fairly abundant in CSF and stable in concentration over time. For example, a feature at m/z 955.489 and 32.1 minutes is detected with good signal-to-noise and has a slight positive slope of 3.3 percent in AD over time and a CV of 70.5% across all subjects at all time points. This feature was identified as a peptide with sequence AQGFTEDTIVFLPQTDK from the protein Prostaglandin-H2D-isomerase (S5 Table, Feature ID 751077582).

**Table 4:** Twenty-five candidate disease progression biomarkers from post-hoc mixed-effect analysis of entire Cohort-2 proteomics data set. Linear mixed-effect model analysis of log area under the curve (AUC) intensity values for features quantitated by dMS in CSF samples taken annually in longitudinal cohort, Cohort-2. Details described in Methods.

								AD		CTL			Peak area (AUC)			
Feature #	m/z	rt (min)	Charge	m.w.	Protein Description	Peptide Sequence	Baseline Fold Change	Change (%/yr)	p-value	Change (%/yr)	p-value	Min	Median	Max	Feature ID#	IPI Number
1	455.7	17.3	2	909.5			1.0	-7.7	0.030	7.3	0.002	240	14,530	43,287	751082760	
2	782.9	23.2	2	1563.8			0.9	-8.7	0.026	5.1	0.009	493	16,151	52,696	751082162	
3	889.42	25.4	2	1775.8			0.9	-25.7	0.005	3.1	0.017	0	12,370	47,051	751083192	
4	635.98	22.3	3	1903.9	Neuronal pentraxin receptor	VAELEHGSSAYSPPDAFK	0.8	-6.9	0.039	4.1	0.016	233	15,355	46,197	751082516	IPI00334238.1
5	954.5	45.6	2	1906			0.7	-16.4	0.009	2.3	0.024	674	47,846	252,806	751080232	
6	639.63	24.8	3	1915.9	Neurosecretory protein VGF	NSEPQDEGELFQGVDPR	0.7	-10.9	0.026	4.0	0.023	1,061	25,643	169,040	751080736	IPI00289501.2
7	958.94	24.8	2	1915.9	Neurosecretory protein VGF	NSEPQDEGELFQGVDPR	0.7	-14.1	0.001	4.9	0.001	545	17,336	118,995	751081805	IPI00289501.2
8	670	31.5	3	2006			1.1	-15.1	0.028	6.0	0.026	0	10,172	31,052	751083913	
9	692.67	23.7	3	2074	Chromogranin A	YPGPQAEGDSEGLSQGLVDR	0.9	-11.0	0.006	5.8	0.002	4,438	160,575	447,877	751078143	IPI00290315.4
10	1038.5	23.7	2	2074	Chromogranin A	YPGPQAEGDSEGLSQGLVDR	0.9	-11.2	0.009	6.7	0.002	611	55,306	197,973	751079338	IPI00290315.4
11	710.69	24.6	3	2128.1			0.9	-12.7	0.037	5.1	0.024	0	15,243	52,888	751082849	
12	721.97	25	3	2161.9	Neurosecretory protein VGF	VGEEDEEAAEAEAEAEEAER	0.7	-18.0	0.000	2.2	0.001	2,054	36,247	92,088	751080944	IPI00289501.2
13	1082.46	25	2	2161.9	Neurosecretory protein VGF	VGEEDEEAAEAEAEAEEAER	0.7	-22.2	0.000	1.8	0.001	776	14,907	40,473	751083850	IPI00289501.2
14	739.35	16.2	3	2214	Chromogranin A	SGEATDGARPQALPEPM[OH]QESK	0.9	-15.2	0.000	-0.4	0.001	875	27,060	183,680	751080593	IPI00290315.4
15	468.22	17.2	5	2335			1.2	-6.0	0.011	2.1	0.010	7,570	53,644	315,132	751079622	
16	793.37	28.7	3	2376.1			1.2	-13.9	0.034	9.0	0.011	0	22,973	109,084	751081116	
17	550.49	18.3	5	2746.4			0.7	-14.2	0.005	5.2	0.004	1,947	109,445	806,451	751078288	
18	687.86	18.3	4	2746.4			0.7	-15.8	0.002	5.9	0.002	759	40,389	293,509	751079713	
19	589.31	19.8	5	2940.5			0.8	-15.6	0.000	5.0	0.000	332	16,266	84,491	751082329	
20	603.52	20.2	5	3011.6			0.7	-11.0	0.010	2.5	0.017	10,041	186,415	970,918	751077996	
21	754.15	20.2	4	3011.6			0.7	-12.0	0.004	2.8	0.008	1,060	17,295	90,609	751081906	
22	606.52	20.1	5	3026.5			0.7	-14.0	0.001	2.9	0.003	364	12,955	64,347	751082959	
23	1107.5	22	3	3318.5	Chromogranin A	AEGNNQAPGEEEEEEEEATNTHPPASLPSQK	1.0	-9.3	0.037	7.5	0.006	2,262	78,864	260,512	751078946	IPI00290315.4
24	963.71	29.1	4	3848.8			1.0	-8.3	0.038	6.2	0.008	113	12,295	35,734	751083524	
25	975.77	49	4	3897			0.7	-11.3	0.003	2.8	0.004	1,444	24,584	156,246	751081006	

### Neurosecretory protein VGF and NPTXR are readily measured with high analytical precision in a sensitive and selective MS/MS based assay

**Fig 3:**
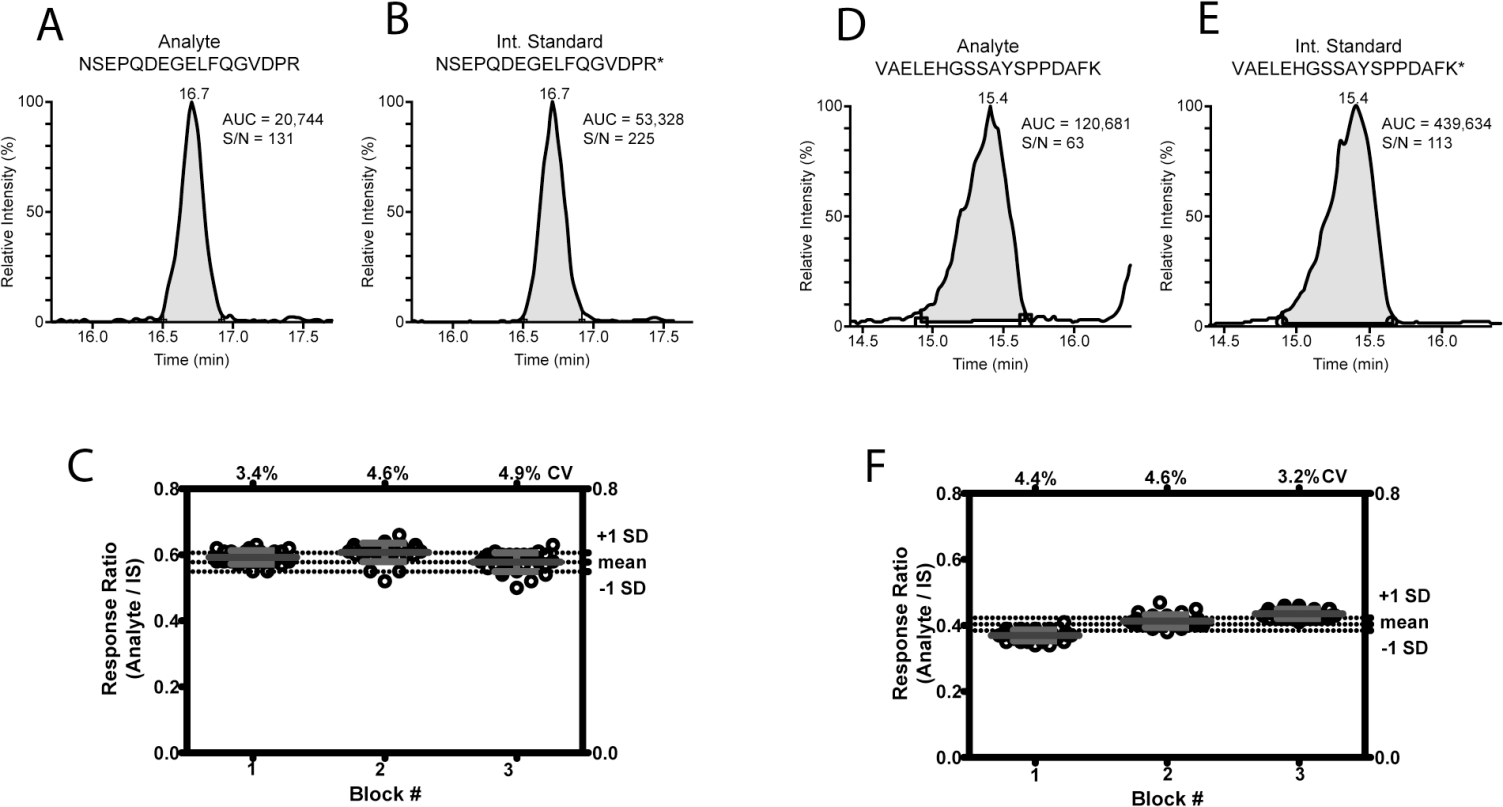
SRM mass spectrometry based assay to quantitate SME1 and SME2 from human CSF. Example total ion current chromatograms for peptide (A) NSEPQDEGELFQGVDPR from neurosecretory protein VGF (SME1) and (B) the corresponding stable isotope labeled internal standard peptide; and (D) VAELEHGSSAYSPPDAFK from Neuronal Pentraxin Receptor (SME2) and the corresponding internal standard peptide (E) measured by SRM. The observed signal corresponds to the injection of a 4μL equivalent of CSF on column with an internal standard concentration of 26 nM. The peak area (AUCs) measured using LCQuan are shown in grey for each peak. A signal-to-noise ratio measured for the endogenous NSEPQDEGELFQGVDPR and VAELEHGSSAYSPPDAFK was 131 and 63, respectively. Analysis 90 individual technical replicates of 100μL CSF (C) and (F). Response ratios were determined for 90 technical replicates of CSF. Samples were processed in 3 blocks of 30 samples. The mean value (blue line) and standard deviation (red error bars) are shown for each block. The mean value and standard deviation for all samples (black dashed line) is shown on the right hand vertical axis. The coefficient of variation is indicated for each block on the top horizontal axis. Within and between block variability, as determined by ANOVA, is shown on the bottom of each plot.

Our discovery proteomic study guided the selection of unique peptides from which to develop a sensitive and precise mass spectrometry based assay suitable for clinical use. As a proof of principle, we selected SME1 (neurosecretory protein VGF) and SME 2 (NPTXR) and developed a multiplexed mass spectrometry based SRM assay to quantitate neurosecretory protein VGF and NPTXR in human CSF. A simplified and improved assay requiring 100μL neat CSF, no antibody reagents, and minimal sample processing was developed. Shown in Fig 3 are the total ion current chromatograms for endogenous VGF (Fig 3A) and the corresponding stable isotope labeled internal standard (Fig 3B). Importantly, we observe a strong positive signal from 4μL equivalent of CSF loaded on column (S/N 131) with an internal standard concentration of 26 nM. Thus, the calculated concentration of endogenous VGF is 11.3 nM. Ion chromatograms for endogenous NPTXR and the internal standard is shown in Fig 3D and 3E, respectively, with a calculated endogenous concentration of 7.98 nM. To determine the reproducibility of the MS/MS based assay, response ratios were measured for 90 technical replicates in 3 blocks of 30 samples. We observed high precision in the measurement with the % coefficient of variation less than 5% when using the stable isotope labeled protein at the internal standard. Interestingly, the within block variability and between block variability for each analyte were different; however the total variability was well below 10% CV.

## Discussion

CSF biochemical markers such as total tau, p-tau and Ab42 are well established markers of AD [7]. Although global analysis of CSF in AD patients has been performed using multiple proteomic technologies; including chip based [30, 31], 2D-DIGE [32–36], and mass spectrometry [37], global quantitative biochemical changes in CSF in AD disease progression however remains largely uncharacterized. Here we have demonstrated a high resolution discovery proteomics approach for the identification of candidate CSF AD markers, testing of these markers in an independent longitudinal AD cohort against age-matched case match controls, and developing a sensitive and selective quantitative multiplexed assay suitable for further rigorous clinical validation. In contrast to traditional LC-MS proteomics discovery experiments, which rely on abundance-dependent sampling by MS/MS [38, 39], we relied on an unbiased analysis of integrated ion signal recorded in high resolution full scan mass spectra (MS1). This method provides several immediate advantages. First, this is a protein identification-independent analysis that utilizes all of the ions detected in the full spectrum MS1 data, not just ions that have corresponding MS/MS spectra (MS2) to yield peptide sequence information. Secondly, significant features, or peptides, are selected based on statistical analysis of the integrated area under the curve (AUC) measures, not spectral counts, that span a wide range (>10^5^) of relative abundance. Thirdly, the method does not utilize pooling or complex chemical labeling steps and thus allows the rapid testing of the particular analytes in an additional cohort(s) to replicate the findings. Finally, as the candidate markers are discovered by full scan mass spectrometry, once the amino acid sequence is determined, tandem mass spectrometry (MS/MS) provides a straightforward way to translate candidate markers into a sensitive and selective multiplexed SRM assay that provides absolute quantification.

The broad aim of this study is to discover and qualify CSF AD biochemical markers in two independent cohorts. To demonstrate that our feature-based approach predicts candidate markers that are reproducible, we set up a prospective experimental design that focused on testing a limited number of features (e.g. two) in blinded follow up experiments. A cross sectional cohort was used for discovery experiments and allowed us to establish predefined hypotheses for two candidate markers; VGF (SME1) and NPTXR (SME2). Given, however, the sample size of our discovery experiment was limited to N = 10 AD and N = 10 control by sample availability (Table 1), we were concerned there may be a high false discovery rate. For an open platform, it is known that a small sample size can lead to false discovery and false negative results, particularly as the biological variation in the disease population tested is unknown. Rather than adjusting for multiple testing in our discovery experiment or performing a post-hoc analysis of two large datasets, we chose to pursue a conservative approach and selected just two markers, using pragmatic selection criteria (described earlier) for further testing in an independent cohort. It is important to note that we chose only two candidate markers and tested only this one hypothesis. Notably, significant reductions in both markers replicate in the second cohort and tracked with disease progression, demonstrating that the feature based dMS approach predicts reproducible candidate markers. Indeed, we anticipate there may be other reproducible candidate CSF markers in the Cohort-1 dataset.

Interestingly, both candidate markers, a peptide from NPTXR and a peptide from neurosecretory protein VGF, are derived from proteins thought to play a role in synaptic plasticity and function. NPTXR is a member of the neuronal pentraxin family (NP) that includes neuronal pentraxin 1 (NP1) and neuronal pentraxin 2 (NP2) also called Narp[40]. These three neuronal pentraxins (NPs) have been proposed to represent a novel neuronal uptake pathway that may function during synapse formation and remodeling [41]. NPs show amino acid homology to c-reactive and acute phase proteins in the immune system and hypothesized to be involved in activity-dependent plasticity [42]. The neuronal pentraxins are expressed predominately in the central nervous system. NPTXR is enriched at excitatory synapses where it associates with AMPA-type glutamate receptors (AMPAR) and enhances synaptogenesis. NPTXR is known to undergo regulated cleavage by the matrix metalloprotease tumor necrosis factor-alpha converting enzyme (TACE) [43] to release the pentraxin domain from the N-terminal transmembrane domain. NPTXR was found to be reduced in proteomic studies of CSF in patients with AD as compared to controls [34, 35]. Yin et al, however, reported NPTXR elevated in another similar study [44]. VGF is a 615 aa poly-peptide encoded by the *vgf* gene [45]. Originally cloned and characterized from NGF-induced differentiation of PC-12 cells towards a neuronal phenotype [46], expression of *vgf* is limited to a subset of neurons in the central and peripheral nervous systems and to specific populations of endocrine cells [45, 47]. VGF contains several dibasic sites and has the properties of a neuropeptide precursor though the exact identities of bioactive VGF derived peptides are unknown [45]. Knock out mice suggest VGF may also play a role in the regulation of energy balance [48]. VGF has been shown to enhance proliferation in hippocampal cells *in vitro* and *in vivo* and to produce antidepressant-like behavioral effects in animal models [49]. In cultured hippocampal neurons, VGF-derived peptides acutely enhance synaptic activity [50]. Using a chip based proteomic method, Carrette reported a 4.8kDa polypeptide decreased in AD as compared to control [30]. Subsequent purification and LC-QTOF based sequencing identified the sequence VGEEDEEAAEAEAEAEEAER corresponding to residues 378–397 of VGF[30]. Both NPTXR and VGF were found to be significantly different in a CSF of presymptomatic persons with familiar Alzheimer’s disease due to PSEN1 and APP mutations as compared to related non carriers by high resolution LC-MS [51].

Although the primary hypothesis in this experiment was to test VGF (SME1) and NPTXR (SME2) in the Cohort-2 longitudinal samples, it is interesting to perform a post-hoc exploratory analysis of the entire Cohort-2 longitudinal data set for additional potential markers that show a significant quantitative change with disease progression. Table 4 contains a list of 25 features that correspond to 20 distinct molecular weights, 18 additional distinct molecular weights other than SME1 and SME2, with molecular mass ranging from 909.5 to 3897.0 Da. In total, VGF, NPTXR and four additional features were sequenced by tandem mass spectrometry. Three identified peptides have a unique amino acid sequence that originates from chromagranin A [52], a secretory 48–53 kDA glycoprotein which is stored and released by neurons in regions relevant for AD [53]. Interestingly, using alternate bioanalytical techniques, CE-MS and 2D-DIGE, to profile CSF from neurodegenerative disorders and cognitively-healthy controls, Jahn et al [54] and Perrin et al [55], respectively, identified Chromagranin A as candidate marker for early stage Alzheimer’s Disease. Finely, one additional unique peptide from neuronal secretory protein VGF was identified (Feature 12 and 13, Table 4) from the list of 25 features, the identical VGEEDEEAAEAEAEAEEAER peptide identified by Carrette et al. [30]. Several possibilities may explain why only 6 of 19 features were successfully identified in this dataset of 25. First, although high resolution mass spectrometry allows one to resolve features that differ by less than 0.01 Da, the precursor ion selection step of an MS/MS experiment is typically 1 to 3 Da wide and limits one’s ability to sequence ions that are close in *m/z*. As a result, MS/MS spectra collected on complex samples, like CSF, often contain fragments derived from multiple peptides, and thus are difficult or impossible to interpret. Secondly, not all peptides fragment favorably under CAD conditions and thus do not yield interpretable MS/MS spectra. Moreover, the peptide may contain a metastable post translational modification (PTM) or unknown PTM. Finally, the molecular entity may be a lipid or another type of molecule and not a peptide. Enhanced methods for MS/MS based sequencing such as electron transfer dissociation provide new opportunities for sequencing previously unidentified features.

As anticipated, remarkable improvement in the analytical precision of the mass spectrometry assay is realized when moving from an open discovery platform, which provides relative quantitation without the requirement of an internal standard, to the targeted SRM assay, which draws on a stable isotope labeled protein internal standard for improved precision and absolute quantification. In two independent studies, Choi at al [56] and Percey et al [57] have shown multiplexed MRM with internal standards for candidate protein biomarker quantitation in CSF. For VGF (SME1) and NPTXR (SME2) shown here, the technical variability or analytical precision improves from a % CV of approximately 45% to a CV of less than 10%. These results clearly demonstrate the straightforwardness for translating from the dMS discovery-based mass spectrometry platform that provides relative label-free quantitation, to an SRM-based clinical grade analytical platform or targeted MS/MS that provides absolute quantification with high precision. In addition, the likelihood for success to convert from one platform (dMS) to another (LC-SRM or LC-MS/MS) is high and the time to convert is minimal, limited primarily by the time it takes to generate the stable isotope labeled internal standard.

Recently, Wildsmith et al developed a targeted-proteomics multiple reaction monitoring assay for the quantitation of 39 peptides derived from 30 proteins in longitudinal CSF samples (Wildsmith et al 2014[58]). Using the AQUA approach ([59]), where stable-isotope peptides for each candidate-peptide were synthesized and used, these authors report an impressive interassay CVs less of less than 20% for all 30 proteins. Interestingly, of the proteins tested in the multiplexed targeted-proteomic assay, 4 proteins showed significant declines in the longitudinal CSF samples including NPTXR and CgA strongly supporting the findings reported here. NPTX and CgA declined approximately 10%/year in the AD patients but not in the aged control or MCI patients ([58]). VGF, however, was not tested in the Wildsmith study.

Careful consideration is needed in the final interpretation of this data set. Although the discovery proteomics technology to explore global biochemical changes in CSF, followed up with a triple quadrupole mass spectrometry based SRM assay to provide absolute quantification and high precision, will be helpful in the development of protein markers, additional work remains to explore these and other markers for utility in decision making. The concept of "fit-for-purpose" biomarker qualification for drug development and decision making has been discussed [60]. Aligned with these concepts, further evaluation of samples from individuals with related or similar diseases such as frontal temporal dementia, vascular dementia and Parkinson's disease along with additional cohorts need to be studied. Moreover, studies to look at co-morbidities and concurrent medications need to be performed. In addition, samples in these two studies were drawn from the same geographic region and thus future studies are needed in geographic and ethnically diverse populations. Given the large difference in APOE4 allele frequency in the AD and Control populations, differences in VGF (SME1) and NPTXR (SME2) may prove to be as related to presence or absence of APOE4 as to the diagnosis of AD. Nonetheless, proteins in CSF that change at 8–10% per year are promising candidates that alone may be useful to track AD progression over time and may be complimentary with other AD disease progression markers such as volumetric imaging, contrast agents coupled with positron emission tomography (PET) imaging and the established biochemical markers such as Ab42, tau and p-tau. Reference blennow [61] Such data may be used to design short and small clinical trials to test novel disease modifying therapies. For mild to moderate AD, additional CSF discovery experiments may be required as there may be biochemical markers in CSF in early disease that may not be picked up in a discovery experiment in late disease.

Collectively these results demonstrate the utility of high resolution differential mass spectrometry discovery proteomics and suggest biochemical CSF markers of AD for further qualification. Interestingly, neurosecretory protein VGF (SME-1) and NPTXR (SME-2) are expressed almost exclusively in the central nervous system, and are reasonable proteins to test in plasma as blood based marker for AD.

## Supporting Information

S1 Fig Linear mixed-effect model analysis of (A) SME1 and (B) SME2 in the longitudinal cohort, Cohort-2, group slope analysis. Area under the curve (AUC) intensity measurements by dMS are shown on log scale. Serial samples taken annually, are plotted years since first visit. The solid line represents the group slope.
(PDF)

S2 Fig Linear mixed-effect model analysis of SME1 in the longitudinal cohort, Cohort-2, individual patients (A) AD, (B) CTL. Area under the curve (AUC) intensity measurements by dMS are shown on log scale. Serial samples taken annually (shown in blue and range from two to seven serial draws) are plotted years since first visit. The solid blue line represents the group slope. Patient number is shown above patient data.
(PDF)

S3 Fig Linear mixed-effect model analysis of SME2 in the longitudinal cohort, Cohort-2, individual patients (A) AD, (B) CTL. Area under the curve (AUC) intensity measurements by dMS are shown on log scale. Serial samples taken annually (shown in blue and range from two to seven serial draws), are plotted years since first visit. The solid line represents the group slope. Patient number is shown above patient data.
(PDF)

S1 Table Interwoven block design for cross sectional study (Cohort-1). (PDF)

S2 Table Cohort-1 Random Forest Classifier, top 100 features. (PDF)

S3 Table Interwoven block design for longitudinal study (Cohort-2). (PDF)

S4 Table SME 1 and SME 2 AUCs in Cohort-2. (PDF)

S5 Table Cohort-2 linear mixed-effect model analysis 8108 features. (PDF)

## References

[1] Smith AD. Imaging the progression of Alzheimer pathology through the brain. Proceedings of the National Academy of Sciences of the United States of America. 2002;99(7):4135–7. 10.1073/pnas.082107399 11929987 PMC123611

[2] Selkoe DJ. Alzheimer's disease is a synaptic failure. Science. 2002;298(5594):789–91. 10.1126/science.1074069 . 12399581

[3] Mount C, Downton C. Alzheimer disease: progress or profit? Nature medicine. 2006;12(7):780–4. Epub 2006/07/11. 10.1038/nm0706-780 . 16829947

[4] Citron M. Alzheimer's disease: strategies for disease modification. Nature reviews Drug discovery. 2010;9(5):387–98. Epub 2010/05/01. 10.1038/nrd2896 . 20431570

[5] Brookmeyer R, Gray S, Kawas C. Projections of Alzheimer's disease in the United States and the public health impact of delaying disease onset. American journal of public health. 1998;88(9):1337–42. Epub 1998/09/16. 9736873 10.2105/ajph.88.9.1337 PMC1509089

[6] Ballatore C, Lee VM, Trojanowski JQ. Tau-mediated neurodegeneration in Alzheimer's disease and related disorders. Nature reviews Neuroscience. 2007;8(9):663–72. Epub 2007/08/09. 10.1038/nrn2194 . 17684513

[7] Blennow K, Hampel H, Weiner M, Zetterberg H. Cerebrospinal fluid and plasma biomarkers in Alzheimer disease. Nature reviews Neurology. 2010;6(3):131–44. Epub 2010/02/17. 10.1038/nrneurol.2010.4 . 20157306

[8] Shaw LM, Vanderstichele H, Knapik-Czajka M, Clark CM, Aisen PS, Petersen RC, et al Cerebrospinal fluid biomarker signature in Alzheimer's disease neuroimaging initiative subjects. Annals of neurology. 2009;65(4):403–13. Epub 2009/03/20. 10.1002/ana.21610 19296504 PMC2696350

[9] Tapiola T, Alafuzoff I, Herukka SK, Parkkinen L, Hartikainen P, Soininen H, et al Cerebrospinal fluid {beta}-amyloid 42 and tau proteins as biomarkers of Alzheimer-type pathologic changes in the brain. Archives of neurology. 2009;66(3):382–9. Epub 2009/03/11. 10.1001/archneurol.2008.596 . 19273758

[10] Beckett LA, Harvey DJ, Gamst A, Donohue M, Kornak J, Zhang H, et al The Alzheimer's Disease Neuroimaging Initiative: Annual change in biomarkers and clinical outcomes. Alzheimer's & dementia: the journal of the Alzheimer's Association. 2010;6(3):257–64. Epub 2010/05/11. 10.1016/j.jalz.2010.03.002 20451874 PMC2867839

[11] Breteler MM. Mapping out biomarkers for Alzheimer disease. JAMA: the journal of the American Medical Association. 2011;305(3):304–5. Epub 2011/01/20. 10.1001/jama.2010.2017 . 21245188

[12] Choi YS, Choe LH, Lee KH. Recent cerebrospinal fluid biomarker studies of Alzheimer's disease. Expert review of proteomics. 2010;7(6):919–29. Epub 2010/12/15. 10.1586/epr.10.75 . 21142892

[13] Vemuri P, Wiste HJ, Weigand SD, Shaw LM, Trojanowski JQ, Weiner MW, et al MRI and CSF biomarkers in normal, MCI, and AD subjects: diagnostic discrimination and cognitive correlations. Neurology. 2009;73(4):287–93. Epub 2009/07/29. 10.1212/WNL.0b013e3181af79e5 19636048 PMC2715210

[14] Lee AY, Paweletz CP, Pollock RM, Settlage RE, Cruz JC, Secrist JP, et al Quantitative analysis of histone deacetylase-1 selective histone modifications by differential mass spectrometry. Journal of proteome research. 2008;7(12):5177–86. Epub 2009/04/16. 10.1021/pr800510p . 19367703

[15] Wiener MC, Sachs JR, Deyanova EG, Yates NA. Differential mass spectrometry: a label-free LC-MS method for finding significant differences in complex peptide and protein mixtures. Analytical chemistry. 2004;76(20):6085–96. Epub 2004/10/16. 10.1021/ac0493875 . 15481957

[16] Meng F, Wiener MC, Sachs JR, Burns C, Verma P, Paweletz CP, et al Quantitative analysis of complex peptide mixtures using FTMS and differential mass spectrometry. Journal of the American Society for Mass Spectrometry. 2007;18(2):226–33. Epub 2006/10/31. 10.1016/j.jasms.2006.09.014 . 17070068

[17] Zhao X, Deyanova EG, Lubbers LS, Zafian P, Li JJ, Liaw A, et al Differential mass spectrometry of rat plasma reveals proteins that are responsive to 17beta-estradiol and a selective estrogen receptor modulator PPT. Journal of proteome research. 2008;7(10):4373–83. Epub 2008/09/13. 10.1021/pr800309z . 18785765

[18] Paweletz CP, Wiener MC, Bondarenko AY, Yates NA, Song Q, Liaw A, et al Application of an end-to-end biomarker discovery platform to identify target engagement markers in cerebrospinal fluid by high resolution differential mass spectrometry. Journal of proteome research. 2010;9(3):1392–401. Epub 2010/01/26. 10.1021/pr900925d . 20095649

[19] Clarke R, Smith AD, Jobst KA, Refsum H, Sutton L, Ueland PM. Folate, vitamin B12, and serum total homocysteine levels in confirmed Alzheimer disease. Archives of neurology. 1998;55(11):1449–55. Epub 1998/11/21. . 9823829 10.1001/archneur.55.11.1449

[20] Jobst KA, Barnetson LP, Shepstone BJ. Accurate prediction of histologically confirmed Alzheimer's disease and the differential diagnosis of dementia: the use of NINCDS-ADRDA and DSM-III-R criteria, SPECT, X-ray CT, and Apo E4 in medial temporal lobe dementias. Oxford Project to Investigate Memory and Aging. International psychogeriatrics / IPA. 1998;10(3):271–302. Epub 1998/10/24. . 9785148 10.1017/s1041610298005389

[21] Hindley NJ, Jobst KA, King E, Barnetson L, Smith A, Haigh AM. High acceptability and low morbidity of diagnostic lumbar puncture in elderly subjects of mixed cognitive status. Acta neurologica Scandinavica. 1995;91(5):405–11. Epub 1995/05/01. . 7639073 10.1111/j.1600-0404.1995.tb07029.x

[22] McKhann G, Drachman D, Folstein M, Katzman R, Price D, Stadlan EM. Clinical diagnosis of Alzheimer's disease: report of the NINCDS-ADRDA Work Group under the auspices of Department of Health and Human Services Task Force on Alzheimer's Disease. Neurology. 1984;34(7):939–44. Epub 1984/07/01. . 6610841 10.1212/wnl.34.7.939

[23] Svetnik V, Liaw A, Tong C, Culberson JC, Sheridan RP, Feuston BP. Random forest: a classification and regression tool for compound classification and QSAR modeling. Journal of chemical information and computer sciences. 2003;43(6):1947–58. Epub 2003/11/25. 10.1021/ci034160g . 14632445

[24] Elias JE, Haas W, Faherty BK, Gygi SP. Comparative evaluation of mass spectrometry platforms used in large-scale proteomics investigations. Nature methods. 2005;2(9):667–75. Epub 2005/08/25. 10.1038/nmeth785 . 16118637

[25] Keller A, Nesvizhskii AI, Kolker E, Aebersold R. Empirical statistical model to estimate the accuracy of peptide identifications made by MS/MS and database search. Analytical chemistry. 2002;74(20):5383–92. Epub 2002/10/31. . 12403597 10.1021/ac025747h

[26] Nesvizhskii AI, Keller A, Kolker E, Aebersold R. A statistical model for identifying proteins by tandem mass spectrometry. Analytical chemistry. 2003;75(17):4646–58. Epub 2003/11/25. . 14632076 10.1021/ac0341261

[27] Williams JH, Wilcock GK, Seeburger J, Dallob A, Laterza O, Potter W, et al Non-linear relationships of cerebrospinal fluid biomarker levels with cognitive function: an observational study. Alzheimer's research & therapy. 2011;3(1):5 Epub 2011/02/19. 10.1186/alzrt64 21329517 PMC3109414

[28] Lam FCaL, M. T.. A modified Wilcoxon rank sum test for paired data. Biometrika. 1983;70:3.

[29] Ransohoff DF. Rules of evidence for cancer molecular-marker discovery and validation. Nature reviews Cancer. 2004;4(4):309–14. Epub 2004/04/02. 10.1038/nrc1322 . 15057290

[30] Carrette O, Demalte I, Scherl A, Yalkinoglu O, Corthals G, Burkhard P, et al A panel of cerebrospinal fluid potential biomarkers for the diagnosis of Alzheimer's disease. Proteomics. 2003;3(8):1486–94. Epub 2003/08/19. 10.1002/pmic.200300470 . 12923774

[31] Simonsen AH, McGuire J, Podust VN, Davies H, Minthon L, Skoog I, et al Identification of a novel panel of cerebrospinal fluid biomarkers for Alzheimer's disease. Neurobiology of aging. 2008;29(7):961–8. Epub 2007/02/27. 10.1016/j.neurobiolaging.2007.01.011 . 17321007

[32] Castano EM, Roher AE, Esh CL, Kokjohn TA, Beach T. Comparative proteomics of cerebrospinal fluid in neuropathologically-confirmed Alzheimer's disease and non-demented elderly subjects. Neurological research. 2006;28(2):155–63. Epub 2006/03/23. 10.1179/016164106x98035 . 16551433

[33] Davidsson P, Westman-Brinkmalm A, Nilsson CL, Lindbjer M, Paulson L, Andreasen N, et al Proteome analysis of cerebrospinal fluid proteins in Alzheimer patients. Neuroreport. 2002;13(5):611–5. Epub 2002/04/26. . 11973456 10.1097/00001756-200204160-00015

[34] Finehout EJ, Franck Z, Choe LH, Relkin N, Lee KH. Cerebrospinal fluid proteomic biomarkers for Alzheimer's disease. Annals of neurology. 2007;61(2):120–9. Epub 2006/12/15. 10.1002/ana.21038 . 17167789

[35] Hu Y, Hosseini A, Kauwe JS, Gross J, Cairns NJ, Goate AM, et al Identification and validation of novel CSF biomarkers for early stages of Alzheimer's disease. Proteomics Clinical applications. 2007;1(11):1373–84. Epub 2007/11/01. 10.1002/prca.200600999 . 21136637

[36] Puchades M, Hansson SF, Nilsson CL, Andreasen N, Blennow K, Davidsson P. Proteomic studies of potential cerebrospinal fluid protein markers for Alzheimer's disease. Brain research Molecular brain research. 2003;118(1–2):140–6. Epub 2003/10/16. . 14559363 10.1016/j.molbrainres.2003.08.005

[37] Abdi F, Quinn JF, Jankovic J, McIntosh M, Leverenz JB, Peskind E, et al Detection of biomarkers with a multiplex quantitative proteomic platform in cerebrospinal fluid of patients with neurodegenerative disorders. Journal of Alzheimer's disease: JAD. 2006;9(3):293–348. Epub 2006/08/18. . 16914840 10.3233/jad-2006-9309

[38] Liu H, Sadygov RG, Yates JR 3rd. A model for random sampling and estimation of relative protein abundance in shotgun proteomics. Analytical chemistry. 2004;76(14):4193–201. Epub 2004/07/16. 10.1021/ac0498563 . 15253663

[39] Sadygov RG, Liu H, Yates JR. Statistical models for protein validation using tandem mass spectral data and protein amino acid sequence databases. Analytical chemistry. 2004;76(6):1664–71. Epub 2004/03/17. 10.1021/ac035112y . 15018565

[40] Dodds DC, Omeis IA, Cushman SJ, Helms JA, Perin MS. Neuronal pentraxin receptor, a novel putative integral membrane pentraxin that interacts with neuronal pentraxin 1 and 2 and taipoxin-associated calcium-binding protein 49. The Journal of biological chemistry. 1997;272(34):21488–94. Epub 1997/08/22. . 9261167 10.1074/jbc.272.34.21488

[41] Kirkpatrick LL, Matzuk MM, Dodds DC, Perin MS. Biochemical interactions of the neuronal pentraxins. Neuronal pentraxin (NP) receptor binds to taipoxin and taipoxin-associated calcium-binding protein 49 via NP1 and NP2. The Journal of biological chemistry. 2000;275(23):17786–92. Epub 2000/04/05. 10.1074/jbc.M002254200 . 10748068

[42] Bjartmar L, Huberman AD, Ullian EM, Renteria RC, Liu X, Xu W, et al Neuronal pentraxins mediate synaptic refinement in the developing visual system. The Journal of neuroscience: the official journal of the Society for Neuroscience. 2006;26(23):6269–81. Epub 2006/06/10. 10.1523/jneurosci.4212-05.2006 16763034 PMC2579897

[43] Cho RW, Park JM, Wolff SB, Xu D, Hopf C, Kim JA, et al mGluR1/5-dependent long-term depression requires the regulated ectodomain cleavage of neuronal pentraxin NPR by TACE. Neuron. 2008;57(6):858–71. Epub 2008/03/28. 10.1016/j.neuron.2008.01.010 18367087 PMC2701195

[44] Yin GN, Lee HW, Cho JY, Suk K. Neuronal pentraxin receptor in cerebrospinal fluid as a potential biomarker for neurodegenerative diseases. Brain research. 2009;1265:158–70. Epub 2009/04/17. 10.1016/j.brainres.2009.01.058 . 19368810

[45] Levi A, Ferri GL, Watson E, Possenti R, Salton SR. Processing, distribution, and function of VGF, a neuronal and endocrine peptide precursor. Cellular and molecular neurobiology. 2004;24(4):517–33. Epub 2004/07/06. . 15233376 10.1023/B:CEMN.0000023627.79947.22 PMC11529936

[46] Levi A, Eldridge JD, Paterson BM. Molecular cloning of a gene sequence regulated by nerve growth factor. Science. 1985;229(4711):393–5. Epub 1985/07/26. . 3839317 10.1126/science.3839317

[47] Canu N, Possenti R, Ricco AS, Rocchi M, Levi A. Cloning, structural organization analysis, and chromosomal assignment of the human gene for the neurosecretory protein VGF. Genomics. 1997;45(2):443–6. Epub 1997/11/05. 10.1006/geno.1997.4945 . 9344675

[48] Hahm S, Mizuno TM, Wu TJ, Wisor JP, Priest CA, Kozak CA, et al Targeted deletion of the Vgf gene indicates that the encoded secretory peptide precursor plays a novel role in the regulation of energy balance. Neuron. 1999;23(3):537–48. Epub 1999/08/05. . 10433265 10.1016/s0896-6273(00)80806-5

[49] Thakker-Varia S, Krol JJ, Nettleton J, Bilimoria PM, Bangasser DA, Shors TJ, et al The neuropeptide VGF produces antidepressant-like behavioral effects and enhances proliferation in the hippocampus. The Journal of neuroscience: the official journal of the Society for Neuroscience. 2007;27(45):12156–67. Epub 2007/11/09. 10.1523/jneurosci.1898-07.2007 17989282 PMC3363962

[50] Alder J, Thakker-Varia S, Bangasser DA, Kuroiwa M, Plummer MR, Shors TJ, et al Brain-derived neurotrophic factor-induced gene expression reveals novel actions of VGF in hippocampal synaptic plasticity. The Journal of neuroscience: the official journal of the Society for Neuroscience. 2003;23(34):10800–8. Epub 2003/12/03. 14645472 10.1523/JNEUROSCI.23-34-10800.2003 PMC3374594

[51] Ringman JM, Schulman H, Becker C, Jones T, Bai Y, Immermann F, et al Proteomic changes in cerebrospinal fluid of presymptomatic and affected persons carrying familial Alzheimer disease mutations. Archives of neurology. 2012;69(1):96–104. Epub 2012/01/11. 10.1001/archneurol.2011.642 22232349 PMC3632731

[52] Blaschko H, Comline RS, Schneider FH, Silver M, Smith AD. Secretion of a chromaffin granule protein, chromogranin, from the adrenal gland after splanchnic stimulation. Nature. 1967;215(5096):58–9. . 6053402 10.1038/215058a0

[53] Somogyi P, Hodgson AJ, DePotter RW, Fischer-Colbrie R, Schober M, Winkler H, et al Chromogranin immunoreactivity in the central nervous system. Immunochemical characterisation, distribution and relationship to catecholamine and enkephalin pathways. Brain research. 1984;320(2–3):193–230. Epub 1984/12/01. . 6084534 10.1016/0165-0173(84)90007-9

[54] Jahn H, Wittke S, Zurbig P, Raedler TJ, Arlt S, Kellmann M, et al Peptide fingerprinting of Alzheimer's disease in cerebrospinal fluid: identification and prospective evaluation of new synaptic biomarkers. PloS one. 2011;6(10):e26540 10.1371/journal.pone.0026540 22046305 PMC3202544

[55] Perrin RJ, Craig-Schapiro R, Malone JP, Shah AR, Gilmore P, Davis AE, et al Identification and validation of novel cerebrospinal fluid biomarkers for staging early Alzheimer's disease. PloS one. 2011;6(1):e16032 10.1371/journal.pone.0016032 21264269 PMC3020224

[56] Choi YS, Hou S, Choe LH, Lee KH. Targeted human cerebrospinal fluid proteomics for the validation of multiple Alzheimer's disease biomarker candidates. Journal of chromatography B, Analytical technologies in the biomedical and life sciences. 2013;930:129–35. 10.1016/j.jchromb.2013.05.003 23735279 PMC3710693

[57] Percy AJ, Yang J, Chambers AG, Simon R, Hardie DB, Borchers CH. Multiplexed MRM with Internal Standards for Cerebrospinal Fluid Candidate Protein Biomarker Quantitation. Journal of proteome research. 2014 10.1021/pr500317d . 24911472

[58] Wildsmith KR, Schauer SP, Smith AM, Arnott D, Zhu Y, Haznedar J, et al Identification of longitudinally dynamic biomarkers in Alzheimer's disease cerebrospinal fluid by targeted proteomics. Molecular neurodegeneration. 2014;9:22 10.1186/1750-1326-9-22 24902845 PMC4061120

[59] Gerber SA, Rush J, Stemman O, Kirschner MW, Gygi SP. Absolute quantification of proteins and phosphoproteins from cell lysates by tandem MS. Proceedings of the National Academy of Sciences of the United States of America. 2003;100(12):6940–5. 10.1073/pnas.0832254100 12771378 PMC165809

[60] Wagner JA. Strategic approach to fit-for-purpose biomarkers in drug development. Annual review of pharmacology and toxicology. 2008;48:631–51. Epub 2007/10/17. 10.1146/annurev.pharmtox.48.113006.094611 . 17937595

[61] Mattsson N, Portelius E, Rolstad S, Gustavsson M, Andreasson U, Stridsberg M, et al Longitudinal Cerebrospinal Fluid Biomarkers over Four Years in Mild Cognitive Impairment. Journal of Alzheimers Disease. 2012;30(4):767–78. 10.3233/Jad-2012-120019 . 22475796

