# Supplementary figures and images for: High Resolution Discovery Proteomics Reveals Candidate Disease Progression Markers of Alzheimer’s Disease in Human Cerebrospinal Fluid
Source: PLoS One. 2015 Aug 13;10(8):e0135365. doi: 10.1371/journal.pone.0135365 (PMC4535975; doi:10.1371/journal.pone.0135365)

SME1

751080736: Est. Change (AD)=-10.9% (p=0.026), Diff. from CTL p=0.023

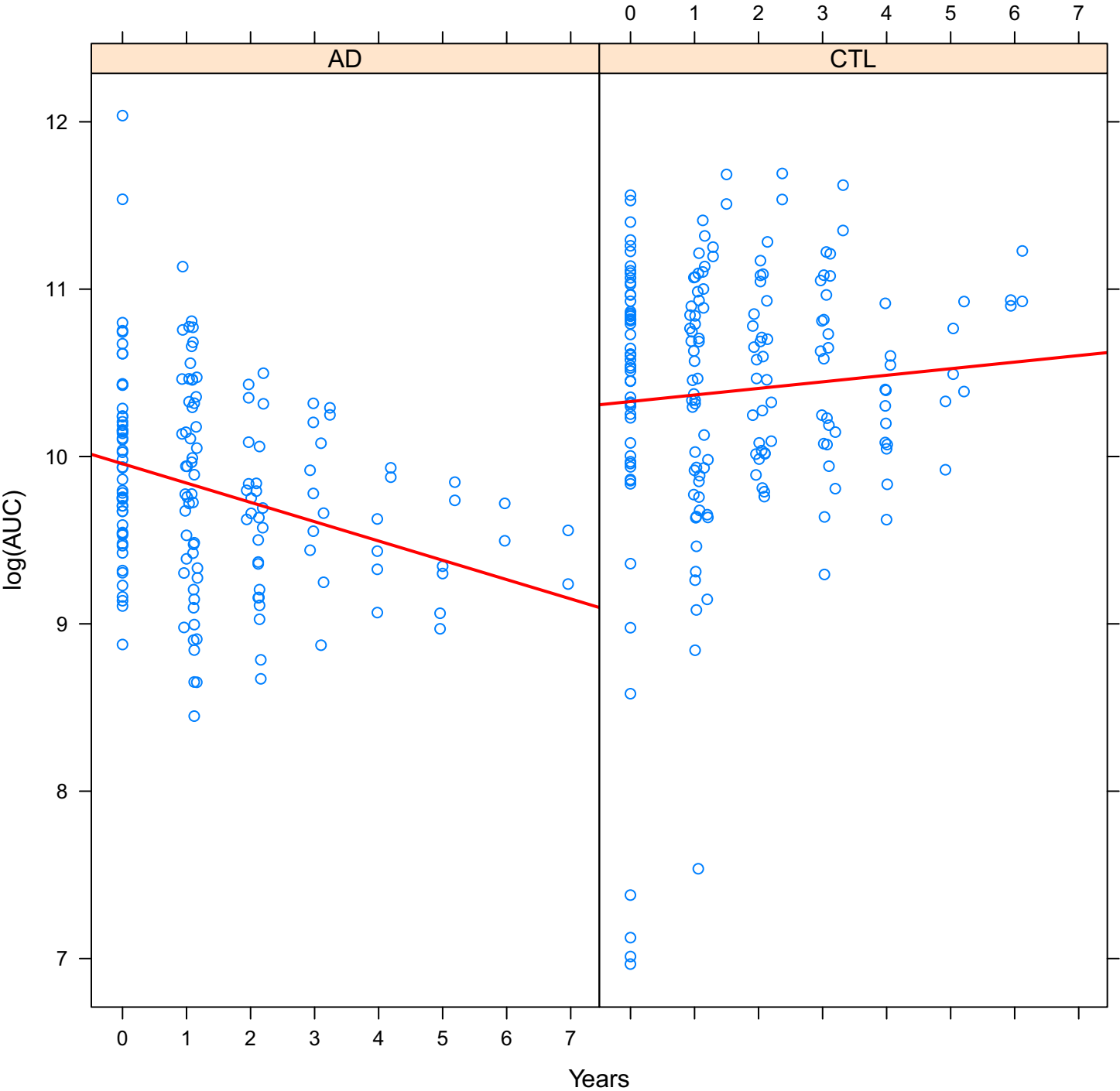

## SME2

**751082516: Est. Change (AD)=-6.9% (p=0.039), Diff. from CTL p=0.016**

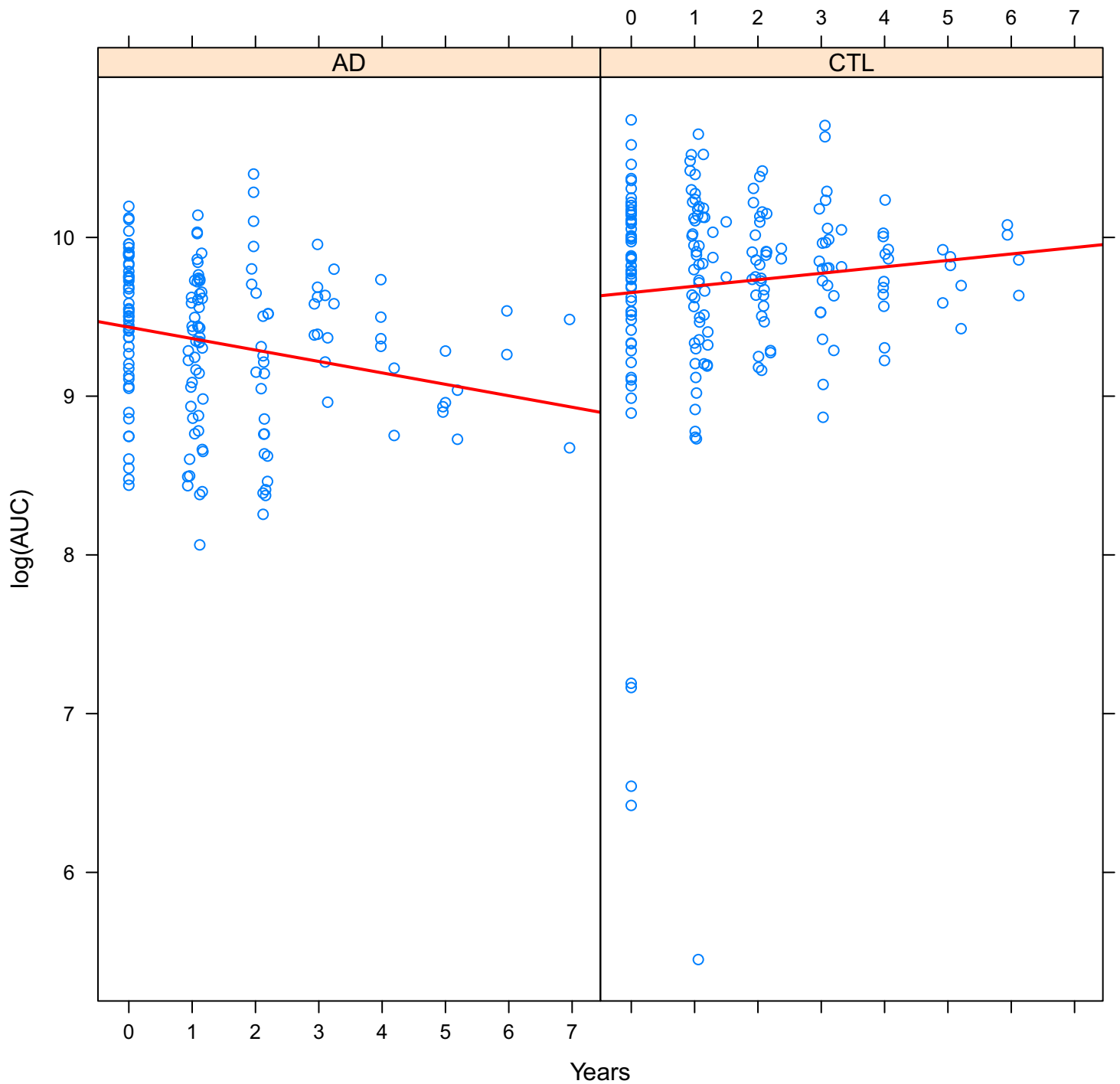

Supplement: S1 Fig — Area under the curve (AUC) intensity measurements by dMS are shown on log scale. Serial samples taken annually, are plotted years since first visit. The solid line represents the group slope. (PDF) [file pone.0135365.s001.pdf]

### 751080736, AD

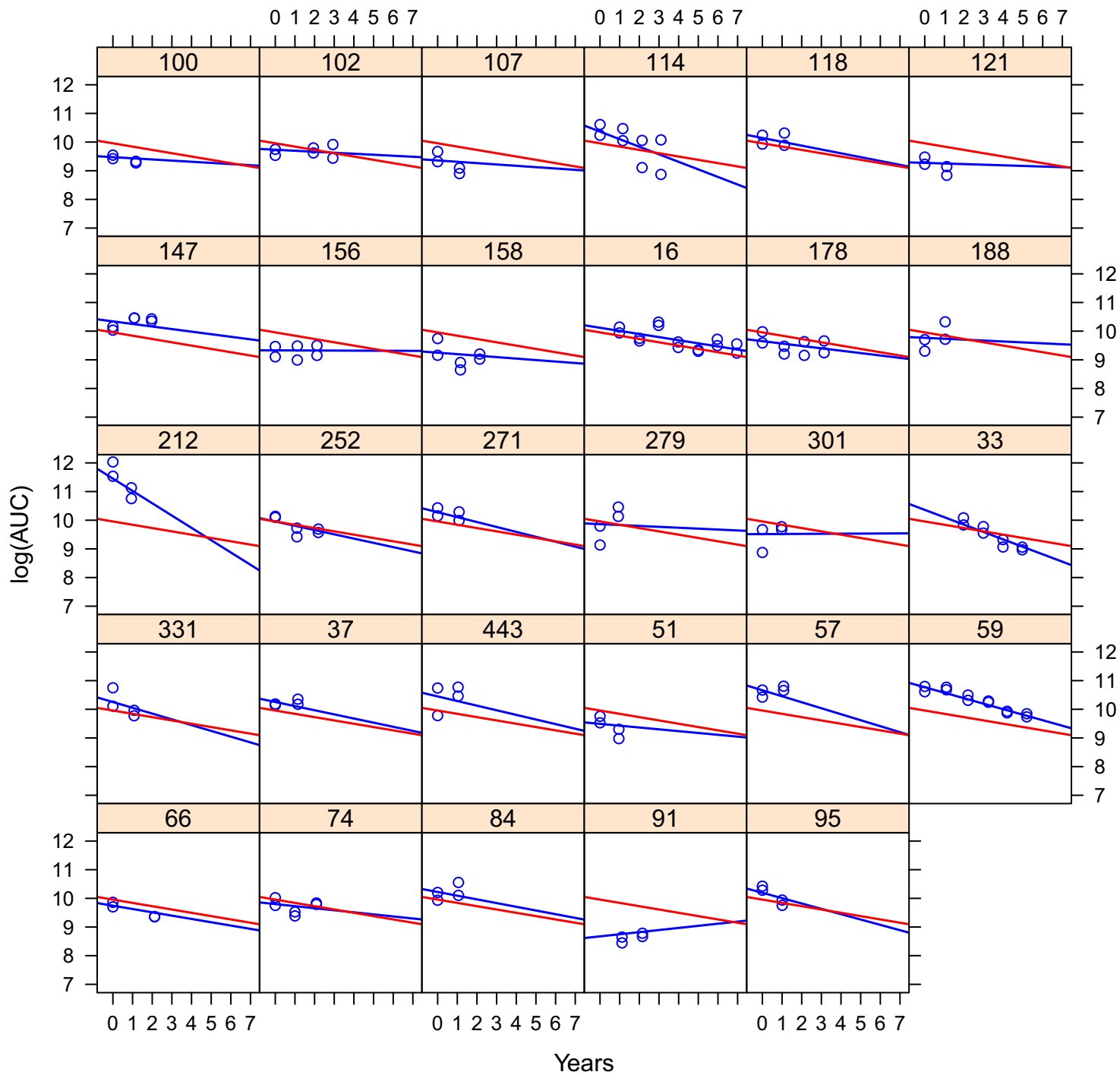

### 751080736, CTL

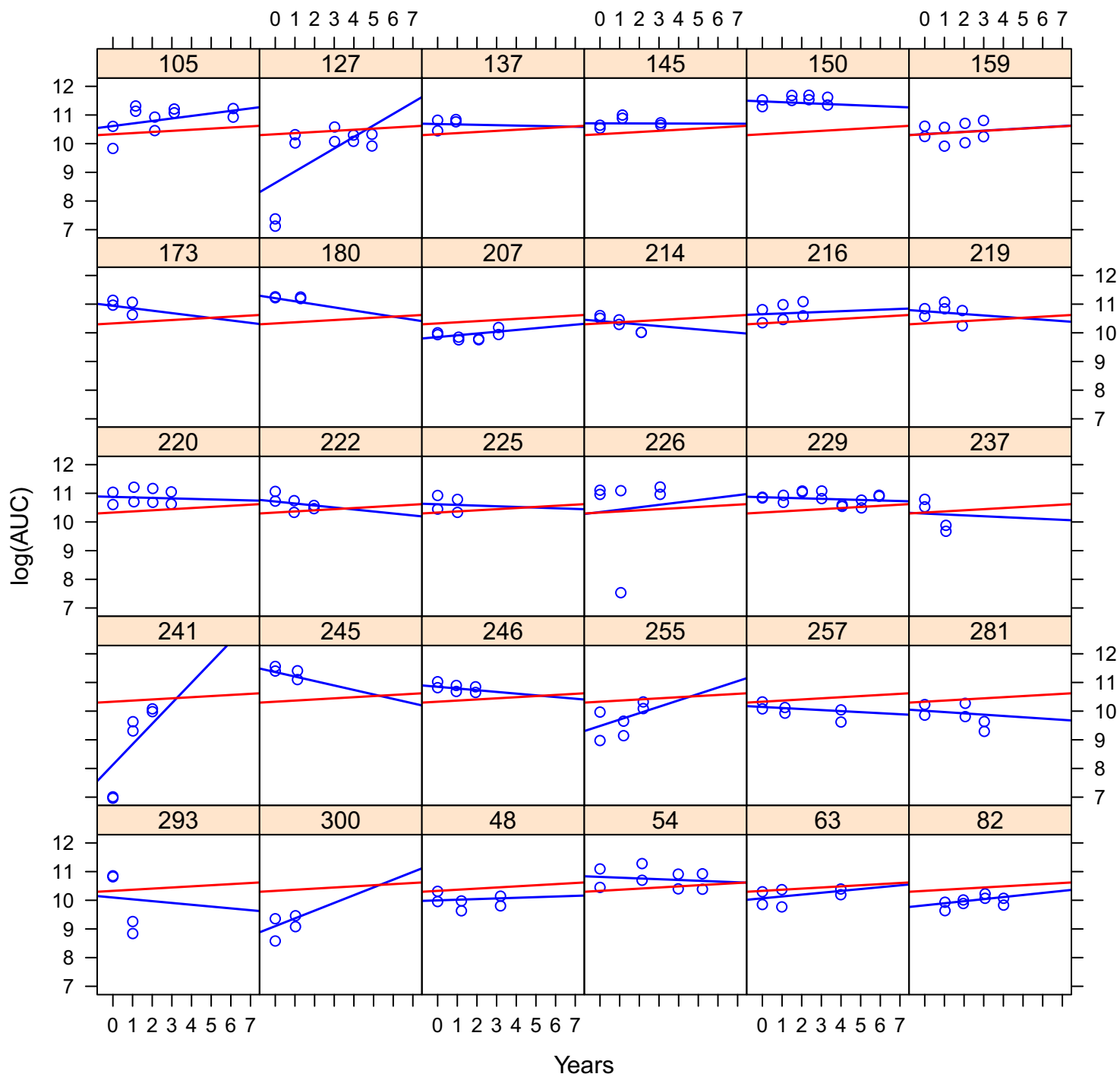

Supplement: S2 Fig — Area under the curve (AUC) intensity measurements by dMS are shown on log scale. Serial samples taken annually (shown in blue and range from two to seven serial draws) are plotted years since first visit. The solid blue line represents the group slope. Patient number is shown above patient data. (PDF) [file pone.0135365.s002.pdf]

751082516, AD

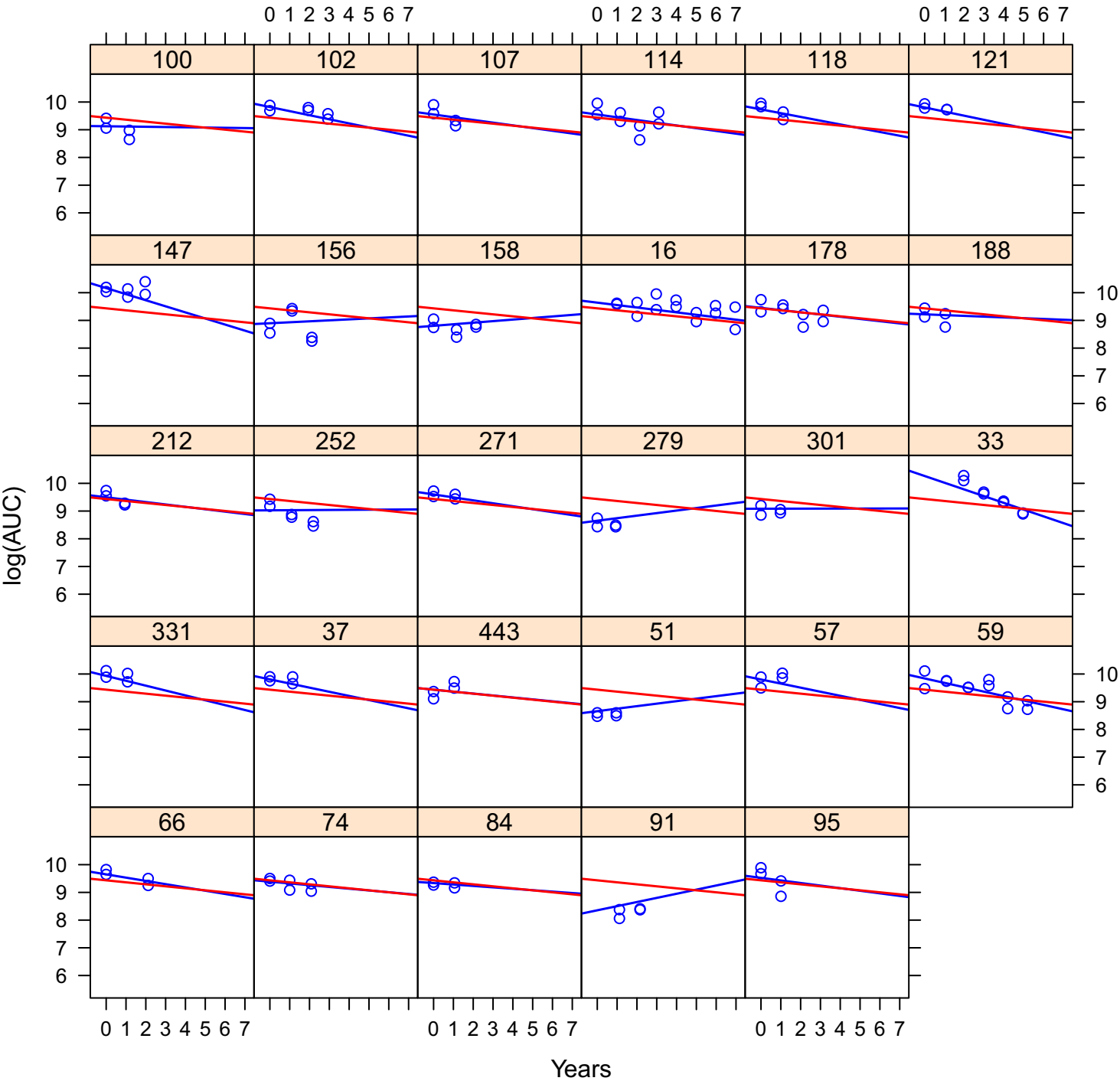

# 751082516, CTL

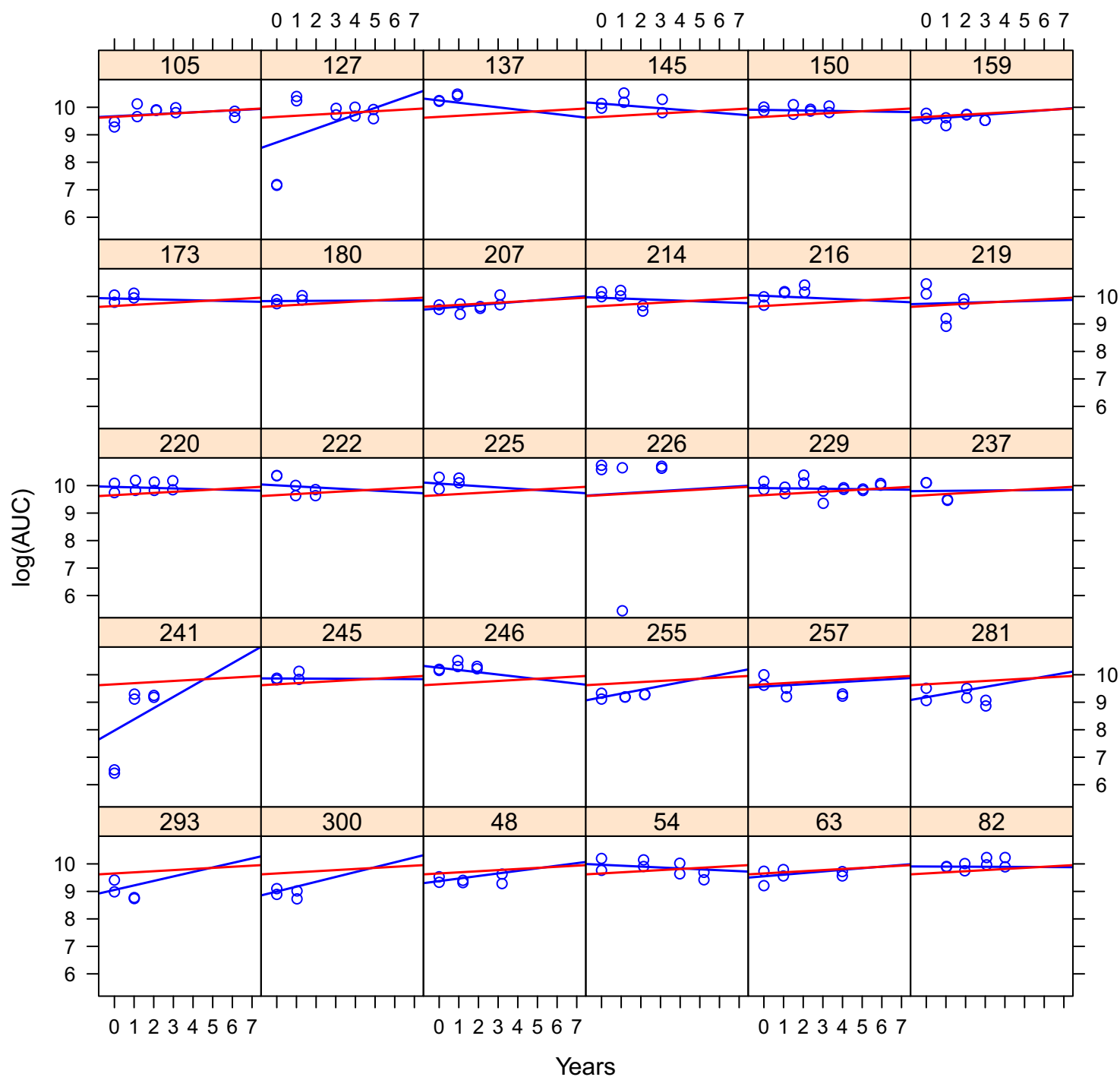

Supplement: S3 Fig — Area under the curve (AUC) intensity measurements by dMS are shown on log scale. Serial samples taken annually (shown in blue and range from two to seven serial draws), are plotted years since first visit. The solid line represents the group slope. Patient number is shown above patient data. (PDF) [file pone.0135365.s003.pdf]
