# Supplementary material for: High Resolution Discovery Proteomics Reveals Candidate Disease Progression Markers of Alzheimer’s Disease in Human Cerebrospinal Fluid
Source: PLoS One. 2015 Aug 13;10(8):e0135365. doi: 10.1371/journal.pone.0135365 (PMC4535975; doi:10.1371/journal.pone.0135365)
Supplement: S1 Table — (PDF) [file pone.0135365.s004.pdf]

S1 Table Interwoven block design for cross sectional study (Cohort-1)

LC-MS Injection Order	LC-MS Block	Sample Type	Description	Blinded Sample Name	Biochem Block	IMD Column	Acquisition Date	File Name
1	1	Study Sample	OPTIMA Phase I, OPP 1, replicate 1	OPP 1	1	2	3-Aug-05	157753
2	1	Study Sample	OPTIMA Phase I, OPP 2, replicate 1	OPP 2	1	4	3-Aug-05	157754
3	1	Study Sample	OPTIMA Phase I, OPP 3, replicate 1	OPP 3	1	5	3-Aug-05	157755
4	1	Study Sample	OPTIMA Phase I, OPP 4, replicate 1	OPP 4	1	6	4-Aug-05	157756
5	1	Study Sample	OPTIMA Phase I, OPP 5, replicate 1	OPP 5	1	1	4-Aug-05	157757
6	1	QC	OPTIMA Phase I, CSF ctrl 2, replicate 1	CSF ctrl 2	1	2	4-Aug-05	157758
7	1	Study Sample	OPTIMA Phase I, OPP 6, replicate 1	OPP 6	1	3	4-Aug-05	157759
8	1	Study Sample	OPTIMA Phase I, OPP 7, replicate 1	OPP 7	1	5	4-Aug-05	157760
9	1	Study Sample	OPTIMA Phase I, OPP 8, replicate 1	OPP 8	1	6	4-Aug-05	157761
10	1	Study Sample	OPTIMA Phase I, OPP 9, replicate 1	OPP 9	1	1	4-Aug-05	157762
11	1	Study Sample	OPTIMA Phase I, OPP 10, replicate 1	OPP 10	1	2	4-Aug-05	157763
12	1	QC	OPTIMA Phase I, CSF ctrl 3, replicate 1	CSF ctrl 3	1	3	4-Aug-05	157764
13	1	Study Sample	OPTIMA Phase I, OPP 11, replicate 1	OPP 11	1	4	4-Aug-05	157765
14	1	Study Sample	OPTIMA Phase I, OPP 12, replicate 1	OPP 12	1	6	4-Aug-05	157766
15	1	Study Sample	OPTIMA Phase I, OPP 13, replicate 1	OPP 13	1	2	4-Aug-05	157767
16	1	Study Sample	OPTIMA Phase I, OPP 14, replicate 1	OPP 14	1	3	4-Aug-05	157768
17	1	Study Sample	OPTIMA Phase I, OPP 15, replicate 1	OPP 15	1	5	4-Aug-05	157769
18	1	QC	OPTIMA Phase I, CSF ctrl 4, replicate 1	CSF ctrl 4	1	4	4-Aug-05	157770
19	1	Study Sample	OPTIMA Phase I, OPP 16, replicate 1	OPP 16	1	6	4-Aug-05	157771
20	1	Study Sample	OPTIMA Phase I, OPP 17, replicate 1	OPP 17	1	1	4-Aug-05	157772
21	1	Study Sample	OPTIMA Phase I, OPP 18, replicate 1	OPP 18	1	3	4-Aug-05	157773
22	1	Study Sample	OPTIMA Phase I, OPP 19, replicate 1	OPP 19	1	4	4-Aug-05	157774
23	1	Study Sample	OPTIMA Phase I, OPP 20, replicate 1	OPP 20	1	6	5-Aug-05	157775
24	1	QC	OPTIMA Phase I, CSF ctrl 5, replicate 1	CSF ctrl 5	1	5	5-Aug-05	157776
25	1	QC	OPTIMA Phase I, Con 03	Con 03			5-Aug-05	157777
26	1	QC	OPTIMA Phase I, Con 04	Con 04			5-Aug-05	157778
27	1	QC	OPTIMA Phase I, CSF ctrl 5, replicate 2	CSF ctrl 5	1	5	5-Aug-05	157779
28	1	Study Sample	OPTIMA Phase I, OPP 20, replicate 2	OPP 20	1	6	5-Aug-05	157780
29	1	Study Sample	OPTIMA Phase I, OPP 19, replicate 2	OPP 19	1	4	5-Aug-05	157781
30	1	Study Sample	OPTIMA Phase I, OPP 18, replicate 2	OPP 18	1	3	5-Aug-05	157782
31	1	Study Sample	OPTIMA Phase I, OPP 17, replicate 2	OPP 17	1	1	5-Aug-05	157783
32	1	Study Sample	OPTIMA Phase I, OPP 16, replicate 2	OPP 16	1	6	5-Aug-05	157784
33	1	QC	OPTIMA Phase I, CSF ctrl 4, replicate 2	CSF ctrl 4	1	4	5-Aug-05	157785
34	1	Study Sample	OPTIMA Phase I, OPP 15, replicate 2	OPP 15	1	5	5-Aug-05	157786
35	1	Study Sample	OPTIMA Phase I, OPP 14, replicate 2	OPP 14	1	3	5-Aug-05	157787
36	1	Study Sample	OPTIMA Phase I, OPP 13, replicate 2	OPP 13	1	2	5-Aug-05	157788
37	1	Study Sample	OPTIMA Phase I, OPP 12, replicate 2	OPP 12	1	6	5-Aug-05	157789
38	1	Study Sample	OPTIMA Phase I, OPP 11, replicate 2	OPP 11	1	4	5-Aug-05	157790
39	1	QC	OPTIMA Phase I, CSF ctrl 3, replicate 2	CSF ctrl 3	1	3	5-Aug-05	157791
40	1	Study Sample	OPTIMA Phase I, OPP 10, replicate 2	OPP 10	1	2	5-Aug-05	157792
41	1	Study Sample	OPTIMA Phase I, OPP 9, replicate 2	OPP 9	1	1	6-Aug-05	157793
42	1	Study Sample	OPTIMA Phase I, OPP 8, replicate 2	OPP 8	1	6	6-Aug-05	157794
43	1	Study Sample	OPTIMA Phase I, OPP 7, replicate 2	OPP 7	1	5	6-Aug-05	157795
44	1	Study Sample	OPTIMA Phase I, OPP 6, replicate 2	OPP 6	1	3	6-Aug-05	157796
45	1	QC	OPTIMA Phase I, CSF ctrl 2, replicate 2	CSF ctrl 2	1	2	6-Aug-05	157797
46	1	Study Sample	OPTIMA Phase I, OPP 5, replicate 2	OPP 5	1	1	6-Aug-05	157798
47	1	Study Sample	OPTIMA Phase I, OPP 4, replicate 2	OPP 4	1	6	6-Aug-05	157799
48	1	Study Sample	OPTIMA Phase I, OPP 3, replicate 2	OPP 3	1	5	6-Aug-05	157800
49	1	Study Sample	OPTIMA Phase I, OPP 2, replicate 2	OPP 2	1	4	6-Aug-05	157801
50	1	Study Sample	OPTIMA Phase I, OPP 1, replicate 2	OPP 1	1	2	6-Aug-05	157802
