# Supplementary material for: High Resolution Discovery Proteomics Reveals Candidate Disease Progression Markers of Alzheimer’s Disease in Human Cerebrospinal Fluid
Source: PLoS One. 2015 Aug 13;10(8):e0135365. doi: 10.1371/journal.pone.0135365 (PMC4535975; doi:10.1371/journal.pone.0135365)
Supplement: S2 Table — (PDF) [file pone.0135365.s005.pdf]

S2 Table Cohort-1 Random Forest Classifier, top 100 features

Rank	Classifier V1	Classifier V2	Feature ID	Peak Centroid m/z	Peak Centroid Time
1	722477306	1.737140405	722477306	763.404	27.834
2	722483894	1.685407481	722483894	555.334	23.504
3	722481114	1.614330103	722481114	749.347	18.527
4	722475290	1.56897457	722475290	775.417	34.302
5	722483245	1.568358924	722483245	914.103	25.805
6	722476350	1.462160702	722476350	635.642	22.129
7	722477570	1.431720625	722477570	589.247	16.13
8	722480377	1.404779334	722480377	505.244	17.14
9	722488454	1.370446987	722488454	933.634	21.315
10	722477539	1.364195635	722477539	975.527	47.352
11	722485183	1.290189225	722485183	1211.864	42.152
12	722484870	1.273569858	722484870	554.805	20.093
13	722487836	1.23115334	722487836	937.949	27.007
14	722485065	1.198431671	722485065	944.511	21.202
15	722482820	1.17358046	722482820	549.959	33.26
16	722482696	1.169239411	722482696	627.3	26.069
17	722488165	1.168854823	722488165	682.654	20.044
18	722487538	1.159623964	722487538	984.455	20.503
19	722475904	1.144900323	722475904	820.409	24.217
20	722477243	1.12748288	722477243	826.493	30.159
21	722485363	1.127418344	722485363	556.803	17.891
22	722482950	1.124450167	722482950	1016.246	30.781
23	722486648	1.121955569	722486648	391.24	25.795
24	722482237	1.1087247	722482237	510.264	15.439
25	722474502	1.099388496	722474502	519.271	24.718
26	722486133	1.089425886	722486133	726.87	19.443
27	722481832	1.083474287	722481832	1084.037	36.832
28	722471497	1.069702186	722471497	954.503	43.779
29	722483498	1.052381206	722483498	800.116	19.232
30	722483801	1.050557779	722483801	838.926	26.531
31	722487689	1.050527195	722487689	576.292	16.892
32	722470272	1.041769761	722470272	550.491	18.741
33	722476274	1.041532581	722476274	985.668	27.815
34	722486399	1.021523187	722486399	1003.445	22.37
35	722477676	1.002027956	722477676	1022.31	43.991
36	722479267	0.994177942	722479267	478.62	29.156
37	722481320	0.991911446	722481320	559.591	17.806
38	722486597	0.98692438	722486597	614.619	27.074
39	722470808	0.982372159	722470808	762.409	48.26
40	722485140	0.958018899	722485140	1040.724	33.626
41	722483210	0.933606955	722483210	1432.676	30.663
42	722479870	0.92778123	722479870	1013.767	25.657
43	722487044	0.924302884	722487044	639.959	27.468
44	722482185	0.910578364	722482185	985.511	45.85
45	722483101	0.902253449	722483101	631.285	15.372
46	722487401	0.893727489	722487401	414.236	16.713
47	722485234	0.884755967	722485234	1149.785	30.852
48	722474226	0.881512438	722474226	639.63	24.831
49	722481244	0.87644276	722481244	784.871	22.507
50	722477637	0.875648389	722477637	472.273	16.353
51	722478318	0.873144693	722478318	820.417	15.951
52	722476160	0.855355589	722476160	1028.004	41.331

53	722483003	0.852322777	722483003	587.342	16.503
54	722480746	0.848141015	722480746	1022.183	36.882
55	722471162	0.846812045	722471162	847.432	46.689
56	722473866	0.837794462	722473866	1046.436	26.803
57	722476837	0.836340602	722476837	887.083	30.7
58	722473433	0.83606331	722473433	642.309	21.035
59	722470619	0.835683258	722470619	802.396	32.558
60	722483121	0.832936916	722483121	889.648	18.876
61	722475128	0.832063611	722475128	1059.146	17.377
62	722487621	0.829593428	722487621	1152.532	24.143
63	722484517	0.811272854	722484517	915.732	36.106
64	722488254	0.811152246	722488254	767.378	17.546
65	722486047	0.80191255	722486047	801.455	24.583
66	722480121	0.796696356	722480121	1117.76	34.092
67	722474419	0.795635484	722474419	574.775	21.005
68	722488431	0.794443588	722488431	374.882	16.522
69	722488055	0.793519799	722488055	564.605	22.309
70	722474856	0.792654904	722474856	561.93	16.252
71	722486752	0.790123577	722486752	601.184	15.407
72	722487016	0.786917526	722487016	626.307	40.055
73	722484707	0.784594179	722484707	804.862	21.217
74	722485259	0.783958268	722485259	1016.503	20.514
75	722476927	0.779364667	722476927	745.317	27.161
76	722484754	0.777986308	722484754	607.314	33.526
77	722481437	0.777641091	722481437	685.347	30.1
78	722472906	0.777514332	722472906	539.777	19.197
79	722476808	0.77668148	722476808	676.528	19.161
80	722472241	0.774992338	722472241	911.982	49.248
81	722480462	0.771631527	722480462	937.983	33.968
82	722476442	0.768634753	722476442	922.68	32.881
83	722476320	0.76499288	722476320	745.663	27.175
84	722486239	0.763873967	722486239	600.717	19.757
85	722488048	0.756036905	722488048	916.44	29.839
86	722483233	0.752879265	722483233	1149.219	35.704
87	722483816	0.752879265	722483816	614.925	20.136
88	722487305	0.748559987	722487305	775.699	31.006
89	722476431	0.743263115	722476431	451.894	14.669
90	722469628	0.739292412	722469628	778.38	22.619
91	722486573	0.726208149	722486573	754.324	31.045
92	722471029	0.713900263	722471029	837.851	16.68
93	722475385	0.712497416	722475385	1095.469	23.629
94	722480068	0.706687665	722480068	835.422	25.233
95	722482464	0.706687665	722482464	1019.783	26.312
96	722488050	0.701275626	722488050	560.792	36.739
97	722487592	0.697301393	722487592	789.859	16.503
98	722486344	0.697016653	722486344	772.857	16.578
99	722488143	0.69179712	722488143	823.965	20.323
100	722486945	0.691105786	722486945	733.525	39.813
