# Supplementary material for: High Resolution Discovery Proteomics Reveals Candidate Disease Progression Markers of Alzheimer’s Disease in Human Cerebrospinal Fluid
Source: PLoS One. 2015 Aug 13;10(8):e0135365. doi: 10.1371/journal.pone.0135365 (PMC4535975; doi:10.1371/journal.pone.0135365)
Supplement: S3 Table — (PDF) [file pone.0135365.s006.pdf]

Interwoven block design for logitudinal study (Cohort-2)

LC-MS Inj	LC-MS Block		Description	Blinded Sample Name	BiochemBlocl	IMD Column	File Name	Dx
1	1	QC	CSF Equil MSBlock_1 #_1	CSF Equil			320165	
2	1	QC	CSF Equil MSBlock_1 #_10	CSF Equil			320166	
3	1	QC	CSF Equil MSBlock_1 #_11	CSF Equil			320167	
4	1	QC	CSF Equil MSBlock_1 #_12	CSF Equil			320168	
5	1	QC	BAN MSBlock_1 #_13	BAN			320169	
6	1	QC	BAN MSBlock_1 #_14	BAN			320170	
7	1	QC	BAN MSBlock_1 #_15	BAN			320171	
8	1	QC	Conalbumin MSBlock_1 #_16	Conalbumin			320172	
9	1	Study Sample	OPTIMA =229, EPNo =11, Years =5.04	M31133	2	4	320173	pMCI
10	1	Study Sample	OPTIMA =16, EPNo =5, Years =2.01	M30996	1	6	320174	AD
11	1	Study Sample	OPTIMA =229, EPNo =13, Years =5.94	M31134	2	4	320175	pMCI
12	1	Study Sample	OPTIMA =16, EPNo =7, Years =2.98	M30997	1	6	320176	AD
13	1	Study Sample	OPTIMA =229, EPNo =3, Years =1.07	M31129	2	4	320177	pMCI
14	1	Study Sample	OPTIMA =16, EPNo =15, Years =6.96	M31001	1	6	320178	AD
15	1	Study Sample	OPTIMA =229, EPNo =5, Years =2.03	M31130	2	4	320179	pMCI
16	1	Study Sample	OPTIMA =16, EPNo =11, Years =5	M30999	1	6	320180	AD
17	1	Study Sample	OPTIMA =229, EPNo =1, Years =0	M31128	2	4	320181	pMCI
18	1	Study Sample	OPTIMA =16, EPNo =13, Years =5.97	M31000	1	6	320182	AD
19	1	QC	BAN MSBlock_1 #_19	BAN			320183	
20	1	QC	Conalbumin MSBlock_1 #_20	Conalbumin			320184	
21	1	Study Sample	OPTIMA =229, EPNo =7, Years =3.02	M31131	2	4	320185	pMCI
22	1	Study Sample	OPTIMA =16, EPNo =3, Years =0.99	M30995	1	6	320186	AD
23	1	Study Sample	OPTIMA =229, EPNo =9, Years =4.06	M31132	2	4	320187	pMCI
24	1	Study Sample	OPTIMA =16, EPNo =9, Years =3.98	M30998	1	6	320188	AD
25	1	Study Sample	OPTIMA =150, EPNo =3, Years =1.5	M31077	1	2	320189	AD
26	1	Study Sample	OPTIMA =74, EPNo =5, Years =2.09	M31032	4	5	320190	sCTL
27	1	Study Sample	OPTIMA =150, EPNo =7, Years =3.32	M31079	1	2	320191	AD
28	1	Study Sample	OPTIMA =74, EPNo =3, Years =1	M31031	4	5	320192	sCTL
29	1	Study Sample	OPTIMA =150, EPNo =5, Years =2.37	M31078	1	2	320193	AD
30	1	Study Sample	OPTIMA =74, EPNo =1, Years =0	M31030	4	5	320194	sCTL
31	1	QC	BAN MSBlock_1 #_31	BAN			320195	
32	1	QC	Conalbumin MSBlock_1 #_32	Conalbumin			320196	
33	1	Study Sample	OPTIMA =150, EPNo =1, Years =0	M31076	1	2	320197	AD
34	1	Study Sample	OPTIMA =102, EPNo =5, Years =1.94	M31046	1	1	320198	AD
35	1	Study Sample	OPTIMA =159, EPNo =5, Years =2.05	M31088	4	3	320199	AD
36	1	Study Sample	OPTIMA =102, EPNo =1, Years =0	M31045	1	1	320200	AD
37	1	Study Sample	OPTIMA =159, EPNo =3, Years =1	M31087	4	3	320201	AD
38	1	Study Sample	OPTIMA =102, EPNo =7, Years =2.93	M31047	1	1	320202	AD
39	1	Study Sample	OPTIMA =159, EPNo =1, Years =0	M31086	4	3	320203	AD
40	1	Study Sample	OPTIMA =57, EPNo =3, Years =1.08	M31018	1	4	320204	sCTL
41	1	Study Sample	OPTIMA =159, EPNo =7, Years =2.99	M31089	4	3	320205	AD
42	1	Study Sample	OPTIMA =57, EPNo =1, Years =0	M31017	1	4	320206	sCTL
43	1	QC	BAN MSBlock_1 #_43	BAN			320207	
44	1	QC	Conalbumin MSBlock_1 #_44	Conalbumin			320208	
45	1	Study Sample	OPTIMA =214, EPNo =3, Years =0.97	M31107	2	6	320209	pCTL.MCI
46	1	Study Sample	OPTIMA =66, EPNo =5, Years =2.12	M31029	1	4	320210	sCTL

47	1 Study Sample	OPTIMA =214, EPNo =1, Years =0	M31106	2	6	320211	pCTL.MCI
48	1 Study Sample	OPTIMA =66, EPNo =1, Years =0	M31028	1	4	320212	sCTL
49	1 Study Sample	OPTIMA =214, EPNo =5, Years =2.1	M31108	2	6	320213	pCTL.MCI
50	1 Study Sample	OPTIMA =137, EPNo =3, Years =0.93	M31069	1	2	320214	AD
51	1 Study Sample	OPTIMA =84, EPNo =1, Years =0	M31037	2	5	320215	sCTL
52	1 Study Sample	OPTIMA =137, EPNo =1, Years =0	M31068	1	2	320216	AD
53	1 Study Sample	OPTIMA =222, EPNo =5, Years =1.97	M31122	2	6	320217	pMCI
54	1 Study Sample	OPTIMA =84, EPNo =3, Years =1.06	M31038	2	5	320218	sCTL
55	1 QC	BAN MSBlock_1 #_55	BAN			320219	
56	1 QC	Conalbumin MSBlock_1 #_56	Conalbumin			320220	
57	1 Study Sample	OPTIMA =222, EPNo =1, Years =0	M31120	2	6	320221	pMCI
58	1 Study Sample	OPTIMA =91, EPNo =3, Years =1.12	M31039	2	6	320222	sCTL
59	1 Study Sample	OPTIMA =222, EPNo =3, Years =0.96	M31121	2	6	320223	pMCI
60	1 Study Sample	OPTIMA =300, EPNo =1, Years =0	M31163	4	6	320224	sCTL
61	1 Study Sample	OPTIMA =91, EPNo =5, Years =2.16	M31040	2	6	320225	sCTL
62	1 Study Sample	OPTIMA =300, EPNo =3, Years =1.03	M31164	4	6	320226	sCTL
63	1 QC	BAN MSBlock_1 #_63	BAN			320227	
64	1 QC	Conalbumin MSBlock_1 #_64	Conalbumin			320228	
65	2 QC	CSF Equil MSBlock_2 #_1	CSF Equil			320229	
66	2 QC	CSF Equil MSBlock_2 #_2	CSF Equil			320230	
67	2 QC	CSF Equil MSBlock_2 #_3	CSF Equil			320231	
68	2 QC	CSF Equil MSBlock_2 #_4	CSF Equil			320232	
69	2 QC	BAN MSBlock_2 #_5	BAN			320233	
70	2 QC	BAN MSBlock_2 #_6	BAN			320234	
71	2 QC	BAN MSBlock_2 #_7	BAN			320235	
72	2 QC	Conalbumin MSBlock_2 #_8	Conalbumin			320236	
73	2 Study Sample	OPTIMA =105, EPNo =1, Years =0	M31048	4	1	320237	AD
74	2 Study Sample	OPTIMA =105, EPNo =13, Years =6.12	M31052	4	1	320238	AD
75	2 Study Sample	OPTIMA =59, EPNo =7, Years =3.24	M31022	3	2	320239	sCTL
76	2 Study Sample	OPTIMA =105, EPNo =3, Years =1.16	M31049	4	1	320240	AD
77	2 Study Sample	OPTIMA =59, EPNo =5, Years =2.2	M31021	3	2	320241	sCTL
78	2 Study Sample	OPTIMA =105, EPNo =7, Years =3.12	M31051	4	1	320242	AD
79	2 Study Sample	OPTIMA =59, EPNo =1, Years =0	M31019	3	2	320243	sCTL
80	2 Study Sample	OPTIMA =105, EPNo =5, Years =2.13	M31050	4	1	320244	AD
81	2 Study Sample	OPTIMA =59, EPNo =9, Years =4.19	M31023	3	2	320245	sCTL
82	2 Study Sample	OPTIMA =59, EPNo =11, Years =5.19	M31024	3	2	320246	sCTL
83	2 QC	BAN MSBlock_2 #_19	BAN			320247	
84	2 QC	Conalbumin MSBlock_2 #_20	Conalbumin			320248	
85	2 Study Sample	OPTIMA =82, EPNo =3, Years =1.03	M31033	2	3	320249	sCTL
86	2 Study Sample	OPTIMA =59, EPNo =3, Years =1.1	M31020	3	2	320250	sCTL
87	2 Study Sample	OPTIMA =82, EPNo =7, Years =3.07	M31035	2	3	320251	sCTL
88	2 Study Sample	OPTIMA =252, EPNo =1, Years =0	M31145	4	3	320252	sCTL
89	2 Study Sample	OPTIMA =82, EPNo =5, Years =1.96	M31034	2	3	320253	sCTL
90	2 Study Sample	OPTIMA =252, EPNo =5, Years =2.19	M31147	4	3	320254	sCTL
91	2 Study Sample	OPTIMA =82, EPNo =9, Years =4.01	M31036	2	3	320255	sCTL
92	2 Study Sample	OPTIMA =252, EPNo =3, Years =1.1	M31146	4	3	320256	sCTL
93	2 Study Sample	OPTIMA =207, EPNo =5, Years =2.09	M31102	3	3	320257	pCTL.AD

94	2	Study Sample	OPTIMA =207, EPNo =1, Years =0	M31100	3	3	320258	pCTL.AD
95	2	QC	BAN MSBlock_2 #_31	BAN			320259	
96	2	QC	Conalbumin MSBlock_2 #_32	Conalbumin			320260	
97	2	Study Sample	OPTIMA =147, EPNo =3, Years =1.09	M31074	1	3	320261	AD
98	2	Study Sample	OPTIMA =207, EPNo =7, Years =3.1	M31103	3	3	320262	pCTL.AD
99	2	Study Sample	OPTIMA =147, EPNo =1, Years =0	M31073	1	3	320263	AD
100	2	Study Sample	OPTIMA =207, EPNo =3, Years =1.07	M31101	3	3	320264	pCTL.AD
101	2	Study Sample	OPTIMA =147, EPNo =5, Years =1.97	M31075	1	3	320265	AD
102	2	Study Sample	OPTIMA =145, EPNo =3, Years =1.14	M31071	1	5	320266	AD
103	2	Study Sample	OPTIMA =37, EPNo =1, Years =0	M31006	1	2	320267	sCTL
104	2	Study Sample	OPTIMA =145, EPNo =7, Years =3.09	M31072	1	5	320268	AD
105	2	Study Sample	OPTIMA =37, EPNo =3, Years =1.15	M31007	1	2	320269	sCTL
106	2	Study Sample	OPTIMA =145, EPNo =1, Years =0	M31070	1	5	320270	AD
107	2	QC	BAN MSBlock_2 #_43	BAN			320271	
108	2	QC	Conalbumin MSBlock_2 #_44	Conalbumin			320272	
109	2	Study Sample	OPTIMA =95, EPNo =3, Years =1.01	M31042	3	3	320273	sCTL
110	2	Study Sample	OPTIMA =226, EPNo =1, Years =0	M31125	2	2	320274	pMCI
111	2	Study Sample	OPTIMA =95, EPNo =1, Years =0	M31041	3	3	320275	sCTL
112	2	Study Sample	OPTIMA =226, EPNo =3, Years =1.06	M31126	2	2	320276	pMCI
113	2	Study Sample	OPTIMA =100, EPNo =1, Years =0	M31043	3	4	320277	AD
114	2	Study Sample	OPTIMA =226, EPNo =7, Years =3.06	M31127	2	2	320278	pMCI
115	2	Study Sample	OPTIMA =293, EPNo =1, Years =0	M31161	4	5	320279	sCTL
116	2	Study Sample	OPTIMA =100, EPNo =3, Years =1.17	M31044	3	4	320280	AD
117	2	Study Sample	OPTIMA =293, EPNo =3, Years =1.01	M31162	4	5	320281	sCTL
118	2	Study Sample	OPTIMA =180, EPNo =1, Years =0	M31096	3	4	320282	ODS
119	2	QC	BAN MSBlock_2 #_55	BAN			320283	
120	2	QC	Conalbumin MSBlock_2 #_56	Conalbumin			320284	
121	2	Study Sample	OPTIMA =215, EPNo =1, Years =0	M31109	2	2	320285	pCTL.MCI
122	2	Study Sample	OPTIMA =180, EPNo =3, Years =1.29	M31097	3	4	320286	ODS
123	2	Study Sample	OPTIMA =245, EPNo =1, Years =0	M31140	4	2	320287	sCTL
124	2	QC	BAN MSBlock_2 #_60	BAN			320288	
125	2	QC	Conalbumin MSBlock_2 #_61	Conalbumin			320289	
126	3	QC	CSF Equil MSBlock_3 #_1	CSF Equil			320290	
127	3	QC	CSF Equil MSBlock_3 #_2	CSF Equil			320291	
128	3	QC	CSF Equil MSBlock_3 #_3	CSF Equil			320292	
129	3	QC	CSF Equil MSBlock_3 #_4	CSF Equil			320293	
130	3	QC	BAN MSBlock_3 #_5	BAN			320294	
131	3	QC	BAN MSBlock_3 #_6	BAN			320295	
132	3	QC	BAN MSBlock_3 #_7	BAN			320296	
133	3	QC	Conalbumin MSBlock_3 #_8	Conalbumin			320297	
134	3	Study Sample	OPTIMA =127, EPNo =9, Years =3.98	M31066	1	1	320298	AD
135	3	Study Sample	OPTIMA =33, EPNo =5, Years =2.98	M31003	2	1	320299	sCTL
136	3	Study Sample	OPTIMA =127, EPNo =1, Years =0	M31063	1	1	320300	AD
137	3	QC	CSF Equil MSBlock_3 #_1	CSF Equil			320301	
138	3	QC	CSF Equil MSBlock_3 #_2	CSF Equil			320302	
139	3	QC	CSF Equil MSBlock_3 #_3	CSF Equil			320303	
140	3	QC	CSF Equil MSBlock_3 #_4	CSF Equil			320304	

141	3 QC	BAN MSBlock_3 #_5	BAN			320305	
142	3 QC	BAN MSBlock_3 #_6	BAN			320306	
143	3 QC	BAN MSBlock_3 #_7	BAN			320307	
144	3 QC	Conalbumin MSBlock_3 #_8	Conalbumin			320308	
145	3 Study Sample	OPTIMA =127, EPNo =9, Years =3.98	M31066	1	1	320309	AD
146	3 Study Sample	OPTIMA =33, EPNo =5, Years =2.98	M31003	2	1	320310	sCTL
147	3 Study Sample	OPTIMA =127, EPNo =1, Years =0	M31063	1	1	320311	AD
148	3 Study Sample	OPTIMA =33, EPNo =7, Years =3.98	M31004	2	1	320312	sCTL
149	3 Study Sample	OPTIMA =127, EPNo =7, Years =3.02	M31065	1	1	320313	AD
150	3 Study Sample	OPTIMA =33, EPNo =3, Years =1.97	M31002	2	1	320314	sCTL
151	3 Study Sample	OPTIMA =127, EPNo =3, Years =1.01	M31064	1	1	320315	AD
152	3 Study Sample	OPTIMA =33, EPNo =9, Years =4.96	M31005	2	1	320316	sCTL
153	3 Study Sample	OPTIMA =127, EPNo =11, Years =4.92	M31067	1	1	320317	AD
154	3 Study Sample	OPTIMA =331, EPNo =3, Years =1.08	M31168	2	5	320318	sCTL
155	3 QC	BAN MSBlock_3 #_19	BAN			320319	
156	3 QC	Conalbumin MSBlock_3 #_20	Conalbumin			320320	
157	3 Study Sample	OPTIMA =54, EPNo =1, Years =0	M31013	3	4	320321	sCTL
158	3 Study Sample	OPTIMA =54, EPNo =5, Years =2.14	M31014	3	4	320322	sCTL
159	3 Study Sample	OPTIMA =331, EPNo =1, Years =0	M31167	2	5	320323	sCTL
160	3 Study Sample	OPTIMA =54, EPNo =9, Years =3.98	M31015	3	4	320324	sCTL
161	3 Study Sample	OPTIMA =54, EPNo =11, Years =5.21	M31016	3	4	320325	sCTL
162	3 Study Sample	OPTIMA =156, EPNo =3, Years =1.12	M31081	3	1	320326	AD
163	3 Study Sample	OPTIMA =216, EPNo =3, Years =1.05	M31111	2	1	320327	pCTL.MCI
164	3 Study Sample	OPTIMA =156, EPNo =1, Years =0	M31080	3	1	320328	AD
165	3 Study Sample	OPTIMA =216, EPNo =5, Years =2.07	M31112	2	1	320329	pCTL.MCI
166	3 Study Sample	OPTIMA =156, EPNo =5, Years =2.12	M31082	3	1	320330	AD
167	3 QC	BAN MSBlock_3 #_31	BAN			320331	
168	3 QC	Conalbumin MSBlock_3 #_32	Conalbumin			320332	
169	3 Study Sample	OPTIMA =51, EPNo =3, Years =0.96	M31012	1	5	320333	sCTL
170	3 Study Sample	OPTIMA =216, EPNo =1, Years =0	M31110	2	1	320334	pCTL.MCI
171	3 Study Sample	OPTIMA =51, EPNo =1, Years =0	M31011	1	5	320335	sCTL
172	3 Study Sample	OPTIMA =63, EPNo =3, Years =0.99	M31026	1	5	320336	sCTL
173	3 Study Sample	OPTIMA =107, EPNo =3, Years =1.11	M31054	3	5	320337	AD
174	3 Study Sample	OPTIMA =63, EPNo =1, Years =0	M31025	1	5	320338	sCTL
175	3 Study Sample	OPTIMA =107, EPNo =1, Years =0	M31053	3	5	320339	AD
176	3 Study Sample	OPTIMA =63, EPNo =9, Years =3.99	M31027	1	5	320340	sCTL
177	3 Study Sample	OPTIMA =118, EPNo =1, Years =0	M31059	2	2	320341	AD
178	3 Study Sample	OPTIMA =241, EPNo =5, Years =2.01	M31139	3	6	320342	sCTL
179	3 QC	BAN MSBlock_3 #_43	BAN			320343	
180	3 QC	Conalbumin MSBlock_3 #_44	Conalbumin			320344	
181	3 Study Sample	OPTIMA =118, EPNo =3, Years =1.12	M31060	2	2	320345	AD
182	3 Study Sample	OPTIMA =241, EPNo =1, Years =0	M31137	3	6	320346	pMCI
183	3 Study Sample	OPTIMA =121, EPNo =3, Years =1.12	M31062	2	2	320347	AD
184	3 Study Sample	OPTIMA =241, EPNo =3, Years =1.02	M31138	3	6	320348	sCTL
185	3 Study Sample	OPTIMA =173, EPNo =3, Years =0.99	M31091	3	3	320349	AD
186	3 Study Sample	OPTIMA =121, EPNo =1, Years =0	M31061	2	2	320350	AD
187	3 Study Sample	OPTIMA =173, EPNo =1, Years =0	M31090	3	3	320351	AD

188	3	Study Sample	OPTIMA =281, EPNo =5, Years =2.06	M31159	4	6	320352	sCTL
189	3	Study Sample	OPTIMA =188, EPNo =1, Years =0	M31098	3	6	320353	ODS
190	3	QC	BAN MSBlock_3 #_54	BAN			320354	
191	3	QC	Conalbumin MSBlock_3 #_55	Conalbumin			320355	
192	3	Study Sample	OPTIMA =281, EPNo =7, Years =3.03	M31160	4	6	320356	sCTL
193	3	Study Sample	OPTIMA =225, EPNo =3, Years =1.01	M31124	4	2	320357	pMCI
194	3	Study Sample	OPTIMA =188, EPNo =3, Years =1.04	M31099	3	6	320358	pCTL.AD
195	3	Study Sample	OPTIMA =281, EPNo =1, Years =0	M31158	4	6	320359	sCTL
196	3	Study Sample	OPTIMA =225, EPNo =1, Years =0	M31123	4	2	320360	pMCI
197	3	Study Sample	OPTIMA =216, EPNo =5, Years =2.07	M31112	2	1	320361	pCTL.MCI
198	3	QC	BAN MSBlock_3 #_61	BAN			320362	
199	3	QC	Conalbumin MSBlock_3 #_62	Conalbumin			320363	
200	4	QC	CSF Equil MSBlock_4 #_1	CSF Equil			320364	
201	4	QC	CSF Equil MSBlock_4 #_2	CSF Equil			320365	
202	4	QC	CSF Equil MSBlock_4 #_3	CSF Equil			320366	
203	4	QC	CSF Equil MSBlock_4 #_4	CSF Equil			320367	
204	4	QC	BAN MSBlock_4 #_5	BAN			320368	
205	4	QC	BAN MSBlock_4 #_6	BAN			320369	
206	4	QC	BAN MSBlock_4 #_7	BAN			320370	
207	4	QC	Conalbumin MSBlock_4 #_8	Conalbumin			320371	
208	4	Study Sample	OPTIMA =219, EPNo =1, Years =0	M31113	3	1	320372	pCTL.MCI
209	4	Study Sample	OPTIMA =114, EPNo =7, Years =3.1	M31058	4	2	320373	AD
210	4	Study Sample	OPTIMA =219, EPNo =5, Years =1.91	M31115	3	1	320374	pMCI
211	4	Study Sample	OPTIMA =114, EPNo =3, Years =1.16	M31056	4	2	320375	AD
212	4	Study Sample	OPTIMA =114, EPNo =1, Years =0	M31055	4	2	320376	AD
213	4	Study Sample	OPTIMA =219, EPNo =3, Years =1.01	M31114	3	1	320377	pMCI
214	4	Study Sample	OPTIMA =114, EPNo =5, Years =2.14	M31057	4	2	320378	AD
215	4	Study Sample	OPTIMA =178, EPNo =7, Years =3.14	M31095	2	3	320379	ODS
216	4	Study Sample	OPTIMA =220, EPNo =5, Years =2.03	M31118	1	3	320380	pMCI
217	4	Study Sample	OPTIMA =178, EPNo =1, Years =0	M31092	2	3	320381	ODS
218	4	QC	BAN MSBlock_4 #_19	BAN			320382	
219	4	QC	Conalbumin MSBlock_4 #_20	Conalbumin			320383	
220	4	Study Sample	OPTIMA =220, EPNo =3, Years =1.07	M31117	1	3	320384	pMCI
221	4	Study Sample	OPTIMA =178, EPNo =5, Years =2.13	M31094	2	3	320385	ODS
222	4	Study Sample	OPTIMA =220, EPNo =1, Years =0	M31116	1	3	320386	pMCI
223	4	Study Sample	OPTIMA =178, EPNo =3, Years =1.11	M31093	2	3	320387	ODS
224	4	Study Sample	OPTIMA =220, EPNo =7, Years =2.97	M31119	1	3	320388	pMCI
225	4	Study Sample	OPTIMA =158, EPNo =3, Years =1.16	M31084	3	5	320389	AD
226	4	Study Sample	OPTIMA =158, EPNo =5, Years =2.14	M31085	3	5	320390	AD
227	4	Study Sample	OPTIMA =257, EPNo =9, Years =4	M31153	1	4	320391	sCTL
228	4	Study Sample	OPTIMA =158, EPNo =1, Years =0	M31083	3	5	320392	AD
229	4	Study Sample	OPTIMA =271, EPNo =3, Years =1.09	M31155	4	4	320393	sCTL
230	4	QC	BAN MSBlock_4 #_31	BAN			320394	
231	4	QC	Conalbumin MSBlock_4 #_32	Conalbumin			320395	
232	4	Study Sample	OPTIMA =257, EPNo =1, Years =0	M31151	1	4	320396	sCTL
233	4	Study Sample	OPTIMA =257, EPNo =3, Years =1.15	M31152	1	4	320397	sCTL
234	4	Study Sample	OPTIMA =271, EPNo =1, Years =0	M31154	4	4	320398	sCTL

235	4 Study Sample	OPTIMA =48, EPNo =7, Years =3.2	M31010	3	5	320399	sCTL
236	4 Study Sample	OPTIMA =212, EPNo =3, Years =0.94	M31105	4	1	320400	pCTL.MCI
237	4 Study Sample	OPTIMA =48, EPNo =1, Years =0	M31008	3	5	320401	sCTL
238	4 Study Sample	OPTIMA =212, EPNo =1, Years =0	M31104	4	1	320402	pCTL.MCI
239	4 Study Sample	OPTIMA =48, EPNo =3, Years =1.21	M31009	3	5	320403	sCTL
240	4 Study Sample	OPTIMA =279, EPNo =1, Years =0	M31156	4	4	320404	sCTL
241	4 Study Sample	OPTIMA =246, EPNo =5, Years =1.93	M31144	3	6	320405	sCTL
242	4 QC	BAN MSBlock_4 #_43	BAN			320406	
243	4 QC	Conalbumin MSBlock_4 #_44	Conalbumin			320407	
244	4 Study Sample	OPTIMA =279, EPNo =3, Years =0.93	M31157	4	4	320408	sCTL
245	4 Study Sample	OPTIMA =246, EPNo =1, Years =0	M31142	3	6	320409	sCTL
246	4 Study Sample	OPTIMA =301, EPNo =3, Years =0.98	M31166	4	5	320410	sCTL
247	4 Study Sample	OPTIMA =246, EPNo =3, Years =0.95	M31143	3	6	320411	sCTL
248	4 Study Sample	OPTIMA =301, EPNo =1, Years =0	M31165	4	5	320412	sCTL
249	4 Study Sample	OPTIMA =245, EPNo =3, Years =1.13	M31141	4	2	320413	sCTL
250	4 Study Sample	OPTIMA =255, EPNo =3, Years =1.2	M31149	4	4	320414	sCTL
251	4 Study Sample	OPTIMA =443, EPNo =1, Years =0	M31169	4	6	320415	sCTL
252	4 Study Sample	OPTIMA =255, EPNo =5, Years =2.2	M31150	4	4	320416	pMCI
253	4 Study Sample	OPTIMA =237, EPNo =3, Years =1.08	M31136	2	5	320417	pMCI
254	4 QC	BAN MSBlock_4 #_55	BAN			320418	
255	4 QC	Conalbumin MSBlock_4 #_56	Conalbumin			320419	
256	4 Study Sample	OPTIMA =443, EPNo =3, Years =1.04	M31170	4	6	320420	sCTL
257	4 Study Sample	OPTIMA =255, EPNo =1, Years =0	M31148	4	4	320421	sCTL
258	4 Study Sample	OPTIMA =237, EPNo =1, Years =0	M31135	2	5	320422	pMCI
259	4 Study Sample	OPTIMA =219, EPNo =3, Years =1.01	M31114	3	1	320423	pMCI
260	4 Study Sample	OPTIMA =212, EPNo =3, Years =0.94	M31105	4	1	320424	pCTL.MCI
261	4 Study Sample	OPTIMA =212, EPNo =1, Years =0	M31104	4	1	320425	pCTL.MCI
262	4 Study Sample	OPTIMA =245, EPNo =3, Years =1.13	M31141	4	2	320426	sCTL
263	4 QC	BAN MSBlock_4 #_60	BAN			320427	
264	4 QC	Conalbumin MSBlock_4 #_61	Conalbumin			320428	
265	5 QC	CSF Equil MSBlock_5 #_1	CSF Equil			320429	
266	5 QC	CSF Equil MSBlock_5 #_2	CSF Equil			320430	
267	5 QC	CSF Equil MSBlock_5 #_3	CSF Equil			320431	
268	5 QC	CSF Equil MSBlock_5 #_4	CSF Equil			320432	
269	5 QC	BAN MSBlock_5 #_5	BAN			320433	
270	5 QC	BAN MSBlock_5 #_6	BAN			320434	
271	5 QC	BAN MSBlock_5 #_7	BAN			320435	
272	5 QC	Conalbumin MSBlock_5 #_8	Conalbumin			320436	
273	5 Study Sample	OPTIMA =229, EPNo =11, Years =5.04	M31133	2	4	320437	pMCI
274	5 Study Sample	OPTIMA =16, EPNo =5, Years =2.01	M30996	1	6	320438	AD
275	5 Study Sample	OPTIMA =229, EPNo =13, Years =5.94	M31134	2	4	320439	pMCI
276	5 Study Sample	OPTIMA =16, EPNo =7, Years =2.98	M30997	1	6	320440	AD
277	5 Study Sample	OPTIMA =229, EPNo =3, Years =1.07	M31129	2	4	320441	pMCI
278	5 Study Sample	OPTIMA =16, EPNo =15, Years =6.96	M31001	1	6	320442	AD
279	5 Study Sample	OPTIMA =229, EPNo =5, Years =2.03	M31130	2	4	320443	pMCI
280	5 Study Sample	OPTIMA =16, EPNo =11, Years =5	M30999	1	6	320444	AD
281	5 Study Sample	OPTIMA =229, EPNo =1, Years =0	M31128	2	4	320445	pMCI

282	5 Study Sample	OPTIMA =16, EPNo =13, Years =5.97	M31000	1	6	320446	AD
283	5 QC	BAN MSBlock_5 #_19	BAN			320447	
284	5 QC	Conalbumin MSBlock_5 #_20	Conalbumin			320448	
285	5 Study Sample	OPTIMA =229, EPNo =7, Years =3.02	M31131	2	4	320449	pMCI
286	5 Study Sample	OPTIMA =16, EPNo =3, Years =0.99	M30995	1	6	320450	AD
287	5 Study Sample	OPTIMA =229, EPNo =9, Years =4.06	M31132	2	4	320451	pMCI
288	5 Study Sample	OPTIMA =16, EPNo =9, Years =3.98	M30998	1	6	320452	AD
289	5 QC	BAN MSBlock_5 #_25	BAN			320453	
290	5 QC	BAN MSBlock_5 #_26	BAN			320454	
291	5 QC	Conalbumin MSBlock_5 #_27	Conalbumin			320455	
292	5 Study Sample	OPTIMA =16, EPNo =9, Years =3.98	M30998	1	6	320456	AD
293	5 Study Sample	OPTIMA =150, EPNo =3, Years =1.5	M31077	1	2	320457	
294	5 QC	BAN MSBlock_5 #_30	BAN			320458_070228142339	
295	5 QC	BAN MSBlock_5 #_31	BAN			320459	
296	5 QC	Conalbumin MSBlock_5 #_32	Conalbumin			320460	
297	5 Study Sample	OPTIMA =150, EPNo =3, Years =1.5	M31077	1	2	320461	AD
298	5 Study Sample	OPTIMA =74, EPNo =5, Years =2.09	M31032	4	5	320462	sCTL
299	5 Study Sample	OPTIMA =150, EPNo =7, Years =3.32	M31079	1	2	320463	AD
300	5 Study Sample	OPTIMA =74, EPNo =3, Years =1	M31031	4	5	320464	sCTL
301	5 Study Sample	OPTIMA =150, EPNo =5, Years =2.37	M31078	1	2	320465	AD
302	5 Study Sample	OPTIMA =74, EPNo =1, Years =0	M31030	4	5	320466	sCTL
303	5 QC	BAN MSBlock_5 #_39	BAN			320467	
304	5 QC	Conalbumin MSBlock_5 #_40	Conalbumin			320468	
305	5 Study Sample	OPTIMA =150, EPNo =1, Years =0	M31076	1	2	320469	AD
306	5 Study Sample	OPTIMA =102, EPNo =5, Years =1.94	M31046	1	1	320470	AD
307	5 Study Sample	OPTIMA =159, EPNo =5, Years =2.05	M31088	4	3	320471	AD
308	5 Study Sample	OPTIMA =102, EPNo =1, Years =0	M31045	1	1	320472	AD
309	5 Study Sample	OPTIMA =159, EPNo =3, Years =1	M31087	4	3	320473	AD
310	5 Study Sample	OPTIMA =102, EPNo =7, Years =2.93	M31047	1	1	320474	AD
311	5 Study Sample	OPTIMA =159, EPNo =1, Years =0	M31086	4	3	320475	AD
312	5 Study Sample	OPTIMA =57, EPNo =3, Years =1.08	M31018	1	4	320476	sCTL
313	5 Study Sample	OPTIMA =159, EPNo =7, Years =2.99	M31089	4	3	320477	AD
314	5 Study Sample	OPTIMA =57, EPNo =1, Years =0	M31017	1	4	320478	sCTL
315	5 QC	BAN MSBlock_5 #_51	BAN			320479	
316	5 QC	Conalbumin MSBlock_5 #_52	Conalbumin			320480	
317	5 Study Sample	OPTIMA =214, EPNo =3, Years =0.97	M31107	2	6	320481	pCTL.MCI
318	5 Study Sample	OPTIMA =66, EPNo =5, Years =2.12	M31029	1	4	320482	sCTL
319	5 Study Sample	OPTIMA =214, EPNo =1, Years =0	M31106	2	6	320483	pCTL.MCI
320	5 Study Sample	OPTIMA =66, EPNo =1, Years =0	M31028	1	4	320484	sCTL
321	5 Study Sample	OPTIMA =214, EPNo =5, Years =2.1	M31108	2	6	320485	pCTL.MCI
322	5 Study Sample	OPTIMA =137, EPNo =3, Years =0.93	M31069	1	2	320486	AD
323	5 Study Sample	OPTIMA =84, EPNo =1, Years =0	M31037	2	5	320487	sCTL
324	5 Study Sample	OPTIMA =137, EPNo =1, Years =0	M31068	1	2	320488	AD
325	5 Study Sample	OPTIMA =222, EPNo =5, Years =1.97	M31122	2	6	320489	pMCI
326	5 Study Sample	OPTIMA =84, EPNo =3, Years =1.06	M31038	2	5	320490	sCTL
327	5 QC	BAN MSBlock_5 #_63	BAN			320491	
328	5 QC	Conalbumin MSBlock_5 #_64	Conalbumin			320492	

329	5	Study Sample	OPTIMA =222, EPNo =1, Years =0	M31120	2	6	320493	pMCI
330	5	Study Sample	OPTIMA =91, EPNo =3, Years =1.12	M31039	2	6	320494	sCTL
331	5	Study Sample	OPTIMA =222, EPNo =3, Years =0.96	M31121	2	6	320495	pMCI
332	5	Study Sample	OPTIMA =300, EPNo =1, Years =0	M31163	4	6	320496	sCTL
333	5	Study Sample	OPTIMA =91, EPNo =5, Years =2.16	M31040	2	6	320497	sCTL
334	5	Study Sample	OPTIMA =300, EPNo =3, Years =1.03	M31164	4	6	320498	sCTL
335	5	QC	BAN MSBlock_5 #_71	BAN			320499	
336	5	QC	Conalbumin MSBlock_5 #_72	Conalbumin			320500	
337	6	QC	CSF Equil MSBlock_6 #_1	CSF Equil			320501	
338	6	QC	CSF Equil MSBlock_6 #_2	CSF Equil			320502	
339	6	QC	CSF Equil MSBlock_6 #_3	CSF Equil			320503	
340	6	QC	CSF Equil MSBlock_6 #_4	CSF Equil			320504	
341	6	QC	BAN MSBlock_6 #_5	BAN			320505	
342	6	QC	BAN MSBlock_6 #_6	BAN			320506	
343	6	QC	BAN MSBlock_6 #_7	BAN			320507	
344	6	QC	Conalbumin MSBlock_6 #_8	Conalbumin			320508	
345	6	Study Sample	OPTIMA =105, EPNo =1, Years =0	M31048	4	1	320509	AD
346	6	Study Sample	OPTIMA =105, EPNo =13, Years =6.12	M31052	4	1	320510	AD
347	6	Study Sample	OPTIMA =59, EPNo =7, Years =3.24	M31022	3	2	320511	sCTL
348	6	Study Sample	OPTIMA =105, EPNo =3, Years =1.16	M31049	4	1	320512	AD
349	6	Study Sample	OPTIMA =59, EPNo =5, Years =2.2	M31021	3	2	320513	sCTL
350	6	Study Sample	OPTIMA =105, EPNo =7, Years =3.12	M31051	4	1	320514	AD
351	6	Study Sample	OPTIMA =59, EPNo =1, Years =0	M31019	3	2	320515	sCTL
352	6	Study Sample	OPTIMA =105, EPNo =5, Years =2.13	M31050	4	1	320516	AD
353	6	Study Sample	OPTIMA =59, EPNo =9, Years =4.19	M31023	3	2	320517	sCTL
354	6	Study Sample	OPTIMA =59, EPNo =11, Years =5.19	M31024	3	2	320518	sCTL
355	6	QC	BAN MSBlock_6 #_19	BAN			320519	
356	6	QC	Conalbumin MSBlock_6 #_20	Conalbumin			320520	
357	6	Study Sample	OPTIMA =82, EPNo =3, Years =1.03	M31033	2	3	320521	sCTL
358	6	Study Sample	OPTIMA =59, EPNo =3, Years =1.1	M31020	3	2	320522	sCTL
359	6	Study Sample	OPTIMA =82, EPNo =7, Years =3.07	M31035	2	3	320523	sCTL
360	6	Study Sample	OPTIMA =252, EPNo =1, Years =0	M31145	4	3	320524	sCTL
361	6	Study Sample	OPTIMA =82, EPNo =5, Years =1.96	M31034	2	3	320525	sCTL
362	6	Study Sample	OPTIMA =252, EPNo =5, Years =2.19	M31147	4	3	320526	sCTL
363	6	Study Sample	OPTIMA =82, EPNo =9, Years =4.01	M31036	2	3	320527	sCTL
364	6	Study Sample	OPTIMA =252, EPNo =3, Years =1.1	M31146	4	3	320528	sCTL
365	6	Study Sample	OPTIMA =207, EPNo =5, Years =2.09	M31102	3	3	320529	pCTL.AD
366	6	Study Sample	OPTIMA =207, EPNo =1, Years =0	M31100	3	3	320530	pCTL.AD
367	6	QC	BAN MSBlock_6 #_31	BAN			320531	
368	6	QC	Conalbumin MSBlock_6 #_32	Conalbumin			320532	
369	6	Study Sample	OPTIMA =147, EPNo =3, Years =1.09	M31074	1	3	320533	AD
370	6	Study Sample	OPTIMA =207, EPNo =7, Years =3.1	M31103	3	3	320534	pCTL.AD
371	6	Study Sample	OPTIMA =147, EPNo =1, Years =0	M31073	1	3	320535	AD
372	6	Study Sample	OPTIMA =207, EPNo =3, Years =1.07	M31101	3	3	320536	pCTL.AD
373	6	Study Sample	OPTIMA =147, EPNo =5, Years =1.97	M31075	1	3	320537	AD
374	6	Study Sample	OPTIMA =145, EPNo =3, Years =1.14	M31071	1	5	320538	AD
375	6	Study Sample	OPTIMA =37, EPNo =1, Years =0	M31006	1	2	320539	sCTL

376	6	Study Sample	OPTIMA =145, EPNo =7, Years =3.09	M31072	1	5	320540	AD
377	6	Study Sample	OPTIMA =37, EPNo =3, Years =1.15	M31007	1	2	320541	sCTL
378	6	Study Sample	OPTIMA =145, EPNo =1, Years =0	M31070	1	5	320542	AD
379	6	QC	BAN MSBlock_6 #_43	BAN			320543	
380	6	QC	Conalbumin MSBlock_6 #_44	Conalbumin			320544	
381	6	Study Sample	OPTIMA =95, EPNo =3, Years =1.01	M31042	3	3	320545	sCTL
382	6	Study Sample	OPTIMA =226, EPNo =1, Years =0	M31125	2	2	320546	pMCI
383	6	Study Sample	OPTIMA =95, EPNo =1, Years =0	M31041	3	3	320547	sCTL
384	6	Study Sample	OPTIMA =226, EPNo =3, Years =1.06	M31126	2	2	320548	pMCI
385	6	Study Sample	OPTIMA =100, EPNo =1, Years =0	M31043	3	4	320549	AD
386	6	Study Sample	OPTIMA =226, EPNo =7, Years =3.06	M31127	2	2	320550	pMCI
387	6	Study Sample	OPTIMA =293, EPNo =1, Years =0	M31161	4	5	320551	sCTL
388	6	Study Sample	OPTIMA =100, EPNo =3, Years =1.17	M31044	3	4	320552	AD
389	6	Study Sample	OPTIMA =293, EPNo =3, Years =1.01	M31162	4	5	320553	sCTL
390	6	Study Sample	OPTIMA =180, EPNo =1, Years =0	M31096	3	4	320554	ODS
391	6	QC	BAN MSBlock_6 #_55	BAN			320555	
392	6	QC	Conalbumin MSBlock_6 #_56	Conalbumin			320556	
393	6	Study Sample	OPTIMA =215, EPNo =1, Years =0	M31109	2	2	320557	pCTL.MCI
394	6	Study Sample	OPTIMA =180, EPNo =3, Years =1.29	M31097	3	4	320558	ODS
395	6	Study Sample	OPTIMA =245, EPNo =1, Years =0	M31140	4	2	320559	sCTL
396	6	Study Sample	OPTIMA =226, EPNo =3, Years =1.06	M31126	2	2	320560	pMCI
397	6	QC	BAN MSBlock_6 #_60	BAN			320561	
398	6	QC	Conalbumin MSBlock_6 #_61	Conalbumin			320562	
399	7	QC	CSF Equil MSBlock_7 #_1	CSF Equil			320563	
400	7	QC	CSF Equil MSBlock_7 #_2	CSF Equil			320564	
401	7	QC	CSF Equil MSBlock_7 #_3	CSF Equil			320565	
402	7	QC	CSF Equil MSBlock_7 #_4	CSF Equil			320566	
403	7	QC	BAN MSBlock_7 #_5	BAN			320567	
404	7	QC	BAN MSBlock_7 #_6	BAN			320568	
405	7	QC	BAN MSBlock_7 #_7	BAN			320569	
406	7	QC	Conalbumin MSBlock_7 #_8	Conalbumin			320570	
407	7	Study Sample	OPTIMA =127, EPNo =9, Years =3.98	M31066	1	1	320571	AD
408	7	Study Sample	OPTIMA =33, EPNo =5, Years =2.98	M31003	2	1	320572	sCTL
409	7	Study Sample	OPTIMA =127, EPNo =1, Years =0	M31063	1	1	320573	AD
410	7	Study Sample	OPTIMA =33, EPNo =7, Years =3.98	M31004	2	1	320574	sCTL
411	7	Study Sample	OPTIMA =127, EPNo =7, Years =3.02	M31065	1	1	320575	AD
412	7	Study Sample	OPTIMA =33, EPNo =3, Years =1.97	M31002	2	1	320576	sCTL
413	7	Study Sample	OPTIMA =127, EPNo =3, Years =1.01	M31064	1	1	320577	AD
414	7	Study Sample	OPTIMA =33, EPNo =9, Years =4.96	M31005	2	1	320578	sCTL
415	7	Study Sample	OPTIMA =127, EPNo =11, Years =4.92	M31067	1	1	320579	AD
416	7	Study Sample	OPTIMA =331, EPNo =3, Years =1.08	M31168	2	5	320580	sCTL
417	7	QC	BAN MSBlock_7 #_19	BAN			320581	
418	7	QC	Conalbumin MSBlock_7 #_20	Conalbumin			320582	
419	7	Study Sample	OPTIMA =54, EPNo =1, Years =0	M31013	3	4	320583	
420	7	Study Sample	OPTIMA =54, EPNo =5, Years =2.14	M31014	3	4	320584	
421	7	QC	CSF Equil MSBlock_7 #_1 (Block7, inj #1)	CSF Equil			320585	
422	7	QC	CSF Equil MSBlock_7 #_2 (Block7, inj #1)	CSF Equil			320586	

423	7 QC	CSF Equil MSBlock_7 #_3 (Block7, inj #1)	CSF Equil			320587	
424	7 QC	CSF Equil MSBlock_7 #_4 (Block7, inj #1)	CSF Equil			320588	
425	7 QC	BAN MSBlock_7 #_5 (Block7, inj #1)	BAN			320589	
426	7 QC	BAN MSBlock_7 #_6 (Block7, inj #1)	BAN			320590	
427	7 QC	BAN MSBlock_7 #_7 (Block7, inj #1)	BAN			320591	
428	7 QC	Conalbumin MSBlock_7 #_8 (Block7, inj #1)	Conalbumin			320592	
429	7 Study Sample	OPTIMA =127, EPNo =9, Years =3.98	M31066	1	1	320593	AD
430	7 Study Sample	OPTIMA =33, EPNo =5, Years =2.98	M31003	2	1	320594	sCTL
431	7 Study Sample	OPTIMA =127, EPNo =1, Years =0	M31063	1	1	320595	AD
432	7 Study Sample	OPTIMA =33, EPNo =7, Years =3.98	M31004	2	1	320596	sCTL
433	7 Study Sample	OPTIMA =127, EPNo =7, Years =3.02	M31065	1	1	320597	AD
434	7 Study Sample	OPTIMA =33, EPNo =3, Years =1.97	M31002	2	1	320598	sCTL
435	7 Study Sample	OPTIMA =127, EPNo =3, Years =1.01	M31064	1	1	320599	AD
436	7 Study Sample	OPTIMA =33, EPNo =9, Years =4.96	M31005	2	1	320600	sCTL
437	7 Study Sample	OPTIMA =127, EPNo =11, Years =4.92	M31067	1	1	320601	AD
438	7 Study Sample	OPTIMA =331, EPNo =3, Years =1.08	M31168	2	5	320602	sCTL
439	7 QC	BAN MSBlock_7 #_19 (Block7, inj #1)	BAN			320603	
440	7 QC	Conalbumin MSBlock_7 #_20 (Block7, inj #1)	Conalbumin			320604	
441	7 Study Sample	OPTIMA =54, EPNo =1, Years =0	M31013	3	4	320605	sCTL
442	7 Study Sample	OPTIMA =54, EPNo =5, Years =2.14	M31014	3	4	320606	sCTL
443	7 Study Sample	OPTIMA =331, EPNo =1, Years =0	M31167	2	5	320607	sCTL
444	7 Study Sample	OPTIMA =54, EPNo =9, Years =3.98	M31015	3	4	320608	sCTL
445	7 Study Sample	OPTIMA =54, EPNo =11, Years =5.21	M31016	3	4	320609	sCTL
446	7 Study Sample	OPTIMA =156, EPNo =3, Years =1.12	M31081	3	1	320610	AD
447	7 Study Sample	OPTIMA =216, EPNo =3, Years =1.05	M31111	2	1	320611	pCTL.MCI
448	7 Study Sample	OPTIMA =156, EPNo =1, Years =0	M31080	3	1	320612	AD
449	7 Study Sample	OPTIMA =216, EPNo =5, Years =2.07	M31112	2	1	320613	pCTL.MCI
450	7 Study Sample	OPTIMA =156, EPNo =5, Years =2.12	M31082	3	1	320614	AD
451	7 QC	BAN MSBlock_7 #_31	BAN			320615	
452	7 QC	Conalbumin MSBlock_7 #_32	Conalbumin			320616	
453	7 Study Sample	OPTIMA =51, EPNo =3, Years =0.96	M31012	1	5	320617	sCTL
454	7 Study Sample	OPTIMA =216, EPNo =1, Years =0	M31110	2	1	320618	pCTL.MCI
455	7 Study Sample	OPTIMA =51, EPNo =1, Years =0	M31011	1	5	320619	sCTL
456	7 Study Sample	OPTIMA =63, EPNo =3, Years =0.99	M31026	1	5	320620	sCTL
457	7 Study Sample	OPTIMA =107, EPNo =3, Years =1.11	M31054	3	5	320621	AD
458	7 Study Sample	OPTIMA =63, EPNo =1, Years =0	M31025	1	5	320622	sCTL
459	7 Study Sample	OPTIMA =107, EPNo =1, Years =0	M31053	3	5	320623	AD
460	7 Study Sample	OPTIMA =63, EPNo =9, Years =3.99	M31027	1	5	320624	sCTL
461	7 Study Sample	OPTIMA =118, EPNo =1, Years =0	M31059	2	2	320625	AD
462	7 Study Sample	OPTIMA =241, EPNo =5, Years =2.01	M31139	3	6	320626	sCTL
463	7 QC	BAN MSBlock_7 #_43	BAN			320627	
464	7 QC	Conalbumin MSBlock_7 #_44	Conalbumin			320628	
465	7 Study Sample	OPTIMA =118, EPNo =3, Years =1.12	M31060	2	2	320629	AD
466	7 Study Sample	OPTIMA =241, EPNo =1, Years =0	M31137	3	6	320630	pMCI
467	7 Study Sample	OPTIMA =121, EPNo =3, Years =1.12	M31062	2	2	320631	AD
468	7 Study Sample	OPTIMA =241, EPNo =3, Years =1.02	M31138	3	6	320632	sCTL
469	7 Study Sample	OPTIMA =173, EPNo =3, Years =0.99	M31091	3	3	320633	AD

470	7	Study Sample	OPTIMA =121, EPNo =1, Years =0	M31061	2	2	320634	AD
471	7	Study Sample	OPTIMA =173, EPNo =1, Years =0	M31090	3	3	320635	AD
472	7	Study Sample	OPTIMA =281, EPNo =5, Years =2.06	M31159	4	6	320636	sCTL
473	7	Study Sample	OPTIMA =188, EPNo =1, Years =0	M31098	3	6	320637	ODS
474	7	QC	BAN MSBlock_7 #_54	BAN			320638	
475	7	QC	Conalbumin MSBlock_7 #_55	Conalbumin			320639	
476	7	Study Sample	OPTIMA =281, EPNo =7, Years =3.03	M31160	4	6	320640	sCTL
477	7	Study Sample	OPTIMA =225, EPNo =3, Years =1.01	M31124	4	2	320641	pMCI
478	7	Study Sample	OPTIMA =188, EPNo =3, Years =1.04	M31099	3	6	320642	pCTL.AD
479	7	Study Sample	OPTIMA =281, EPNo =1, Years =0	M31158	4	6	320643	sCTL
480	7	Study Sample	OPTIMA =225, EPNo =1, Years =0	M31123	4	2	320644	pMCI
481	7	Study Sample	OPTIMA =127, EPNo =9, Years =3.98	M31066	1	1	320645	AD
482	7	Study Sample	OPTIMA =54, EPNo =11, Years =5.21	M31016	3	4	320646	sCTL
483	7	QC	BAN MSBlock_7 #_61	BAN			320647	
484	7	QC	Conalbumin MSBlock_7 #_62	Conalbumin			320648	
485	8	QC	CSF Equil MSBlock_8 #_1	CSF Equil			320649	
486	8	QC	CSF Equil MSBlock_8 #_2	CSF Equil			320650	
487	8	QC	CSF Equil MSBlock_8 #_3	CSF Equil			320651	
488	8	QC	CSF Equil MSBlock_8 #_4	CSF Equil			320652	
489	8	QC	BAN MSBlock_8 #_5	BAN			320653	
490	8	QC	BAN MSBlock_8 #_6	BAN			320654	
491	8	QC	BAN MSBlock_8 #_7	BAN			320655	
492	8	QC	Conalbumin MSBlock_8 #_8	Conalbumin			320656	
493	8	Study Sample	OPTIMA =219, EPNo =1, Years =0	M31113	3	1	320657	pCTL.MCI
494	8	Study Sample	OPTIMA =114, EPNo =7, Years =3.1	M31058	4	2	320658	AD
495	8	Study Sample	OPTIMA =219, EPNo =5, Years =1.91	M31115	3	1	320659	pMCI
496	8	Study Sample	OPTIMA =114, EPNo =3, Years =1.16	M31056	4	2	320660	AD
497	8	Study Sample	OPTIMA =114, EPNo =1, Years =0	M31055	4	2	320661	AD
498	8	Study Sample	OPTIMA =219, EPNo =3, Years =1.01	M31114	3	1	320662	pMCI
499	8	Study Sample	OPTIMA =114, EPNo =5, Years =2.14	M31057	4	2	320663	AD
500	8	Study Sample	OPTIMA =178, EPNo =7, Years =3.14	M31095	2	3	320664	ODS
501	8	Study Sample	OPTIMA =220, EPNo =5, Years =2.03	M31118	1	3	320665	pMCI
502	8	Study Sample	OPTIMA =178, EPNo =1, Years =0	M31092	2	3	320666	ODS
503	8	QC	BAN MSBlock_8 #_19	BAN			320667	
504	8	QC	Conalbumin MSBlock_8 #_20	Conalbumin			320668	
505	8	Study Sample	OPTIMA =220, EPNo =3, Years =1.07	M31117	1	3	320669	pMCI
506	8	Study Sample	OPTIMA =178, EPNo =5, Years =2.13	M31094	2	3	320670	ODS
507	8	Study Sample	OPTIMA =220, EPNo =1, Years =0	M31116	1	3	320671	pMCI
508	8	Study Sample	OPTIMA =178, EPNo =3, Years =1.11	M31093	2	3	320672	ODS
509	8	Study Sample	OPTIMA =220, EPNo =7, Years =2.97	M31119	1	3	320673	pMCI
510	8	Study Sample	OPTIMA =158, EPNo =3, Years =1.16	M31084	3	5	320674	AD
511	8	Study Sample	OPTIMA =158, EPNo =5, Years =2.14	M31085	3	5	320675	AD
512	8	Study Sample	OPTIMA =257, EPNo =9, Years =4	M31153	1	4	320676	sCTL
513	8	Study Sample	OPTIMA =158, EPNo =1, Years =0	M31083	3	5	320677	AD
514	8	Study Sample	OPTIMA =271, EPNo =3, Years =1.09	M31155	4	4	320678	sCTL
515	8	QC	BAN MSBlock_8 #_31	BAN			320679	
516	8	QC	Conalbumin MSBlock_8 #_32	Conalbumin			320680	

517	8 Study Sample	OPTIMA =257, EPNo =1, Years =0	M31151	1	4	320681	sCTL
518	8 Study Sample	OPTIMA =257, EPNo =3, Years =1.15	M31152	1	4	320682	sCTL
519	8 Study Sample	OPTIMA =271, EPNo =1, Years =0	M31154	4	4	320683	sCTL
520	8 Study Sample	OPTIMA =48, EPNo =7, Years =3.2	M31010	3	5	320684	sCTL
521	8 Study Sample	OPTIMA =212, EPNo =3, Years =0.94	M31105	4	1	320685	pCTL.MCI
522	8 Study Sample	OPTIMA =48, EPNo =1, Years =0	M31008	3	5	320686	sCTL
523	8 Study Sample	OPTIMA =212, EPNo =1, Years =0	M31104	4	1	320687	pCTL.MCI
524	8 Study Sample	OPTIMA =48, EPNo =3, Years =1.21	M31009	3	5	320688	sCTL
525	8 Study Sample	OPTIMA =279, EPNo =1, Years =0	M31156	4	4	320689	sCTL
526	8 Study Sample	OPTIMA =246, EPNo =5, Years =1.93	M31144	3	6	320690	sCTL
527	8 QC	BAN MSBlock_8 #_43	BAN			320691	
528	8 QC	Conalbumin MSBlock_8 #_44	Conalbumin			320692	
529	8 Study Sample	OPTIMA =279, EPNo =3, Years =0.93	M31157	4	4	320693	sCTL
530	8 Study Sample	OPTIMA =246, EPNo =1, Years =0	M31142	3	6	320694	sCTL
531	8 Study Sample	OPTIMA =301, EPNo =3, Years =0.98	M31166	4	5	320695	sCTL
532	8 Study Sample	OPTIMA =246, EPNo =3, Years =0.95	M31143	3	6	320696	sCTL
533	8 Study Sample	OPTIMA =301, EPNo =1, Years =0	M31165	4	5	320697	sCTL
534	8 Study Sample	OPTIMA =245, EPNo =3, Years =1.13	M31141	4	2	320698	sCTL
535	8 Study Sample	OPTIMA =255, EPNo =3, Years =1.2	M31149	4	4	320699	sCTL
536	8 Study Sample	OPTIMA =443, EPNo =1, Years =0	M31169	4	6	320700	sCTL
537	8 Study Sample	OPTIMA =255, EPNo =5, Years =2.2	M31150	4	4	320701	sCTL
538	8 Study Sample	OPTIMA =237, EPNo =3, Years =1.08	M31136	2	5	320702	pMCI
539	8 QC	BAN MSBlock_8 #_55	BAN			320703	
540	8 QC	Conalbumin MSBlock_8 #_56	Conalbumin			320704	
541	8 Study Sample	OPTIMA =443, EPNo =3, Years =1.04	M31170	4	6	320705	sCTL
542	8 Study Sample	OPTIMA =255, EPNo =1, Years =0	M31148	4	4	320706	sCTL
543	8 Study Sample	OPTIMA =237, EPNo =1, Years =0	M31135	2	5	320707	pMCI
544	8 QC	BAN MSBlock_8 #_60	BAN			320708	
545	8 QC	Conalbumin MSBlock_8 #_61	Conalbumin			320709	
546	9 QC	CSF Equil MSBlock_9 #_1	CSF Equil			320710	
547	9 QC	CSF Equil MSBlock_9 #_2	CSF Equil			320711	
548	9 QC	CSF Equil MSBlock_9 #_3	CSF Equil			320712	
549	9 QC	CSF Equil MSBlock_9 #_4	CSF Equil			320713	
550	9 QC	BAN MSBlock_9 #_5	BAN			320714	
551	9 QC	BAN MSBlock_9 #_6	BAN			320715	
552	9 QC	BAN MSBlock_9 #_7	BAN			320716	
553	9 QC	Conalbumin MSBlock_9 #_8	Conalbumin			320717	
554	9 QC	1418-OPT-P004-PosCtrl-1-Agilent-1	M31171	1	1	320718	
555	9 QC	1418-OPT-P004-PosCtrl-2-Agilent-1	M31172	1	2	320719	
556	9 QC	1418-OPT-P004-PosCtrl-3-Agilent-1	M31173	1	3	320720	
557	9 QC	1418-OPT-P004-PosCtrl-4-Agilent-1	M31174	1	4	320721	
558	9 QC	1418-OPT-P004-PosCtrl-5-Agilent-1	M31175	1	5	320722	
559	9 QC	1418-OPT-P004-PosCtrl-6-Agilent-1	M31176	1	6	320723	
560	9 QC	1418-OPT-P004-PosCtrl-7-Agilent-1	M31177	1	1	320724	
561	9 QC	1418-OPT-P004-PosCtrl-8-Agilent-1	M31178	1	2	320725	
562	9 QC	1418-OPT-P004-PosCtrl-9-Agilent-1	M31179	1	3	320726	
563	9 QC	BAN MSBlock_9 #_18	BAN			320727	

564	9 QC	Conalbumin MSBlock_9 #_19	Conalbumin			320728
565	9 QC	1418-OPT-P004-PosCtrl-10-Agilent-1	M31180	1	4	320729
566	9 QC	1418-OPT-P004-PosCtrl-11-Agilent-1	M31181	2	1	320730
567	9 QC	1418-OPT-P004-PosCtrl-12-Agilent-1	M31182	2	2	320731
568	9 QC	1418-OPT-P004-PosCtrl-13-Agilent-1	M31183	2	3	320732
569	9 QC	1418-OPT-P004-PosCtrl-14-Agilent-1	M31184	2	4	320733
570	9 QC	1418-OPT-P004-PosCtrl-15-Agilent-1	M31185	2	5	320734
571	9 QC	1418-OPT-P004-PosCtrl-16-Agilent-1	M31186	2	6	320735
572	9 QC	1418-OPT-P004-PosCtrl-17-Agilent-1	M31187	2	1	320736
573	9 QC	1418-OPT-P004-PosCtrl-18-Agilent-1	M31188	2	2	320737
574	9 QC	1418-OPT-P004-PosCtrl-19-Agilent-1	M31189	2	3	320738
575	9 QC	BAN MSBlock_9 #_30	BAN			320739
576	9 QC	Conalbumin MSBlock_9 #_31	Conalbumin			320740
577	9 QC	1418-OPT-P004-PosCtrl-20-Agilent-1	M31190	2	5	320741
578	9 QC	1418-OPT-P004-PosCtrl-21-Agilent-1	M31191	3	1	320742
579	9 QC	1418-OPT-P004-PosCtrl-22-Agilent-1	M31192	3	2	320743
580	9 QC	1418-OPT-P004-PosCtrl-23-Agilent-1	M31193	3	3	320744
581	9 QC	1418-OPT-P004-PosCtrl-24-Agilent-1	M31194	3	4	320745
582	9 QC	1418-OPT-P004-PosCtrl-25-Agilent-1	M31195	3	5	320746
583	9 QC	1418-OPT-P004-PosCtrl-26-Agilent-1	M31196	3	6	320747
584	9 QC	1418-OPT-P004-PosCtrl-27-Agilent-1	M31197	3	1	320748
585	9 QC	1418-OPT-P004-PosCtrl-28-Agilent-1	M31198	3	2	320749
586	9 QC	BAN MSBlock_9 #_41	BAN			320750
587	9 QC	Conalbumin MSBlock_9 #_42	Conalbumin			320751
588	9 QC	1418-OPT-P004-PosCtrl-29-Agilent-1	M31199	3	3	320752
589	9 QC	1418-OPT-P004-PosCtrl-30-Agilent-1	M31200	3	4	320753
590	9 QC	1418-OPT-P004-PosCtrl-31-Agilent-1	M31201	4	1	320754
591	9 QC	1418-OPT-P004-PosCtrl-32-Agilent-1	M31202	4	2	320755
592	9 QC	1418-OPT-P004-PosCtrl-33-Agilent-1	M31203	4	3	320756
593	9 QC	1418-OPT-P004-PosCtrl-34-Agilent-1	M31204	4	4	320757
594	9 QC	1418-OPT-P004-PosCtrl-35-Agilent-1	M31205	4	5	320758
595	9 QC	1418-OPT-P004-PosCtrl-36-Agilent-1	M31206	4	6	320759
596	9 QC	1418-OPT-P004-PosCtrl-37-Agilent-1	M31207	4	1	320760
597	9 QC	BAN MSBlock_9 #_52	BAN			320761
598	9 QC	Conalbumin MSBlock_9 #_53	Conalbumin			320762
599	9 QC	1418-OPT-P004-PosCtrl-38-Agilent-1	M31208	4	2	320763
600	9 QC	1418-OPT-P004-PosCtrl-39-Agilent-1	M31209	4	3	320764
601	9 QC	QC_01	BAN_01			320765
602	9 QC	QC_01	Conalbumin_01			320766
603	9 QC	QC_02	BAN_02			320767
604	9 QC	QC_02	Conalbumin_02			320768
