# Supplementary material for: High Resolution Discovery Proteomics Reveals Candidate Disease Progression Markers of Alzheimer’s Disease in Human Cerebrospinal Fluid
Source: PLoS One. 2015 Aug 13;10(8):e0135365. doi: 10.1371/journal.pone.0135365 (PMC4535975; doi:10.1371/journal.pone.0135365)
Supplement: S4 Table — (PDF) [file pone.0135365.s007.pdf]

	Feature ID	751080736	751082516				
	Peak Centroid m/z	639.629	635.978				
	Peak Centroid Time	24.826	22.267	File Name	Description	Sample Name	Dx
1	Intensity [809175]	47366.461	18475.074	320173	OPTIMA =229, EPNo =11, Years =5.04	M31133	pMCI
2	Intensity [809281]	54222.992	22390.539	320175	OPTIMA =229, EPNo =13, Years =5.94	M31134	pMCI
3	Intensity [809409]	56005.656	16554.812	320177	OPTIMA =229, EPNo =3, Years =1.07	M31129	pMCI
4	Intensity [809479]	65052.219	24273.898	320179	OPTIMA =229, EPNo =5, Years =2.03	M31130	pMCI
5	Intensity [809549]	52395.516	19215.512	320181	OPTIMA =229, EPNo =1, Years =0	M31128	pMCI
6	Intensity [809693]	65127.734	11600.41	320185	OPTIMA =229, EPNo =7, Years =3.02	M31131	pMCI
7	Intensity [809768]	40193.133	19256.371	320187	OPTIMA =229, EPNo =9, Years =4.06	M31132	pMCI
8	Intensity [809869]	17918.395	8498.25	320190	OPTIMA =74, EPNo =5, Years =2.09	M31032	sCTL
9	Intensity [809938]	13761.064	8837.122	320192	OPTIMA =74, EPNo =3, Years =1	M31031	sCTL
10	Intensity [810019]	22608.303	12226.818	320194	OPTIMA =74, EPNo =1, Years =0	M31030	sCTL
11	Intensity [810211]	18004.604	18074.896	320198	OPTIMA =102, EPNo =5, Years =1.94	M31046	AD
12	Intensity [810316]	17153.541	16146.535	320200	OPTIMA =102, EPNo =1, Years =0	M31045	AD
13	Intensity [810391]	20298.779	11915.255	320202	OPTIMA =102, EPNo =7, Years =2.93	M31047	AD
14	Intensity [810753]	11597.91	10452.768	320210	OPTIMA =66, EPNo =5, Years =2.12	M31029	sCTL
15	Intensity [810874]	16416.027	15527.081	320212	OPTIMA =66, EPNo =1, Years =0	M31028	sCTL
16	Intensity [811158]	27120.297	10593.011	320215	OPTIMA =84, EPNo =1, Years =0	M31037	sCTL
17	Intensity [811407]	38481.664	11470.803	320218	OPTIMA =84, EPNo =3, Years =1.06	M31038	sCTL
18	Intensity [811648]	5727.94	4361.952	320222	OPTIMA =91, EPNo =3, Years =1.12	M31039	sCTL
19	Intensity [811864]	6538.816	4336.864	320225	OPTIMA =91, EPNo =5, Years =2.16	M31040	sCTL
20	Intensity [813481]	82353.477	15730.792	320240	OPTIMA =105, EPNo =3, Years =1.16	M31049	AD
21	Intensity [813601]	73996.484	18187.684	320242	OPTIMA =105, EPNo =7, Years =3.12	M31051	AD
22	Intensity [813732]	55878.031	20162.215	320244	OPTIMA =105, EPNo =5, Years =2.13	M31050	AD
23	Intensity [814245]	24379.41	9646.006	320252	OPTIMA =252, EPNo =1, Years =0	M31145	sCTL
24	Intensity [814369]	14400.014	4737.787	320254	OPTIMA =252, EPNo =5, Years =2.19	M31147	sCTL
25	Intensity [814471]	12369.334	6514.544	320256	OPTIMA =252, EPNo =3, Years =1.1	M31146	sCTL
26	Intensity [814816]	34831.645	18844.479	320261	OPTIMA =147, EPNo =3, Years =1.09	M31074	AD
27	Intensity [814941]	22873.555	22943.445	320263	OPTIMA =147, EPNo =1, Years =0	M31073	AD
28	Intensity [815090]	33896.258	20817.301	320265	OPTIMA =147, EPNo =5, Years =1.97	M31075	AD
29	Intensity [815228]	25562.098	17296.297	320267	OPTIMA =37, EPNo =1, Years =0	M31006	sCTL
30	Intensity [815375]	26307.348	15607.936	320269	OPTIMA =37, EPNo =3, Years =1.15	M31007	sCTL
31	Intensity [815639]	17306.492	7056.44	320273	OPTIMA =95, EPNo =3, Years =1.01	M31042	sCTL
32	Intensity [815785]	29360.604	15941.396	320275	OPTIMA =95, EPNo =1, Years =0	M31041	sCTL
33	Intensity [815916]	12387.319	8625.85	320277	OPTIMA =100, EPNo =1, Years =0	M31043	AD
34	Intensity [816134]	10668.58	5720.723	320280	OPTIMA =100, EPNo =3, Years =1.17	M31044	AD
35	Intensity [819347]	17674.662	16103.648	320310	OPTIMA =33, EPNo =5, Years =2.98	M31003	sCTL
36	Intensity [819472]	11230.998	11644.568	320312	OPTIMA =33, EPNo =7, Years =3.98	M31004	sCTL
37	Intensity [819611]	24008.521	24394.957	320314	OPTIMA =33, EPNo =3, Years =1.97	M31002	sCTL
38	Intensity [819762]	8637.624	7334.342	320316	OPTIMA =33, EPNo =9, Years =4.96	M31005	sCTL
39	Intensity [820406]	13164.601	12460.148	320326	OPTIMA =156, EPNo =3, Years =1.12	M31081	AD
40	Intensity [820551]	12935.473	7311.68	320328	OPTIMA =156, EPNo =1, Years =0	M31080	AD
41	Intensity [820683]	13383.623	4402.771	320330	OPTIMA =156, EPNo =5, Years =2.12	M31082	AD
42	Intensity [820922]	10988.572	5448.142	320333	OPTIMA =51, EPNo =3, Years =0.96	M31012	sCTL
43	Intensity [821060]	17282.932	5455.5	320335	OPTIMA =51, EPNo =1, Years =0	M31011	sCTL
44	Intensity [821185]	8928.305	11354.578	320337	OPTIMA =107, EPNo =3, Years =1.11	M31054	AD
45	Intensity [821308]	15884.217	20019.637	320339	OPTIMA =107, EPNo =1, Years =0	M31053	AD
46	Intensity [821435]	28072.789	21105.371	320341	OPTIMA =118, EPNo =1, Years =0	M31059	AD
47	Intensity [821665]	30304.48	15436.188	320345	OPTIMA =118, EPNo =3, Years =1.12	M31060	AD
48	Intensity [821791]	9390.327	16774.227	320347	OPTIMA =121, EPNo =3, Years =1.12	M31062	AD
49	Intensity [821991]	13101.674	20667.617	320350	OPTIMA =121, EPNo =1, Years =0	M31061	AD
50	Intensity [822149]	16413.719	12665.179	320353	OPTIMA =188, EPNo =1, Years =0	M31098	ODS
51	Intensity [822440]	30570.98	10351.364	320358	OPTIMA =188, EPNo =3, Years =1.04	M31099	pCTL.AD
52	Intensity [824039]	23862.412	15291.174	320373	OPTIMA =114, EPNo =7, Years =3.1	M31058	AD
53	Intensity [824299]	35379.582	15008.818	320375	OPTIMA =114, EPNo =3, Years =1.16	M31056	AD
54	Intensity [824393]	40735.52	21162.756	320376	OPTIMA =114, EPNo =1, Years =0	M31055	AD
55	Intensity [824419]	23396.314	9349.729	320378	OPTIMA =114, EPNo =5, Years =2.14	M31057	AD
56	Intensity [824424]	15709.824	11704.861	320379	OPTIMA =178, EPNo =7, Years =3.14	M31095	ODS
57	Intensity [824434]	21634.543	17167.205	320381	OPTIMA =178, EPNo =1, Years =0	M31092	ODS
58	Intensity [824572]	15298.939	10044.918	320385	OPTIMA =178, EPNo =5, Years =2.13	M31094	ODS
59	Intensity [824691]	13034.486	14184.887	320387	OPTIMA =178, EPNo =3, Years =1.11	M31093	ODS
60	Intensity [824803]	7400.756	5793.404	320389	OPTIMA =158, EPNo =3, Years =1.16	M31084	AD
61	Intensity [824859]	9948.737	7020.818	320390	OPTIMA =158, EPNo =5, Years =2.14	M31085	AD
62	Intensity [824982]	17075.73	8517.562	320392	OPTIMA =158, EPNo =1, Years =0	M31083	AD
63	Intensity [825069]	29571.67	14877.532	320393	OPTIMA =271, EPNo =3, Years =1.09	M31155	sCTL
64	Intensity [825377]	34021.375	16760.557	320398	OPTIMA =271, EPNo =1, Years =0	M31154	sCTL
65	Intensity [826169]	17604.535	7599.067	320410	OPTIMA =301, EPNo =3, Years =0.98	M31166	sCTL
66	Intensity [826282]	15889.811	9933.112	320412	OPTIMA =301, EPNo =1, Years =0	M31165	sCTL
67	Intensity [828645]	36044.805	19500.145	320437	OPTIMA =229, EPNo =11, Years =5.04	M31133	pMCI
68	Intensity [828758]	56116.891	23848.605	320439	OPTIMA =229, EPNo =13, Years =5.94	M31134	pMCI

69	Intensity [828869]	43795.227	20892.049	320441	OPTIMA =229, EPNo =3, Years =1.07	M31129	pMCI
70	Intensity [829005]	62685.891	32320.695	320443	OPTIMA =229, EPNo =5, Years =2.03	M31130	pMCI
71	Intensity [829171]	50952.895	25798.342	320445	OPTIMA =229, EPNo =1, Years =0	M31128	pMCI
72	Intensity [829504]	49917.469	18075.812	320449	OPTIMA =229, EPNo =7, Years =3.02	M31131	pMCI
73	Intensity [829669]	38058.805	20424.459	320451	OPTIMA =229, EPNo =9, Years =4.06	M31132	pMCI
74	Intensity [830676]	18777.398	11071.506	320462	OPTIMA =74, EPNo =5, Years =2.09	M31032	sCTL
75	Intensity [830838]	11960.18	12586.79	320464	OPTIMA =74, EPNo =3, Years =1	M31031	sCTL
76	Intensity [831014]	17251.9	13438.599	320466	OPTIMA =74, EPNo =1, Years =0	M31030	sCTL
77	Intensity [831338]	15136.217	16399.102	320470	OPTIMA =102, EPNo =5, Years =1.94	M31046	AD
78	Intensity [831497]	13904.746	19613.176	320472	OPTIMA =102, EPNo =1, Years =0	M31045	AD
79	Intensity [831667]	12592.551	14494.191	320474	OPTIMA =102, EPNo =7, Years =2.93	M31047	AD
80	Intensity [832325]	11733.346	13410.118	320482	OPTIMA =66, EPNo =5, Years =2.12	M31029	sCTL
81	Intensity [832489]	19227.568	18507.486	320484	OPTIMA =66, EPNo =1, Years =0	M31028	sCTL
82	Intensity [832740]	20701.086	11758.547	320487	OPTIMA =84, EPNo =1, Years =0	M31037	sCTL
83	Intensity [832981]	24535.988	9567.77	320490	OPTIMA =84, EPNo =3, Years =1.06	M31038	sCTL
84	Intensity [833401]	4672.511	3175.845	320494	OPTIMA =91, EPNo =3, Years =1.12	M31039	sCTL
85	Intensity [833745]	5838.401	4495.859	320497	OPTIMA =91, EPNo =5, Years =2.16	M31040	sCTL
86	Intensity [835230]	55690	19121.961	320510	OPTIMA =105, EPNo =13, Years =6.12	M31052	AD
87	Intensity [835659]	25326.582	12459.332	320524	OPTIMA =252, EPNo =1, Years =0	M31145	sCTL
88	Intensity [835718]	16205.523	5557.428	320526	OPTIMA =252, EPNo =5, Years =2.19	M31147	sCTL
89	Intensity [835779]	16757.789	7169.875	320528	OPTIMA =252, EPNo =3, Years =1.1	M31146	sCTL
90	Intensity [835949]	34947.141	25349.48	320533	OPTIMA =147, EPNo =3, Years =1.09	M31074	AD
91	Intensity [836026]	25849.152	26796.977	320535	OPTIMA =147, EPNo =1, Years =0	M31073	AD
92	Intensity [836090]	31312.633	32848.406	320537	OPTIMA =147, EPNo =5, Years =1.97	M31075	AD
93	Intensity [836149]	26582.359	19985.373	320539	OPTIMA =37, EPNo =1, Years =0	M31006	sCTL
94	Intensity [836233]	31490.633	19939.605	320541	OPTIMA =37, EPNo =3, Years =1.15	M31007	sCTL
95	Intensity [836413]	20789.656	12291.176	320545	OPTIMA =95, EPNo =3, Years =1.01	M31042	sCTL
96	Intensity [836492]	33799.219	19815.064	320547	OPTIMA =95, EPNo =1, Years =0	M31041	sCTL
97	Intensity [836542]	13998.232	12283.918	320549	OPTIMA =100, EPNo =1, Years =0	M31043	AD
98	Intensity [836612]	11317.936	7962.537	320552	OPTIMA =100, EPNo =3, Years =1.17	M31044	AD
99	Intensity [838918]	14109.621	15131.911	320594	OPTIMA =33, EPNo =5, Years =2.98	M31003	sCTL
100	Intensity [838956]	8676.066	11089.956	320596	OPTIMA =33, EPNo =7, Years =3.98	M31004	sCTL
101	Intensity [838984]	18726.314	29273.668	320598	OPTIMA =33, EPNo =3, Years =1.97	M31002	sCTL
102	Intensity [839011]	7872.565	7583.321	320600	OPTIMA =33, EPNo =9, Years =4.96	M31005	sCTL
103	Intensity [839184]	8072.375	11423.941	320610	OPTIMA =156, EPNo =3, Years =1.12	M31081	AD
104	Intensity [839219]	9021.639	5146.816	320612	OPTIMA =156, EPNo =1, Years =0	M31080	AD
105	Intensity [839257]	9478.207	3849.471	320614	OPTIMA =156, EPNo =5, Years =2.12	M31082	AD
106	Intensity [839308]	7936.91	4903.26	320617	OPTIMA =51, EPNo =3, Years =0.96	M31012	sCTL
107	Intensity [839343]	13791.634	4801.814	320619	OPTIMA =51, EPNo =1, Years =0	M31011	sCTL
108	Intensity [839378]	7357.368	9364.831	320621	OPTIMA =107, EPNo =3, Years =1.11	M31054	AD
109	Intensity [839413]	11150.439	14542.863	320623	OPTIMA =107, EPNo =1, Years =0	M31053	AD
110	Intensity [839450]	20606.359	18700.309	320625	OPTIMA =118, EPNo =1, Years =0	M31059	AD
111	Intensity [839519]	19768.895	11739.901	320629	OPTIMA =118, EPNo =3, Years =1.12	M31060	AD
112	Intensity [839560]	6932.368	16918.223	320631	OPTIMA =121, EPNo =3, Years =1.12	M31062	AD
113	Intensity [839640]	10197.193	17785.133	320634	OPTIMA =121, EPNo =1, Years =0	M31061	AD
114	Intensity [839707]	11005.159	9212.961	320637	OPTIMA =188, EPNo =1, Years =0	M31098	ODS
115	Intensity [839829]	16652.141	6394.959	320642	OPTIMA =188, EPNo =3, Years =1.04	M31099	pCTL.AD
116	Intensity [840387]	7138.538	10050.993	320658	OPTIMA =114, EPNo =7, Years =3.1	M31058	AD
117	Intensity [840454]	23177.355	10978.377	320660	OPTIMA =114, EPNo =3, Years =1.16	M31056	AD
118	Intensity [840498]	27947.738	13939.293	320661	OPTIMA =114, EPNo =1, Years =0	M31055	AD
119	Intensity [840573]	9054.778	5635.565	320663	OPTIMA =114, EPNo =5, Years =2.14	M31057	AD
120	Intensity [840710]	10396.062	7800.37	320664	OPTIMA =178, EPNo =7, Years =3.14	M31095	ODS
121	Intensity [840891]	14636.365	11070.732	320666	OPTIMA =178, EPNo =1, Years =0	M31092	ODS
122	Intensity [841050]	9495.2	6377.734	320670	OPTIMA =178, EPNo =5, Years =2.13	M31094	ODS
123	Intensity [841147]	9949.982	12465.832	320672	OPTIMA =178, EPNo =3, Years =1.11	M31093	ODS
124	Intensity [841242]	5719.258	4439.874	320674	OPTIMA =158, EPNo =3, Years =1.16	M31084	AD
125	Intensity [841290]	8334.401	6385.896	320675	OPTIMA =158, EPNo =5, Years =2.14	M31085	AD
126	Intensity [841400]	9527.421	6296.457	320677	OPTIMA =158, EPNo =1, Years =0	M31083	AD
127	Intensity [841453]	21848.117	12584.837	320678	OPTIMA =271, EPNo =3, Years =1.09	M31155	sCTL
128	Intensity [841761]	25723.408	13737.104	320683	OPTIMA =271, EPNo =1, Years =0	M31154	sCTL
129	Intensity [842155]	15926.613	8578.912	320695	OPTIMA =301, EPNo =3, Years =0.98	M31166	sCTL
130	Intensity [842216]	7166.391	7029.471	320697	OPTIMA =301, EPNo =1, Years =0	M31165	sCTL
131	Intensity [809230]	15688.6	9431.209	320174	OPTIMA =16, EPNo =5, Years =2.01	M30996	AD
132	Intensity [809367]	27019.195	11975.903	320176	OPTIMA =16, EPNo =7, Years =2.98	M30997	AD
133	Intensity [809444]	10283.305	5855.12	320178	OPTIMA =16, EPNo =15, Years =6.96	M31001	AD
134	Intensity [809513]	10948.164	7782.336	320180	OPTIMA =16, EPNo =11, Years =5	M30999	AD
135	Intensity [809586]	13317.098	10537.309	320182	OPTIMA =16, EPNo =13, Years =5.97	M31000	AD
136	Intensity [809727]	25476.41	14580.656	320186	OPTIMA =16, EPNo =3, Years =0.99	M30995	AD
137	Intensity [809802]	12520.501	13337.653	320188	OPTIMA =16, EPNo =9, Years =3.98	M30998	AD
138	Intensity [809835]	118906.125	17156.432	320189	OPTIMA =150, EPNo =3, Years =1.5	M31077	AD
139	Intensity [809903]	111535.266	18317.273	320191	OPTIMA =150, EPNo =7, Years =3.32	M31079	AD

140	Intensity [809984]	119597.406	20553.746	320193	OPTIMA =150, EPNo =5, Years =2.37	M31078	AD
141	Intensity [810153]	101708.906	19386.131	320197	OPTIMA =150, EPNo =1, Years =0	M31076	AD
142	Intensity [810261]	44870.969	17036.488	320199	OPTIMA =159, EPNo =5, Years =2.05	M31088	AD
143	Intensity [810354]	38966.68	15081.756	320201	OPTIMA =159, EPNo =3, Years =1	M31087	AD
144	Intensity [810429]	40641.031	14774.625	320203	OPTIMA =159, EPNo =1, Years =0	M31086	AD
145	Intensity [810473]	49474.328	22772.781	320204	OPTIMA =57, EPNo =3, Years =1.08	M31018	sCTL
146	Intensity [810521]	49558.473	13727.971	320205	OPTIMA =159, EPNo =7, Years =2.99	M31089	AD
147	Intensity [810560]	43226.82	13356.912	320206	OPTIMA =57, EPNo =1, Years =0	M31017	sCTL
148	Intensity [810715]	34755.766	22518.521	320209	OPTIMA =214, EPNo =3, Years =0.97	M31107	pCTL.MCI
149	Intensity [810803]	36729.102	21853.027	320211	OPTIMA =214, EPNo =1, Years =0	M31106	pCTL.MCI
150	Intensity [810914]	22395.535	12953.955	320213	OPTIMA =214, EPNo =5, Years =2.1	M31108	pCTL.MCI
151	Intensity [811047]	51306.555	33552.137	320214	OPTIMA =137, EPNo =3, Years =0.93	M31069	AD
152	Intensity [811228]	50056.305	28253.535	320216	OPTIMA =137, EPNo =1, Years =0	M31068	AD
153	Intensity [811325]	39310.25	19112.559	320217	OPTIMA =222, EPNo =5, Years =1.97	M31122	pMCI
154	Intensity [811574]	64097.754	31540.213	320221	OPTIMA =222, EPNo =1, Years =0	M31120	pMCI
155	Intensity [811723]	46450.523	22249.982	320223	OPTIMA =222, EPNo =3, Years =0.96	M31121	pMCI
156	Intensity [811796]	11617.707	8986.884	320224	OPTIMA =300, EPNo =1, Years =0	M31163	sCTL
157	Intensity [811920]	12884.386	8270.049	320226	OPTIMA =300, EPNo =3, Years =1.03	M31164	sCTL
158	Intensity [813234]	40410.215	13114.565	320237	OPTIMA =105, EPNo =1, Years =0	M31048	AD
159	Intensity [813323]	75278.977	15287.799	320238	OPTIMA =105, EPNo =13, Years =6.12	M31052	AD
160	Intensity [813397]	29479.994	14511.562	320239	OPTIMA =59, EPNo =7, Years =3.24	M31022	sCTL
161	Intensity [813537]	36255.262	13634.637	320241	OPTIMA =59, EPNo =5, Years =2.2	M31021	sCTL
162	Intensity [813661]	40755.953	13045.866	320243	OPTIMA =59, EPNo =1, Years =0	M31019	sCTL
163	Intensity [813791]	20596.613	6328.015	320245	OPTIMA =59, EPNo =9, Years =4.19	M31023	sCTL
164	Intensity [813848]	18907.289	6179.393	320246	OPTIMA =59, EPNo =11, Years =5.19	M31024	sCTL
165	Intensity [814050]	20636.492	19726.168	320249	OPTIMA =82, EPNo =3, Years =1.03	M31033	sCTL
166	Intensity [814118]	47699.383	17411.779	320250	OPTIMA =59, EPNo =3, Years =1.1	M31020	sCTL
167	Intensity [814173]	23665.426	21344.014	320251	OPTIMA =82, EPNo =7, Years =3.07	M31035	sCTL
168	Intensity [814299]	19742.566	17190.191	320253	OPTIMA =82, EPNo =5, Years =1.96	M31034	sCTL
169	Intensity [814420]	18663.91	19846.129	320255	OPTIMA =82, EPNo =9, Years =4.01	M31036	sCTL
170	Intensity [814535]	17340.367	14304.59	320257	OPTIMA =207, EPNo =5, Years =2.09	M31102	pCTL.AD
171	Intensity [814607]	20718.389	13801.277	320258	OPTIMA =207, EPNo =1, Years =0	M31100	pCTL.AD
172	Intensity [814883]	20803.301	16291.553	320262	OPTIMA =207, EPNo =7, Years =3.1	M31103	pCTL.AD
173	Intensity [815029]	17296.184	11564.561	320264	OPTIMA =207, EPNo =3, Years =1.07	M31101	pCTL.AD
174	Intensity [815151]	53565.109	26489.062	320266	OPTIMA =145, EPNo =3, Years =1.14	M31071	AD
175	Intensity [815297]	42198.031	18156.805	320268	OPTIMA =145, EPNo =7, Years =3.09	M31072	AD
176	Intensity [815448]	37927.68	21457.039	320270	OPTIMA =145, EPNo =1, Years =0	M31070	AD
177	Intensity [815699]	57853.008	39504.871	320274	OPTIMA =226, EPNo =1, Years =0	M31125	pMCI
178	Intensity [815851]	65832.031	42249.258	320276	OPTIMA =226, EPNo =3, Years =1.06	M31126	pMCI
179	Intensity [815990]	57875.699	41544.598	320278	OPTIMA =226, EPNo =7, Years =3.06	M31127	pMCI
180	Intensity [816071]	50078.574	8004.773	320279	OPTIMA =293, EPNo =1, Years =0	M31161	sCTL
181	Intensity [816199]	10526.518	6491.799	320281	OPTIMA =293, EPNo =3, Years =1.01	M31162	sCTL
182	Intensity [816259]	77633.312	16986.824	320282	OPTIMA =180, EPNo =1, Years =0	M31096	ODS
183	Intensity [816458]	25355.887	17915.539	320285	OPTIMA =215, EPNo =1, Years =0	M31109	pCTL.MCI
184	Intensity [816523]	72851.477	19421.277	320286	OPTIMA =180, EPNo =3, Years =1.29	M31097	ODS
185	Intensity [816584]	105063.156	19453.141	320287	OPTIMA =245, EPNo =1, Years =0	M31140	sCTL
186	Intensity [819288]	29822.393	22160.42	320309	OPTIMA =127, EPNo =9, Years =3.98	M31066	AD
187	Intensity [819409]	1243.15	1293.552	320311	OPTIMA =127, EPNo =1, Years =0	M31063	AD
188	Intensity [819541]	39526.438	21263.803	320313	OPTIMA =127, EPNo =7, Years =3.02	M31065	AD
189	Intensity [819698]	30217.207	27976.963	320315	OPTIMA =127, EPNo =3, Years =1.01	M31064	AD
190	Intensity [819837]	30630.457	20395.523	320317	OPTIMA =127, EPNo =11, Years =4.92	M31067	AD
191	Intensity [819915]	21301.363	22575.566	320318	OPTIMA =331, EPNo =3, Years =1.08	M31168	sCTL
192	Intensity [820092]	65932.789	26930.004	320321	OPTIMA =54, EPNo =1, Years =0	M31013	sCTL
193	Intensity [820153]	79450.461	25595.004	320322	OPTIMA =54, EPNo =5, Years =2.14	M31014	sCTL
194	Intensity [820215]	46751.113	24928.871	320323	OPTIMA =331, EPNo =1, Years =0	M31167	sCTL
195	Intensity [820273]	55031.402	22632.928	320324	OPTIMA =54, EPNo =9, Years =3.98	M31015	sCTL
196	Intensity [820346]	55633.195	16275.031	320325	OPTIMA =54, EPNo =11, Years =5.21	M31016	sCTL
197	Intensity [820472]	58999.645	26316.398	320327	OPTIMA =216, EPNo =3, Years =1.05	M31111	pCTL.MCI
198	Intensity [820986]	49596.254	21832.406	320334	OPTIMA =216, EPNo =1, Years =0	M31110	pCTL.MCI
199	Intensity [821120]	32022.223	17992.482	320336	OPTIMA =63, EPNo =3, Years =0.99	M31026	sCTL
200	Intensity [821248]	29731.086	16918.373	320338	OPTIMA =63, EPNo =1, Years =0	M31025	sCTL
201	Intensity [821372]	32727.059	16691.828	320340	OPTIMA =63, EPNo =9, Years =3.99	M31027	sCTL
202	Intensity [821498]	23906.898	10408.16	320342	OPTIMA =241, EPNo =5, Years =2.01	M31139	sCTL
203	Intensity [821732]	1110.512	694.65	320346	OPTIMA =241, EPNo =1, Years =0	M31137	pMCI
204	Intensity [821860]	15281.621	10931.395	320348	OPTIMA =241, EPNo =3, Years =1.02	M31138	sCTL
205	Intensity [821921]	64220.711	24873.02	320349	OPTIMA =173, EPNo =3, Years =0.99	M31091	AD
206	Intensity [822043]	68763.164	23242.98	320351	OPTIMA =173, EPNo =1, Years =0	M31090	AD
207	Intensity [822096]	29002.42	13421.753	320352	OPTIMA =281, EPNo =5, Years =2.06	M31159	sCTL
208	Intensity [822331]	15357.52	8724.931	320356	OPTIMA =281, EPNo =7, Years =3.03	M31160	sCTL
209	Intensity [822386]	48619.324	29047.186	320357	OPTIMA =225, EPNo =3, Years =1.01	M31124	pMCI
210	Intensity [822492]	27768.445	13513.383	320359	OPTIMA =281, EPNo =1, Years =0	M31158	sCTL

211	Intensity [822546]	55734.207	29975.949	320360	OPTIMA =225, EPNo =1, Years =0	M31123	pMCI
212	Intensity [822600]	65524.613	33499.121	320361	OPTIMA =216, EPNo =5, Years =2.07	M31112	pCTL.MCI
213	Intensity [823985]	51378.184	34915.781	320372	OPTIMA =219, EPNo =1, Years =0	M31113	pCTL.MCI
214	Intensity [824128]	48061.621	20088.816	320374	OPTIMA =219, EPNo =5, Years =1.91	M31115	pMCI
215	Intensity [824427]	71022.312	25134.117	320380	OPTIMA =220, EPNo =5, Years =2.03	M31118	pMCI
216	Intensity [824495]	74268.641	26788.688	320384	OPTIMA =220, EPNo =3, Years =1.07	M31117	pMCI
217	Intensity [824637]	62278.773	24056.48	320386	OPTIMA =220, EPNo =1, Years =0	M31116	pMCI
218	Intensity [824759]	63078.055	26375.031	320388	OPTIMA =220, EPNo =7, Years =2.97	M31119	pMCI
219	Intensity [824922]	23073.01	10991.855	320391	OPTIMA =257, EPNo =9, Years =4	M31153	sCTL
220	Intensity [825246]	30473.965	22096.83	320396	OPTIMA =257, EPNo =1, Years =0	M31151	sCTL
221	Intensity [825455]	25495.33	15256.975	320399	OPTIMA =48, EPNo =7, Years =3.2	M31010	sCTL
222	Intensity [825569]	30198.07	13842.228	320401	OPTIMA =48, EPNo =1, Years =0	M31008	sCTL
223	Intensity [825716]	21613.518	12146.823	320403	OPTIMA =48, EPNo =3, Years =1.21	M31009	sCTL
224	Intensity [825771]	17986.988	6286.771	320404	OPTIMA =279, EPNo =1, Years =0	M31156	sCTL
225	Intensity [825837]	51620.438	30008.328	320405	OPTIMA =246, EPNo =5, Years =1.93	M31144	sCTL
226	Intensity [826054]	35014.125	4882.391	320408	OPTIMA =279, EPNo =3, Years =0.93	M31157	sCTL
227	Intensity [826115]	61607.695	26958.891	320409	OPTIMA =246, EPNo =1, Years =0	M31142	sCTL
228	Intensity [826229]	54095.992	29749.973	320411	OPTIMA =246, EPNo =3, Years =0.95	M31143	sCTL
229	Intensity [826441]	46377.344	11727.834	320415	OPTIMA =443, EPNo =1, Years =0	M31169	sCTL
230	Intensity [826792]	47856.504	16802.568	320420	OPTIMA =443, EPNo =3, Years =1.04	M31170	sCTL
231	Intensity [827022]	68552.266	10784.427	320424	OPTIMA =212, EPNo =3, Years =0.94	M31105	pCTL.MCI
232	Intensity [827087]	169039.781	17037.479	320425	OPTIMA =212, EPNo =1, Years =0	M31104	pCTL.MCI
233	Intensity [828701]	17191.305	15513.914	320438	OPTIMA =16, EPNo =5, Years =2.01	M30996	AD
234	Intensity [828812]	30286.078	21084.52	320440	OPTIMA =16, EPNo =7, Years =2.98	M30997	AD
235	Intensity [828935]	14176.311	13140.918	320442	OPTIMA =16, EPNo =15, Years =6.96	M31001	AD
236	Intensity [829083]	11445.459	10770.268	320444	OPTIMA =16, EPNo =11, Years =5	M30999	AD
237	Intensity [829258]	16660.301	13867.939	320446	OPTIMA =16, EPNo =13, Years =5.97	M31000	AD
238	Intensity [829591]	20801.258	15104.018	320450	OPTIMA =16, EPNo =3, Years =0.99	M30995	AD
239	Intensity [830185]	15177.918	16884.572	320456	OPTIMA =16, EPNo =9, Years =3.98	M30998	AD
240	Intensity [831830]	42584.516	19192.258	320476	OPTIMA =57, EPNo =3, Years =1.08	M31018	sCTL
241	Intensity [832004]	33775.977	19807.176	320478	OPTIMA =57, EPNo =1, Years =0	M31017	sCTL
242	Intensity [835199]	18715.082	10797.506	320509	OPTIMA =105, EPNo =1, Years =0	M31048	AD
243	Intensity [835256]	28266.758	18037.297	320511	OPTIMA =59, EPNo =7, Years =3.24	M31022	sCTL
244	Intensity [835282]	68581.766	24997.633	320512	OPTIMA =105, EPNo =3, Years =1.16	M31049	AD
245	Intensity [835309]	30207.195	13607.68	320513	OPTIMA =59, EPNo =5, Years =2.2	M31021	sCTL
246	Intensity [835335]	64817.336	21743.473	320514	OPTIMA =105, EPNo =7, Years =3.12	M31051	AD
247	Intensity [835360]	49054.984	24664.648	320515	OPTIMA =59, EPNo =1, Years =0	M31019	sCTL
248	Intensity [835386]	34852.109	19709.602	320516	OPTIMA =105, EPNo =5, Years =2.13	M31050	AD
249	Intensity [835411]	19484.801	9668.724	320517	OPTIMA =59, EPNo =9, Years =4.19	M31023	sCTL
250	Intensity [835436]	16965.342	8415.641	320518	OPTIMA =59, EPNo =11, Years =5.19	M31024	sCTL
251	Intensity [835584]	43639.109	17039.482	320522	OPTIMA =59, EPNo =3, Years =1.1	M31020	sCTL
252	Intensity [836763]	23505.307	23963.562	320557	OPTIMA =215, EPNo =1, Years =0	M31109	pCTL.MCI
253	Intensity [839046]	17616.943	16663.828	320602	OPTIMA =331, EPNo =3, Years =1.08	M31168	sCTL
254	Intensity [839097]	34604.289	17504.709	320605	OPTIMA =54, EPNo =1, Years =0	M31013	sCTL
255	Intensity [839115]	44408.707	20096.844	320606	OPTIMA =54, EPNo =5, Years =2.14	M31014	sCTL
256	Intensity [839133]	24605.383	19716.721	320607	OPTIMA =331, EPNo =1, Years =0	M31167	sCTL
257	Intensity [839150]	32887.844	15382.223	320608	OPTIMA =54, EPNo =9, Years =3.98	M31015	sCTL
258	Intensity [839909]	32484.008	12402.23	320646	OPTIMA =54, EPNo =11, Years =5.21	M31016	sCTL
259	Intensity [841795]	18183.551	10810.754	320684	OPTIMA =48, EPNo =7, Years =3.2	M31010	sCTL
260	Intensity [841826]	46965.328	10149.358	320685	OPTIMA =212, EPNo =3, Years =0.94	M31105	pCTL.MCI
261	Intensity [841858]	21122.492	11331.919	320686	OPTIMA =48, EPNo =1, Years =0	M31008	sCTL
262	Intensity [841892]	102505.086	14057.739	320687	OPTIMA =212, EPNo =1, Years =0	M31104	pCTL.MCI
263	Intensity [841923]	15310.918	11192.458	320688	OPTIMA =48, EPNo =3, Years =1.21	M31009	sCTL
264	Intensity [841955]	9309.554	4623.473	320689	OPTIMA =279, EPNo =1, Years =0	M31156	sCTL
265	Intensity [841986]	42365.008	27436.066	320690	OPTIMA =246, EPNo =5, Years =1.93	M31144	sCTL
266	Intensity [842086]	25229.969	4609.71	320693	OPTIMA =279, EPNo =3, Years =0.93	M31157	sCTL
267	Intensity [842119]	50061.828	25825.834	320694	OPTIMA =246, EPNo =1, Years =0	M31142	sCTL
268	Intensity [842185]	43897.578	37110.867	320696	OPTIMA =246, EPNo =3, Years =0.95	M31143	sCTL
269	Intensity [842314]	17730.758	9033.494	320700	OPTIMA =443, EPNo =1, Years =0	M31169	sCTL
270	Intensity [842468]	35056.496	13307.49	320705	OPTIMA =443, EPNo =3, Years =1.04	M31170	sCTL
271	Intensity [825307]	25055.346	13514.977	320397	OPTIMA =257, EPNo =3, Years =1.15	M31152	sCTL
272	Intensity [826391]	15564.623	9871.209	320414	OPTIMA =255, EPNo =3, Years =1.2	M31149	sCTL
273	Intensity [826534]	30457.312	10662.9	320416	OPTIMA =255, EPNo =5, Years =2.2	M31150	sCTL
274	Intensity [826597]	19651.367	13312.865	320417	OPTIMA =237, EPNo =3, Years =1.08	M31136	pMCI
275	Intensity [826852]	21357.914	11280.356	320421	OPTIMA =255, EPNo =1, Years =0	M31148	sCTL
276	Intensity [826906]	48701.641	24642.047	320422	OPTIMA =237, EPNo =1, Years =0	M31135	pMCI
277	Intensity [826960]	64411.664	9950.537	320423	OPTIMA =219, EPNo =3, Years =1.01	M31114	pMCI
278	Intensity [827154]	90355.617	18682.408	320426	OPTIMA =245, EPNo =3, Years =1.13	M31141	sCTL
279	Intensity [830592]	99632.852	24303.668	320461	OPTIMA =150, EPNo =3, Years =1.5	M31077	AD
280	Intensity [830759]	85049.516	23119.797	320463	OPTIMA =150, EPNo =7, Years =3.32	M31079	AD
281	Intensity [830932]	102358.422	19259.59	320465	OPTIMA =150, EPNo =5, Years =2.37	M31078	AD

282	Intensity [831257]	80339.117	22261.203	320469	OPTIMA =150, EPNo =1, Years =0	M31076	AD
283	Intensity [831419]	22815.453	16738.031	320471	OPTIMA =159, EPNo =5, Years =2.05	M31088	AD
284	Intensity [831577]	20288.164	11329.955	320473	OPTIMA =159, EPNo =3, Years =1	M31087	AD
285	Intensity [831755]	28374.637	17758.699	320475	OPTIMA =159, EPNo =1, Years =0	M31086	AD
286	Intensity [831914]	28211.35	13753.094	320477	OPTIMA =159, EPNo =7, Years =2.99	M31089	AD
287	Intensity [832244]	29610.25	27539.16	320481	OPTIMA =214, EPNo =3, Years =0.97	M31107	pCTL.MCI
288	Intensity [832410]	40393.848	26252.969	320483	OPTIMA =214, EPNo =1, Years =0	M31106	pCTL.MCI
289	Intensity [832578]	22482.379	15864.934	320485	OPTIMA =214, EPNo =5, Years =2.1	M31108	pCTL.MCI
290	Intensity [832659]	47366.098	35675.445	320486	OPTIMA =137, EPNo =3, Years =0.93	M31069	AD
291	Intensity [832820]	34621.891	27474.941	320488	OPTIMA =137, EPNo =1, Years =0	M31068	AD
292	Intensity [832900]	35125.094	15319.671	320489	OPTIMA =222, EPNo =5, Years =1.97	M31122	pMCI
293	Intensity [833283]	45681.094	31923.934	320493	OPTIMA =222, EPNo =1, Years =0	M31120	pMCI
294	Intensity [833500]	30852.227	15328.71	320495	OPTIMA =222, EPNo =3, Years =0.96	M31121	pMCI
295	Intensity [833602]	5339.627	7285.993	320496	OPTIMA =300, EPNo =1, Years =0	M31163	sCTL
296	Intensity [833874]	8807.146	6195.71	320498	OPTIMA =300, EPNo =3, Years =1.03	M31164	sCTL
297	Intensity [835542]	15389.113	20133.906	320521	OPTIMA =82, EPNo =3, Years =1.03	M31033	sCTL
298	Intensity [835633]	27714.734	27839.611	320523	OPTIMA =82, EPNo =7, Years =3.07	M31035	sCTL
299	Intensity [835694]	22354.938	22362.23	320525	OPTIMA =82, EPNo =5, Years =1.96	M31034	sCTL
300	Intensity [835751]	23664.059	27887.199	320527	OPTIMA =82, EPNo =9, Years =4.01	M31036	sCTL
301	Intensity [835820]	17854.129	15290.269	320529	OPTIMA =207, EPNo =5, Years =2.09	M31102	pCTL.AD
302	Intensity [835855]	22111.656	16212.458	320530	OPTIMA =207, EPNo =1, Years =0	M31100	pCTL.AD
303	Intensity [835994]	26573.965	23319.305	320534	OPTIMA =207, EPNo =7, Years =3.1	M31103	pCTL.AD
304	Intensity [836054]	18985.836	16816.859	320536	OPTIMA =207, EPNo =3, Years =1.07	M31101	pCTL.AD
305	Intensity [836115]	59949.758	37160.883	320538	OPTIMA =145, EPNo =3, Years =1.14	M31071	AD
306	Intensity [836185]	45814.266	29447.27	320540	OPTIMA =145, EPNo =7, Years =3.09	M31072	AD
307	Intensity [836261]	42030.945	25375.852	320542	OPTIMA =145, EPNo =1, Years =0	M31070	AD
308	Intensity [836453]	66875.891	46196.906	320546	OPTIMA =226, EPNo =1, Years =0	M31125	pMCI
309	Intensity [836566]	74791.75	44601.508	320550	OPTIMA =226, EPNo =7, Years =3.06	M31127	pMCI
310	Intensity [836589]	51865.547	12283.709	320551	OPTIMA =293, EPNo =1, Years =0	M31161	sCTL
311	Intensity [836636]	6922.921	6256.759	320553	OPTIMA =293, EPNo =3, Years =1.01	M31162	sCTL
312	Intensity [836658]	75043.047	19616.281	320554	OPTIMA =180, EPNo =1, Years =0	M31096	ODS
313	Intensity [836797]	76994.523	22763.586	320558	OPTIMA =180, EPNo =3, Years =1.29	M31097	ODS
314	Intensity [836842]	89418.109	18414.684	320559	OPTIMA =245, EPNo =1, Years =0	M31140	sCTL
315	Intensity [836866]	1876.058	233.031	320560	OPTIMA =226, EPNo =3, Years =1.06	M31126	pMCI
316	Intensity [838937]	1602.855	1328.321	320595	OPTIMA =127, EPNo =1, Years =0	M31063	AD
317	Intensity [838974]	23793.859	16770.666	320597	OPTIMA =127, EPNo =7, Years =3.02	M31065	AD
318	Intensity [838993]	22631.133	32763.295	320599	OPTIMA =127, EPNo =3, Years =1.01	M31064	AD
319	Intensity [839029]	20357.102	14585.187	320601	OPTIMA =127, EPNo =11, Years =4.92	M31067	AD
320	Intensity [839202]	35109.238	25330.07	320611	OPTIMA =216, EPNo =3, Years =1.05	M31111	pCTL.MCI
321	Intensity [839239]	40038.758	25861.359	320613	OPTIMA =216, EPNo =5, Years =2.07	M31112	pCTL.MCI
322	Intensity [839325]	31394.199	16078.314	320618	OPTIMA =216, EPNo =1, Years =0	M31110	pCTL.MCI
323	Intensity [839361]	17546.047	14257.319	320620	OPTIMA =63, EPNo =3, Years =0.99	M31026	sCTL
324	Intensity [839396]	19063.621	10022.699	320622	OPTIMA =63, EPNo =1, Years =0	M31025	sCTL
325	Intensity [839431]	26851.676	14275.388	320624	OPTIMA =63, EPNo =9, Years =3.99	M31027	sCTL
326	Intensity [839468]	21699.035	9717.917	320626	OPTIMA =241, EPNo =5, Years =2.01	M31139	sCTL
327	Intensity [839538]	1061.244	615.347	320630	OPTIMA =241, EPNo =1, Years =0	M31137	pMCI
328	Intensity [839594]	11072.477	9119.02	320632	OPTIMA =241, EPNo =3, Years =1.02	M31138	sCTL
329	Intensity [839620]	41395.57	20954.197	320633	OPTIMA =173, EPNo =3, Years =0.99	M31091	AD
330	Intensity [839661]	58007.855	17878.891	320635	OPTIMA =173, EPNo =1, Years =0	M31090	AD
331	Intensity [839684]	18248.332	9538.059	320636	OPTIMA =281, EPNo =5, Years =2.06	M31159	sCTL
332	Intensity [839791]	10889.596	7095.629	320640	OPTIMA =281, EPNo =7, Years =3.03	M31160	sCTL
333	Intensity [839810]	30865.465	24474.539	320641	OPTIMA =225, EPNo =3, Years =1.01	M31124	pMCI
334	Intensity [839849]	19208.145	8645.852	320643	OPTIMA =281, EPNo =1, Years =0	M31158	sCTL
335	Intensity [839870]	34556.148	19421.6	320644	OPTIMA =225, EPNo =1, Years =0	M31123	pMCI
336	Intensity [839890]	23955.453	16059.555	320645	OPTIMA =127, EPNo =9, Years =3.98	M31066	AD
337	Intensity [840361]	39277.859	24161.391	320657	OPTIMA =219, EPNo =1, Years =0	M31113	pCTL.MCI
338	Intensity [840417]	28211.156	16920.057	320659	OPTIMA =219, EPNo =5, Years =1.91	M31115	pMCI
339	Intensity [840537]	51060.316	7453.638	320662	OPTIMA =219, EPNo =3, Years =1.01	M31114	pMCI
340	Intensity [840838]	43825.512	18543.072	320665	OPTIMA =220, EPNo =5, Years =2.03	M31118	pMCI
341	Intensity [841007]	44617.793	18559.492	320669	OPTIMA =220, EPNo =3, Years =1.07	M31117	pMCI
342	Intensity [841097]	40558.996	17090.254	320671	OPTIMA =220, EPNo =1, Years =0	M31116	pMCI
343	Intensity [841195]	41352.68	18970.273	320673	OPTIMA =220, EPNo =7, Years =2.97	M31119	pMCI
344	Intensity [841346]	15109.942	10146.261	320676	OPTIMA =257, EPNo =9, Years =4	M31153	sCTL
345	Intensity [841694]	23896.834	15104.284	320681	OPTIMA =257, EPNo =1, Years =0	M31151	sCTL
346	Intensity [841729]	20583.258	9937.393	320682	OPTIMA =257, EPNo =5, Years =1.15	M31152	sCTL
347	Intensity [842249]	66383.172	25034.145	320698	OPTIMA =245, EPNo =3, Years =1.13	M31141	sCTL
348	Intensity [842281]	9383.795	9799.741	320699	OPTIMA =255, EPNo =3, Years =1.2	M31149	sCTL
349	Intensity [842343]	24148.301	10803.502	320701	OPTIMA =255, EPNo =5, Years =2.2	M31150	sCTL
350	Intensity [842374]	15967.262	12929.273	320702	OPTIMA =237, EPNo =3, Years =1.08	M31136	pMCI
351	Intensity [842498]	7920.779	9119.039	320706	OPTIMA =255, EPNo =1, Years =0	M31148	sCTL
352	Intensity [842528]	37405.305	24399.461	320707	OPTIMA =237, EPNo =1, Years =0	M31135	pMCI
