# Supplementary material for: High Resolution Discovery Proteomics Reveals Candidate Disease Progression Markers of Alzheimer’s Disease in Human Cerebrospinal Fluid
Source: PLoS One. 2015 Aug 13;10(8):e0135365. doi: 10.1371/journal.pone.0135365 (PMC4535975; doi:10.1371/journal.pone.0135365)
Supplement: S5 Table — (PDF) [file pone.0135365.s008.pdf]

S5 Table   Cohort-2 linear mixed-effect model analysis of 8108 features

FeatureID	Peak.Centr	Peak.Centr	pctChange	pAD	pADCTL	Protein Description	Peptide Sequence
751077575	637.326	32.167	2.7	0.387	0.601	Tax_Id=9606 Gene_Symbol=PTGDS Prostaglandin-H2 D-isomerase	AQGFTEDTIVFLPQTDK
751077576	637.661	32.182	2.5	0.428	0.585	Tax_Id=9606 Gene_Symbol=PTGDS Prostaglandin-H2 D-isomerase	AQGFTEDTIVFLPQTDK
751077577	637.995	32.204	2.5	0.443	0.603	Tax_Id=9606 Gene_Symbol=PTGDS Prostaglandin-H2 D-isomerase	AQGFTEDTIVFLPQTDK
751077578	638.329	32.217	2.7	0.368	0.656	Tax_Id=9606 Gene_Symbol=PTGDS Prostaglandin-H2 D-isomerase	AQGFTEDTIVFLPQTDK
751077579	638.663	32.251	2.7	0.355	0.624	Tax_Id=9606 Gene_Symbol=PTGDS Prostaglandin-H2 D-isomerase	AQGFTEDTIVFLPQTDK
751077580	638.998	32.262	2.5	0.353	0.499	Tax_Id=9606 Gene_Symbol=PTGDS Prostaglandin-H2 D-isomerase	AQGFTEDTIVFLPQTDK
751077581	639.331	32.186	2.1	0.415	0.628	Tax_Id=9606 Gene_Symbol=PTGDS Prostaglandin-H2 D-isomerase	AQGFTEDTIVFLPQTDK
751077582	955.489	32.135	3.3	0.452	0.773	Tax_Id=9606 Gene_Symbol=PTGDS Prostaglandin-H2 D-isomerase	AQGFTEDTIVFLPQTDK
751077583	955.99	32.168	3.4	0.454	0.769	Tax_Id=9606 Gene_Symbol=PTGDS Prostaglandin-H2 D-isomerase	AQGFTEDTIVFLPQTDK
751077584	956.492	32.211	3.1	0.462	0.763	Tax_Id=9606 Gene_Symbol=PTGDS Prostaglandin-H2 D-isomerase	AQGFTEDTIVFLPQTDK
751077585	956.993	32.236	3	0.449	0.802	Tax_Id=9606 Gene_Symbol=PTGDS Prostaglandin-H2 D-isomerase	AQGFTEDTIVFLPQTDK
751077586	957.495	32.26	3.3	0.362	0.791	Tax_Id=9606 Gene_Symbol=PTGDS Prostaglandin-H2 D-isomerase	AQGFTEDTIVFLPQTDK
751077587	957.996	32.298	2.7	0.341	0.507	Tax_Id=9606 Gene_Symbol=PTGDS Prostaglandin-H2 D-isomerase	AQGFTEDTIVFLPQTDK
751077588	958.498	32.211	1.3	0.673	0.652	Tax_Id=9606 Gene_Symbol=PTGDS Prostaglandin-H2 D-isomerase	AQGFTEDTIVFLPQTDK
751077589	595.966	18.837	2.3	0.656	0.439		
751077590	596.3	18.878	2.1	0.675	0.449		
751077591	596.635	18.92	1.5	0.747	0.425		
751077592	596.969	18.941	1.3	0.752	0.435		
751077593	597.304	18.938	1.2	0.792	0.493		
751077594	597.638	18.954	1.9	0.667	0.582		
751077595	597.972	18.774	2.9	0.612	0.507		
751077596	391.286	66.095	0.8	0.931	0.395		
751077597	392.289	66.097	0.9	0.914	0.395		
751077598	393.293	66.086	1	0.899	0.389		
751077599	394.297	66.043	5.4	0.552	0.178		
751077600	712.862	28.741	5.5	0.253	0.233		
751077601	713.364	28.747	5.5	0.237	0.231		
751077602	713.865	28.744	5.5	0.26	0.248		
751077603	714.363	28.691	2.6	0.548	0.444		
751077604	714.867	28.735	6.4	0.206	0.257		
751077605	715.364	28.694	3.8	0.178	0.313		
751077606	893.448	18.826	3.3	0.606	0.552		
751077607	893.949	18.869	2.3	0.699	0.468		
751077608	894.45	18.904	1.3	0.722	0.188		
751077609	894.951	18.918	1.4	0.7	0.251		
751077610	895.452	18.926	1	0.778	0.263		
751077611	895.954	18.908	-0.2	0.963	0.236		
751077612	896.451	18.703	-2.4	0.873	0.671		
751077613	965.109	51.003	2.8	0.602	0.302		
751077614	965.262	51.005	3.1	0.564	0.363		
751077615	965.414	51.002	2.6	0.616	0.351		
751077616	965.57	50.999	3.2	0.541	0.354		
751077617	965.724	51.003	4.1	0.442	0.391		
751077618	965.875	51.02	1.3	0.822	0.271		
751077619	965.952	50.994	6.4	0.207	0.471		
751077620	966.028	51.046	7.4	0.528	0.057		
751077621	964.874	51.006	3	0.583	0.33		
751077622	965.031	50.999	2.7	0.623	0.306		
751077623	965.187	51.002	3.3	0.534	0.347		
751077624	965.336	51.003	3	0.585	0.395		
751077625	965.492	51.004	2.8	0.592	0.356		
751077626	965.648	51.006	3	0.567	0.337		
751077627	965.8	51.007	3	0.577	0.331		
751077628	896.243	51.005	3.4	0.527	0.359		
751077629	896.387	51.007	3.4	0.515	0.41		
751077630	896.527	51.007	3.1	0.569	0.398		
751077631	896.671	51.011	4.1	0.431	0.4		
751077632	896.813	51.019	4	0.46	0.398		
751077633	896.954	51.05	21.7	0.042	0.506		
751077634	809.401	17.139	11.2	0.223	0.925		
751077635	809.902	17.164	9.5	0.27	0.994		
751077636	810.402	17.182	7.4	0.345	0.901		
751077637	810.903	17.192	7.1	0.354	0.902		
751077638	811.405	17.195	5.8	0.527	0.778		
751077639	811.907	17.201	2.8	0.916	0.351		
751077640	799.406	19.352	0.6	0.921	0.852		
751077641	799.908	19.373	0.4	0.941	0.839		
751077642	800.409	19.358	0.6	0.921	0.864		
751077643	800.911	19.358	0.3	0.955	0.839		
751077644	801.412	19.365	0.6	0.926	0.875		
751077645	801.913	19.412	-0.6	0.922	0.634		
751077646	544.297	24.377	3.1	0.663	0.371	Tax_Id=9606 Gene_Symbol=HBA2;HBA1 Hemoglobin alpha-2	M[147.0355]FLSFPTTK
751077647	544.798	24.376	2.8	0.685	0.341	Tax_Id=9606 Gene_Symbol=HBA2;HBA1 Hemoglobin alpha-2	M[147.0355]FLSFPTTK
751077648	545.3	24.376	2.9	0.685	0.351	Tax_Id=9606 Gene_Symbol=HBA2;HBA1 Hemoglobin alpha-2	M[147.0355]FLSFPTTK
751077649	545.8	24.372	4.7	0.791	0.228	Tax_Id=9606 Gene_Symbol=HBA2;HBA1 Hemoglobin alpha-2	M[147.0355]FLSFPTTK
751077650	546.299	24.317	0.9	0.952	0.057	Tax_Id=9606 Gene_Symbol=HBA2;HBA1 Hemoglobin alpha-2	M[147.0355]FLSFPTTK
751077651	644.964	24.017	3.2	0.627	0.919		
751077652	645.298	24.047	3.1	0.636	0.932		
751077653	645.632	24.068	2.9	0.656	0.904		
751077654	645.966	24.116	2.3	0.708	0.987		
751077655	646.299	24.042	5.1	0.456	0.657		
751077656	646.634	24.057	3.8	0.482	0.538		
751077657	896.314	51.005	3.6	0.481	0.357		
751077658	896.458	51.004	3.7	0.483	0.438		
751077659	896.598	51.011	3.4	0.517	0.39		
751077660	896.741	51.015	4.9	0.361	0.482		
751077661	896.885	51.031	5.3	0.386	0.534		
751077662	897.024	51.049	175.1	0.003	0.109		
751077663	794.396	33.059	4.9	0.429	0.357		
751077664	794.897	33.065	5.1	0.429	0.373		
751077665	795.398	33.07	4.8	0.439	0.332		
751077666	795.899	33.062	5.2	0.345	0.235		

751077667	796.399	33.037	6.7	0.474	0.657		
751077668	796.901	33.073	4.2	0.76	0.419		
751077669	510.736	14.891	4.3	0.453	0.433		
751077670	511.239	14.895	4.5	0.441	0.522		
751077671	511.739	14.894	6.8	0.559	0.404		
751077672	512.241	14.896	-32.3	0.318	0.176		
751077673	512.746	14.853	5.8	0.74	0.302		
751077674	803.549	66.14	0	1	0.492		
751077675	804.553	66.138	0.1	0.993	0.488		
751077676	805.556	66.137	0.1	0.986	0.468		
751077677	806.56	66.137	-0.1	0.994	0.483		
751077678	813.717	52.252	8.8	0.023	0.99		
751077679	813.894	52.146	8.8	0.024	0.871		
751077680	814.07	52.258	8.4	0.034	0.898		
751077681	836.558	51	3.4	0.509	0.392		
751077682	836.691	51.001	3.8	0.475	0.424		
751077683	836.824	51	4.2	0.406	0.401		
751077684	836.958	51.002	3.6	0.48	0.409		
751077685	837.092	51.005	3.5	0.532	0.5		
751077686	837.225	51.014	4.1	0.464	0.429		
751077687	837.358	50.986	11	0.152	0.769		
751077688	836.357	51.003	2.7	0.623	0.325		
751077689	836.491	50.997	4	0.463	0.495		
751077690	836.626	50.999	4.1	0.447	0.502		
751077691	836.757	51.001	3	0.564	0.359		
751077692	836.891	51.001	3.9	0.458	0.417		
751077693	837.025	51.001	3.2	0.551	0.44		
751077694	837.159	51.009	3.9	0.479	0.362		
751077695	837.292	51.018	17	0.067	0.949		
751077696	539.935	17.127	9.3	0.216	0.956		
751077697	540.27	17.179	9.5	0.203	0.986		
751077698	540.604	17.155	8	0.246	0.944		
751077699	540.938	17.162	7	0.329	0.836		
751077700	541.272	17.115	14.6	0.333	0.791		
751077701	822.904	21.432	-0.9	0.87	0.192	Tax_Id=9606 Gene_Symbol=CST3 Cystatin-C	LVGGPMDASVEEEGVR
751077702	823.405	21.432	-1.1	0.844	0.171	Tax_Id=9606 Gene_Symbol=CST3 Cystatin-C	LVGGPMDASVEEEGVR
751077703	823.906	21.432	-1	0.849	0.165	Tax_Id=9606 Gene_Symbol=CST3 Cystatin-C	LVGGPMDASVEEEGVR
751077704	824.407	21.428	-0.5	0.921	0.191	Tax_Id=9606 Gene_Symbol=CST3 Cystatin-C	LVGGPMDASVEEEGVR
751077705	824.908	21.428	-1.9	0.699	0.051	Tax_Id=9606 Gene_Symbol=CST3 Cystatin-C	LVGGPMDASVEEEGVR
751077706	825.409	21.435	-20	0.242	0.556	Tax_Id=9606 Gene_Symbol=CST3 Cystatin-C	LVGGPMDASVEEEGVR
751077707	720.86	25.642	0.1	0.958	0.416		
751077708	721.362	25.677	0.2	0.944	0.4		
751077709	721.863	25.648	0.2	0.929	0.426		
751077710	722.364	25.661	-0.1	0.98	0.331		
751077711	722.868	25.683	-0.6	0.831	0.333		
751077712	559.31	20.9	6.6	0.227	0.998	Tax_Id=9606 Gene_Symbol=CLU Isoform 2 of Clusterin	TLLSNLEEAK
751077713	559.812	20.893	6.9	0.215	0.968	Tax_Id=9606 Gene_Symbol=CLU Isoform 2 of Clusterin	TLLSNLEEAK
751077714	560.313	20.897	6.9	0.221	0.942	Tax_Id=9606 Gene_Symbol=CLU Isoform 2 of Clusterin	TLLSNLEEAK
751077715	560.814	20.889	7.5	0.186	0.948	Tax_Id=9606 Gene_Symbol=CLU Isoform 2 of Clusterin	TLLSNLEEAK
751077716	561.314	20.887	-14.8	0.397	0.294	Tax_Id=9606 Gene_Symbol=CLU Isoform 2 of Clusterin	TLLSNLEEAK
751077717	413.268	66.173	NA	NA	NA		
751077718	414.271	66.172	1.9	0.81	0.378		
751077719	415.275	66.152	1.7	0.81	0.422		
751077720	719.364	28.599	-2.1	0.712	0.599	Tax_Id=9606 Gene_Symbol=AGT Angiotensinogen	SLDFTELDVAAEK
751077721	719.866	28.59	-1.2	0.81	0.585	Tax_Id=9606 Gene_Symbol=AGT Angiotensinogen	SLDFTELDVAAEK
751077722	720.366	28.568	-0.7	0.898	0.551	Tax_Id=9606 Gene_Symbol=AGT Angiotensinogen	SLDFTELDVAAEK
751077723	700.847	20.231	4.8	0.496	0.811		
751077724	701.349	20.263	4.1	0.536	0.755		
751077725	701.85	20.288	2.4	0.686	0.65		
751077726	702.35	20.245	1.8	0.753	0.631		
751077727	702.854	20.308	1.2	0.831	0.315		
751077728	464.234	16.476	2.8	0.737	0.828		
751077729	464.569	16.478	3	0.684	0.904		
751077730	464.903	16.479	2.5	0.765	0.844		
751077731	465.237	16.481	0.5	0.941	0.399		
751077732	465.572	16.487	-24.5	0.073	0.035		
751077733	573.28	18.518	2.9	0.547	0.275		
751077734	573.781	18.517	2.7	0.565	0.26		
751077735	574.284	18.512	2.9	0.56	0.287		
751077736	574.784	18.51	3.4	0.59	0.526		
751077737	768.567	52.255	9.1	0.027	0.842		
751077738	768.787	52.259	9.2	0.021	0.813		
751077739	813.66	52.151	7	0.097	0.961		
751077740	813.836	52.263	8.1	0.038	0.994		
751077741	814.011	52.181	7.9	0.041	0.896		
751077742	814.128	52.285	8.6	0.032	0.887		
751077743	578.306	19.675	1.1	0.82	0.623		
751077744	578.808	19.691	1.1	0.83	0.601		
751077745	579.309	19.708	1.3	0.804	0.661		
751077746	579.81	19.726	-0.1	0.977	0.528		
751077747	711.603	34.463	8.4	0.368	0.643	Tax_Id=9606 Gene_Symbol=GSN Isoform 1 of Gelsolin	NWRDPDQTDGLGLSYLSSHIANVER
751077748	711.854	34.463	8.2	0.354	0.625	Tax_Id=9606 Gene_Symbol=GSN Isoform 1 of Gelsolin	NWRDPDQTDGLGLSYLSSHIANVER
751077749	712.105	34.463	8.9	0.341	0.689	Tax_Id=9606 Gene_Symbol=GSN Isoform 1 of Gelsolin	NWRDPDQTDGLGLSYLSSHIANVER
751077750	712.355	34.467	9.2	0.368	0.688	Tax_Id=9606 Gene_Symbol=GSN Isoform 1 of Gelsolin	NWRDPDQTDGLGLSYLSSHIANVER
751077751	712.606	34.459	6.7	0.422	0.516	Tax_Id=9606 Gene_Symbol=GSN Isoform 1 of Gelsolin	NWRDPDQTDGLGLSYLSSHIANVER
751077752	712.857	34.454	5.4	0.57	0.201	Tax_Id=9606 Gene_Symbol=GSN Isoform 1 of Gelsolin	NWRDPDQTDGLGLSYLSSHIANVER
751077753	713.108	34.455	1.6	0.88	0.134	Tax_Id=9606 Gene_Symbol=GSN Isoform 1 of Gelsolin	NWRDPDQTDGLGLSYLSSHIANVER
751077754	719.705	34.832	6.9	0.25	0.58		
751077755	720.04	34.833	6.6	0.276	0.573		
751077756	720.374	34.939	6.2	0.286	0.648		
751077757	720.708	34.854	5.6	0.34	0.589		
751077758	721.042	34.858	5.2	0.324	0.488		
751077759	721.375	35.05	6.8	0.268	0.747		

751077760	585.305	16.388	0.4	0.933	0.232		
751077761	585.64	16.388	0.3	0.957	0.22		
751077762	585.974	16.391	0.5	0.918	0.227		
751077763	586.309	16.39	-0.9	0.862	0.157		
751077764	586.643	16.39	-22.2	0.086	0.03		
751077765	428.251	16.924	5.4	0.477	0.826		
751077766	428.753	16.923	5.3	0.492	0.837		
751077767	429.255	16.923	5.5	0.51	0.885		
751077768	429.756	16.93	-7.2	0.746	0.508		
751077769	689.698	49.972	15.1	0.111	0.861		
751077770	966.339	50.669	1	0.845	0.356		
751077771	966.494	50.761	1.3	0.798	0.356		
751077772	966.647	50.745	0.8	0.861	0.316		
751077773	966.802	50.76	0.7	0.879	0.293		
751077774	966.955	50.798	0.1	0.986	0.237		
751077775	1045.619	50.993	3	0.583	0.38		
751077776	1045.868	51.116	5.9	0.248	0.861		
751077777	671.36	31.357	3.6	0.394	0.824		
751077778	671.862	31.356	3.4	0.423	0.816		
751077779	672.364	31.356	3.5	0.424	0.805		
751077780	672.864	31.361	3.3	0.484	0.79		
751077781	966.418	50.757	0.6	0.908	0.347		
751077782	966.57	50.748	0.5	0.92	0.287		
751077783	966.724	50.746	-0.2	0.964	0.134		
751077784	966.879	50.78	0.5	0.927	0.304		
751077785	967.03	50.82	-0.5	0.918	0.175		
751077786	897.388	50.711	1	0.839	0.32		
751077787	897.53	50.722	0.9	0.85	0.357		
751077788	897.67	50.729	0	0.993	0.311		
751077789	897.813	50.708	-0.4	0.931	0.198		
751077790	897.958	50.686	0.5	0.916	0.237		
751077791	775.409	49.969	14.3	0.12	0.986		
751077792	775.534	49.971	13.5	0.143	0.997		
751077793	775.66	49.971	15	0.11	0.878		
751077794	775.785	49.971	14.9	0.108	0.906		
751077795	775.91	49.971	13.9	0.136	0.956		
751077796	776.035	49.971	13.8	0.133	0.981		
751077797	776.16	49.97	14.7	0.121	0.963		
751077798	776.285	49.97	12.3	0.155	0.901		
751077799	776.411	49.967	7.4	0.534	0.359		
751077800	776.536	49.969	11.8	0.136	0.626		
751077801	776.66	49.961	16.1	0.035	0.7		
751077802	689.586	49.972	17.6	0.079	0.688		
751077803	689.809	49.972	16.2	0.088	0.815		
751077804	690.031	49.974	16.1	0.076	0.819		
751077805	629.832	16.69	1.5	0.843	0.528		
751077806	630.334	16.69	1.4	0.848	0.537		
751077807	630.835	16.684	1.1	0.883	0.533		
751077808	631.337	16.689	0.6	0.969	0.412		
751077809	631.84	16.696	-30.7	0.025	0.005		
751077810	1056.17	37.384	6.7	0.389	0.758		
751077811	1056.504	37.416	6	0.437	0.697		
751077812	1056.837	37.453	6	0.441	0.681		
751077813	1057.171	37.492	5.6	0.463	0.647		
751077814	1057.504	37.534	5.6	0.493	0.633		
751077815	1057.838	37.555	5.6	0.496	0.609		
751077816	1058.171	37.565	6.2	0.349	0.702		
751077817	1058.507	37.537	6.4	0.205	0.853		
751077818	537.776	14.997	3.6	0.616	0.488	Tax_Id=9606 Gene_Symbol=CLU Isoform 2 of Clusterin	IDSLLENDR
751077819	538.278	14.999	3.6	0.621	0.482	Tax_Id=9606 Gene_Symbol=CLU Isoform 2 of Clusterin	IDSLLENDR
751077820	538.779	14.996	6	0.778	0.385	Tax_Id=9606 Gene_Symbol=CLU Isoform 2 of Clusterin	IDSLLENDR
751077821	539.281	14.983	4.4	0.758	0.342	Tax_Id=9606 Gene_Symbol=CLU Isoform 2 of Clusterin	IDSLLENDR
751077822	897.315	50.704	0.7	0.889	0.324		
751077823	897.46	50.731	0.9	0.861	0.346		
751077824	897.6	50.725	1	0.837	0.302		
751077825	897.743	50.711	1	0.847	0.384		
751077826	897.886	50.69	-0.2	0.966	0.279		
751077827	1193.831	66.101	1.9	0.83	0.436		
751077828	1194.835	66.101	1.8	0.835	0.444		
751077829	1195.838	66.101	2.1	0.802	0.42		
751077830	1196.841	66.101	1.5	0.846	0.454		
751077831	1197.844	66.106	-0.6	0.927	0.469		
751077832	806.119	31.06	20.6	0.185	0.581		
751077833	806.377	31.065	18.1	0.229	0.627		
751077834	806.624	31.069	15.1	0.32	0.745		
751077835	806.874	31.076	11.9	0.412	0.817		
751077836	807.122	31.08	12.7	0.351	0.785		
751077837	807.377	31.081	11	0.406	0.732		
751077838	807.626	31.092	3.2	0.839	0.23		
751077839	807.878	31.083	-0.2	0.989	0.377		
751077840	510.6	22.847	2.5	0.66	0.705		
751077841	510.935	22.846	2.7	0.643	0.735		
751077842	511.269	22.845	2.2	0.714	0.653		
751077843	511.603	22.844	2.2	0.696	0.7		
751077844	511.934	22.951	1.8	0.832	0.28		
751077845	779.772	51.656	-2.7	0.582	0.098		
751077846	780.107	51.659	-2.2	0.61	0.085		
751077847	780.441	51.664	-4.1	0.333	0.031		
751077848	780.774	51.661	-1.4	0.717	0.098		
751077849	781.109	51.642	0.2	0.951	0.245		
751077850	762.154	49.491	0.7	0.906	0.167	Tax_Id=9606 Gene_Symbol=B3GNT1 N-acetyllactosaminide beta-1,3-N-acet	TTMDPNDVILATHASVDNLLHLSGLLER
751077851	762.405	49.49	0.4	0.94	0.151	Tax_Id=9606 Gene_Symbol=B3GNT1 N-acetyllactosaminide beta-1,3-N-acet	TTMDPNDVILATHASVDNLLHLSGLLER
751077852	762.655	49.492	0.5	0.928	0.159	Tax_Id=9606 Gene_Symbol=B3GNT1 N-acetyllactosaminide beta-1,3-N-acet	TTMDPNDVILATHASVDNLLHLSGLLER

751077853	762.906	49.491	0.7	0.9	0.185	Tax_Id=9606 Gene_Symbol=B3GNT1 N-acetyllactosaminide beta-1,3-N-acet	TTMDPNDVILATHASVDNLLHLSGLLER
751077854	763.156	49.491	0.7	0.9	0.158	Tax_Id=9606 Gene_Symbol=B3GNT1 N-acetyllactosaminide beta-1,3-N-acet	TTMDPNDVILATHASVDNLLHLSGLLER
751077855	763.407	49.488	4.3	0.778	0.376	Tax_Id=9606 Gene_Symbol=B3GNT1 N-acetyllactosaminide beta-1,3-N-acet	TTMDPNDVILATHASVDNLLHLSGLLER
751077856	763.658	49.483	2.4	0.848	0.294	Tax_Id=9606 Gene_Symbol=B3GNT1 N-acetyllactosaminide beta-1,3-N-acet	TTMDPNDVILATHASVDNLLHLSGLLER
751077857	787.88	24.91	-3.6	0.459	0.118		
751077858	788.382	24.908	-3.4	0.491	0.131		
751077859	788.884	24.906	-3.5	0.46	0.108		
751077860	789.385	24.914	-1.8	0.74	0.304		
751077861	789.886	24.926	-3	0.598	0.283		
751077862	768.622	52.238	8.9	0.028	0.933		
751077863	768.732	52.256	7.2	0.093	0.888		
751077864	768.844	52.179	9.4	0.021	0.951		
751077865	768.512	52.177	7.2	0.1	0.934		
751077866	741.373	21.81	7.2	0.203	0.958		
751077867	741.874	21.808	7.3	0.199	0.95		
751077868	742.375	21.807	7.6	0.177	0.941		
751077869	742.877	21.813	7.1	0.187	0.979		
751077870	743.376	21.815	8.8	0.157	0.853		
751077871	802.394	30.134	0	0.999	0.463		
751077872	802.895	30.151	0	0.989	0.431		
751077873	803.396	30.167	-0.1	0.96	0.43		
751077874	803.897	30.185	-0.3	0.899	0.422		
751077875	804.398	30.138	0	0.997	0.587		
751077876	804.898	30.131	18	0.323	0.352		
751077877	596.817	22.529	10	0.105	0.404		
751077878	597.318	22.533	10	0.105	0.409		
751077879	597.819	22.532	10.3	0.1	0.395		
751077880	598.32	22.533	9.9	0.189	0.664		
751077881	662.338	27.427	6.9	0.299	0.473		
751077882	662.839	27.421	7.5	0.297	0.513		
751077883	663.341	27.436	7	0.268	0.624		
751077884	663.843	27.483	8.8	0.259	0.883		
751077885	664.341	27.572	6.8	0.455	0.557		
751077886	812.646	38.41	3.8	0.708	0.597		
751077887	812.896	38.408	3	0.767	0.568		
751077888	813.147	38.408	4.3	0.672	0.616		
751077889	813.397	38.409	3.6	0.729	0.616		
751077890	813.648	38.408	4.2	0.684	0.605		
751077891	813.898	38.407	3.5	0.742	0.515		
751077892	814.149	38.406	3.5	0.631	0.465		
751077893	814.4	38.41	8.6	0.27	0.508		
751077894	964.953	51.003	3.3	0.543	0.381		
751077895	681.537	36.367	5.5	0.509	0.41		
751077896	681.738	36.363	5.6	0.517	0.418		
751077897	681.938	36.365	5.2	0.536	0.388		
751077898	682.138	36.364	4.8	0.562	0.37		
751077899	682.338	36.357	4.4	0.527	0.42		
751077900	682.539	36.364	6.5	0.356	0.512		
751077901	682.74	36.368	12.2	0.302	0.468		
751077902	682.937	36.378	-0.5	0.947	0.237		
751077903	460.287	28.223	8.8	0.299	0.658		
751077904	460.621	28.221	9.2	0.286	0.643		
751077905	460.956	28.215	9	0.305	0.626		
751077906	461.29	28.193	8.1	0.374	0.686		
751077907	461.624	28.178	6.9	0.895	0.534		
751077908	595.808	18.33	4.3	0.492	0.827		
751077909	596.811	18.315	4.1	0.509	0.871		
751077910	571.302	18.948	6	0.216	0.944		
751077911	571.804	18.948	6.5	0.182	0.962		
751077912	572.303	18.961	6.2	0.206	0.939		
751077913	572.806	18.992	8.1	0.548	0.689		
751077914	442.185	15.35	5.7	0.44	0.278		
751077915	442.519	15.351	7.8	0.579	0.347		
751077916	442.853	15.351	10.4	0.358	0.253		
751077917	443.185	15.351	12	0.488	0.202		
751077918	443.519	15.359	10.3	0.279	0.403		
751077919	613.809	22.553	0.8	0.871	0.478	Tax_Id=9606 Gene_Symbol=CST3 Cystatin-C	ALDFAVGEYNK
751077920	614.311	22.566	0.7	0.892	0.448	Tax_Id=9606 Gene_Symbol=CST3 Cystatin-C	ALDFAVGEYNK
751077921	614.814	22.621	0.8	0.871	0.503	Tax_Id=9606 Gene_Symbol=CST3 Cystatin-C	ALDFAVGEYNK
751077922	615.314	22.529	-0.4	0.966	0.868	Tax_Id=9606 Gene_Symbol=CST3 Cystatin-C	ALDFAVGEYNK
751077923	615.82	22.503	4.3	0.738	0.964	Tax_Id=9606 Gene_Symbol=CST3 Cystatin-C	ALDFAVGEYNK
751077924	728.062	52.184	8.5	0.037	0.94		
751077925	728.22	52.179	8.6	0.038	0.776		
751077926	728.377	52.261	9.3	0.02	0.763		
751077927	728.536	52.268	9.9	0.018	0.874		
751077928	729.371	25.07	7.3	0.262	0.408		
751077929	729.705	25.046	6.7	0.301	0.445		
751077930	730.039	25.05	6.9	0.284	0.443		
751077931	730.374	25.078	7.8	0.225	0.366		
751077932	730.708	25.043	7.4	0.282	0.38		
751077933	731.043	25.045	-8.2	0.47	0.34		
751077934	669.346	29.801	4.1	0.412	0.164		
751077935	669.847	29.825	4	0.423	0.152		
751077936	670.348	29.821	4.7	0.324	0.161		
751077937	670.847	29.821	5.1	0.271	0.176		
751077938	733.687	18.464	-7	0.313	0.039	Tax_Id=9606 Gene_Symbol=CHGA Chromogranin-A	SGEATDGARPQALPEPMQESK
751077939	734.021	18.473	-7.2	0.293	0.038	Tax_Id=9606 Gene_Symbol=CHGA Chromogranin-A	SGEATDGARPQALPEPMQESK
751077940	734.355	18.471	-7.1	0.268	0.029	Tax_Id=9606 Gene_Symbol=CHGA Chromogranin-A	SGEATDGARPQALPEPMQESK
751077941	734.69	18.484	-4.3	0.752	0.101	Tax_Id=9606 Gene_Symbol=CHGA Chromogranin-A	SGEATDGARPQALPEPMQESK
751077942	735.024	18.487	-6.5	0.493	0.023	Tax_Id=9606 Gene_Symbol=CHGA Chromogranin-A	SGEATDGARPQALPEPMQESK
751077943	735.358	18.495	-25.7	0.142	0.056	Tax_Id=9606 Gene_Symbol=CHGA Chromogranin-A	SGEATDGARPQALPEPMQESK
751077944	626.817	20.729	4.3	0.322	0.471	Tax_Id=9606 Gene_Symbol=APOA1 Apolipoprotein A-I	VQPYLDDFQK
751077945	627.318	20.728	4.1	0.353	0.446	Tax_Id=9606 Gene_Symbol=APOA1 Apolipoprotein A-I	VQPYLDDFQK

751077946	627.82	20.733	4.4	0.31	0.504	Tax_Id=9606 Gene_Symbol=APOA1 Apolipoprotein A-I	VQPYLDDFQK
751077947	628.322	20.63	5.1	0.235	0.566	Tax_Id=9606 Gene_Symbol=APOA1 Apolipoprotein A-I	VQPYLDDFQK
751077948	670.632	19.778	9.4	0.185	0.615		
751077949	670.966	19.793	9.1	0.19	0.575		
751077950	671.3	19.808	8.1	0.205	0.531		
751077951	671.634	19.812	7.6	0.219	0.471		
751077952	671.967	19.778	6.1	0.636	0.27		
751077953	493.772	22.645	4.9	0.651	0.222		
751077954	494.274	22.648	4.7	0.657	0.222		
751077955	494.776	22.644	5	0.639	0.249		
751077956	495.274	22.689	3.2	0.85	0.518		
751077957	1087.592	24.389	3.7	0.645	0.391		
751077958	1088.595	24.388	3.7	0.654	0.371		
751077959	1089.597	24.387	5.2	0.714	0.254		
751077960	1090.597	24.396	0.6	0.954	0.081		
751077961	598.727	20.305	1.5	0.893	0.592		
751077962	599.229	20.306	-0.1	0.993	0.539		
751077963	599.729	20.308	6.2	0.591	0.276		
751077964	600.228	20.31	4.8	0.671	0.223		
751077965	600.728	20.338	-1.3	0.906	0.198		
751077966	1137.531	36.479	-0.4	0.914	0.412		
751077967	1137.865	36.467	-0.8	0.838	0.361		
751077968	1138.2	36.472	-0.8	0.847	0.384		
751077969	1138.533	36.468	-0.4	0.927	0.398		
751077970	1138.867	36.463	-0.8	0.85	0.375		
751077971	1139.201	36.449	-0.6	0.888	0.32		
751077972	1139.535	36.442	-0.2	0.96	0.407		
751077973	1139.87	36.438	-2.4	0.579	0.197		
751077974	552.294	20.713	-3.3	0.379	0.341		
751077975	552.796	20.713	-3.2	0.403	0.372		
751077976	553.297	20.718	-3	0.438	0.407		
751077977	553.797	20.727	-0.6	0.951	0.659		
751077978	764.836	26.944	-5.9	0.177	0.08		
751077979	765.087	26.954	-6.3	0.14	0.065		
751077980	765.337	26.961	-6.1	0.144	0.069		
751077981	765.588	26.975	-6.2	0.14	0.062		
751077982	765.839	26.982	-6.1	0.146	0.068		
751077983	766.09	26.991	-7.2	0.09	0.03		
751077984	766.34	26.979	-4.6	0.327	0.134		
751077985	749.408	17.992	3.7	0.575	0.676	Tax_Id=9606 Gene_Symbol=APOE Apolipoprotein E	AATVGSLAGQPLQER
751077986	749.91	17.993	3.7	0.577	0.657	Tax_Id=9606 Gene_Symbol=APOE Apolipoprotein E	AATVGSLAGQPLQER
751077987	750.411	17.995	3.1	0.627	0.608	Tax_Id=9606 Gene_Symbol=APOE Apolipoprotein E	AATVGSLAGQPLQER
751077988	750.913	18	4.7	0.523	0.8	Tax_Id=9606 Gene_Symbol=APOE Apolipoprotein E	AATVGSLAGQPLQER
751077989	751.415	18.014	-1	0.964	0.454	Tax_Id=9606 Gene_Symbol=APOE Apolipoprotein E	AATVGSLAGQPLQER
751077990	784.274	51.001	4.7	0.419	0.55		
751077991	784.4	51.057	3	0.555	0.364		
751077992	784.523	51.001	4.1	0.465	0.473		
751077993	784.649	51.1	4.4	0.381	0.781		
751077994	784.773	51.001	4	0.507	0.596		
751077995	603.319	20.166	-11.1	0.011	0.018		
751077996	603.52	20.17	-11	0.01	0.017		
751077997	603.72	20.176	-11	0.011	0.017		
751077998	603.921	20.18	-10.7	0.013	0.023		
751077999	604.121	20.186	-11.2	0.011	0.021		
751078000	604.321	20.211	-10.2	0.013	0.019		
751078001	604.523	20.213	-33.6	0.003	0.016		
751078002	424.56	15.567	5.4	0.51	0.778		
751078003	424.895	15.566	5.4	0.51	0.803		
751078004	425.229	15.566	5.5	0.514	0.795		
751078005	425.563	15.574	10.1	0.384	0.599		
751078006	425.897	15.579	-19.2	0.743	0.789		
751078007	579.842	17.119	6.6	0.233	0.505		
751078008	580.344	17.116	6.2	0.243	0.447		
751078009	580.845	17.117	6.5	0.209	0.468		
751078010	581.346	17.122	5.8	0.384	0.241		
751078011	700.843	27.416	3	0.583	0.354	Tax_Id=9606 Gene_Symbol=APOA1 Apolipoprotein A-I	DYVSQFEGSALGK
751078012	701.344	27.417	1.8	0.736	0.27	Tax_Id=9606 Gene_Symbol=APOA1 Apolipoprotein A-I	DYVSQFEGSALGK
751078013	701.846	27.42	3.2	0.539	0.375	Tax_Id=9606 Gene_Symbol=APOA1 Apolipoprotein A-I	DYVSQFEGSALGK
751078014	702.347	27.422	3.3	0.651	0.332	Tax_Id=9606 Gene_Symbol=APOA1 Apolipoprotein A-I	DYVSQFEGSALGK
751078015	646.32	25.127	2.5	0.642	0.884		
751078016	646.655	25.135	3.1	0.585	0.945		
751078017	646.99	25.123	2.6	0.647	0.928		
751078018	647.325	25.113	2.9	0.62	0.986		
751078019	647.66	25.114	3	0.619	0.98		
751078020	647.995	25.063	-4.1	0.829	0.307		
751078021	719.08	25.443	7.7	0.334	0.678	Tax_Id=9606 Gene_Symbol=GSN Isoform 1 of Gelsolin	VPFDAATLHTSTAMAAQHGMDDDGTGQK
751078022	719.331	25.446	7.4	0.339	0.65	Tax_Id=9606 Gene_Symbol=GSN Isoform 1 of Gelsolin	VPFDAATLHTSTAMAAQHGMDDDGTGQK
751078023	719.582	25.448	7.7	0.331	0.675	Tax_Id=9606 Gene_Symbol=GSN Isoform 1 of Gelsolin	VPFDAATLHTSTAMAAQHGMDDDGTGQK
751078024	719.832	25.459	6.3	0.38	0.592	Tax_Id=9606 Gene_Symbol=GSN Isoform 1 of Gelsolin	VPFDAATLHTSTAMAAQHGMDDDGTGQK
751078025	720.082	25.46	8.5	0.329	0.762	Tax_Id=9606 Gene_Symbol=GSN Isoform 1 of Gelsolin	VPFDAATLHTSTAMAAQHGMDDDGTGQK
751078026	720.334	25.473	5.6	0.378	0.718	Tax_Id=9606 Gene_Symbol=GSN Isoform 1 of Gelsolin	VPFDAATLHTSTAMAAQHGMDDDGTGQK
751078027	720.582	25.472	5.1	0.399	0.315	Tax_Id=9606 Gene_Symbol=GSN Isoform 1 of Gelsolin	VPFDAATLHTSTAMAAQHGMDDDGTGQK
751078028	798.594	66.083	2.1	0.806	0.32		
751078029	799.597	66.082	2.2	0.785	0.302		
751078030	800.6	66.082	2.5	0.746	0.276		
751078031	801.605	66.071	1.3	0.853	0.257		
751078032	1089.854	41.364	3.9	0.582	0.751	Tax_Id=9606 Gene_Symbol=PTGDS Prostaglandin-H2 D-isomerase	SPHWGSTYSVSVVETDYDQYALLYSQGSK
751078033	1090.188	41.382	3.5	0.631	0.69	Tax_Id=9606 Gene_Symbol=PTGDS Prostaglandin-H2 D-isomerase	SPHWGSTYSVSVVETDYDQYALLYSQGSK
751078034	1090.522	41.373	3.1	0.674	0.695	Tax_Id=9606 Gene_Symbol=PTGDS Prostaglandin-H2 D-isomerase	SPHWGSTYSVSVVETDYDQYALLYSQGSK
751078035	1090.857	41.376	2.9	0.678	0.619	Tax_Id=9606 Gene_Symbol=PTGDS Prostaglandin-H2 D-isomerase	SPHWGSTYSVSVVETDYDQYALLYSQGSK
751078036	1091.19	41.352	2.6	0.704	0.659	Tax_Id=9606 Gene_Symbol=PTGDS Prostaglandin-H2 D-isomerase	SPHWGSTYSVSVVETDYDQYALLYSQGSK
751078037	1091.524	41.287	1.3	0.834	0.584	Tax_Id=9606 Gene_Symbol=PTGDS Prostaglandin-H2 D-isomerase	SPHWGSTYSVSVVETDYDQYALLYSQGSK
751078038	1091.858	41.162	-1.6	0.778	0.322	Tax_Id=9606 Gene_Symbol=PTGDS Prostaglandin-H2 D-isomerase	SPHWGSTYSVSVVETDYDQYALLYSQGSK

751078039	1092.196	41.19	-7	0.359	0.107	Tax_Id=9606 Gene_Symbol=PTGDS Prostaglandin-H2 D-isomerase	SPHWGSTYSVSVVETDYDQYALLYSQGSK
751078040	728.168	52.248	7.2	0.099	0.984		
751078041	728.325	52.261	9.2	0.021	0.991		
751078042	728.484	52.232	7.5	0.088	0.995		
751078043	728.641	52.178	7.2	0.099	0.743		
751078044	693.867	37.167	2.4	0.538	0.355	Tax_Id=9606 Gene_Symbol=APOA1 Apolipoprotein A-I	VSFLSALEEYTK
751078045	694.368	37.152	2.1	0.586	0.325	Tax_Id=9606 Gene_Symbol=APOA1 Apolipoprotein A-I	VSFLSALEEYTK
751078046	694.869	37.167	2.3	0.556	0.321	Tax_Id=9606 Gene_Symbol=APOA1 Apolipoprotein A-I	VSFLSALEEYTK
751078047	695.37	37.15	0.3	0.931	0.18	Tax_Id=9606 Gene_Symbol=APOA1 Apolipoprotein A-I	VSFLSALEEYTK
751078048	695.872	37.119	2.7	0.454	0.219	Tax_Id=9606 Gene_Symbol=APOA1 Apolipoprotein A-I	VSFLSALEEYTK
751078049	613.778	17.887	3.6	0.532	0.273		
751078050	614.279	17.873	3.8	0.531	0.277		
751078051	614.781	17.898	4	0.53	0.306		
751078052	615.286	17.766	2	0.779	0.353		
751078053	784.042	46.511	2	0.769	0.514	Tax_Id=9606 Gene_Symbol=APOA2 Apolipoprotein A-II	EPC[160.0302]VESLVSQYFQTVTDYGK
751078054	784.376	46.511	2.4	0.722	0.548	Tax_Id=9606 Gene_Symbol=APOA2 Apolipoprotein A-II	EPC[160.0302]VESLVSQYFQTVTDYGK
751078055	784.71	46.512	2.9	0.681	0.587	Tax_Id=9606 Gene_Symbol=APOA2 Apolipoprotein A-II	EPC[160.0302]VESLVSQYFQTVTDYGK
751078056	785.044	46.517	2.5	0.715	0.542	Tax_Id=9606 Gene_Symbol=APOA2 Apolipoprotein A-II	EPC[160.0302]VESLVSQYFQTVTDYGK
751078057	785.378	46.518	1.4	0.836	0.466	Tax_Id=9606 Gene_Symbol=APOA2 Apolipoprotein A-II	EPC[160.0302]VESLVSQYFQTVTDYGK
751078058	785.712	46.523	0.8	0.905	0.475	Tax_Id=9606 Gene_Symbol=APOA2 Apolipoprotein A-II	EPC[160.0302]VESLVSQYFQTVTDYGK
751078059	1046.204	51.202	14.2	0.007	0.734		
751078060	607.837	17.461	0.9	0.908	0.376	Tax_Id=9606 Gene_Symbol=SERPINF1 Pigment epithelium-derived factor	ELLDTVTAPQK
751078061	608.339	17.463	2.2	0.779	0.453	Tax_Id=9606 Gene_Symbol=SERPINF1 Pigment epithelium-derived factor	ELLDTVTAPQK
751078062	608.841	17.446	1.8	0.816	0.417	Tax_Id=9606 Gene_Symbol=SERPINF1 Pigment epithelium-derived factor	ELLDTVTAPQK
751078063	609.342	17.457	4.6	0.776	0.499	Tax_Id=9606 Gene_Symbol=SERPINF1 Pigment epithelium-derived factor	ELLDTVTAPQK
751078064	605.958	25.822	5.4	0.159	0.851		
751078065	606.292	25.822	5.7	0.14	0.868		
751078066	606.627	25.824	5.8	0.137	0.895		
751078067	606.961	25.827	5.9	0.122	0.965		
751078068	607.295	25.829	5	0.325	0.548		
751078069	464.816	19.909	0.8	0.845	0.115		
751078070	465.017	19.906	0.7	0.864	0.121		
751078071	465.217	19.909	0.6	0.887	0.103		
751078072	465.418	19.903	0.9	0.817	0.108		
751078073	465.618	19.921	0.6	0.887	0.115		
751078074	465.819	19.884	0.9	0.948	0.219		
751078075	466.019	19.863	-26.6	0.164	0.057		
751078076	734.717	35.865	6.3	0.509	0.414	Tax_Id=9606 Gene_Symbol=APOA1 Apolipoprotein A-I	LREQLGPVTQEFWDNLEK
751078077	735.051	35.866	6.2	0.535	0.407	Tax_Id=9606 Gene_Symbol=APOA1 Apolipoprotein A-I	LREQLGPVTQEFWDNLEK
751078078	735.385	35.87	5.8	0.536	0.388	Tax_Id=9606 Gene_Symbol=APOA1 Apolipoprotein A-I	LREQLGPVTQEFWDNLEK
751078079	735.719	35.87	7.7	0.647	0.374	Tax_Id=9606 Gene_Symbol=APOA1 Apolipoprotein A-I	LREQLGPVTQEFWDNLEK
751078080	736.054	35.869	9.5	0.402	0.369	Tax_Id=9606 Gene_Symbol=APOA1 Apolipoprotein A-I	LREQLGPVTQEFWDNLEK
751078081	613.303	30.874	10.8	0.275	0.678	Tax_Id=9606 Gene_Symbol=HPX Hemopexin	SGAQATWTELPWPHEK
751078082	613.638	30.871	12	0.271	0.732	Tax_Id=9606 Gene_Symbol=HPX Hemopexin	SGAQATWTELPWPHEK
751078083	613.972	30.866	11.2	0.273	0.679	Tax_Id=9606 Gene_Symbol=HPX Hemopexin	SGAQATWTELPWPHEK
751078084	614.306	30.877	8.4	0.161	0.988	Tax_Id=9606 Gene_Symbol=HPX Hemopexin	SGAQATWTELPWPHEK
751078085	614.64	30.885	13.4	0.252	0.506	Tax_Id=9606 Gene_Symbol=HPX Hemopexin	SGAQATWTELPWPHEK
751078086	1240.237	36.234	0.9	0.819	0.555		
751078087	1240.571	36.233	0.9	0.83	0.582		
751078088	1240.905	36.235	0.9	0.826	0.589		
751078089	1241.24	36.233	0.8	0.828	0.573		
751078090	1241.574	36.234	0.6	0.868	0.559		
751078091	1241.908	36.233	1	0.796	0.599		
751078092	1242.24	36.225	1.5	0.725	0.8		
751078093	1242.571	36.192	1.4	0.712	0.757		
751078094	784.336	51.004	3.6	0.528	0.525		
751078095	784.462	51.001	4.2	0.467	0.596		
751078096	784.585	50.999	3.8	0.487	0.488		
751078097	784.711	51.004	1.9	0.743	0.346		
751078098	784.836	51.006	4.3	0.45	0.504		
751078099	772.068	21.566	6.1	0.124	0.996		
751078100	772.403	21.527	6.6	0.1	0.896		
751078101	772.737	21.555	6.5	0.109	0.926		
751078102	773.071	21.547	6.2	0.122	0.957		
751078103	773.406	21.439	8.6	0.063	0.652		
751078104	773.739	21.511	9	0.534	0.491		
751078105	816.834	51.972	14.7	0.236	0.467		
751078106	816.953	52.175	7.5	0.071	0.747		
751078107	817.073	52.179	8.3	0.044	0.615		
751078108	817.247	52.139	9.8	0.014	0.793		
751078109	817.366	52.183	7.4	0.048	0.743		
751078110	817.483	52.221	9.2	0.011	0.594		
751078111	817.6	52.167	9.3	0.007	0.704		
751078112	496.562	15.421	5	0.74	0.404		
751078113	496.896	15.423	2.7	0.755	0.348		
751078114	497.231	15.42	4.6	0.762	0.396		
751078115	497.565	15.425	4.1	0.847	0.338		
751078116	497.899	15.432	-26.6	0.193	0.011		
751078117	855.498	16.932	5.5	0.522	0.97		
751078118	856.501	16.932	5.6	0.529	0.993		
751078119	857.504	16.935	4.3	0.763	0.509		
751078120	913.485	24.575	2.2	0.735	0.396		
751078121	914.488	24.575	1.5	0.81	0.295		
751078122	915.491	24.574	1.4	0.887	0.214		
751078123	916.493	24.6	-13.3	0.565	0.155		
751078124	701.526	50.261	6.4	0.312	0.392		
751078125	701.693	50.261	6.2	0.325	0.434		
751078126	701.86	50.263	6.8	0.292	0.438		
751078127	702.027	50.263	6.6	0.346	0.497		
751078128	702.194	50.265	5.7	0.335	0.488		
751078129	702.361	50.269	6.2	0.286	0.543		
751078130	702.529	50.264	6	0.282	0.691		
751078131	702.696	50.283	-6.2	0.411	0.405		

751078132	746.358	29.363	1.3	0.84	0.304		
751078133	746.861	29.173	1.7	0.771	0.316		
751078134	747.364	29.139	-0.6	0.833	0.126		
751078135	864.577	52.252	8.2	0.022	0.945		
751078136	864.763	52.261	8	0.025	0.924		
751078137	864.951	52.229	9.5	0.014	0.966		
751078138	560.313	15.126	7.7	0.361	0.531		
751078139	560.815	15.125	7.4	0.388	0.51		
751078140	561.316	15.123	8.3	0.605	0.417		
751078141	561.818	15.142	5	0.743	0.178		
751078142	692.331	23.658	-11	0.007	0.002	Tax_Id=9606 Gene_Symbol=CHGA Chromogranin-A	YPGPQAEGDSEGLSQGLVDR
751078143	692.666	23.667	-11	0.006	0.002	Tax_Id=9606 Gene_Symbol=CHGA Chromogranin-A	YPGPQAEGDSEGLSQGLVDR
751078144	693	23.677	-10.1	0.011	0.002	Tax_Id=9606 Gene_Symbol=CHGA Chromogranin-A	YPGPQAEGDSEGLSQGLVDR
751078145	693.334	23.68	-11.1	0.006	0.003	Tax_Id=9606 Gene_Symbol=CHGA Chromogranin-A	YPGPQAEGDSEGLSQGLVDR
751078146	693.669	23.677	-9.5	0.023	0.015	Tax_Id=9606 Gene_Symbol=CHGA Chromogranin-A	YPGPQAEGDSEGLSQGLVDR
751078147	966.262	50.656	1.6	0.758	0.407		
751078148	822.907	26.64	5.4	0.331	0.92	Tax_Id=9606 Gene_Symbol=CST3 Cystatin-C	LVGGPMDASVEEEGVR
751078149	823.409	26.642	5.3	0.352	0.924	Tax_Id=9606 Gene_Symbol=CST3 Cystatin-C	LVGGPMDASVEEEGVR
751078150	823.909	26.642	5.4	0.344	0.915	Tax_Id=9606 Gene_Symbol=CST3 Cystatin-C	LVGGPMDASVEEEGVR
751078151	824.41	26.645	5	0.363	0.992	Tax_Id=9606 Gene_Symbol=CST3 Cystatin-C	LVGGPMDASVEEEGVR
751078152	824.908	26.645	4.7	0.414	0.666	Tax_Id=9606 Gene_Symbol=CST3 Cystatin-C	LVGGPMDASVEEEGVR
751078153	804.631	36.612	16	0.223	0.43		
751078154	804.882	36.615	16.4	0.21	0.413		
751078155	805.132	36.616	16	0.221	0.406		
751078156	805.383	36.623	15.8	0.221	0.349		
751078157	805.633	36.636	13.8	0.274	0.453		
751078158	805.883	36.641	12.3	0.307	0.524		
751078159	806.133	36.649	1.3	0.915	0.166		
751078160	897.312	50.374	0.4	0.94	0.398		
751078161	897.386	50.4	-1.2	0.834	0.168		
751078162	897.531	50.397	-0.5	0.93	0.272		
751078163	897.672	50.406	-0.5	0.926	0.272		
751078164	897.816	50.388	-0.5	0.92	0.29		
751078165	897.96	50.392	2.1	0.696	0.289		
751078166	898.099	50.466	0.5	0.905	0.171		
751078167	633.372	38.954	3.3	0.494	0.173	Tax_Id=9606 Gene_Symbol=AGT Angiotensinogen	QPFVQGLALYTPVVLPR
751078168	633.706	38.953	3.7	0.419	0.178	Tax_Id=9606 Gene_Symbol=AGT Angiotensinogen	QPFVQGLALYTPVVLPR
751078169	634.041	38.953	3.8	0.414	0.166	Tax_Id=9606 Gene_Symbol=AGT Angiotensinogen	QPFVQGLALYTPVVLPR
751078170	634.375	38.954	3.1	0.625	0.308	Tax_Id=9606 Gene_Symbol=AGT Angiotensinogen	QPFVQGLALYTPVVLPR
751078171	634.709	38.957	4.4	0.773	0.217	Tax_Id=9606 Gene_Symbol=AGT Angiotensinogen	QPFVQGLALYTPVVLPR
751078172	635.044	38.963	-6.5	0.644	0.227	Tax_Id=9606 Gene_Symbol=AGT Angiotensinogen	QPFVQGLALYTPVVLPR
751078173	897.46	50.404	-0.6	0.908	0.362		
751078174	897.601	50.393	-0.3	0.955	0.207		
751078175	897.743	50.39	-1.9	0.722	0.191		
751078176	897.889	50.383	-1.3	0.806	0.251		
751078177	898.029	50.407	0.7	0.886	0.131		
751078178	758.04	35.642	5.9	0.46	0.464		
751078179	758.373	35.642	5.5	0.468	0.456		
751078180	758.706	35.647	4.3	0.523	0.369		
751078181	759.039	35.652	4.3	0.48	0.388		
751078182	759.378	35.575	3.8	0.525	0.459		
751078183	759.714	35.724	-9	0.497	0.037		
751078184	817.013	52.155	8.3	0.051	0.807		
751078185	817.132	52.307	8.1	0.043	0.735		
751078186	817.306	52.256	7.2	0.068	0.833		
751078187	817.424	52.232	8.2	0.024	0.978		
751078188	817.542	52.207	6.9	0.051	0.823		
751078189	697.819	23.926	7.3	0.315	0.604	Tax_Id=9606 Gene_Symbol=TTR Transthyretin	AADDTWEPFASGK
751078190	698.32	23.931	7.1	0.292	0.582	Tax_Id=9606 Gene_Symbol=TTR Transthyretin	AADDTWEPFASGK
751078191	698.821	23.927	8.1	0.309	0.662	Tax_Id=9606 Gene_Symbol=TTR Transthyretin	AADDTWEPFASGK
751078192	699.325	23.967	9.3	0.148	0.934	Tax_Id=9606 Gene_Symbol=TTR Transthyretin	AADDTWEPFASGK
751078193	901.144	49.202	6.7	0.436	0.984		
751078194	901.311	49.272	4.6	0.594	0.911		
751078195	901.478	49.292	3.1	0.7	0.742		
751078196	901.645	49.347	2.6	0.736	0.76		
751078197	901.812	49.362	0.8	0.904	0.574		
751078198	901.979	49.352	-0.1	0.993	0.514		
751078199	902.145	49.371	0.3	0.963	0.589		
751078200	902.312	49.386	-0.2	0.974	0.58		
751078201	902.48	49.376	0.4	0.931	0.366		
751078202	902.647	49.414	-3.7	0.354	0.206		
751078203	705.327	29.98	1.3	0.798	0.235		
751078204	705.828	29.987	1.4	0.782	0.267		
751078205	706.332	29.755	1.1	0.86	0.225		
751078206	706.83	30.009	1.2	0.829	0.301		
751078207	708.679	31.195	0.8	0.929	0.498		
751078208	709.013	31.196	0.8	0.92	0.528		
751078209	709.347	31.199	1.2	0.886	0.515		
751078210	709.681	31.199	1.5	0.871	0.605		
751078211	710.016	31.197	-1.3	0.881	0.351		
751078212	710.346	31.148	-17.6	0.267	0.19		
751078213	572.807	16.848	4.2	0.58	0.996	Tax_Id=9606 Gene_Symbol=ITIH4 Isoform 2 of Inter-alpha-trypsin inhibitor h	GPDVLTATVSGK
751078214	573.309	16.85	4.5	0.543	0.999	Tax_Id=9606 Gene_Symbol=ITIH4 Isoform 2 of Inter-alpha-trypsin inhibitor h	GPDVLTATVSGK
751078215	573.809	16.84	3.6	0.639	0.926	Tax_Id=9606 Gene_Symbol=ITIH4 Isoform 2 of Inter-alpha-trypsin inhibitor h	GPDVLTATVSGK
751078216	574.31	16.849	-13.3	0.48	0.261	Tax_Id=9606 Gene_Symbol=ITIH4 Isoform 2 of Inter-alpha-trypsin inhibitor h	GPDVLTATVSGK
751078217	968.981	25.139	2.1	0.722	0.825		
751078218	969.483	25.143	2.8	0.646	0.91		
751078219	969.984	25.146	2.1	0.723	0.816		
751078220	970.485	25.143	1.1	0.838	0.666		
751078221	970.987	25.142	-21.8	0.032	0.038		
751078222	600.97	19.377	0.6	0.901	0.367		
751078223	601.305	19.426	0.4	0.935	0.361		
751078224	601.639	19.439	0.2	0.963	0.39		

751078225	601.973	19.371	-0.1	0.976	0.364		
751078226	602.308	19.332	-3	0.545	0.212		
751078227	602.648	19.429	-1.9	0.902	0.711		
751078228	615.297	32.413	7.4	0.219	0.753		
751078229	615.631	32.409	8.1	0.196	0.659		
751078230	615.965	32.424	7.6	0.228	0.667		
751078231	616.302	32.521	8	0.247	0.643		
751078232	616.64	32.634	5.8	0.33	0.947		
751078233	704.418	28.543	2.7	0.514	0.292	Tax_Id=9606 Gene_Symbol=KLK6 Isoform 2 of Kallikrein-6	LSELIQPLPLER
751078234	704.92	28.543	2.5	0.554	0.301	Tax_Id=9606 Gene_Symbol=KLK6 Isoform 2 of Kallikrein-6	LSELIQPLPLER
751078235	705.422	28.543	3.7	0.433	0.466	Tax_Id=9606 Gene_Symbol=KLK6 Isoform 2 of Kallikrein-6	LSELIQPLPLER
751078236	705.923	28.532	1.5	0.74	0.3	Tax_Id=9606 Gene_Symbol=KLK6 Isoform 2 of Kallikrein-6	LSELIQPLPLER
751078237	738.371	25.014	5.7	0.371	0.975		
751078238	738.704	25.022	4.9	0.394	0.957		
751078239	739.038	25.044	2.8	0.542	0.707		
751078240	739.372	25.04	2.3	0.505	0.525		
751078241	739.707	25.05	1.6	0.599	0.372		
751078242	740.039	25.013	-0.8	0.802	0.302		
751078243	826.061	34.73	-7.8	0.227	0.07	Tax_Id=9606 Gene_Symbol=CHGA Chromogranin-A	HSGFEDELSEVLENQSSQAELK
751078244	826.395	34.734	-7.8	0.239	0.079	Tax_Id=9606 Gene_Symbol=CHGA Chromogranin-A	HSGFEDELSEVLENQSSQAELK
751078245	826.73	34.743	-7.9	0.223	0.075	Tax_Id=9606 Gene_Symbol=CHGA Chromogranin-A	HSGFEDELSEVLENQSSQAELK
751078246	827.064	34.745	-8.1	0.202	0.072	Tax_Id=9606 Gene_Symbol=CHGA Chromogranin-A	HSGFEDELSEVLENQSSQAELK
751078247	827.398	34.716	-7	0.226	0.048	Tax_Id=9606 Gene_Symbol=CHGA Chromogranin-A	HSGFEDELSEVLENQSSQAELK
751078248	827.734	34.726	-29	0.024	0.004	Tax_Id=9606 Gene_Symbol=CHGA Chromogranin-A	HSGFEDELSEVLENQSSQAELK
751078249	728.116	52.125	10	0.013	0.913		
751078250	728.43	52.289	7.7	0.078	0.896		
751078251	837.627	50.749	2	0.709	0.449		
751078252	837.76	50.773	-0.2	0.961	0.288		
751078253	837.893	50.758	0.8	0.883	0.338		
751078254	838.026	50.75	-0.9	0.861	0.243		
751078255	838.159	50.756	0.2	0.975	0.285		
751078256	529.267	17.91	9.4	0.077	0.757		
751078257	530.271	17.913	9.4	0.079	0.751		
751078258	531.275	17.895	22.4	0.211	0.676		
751078259	955.74	32.04	4.1	0.595	0.591		
751078260	956.242	32.057	3.8	0.61	0.575		
751078261	837.694	50.75	-0.9	0.846	0.201		
751078262	837.826	50.747	0.9	0.864	0.353		
751078263	837.96	50.769	1.6	0.732	0.434		
751078264	838.093	50.757	0.3	0.949	0.391		
751078265	771.515	52.225	11.8	0.085	0.935		
751078266	771.68	51.813	8	0.061	0.561		
751078267	771.846	52.198	7.1	0.108	0.939		
751078268	771.955	52.16	9.1	0.024	0.805		
751078269	772.124	52.243	6.4	0.135	0.91		
751078270	772.237	51.989	5.6	0.117	0.843		
751078271	678.348	27.687	8.8	0.167	0.388		
751078272	678.85	27.686	8.6	0.169	0.385		
751078273	679.352	27.687	9.4	0.133	0.339		
751078274	679.853	27.684	8.9	0.17	0.852		
751078275	680.358	27.733	5.2	0.725	0.72		
751078276	1005.447	19.785	7.7	0.273	0.453		
751078277	1005.948	19.807	5.2	0.331	0.208		
751078278	1006.449	19.825	4.8	0.354	0.193		
751078279	1006.95	19.834	4.1	0.68	0.165		
751078280	1007.451	19.836	4.6	0.712	0.146		
751078281	1007.953	19.88	29.5	0.363	0.949		
751078282	732.834	19.465	1	0.862	0.278	Tax_Id=9606 Gene_Symbol=DKK3 cDNA FLJ52545, highly similar to Dickko	SAVEEMEAEEAAAK
751078283	733.335	19.48	0.7	0.896	0.259	Tax_Id=9606 Gene_Symbol=DKK3 cDNA FLJ52545, highly similar to Dickko	SAVEEMEAEEAAAK
751078284	733.836	19.5	1	0.86	0.232	Tax_Id=9606 Gene_Symbol=DKK3 cDNA FLJ52545, highly similar to Dickko	SAVEEMEAEEAAAK
751078285	734.338	19.524	-2.9	0.692	0.184	Tax_Id=9606 Gene_Symbol=DKK3 cDNA FLJ52545, highly similar to Dickko	SAVEEMEAEEAAAK
751078286	734.839	19.497	-33.6	0.102	0.007	Tax_Id=9606 Gene_Symbol=DKK3 cDNA FLJ52545, highly similar to Dickko	SAVEEMEAEEAAAK
751078287	550.29	18.3	-13.8	0.008	0.007		
751078288	550.491	18.301	-14.2	0.005	0.004		
751078289	550.691	18.302	-14.9	0.003	0.004		
751078290	550.892	18.305	-15.1	0.004	0.003		
751078291	551.092	18.309	-19.1	0	0.001		
751078292	551.298	18.288	-9.2	0.444	0.221		
751078293	489.768	18.197	11.7	0.018	0.768		
751078294	490.269	18.194	11.9	0.021	0.747		
751078295	490.771	18.197	12.6	0.079	0.462		
751078296	528.328	17.423	2.5	0.665	0.464	Tax_Id=9606 Gene_Symbol=SERPINF1 Pigment epithelium-derived factor	TVQAVLTVPK
751078297	528.83	17.428	2.3	0.682	0.446	Tax_Id=9606 Gene_Symbol=SERPINF1 Pigment epithelium-derived factor	TVQAVLTVPK
751078298	529.331	17.431	2.2	0.708	0.438	Tax_Id=9606 Gene_Symbol=SERPINF1 Pigment epithelium-derived factor	TVQAVLTVPK
751078299	529.833	17.449	-5.2	0.775	0.393	Tax_Id=9606 Gene_Symbol=SERPINF1 Pigment epithelium-derived factor	TVQAVLTVPK
751078300	607.312	35.902	0.9	0.857	0.892		
751078301	607.81	35.831	3.3	0.359	0.795		
751078302	608.313	35.88	3	0.385	0.668		
751078303	608.815	35.908	0.5	0.894	0.985		
751078304	460.911	18.121	6.6	0.074	0.604		
751078305	461.246	18.127	6.3	0.087	0.544		
751078306	461.58	18.137	6.2	0.102	0.535		
751078307	461.914	18.135	7	0.095	0.607		
751078308	850.436	22.117	8.9	0.09	0.692		
751078309	851.44	22.116	8.7	0.1	0.66		
751078310	852.441	22.127	9.3	0.282	0.373		
751078311	582.964	30.376	3.9	0.565	0.761		
751078312	583.298	30.379	4.6	0.504	0.881		
751078313	583.633	30.373	3.9	0.581	0.833		
751078314	583.967	30.365	4.7	0.508	0.92		
751078315	584.3	30.361	-3.8	0.694	0.037		
751078316	499.94	17.993	2.1	0.703	0.409		
751078317	500.275	17.993	2.4	0.665	0.451		

751078318	500.609	17.995	2.5	0.667	0.434		
751078319	500.943	18.001	4.8	0.825	0.435		
751078320	501.278	18.007	-39.1	0.064	0.072		
751078321	966.416	50.423	1.6	0.79	0.44		
751078322	966.493	50.417	0.3	0.955	0.374		
751078323	966.646	50.411	-0.8	0.894	0.165		
751078324	966.801	50.425	0.9	0.925	0.154		
751078325	966.954	50.434	3.2	0.756	0.163		
751078326	585.317	29.646	2.8	0.547	0.476		
751078327	585.818	29.657	2.7	0.57	0.505		
751078328	586.319	29.666	2.4	0.578	0.533		
751078329	586.821	29.7	-35.7	0.17	0.26		
751078330	1045.536	51.068	2.2	0.663	0.29		
751078331	1045.783	50.999	3.9	0.475	0.468		
751078332	1046.036	51.02	3.4	0.521	0.304		
751078333	548.937	21.415	0.2	0.979	0.286		
751078334	549.27	21.407	-0.2	0.971	0.245		
751078335	549.605	21.41	0.3	0.963	0.264		
751078336	549.939	21.407	-0.6	0.926	0.081		
751078337	550.273	21.326	2.3	0.91	0.475		
751078338	966.569	50.409	0.4	0.939	0.298		
751078339	966.724	50.41	-0.3	0.959	0.218		
751078340	471.288	16.743	4.8	0.4	0.963		
751078341	471.79	16.742	5.5	0.345	0.955		
751078342	472.292	16.743	12.6	0.066	0.638		
751078343	697.357	34.532	2.6	0.674	0.725	Tax_Id=9606 Gene_Symbol=CLU Isoform 2 of Clusterin	ASSIIDELFQDR
751078344	697.859	34.554	2.4	0.694	0.702	Tax_Id=9606 Gene_Symbol=CLU Isoform 2 of Clusterin	ASSIIDELFQDR
751078345	698.36	34.563	2	0.763	0.789	Tax_Id=9606 Gene_Symbol=CLU Isoform 2 of Clusterin	ASSIIDELFQDR
751078346	698.862	34.537	1.1	0.878	0.536	Tax_Id=9606 Gene_Symbol=CLU Isoform 2 of Clusterin	ASSIIDELFQDR
751078347	847.094	48.524	-2.2	0.695	0.24		
751078348	847.428	48.528	-2.5	0.678	0.243		
751078349	847.763	48.535	-2.1	0.744	0.299		
751078350	848.097	48.54	-2.4	0.688	0.268		
751078351	848.431	48.552	-4.5	0.435	0.237		
751078352	848.766	48.54	-15.2	0.017	0.014		
751078353	618.321	34.654	7.2	0.354	0.582		
751078354	618.822	34.662	6.9	0.374	0.604		
751078355	619.323	34.576	7.6	0.285	0.85		
751078356	619.827	34.539	10.6	0.627	0.639		
751078357	534.258	22.077	1.4	0.866	0.751		
751078358	534.592	22.075	1.5	0.857	0.754		
751078359	534.926	22.076	-0.9	0.929	0.143		
751078360	535.26	22.087	-0.6	0.96	0.225		
751078361	535.594	22.074	-48.9	0.006	0.003		
751078362	386.237	15.192	0.9	0.854	0.495		
751078363	386.571	15.192	2	0.688	0.575		
751078364	386.905	15.195	9.5	0.223	0.736		
751078365	387.24	15.196	-25.5	0.23	0.046		
751078366	1025.113	37.654	0.7	0.843	0.146		
751078367	1025.447	37.62	0.8	0.827	0.142		
751078368	1025.781	37.625	1.1	0.749	0.175		
751078369	1026.116	37.628	1.2	0.748	0.201		
751078370	1026.45	37.626	0.8	0.818	0.115		
751078371	1026.784	37.606	1.3	0.71	0.179		
751078372	1027.12	37.494	0.8	0.806	0.169		
751078373	775.917	21.239	7.5	0.395	0.246		
751078374	776.419	21.245	7.7	0.393	0.239		
751078375	776.919	21.235	6.6	0.448	0.284		
751078376	777.415	21.372	5.9	0.443	0.336		
751078377	516.265	16.664	5	0.445	0.35	Tax_Id=9606 Gene_Symbol=APOA1 Apolipoprotein A-I	LSPLGEEMR
751078378	516.766	16.665	3.9	0.689	0.262	Tax_Id=9606 Gene_Symbol=APOA1 Apolipoprotein A-I	LSPLGEEMR
751078379	517.267	16.658	3.5	0.834	0.186	Tax_Id=9606 Gene_Symbol=APOA1 Apolipoprotein A-I	LSPLGEEMR
751078380	985.929	34.401	14.4	0.083	0.724		
751078381	986.179	34.396	13.6	0.097	0.761		
751078382	986.429	34.398	13.9	0.095	0.726		
751078383	986.68	34.402	13.8	0.09	0.722		
751078384	986.931	34.41	12.8	0.098	0.773		
751078385	987.181	34.408	11.9	0.113	0.776		
751078386	987.432	34.375	8.8	0.199	0.976		
751078387	987.682	34.381	6.7	0.33	0.655		
751078388	858.777	44.947	0.3	0.948	0.284		
751078389	859.111	44.949	0.7	0.886	0.358		
751078390	859.445	44.923	0	0.996	0.274		
751078391	859.78	44.962	0.9	0.86	0.397		
751078392	860.114	44.972	1.7	0.748	0.631		
751078393	860.448	44.908	-2.2	0.607	0.145		
751078394	771.379	52.223	12.7	0.1	0.989		
751078395	771.56	52.254	12.4	0.034	0.812		
751078396	771.737	52.204	9.8	0.013	0.899		
751078397	981.821	30.658	-1.7	0.763	0.285	Tax_Id=9606 Gene_Symbol=SPARCL1 cDNA FLJ52396, highly similar to SF	KLSENTDFLAPGVSSFTDSNQQESITK
751078398	982.156	30.674	-1.7	0.758	0.275	Tax_Id=9606 Gene_Symbol=SPARCL1 cDNA FLJ52396, highly similar to SF	KLSENTDFLAPGVSSFTDSNQQESITK
751078399	982.49	30.694	-2.1	0.686	0.248	Tax_Id=9606 Gene_Symbol=SPARCL1 cDNA FLJ52396, highly similar to SF	KLSENTDFLAPGVSSFTDSNQQESITK
751078400	982.824	30.714	-2.8	0.606	0.24	Tax_Id=9606 Gene_Symbol=SPARCL1 cDNA FLJ52396, highly similar to SF	KLSENTDFLAPGVSSFTDSNQQESITK
751078401	983.158	30.726	-4.1	0.403	0.133	Tax_Id=9606 Gene_Symbol=SPARCL1 cDNA FLJ52396, highly similar to SF	KLSENTDFLAPGVSSFTDSNQQESITK
751078402	983.487	30.754	-4.2	0.727	0.255	Tax_Id=9606 Gene_Symbol=SPARCL1 cDNA FLJ52396, highly similar to SF	KLSENTDFLAPGVSSFTDSNQQESITK
751078403	647.796	47.63	11.4	0.179	0.964		
751078404	647.939	47.632	12.9	0.143	0.797		
751078405	648.082	47.633	12.3	0.155	0.875		
751078406	648.225	47.637	12.8	0.129	0.856		
751078407	648.368	47.64	11.8	0.153	0.87		
751078408	648.512	47.647	11.7	0.17	0.847		
751078409	648.655	47.644	10.4	0.198	0.925		
751078410	648.798	47.641	11.7	0.205	0.96		

751078411	648.941	47.627	37	0.06	0.323		
751078412	636.844	31.254	4.8	0.639	0.409		
751078413	637.094	31.251	5.6	0.528	0.474		
751078414	637.35	31.118	8.1	0.53	0.698		
751078415	637.596	31.252	3.3	0.717	0.331		
751078416	637.847	31.247	3.8	0.801	0.336		
751078417	638.097	31.261	8.5	0.544	0.179		
751078418	844.813	50.71	10.9	0.109	0.618		
751078419	844.981	50.711	7.8	0.208	0.766		
751078420	845.147	50.71	7.7	0.205	0.808		
751078421	845.315	50.72	7.3	0.233	0.756		
751078422	845.482	50.708	7.9	0.194	0.743		
751078423	845.648	50.706	6.5	0.268	0.946		
751078424	845.816	50.705	9.7	0.14	0.753		
751078425	845.983	50.702	6.7	0.419	0.489		
751078426	488.291	27.427	5.2	0.157	0.643		
751078427	488.625	27.425	4.9	0.195	0.577		
751078428	488.959	27.412	4.5	0.227	0.502		
751078429	489.294	27.401	3.6	0.337	0.314		
751078430	489.628	27.387	46.4	0.09	0.837		
751078431	477.242	16.203	7.2	0.299	0.805		
751078432	477.576	16.209	6.8	0.337	0.794		
751078433	477.91	16.21	7.6	0.319	0.782		
751078434	478.245	16.223	-2.1	0.792	0.237		
751078435	539.776	18.471	-3.9	0.452	0.218		
751078436	540.278	18.473	-5	0.304	0.152		
751078437	540.78	18.479	-4.4	0.405	0.203		
751078438	486.025	19.896	-1.9	0.721	0.345		
751078439	486.225	19.867	-2.8	0.576	0.221		
751078440	486.426	19.918	-1.6	0.77	0.395		
751078441	486.626	19.953	-1.7	0.747	0.377		
751078442	486.826	20.003	-2.3	0.671	0.321		
751078443	487.026	20.084	-2.2	0.723	0.422		
751078444	521.014	24.067	8.5	0.076	0.871		
751078445	521.264	24.066	8.4	0.078	0.845		
751078446	521.515	24.059	8.9	0.08	0.794		
751078447	521.766	24.044	9.1	0.06	0.718		
751078448	522.016	24.051	12	0.431	0.642		
751078449	1046.537	51.243	15	0.004	0.721		
751078450	746.872	29.932	6.1	0.29	0.593		
751078451	747.377	29.863	4.7	0.142	0.142		
751078452	747.879	29.843	8.4	0.188	0.826		
751078453	748.382	29.786	5.3	0.137	0.156		
751078454	586.317	46.089	5.8	0.502	0.883		
751078455	586.652	46.089	6	0.48	0.866		
751078456	586.986	46.085	5.8	0.502	0.899		
751078457	587.32	46.08	4.6	0.58	0.976		
751078458	587.655	46.068	7.6	0.47	0.834		
751078459	1245.57	36.141	1	0.814	0.756		
751078460	1245.905	36.158	1	0.808	0.737		
751078461	1246.239	36.163	0.9	0.83	0.761		
751078462	1246.573	36.175	0.8	0.843	0.73		
751078463	1246.908	36.17	0.9	0.838	0.727		
751078464	1247.242	36.156	-0.1	0.976	0.577		
751078465	1247.573	36.181	0.8	0.848	0.75		
751078466	1247.902	36.208	0	0.992	0.564		
751078467	1248.236	36.23	1.2	0.762	0.794		
751078468	1248.57	36.245	-1.9	0.728	0.655		
751078469	819.062	34.315	-1.1	0.904	0.413	Tax_Id=9606 Gene_Symbol=TTR Transthyretin	TSESGELHGLTTEEEFVEGIYK
751078470	819.397	34.314	0.3	0.978	0.469	Tax_Id=9606 Gene_Symbol=TTR Transthyretin	TSESGELHGLTTEEEFVEGIYK
751078471	819.731	34.313	-4.1	0.702	0.311	Tax_Id=9606 Gene_Symbol=TTR Transthyretin	TSESGELHGLTTEEEFVEGIYK
751078472	820.065	34.311	0.2	0.986	0.44	Tax_Id=9606 Gene_Symbol=TTR Transthyretin	TSESGELHGLTTEEEFVEGIYK
751078473	820.399	34.3	2.9	0.773	0.908	Tax_Id=9606 Gene_Symbol=TTR Transthyretin	TSESGELHGLTTEEEFVEGIYK
751078474	820.735	34.3	11.7	0.699	0.463	Tax_Id=9606 Gene_Symbol=TTR Transthyretin	TSESGELHGLTTEEEFVEGIYK
751078475	830.439	39.675	9.9	0.071	0.796	Tax_Id=9606 Gene_Symbol=TTR Transthyretin	YTIAALLSPYSYSTTAVVTNPKE
751078476	830.775	39.677	9.9	0.072	0.758	Tax_Id=9606 Gene_Symbol=TTR Transthyretin	YTIAALLSPYSYSTTAVVTNPKE
751078477	831.108	39.677	9.8	0.08	0.827	Tax_Id=9606 Gene_Symbol=TTR Transthyretin	YTIAALLSPYSYSTTAVVTNPKE
751078478	831.442	39.677	9.6	0.093	0.805	Tax_Id=9606 Gene_Symbol=TTR Transthyretin	YTIAALLSPYSYSTTAVVTNPKE
751078479	831.777	39.685	11.1	0.093	0.726	Tax_Id=9606 Gene_Symbol=TTR Transthyretin	YTIAALLSPYSYSTTAVVTNPKE
751078480	832.112	39.698	0.2	0.988	0.974	Tax_Id=9606 Gene_Symbol=TTR Transthyretin	YTIAALLSPYSYSTTAVVTNPKE
751078481	690.816	39.82	3.1	0.718	0.206		
751078482	691.317	39.828	2.4	0.779	0.163		
751078483	691.818	39.836	1.9	0.809	0.122		
751078484	692.32	39.827	1.3	0.826	0.112		
751078485	692.822	39.85	0	0.998	0.103		
751078486	501.221	15.288	0.5	0.944	0.346	Tax_Id=9606 Gene_Symbol=SCG5 Isoform 1 of Neuroendocrine protein 7B2	SVPHFSDEDKDPE
751078487	501.556	15.278	0.9	0.883	0.343	Tax_Id=9606 Gene_Symbol=SCG5 Isoform 1 of Neuroendocrine protein 7B2	SVPHFSDEDKDPE
751078488	501.889	15.298	1.2	0.846	0.35	Tax_Id=9606 Gene_Symbol=SCG5 Isoform 1 of Neuroendocrine protein 7B2	SVPHFSDEDKDPE
751078489	502.224	15.223	2	0.923	0.407	Tax_Id=9606 Gene_Symbol=SCG5 Isoform 1 of Neuroendocrine protein 7B2	SVPHFSDEDKDPE
751078490	1342.602	40.299	6.5	0.059	0.894		
751078491	1342.936	40.304	6	0.087	0.836		
751078492	1343.271	40.298	5.9	0.09	0.831		
751078493	1343.604	40.295	5.9	0.085	0.778		
751078494	1343.939	40.287	5.9	0.09	0.78		
751078495	1344.273	40.277	6.5	0.059	0.943		
751078496	1344.608	40.248	5.5	0.128	0.851		
751078497	1344.941	40.199	4.5	0.236	0.763		
751078498	1046.702	50.664	-1.5	0.771	0.175		
751078499	1046.871	50.731	1.7	0.748	0.376		
751078500	1047.034	50.754	0.8	0.871	0.259		
751078501	1047.203	50.735	2.6	0.621	0.572		
751078502	1047.367	50.77	0.4	0.941	0.305		
751078503	729.372	36.164	-13.3	0.11	0.059	Tax_Id=9606 Gene_Symbol=CHGA Chromogranin-A	RPEDQELESLSAIEAELEK

751078504	729.706	36.168	-13	0.145	0.073	Tax_Id=9606 Gene_Symbol=CHGA Chromogranin-A	RPEDQELESLSAIEAELEK
751078505	730.041	36.155	-11.3	0.062	0.034	Tax_Id=9606 Gene_Symbol=CHGA Chromogranin-A	RPEDQELESLSAIEAELEK
751078506	730.374	36.164	-12.5	0.015	0.012	Tax_Id=9606 Gene_Symbol=CHGA Chromogranin-A	RPEDQELESLSAIEAELEK
751078507	730.708	36.196	-26.9	0.006	0.015	Tax_Id=9606 Gene_Symbol=CHGA Chromogranin-A	RPEDQELESLSAIEAELEK
751078508	489.27	16.471	3.1	0.59	0.505		
751078509	489.772	16.469	-18.1	0.231	0.189		
751078510	939.122	33.52	-3.5	0.564	0.415	Tax_Id=9606 Gene_Symbol=SPARCL1 cDNA FLJ52396, highly similar to SF	LSENTDFLAPGVSSFTDSNQQESITK
751078511	939.456	33.554	-3.7	0.546	0.408	Tax_Id=9606 Gene_Symbol=SPARCL1 cDNA FLJ52396, highly similar to SF	LSENTDFLAPGVSSFTDSNQQESITK
751078512	939.791	33.587	-3.8	0.515	0.395	Tax_Id=9606 Gene_Symbol=SPARCL1 cDNA FLJ52396, highly similar to SF	LSENTDFLAPGVSSFTDSNQQESITK
751078513	940.124	33.578	-4.4	0.414	0.303	Tax_Id=9606 Gene_Symbol=SPARCL1 cDNA FLJ52396, highly similar to SF	LSENTDFLAPGVSSFTDSNQQESITK
751078514	940.459	33.605	-7.1	0.193	0.17	Tax_Id=9606 Gene_Symbol=SPARCL1 cDNA FLJ52396, highly similar to SF	LSENTDFLAPGVSSFTDSNQQESITK
751078515	940.793	33.63	-18.4	0.006	0.01	Tax_Id=9606 Gene_Symbol=SPARCL1 cDNA FLJ52396, highly similar to SF	LSENTDFLAPGVSSFTDSNQQESITK
751078516	697.892	26.004	2.4	0.644	0.469		
751078517	698.392	26.015	5.7	0.374	0.938		
751078518	698.895	26.016	3.1	0.6	0.684		
751078519	699.397	26.008	4.3	0.481	0.86		
751078520	1093.555	25.09	6.2	0.336	0.523		
751078521	1094.057	25.091	5.7	0.381	0.501		
751078522	1094.558	25.084	6.2	0.321	0.542		
751078523	1095.06	25.073	5.9	0.349	0.554		
751078524	1095.561	25.049	4	0.534	0.947		
751078525	692.347	27.758	2.1	0.669	0.351	Tax_Id=9606 Gene_Symbol=SERPINF1 Pigment epithelium-derived factor	LQSLFDSPDFSK
751078526	692.849	27.764	2.2	0.674	0.375	Tax_Id=9606 Gene_Symbol=SERPINF1 Pigment epithelium-derived factor	LQSLFDSPDFSK
751078527	693.35	27.766	3	0.59	0.455	Tax_Id=9606 Gene_Symbol=SERPINF1 Pigment epithelium-derived factor	LQSLFDSPDFSK
751078528	693.853	27.768	2.2	0.796	0.654	Tax_Id=9606 Gene_Symbol=SERPINF1 Pigment epithelium-derived factor	LQSLFDSPDFSK
751078529	706.316	15.6	3.3	0.732	0.37	Tax_Id=9606 Gene_Symbol=SPP1 Isoform A of Osteopontin	ANDESNEHSDVIDSQELSK
751078530	706.651	15.609	3	0.757	0.375	Tax_Id=9606 Gene_Symbol=SPP1 Isoform A of Osteopontin	ANDESNEHSDVIDSQELSK
751078531	706.985	15.609	0.7	0.946	0.301	Tax_Id=9606 Gene_Symbol=SPP1 Isoform A of Osteopontin	ANDESNEHSDVIDSQELSK
751078532	707.319	15.608	-1.7	0.857	0.118	Tax_Id=9606 Gene_Symbol=SPP1 Isoform A of Osteopontin	ANDESNEHSDVIDSQELSK
751078533	707.653	15.62	-31.6	0.204	0.076	Tax_Id=9606 Gene_Symbol=SPP1 Isoform A of Osteopontin	ANDESNEHSDVIDSQELSK
751078534	606.836	19.332	-0.1	0.982	0.235		
751078535	607.337	19.337	-0.5	0.936	0.212		
751078536	607.838	19.333	-1.4	0.821	0.226		
751078537	608.341	19.348	-17.8	0.209	0.153		
751078538	531.779	18.588	4.7	0.148	0.799		
751078539	532.28	18.588	5.1	0.123	0.82		
751078540	532.782	18.584	4	0.292	0.971		
751078541	706.331	17.392	-6	0.19	0.089	Tax_Id=9606 Gene_Symbol=CHGB Secretogranin-1	NYLNYGEEGAPGK
751078542	706.833	17.394	-6.5	0.157	0.081	Tax_Id=9606 Gene_Symbol=CHGB Secretogranin-1	NYLNYGEEGAPGK
751078543	707.334	17.394	-6.7	0.132	0.064	Tax_Id=9606 Gene_Symbol=CHGB Secretogranin-1	NYLNYGEEGAPGK
751078544	707.835	17.406	-41.1	0.066	0.109	Tax_Id=9606 Gene_Symbol=CHGB Secretogranin-1	NYLNYGEEGAPGK
751078545	944.447	28.045	-4	0.693	0.072		
751078546	944.647	28.048	7.1	0.536	0.885		
751078547	944.847	28.052	6.8	0.535	0.822		
751078548	945.048	28.053	6.1	0.589	0.741		
751078549	945.248	28.059	-2.8	0.676	0.125		
751078550	945.447	28.069	-2.5	0.715	0.202		
751078551	945.652	28.072	-6.7	0.216	0.029		
751078552	945.851	28.089	-31.1	0.055	0.016		
751078553	946.05	28.109	1.1	0.936	0.374		
751078554	686.851	16.166	2.1	0.797	0.314		
751078555	687.352	16.167	1.2	0.82	0.092		
751078556	687.855	16.165	1.8	0.881	0.171		
751078557	688.357	16.156	-0.6	0.964	0.107		
751078558	522.755	17.836	2.6	0.675	0.925		
751078559	523.257	17.838	2.5	0.707	0.891		
751078560	523.758	17.842	4.1	0.649	0.827		
751078561	524.26	17.858	-1.1	0.97	0.823		
751078562	670.983	26.823	4.2	0.487	0.288		
751078563	671.317	26.826	3.7	0.495	0.263		
751078564	671.651	26.826	4.2	0.525	0.268		
751078565	671.986	26.823	2.7	0.772	0.115		
751078566	672.32	26.831	1.4	0.8	0.316		
751078567	507.283	15.929	6.8	0.175	0.901		
751078568	694.35	24.048	8.8	0.126	0.749	Tax_Id=9606 Gene_Symbol=AHSG Alpha-2-HS-glycoprotein	HTFMGVVSLGSPSGEVSHPR
751078569	694.684	24.044	9.1	0.109	0.786	Tax_Id=9606 Gene_Symbol=AHSG Alpha-2-HS-glycoprotein	HTFMGVVSLGSPSGEVSHPR
751078570	695.019	24.034	7.8	0.109	0.718	Tax_Id=9606 Gene_Symbol=AHSG Alpha-2-HS-glycoprotein	HTFMGVVSLGSPSGEVSHPR
751078571	695.35	24.094	9.3	0.081	0.778	Tax_Id=9606 Gene_Symbol=AHSG Alpha-2-HS-glycoprotein	HTFMGVVSLGSPSGEVSHPR
751078572	695.687	24.031	8.6	0.115	0.603	Tax_Id=9606 Gene_Symbol=AHSG Alpha-2-HS-glycoprotein	HTFMGVVSLGSPSGEVSHPR
751078573	792.377	37.376	4.7	0.443	0.591		
751078574	792.628	37.396	4.5	0.498	0.57		
751078575	792.878	37.414	4	0.577	0.525		
751078576	793.128	37.44	3.8	0.623	0.493		
751078577	793.384	37.424	4.6	0.506	0.6		
751078578	793.628	37.465	4.6	0.439	0.582		
751078579	793.881	37.487	3.9	0.537	0.501		
751078580	469.746	16.394	5	0.342	0.773		
751078581	470.248	16.395	5	0.371	0.746		
751078582	470.749	16.403	9.2	0.381	0.639		
751078583	814.543	51.389	7.1	0.01	0.746		
751078584	814.66	51.484	7.7	0.009	0.709		
751078585	814.778	51.574	6.3	0.031	0.391		
751078586	814.895	51.448	7.4	0.009	0.722		
751078587	815.012	51.418	4.4	0.157	0.475		
751078588	815.131	51.336	5	0.248	0.859		
751078589	1067.842	51.863	-29.7	0.119	0.181	Tax_Id=9606 Gene_Symbol=AGT Angiotensinogen	TIHLTMPQLVLQGSYDLQDLLAQAELPAILHTELN
751078590	1068.094	51.93	3.8	0.505	0.34	Tax_Id=9606 Gene_Symbol=AGT Angiotensinogen	TIHLTMPQLVLQGSYDLQDLLAQAELPAILHTELN
751078591	1068.375	52.003	5	0.276	0.404	Tax_Id=9606 Gene_Symbol=AGT Angiotensinogen	TIHLTMPQLVLQGSYDLQDLLAQAELPAILHTELN
751078592	1068.595	51.88	2.4	0.66	0.305	Tax_Id=9606 Gene_Symbol=AGT Angiotensinogen	TIHLTMPQLVLQGSYDLQDLLAQAELPAILHTELN
751078593	1068.837	51.874	4.5	0.531	0.592	Tax_Id=9606 Gene_Symbol=AGT Angiotensinogen	TIHLTMPQLVLQGSYDLQDLLAQAELPAILHTELN
751078594	1069.099	51.851	3.7	0.677	0.478	Tax_Id=9606 Gene_Symbol=AGT Angiotensinogen	TIHLTMPQLVLQGSYDLQDLLAQAELPAILHTELN
751078595	629.818	42.23	4.4	0.305	0.498		
751078596	630.32	42.223	4.5	0.295	0.511		

751078597	630.821	42.218	4.7	0.266	0.54		
751078598	631.323	42.186	-2.3	0.904	0.311		
751078599	743.871	31.61	7	0.281	0.529		
751078600	744.368	31.544	6.6	0.298	0.561		
751078601	744.873	31.601	8.6	0.531	0.553		
751078602	745.371	31.462	7.3	0.683	0.997		
751078603	508.872	22.932	-4.1	0.38	0.125		
751078604	509.072	22.931	-3.7	0.427	0.122		
751078605	509.273	22.929	-4.3	0.359	0.1		
751078606	509.473	22.931	-4.3	0.355	0.082		
751078607	509.674	22.93	-3.8	0.479	0.162		
751078608	509.875	22.924	-5.7	0.646	0.229		
751078609	865.452	51.392	5.8	0.033	0.195		
751078610	865.577	51.504	7.6	0.01	0.68		
751078611	865.7	51.485	5.5	0.043	0.232		
751078612	865.826	51.452	4.1	0.143	0.255		
751078613	865.951	51.355	6.2	0.053	0.764		
751078614	866.076	51.339	-5.9	0.79	0.67		
751078615	693.828	33.764	2.5	0.753	0.146		
751078616	694.079	33.764	13.4	0.192	0.951		
751078617	694.329	33.766	14.3	0.166	0.955		
751078618	694.58	33.767	14.3	0.212	0.964		
751078619	694.831	34.028	8.3	0.243	0.788		
751078620	695.081	33.773	-1.6	0.898	0.091		
751078621	678.37	16.168	8.4	0.291	0.732		
751078622	678.872	16.169	9.4	0.249	0.656		
751078623	679.373	16.171	6.7	0.402	0.867		
751078624	679.874	16.169	9.2	0.6	0.493		
751078625	813.124	30.923	12.8	0.215	0.991		
751078626	813.375	30.915	9.5	0.321	0.755		
751078627	813.626	30.924	8.7	0.345	0.678		
751078628	813.876	30.924	6.6	0.422	0.606		
751078629	814.126	30.939	7.5	0.385	0.846		
751078630	814.376	30.967	3.9	0.662	0.69		
751078631	814.626	30.997	6.3	0.654	0.354		
751078632	1130.909	52.142	13.7	0.159	0.835		
751078633	966.185	50.645	0.4	0.934	0.248		
751078634	611.765	16.103	57.1	0.036	0.298	Tax_Id=9606 Gene_Symbol=APOE Apolipoprotein E	LGADMEDVC[160.0302]GR
751078635	612.267	16.102	25.3	0.067	0.649	Tax_Id=9606 Gene_Symbol=APOE Apolipoprotein E	LGADMEDVC[160.0302]GR
751078636	612.773	16.071	2	0.801	0.163	Tax_Id=9606 Gene_Symbol=APOE Apolipoprotein E	LGADMEDVC[160.0302]GR
751078637	613.268	16.096	-13.3	0.348	0.111	Tax_Id=9606 Gene_Symbol=APOE Apolipoprotein E	LGADMEDVC[160.0302]GR
751078638	626.362	29.514	3.4	0.461	0.059		
751078639	626.863	29.525	3.5	0.447	0.071		
751078640	627.365	29.539	3.8	0.442	0.074		
751078641	627.867	29.431	6.2	0.608	0.247		
751078642	713.703	29.683	4.1	0.469	0.679		
751078643	714.038	29.683	4.3	0.445	0.674		
751078644	714.371	29.705	1.5	0.793	0.553		
751078645	714.706	29.681	0.6	0.916	0.362		
751078646	715.04	29.693	-17.9	0.277	0.312		
751078647	1007.2	40.29	2.7	0.341	0.748		
751078648	1007.452	40.261	2.8	0.357	0.73		
751078649	1007.703	40.267	2.8	0.348	0.793		
751078650	1007.953	40.261	2.4	0.412	0.74		
751078651	1008.204	40.253	2.8	0.349	0.789		
751078652	1008.455	40.26	3.3	0.231	0.919		
751078653	1008.705	40.168	1.1	0.722	0.452		
751078654	1008.954	40.147	4.2	0.242	0.933		
751078655	574.803	23.232	9.6	0.21	0.475		
751078656	575.305	23.242	9.3	0.222	0.476		
751078657	575.805	23.253	12.5	0.097	0.16		
751078658	666.079	29.594	-3.9	0.367	0.049		
751078659	666.33	29.592	-4.2	0.335	0.054		
751078660	666.581	29.599	-4.5	0.301	0.047		
751078661	666.831	29.599	-4.2	0.339	0.047		
751078662	667.082	29.606	-3.8	0.399	0.057		
751078663	667.333	29.786	-4.7	0.218	0.018		
751078664	992.463	42.861	16.6	0.084	0.477		
751078665	992.797	42.866	17.1	0.077	0.465		
751078666	993.131	42.886	17.6	0.074	0.46		
751078667	993.465	42.898	19.2	0.058	0.401		
751078668	993.799	42.891	18.7	0.068	0.43		
751078669	994.133	42.887	13.5	0.114	0.606		
751078670	994.47	42.879	8.6	0.304	0.548		
751078671	882.474	37.006	5.9	0.243	0.843		
751078672	882.808	37.019	5.6	0.244	0.828		
751078673	883.143	37.02	5.6	0.252	0.762		
751078674	883.477	37.088	6.4	0.213	0.878		
751078675	883.811	36.972	5.6	0.283	0.853		
751078676	884.147	36.931	2.8	0.621	0.886		
751078677	607.28	20.037	-2.1	0.715	0.45		
751078678	607.53	19.985	-0.8	0.889	0.522		
751078679	607.781	20.011	-1.4	0.807	0.478		
751078680	608.031	20.046	-2	0.718	0.447		
751078681	608.282	20.114	-2.2	0.684	0.396		
751078682	608.532	20.11	-5.9	0.325	0.084		
751078683	434.555	15.933	4.1	0.366	0.648		
751078684	434.889	15.979	3.8	0.304	0.767		
751078685	435.223	15.982	4.6	0.196	0.981		
751078686	435.557	15.985	5.1	0.476	0.731		
751078687	748.852	37.881	5.9	0.214	0.701		
751078688	749.103	37.893	6.5	0.257	0.66		
751078689	749.353	37.892	7.3	0.174	0.712		

751078690	749.604	37.915	6.8	0.224	0.67		
751078691	749.859	37.98	15.7	0.149	0.85		
751078692	1046.788	50.739	2.3	0.671	0.461		
751078693	1046.952	50.737	1.4	0.795	0.396		
751078694	1047.12	50.743	1.5	0.78	0.331		
751078695	1047.284	50.755	1.1	0.834	0.278		
751078696	1047.452	50.785	-3.1	0.566	0.168		
751078697	1047.532	50.8	-0.1	0.984	0.143		
751078698	662.302	23.529	5.7	0.22	0.869		
751078699	662.801	23.517	12.2	0.186	0.984		
751078700	663.305	23.531	6.3	0.174	0.865		
751078701	663.804	23.536	6.7	0.66	0.391		
751078702	814.601	51.404	6.8	0.018	0.546		
751078703	814.719	51.499	7.5	0.009	0.75		
751078704	814.836	51.444	7	0.018	0.691		
751078705	814.954	51.442	6.2	0.043	0.469		
751078706	815.072	51.357	4.5	0.166	0.511		
751078707	815.187	51.325	15.9	0.07	0.454		
751078708	580.769	19.909	-1.1	0.832	0.173		
751078709	581.02	19.905	0	0.996	0.268		
751078710	581.271	19.933	0.4	0.933	0.396		
751078711	581.521	19.893	0.2	0.97	0.279		
751078712	581.771	19.899	0.7	0.88	0.243		
751078713	582.022	19.837	-13.4	0.286	0.127		
751078714	873.946	30.38	14.2	0.22	0.365		
751078715	874.446	30.384	15.2	0.191	0.328		
751078716	874.948	30.378	15.1	0.201	0.346		
751078717	875.448	30.37	15	0.411	0.379		
751078718	558.334	35.88	-0.5	0.934	0.655		
751078719	558.669	35.88	-0.6	0.922	0.685		
751078720	559.003	35.881	-1.2	0.841	0.548		
751078721	559.337	35.887	-32.9	0.294	0.268		
751078722	559.672	35.882	-22.9	0.414	0.936		
751078723	720.861	28.702	6.3	0.284	0.609		
751078724	721.384	28.774	4	0.388	0.187		
751078725	784.901	51.197	13.1	0.012	0.986		
751078726	656.682	37.409	-2.2	0.582	0.071	Tax_Id=9606 Gene_Symbol=APLP1 Isoform 2 of Amyloid-like protein 1	AALEGFLAALQADPPQAER
751078727	657.016	37.412	-2.4	0.557	0.071	Tax_Id=9606 Gene_Symbol=APLP1 Isoform 2 of Amyloid-like protein 1	AALEGFLAALQADPPQAER
751078728	657.351	37.419	-2.5	0.552	0.079	Tax_Id=9606 Gene_Symbol=APLP1 Isoform 2 of Amyloid-like protein 1	AALEGFLAALQADPPQAER
751078729	657.685	37.422	-2.7	0.484	0.045	Tax_Id=9606 Gene_Symbol=APLP1 Isoform 2 of Amyloid-like protein 1	AALEGFLAALQADPPQAER
751078730	658.02	37.412	-5.6	0.175	0.018	Tax_Id=9606 Gene_Symbol=APLP1 Isoform 2 of Amyloid-like protein 1	AALEGFLAALQADPPQAER
751078731	511.643	17.173	-2.2	0.703	0.201		
751078732	511.844	17.175	-2.1	0.713	0.216		
751078733	512.044	17.177	-2.9	0.621	0.166		
751078734	512.245	17.191	-3.3	0.575	0.205		
751078735	512.445	17.188	-3.8	0.52	0.111		
751078736	512.645	17.164	-4.6	0.771	0.412		
751078737	638.053	43.003	7.2	0.191	0.609	Tax_Id=9606 Gene_Symbol=CP Ceruloplasmin	TTIEKPVWLGFLGPIIK
751078738	638.387	43.002	7.4	0.177	0.616	Tax_Id=9606 Gene_Symbol=CP Ceruloplasmin	TTIEKPVWLGFLGPIIK
751078739	638.721	43.001	7.1	0.206	0.571	Tax_Id=9606 Gene_Symbol=CP Ceruloplasmin	TTIEKPVWLGFLGPIIK
751078740	639.056	43.002	7.1	0.457	0.406	Tax_Id=9606 Gene_Symbol=CP Ceruloplasmin	TTIEKPVWLGFLGPIIK
751078741	639.39	43.007	13.8	0.278	0.58	Tax_Id=9606 Gene_Symbol=CP Ceruloplasmin	TTIEKPVWLGFLGPIIK
751078742	639.724	43.017	7.6	0.695	0.602	Tax_Id=9606 Gene_Symbol=CP Ceruloplasmin	TTIEKPVWLGFLGPIIK
751078743	676.32	29.567	5.6	0.487	0.557		
751078744	676.519	29.529	7.7	0.339	0.617		
751078745	676.72	29.53	6.5	0.396	0.602		
751078746	676.92	29.533	11.3	0.242	0.497		
751078747	677.12	29.539	7	0.372	0.428		
751078748	677.326	29.519	3.5	0.635	0.473		
751078749	677.52	29.554	3.7	0.551	0.649		
751078750	677.721	29.565	-4.1	0.827	0.792		
751078751	578.319	17.971	4.3	0.498	0.745	Tax_Id=9606 Gene_Symbol=CFB Isoform 1 of Complement factor B (Fragme	EELLPAQDIK
751078752	578.82	17.972	3.9	0.521	0.667	Tax_Id=9606 Gene_Symbol=CFB Isoform 1 of Complement factor B (Fragme	EELLPAQDIK
751078753	579.322	17.977	5.7	0.364	0.623	Tax_Id=9606 Gene_Symbol=CFB Isoform 1 of Complement factor B (Fragme	EELLPAQDIK
751078754	692.636	50.369	22.7	0.001	0.899		
751078755	692.785	50.361	22.7	0.002	0.842		
751078756	692.935	50.343	37.4	0.021	0.993		
751078757	635.378	15.386	10.2	0.54	0.475		
751078758	636.382	15.385	8.7	0.555	0.438		
751078759	637.386	15.38	0.1	0.993	0.112		
751078760	1191.551	36.345	-1.4	0.739	0.338		
751078761	1191.885	36.364	-0.8	0.847	0.418		
751078762	1192.219	36.366	-0.6	0.875	0.408		
751078763	1192.553	36.361	-0.5	0.904	0.486		
751078764	1192.887	36.355	-0.4	0.925	0.458		
751078765	1193.221	36.339	-0.2	0.97	0.459		
751078766	1193.556	36.336	0.1	0.982	0.67		
751078767	515.997	20.207	-1.3	0.823	0.224		
751078768	516.248	20.204	-1.2	0.831	0.224		
751078769	516.499	20.221	-1.5	0.775	0.209		
751078770	516.749	20.235	-3.5	0.489	0.157		
751078771	517	20.205	-1.6	0.856	0.485		
751078772	677.344	26.56	-1.6	0.541	0.216		
751078773	677.845	26.563	-2.1	0.444	0.238		
751078774	678.347	26.552	-2.3	0.38	0.22		
751078775	678.847	26.556	-4.5	0.164	0.271		
751078776	647.875	24.895	5.7	0.383	0.599		
751078777	648.377	24.897	6.2	0.345	0.566		
751078778	648.878	24.898	5.7	0.395	0.592		
751078779	850.372	29.796	6.9	0.489	0.778		
751078780	850.873	29.797	7.4	0.632	0.528		
751078781	851.373	29.794	3.3	0.809	0.181		
751078782	851.873	29.792	9.6	0.698	0.276		

751078783	852.375	29.797	-11.9	0.526	0.118		
751078784	661.837	34.67	5.8	0.406	0.815		
751078785	662.336	34.666	5.4	0.379	0.994		
751078786	662.84	34.697	5.3	0.48	0.933		
751078787	663.341	34.736	3.7	0.759	0.752		
751078788	548.932	24.966	2.2	0.674	0.69		
751078789	549.267	24.966	2	0.697	0.65		
751078790	549.601	24.967	2.6	0.77	0.747		
751078791	426.539	14.801	1.7	0.821	0.518		
751078792	426.874	14.8	3.1	0.782	0.451		
751078793	427.208	14.798	4.4	0.668	0.51		
751078794	427.542	14.797	-2.7	0.887	0.453		
751078795	1273.653	32.022	6.7	0.435	0.75		
751078796	1273.988	32.029	6.6	0.39	0.811		
751078797	1274.323	32.03	6.2	0.413	0.771		
751078798	1274.657	32.036	6.3	0.396	0.699		
751078799	1274.991	32.038	5.7	0.461	0.719		
751078800	1275.326	32.027	4.4	0.434	0.529		
751078801	1275.662	31.998	6.9	0.446	0.4		
751078802	625.838	28.097	2.1	0.696	0.491	Tax_Id=9606 Gene_Symbol=SERPINF1 Pigment epithelium-derived factor	DTDTGALLFIGK
751078803	626.34	28.031	5.5	0.344	0.815	Tax_Id=9606 Gene_Symbol=SERPINF1 Pigment epithelium-derived factor	DTDTGALLFIGK
751078804	626.842	28.025	5.3	0.438	0.874	Tax_Id=9606 Gene_Symbol=SERPINF1 Pigment epithelium-derived factor	DTDTGALLFIGK
751078805	627.34	27.866	5.7	0.677	0.586	Tax_Id=9606 Gene_Symbol=SERPINF1 Pigment epithelium-derived factor	DTDTGALLFIGK
751078806	575.016	39.464	4.8	0.478	0.518	Tax_Id=9606 Gene_Symbol=AGT Angiotensinogen	VLSALQAVQGLLVAQGR
751078807	575.35	39.463	4.2	0.528	0.462	Tax_Id=9606 Gene_Symbol=AGT Angiotensinogen	VLSALQAVQGLLVAQGR
751078808	575.684	39.463	9.8	0.515	0.51	Tax_Id=9606 Gene_Symbol=AGT Angiotensinogen	VLSALQAVQGLLVAQGR
751078809	576.019	39.46	8.1	0.641	0.322	Tax_Id=9606 Gene_Symbol=AGT Angiotensinogen	VLSALQAVQGLLVAQGR
751078810	576.353	39.463	36.8	0.277	0.579	Tax_Id=9606 Gene_Symbol=AGT Angiotensinogen	VLSALQAVQGLLVAQGR
751078811	753.727	26.854	4.2	0.401	0.854		
751078812	754.73	26.899	1.2	0.756	0.541		
751078813	614.974	18.905	5.9	0.301	0.806		
751078814	615.308	18.931	5.7	0.299	0.849		
751078815	615.642	18.946	4.9	0.325	0.77		
751078816	615.976	18.955	5.1	0.287	0.869		
751078817	616.31	18.989	10.7	0.394	0.969		
751078818	588.282	16.634	9.2	0.168	0.449	Tax_Id=9606 Gene_Symbol=CLU Isoform 2 of Clusterin	EILSVDC[160.0302]STNNPSQAK
751078819	588.617	16.633	9.2	0.171	0.448	Tax_Id=9606 Gene_Symbol=CLU Isoform 2 of Clusterin	EILSVDC[160.0302]STNNPSQAK
751078820	588.951	16.635	8.3	0.222	0.38	Tax_Id=9606 Gene_Symbol=CLU Isoform 2 of Clusterin	EILSVDC[160.0302]STNNPSQAK
751078821	589.285	16.642	9.8	0.171	0.573	Tax_Id=9606 Gene_Symbol=CLU Isoform 2 of Clusterin	EILSVDC[160.0302]STNNPSQAK
751078822	589.619	16.647	24.4	0.562	0.814	Tax_Id=9606 Gene_Symbol=CLU Isoform 2 of Clusterin	EILSVDC[160.0302]STNNPSQAK
751078823	692.586	50.372	22.8	0.002	0.816		
751078824	692.735	50.364	23.4	0.001	0.865		
751078825	692.885	50.336	26.8	0.012	0.659		
751078826	771.416	17.654	6.3	0.406	0.81	Tax_Id=9606 Gene_Symbol=C4A;C4B complement component 4B prepropro	VLSLAQEQVGGSPEK
751078827	771.917	17.653	6.4	0.399	0.807	Tax_Id=9606 Gene_Symbol=C4A;C4B complement component 4B prepropro	VLSLAQEQVGGSPEK
751078828	772.419	17.658	4.2	0.685	0.734	Tax_Id=9606 Gene_Symbol=C4A;C4B complement component 4B prepropro	VLSLAQEQVGGSPEK
751078829	772.92	17.666	-6.1	0.756	0.38	Tax_Id=9606 Gene_Symbol=C4A;C4B complement component 4B prepropro	VLSLAQEQVGGSPEK
751078830	698.957	47.727	10.3	0.122	0.603		
751078831	699.157	47.731	10.2	0.157	0.473		
751078832	699.358	47.729	9.9	0.139	0.47		
751078833	699.558	47.732	9.7	0.127	0.464		
751078834	699.758	47.736	9.3	0.139	0.484		
751078835	699.959	47.738	9.8	0.127	0.429		
751078836	700.159	47.741	10.2	0.255	0.522		
751078837	671.4	18.037	8	0.298	0.755		
751078838	672.404	18.04	3.5	0.63	0.373		
751078839	673.407	18.05	-10.6	0.614	0.221		
751078840	479.91	28.583	-0.7	0.884	0.5	Tax_Id=9606 Gene_Symbol=AGT Angiotensinogen	SLDFTELDVAAEK
751078841	480.245	28.583	-1.4	0.766	0.431	Tax_Id=9606 Gene_Symbol=AGT Angiotensinogen	SLDFTELDVAAEK
751078842	480.579	28.569	-3.7	0.712	0.552	Tax_Id=9606 Gene_Symbol=AGT Angiotensinogen	SLDFTELDVAAEK
751078843	480.913	28.528	-30.3	0.286	0.293	Tax_Id=9606 Gene_Symbol=AGT Angiotensinogen	SLDFTELDVAAEK
751078844	603.815	25.028	-2.5	0.669	0.595		
751078845	604.066	25.026	-1.6	0.79	0.686		
751078846	604.316	25.023	-2.2	0.71	0.664		
751078847	604.567	25.029	-2.7	0.648	0.601		
751078848	604.818	25.017	-2.6	0.838	0.43		
751078849	669.941	46.312	10.5	0.097	0.899		
751078850	457.244	24.57	2.8	0.644	0.447		
751078851	457.746	24.57	3.1	0.636	0.484		
751078852	458.247	24.581	6.4	0.77	0.283		
751078853	634.067	44.766	10	0.094	0.854		
751078854	634.268	44.764	9	0.11	0.77		
751078855	634.468	44.776	9.2	0.1	0.812		
751078856	634.669	44.778	9.8	0.08	0.906		
751078857	744.384	39.483	1.1	0.867	0.234	Tax_Id=9606 Gene_Symbol=CHGB Secretogranin-1	ADQTVLTEDEKKELENLAAMDLELQK
751078858	744.635	39.484	1.5	0.836	0.266	Tax_Id=9606 Gene_Symbol=CHGB Secretogranin-1	ADQTVLTEDEKKELENLAAMDLELQK
751078859	744.886	39.488	1.2	0.879	0.257	Tax_Id=9606 Gene_Symbol=CHGB Secretogranin-1	ADQTVLTEDEKKELENLAAMDLELQK
751078860	745.136	39.498	1.6	0.842	0.267	Tax_Id=9606 Gene_Symbol=CHGB Secretogranin-1	ADQTVLTEDEKKELENLAAMDLELQK
751078861	745.387	39.504	6.3	0.533	0.281	Tax_Id=9606 Gene_Symbol=CHGB Secretogranin-1	ADQTVLTEDEKKELENLAAMDLELQK
751078862	745.635	39.493	3.8	0.681	0.248	Tax_Id=9606 Gene_Symbol=CHGB Secretogranin-1	ADQTVLTEDEKKELENLAAMDLELQK
751078863	731.396	14.849	4.9	0.765	0.455		
751078864	732.399	14.842	3.3	0.882	0.396		
751078865	733.402	14.836	-0.5	0.979	0.218		
751078866	822.472	49.777	4.7	0.467	0.699	Tax_Id=9606 Gene_Symbol=AGT Angiotensinogen	ADSQAQLLLSTVVGVFTAPGLHLK
751078867	822.806	49.778	0.5	0.93	0.271	Tax_Id=9606 Gene_Symbol=AGT Angiotensinogen	ADSQAQLLLSTVVGVFTAPGLHLK
751078868	823.14	49.779	2.6	0.653	0.4	Tax_Id=9606 Gene_Symbol=AGT Angiotensinogen	ADSQAQLLLSTVVGVFTAPGLHLK
751078869	823.475	49.777	-2.4	0.688	0.144	Tax_Id=9606 Gene_Symbol=AGT Angiotensinogen	ADSQAQLLLSTVVGVFTAPGLHLK
751078870	823.81	49.781	-40.8	0.006	0.013	Tax_Id=9606 Gene_Symbol=AGT Angiotensinogen	ADSQAQLLLSTVVGVFTAPGLHLK
751078871	980.49	32.134	3.6	0.48	0.722		
751078872	980.992	32.158	3.3	0.527	0.722		
751078873	981.493	32.177	2.7	0.591	0.617		
751078874	981.994	32.177	2	0.666	0.556		
751078875	982.492	32.123	-4.2	0.448	0.276		

751078876	1031.798	32.624	12.1	0.394	0.93		
751078877	1032.132	32.625	11.5	0.395	0.991		
751078878	1032.466	32.644	10.6	0.443	0.946		
751078879	1032.8	32.67	8.2	0.524	0.908		
751078880	1033.134	32.682	3.7	0.732	0.249		
751078881	1033.467	32.709	2.6	0.802	0.252		
751078882	1033.802	32.681	0.2	0.984	0.22		
751078883	865.515	51.466	6	0.074	0.244		
751078884	865.763	51.556	5.9	0.071	0.545		
751078885	805.628	44.117	8.4	0.354	0.852	Tax_Id=9606 Gene_Symbol=HPX Hemopexin	DGWHSWPIAHQWPQGPSAVDAAFSWEEK
751078886	805.879	44.109	9.4	0.3	0.935	Tax_Id=9606 Gene_Symbol=HPX Hemopexin	DGWHSWPIAHQWPQGPSAVDAAFSWEEK
751078887	806.13	44.12	8.1	0.349	0.881	Tax_Id=9606 Gene_Symbol=HPX Hemopexin	DGWHSWPIAHQWPQGPSAVDAAFSWEEK
751078888	806.381	44.097	10.3	0.237	0.961	Tax_Id=9606 Gene_Symbol=HPX Hemopexin	DGWHSWPIAHQWPQGPSAVDAAFSWEEK
751078889	806.631	44.121	8.5	0.352	0.862	Tax_Id=9606 Gene_Symbol=HPX Hemopexin	DGWHSWPIAHQWPQGPSAVDAAFSWEEK
751078890	806.881	44.09	7.4	0.413	0.735	Tax_Id=9606 Gene_Symbol=HPX Hemopexin	DGWHSWPIAHQWPQGPSAVDAAFSWEEK
751078891	662.775	15.351	9.3	0.508	0.209		
751078892	663.276	15.35	8.6	0.442	0.238		
751078893	663.776	15.351	10	0.536	0.163		
751078894	664.277	15.356	8.9	0.685	0.143		
751078895	691.859	52.211	5.9	0.176	0.869		
751078896	692.009	52.204	8.4	0.032	0.724		
751078897	692.159	52.14	8.5	0.029	0.863		
751078898	812.455	15.616	0.9	0.902	0.366		
751078899	813.458	15.617	0.2	0.975	0.308		
751078900	814.461	15.624	-9.9	0.526	0.052		
751078901	681.851	31.008	7.9	0.325	0.702		
751078902	682.018	31	8.8	0.346	0.689		
751078903	682.185	31.008	9.1	0.347	0.752		
751078904	682.352	31.008	6.4	0.354	0.623		
751078905	682.52	31.018	7	0.373	0.6		
751078906	682.686	31.011	9	0.561	0.515		
751078907	682.854	31.037	1.4	0.899	0.127		
751078908	683.02	30.991	-7.7	0.652	0.156		
751078909	629.389	14.61	4.5	0.567	0.185		
751078910	630.392	14.605	5	0.743	0.142		
751078911	631.396	14.6	-14	0.421	0.035		
751078912	471.288	18.883	3.4	0.646	0.936		
751078913	471.79	18.886	3.8	0.609	0.993		
751078914	472.292	18.878	2.9	0.846	0.559		
751078915	514.805	19.2	0.6	0.911	0.421		
751078916	515.306	19.218	0.7	0.9	0.423		
751078917	515.807	19.204	0	0.998	0.446		
751078918	516.309	19.183	-24.8	0.173	0.251		
751078919	806.902	32.205	2.5	0.581	0.22	Tax_Id=9606 Gene_Symbol=APOA1 Apolipoprotein A-I	LLDNWDSVTSTFSK
751078920	807.404	32.202	3	0.538	0.206	Tax_Id=9606 Gene_Symbol=APOA1 Apolipoprotein A-I	LLDNWDSVTSTFSK
751078921	807.905	32.213	5.3	0.586	0.306	Tax_Id=9606 Gene_Symbol=APOA1 Apolipoprotein A-I	LLDNWDSVTSTFSK
751078922	808.406	32.229	2.2	0.798	0.338	Tax_Id=9606 Gene_Symbol=APOA1 Apolipoprotein A-I	LLDNWDSVTSTFSK
751078923	668.585	31.339	-1.1	0.873	0.367		
751078924	668.836	31.342	-0.7	0.913	0.361		
751078925	669.086	31.344	-0.1	0.988	0.439		
751078926	669.331	31.28	-1.2	0.833	0.443		
751078927	669.587	31.345	-2.3	0.716	0.127		
751078928	669.837	31.333	-8.4	0.475	0.543		
751078929	973.862	31.972	-8.6	0.269	0.101		
751078930	974.062	31.986	-6.1	0.42	0.173		
751078931	974.263	31.989	-6.6	0.368	0.159		
751078932	974.463	32.022	-4.2	0.523	0.345		
751078933	974.664	32.025	-5.8	0.435	0.216		
751078934	974.864	32.053	-8.2	0.232	0.09		
751078935	975.066	32.037	-10.8	0.125	0.052		
751078936	881.922	16.644	9.7	0.189	0.452	Tax_Id=9606 Gene_Symbol=CLU Isoform 2 of Clusterin	EILSVDC[160.0302]STNNPSQAK
751078937	882.423	16.647	9.2	0.204	0.433	Tax_Id=9606 Gene_Symbol=CLU Isoform 2 of Clusterin	EILSVDC[160.0302]STNNPSQAK
751078938	882.924	16.653	9.4	0.202	0.447	Tax_Id=9606 Gene_Symbol=CLU Isoform 2 of Clusterin	EILSVDC[160.0302]STNNPSQAK
751078939	883.425	16.657	2.7	0.799	0.261	Tax_Id=9606 Gene_Symbol=CLU Isoform 2 of Clusterin	EILSVDC[160.0302]STNNPSQAK
751078940	883.926	16.667	52	0.219	0.643	Tax_Id=9606 Gene_Symbol=CLU Isoform 2 of Clusterin	EILSVDC[160.0302]STNNPSQAK
751078941	440.87	16.758	2.6	0.692	0.68	Tax_Id=9606 Gene_Symbol=GSN Isoform 1 of Gelsolin	SEDC[160.0302]FILDHGK
751078942	441.204	16.767	-14.8	0.371	0.717	Tax_Id=9606 Gene_Symbol=GSN Isoform 1 of Gelsolin	SEDC[160.0302]FILDHGK
751078943	441.539	16.762	3.8	0.668	0.547	Tax_Id=9606 Gene_Symbol=GSN Isoform 1 of Gelsolin	SEDC[160.0302]FILDHGK
751078944	441.872	16.768	-25.7	0.391	0.889	Tax_Id=9606 Gene_Symbol=GSN Isoform 1 of Gelsolin	SEDC[160.0302]FILDHGK
751078945	1107.163	21.973	-9.5	0.037	0.004	Tax_Id=9606 Gene_Symbol=CHGA Chromogranin-A	AEGNNQAPGEEEEEEEEATNTHPPASLPSQK
751078946	1107.497	22.027	-9.3	0.037	0.006	Tax_Id=9606 Gene_Symbol=CHGA Chromogranin-A	AEGNNQAPGEEEEEEEEATNTHPPASLPSQK
751078947	1107.831	22.077	-9.4	0.026	0.005	Tax_Id=9606 Gene_Symbol=CHGA Chromogranin-A	AEGNNQAPGEEEEEEEEATNTHPPASLPSQK
751078948	1108.164	22.119	-9.1	0.028	0.005	Tax_Id=9606 Gene_Symbol=CHGA Chromogranin-A	AEGNNQAPGEEEEEEEEATNTHPPASLPSQK
751078949	1108.498	22.16	-8.7	0.027	0.004	Tax_Id=9606 Gene_Symbol=CHGA Chromogranin-A	AEGNNQAPGEEEEEEEEATNTHPPASLPSQK
751078950	1108.831	22.212	-13.3	0.02	0.005	Tax_Id=9606 Gene_Symbol=CHGA Chromogranin-A	AEGNNQAPGEEEEEEEEATNTHPPASLPSQK
751078951	1066.301	47.352	10.2	0.111	0.645		
751078952	1066.547	47.47	9.7	0.109	0.566		
751078953	1066.797	47.545	9	0.098	0.514		
751078954	1067.049	47.341	8.7	0.053	0.399		
751078955	1067.301	47.302	8.9	0.029	0.309		
751078956	976.458	50.726	1.2	0.845	0.907	Tax_Id=9606 Gene_Symbol=DKK3 cDNA FLJ52545, highly similar to Dickko	EVPDEYEVGSFMEEVRQELEDLER
751078957	976.791	50.714	1.8	0.785	0.733	Tax_Id=9606 Gene_Symbol=DKK3 cDNA FLJ52545, highly similar to Dickko	EVPDEYEVGSFMEEVRQELEDLER
751078958	977.127	50.713	2.8	0.707	0.912	Tax_Id=9606 Gene_Symbol=DKK3 cDNA FLJ52545, highly similar to Dickko	EVPDEYEVGSFMEEVRQELEDLER
751078959	977.459	50.717	1	0.871	0.947	Tax_Id=9606 Gene_Symbol=DKK3 cDNA FLJ52545, highly similar to Dickko	EVPDEYEVGSFMEEVRQELEDLER
751078960	977.796	50.74	2.1	0.702	0.973	Tax_Id=9606 Gene_Symbol=DKK3 cDNA FLJ52545, highly similar to Dickko	EVPDEYEVGSFMEEVRQELEDLER
751078961	634.167	44.757	9.4	0.117	0.864		
751078962	634.368	44.753	9.4	0.112	0.883		
751078963	634.568	44.785	7.9	0.142	0.681		
751078964	634.769	44.791	8.4	0.142	0.714		
751078965	634.97	44.75	-3.7	0.673	0.092		
751078966	731.17	52.144	8.3	0.065	0.933		
751078967	731.326	52.195	5.9	0.189	0.699		
751078968	731.485	52.148	7.1	0.067	0.896		

751078969	731.642	51.891	7.6	0.108	0.645		
751078970	669.829	46.315	10.2	0.108	0.988		
751078971	670.052	46.321	9.7	0.12	0.995		
751078972	853.397	36.432	-1.7	0.629	0.207		
751078973	853.648	36.437	-1.7	0.639	0.222		
751078974	853.899	36.43	-1.8	0.623	0.191		
751078975	854.15	36.411	-1.8	0.606	0.215		
751078976	854.399	36.37	-1.7	0.609	0.17		
751078977	854.651	36.42	-0.9	0.826	0.351		
751078978	854.902	36.425	-12.9	0.063	0.076		
751078979	618.351	32.157	6.3	0.137	0.872	Tax_Id=9606 Gene_Symbol=APOA1 Apolipoprotein A-I	DLATVYVDVLK
751078980	618.853	32.156	6.3	0.138	0.883	Tax_Id=9606 Gene_Symbol=APOA1 Apolipoprotein A-I	DLATVYVDVLK
751078981	619.355	32.237	5.1	0.212	0.994	Tax_Id=9606 Gene_Symbol=APOA1 Apolipoprotein A-I	DLATVYVDVLK
751078982	656.352	18.137	6.3	0.38	0.836		
751078983	656.853	18.137	6.1	0.415	0.858		
751078984	657.354	18.114	6	0.434	0.982		
751078985	577.282	18.331	5.4	0.389	0.473		
751078986	577.783	18.334	4.6	0.475	0.426		
751078987	578.285	18.338	3.2	0.563	0.253		
751078988	691.659	52.199	8	0.066	0.821		
751078989	691.809	52.186	6.3	0.116	0.591		
751078990	691.958	52.144	7.5	0.085	0.796		
751078991	692.11	52.134	6.7	0.116	0.758		
751078992	495.263	25.733	11.2	0.132	0.338		
751078993	495.765	25.734	12.2	0.122	0.298		
751078994	496.266	25.743	7.1	0.415	0.738		
751078995	660.355	21.526	1.3	0.69	0.071	Tax_Id=9606 Gene_Symbol=GSN Isoform 1 of Gelsolin	AGALNSNDAFVLK
751078996	660.856	21.515	1	0.748	0.066	Tax_Id=9606 Gene_Symbol=GSN Isoform 1 of Gelsolin	AGALNSNDAFVLK
751078997	661.357	21.519	0.7	0.816	0.049	Tax_Id=9606 Gene_Symbol=GSN Isoform 1 of Gelsolin	AGALNSNDAFVLK
751078998	661.859	21.517	-3.1	0.819	0.775	Tax_Id=9606 Gene_Symbol=GSN Isoform 1 of Gelsolin	AGALNSNDAFVLK
751078999	435.775	15.466	2.5	0.222	0.568		
751079000	436.277	15.464	2.7	0.21	0.615		
751079001	436.778	15.488	2.6	0.604	0.967		
751079002	802.146	33.972	7.7	0.362	0.779		
751079003	802.396	33.973	9.5	0.297	0.881		
751079004	802.647	33.977	8.2	0.365	0.782		
751079005	802.898	34.017	6.8	0.445	0.739		
751079006	803.148	33.992	5.8	0.469	0.696		
751079007	803.402	34.201	4.2	0.389	0.983		
751079008	688.37	27.492	3.1	0.665	0.27		
751079009	689.374	27.46	3.5	0.674	0.304		
751079010	690.377	27.421	-0.6	0.954	0.243		
751079011	489.271	31.221	9.3	0.112	0.513		
751079012	489.773	31.217	10.3	0.108	0.575		
751079013	490.274	31.166	12.3	0.423	0.251		
751079014	769.567	51.503	7.2	0.015	0.488		
751079015	769.678	51.659	6.3	0.042	0.486		
751079016	769.845	51.369	7	0.032	0.337		
751079017	393.224	14.514	0.9	0.837	0.096		
751079018	393.726	14.506	0.6	0.897	0.086		
751079019	394.228	14.496	-0.9	0.942	0.098		
751079020	567.944	15.025	7.5	0.409	0.604		
751079021	568.278	15.026	7.4	0.417	0.579		
751079022	568.612	15.025	6.9	0.771	0.375		
751079023	568.946	15.035	19.6	0.295	0.974		
751079024	886.038	49.975	-8	0.427	0.099		
751079025	886.182	49.972	16.3	0.094	0.799		
751079026	886.325	49.972	16.6	0.089	0.855		
751079027	886.468	49.973	16.2	0.091	0.789		
751079028	886.611	49.972	17.3	0.094	0.696		
751079029	886.754	49.972	18	0.08	0.744		
751079030	886.898	49.973	15.2	0.104	0.946		
751079031	887.041	49.973	16	0.087	0.771		
751079032	887.184	49.974	6.9	0.421	0.398		
751079033	837.626	50.422	2.4	0.683	0.394		
751079034	837.761	50.411	-0.1	0.989	0.304		
751079035	837.892	50.423	0.1	0.986	0.22		
751079036	838.026	50.426	-0.5	0.916	0.115		
751079037	838.158	50.437	-2.7	0.746	0.091		
751079038	785.891	41.493	5.2	0.038	0.297	Tax_Id=9606 Gene_Symbol=TTR Transthyretin	TSESGELHGLTTEEEFVEGIYKVEIDTK
751079039	786.141	41.538	5.3	0.037	0.296	Tax_Id=9606 Gene_Symbol=TTR Transthyretin	TSESGELHGLTTEEEFVEGIYKVEIDTK
751079040	786.392	41.498	4.3	0.086	0.457	Tax_Id=9606 Gene_Symbol=TTR Transthyretin	TSESGELHGLTTEEEFVEGIYKVEIDTK
751079041	786.643	41.495	4.7	0.056	0.358	Tax_Id=9606 Gene_Symbol=TTR Transthyretin	TSESGELHGLTTEEEFVEGIYKVEIDTK
751079042	786.894	41.456	5.4	0.058	0.404	Tax_Id=9606 Gene_Symbol=TTR Transthyretin	TSESGELHGLTTEEEFVEGIYKVEIDTK
751079043	787.145	41.376	5.6	0.063	0.418	Tax_Id=9606 Gene_Symbol=TTR Transthyretin	TSESGELHGLTTEEEFVEGIYKVEIDTK
751079044	787.395	41.284	2.5	0.876	0.345	Tax_Id=9606 Gene_Symbol=TTR Transthyretin	TSESGELHGLTTEEEFVEGIYKVEIDTK
751079045	930.426	36.157	0.1	0.974	0.49		
751079046	930.677	36.178	-0.5	0.893	0.44		
751079047	930.928	36.176	0.1	0.974	0.481		
751079048	931.179	36.175	-0.5	0.892	0.495		
751079049	931.429	36.156	-0.3	0.947	0.508		
751079050	931.679	36.159	-0.3	0.947	0.463		
751079051	931.923	36.118	0.9	0.868	0.626		
751079052	546.63	21.927	1.4	0.783	0.448		
751079053	546.964	21.922	1.2	0.817	0.414		
751079054	547.299	21.92	1.7	0.744	0.509		
751079055	547.633	21.921	0	0.996	0.304		
751079056	769.346	51.398	5.4	0.076	0.299		
751079057	769.457	51.464	5.5	0.053	0.405		
751079058	769.623	51.616	7	0.014	0.527		
751079059	769.734	51.513	6.1	0.047	0.718		
751079060	837.559	50.783	1.3	0.815	0.444		
751079061	487.752	14.725	3.5	0.664	0.595	Tax_Id=9606 Gene_Symbol=CNDP1 Beta-Ala-His dipeptidase	HLEDVFSK

751079062	488.253	14.724	-0.8	0.919	0.392	Tax_Id=9606 Gene_Symbol=CNDP1 Beta-Ala-His dipeptidase	HLEDVFSK
751079063	488.755	14.716	16.9	0.288	0.711	Tax_Id=9606 Gene_Symbol=CNDP1 Beta-Ala-His dipeptidase	HLEDVFSK
751079064	489.257	14.635	-6	0.836	0.555	Tax_Id=9606 Gene_Symbol=CNDP1 Beta-Ala-His dipeptidase	HLEDVFSK
751079065	772.061	49.474	4.1	0.507	0.512		
751079066	772.396	49.481	3.7	0.507	0.461		
751079067	772.721	49.398	1.2	0.81	0.283		
751079068	773.064	49.463	1.3	0.772	0.159		
751079069	773.407	49.384	1.4	0.755	0.174		
751079070	830.901	17.386	-6.4	0.052	0.212	Tax_Id=9606 Gene_Symbol=CST3 Cystatin-C	LVGGPM[147.0355]DASVEEEGVR
751079071	831.403	17.385	-6.9	0.043	0.197	Tax_Id=9606 Gene_Symbol=CST3 Cystatin-C	LVGGPM[147.0355]DASVEEEGVR
751079072	831.904	17.395	-7	0.056	0.25	Tax_Id=9606 Gene_Symbol=CST3 Cystatin-C	LVGGPM[147.0355]DASVEEEGVR
751079073	832.406	17.396	-9.9	0.614	0.941	Tax_Id=9606 Gene_Symbol=CST3 Cystatin-C	LVGGPM[147.0355]DASVEEEGVR
751079074	713.889	17.401	6.3	0.329	0.842		
751079075	714.391	17.447	5.5	0.387	0.896		
751079076	714.892	17.418	5.4	0.422	0.964		
751079077	715.395	17.448	-0.3	0.98	0.671		
751079078	785.151	50.621	-1.7	0.777	0.401		
751079079	785.275	50.712	0.8	0.889	0.514		
751079080	785.401	50.717	0.2	0.964	0.369		
751079081	785.525	50.778	1.1	0.815	0.464		
751079082	785.65	50.745	4.2	0.457	0.767		
751079083	785.775	50.773	1.7	0.738	0.635		
751079084	785.901	50.705	5.9	0.258	0.802		
751079085	617.104	49.781	0.7	0.896	0.277	Tax_Id=9606 Gene_Symbol=AGT Angiotensinogen	ADSQAQLLLSTVVGVFTAPGLHLK
751079086	617.355	49.782	7.2	0.332	0.99	Tax_Id=9606 Gene_Symbol=AGT Angiotensinogen	ADSQAQLLLSTVVGVFTAPGLHLK
751079087	617.605	49.782	-1.9	0.715	0.152	Tax_Id=9606 Gene_Symbol=AGT Angiotensinogen	ADSQAQLLLSTVVGVFTAPGLHLK
751079088	617.856	49.781	-0.4	0.966	0.472	Tax_Id=9606 Gene_Symbol=AGT Angiotensinogen	ADSQAQLLLSTVVGVFTAPGLHLK
751079089	618.107	49.787	29.9	0.1	0.619	Tax_Id=9606 Gene_Symbol=AGT Angiotensinogen	ADSQAQLLLSTVVGVFTAPGLHLK
751079090	755.433	15.513	0	0.998	0.155		
751079091	756.436	15.516	-1.7	0.934	0.3		
751079092	757.438	15.548	-4.4	0.829	0.351		
751079093	431.742	15.422	12.2	0.144	0.862		
751079094	431.993	15.422	13.5	0.129	0.921		
751079095	432.244	15.424	14.3	0.106	0.98		
751079096	432.495	15.423	250.5	0.012	0.133		
751079097	1046.101	27.311	-7.3	0.069	0.027		
751079098	1046.436	27.315	-7.5	0.069	0.036		
751079099	1046.769	27.323	-7.4	0.069	0.038		
751079100	1047.103	27.341	-6.9	0.087	0.044		
751079101	1047.443	27.299	-6.9	0.1	0.067		
751079102	1047.775	27.431	-6.3	0.101	0.015		
751079103	435.769	16.485	8.6	0.039	0.66	Tax_Id=9606 Gene_Symbol=A1BG Alpha-1B-glycoprotein	LLELTGPK
751079104	436.271	16.549	9.5	0.133	0.935	Tax_Id=9606 Gene_Symbol=A1BG Alpha-1B-glycoprotein	LLELTGPK
751079105	436.772	16.585	13.7	0.517	0.671	Tax_Id=9606 Gene_Symbol=A1BG Alpha-1B-glycoprotein	LLELTGPK
751079106	518.26	17.627	13.5	0.141	0.197		
751079107	518.762	17.649	14.3	0.125	0.172		
751079108	519.263	17.671	7.1	0.461	0.966		
751079109	825.366	23.175	3.9	0.7	0.238	Tax_Id=9606 Gene_Symbol=CP Ceruloplasmin	MFTTAPDQVDKEDEDFQESNK
751079110	825.7	23.175	4	0.695	0.268	Tax_Id=9606 Gene_Symbol=CP Ceruloplasmin	MFTTAPDQVDKEDEDFQESNK
751079111	826.034	23.184	4.2	0.677	0.26	Tax_Id=9606 Gene_Symbol=CP Ceruloplasmin	MFTTAPDQVDKEDEDFQESNK
751079112	826.369	23.192	4.3	0.65	0.265	Tax_Id=9606 Gene_Symbol=CP Ceruloplasmin	MFTTAPDQVDKEDEDFQESNK
751079113	826.697	23.198	6.5	0.727	0.371	Tax_Id=9606 Gene_Symbol=CP Ceruloplasmin	MFTTAPDQVDKEDEDFQESNK
751079114	827.037	23.221	-7.2	0.742	0.116	Tax_Id=9606 Gene_Symbol=CP Ceruloplasmin	MFTTAPDQVDKEDEDFQESNK
751079115	724.364	23.821	7.2	0.138	0.759		
751079116	724.699	23.826	7.5	0.132	0.749		
751079117	725.034	23.827	7.8	0.127	0.719		
751079118	725.366	23.815	9.4	0.101	0.674		
751079119	725.701	23.845	2.6	0.524	0.493		
751079120	785.338	50.715	0.8	0.888	0.44		
751079121	785.463	50.722	0.8	0.88	0.355		
751079122	785.587	50.782	0.1	0.985	0.448		
751079123	785.712	50.78	-0.5	0.929	0.298		
751079124	785.837	50.76	0.7	0.889	0.329		
751079125	795.139	39.879	13	0.158	0.975		
751079126	795.263	39.899	14.2	0.143	0.992		
751079127	795.389	39.884	12.4	0.179	0.994		
751079128	795.514	39.896	14.3	0.136	0.953		
751079129	795.639	39.897	12.8	0.133	0.849		
751079130	795.764	39.904	9.4	0.189	0.638		
751079131	795.889	39.894	9.9	0.214	0.548		
751079132	796.014	39.806	4.5	0.589	0.277		
751079133	1079.059	34.835	6.5	0.302	0.54	Tax_Id=9606 Gene_Symbol=C3 Complement C3 (Fragment)	ILLQGTPVAQMTEDAVDAER
751079134	1079.561	34.838	6.3	0.297	0.508	Tax_Id=9606 Gene_Symbol=C3 Complement C3 (Fragment)	ILLQGTPVAQMTEDAVDAER
751079135	1080.061	34.849	7.1	0.293	0.572	Tax_Id=9606 Gene_Symbol=C3 Complement C3 (Fragment)	ILLQGTPVAQMTEDAVDAER
751079136	1080.559	34.866	7.7	0.238	0.901	Tax_Id=9606 Gene_Symbol=C3 Complement C3 (Fragment)	ILLQGTPVAQMTEDAVDAER
751079137	1081.065	34.874	2.1	0.813	0.245	Tax_Id=9606 Gene_Symbol=C3 Complement C3 (Fragment)	ILLQGTPVAQMTEDAVDAER
751079138	762.336	14.663	3	0.899	0.296		
751079139	763.346	14.667	2.5	0.912	0.304		
751079140	764.351	14.721	1.1	0.952	0.163		
751079141	854.475	51.868	-3.1	0.65	0.092	Tax_Id=9606 Gene_Symbol=AGT Angiotensinogen	TIHLTMPQLVLQGSYDLQDLLAQAELPAILHTELN
751079142	854.674	51.856	0.4	0.957	0.248	Tax_Id=9606 Gene_Symbol=AGT Angiotensinogen	TIHLTMPQLVLQGSYDLQDLLAQAELPAILHTELN
751079143	854.877	51.879	0.5	0.937	0.198	Tax_Id=9606 Gene_Symbol=AGT Angiotensinogen	TIHLTMPQLVLQGSYDLQDLLAQAELPAILHTELN
751079144	855.08	51.897	2.2	0.748	0.347	Tax_Id=9606 Gene_Symbol=AGT Angiotensinogen	TIHLTMPQLVLQGSYDLQDLLAQAELPAILHTELN
751079145	855.278	51.865	-1.3	0.848	0.142	Tax_Id=9606 Gene_Symbol=AGT Angiotensinogen	TIHLTMPQLVLQGSYDLQDLLAQAELPAILHTELN
751079146	855.477	51.852	-8.7	0.474	0.038	Tax_Id=9606 Gene_Symbol=AGT Angiotensinogen	TIHLTMPQLVLQGSYDLQDLLAQAELPAILHTELN
751079147	693.324	18.544	5.2	0.367	0.429		
751079148	693.825	18.541	4.2	0.427	0.506		
751079149	694.326	18.484	-9.7	0.168	0.154		
751079150	600.626	18.827	0.5	0.917	0.259		
751079151	600.962	18.556	2.3	0.626	0.533		
751079152	691.632	49.404	-4.2	0.441	0.051	Tax_Id=9606 Gene_Symbol=SCG3 Secretogranin-3	LLNLGLITESQAHTLEDEVAEVLQK
751079153	691.882	49.401	-1.7	0.756	0.079	Tax_Id=9606 Gene_Symbol=SCG3 Secretogranin-3	LLNLGLITESQAHTLEDEVAEVLQK
751079154	692.132	49.402	-2.1	0.701	0.073	Tax_Id=9606 Gene_Symbol=SCG3 Secretogranin-3	LLNLGLITESQAHTLEDEVAEVLQK

751079155	692.383	49.405	-4.8	0.402	0.038	Tax_Id=9606 Gene_Symbol=SCG3 Secretogranin-3	LLNLGLITESQAHTLEDEVAEVLQK
751079156	692.634	49.406	-3.9	0.692	0.071	Tax_Id=9606 Gene_Symbol=SCG3 Secretogranin-3	LLNLGLITESQAHTLEDEVAEVLQK
751079157	380.451	14.937	5.2	0.5	0.477	Tax_Id=9606 Gene_Symbol=B2M Beta-2-microglobulin	IEKVEHSDLSFSK
751079158	380.702	14.937	8.9	0.67	0.363	Tax_Id=9606 Gene_Symbol=B2M Beta-2-microglobulin	IEKVEHSDLSFSK
751079159	380.953	14.937	8.2	0.704	0.404	Tax_Id=9606 Gene_Symbol=B2M Beta-2-microglobulin	IEKVEHSDLSFSK
751079160	381.203	14.921	7.3	0.635	0.271	Tax_Id=9606 Gene_Symbol=B2M Beta-2-microglobulin	IEKVEHSDLSFSK
751079161	521.784	24.818	8	0.041	0.558		
751079162	522.286	24.801	7.9	0.062	0.613		
751079163	522.787	24.804	15.9	0.096	0.591		
751079164	1157.594	49.498	1.5	0.792	0.347		
751079165	1158.095	49.504	2.2	0.694	0.432		
751079166	1158.597	49.515	3.5	0.581	0.646		
751079167	1159.098	49.505	2.4	0.689	0.479		
751079168	1159.601	49.487	0.5	0.92	0.608		
751079169	754.396	26.877	2.1	0.62	0.582		
751079170	495.272	66.121	0.7	0.929	0.458		
751079171	496.275	66.113	1.4	0.849	0.393		
751079172	497.278	66.095	-24.1	0.409	0.923		
751079173	817.64	41.377	3.1	0.586	0.704	Tax_Id=9606 Gene_Symbol=PTGDS Prostaglandin-H2 D-isomerase	SPHWGSTYSVSVVETDYDQYALLYSQGSK
751079174	817.891	41.413	3.5	0.573	0.661	Tax_Id=9606 Gene_Symbol=PTGDS Prostaglandin-H2 D-isomerase	SPHWGSTYSVSVVETDYDQYALLYSQGSK
751079175	818.141	41.415	3.7	0.556	0.695	Tax_Id=9606 Gene_Symbol=PTGDS Prostaglandin-H2 D-isomerase	SPHWGSTYSVSVVETDYDQYALLYSQGSK
751079176	818.392	41.408	3.5	0.58	0.677	Tax_Id=9606 Gene_Symbol=PTGDS Prostaglandin-H2 D-isomerase	SPHWGSTYSVSVVETDYDQYALLYSQGSK
751079177	818.643	41.378	1.9	0.756	0.563	Tax_Id=9606 Gene_Symbol=PTGDS Prostaglandin-H2 D-isomerase	SPHWGSTYSVSVVETDYDQYALLYSQGSK
751079178	818.893	41.1	-0.6	0.926	0.45	Tax_Id=9606 Gene_Symbol=PTGDS Prostaglandin-H2 D-isomerase	SPHWGSTYSVSVVETDYDQYALLYSQGSK
751079179	469.947	28.554	3.4	0.301	0.142		
751079180	470.281	28.552	3.1	0.327	0.1		
751079181	470.615	28.545	2.9	0.391	0.12		
751079182	470.95	28.473	24.4	0.106	0.397		
751079183	837.559	50.415	3.3	0.721	0.223		
751079184	837.693	50.423	-1.4	0.8	0.136		
751079185	837.827	50.421	0.3	0.96	0.316		
751079186	837.959	50.426	0.5	0.926	0.339		
751079187	838.092	50.432	1.3	0.817	0.489		
751079188	645.312	29.309	0.9	0.845	0.105	Tax_Id=9606 Gene_Symbol=APP Isoform L-APP733 of Amyloid beta A4 prot	STNLHDYGMLLPC[160.0302]GIDK
751079189	645.648	29.293	0.9	0.841	0.126	Tax_Id=9606 Gene_Symbol=APP Isoform L-APP733 of Amyloid beta A4 prot	STNLHDYGMLLPC[160.0302]GIDK
751079190	645.981	29.298	1.4	0.769	0.127	Tax_Id=9606 Gene_Symbol=APP Isoform L-APP733 of Amyloid beta A4 prot	STNLHDYGMLLPC[160.0302]GIDK
751079191	646.314	29.337	13.6	0.41	0.529	Tax_Id=9606 Gene_Symbol=APP Isoform L-APP733 of Amyloid beta A4 prot	STNLHDYGMLLPC[160.0302]GIDK
751079192	646.648	29.346	20.1	0.359	0.417	Tax_Id=9606 Gene_Symbol=APP Isoform L-APP733 of Amyloid beta A4 prot	STNLHDYGMLLPC[160.0302]GIDK
751079193	618.974	27.621	2.3	0.686	0.287		
751079194	619.307	27.584	1.6	0.783	0.244		
751079195	619.643	27.601	1.2	0.847	0.244		
751079196	619.977	27.608	-0.6	0.95	0.068		
751079197	620.311	27.591	8.2	0.666	0.281		
751079198	694.338	35.908	-1	0.89	0.26		
751079199	694.672	35.905	-0.7	0.923	0.279		
751079200	695.006	35.904	-0.6	0.939	0.108		
751079201	695.339	35.922	0.5	0.952	0.493		
751079202	695.673	35.915	-6	0.465	0.054		
751079203	647.299	36.138	5.1	0.605	0.718		
751079204	647.8	36.156	1.1	0.912	0.214		
751079205	648.303	36.121	5.8	0.512	0.916		
751079206	648.802	36.161	5.7	0.532	0.944		
751079207	1079.131	37.398	3	0.331	0.352		
751079208	1079.465	37.39	3.7	0.234	0.405		
751079209	1079.8	37.395	3.3	0.302	0.34		
751079210	1080.134	37.398	2.9	0.379	0.279		
751079211	1080.468	37.385	2.5	0.425	0.291		
751079212	1080.802	37.292	2.9	0.397	0.379		
751079213	1081.136	37.121	0.4	0.9	0.345		
751079214	581.965	29.018	6.5	0.2	0.748		
751079215	582.3	29.017	6.3	0.205	0.702		
751079216	582.634	29.04	6.6	0.21	0.771		
751079217	582.968	29.049	11	0.439	0.534		
751079218	856.903	26.862	6.9	0.627	0.419		
751079219	857.152	26.88	8.2	0.355	0.625		
751079220	857.403	26.881	8.1	0.355	0.654		
751079221	857.653	26.884	4.6	0.56	0.195		
751079222	857.904	26.896	6.1	0.43	0.555		
751079223	858.154	26.855	2.7	0.766	0.159		
751079224	858.407	26.954	1	0.87	0.12		
751079225	670.336	24.524	0.3	0.922	0.923		
751079226	670.837	24.529	-0.4	0.907	0.886		
751079227	671.338	24.537	-0.6	0.849	0.845		
751079228	671.836	24.585	-18.3	0.405	0.765		
751079229	1067.046	47.937	9.8	0.013	0.312		
751079230	1067.295	47.903	10.3	0.009	0.42		
751079231	1067.548	47.683	10.6	0.01	0.474		
751079232	1067.798	47.681	10.5	0.022	0.427		
751079233	1068.048	47.702	7.2	0.354	0.348		
751079234	659.701	50.383	23.1	0.001	0.925		
751079235	659.843	50.393	22.7	0.001	0.969		
751079236	659.986	50.339	18.1	0.091	0.322		
751079237	695.849	16.472	3.1	0.75	0.778		
751079238	696.35	16.475	-0.3	0.978	0.908		
751079239	696.852	16.474	-28.7	0.247	0.384		
751079240	479.288	16.82	14	0.163	0.847		
751079241	480.291	16.824	12.1	0.595	0.594		
751079242	682.882	47.293	9.5	0.143	0.801		
751079243	683.384	47.3	10.5	0.134	0.854		
751079244	683.883	47.332	8.9	0.072	0.711		
751079245	684.382	47.363	5.2	0.316	0.842		
751079246	415.908	16.907	3.4	0.378	0.305		
751079247	416.242	16.902	2.9	0.465	0.242		

751079248	416.576	16.905	0.2	0.973	0.191		
751079249	416.911	16.878	-10.6	0.669	0.046		
751079250	613.56	30.407	2.6	0.537	0.554		
751079251	613.811	30.374	2.2	0.58	0.525		
751079252	614.062	30.389	2.5	0.547	0.464		
751079253	614.312	30.053	2.4	0.532	0.569		
751079254	608.296	15.436	5.2	0.426	0.886		
751079255	609.299	15.449	3.1	0.672	0.439		
751079256	610.297	15.522	-7	0.715	0.146		
751079257	755.594	47.64	10.7	0.202	0.933		
751079258	755.762	47.638	12	0.148	0.959		
751079259	755.929	47.64	11.4	0.159	0.965		
751079260	756.096	47.644	12	0.149	0.985		
751079261	756.263	47.647	11.7	0.148	1		
751079262	756.43	47.64	11.2	0.153	0.974		
751079263	756.597	47.635	11.6	0.145	0.641		
751079264	756.766	47.615	7.3	0.337	0.858		
751079265	1175.565	46.495	4.2	0.613	0.561	Tax_Id=9606 Gene_Symbol=APOA2 Apolipoprotein A-II	EPC[160.0302]VESLVSQYFQTVTDYGK
751079266	1176.066	46.493	3.2	0.69	0.533	Tax_Id=9606 Gene_Symbol=APOA2 Apolipoprotein A-II	EPC[160.0302]VESLVSQYFQTVTDYGK
751079267	1176.567	46.464	2.5	0.72	0.437	Tax_Id=9606 Gene_Symbol=APOA2 Apolipoprotein A-II	EPC[160.0302]VESLVSQYFQTVTDYGK
751079268	1177.066	46.505	1.1	0.859	0.344	Tax_Id=9606 Gene_Symbol=APOA2 Apolipoprotein A-II	EPC[160.0302]VESLVSQYFQTVTDYGK
751079269	1177.569	46.44	7.8	0.238	0.863	Tax_Id=9606 Gene_Symbol=APOA2 Apolipoprotein A-II	EPC[160.0302]VESLVSQYFQTVTDYGK
751079270	629.668	50.371	20.9	0.002	0.728		
751079271	629.805	50.378	22.2	0.002	0.905		
751079272	629.941	50.349	19.8	0.027	0.717		
751079273	1342.942	35.749	3.2	0.432	0.656		
751079274	1343.276	35.76	2.8	0.503	0.668		
751079275	1343.61	35.767	2.7	0.527	0.627		
751079276	1343.945	35.764	2	0.633	0.559		
751079277	1344.28	35.759	3.2	0.44	0.76		
751079278	1344.615	35.78	2.3	0.581	0.565		
751079279	1344.949	35.725	1.4	0.752	0.395		
751079280	659.653	50.355	22	0.002	0.949		
751079281	659.795	50.354	24.7	0.001	0.986		
751079282	659.938	50.358	20.4	0.002	0.568		
751079283	898.531	50.689	-3.2	0.341	0.007		
751079284	898.672	50.693	-3.1	0.382	0.008		
751079285	898.814	50.694	-3.9	0.274	0.011		
751079286	898.957	50.698	-4.4	0.175	0.001		
751079287	613.56	28.571	48.1	0.013	0.503		
751079288	613.811	28.577	99.5	0.065	0.371		
751079289	614.061	28.571	57.3	0.068	0.94		
751079290	614.312	28.574	47.9	0.036	0.918		
751079291	614.563	28.561	248.3	0.071	0.234		
751079292	842.376	29.017	2.4	0.631	0.729		
751079293	842.626	29.018	2.2	0.667	0.768		
751079294	842.877	29.025	2.2	0.653	0.667		
751079295	843.127	29.03	1.3	0.735	0.352		
751079296	843.377	29.039	1.6	0.694	0.554		
751079297	843.628	29.051	0.1	0.975	0.422		
751079298	843.878	29.065	0.2	0.987	0.486		
751079299	844.129	29.027	-25.1	0.238	0.28		
751079300	472.783	17.728	9.7	0.199	0.963		
751079301	473.286	17.734	10.1	0.238	0.949		
751079302	473.787	17.732	3.1	0.874	0.242		
751079303	718.685	18.996	7.5	0.356	0.377		
751079304	719.019	19.008	7.4	0.341	0.382		
751079305	719.353	19.021	6.2	0.467	0.363		
751079306	719.688	19.05	-0.7	0.979	0.658		
751079307	720.022	19.102	4.6	0.897	0.357		
751079308	425.72	22.112	8.9	0.17	0.641		
751079309	426.222	22.113	11.4	0.127	0.643		
751079310	426.723	22.152	6.7	0.22	0.563		
751079311	830.623	21.964	-9	0.042	0.003		
751079312	830.875	22.018	-8.6	0.08	0.022		
751079313	831.123	22.058	-9.1	0.046	0.008		
751079314	831.375	22.13	-9.5	0.033	0.009		
751079315	831.624	22.131	-9.5	0.104	0.019		
751079316	831.877	22.17	-12	0.033	0.035		
751079317	986.542	22.664	1.2	0.908	0.154		
751079318	987.544	22.663	5.9	0.803	0.386		
751079319	988.547	22.671	-1.6	0.923	0.054		
751079320	489.937	16.141	4.4	0.358	0.656		
751079321	490.271	16.232	3.7	0.426	0.624		
751079322	490.605	16.139	4.2	0.396	0.738		
751079323	490.939	16.143	46	0.081	0.484		
751079324	934.426	36.127	0.5	0.898	0.814		
751079325	934.677	36.121	0.5	0.897	0.84		
751079326	934.927	36.117	1.1	0.78	0.855		
751079327	935.178	36.112	0.8	0.842	0.815		
751079328	935.429	36.098	0.3	0.941	0.728		
751079329	935.679	36.098	0.6	0.884	0.926		
751079330	935.93	36.108	-2.2	0.847	0.8		
751079331	936.178	36.134	-20.1	0.195	0.322		
751079332	1245.161	39.675	10.2	0.141	0.974	Tax_Id=9606 Gene_Symbol=TTR Transthyretin	YTIAALLSPYSYSTTAVVTNPKE
751079333	1245.663	39.676	9.6	0.128	0.957	Tax_Id=9606 Gene_Symbol=TTR Transthyretin	YTIAALLSPYSYSTTAVVTNPKE
751079334	1246.164	39.675	9.8	0.121	0.966	Tax_Id=9606 Gene_Symbol=TTR Transthyretin	YTIAALLSPYSYSTTAVVTNPKE
751079335	1246.665	39.684	10.2	0.144	0.915	Tax_Id=9606 Gene_Symbol=TTR Transthyretin	YTIAALLSPYSYSTTAVVTNPKE
751079336	1247.166	39.681	10.7	0.414	0.691	Tax_Id=9606 Gene_Symbol=TTR Transthyretin	YTIAALLSPYSYSTTAVVTNPKE
751079337	1037.996	23.676	-13.1	0.003	0.001	Tax_Id=9606 Gene_Symbol=CHGA Chromogranin-A	YPGPQAEGDSEGLSQGLVDR
751079338	1038.498	23.689	-11.2	0.009	0.002	Tax_Id=9606 Gene_Symbol=CHGA Chromogranin-A	YPGPQAEGDSEGLSQGLVDR
751079339	1038.999	23.712	-46.5	0.085	0.197	Tax_Id=9606 Gene_Symbol=CHGA Chromogranin-A	YPGPQAEGDSEGLSQGLVDR
751079340	1039.5	23.727	-44	0.054	0.129	Tax_Id=9606 Gene_Symbol=CHGA Chromogranin-A	YPGPQAEGDSEGLSQGLVDR

751079341	1040.002	23.764	-44.7	0.036	0.128	Tax_Id=9606 Gene_Symbol=CHGA Chromogranin-A	YPGPQAEGDSEGLSQGLVDR
751079342	689.93	28.209	9.5	0.313	0.729		
751079343	690.43	28.177	9	0.32	0.668		
751079344	690.931	28.163	9.5	0.316	0.621		
751079345	836.938	33.797	6.7	0.298	0.94		
751079346	837.439	33.798	7.7	0.243	0.986		
751079347	837.941	33.799	7.2	0.269	0.971		
751079348	838.442	33.808	9.4	0.275	0.938		
751079349	723.08	22.835	-3.4	0.311	0.069		
751079350	723.33	22.843	-2.9	0.419	0.1		
751079351	723.581	22.852	-3.5	0.327	0.065		
751079352	723.832	22.866	-2.2	0.642	0.325		
751079353	724.08	22.857	-2.5	0.53	0.19		
751079354	724.329	22.807	-3.3	0.445	0.127		
751079355	471.26	23.34	9.5	0.143	0.182		
751079356	471.762	23.34	10.5	0.106	0.143		
751079357	472.263	23.347	10.8	0.11	0.368		
751079358	634.886	23.736	1.5	0.771	0.224	Tax_Id=9606 Gene_Symbol=AGT Angiotensinogen	ALQDQLVLVAAK
751079359	635.387	23.734	1.2	0.808	0.199	Tax_Id=9606 Gene_Symbol=AGT Angiotensinogen	ALQDQLVLVAAK
751079360	635.889	23.718	1.4	0.781	0.187	Tax_Id=9606 Gene_Symbol=AGT Angiotensinogen	ALQDQLVLVAAK
751079361	636.39	23.7	-14.6	0.5	0.537	Tax_Id=9606 Gene_Symbol=AGT Angiotensinogen	ALQDQLVLVAAK
751079362	898.531	50.227	-7.7	0.178	0.018		
751079363	898.673	50.227	-4.8	0.407	0.093		
751079364	898.814	50.226	-10.5	0.105	0.034		
751079365	898.958	50.242	-6.9	0.243	0.058		
751079366	899.1	50.242	-7	0.47	0.177		
751079367	949.558	38.96	4.2	0.584	0.401	Tax_Id=9606 Gene_Symbol=AGT Angiotensinogen	QPFVQGLALYTPVVLPR
751079368	950.059	38.959	2.9	0.527	0.134	Tax_Id=9606 Gene_Symbol=AGT Angiotensinogen	QPFVQGLALYTPVVLPR
751079369	950.56	38.961	7.2	0.629	0.456	Tax_Id=9606 Gene_Symbol=AGT Angiotensinogen	QPFVQGLALYTPVVLPR
751079370	951.062	38.964	5.4	0.505	0.488	Tax_Id=9606 Gene_Symbol=AGT Angiotensinogen	QPFVQGLALYTPVVLPR
751079371	951.565	38.969	1.8	0.908	0.659	Tax_Id=9606 Gene_Symbol=AGT Angiotensinogen	QPFVQGLALYTPVVLPR
751079372	629.714	50.377	19.9	0.001	0.604		
751079373	629.85	50.367	22.6	0.001	0.994		
751079374	629.987	50.35	20.8	0	0.751		
751079375	561.244	18.283	19.8	0.079	0.797		
751079376	561.745	18.292	1.1	0.934	0.048		
751079377	562.245	18.364	5.8	0.523	0.42		
751079378	963.486	32.137	3.3	0.518	0.699		
751079379	963.99	32.176	3.3	0.513	0.67		
751079380	964.487	32.211	3.2	0.508	0.664		
751079381	964.993	32.143	2.1	0.55	0.529		
751079382	965.483	32.099	2.7	0.813	0.405		
751079383	872.447	29.011	7.7	0.439	0.469	Tax_Id=9606 Gene_Symbol=PTGDS Prostaglandin-H2 D-isomerase	TMLLQPAGSLGSYSYR
751079384	872.949	29.012	6.1	0.334	0.533	Tax_Id=9606 Gene_Symbol=PTGDS Prostaglandin-H2 D-isomerase	TMLLQPAGSLGSYSYR
751079385	642.321	32.132	3	0.527	0.749		
751079386	642.658	32.16	3.4	0.46	0.763		
751079387	643.011	32.18	0.2	0.949	0.265		
751079388	651.337	15.848	6.6	0.334	0.932		
751079389	652.34	15.867	2.8	0.677	0.722		
751079390	653.343	15.866	6.9	0.762	0.786		
751079391	755.405	31.543	9.6	0.1	0.87		
751079392	755.906	31.544	10.1	0.113	0.797		
751079393	756.408	31.509	11.3	0.102	0.696		
751079394	1274.902	40.188	3.9	0.221	0.775		
751079395	1275.241	40.281	4	0.205	0.82		
751079396	1275.576	40.301	3.8	0.233	0.813		
751079397	1275.911	40.305	3.5	0.274	0.792		
751079398	1276.246	40.294	4.2	0.172	0.846		
751079399	1276.58	40.289	4.2	0.208	0.882		
751079400	1276.914	40.147	3.5	0.35	0.787		
751079401	860.838	35.631	11.9	0.441	0.487		
751079402	861.038	35.631	8.5	0.198	0.759		
751079403	861.239	35.633	12.8	0.597	0.466		
751079404	861.439	35.629	9.3	0.117	0.773		
751079405	861.64	35.636	7	0.415	0.544		
751079406	861.84	35.635	12.5	0.418	0.573		
751079407	862.041	35.641	9.4	0.458	0.883		
751079408	662.319	24.179	-0.7	0.909	0.381		
751079409	662.655	24.125	-1.4	0.785	0.444		
751079410	662.99	24.117	-2.3	0.632	0.351		
751079411	663.323	24.184	0.2	0.975	0.507		
751079412	663.658	24.145	-21.7	0.01	0.006		
751079413	762.054	28.403	-7.6	0.134	0.153		
751079414	762.388	28.403	-7.2	0.154	0.157		
751079415	762.722	28.402	-7.4	0.144	0.123		
751079416	763.057	28.401	-7.9	0.168	0.165		
751079417	763.391	28.433	-39.4	0.015	0.039		
751079418	652.667	30.57	-6.5	0.377	0.321	Tax_Id=9606 Gene_Symbol=SERPINF1 Pigment epithelium-derived factor	ALYYDLISSPDIHGTYK
751079419	653.001	30.569	-8.8	0.243	0.219	Tax_Id=9606 Gene_Symbol=SERPINF1 Pigment epithelium-derived factor	ALYYDLISSPDIHGTYK
751079420	653.336	30.562	-6.2	0.464	0.335	Tax_Id=9606 Gene_Symbol=SERPINF1 Pigment epithelium-derived factor	ALYYDLISSPDIHGTYK
751079421	653.67	30.571	-7.8	0.588	0.724	Tax_Id=9606 Gene_Symbol=SERPINF1 Pigment epithelium-derived factor	ALYYDLISSPDIHGTYK
751079422	703.031	25.998	-4.6	0.57	0.341		
751079423	703.365	25.996	-5.1	0.53	0.3		
751079424	703.7	26.004	-5	0.525	0.346		
751079425	704.034	26.014	-6.7	0.397	0.22		
751079426	704.368	25.999	-5.7	0.371	0.169		
751079427	704.408	44.759	9	0.127	0.821		
751079428	704.63	44.756	8.8	0.119	0.889		
751079429	704.854	44.814	8.6	0.132	0.88		
751079430	705.076	44.769	8	0.122	0.8		
751079431	606.724	16.027	-10	0.104	0.295		
751079432	607.226	16.028	-24.9	0.099	0.238		
751079433	607.726	16.029	-23.4	0.31	0.638		

751079434	817.875	19.888	16.3	0.047	0.247		
751079435	818.376	19.874	15.4	0.042	0.264		
751079436	818.878	19.877	15.5	0.045	0.239		
751079437	819.379	19.886	9.8	0.341	0.468		
751079438	572.923	25.292	12	0.219	0.259		
751079439	573.257	25.281	17.4	0.297	0.489		
751079440	573.591	25.296	18.3	0.46	0.366		
751079441	573.925	25.302	28.9	0.186	0.349		
751079442	574.258	25.297	42.8	0.134	0.915		
751079443	765.399	22.86	5.5	0.437	0.829		
751079444	765.898	22.872	5.5	0.434	0.846		
751079445	766.395	22.827	4.2	0.563	0.934		
751079446	766.892	22.783	3.7	0.549	0.706		
751079447	670.164	46.33	10.1	0.127	0.944		
751079448	621.253	11.258	3.1	0.853	0.211		
751079449	622.256	11.257	8	0.593	0.211		
751079450	623.259	11.258	8.4	0.577	0.188		
751079451	827.429	30.195	4.7	0.405	0.942		
751079452	827.93	30.218	5.2	0.393	0.902		
751079453	828.431	30.239	6.3	0.296	0.776		
751079454	828.933	30.214	-7	0.528	0.531		
751079455	753.182	46.332	10.5	0.074	0.861		
751079456	753.307	46.36	10.8	0.085	0.965		
751079457	753.433	46.373	10.1	0.082	0.827		
751079458	753.558	46.383	10.4	0.074	0.942		
751079459	753.683	46.16	10.5	0.069	0.983		
751079460	753.809	46.4	9.8	0.081	0.823		
751079461	753.934	46.408	8.2	0.137	0.768		
751079462	754.06	46.143	11.4	0.051	0.994		
751079463	754.185	46.353	10.4	0.026	0.674		
751079464	754.311	46.133	16.9	0.102	0.757		
751079465	1141.771	50.719	1.5	0.813	0.53		
751079466	1141.954	50.741	0.7	0.916	0.26		
751079467	1142.131	50.735	0.6	0.916	0.304		
751079468	1142.222	50.741	0.8	0.901	0.518		
751079469	1142.312	50.752	3.8	0.559	0.715		
751079470	1142.403	50.756	4.5	0.484	0.711		
751079471	1142.495	50.77	-0.7	0.899	0.24		
751079472	1142.586	50.789	1.4	0.824	0.357		
751079473	523.287	16.653	16	0.086	0.97		
751079474	523.622	16.653	19.2	0.065	0.848		
751079475	523.956	16.656	21.6	0.073	0.868		
751079476	524.298	16.671	3.5	0.636	0.567		
751079477	1190.931	18.758	4.2	0.729	0.18		
751079478	1191.265	18.76	9.5	0.703	0.471		
751079479	1191.601	18.736	4.2	0.725	0.317		
751079480	1191.933	18.775	5.7	0.809	0.42		
751079481	1192.268	18.794	2.4	0.812	0.227		
751079482	1192.605	18.722	0.4	0.964	0.288		
751079483	423.564	16.104	6.4	0.432	0.876	Tax_Id=9606 Gene_Symbol=HPX Hemopexin	FDPVRGEVPPR
751079484	423.898	16.092	7	0.42	0.934	Tax_Id=9606 Gene_Symbol=HPX Hemopexin	FDPVRGEVPPR
751079485	424.232	16.042	2.6	0.843	0.32	Tax_Id=9606 Gene_Symbol=HPX Hemopexin	FDPVRGEVPPR
751079486	524.758	15.099	1.4	0.796	0.389		
751079487	525.259	15.107	1.1	0.846	0.319		
751079488	525.761	15.123	-0.9	0.925	0.193		
751079489	1275.247	35.794	2.5	0.553	0.52		
751079490	1275.582	35.806	3.5	0.401	0.752		
751079491	1275.916	35.806	2.9	0.492	0.638		
751079492	1276.25	35.801	3.2	0.455	0.686		
751079493	1276.584	35.794	3.3	0.471	0.676		
751079494	1276.919	35.8	2.1	0.608	0.455		
751079495	1277.255	35.794	5.5	0.196	0.843		
751079496	465.239	34.552	2.2	0.672	0.658		
751079497	465.573	34.554	2.7	0.618	0.695		
751079498	465.908	34.559	2.8	0.878	0.512		
751079499	466.242	34.57	14.3	0.681	0.945		
751079500	898.459	50.232	-8.2	0.162	0.026		
751079501	898.603	50.226	-7.2	0.215	0.049		
751079502	898.743	50.226	-8	0.152	0.044		
751079503	898.887	50.231	-7.7	0.181	0.043		
751079504	899.028	50.245	-2.3	0.757	0.243		
751079505	898.602	50.748	-2.7	0.416	0.007		
751079506	898.743	50.688	-3	0.36	0.006		
751079507	898.886	50.681	-3.2	0.342	0.002		
751079508	899.028	50.695	-0.9	0.793	0.017		
751079509	426.537	17.157	-2.2	0.721	0.192		
751079510	426.704	17.156	-3.2	0.601	0.165		
751079511	426.871	17.158	-2.8	0.649	0.16		
751079512	427.038	17.159	-4.8	0.435	0.106		
751079513	427.205	17.143	-1.7	0.797	0.206		
751079514	427.372	17.132	-23.6	0.255	0.314		
751079515	644.353	29.005	5.5	0.17	0.76		
751079516	644.604	29.027	5.6	0.182	0.8		
751079517	644.858	29.039	5.5	0.173	0.734		
751079518	645.106	29.027	5.5	0.159	0.88		
751079519	645.364	29.097	5.5	0.32	0.649		
751079520	1314.239	34.401	13.2	0.097	0.774		
751079521	1314.573	34.388	12.7	0.108	0.808		
751079522	1314.907	34.383	12.8	0.102	0.764		
751079523	1315.241	34.389	12.2	0.112	0.82		
751079524	1315.576	34.397	11.7	0.101	0.857		
751079525	1315.91	34.408	10	0.155	0.984		
751079526	1316.248	34.424	10	0.113	0.884		

751079527	1316.587	34.35	16.1	0.463	0.71		
751079528	772.838	49.274	1.7	0.801	0.566		
751079529	772.981	49.302	1.3	0.84	0.591		
751079530	773.125	49.322	1.8	0.759	0.51		
751079531	773.267	49.333	2	0.729	0.613		
751079532	645.272	16.198	10.9	0.148	0.577		
751079533	646.278	16.228	8	0.3	0.788		
751079534	581.332	22.062	1.5	0.768	0.437		
751079535	581.834	22.065	1.4	0.78	0.444		
751079536	582.335	22.066	2.4	0.907	0.464		
751079537	1046.87	51.25	15.6	0.007	0.587		
751079538	411.719	18.312	11.6	0.062	0.77		
751079539	412.221	18.315	19.5	0.081	0.67		
751079540	412.723	18.304	47.9	0.171	0.401		
751079541	587.813	24.166	9.2	0.185	0.548	Tax_Id=9606 Gene_Symbol=NFASC Isoform 7 of Neurofascin	DLELTDLAER
751079542	588.315	24.148	9.2	0.136	0.928	Tax_Id=9606 Gene_Symbol=NFASC Isoform 7 of Neurofascin	DLELTDLAER
751079543	588.816	24.173	13.8	0.573	0.796	Tax_Id=9606 Gene_Symbol=NFASC Isoform 7 of Neurofascin	DLELTDLAER
751079544	704.52	44.882	9	0.143	0.945		
751079545	704.742	44.776	9	0.123	0.885		
751079546	704.965	44.789	8.4	0.127	0.804		
751079547	705.188	44.806	8.8	0.099	0.868		
751079548	692.535	50.366	21.3	0.002	0.726		
751079549	692.685	50.356	21	0.004	0.69		
751079550	692.835	50.366	21.6	0.002	0.805		
751079551	606.302	16.178	-6.1	0.428	0.753		
751079552	606.637	16.183	-4.8	0.267	0.352		
751079553	606.971	16.186	-5.7	0.696	0.966		
751079554	607.305	16.173	-18.5	0.138	0.313		
751079555	601.297	18.58	3	0.509	0.598		
751079556	601.633	18.59	2.4	0.582	0.493		
751079557	601.967	18.621	3.1	0.503	0.505		
751079558	551.953	30.19	4.2	0.43	0.941		
751079559	552.287	30.199	3.3	0.513	0.826		
751079560	552.621	30.213	2	0.754	0.541		
751079561	552.956	30.213	-1.4	0.825	0.365		
751079562	1146.154	41.412	3.1	0.721	0.452		
751079563	1146.354	41.414	5.4	0.561	0.623		
751079564	1146.555	41.413	4.7	0.601	0.557		
751079565	1146.755	41.413	6.8	0.492	0.612		
751079566	1146.955	41.417	8.4	0.443	0.68		
751079567	1147.156	41.421	6	0.537	0.51		
751079568	1147.357	41.42	3.6	0.687	0.328		
751079569	1147.559	41.418	5.6	0.544	0.64		
751079570	498.33	15.663	1.5	0.843	0.336		
751079571	499.333	15.664	7.5	0.711	0.406		
751079572	713.355	23.041	-22.7	0.198	0.262		
751079573	713.692	22.853	0.4	0.933	0.421		
751079574	714.026	22.895	1	0.84	0.449		
751079575	714.359	22.958	2.8	0.648	0.539		
751079576	714.692	22.989	6.3	0.653	0.98		
751079577	774.1	32.671	4.9	0.63	0.261		
751079578	774.351	32.684	11.9	0.251	0.954		
751079579	774.602	32.678	11.9	0.249	0.929		
751079580	774.852	32.69	4.5	0.683	0.242		
751079581	775.102	32.697	7.4	0.51	0.753		
751079582	775.352	32.707	4.2	0.619	0.186		
751079583	792.402	31.605	6.3	0.389	0.509	Tax_Id=9606 Gene_Symbol=GSN Isoform 1 of Gelsolin	VHVSEEGTEPEAMLQVLGPKPALPAGTEDTAKE
751079584	792.602	31.626	8.9	0.286	0.695	Tax_Id=9606 Gene_Symbol=GSN Isoform 1 of Gelsolin	VHVSEEGTEPEAMLQVLGPKPALPAGTEDTAKE
751079585	792.803	31.635	8.3	0.288	0.688	Tax_Id=9606 Gene_Symbol=GSN Isoform 1 of Gelsolin	VHVSEEGTEPEAMLQVLGPKPALPAGTEDTAKE
751079586	793.003	31.63	10.4	0.517	0.547	Tax_Id=9606 Gene_Symbol=GSN Isoform 1 of Gelsolin	VHVSEEGTEPEAMLQVLGPKPALPAGTEDTAKE
751079587	793.204	31.629	6	0.653	0.208	Tax_Id=9606 Gene_Symbol=GSN Isoform 1 of Gelsolin	VHVSEEGTEPEAMLQVLGPKPALPAGTEDTAKE
751079588	793.406	31.599	7.2	0.584	0.305	Tax_Id=9606 Gene_Symbol=GSN Isoform 1 of Gelsolin	VHVSEEGTEPEAMLQVLGPKPALPAGTEDTAKE
751079589	793.606	31.558	1.4	0.962	0.755	Tax_Id=9606 Gene_Symbol=GSN Isoform 1 of Gelsolin	VHVSEEGTEPEAMLQVLGPKPALPAGTEDTAKE
751079590	478.735	18.286	3	0.785	0.184		
751079591	478.986	18.281	0.8	0.952	0.292		
751079592	479.237	18.281	3.3	0.834	0.143		
751079593	479.488	18.281	-11.7	0.613	0.058		
751079594	479.739	18.29	-45.4	0.043	0.087		
751079595	695.012	31.264	4.2	0.253	0.988		
751079596	695.346	31.262	4.4	0.241	0.93		
751079597	695.681	31.266	3.7	0.316	0.915		
751079598	696.017	31.28	3.7	0.105	0.534		
751079599	696.354	31.295	-6.4	0.555	0.695		
751079600	837.851	16.35	-3.1	0.846	0.237		
751079601	838.353	16.35	-2.7	0.862	0.236		
751079602	838.854	16.353	-3.3	0.814	0.182		
751079603	839.356	16.362	-11.7	0.461	0.027		
751079604	649.365	24.139	-2.3	0.628	0.017	Tax_Id=9606 Gene_Symbol=AGT Angiotensinogen	DPTFIPAPIQAK
751079605	649.866	24.137	-1.5	0.735	0.017	Tax_Id=9606 Gene_Symbol=AGT Angiotensinogen	DPTFIPAPIQAK
751079606	650.368	24.14	-2.3	0.635	0.027	Tax_Id=9606 Gene_Symbol=AGT Angiotensinogen	DPTFIPAPIQAK
751079607	969.815	44.205	-1	0.851	0.057		
751079608	970.149	44.21	-0.9	0.864	0.079		
751079609	970.483	44.212	-1.4	0.788	0.063		
751079610	970.817	44.21	-1.6	0.872	0.13		
751079611	971.151	44.211	-1.6	0.754	0.051		
751079612	971.484	44.211	-3.4	0.697	0.091		
751079613	971.823	44.206	-3.6	0.749	0.145		
751079614	883.209	40.325	15.6	0.113	0.819		
751079615	883.459	40.322	12.1	0.122	0.704		
751079616	883.711	40.333	11	0.121	0.583		
751079617	883.96	40.34	10	0.163	0.537		
751079618	884.216	40.367	5.1	0.298	0.134		
751079619	884.459	40.308	4.1	0.338	0.129		

751079620	884.721	40.391	4.1	0.326	0.368		
751079621	468.015	17.197	-5.8	0.016	0.023		
751079622	468.216	17.194	-6	0.011	0.01		
751079623	468.416	17.195	-6	0.011	0.01		
751079624	468.617	17.193	-6.9	0.008	0.038		
751079625	468.817	17.189	-23.3	0.037	0.089		
751079626	725.037	27.476	-1.5	0.609	0.685		
751079627	725.371	27.483	-1.6	0.598	0.575		
751079628	725.708	27.45	-1.9	0.548	0.667		
751079629	726.039	27.502	3	0.802	0.422		
751079630	731.86	35.344	8.1	0.641	0.533	Tax_Id=9606 Gene_Symbol=ENPP2 Isoform 1 of Ectonucleotide pyrophosph	NGVNVISGPIFDYDYDGLHDTEDKIK
751079631	732.11	35.337	0.7	0.948	0.394	Tax_Id=9606 Gene_Symbol=ENPP2 Isoform 1 of Ectonucleotide pyrophosph	NGVNVISGPIFDYDYDGLHDTEDKIK
751079632	732.361	35.327	8.4	0.627	0.555	Tax_Id=9606 Gene_Symbol=ENPP2 Isoform 1 of Ectonucleotide pyrophosph	NGVNVISGPIFDYDYDGLHDTEDKIK
751079633	732.612	35.342	7.6	0.746	0.434	Tax_Id=9606 Gene_Symbol=ENPP2 Isoform 1 of Ectonucleotide pyrophosph	NGVNVISGPIFDYDYDGLHDTEDKIK
751079634	732.862	35.34	-4.6	0.667	0.351	Tax_Id=9606 Gene_Symbol=ENPP2 Isoform 1 of Ectonucleotide pyrophosph	NGVNVISGPIFDYDYDGLHDTEDKIK
751079635	1001.007	32.154	6.5	0.275	0.87		
751079636	1001.209	32.169	8.9	0.166	0.807		
751079637	1001.41	32.177	7.7	0.191	0.843		
751079638	1001.61	32.187	7.2	0.195	0.862		
751079639	1001.81	32.197	5.3	0.278	0.89		
751079640	1002.011	32.212	5.5	0.269	0.976		
751079641	1002.211	32.217	5.2	0.278	0.931		
751079642	1002.412	32.236	4.6	0.346	0.748		
751079643	1002.613	32.242	-3.6	0.769	0.59		
751079644	760.354	28.156	1.8	0.741	0.664		
751079645	760.608	28.174	3.3	0.604	0.839		
751079646	760.859	28.217	1.8	0.758	0.671		
751079647	761.109	28.172	3.3	0.608	0.723		
751079648	761.36	28.19	-0.4	0.958	0.102		
751079649	761.61	28.158	-2.5	0.816	0.142		
751079650	761.861	28.228	-7.1	0.255	0.087		
751079651	744.341	15.422	4.4	0.781	0.362	Tax_Id=9606 Gene_Symbol=DKK3 cDNA FLJ52545, highly similar to Dickko	EVEELMEDTQHK
751079652	744.843	15.42	3.7	0.716	0.172	Tax_Id=9606 Gene_Symbol=DKK3 cDNA FLJ52545, highly similar to Dickko	EVEELMEDTQHK
751079653	745.343	15.428	2.7	0.802	0.128	Tax_Id=9606 Gene_Symbol=DKK3 cDNA FLJ52545, highly similar to Dickko	EVEELMEDTQHK
751079654	745.845	15.428	-39.4	0.031	0.005	Tax_Id=9606 Gene_Symbol=DKK3 cDNA FLJ52545, highly similar to Dickko	EVEELMEDTQHK
751079655	484.781	14.324	4.7	0.419	0.947	Tax_Id=9606 Gene_Symbol=APOE Apolipoprotein E	LGPLVEQGR
751079656	485.282	14.323	5.2	0.387	0.902	Tax_Id=9606 Gene_Symbol=APOE Apolipoprotein E	LGPLVEQGR
751079657	485.784	14.32	34.8	0.395	0.386	Tax_Id=9606 Gene_Symbol=APOE Apolipoprotein E	LGPLVEQGR
751079658	486.281	14.279	66.4	0.013	0.354	Tax_Id=9606 Gene_Symbol=APOE Apolipoprotein E	LGPLVEQGR
751079659	439.243	17.508	-3.9	0.483	0.166		
751079660	439.577	17.511	-4.2	0.447	0.149		
751079661	439.912	17.521	-4.3	0.543	0.043		
751079662	618.821	28.379	8.9	0.542	0.567		
751079663	619.316	28.404	6	0.352	0.743		
751079664	619.824	28.377	7.3	0.73	0.377		
751079665	620.319	28.455	5.4	0.639	0.993		
751079666	610.82	20.819	1.8	0.788	0.998		
751079667	611.321	20.819	1.1	0.876	0.907		
751079668	611.823	20.829	4.2	0.621	0.801		
751079669	612.314	20.894	1.7	0.931	0.347		
751079670	900.386	50.482	4.8	0.509	0.61		
751079671	900.527	50.483	4.2	0.545	0.563		
751079672	900.672	50.495	1.7	0.772	0.353		
751079673	900.815	50.488	2	0.721	0.268		
751079674	900.957	50.488	-0.2	0.969	0.255		
751079675	901.099	50.479	-4.4	0.563	0.089		
751079676	1288.582	40.562	1	0.794	0.29		
751079677	1288.918	40.613	1	0.797	0.326		
751079678	1289.253	40.57	1.2	0.761	0.325		
751079679	1289.584	40.569	1.4	0.709	0.318		
751079680	1289.92	40.564	1.1	0.768	0.272		
751079681	1290.255	40.543	0.5	0.902	0.252		
751079682	1290.59	40.441	1.8	0.666	0.39		
751079683	900.939	18.84	1.8	0.771	0.411		
751079684	901.444	18.821	1.8	0.771	0.409		
751079685	901.946	18.83	0.9	0.869	0.301		
751079686	902.448	18.848	-0.6	0.877	0.12		
751079687	902.951	18.827	-3.4	0.807	0.957		
751079688	865.931	21.968	1.5	0.816	0.301	Tax_Id=9606 Gene_Symbol=APOE Apolipoprotein E	SELEEQLTPVAEETR
751079689	866.432	21.974	2.6	0.701	0.374	Tax_Id=9606 Gene_Symbol=APOE Apolipoprotein E	SELEEQLTPVAEETR
751079690	866.934	21.965	0.9	0.878	0.284	Tax_Id=9606 Gene_Symbol=APOE Apolipoprotein E	SELEEQLTPVAEETR
751079691	867.44	21.987	-2.1	0.913	0.448	Tax_Id=9606 Gene_Symbol=APOE Apolipoprotein E	SELEEQLTPVAEETR
751079692	824.41	27.707	13.9	0.102	0.928		
751079693	825.414	27.714	14.1	0.119	0.79		
751079694	826.416	27.685	2.8	0.902	0.128		
751079695	508.28	15.836	9.4	0.147	0.857	Tax_Id=9606 Gene_Symbol=CFB Isoform 1 of Complement factor B (Fragme	DISEVVTPR
751079696	508.778	15.783	10.3	0.18	0.739	Tax_Id=9606 Gene_Symbol=CFB Isoform 1 of Complement factor B (Fragme	DISEVVTPR
751079697	509.283	15.837	11.3	0.44	0.55	Tax_Id=9606 Gene_Symbol=CFB Isoform 1 of Complement factor B (Fragme	DISEVVTPR
751079698	886.746	31.309	-13.6	0.087	0.044		
751079699	887.08	31.306	-13.7	0.078	0.037		
751079700	887.413	31.326	-11.3	0.054	0.031		
751079701	887.748	31.325	-11.4	0.109	0.012		
751079702	888.082	31.327	-50	0.016	0.038		
751079703	675.963	14.688	-1.1	0.806	0.602		
751079704	676.297	14.706	-0.9	0.825	0.563		
751079705	676.631	14.73	-0.7	0.867	0.676		
751079706	676.964	14.767	-10.9	0.172	0.281		
751079707	677.298	14.732	-2.9	0.886	0.784		
751079708	769.402	51.427	6.7	0.016	0.361		
751079709	578.852	15.193	1.7	0.734	0.485	Tax_Id=9606 Gene_Symbol=APOA2 Apolipoprotein A-II	SKEQLTPLIK
751079710	579.353	15.192	-0.9	0.863	0.322	Tax_Id=9606 Gene_Symbol=APOA2 Apolipoprotein A-II	SKEQLTPLIK
751079711	579.855	15.197	18	0.152	0.693	Tax_Id=9606 Gene_Symbol=APOA2 Apolipoprotein A-II	SKEQLTPLIK
751079712	687.612	18.302	-17.4	0.001	0.001		

751079713	687.862	18.301	-15.8	0.002	0.002		
751079714	688.113	18.306	-16.2	0.001	0.001		
751079715	688.365	18.323	-20.7	0	0		
751079716	688.614	18.312	-60.8	0	0		
751079717	1046.871	50.407	1.6	0.803	0.549		
751079718	1047.03	50.258	1.1	0.837	0.376		
751079719	1047.203	50.411	2	0.744	0.501		
751079720	1047.37	50.435	-3.7	0.598	0.168		
751079721	1047.455	50.437	2.7	0.758	0.387		
751079722	939.637	31.347	13.4	0.595	0.617		
751079723	939.887	31.348	0.2	0.985	0.087		
751079724	940.138	31.354	0.1	0.993	0.082		
751079725	940.388	31.349	-0.2	0.986	0.109		
751079726	940.638	31.357	5.2	0.823	0.297		
751079727	940.888	31.362	-3.3	0.777	0.058		
751079728	941.14	31.362	0	0.998	0.331		
751079729	967.495	50.204	-8.8	0.105	0.013		
751079730	967.651	50.21	-5.9	0.324	0.083		
751079731	967.801	50.211	-7.6	0.191	0.044		
751079732	967.957	50.212	-7.1	0.213	0.032		
751079733	968.109	50.228	-7.5	0.342	0.24		
751079734	1046.781	50.315	1.6	0.776	0.577		
751079735	1046.953	50.41	-0.5	0.933	0.298		
751079736	1047.119	50.413	1.8	0.731	0.346		
751079737	1047.286	50.426	1.9	0.833	0.157		
751079738	721.353	28.702	5.9	0.29	0.36		
751079739	721.875	28.75	4.1	0.455	0.124		
751079740	722.378	28.742	4.2	0.417	0.135		
751079741	722.878	28.768	-0.9	0.929	0.268		
751079742	635.336	38.879	3.4	0.666	0.178		
751079743	635.67	38.879	3.8	0.827	0.207		
751079744	636.004	38.877	2.5	0.855	0.063		
751079745	636.338	38.871	1.7	0.87	0.063		
751079746	636.672	38.874	13	0.553	0.159		
751079747	813.477	23.381	5.5	0.548	0.733		
751079748	814.48	23.399	6.8	0.677	0.738		
751079749	415.907	17.819	4.3	0.419	0.239		
751079750	416.242	17.824	2.4	0.646	0.104		
751079751	416.576	17.818	18.7	0.177	0.68		
751079752	416.911	17.826	-7.8	0.773	0.157		
751079753	745.013	27.048	-6.7	0.261	0.107		
751079754	745.344	27.098	-6.3	0.263	0.117		
751079755	745.672	27.101	-11.2	0.052	0.037		
751079756	746.002	27.119	-28.5	0.021	0.05		
751079757	653.994	32.132	3	0.536	0.755		
751079758	654.328	32.149	4	0.435	0.848		
751079759	654.663	32.149	3.1	0.558	0.722		
751079760	654.995	32.138	1.2	0.807	0.518		
751079761	1007.461	35.76	0.9	0.834	0.655		
751079762	1007.707	35.751	-0.1	0.975	0.472		
751079763	1007.958	35.76	0.6	0.9	0.542		
751079764	1008.21	35.756	-0.3	0.952	0.608		
751079765	1008.463	35.787	-0.2	0.96	0.483		
751079766	1008.71	35.736	1	0.812	0.719		
751079767	1008.961	35.724	2.3	0.896	0.703		
751079768	713.028	22.61	0.4	0.944	0.629	Tax_Id=9606 Gene_Symbol=A2M Alpha-2-macroglobulin	HNVYINGITYTPVSSTNEK
751079769	713.362	22.55	-4.7	0.548	0.581	Tax_Id=9606 Gene_Symbol=A2M Alpha-2-macroglobulin	HNVYINGITYTPVSSTNEK
751079770	553.301	23.374	7.6	0.246	0.643		
751079771	553.802	23.358	8	0.263	0.66		
751079772	554.303	23.371	6.8	0.494	0.572		
751079773	796.394	33.849	5.4	0.509	0.616	Tax_Id=9606 Gene_Symbol=GSN Isoform 1 of Gelsolin	DPDQTDGLGLSYLSSHIANVER
751079774	796.728	33.854	2.7	0.757	0.425	Tax_Id=9606 Gene_Symbol=GSN Isoform 1 of Gelsolin	DPDQTDGLGLSYLSSHIANVER
751079775	797.062	33.852	2.7	0.748	0.382	Tax_Id=9606 Gene_Symbol=GSN Isoform 1 of Gelsolin	DPDQTDGLGLSYLSSHIANVER
751079776	797.393	33.726	0.9	0.84	0.255	Tax_Id=9606 Gene_Symbol=GSN Isoform 1 of Gelsolin	DPDQTDGLGLSYLSSHIANVER
751079777	797.731	33.852	5.1	0.724	0.648	Tax_Id=9606 Gene_Symbol=GSN Isoform 1 of Gelsolin	DPDQTDGLGLSYLSSHIANVER
751079778	697.857	22.597	4	0.508	0.998		
751079779	698.86	22.595	3.1	0.628	0.905		
751079780	967.648	50.681	-4.4	0.298	0.013		
751079781	967.803	50.679	-3.1	0.37	0.005		
751079782	967.954	50.7	-1.4	0.687	0.005		
751079783	687.831	15.396	-0.1	0.995	0.247	Tax_Id=9606 Gene_Symbol=APP Isoform L-APP733 of Amyloid beta A4 prot	VESLEQEAANER
751079784	688.333	15.395	-0.5	0.961	0.207	Tax_Id=9606 Gene_Symbol=APP Isoform L-APP733 of Amyloid beta A4 prot	VESLEQEAANER
751079785	688.834	15.404	3.4	0.894	0.957	Tax_Id=9606 Gene_Symbol=APP Isoform L-APP733 of Amyloid beta A4 prot	VESLEQEAANER
751079786	500.294	16.576	9.2	0.305	0.397		
751079787	500.545	16.576	9	0.308	0.388		
751079788	500.796	16.576	8	0.328	0.507		
751079789	501.046	16.578	9.9	0.469	0.867		
751079790	501.297	16.583	-12.6	0.622	0.211		
751079791	728.857	28.74	5.7	0.359	0.237		
751079792	729.358	28.718	6.7	0.297	0.343		
751079793	729.864	28.735	5.4	0.398	0.38		
751079794	887.367	20.265	2.5	0.67	0.65		
751079795	887.869	20.272	1.7	0.762	0.591		
751079796	888.369	20.279	2.7	0.681	0.686		
751079797	888.871	20.292	-11.4	0.127	0.067		
751079798	889.376	20.328	2.4	0.926	0.924		
751079799	1063.629	51.88	9.9	0.009	0.591		
751079800	723.854	28.74	5.6	0.534	0.231		
751079801	724.355	28.747	4.1	0.405	0.218		
751079802	724.857	28.747	6.3	0.603	0.398		
751079803	552.294	24.381	1.9	0.774	0.266		
751079804	552.798	24.382	2.5	0.734	0.275		
751079805	553.298	24.363	0.3	0.968	0.391		

751079806	802.098	31.166	4.5	0.338	0.424		
751079807	802.433	31.187	3.9	0.428	0.459		
751079808	802.767	31.169	3.3	0.482	0.382		
751079809	803.101	31.168	6.4	0.442	0.648		
751079810	803.438	31.188	-36.8	0.038	0.055		
751079811	527.764	15.922	1.2	0.87	0.554		
751079812	528.268	15.93	1.3	0.874	0.51		
751079813	528.768	15.934	-8.5	0.638	0.199		
751079814	622.641	22.99	-0.5	0.946	0.605		
751079815	622.975	22.992	-0.8	0.907	0.522		
751079816	623.309	23.03	-0.1	0.989	0.396		
751079817	623.643	23.007	3.2	0.695	0.529		
751079818	875.423	20.282	-1.9	0.883	0.083		
751079819	876.427	20.284	-2.7	0.896	0.125		
751079820	877.426	20.296	-7.8	0.629	0.036		
751079821	683.348	35.882	2.8	0.662	0.57		
751079822	683.494	35.912	7.2	0.665	0.599		
751079823	683.637	35.91	5.6	0.73	0.564		
751079824	683.78	35.905	3.3	0.688	0.604		
751079825	683.923	35.914	2	0.766	0.484		
751079826	684.066	35.925	3.9	0.591	0.352		
751079827	684.209	35.927	2.1	0.658	0.288		
751079828	684.351	35.862	1.5	0.852	0.715		
751079829	423.704	16.41	-22.8	0.232	0.328		
751079830	424.206	16.403	-0.6	0.968	0.446		
751079831	501.77	19.235	-2.3	0.618	0.842		
751079832	502.272	19.243	-2.9	0.556	0.77		
751079833	667.555	16.357	1.9	0.934	0.26		
751079834	667.806	16.364	2.1	0.894	0.249		
751079835	668.057	16.365	1.8	0.909	0.228		
751079836	668.308	16.363	-1	0.961	0.733		
751079837	668.558	16.374	33.2	0.392	0.846		
751079838	592.337	24.581	3.6	0.714	0.78		
751079839	593.341	24.505	5.3	0.821	0.602		
751079840	783.58	66.056	3.8	0.654	0.409		
751079841	784.583	66.085	4.3	0.596	0.385		
751079842	785.587	66.049	2.1	0.784	0.469		
751079843	476.22	15.778	6.5	0.342	0.642		
751079844	476.421	15.776	8.6	0.21	0.469		
751079845	476.621	15.777	8.2	0.238	0.449		
751079846	476.822	15.78	7	0.297	0.626		
751079847	477.022	15.79	-4.3	0.596	0.318		
751079848	644.655	32.17	2.1	0.464	0.466		
751079849	644.989	32.175	2.3	0.405	0.422		
751079850	645.323	32.118	2.2	0.458	0.382		
751079851	645.658	32.129	-0.4	0.886	0.218		
751079852	901.453	23.326	9.8	0.301	0.319		
751079853	901.956	23.337	10.3	0.287	0.326		
751079854	902.457	23.329	9.6	0.326	0.329		
751079855	902.957	23.345	-11.2	0.35	0.608		
751079856	515.786	25.804	7.2	0.615	0.324		
751079857	516.286	25.814	6.3	0.395	0.729		
751079858	516.789	25.819	4.2	0.794	0.111		
751079859	656.345	33.311	3.4	0.715	0.648		
751079860	657.348	33.271	6.3	0.682	0.563		
751079861	428.743	15.328	1.6	0.644	0.164		
751079862	429.245	15.327	3.7	0.437	0.61		
751079863	522.916	14.543	8.2	0.612	0.406	Tax_Id=9606 Gene_Symbol=CP Ceruloplasmin	VNKDDEEFIESNK
751079864	523.25	14.542	8.5	0.719	0.379	Tax_Id=9606 Gene_Symbol=CP Ceruloplasmin	VNKDDEEFIESNK
751079865	523.587	14.573	8.9	0.669	0.384	Tax_Id=9606 Gene_Symbol=CP Ceruloplasmin	VNKDDEEFIESNK
751079866	523.924	14.598	9.8	0.504	0.621	Tax_Id=9606 Gene_Symbol=CP Ceruloplasmin	VNKDDEEFIESNK
751079867	965.09	47.608	5.4	0.323	0.514		
751079868	965.192	47.609	5.7	0.309	0.772		
751079869	965.293	47.608	6.1	0.244	0.85		
751079870	965.392	47.608	5.8	0.281	0.757		
751079871	965.491	47.611	6.6	0.183	0.862		
751079872	965.591	47.615	5.9	0.294	0.78		
751079873	965.691	47.617	5.9	0.214	0.808		
751079874	965.79	47.618	5.8	0.232	0.743		
751079875	965.99	47.626	9.1	0.269	0.43		
751079876	1136.561	35.652	3.9	0.528	0.194	Tax_Id=9606 Gene_Symbol=GSN Isoform 1 of Gelsolin	AQPVQVAEGSEPDGFWEALGGK
751079877	1137.061	35.647	5.9	0.447	0.428	Tax_Id=9606 Gene_Symbol=GSN Isoform 1 of Gelsolin	AQPVQVAEGSEPDGFWEALGGK
751079878	1137.559	35.524	0.1	0.973	0.916	Tax_Id=9606 Gene_Symbol=GSN Isoform 1 of Gelsolin	AQPVQVAEGSEPDGFWEALGGK
751079879	1138.063	35.646	6.4	0.28	0.967	Tax_Id=9606 Gene_Symbol=GSN Isoform 1 of Gelsolin	AQPVQVAEGSEPDGFWEALGGK
751079880	565.605	23.609	2.3	0.716	0.308		
751079881	565.94	23.615	1.5	0.792	0.216		
751079882	566.274	23.631	1.5	0.777	0.233		
751079883	566.609	23.617	0.3	0.987	0.119		
751079884	900.438	18.824	1.4	0.797	0.318		
751079885	602.379	50.365	20.3	0.004	0.8		
751079886	602.509	50.368	21	0.002	0.72		
751079887	602.64	50.339	23	0.125	0.316		
751079888	606.629	18.826	1.8	0.742	0.397		
751079889	606.963	18.85	0.5	0.919	0.34		
751079890	607.29	18.726	3.2	0.564	0.59		
751079891	607.632	18.956	-1.6	0.647	0.087		
751079892	458.237	16.122	7.3	0.248	0.976		
751079893	458.572	16.126	7.8	0.234	0.993		
751079894	458.906	16.128	10	0.469	0.729		
751079895	527.278	15.997	6	0.198	0.721		
751079896	527.783	16.054	5	0.305	0.674		
751079897	659.604	50.379	19.4	0.003	0.657		
751079898	660.606	50.309	23.3	0.312	0.679		

751079899	742.382	28.982	2.3	0.234	0.565
751079900	742.717	28.947	2	0.326	0.646
751079901	743.051	28.957	1.6	0.448	0.81
751079902	743.386	28.951	1.1	0.609	0.927
751079903	743.721	28.948	-0.2	0.99	0.843
751079904	728.954	23.558	-3.4	0.76	0.082
751079905	729.079	23.559	0.5	0.949	0.156
751079906	729.205	23.56	-0.5	0.947	0.119
751079907	729.33	23.562	-1.5	0.854	0.154
751079908	729.456	23.564	-0.9	0.897	0.139
751079909	729.581	23.571	0.1	0.992	0.17
751079910	729.706	23.559	10	0.309	0.853
751079911	729.83	23.589	-1.2	0.887	0.161
751079912	729.962	23.518	-13.9	0.291	0.165
751079913	992.981	24.157	-0.7	0.889	0.495
751079914	993.482	24.148	-1.5	0.773	0.4
751079915	993.983	24.146	-0.9	0.871	0.525
751079916	994.484	24.153	-5.4	0.222	0.051
751079917	994.985	24.208	-42.6	0.03	0.015
751079918	499.255	19.718	2.7	0.618	0.396
751079919	499.506	19.723	3.5	0.497	0.465
751079920	499.757	19.725	2.5	0.64	0.419
751079921	500.008	19.742	28.1	0.268	0.894
751079922	532.758	30.1	4.4	0.477	0.838
751079923	533.26	30.106	3.8	0.547	0.73
751079924	533.762	30.112	8.9	0.537	0.745
751079925	610.154	44.33	6.9	0.486	0.42
751079926	610.321	44.325	8.1	0.608	0.385
751079927	610.488	44.33	6.9	0.407	0.454
751079928	610.655	44.334	7.4	0.403	0.459
751079929	610.823	44.322	4.7	0.547	0.277
751079930	610.989	44.322	8.1	0.409	0.229
751079931	611.157	44.283	16.8	0.37	0.944
751079932	967.572	50.212	-7	0.23	0.073
751079933	967.726	50.213	-7.1	0.209	0.07
751079934	967.879	50.213	-9.9	0.067	0.007
751079935	968.033	50.211	-10.7	0.085	0.025
751079936	968.185	50.155	-10	0.4	0.562
751079937	636.358	29.439	-6.4	0.151	0.032
751079938	636.692	29.448	-7.5	0.092	0.016
751079939	637.027	29.425	-7.1	0.147	0.054
751079940	637.363	29.275	-7.1	0.38	0.139
751079941	1147.517	43.38	0.9	0.832	0.249
751079942	1147.716	43.406	4.7	0.359	0.69
751079943	1147.915	43.419	4	0.386	0.647
751079944	1148.116	43.413	4.7	0.328	0.656
751079945	1148.316	43.413	4.1	0.4	0.558
751079946	1148.516	43.414	3.7	0.435	0.571
751079947	1148.717	43.408	3.2	0.501	0.753
751079948	1148.917	43.397	2.4	0.618	0.531
751079949	682.997	24.789	-3.1	0.646	0.196
751079950	683.331	24.783	-3.6	0.557	0.176
751079951	683.665	24.787	-3.4	0.615	0.205
751079952	683.999	24.789	-3.6	0.571	0.203
751079953	684.334	24.811	-48.6	0.001	0.063
751079954	696.347	22.825	10	0.319	0.306
751079955	696.681	22.796	13.1	0.184	0.175
751079956	697.015	22.794	12.2	0.186	0.228
751079957	697.349	22.805	7.3	0.22	0.722
751079958	697.325	14.856	2.5	0.803	0.156
751079959	698.328	14.853	8.6	0.695	0.393
751079960	918.448	18.87	1.5	0.81	0.354
751079961	918.95	18.849	0.4	0.918	0.162
751079962	919.451	18.911	-2	0.808	0.267
751079963	919.953	18.876	-7.5	0.564	0.237
751079964	628.637	20.691	-28.4	0.078	0.127
751079965	628.971	20.69	-7.4	0.182	0.115
751079966	629.305	20.812	-3.6	0.528	0.288
751079967	629.64	20.706	-15.2	0.206	0.159
751079968	599.275	36.353	13.3	0.591	0.572
751079969	599.476	36.351	5.3	0.446	0.217
751079970	599.676	36.361	11.1	0.271	0.595
751079971	599.877	36.352	12.5	0.499	0.265
751079972	600.077	36.352	10.4	0.489	0.282
751079973	600.28	36.515	0.3	0.94	0.562
751079974	651	28.092	2	0.843	0.239
751079975	651.334	28.092	-0.1	0.989	0.067
751079976	651.668	28.102	0.6	0.928	0.076
751079977	652.002	28.11	-5.2	0.595	0.041
751079978	578.975	23.366	-3.2	0.381	0.068
751079979	579.309	23.523	-5.6	0.145	0.025
751079980	579.644	23.36	-4	0.285	0.061
751079981	579.978	23.356	-20.3	0.085	0.117
751079982	751.867	29.238	1.7	0.681	0.654
751079983	752.37	29.275	2.1	0.599	0.581
751079984	752.87	29.273	1.6	0.701	0.585
751079985	753.368	29.332	-2.4	0.466	0.751
751079986	1078.249	30.79	7.2	0.168	0.61
751079987	1078.501	30.791	9.1	0.1	0.402
751079988	1078.751	30.794	8.3	0.14	0.394
751079989	1079.004	30.79	10.3	0.076	0.33
751079990	1079.262	30.811	8.6	0.103	0.402
751079991	1079.5	30.83	7.2	0.181	0.775

751079992	581.902	27.28	-3	0.549	0.148	Tax_Id=9606 Gene_Symbol=CD99L2 Isoform 3 of CD99 antigen-like protein	RKPIAGGGGFSDKDLEDIVGGGEYKPDK
751079993	582.103	27.277	-3.1	0.527	0.182	Tax_Id=9606 Gene_Symbol=CD99L2 Isoform 3 of CD99 antigen-like protein	RKPIAGGGGFSDKDLEDIVGGGEYKPDK
751079994	582.303	27.282	-3.2	0.509	0.154	Tax_Id=9606 Gene_Symbol=CD99L2 Isoform 3 of CD99 antigen-like protein	RKPIAGGGGFSDKDLEDIVGGGEYKPDK
751079995	582.504	27.283	-4.6	0.736	0.266	Tax_Id=9606 Gene_Symbol=CD99L2 Isoform 3 of CD99 antigen-like protein	RKPIAGGGGFSDKDLEDIVGGGEYKPDK
751079996	582.705	27.284	-2.7	0.767	0.149	Tax_Id=9606 Gene_Symbol=CD99L2 Isoform 3 of CD99 antigen-like protein	RKPIAGGGGFSDKDLEDIVGGGEYKPDK
751079997	582.906	27.279	-16.3	0.434	0.04	Tax_Id=9606 Gene_Symbol=CD99L2 Isoform 3 of CD99 antigen-like protein	RKPIAGGGGFSDKDLEDIVGGGEYKPDK
751079998	732.307	26.321	-2	0.787	0.488		
751079999	732.508	26.32	-18	0.251	0.302		
751080000	732.708	26.327	-3.5	0.633	0.333		
751080001	732.909	26.329	-2.3	0.72	0.388		
751080002	733.107	26.346	-0.9	0.907	0.643		
751080003	733.31	26.333	-0.5	0.937	0.598		
751080004	733.509	26.335	-7.3	0.674	0.723		
751080005	817.931	35.253	1.5	0.765	0.395	Tax_Id=9606 Gene_Symbol=APOA4 apolipoprotein A-IV precursor	SELTQQLNALFQDK
751080006	818.433	35.26	1.7	0.744	0.456	Tax_Id=9606 Gene_Symbol=APOA4 apolipoprotein A-IV precursor	SELTQQLNALFQDK
751080007	818.934	35.265	1.7	0.722	0.425	Tax_Id=9606 Gene_Symbol=APOA4 apolipoprotein A-IV precursor	SELTQQLNALFQDK
751080008	819.433	35.315	-1.9	0.774	0.362	Tax_Id=9606 Gene_Symbol=APOA4 apolipoprotein A-IV precursor	SELTQQLNALFQDK
751080009	1044.019	22.803	12.6	0.221	0.186		
751080010	1044.521	22.803	12.7	0.214	0.178		
751080011	1045.022	22.802	11.7	0.237	0.212		
751080012	1045.524	22.805	0.6	0.954	0.471		
751080013	505.895	16.531	10.1	0.099	0.298		
751080014	506.231	16.556	12.4	0.058	0.206		
751080015	506.564	16.531	11	0.147	0.2		
751080016	506.898	16.536	139.4	0.009	0.088		
751080017	966.878	50.424	0.3	0.961	0.319		
751080018	860.705	25.189	3.2	0.831	0.273	Tax_Id=9606 Gene_Symbol=KLK6 Isoform 2 of Kallikrein-6	EEC[160.0302]EHAYPGQITQNMLC[160.0302]AG
751080019	861.039	25.19	2.2	0.838	0.275	Tax_Id=9606 Gene_Symbol=KLK6 Isoform 2 of Kallikrein-6	EEC[160.0302]EHAYPGQITQNMLC[160.0302]AG
751080020	861.373	25.186	1.7	0.858	0.25	Tax_Id=9606 Gene_Symbol=KLK6 Isoform 2 of Kallikrein-6	EEC[160.0302]EHAYPGQITQNMLC[160.0302]AG
751080021	861.706	25.191	0.3	0.979	0.115	Tax_Id=9606 Gene_Symbol=KLK6 Isoform 2 of Kallikrein-6	EEC[160.0302]EHAYPGQITQNMLC[160.0302]AG
751080022	862.04	25.224	3.8	0.861	0.336	Tax_Id=9606 Gene_Symbol=KLK6 Isoform 2 of Kallikrein-6	EEC[160.0302]EHAYPGQITQNMLC[160.0302]AG
751080023	862.38	25.279	28.7	0.442	0.628	Tax_Id=9606 Gene_Symbol=KLK6 Isoform 2 of Kallikrein-6	EEC[160.0302]EHAYPGQITQNMLC[160.0302]AG
751080024	669.718	46.303	10.8	0.106	0.842		
751080025	471.241	16.175	6.5	0.477	0.837		
751080026	471.743	16.173	-0.5	0.942	0.748		
751080027	464.915	21.669	-2.4	0.657	0.431		
751080028	465.25	21.667	-4	0.445	0.224		
751080029	465.584	21.67	-4.8	0.421	0.089		
751080030	508.786	14.645	0.9	0.923	0.195		
751080031	509.288	14.645	11.9	0.606	0.548		
751080032	509.789	14.643	-14.9	0.379	0.022		
751080033	608.615	18.856	2.7	0.416	0.33		
751080034	608.95	18.878	2.9	0.317	0.229		
751080035	609.284	18.871	2.6	0.323	0.288		
751080036	609.619	18.918	-19.6	0.133	0.26		
751080037	837.001	35.894	-1.4	0.852	0.713		
751080038	837.502	35.892	-2	0.81	0.71		
751080039	838.004	35.893	1.8	0.871	0.507		
751080040	838.506	35.902	-12.6	0.311	0.223		
751080041	447.225	17.884	-1.1	0.956	0.283		
751080042	448.228	17.934	-1.4	0.922	0.304		
751080043	549.761	28.265	0	0.996	0.664		
751080044	550.263	28.231	0.2	0.963	0.643		
751080045	550.765	28.078	0.4	0.928	0.76		
751080046	601.303	23.325	14.7	0.073	0.116		
751080047	601.636	23.328	14.3	0.096	0.166		
751080048	601.971	23.315	12.5	0.131	0.188		
751080049	602.306	23.319	7.8	0.278	0.561		
751080050	822.056	31.098	10.9	0.106	0.74		
751080051	822.39	31.092	10.7	0.104	0.728		
751080052	822.724	31.106	11.7	0.087	0.654		
751080053	823.06	31.117	-2.5	0.77	0.187		
751080054	523.293	25.441	6.4	0.129	0.83		
751080055	523.628	25.349	6.7	0.114	0.851		
751080056	523.962	25.349	10.4	0.401	0.693		
751080057	524.296	25.307	6.4	0.5	0.551		
751080058	1014.465	27.202	1.4	0.844	0.62		
751080059	1014.717	27.193	-0.2	0.968	0.421		
751080060	1014.967	27.206	1.6	0.793	0.542		
751080061	1015.217	27.213	0.3	0.962	0.417		
751080062	1015.468	27.218	0.3	0.966	0.486		
751080063	1015.719	27.209	-3.2	0.743	0.259		
751080064	1015.971	27.207	4.4	0.841	0.766		
751080065	628.3	21.068	1.8	0.774	0.414	Tax_Id=9606 Gene_Symbol=ENPP2 Isoform 1 of Ectonucleotide pyrophosph	TYLHTYESEI
751080066	628.801	21.048	1.5	0.821	0.39	Tax_Id=9606 Gene_Symbol=ENPP2 Isoform 1 of Ectonucleotide pyrophosph	TYLHTYESEI
751080067	919.969	32.039	14.4	0.082	0.487		
751080068	920.471	32.041	13.1	0.102	0.556		
751080069	920.973	32.048	14.2	0.169	0.667		
751080070	921.473	32.038	3.8	0.671	0.896		
751080071	966.479	32.308	2.3	0.241	0.603		
751080072	966.983	32.209	2.3	0.261	0.583		
751080073	967.485	32.201	2.8	0.31	0.932		
751080074	967.988	32.143	1.2	0.665	0.346		
751080075	855.941	31.734	1.8	0.816	0.734		
751080076	856.441	31.752	2.3	0.746	0.773		
751080077	856.944	31.714	7.1	0.359	0.448		
751080078	857.445	31.675	-4.6	0.874	0.937		
751080079	737.711	28.623	-0.8	0.866	0.314		
751080080	738.045	28.623	-1.1	0.814	0.348		
751080081	738.382	28.627	-5.6	0.312	0.241		
751080082	738.717	28.638	2.1	0.902	0.921		
751080083	614.672	29.29	4.3	0.515	0.574	Tax_Id=9606 Gene_Symbol=C3 Complement C3 (Fragment)	VHQYFNVELIQPGAVK
751080084	615.006	29.292	4	0.512	0.58	Tax_Id=9606 Gene_Symbol=C3 Complement C3 (Fragment)	VHQYFNVELIQPGAVK

751080085	615.338	29.315	9.5	0.118	0.956	Tax_Id=9606 Gene_Symbol=C3 Complement C3 (Fragment)	VHQYFNVELIQPGAVK
751080086	615.674	29.301	12.7	0.573	0.713	Tax_Id=9606 Gene_Symbol=C3 Complement C3 (Fragment)	VHQYFNVELIQPGAVK
751080087	837.493	50.778	-0.3	0.951	0.27		
751080088	967.726	50.671	-4.1	0.27	0.009		
751080089	967.878	50.682	-2.7	0.472	0.011		
751080090	968.031	50.654	-4.2	0.268	0.007		
751080091	692.288	17.571	-0.6	0.907	0.34		
751080092	692.79	17.652	-0.8	0.882	0.336		
751080093	560.294	12.831	15	0.348	0.721		
751080094	561.297	12.825	15.4	0.47	0.573		
751080095	562.3	12.822	37.4	0.085	0.629		
751080096	853.238	47.36	9.6	0.095	0.498		
751080097	853.437	47.476	10.2	0.12	0.745		
751080098	853.637	47.547	8.4	0.109	0.437		
751080099	853.839	47.338	8.6	0.068	0.38		
751080100	854.04	47.317	8	0.087	0.374		
751080101	921.96	18.889	8.4	0.269	0.912		
751080102	922.461	18.921	6.7	0.345	0.812		
751080103	922.962	18.938	4	0.542	0.669		
751080104	923.463	18.954	12.7	0.306	0.452		
751080105	521.747	15.9	6.2	0.341	0.801		
751080106	522.249	15.893	4.8	0.464	0.961		
751080107	522.75	15.892	5.8	0.599	0.375		
751080108	408.213	14.899	11	0.314	0.279		
751080109	409.217	14.878	6.5	0.639	0.287		
751080110	514.612	17.652	5.2	0.316	0.64		
751080111	514.946	17.653	5.6	0.539	0.812		
751080112	515.28	17.69	-10.6	0.547	0.429		
751080113	515.615	17.672	-6.8	0.806	0.293		
751080114	675.341	27.863	-4.9	0.271	0.16	Tax_Id=9606 Gene_Symbol=CHL1 Isoform 1 of Neural cell adhesion molecul	VNGSPVDNHPFAGDVVFPR
751080115	675.676	27.859	-4.9	0.259	0.163	Tax_Id=9606 Gene_Symbol=CHL1 Isoform 1 of Neural cell adhesion molecul	VNGSPVDNHPFAGDVVFPR
751080116	676.01	27.865	-5.9	0.185	0.111	Tax_Id=9606 Gene_Symbol=CHL1 Isoform 1 of Neural cell adhesion molecul	VNGSPVDNHPFAGDVVFPR
751080117	676.346	27.877	-21.9	0.01	0.018	Tax_Id=9606 Gene_Symbol=CHL1 Isoform 1 of Neural cell adhesion molecul	VNGSPVDNHPFAGDVVFPR
751080118	728.905	51.48	3.8	0.222	0.51		
751080119	729.064	51.37	6.5	0.03	0.489		
751080120	729.22	51.725	6.5	0.03	0.827		
751080121	729.378	51.398	6.3	0.153	0.924		
751080122	494.25	22.139	9.7	0.485	0.531		
751080123	636.794	31.334	3.3	0.423	0.37		
751080124	637.294	30.945	-0.8	0.919	0.457		
751080125	637.797	31.286	3	0.509	0.44		
751080126	642.989	32.136	2	0.614	0.577		
751080127	643.337	32.134	0.8	0.792	0.289		
751080128	643.672	32.154	0.3	0.931	0.214		
751080129	644.006	32.143	-1.2	0.716	0.318		
751080130	577.622	21.948	0.8	0.899	0.326		
751080131	577.956	21.948	1.9	0.77	0.41		
751080132	578.29	21.937	-0.9	0.866	0.302		
751080133	578.626	21.938	-17.4	0.386	0.055		
751080134	826.758	33.691	-2.8	0.643	0.777		
751080135	827.093	33.702	-2.5	0.671	0.862		
751080136	827.428	33.697	-3.1	0.605	0.767		
751080137	827.763	33.687	-4.4	0.455	0.592		
751080138	828.095	33.658	-27.8	0.077	0.179		
751080139	494.751	21.828	9.5	0.512	0.554		
751080140	495.253	21.775	9.3	0.507	0.57		
751080141	495.758	21.555	23.6	0.307	0.526		
751080142	452.906	15.206	6.4	0.141	0.948		
751080143	453.241	15.203	6.9	0.116	0.898		
751080144	453.574	15.22	9	0.074	0.682		
751080145	719.87	35.559	7.7	0.215	0.712		
751080146	720.873	35.581	7.7	0.243	0.733		
751080147	785.337	50.401	2.2	0.689	0.746		
751080148	785.462	50.402	1.8	0.765	0.493		
751080149	785.587	50.427	1.8	0.766	0.425		
751080150	785.712	50.431	2.8	0.752	0.186		
751080151	785.837	50.432	5.8	0.624	0.273		
751080152	683.848	42.182	-2.8	0.682	0.259		
751080153	684.099	42.177	-3.7	0.554	0.203		
751080154	684.349	42.18	-2.8	0.666	0.241		
751080155	684.6	42.173	-4.1	0.573	0.171		
751080156	684.85	42.26	5.6	0.463	0.859		
751080157	1169.222	42.673	12.6	0.179	0.437		
751080158	1169.366	42.696	12.4	0.206	0.423		
751080159	1169.51	42.694	12.1	0.225	0.554		
751080160	1169.653	42.756	11.6	0.235	0.524		
751080161	1169.791	42.733	9.9	0.25	0.646		
751080162	1169.942	42.834	9	0.201	0.667		
751080163	1170.082	42.695	8	0.316	0.682		
751080164	570.818	16.155	7.4	0.211	0.989	Tax_Id=9606 Gene_Symbol=PLG Plasminogen	EAQLPVIENK
751080165	571.32	16.158	7	0.231	0.907	Tax_Id=9606 Gene_Symbol=PLG Plasminogen	EAQLPVIENK
751080166	571.822	16.189	12.2	0.235	0.715	Tax_Id=9606 Gene_Symbol=PLG Plasminogen	EAQLPVIENK
751080167	927.961	27.614	0.7	0.898	0.134	Tax_Id=9606 Gene_Symbol=SPP1 Isoform A of Osteopontin	AIPVAQDLNAPSDWDSR
751080168	928.454	27.539	-0.5	0.925	0.098	Tax_Id=9606 Gene_Symbol=SPP1 Isoform A of Osteopontin	AIPVAQDLNAPSDWDSR
751080169	928.963	27.621	0.6	0.927	0.112	Tax_Id=9606 Gene_Symbol=SPP1 Isoform A of Osteopontin	AIPVAQDLNAPSDWDSR
751080170	929.466	27.593	-8.3	0.415	0.1	Tax_Id=9606 Gene_Symbol=SPP1 Isoform A of Osteopontin	AIPVAQDLNAPSDWDSR
751080171	622.037	31.264	10.9	0.29	0.984		
751080172	622.162	31.272	4.5	0.712	0.532		
751080173	622.289	31.285	8.1	0.276	0.96		
751080174	622.413	31.284	6.2	0.624	0.556		
751080175	622.539	31.318	3.2	0.784	0.422		
751080176	622.662	31.298	-17.3	0.258	0.027		
751080177	717.369	30.193	2	0.77	0.199		

751080178	717.704	30.211	3	0.685	0.274		
751080179	718.038	30.221	3.9	0.702	0.2		
751080180	718.372	30.251	4.1	0.85	0.284		
751080181	906.666	27.067	3.1	0.628	0.645		
751080182	906.917	27.075	2.8	0.635	0.647		
751080183	907.168	27.082	3.6	0.56	0.67		
751080184	907.418	27.094	5.7	0.367	0.923		
751080185	907.668	27.08	6.4	0.4	0.998		
751080186	907.921	27.014	5.8	0.694	0.585		
751080187	518.263	16.284	0.9	0.968	0.315		
751080188	519.266	16.311	-0.9	0.954	0.227		
751080189	639.303	17.207	-2.6	0.657	0.211		
751080190	639.554	17.157	-2.6	0.647	0.155		
751080191	639.804	17.169	-3.1	0.603	0.172		
751080192	640.055	17.167	-4.8	0.441	0.137		
751080193	687.796	17.318	8.8	0.299	0.967		
751080194	688.295	17.386	11.5	0.297	0.829		
751080195	688.797	17.346	2.3	0.805	0.502		
751080196	452.236	24.689	2.4	0.501	0.601		
751080197	452.487	24.703	2.6	0.456	0.647		
751080198	452.738	24.679	2.6	0.476	0.573		
751080199	452.989	24.671	2.8	0.521	0.741		
751080200	601.804	17.794	18	0.028	0.634		
751080201	602.305	17.79	21.5	0.025	0.519		
751080202	602.807	17.779	16.3	0.112	0.914		
751080203	579.263	16.235	3.7	0.684	0.59	Tax_Id=9606 Gene_Symbol=SERPINF1 Pigment epithelium-derived factor	YGLDSDLSC[160.0302]K
751080204	579.764	16.235	3.7	0.722	0.636	Tax_Id=9606 Gene_Symbol=SERPINF1 Pigment epithelium-derived factor	YGLDSDLSC[160.0302]K
751080205	580.264	16.151	2.7	0.894	0.38	Tax_Id=9606 Gene_Symbol=SERPINF1 Pigment epithelium-derived factor	YGLDSDLSC[160.0302]K
751080206	783.971	43.275	2.1	0.798	0.614		
751080207	784.473	43.292	2.5	0.799	0.763		
751080208	784.976	43.304	3.1	0.702	0.809		
751080209	785.474	43.251	15.6	0.392	0.959		
751080210	639.306	14.803	8.1	0.626	0.815		
751080211	639.808	14.802	-28.2	0.407	0.376		
751080212	640.309	14.802	-5.8	0.761	0.158		
751080213	802.392	33.048	8.1	0.343	0.773		
751080214	941.046	45.518	-1.3	0.863	0.424		
751080215	941.548	45.533	-1.2	0.864	0.424		
751080216	942.048	45.537	-0.7	0.92	0.478		
751080217	942.554	45.557	-1.7	0.765	0.47		
751080218	540.31	18.486	-0.3	0.948	0.103		
751080219	540.812	18.479	-0.7	0.859	0.085		
751080220	692.365	21.483	-0.4	0.949	0.291		
751080221	693.369	21.454	0.8	0.947	0.385		
751080222	836.625	25.964	26.8	0.038	0.113	Tax_Id=9606 Gene_Symbol=FBLN1 Isoform C of Fibulin-1	MVQEQC[160.0302]C[160.0302]HSQLEELHC[16
751080223	836.876	25.983	25.2	0.044	0.091	Tax_Id=9606 Gene_Symbol=FBLN1 Isoform C of Fibulin-1	MVQEQC[160.0302]C[160.0302]HSQLEELHC[16
751080224	837.126	25.993	22.4	0.07	0.11	Tax_Id=9606 Gene_Symbol=FBLN1 Isoform C of Fibulin-1	MVQEQC[160.0302]C[160.0302]HSQLEELHC[16
751080225	837.377	25.996	22.3	0.072	0.125	Tax_Id=9606 Gene_Symbol=FBLN1 Isoform C of Fibulin-1	MVQEQC[160.0302]C[160.0302]HSQLEELHC[16
751080226	837.627	25.991	13.2	0.209	0.959	Tax_Id=9606 Gene_Symbol=FBLN1 Isoform C of Fibulin-1	MVQEQC[160.0302]C[160.0302]HSQLEELHC[16
751080227	837.878	25.998	24	0.238	0.942	Tax_Id=9606 Gene_Symbol=FBLN1 Isoform C of Fibulin-1	MVQEQC[160.0302]C[160.0302]HSQLEELHC[16
751080228	653.833	28.171	4.4	0.375	0.796	Tax_Id=9606 Gene_Symbol=CFB Isoform 1 of Complement factor B (Fragme	EKLQDEDLGFL
751080229	654.332	28.22	4.8	0.366	0.897	Tax_Id=9606 Gene_Symbol=CFB Isoform 1 of Complement factor B (Fragme	EKLQDEDLGFL
751080230	654.836	28.17	3	0.606	0.742	Tax_Id=9606 Gene_Symbol=CFB Isoform 1 of Complement factor B (Fragme	EKLQDEDLGFL
751080231	953.996	45.641	-16.4	0.009	0.025		
751080232	954.498	45.642	-16.4	0.009	0.024		
751080233	954.999	45.646	-17	0.006	0.017		
751080234	731.984	44.328	8	0.37	0.422		
751080235	732.185	44.327	7.5	0.4	0.417		
751080236	732.385	44.328	6.6	0.385	0.375		
751080237	732.586	44.33	6.1	0.378	0.353		
751080238	732.787	44.324	6.3	0.345	0.339		
751080239	732.987	44.312	6.1	0.364	0.25		
751080240	733.189	44.313	5.3	0.457	0.159		
751080241	844.427	31.846	8.3	0.323	0.882	Tax_Id=9606 Gene_Symbol=CP Ceruloplasmin	SVPPSASHVAPTETFTYEWTVPK
751080242	844.761	31.85	6.7	0.668	0.492	Tax_Id=9606 Gene_Symbol=CP Ceruloplasmin	SVPPSASHVAPTETFTYEWTVPK
751080243	845.087	31.939	2.3	0.711	0.929	Tax_Id=9606 Gene_Symbol=CP Ceruloplasmin	SVPPSASHVAPTETFTYEWTVPK
751080244	649.976	32.272	3.1	0.155	0.581		
751080245	650.311	32.264	3.3	0.107	0.692		
751080246	650.645	32.239	2.7	0.181	0.663		
751080247	650.979	32.22	1.6	0.491	0.58		
751080248	690.849	20.495	8.8	0.161	0.727		
751080249	691.352	20.44	11.9	0.1	0.443		
751080250	691.853	20.476	12.5	0.108	0.535		
751080251	564.798	22.11	5	0.404	0.771	Tax_Id=9606 Gene_Symbol=CP Ceruloplasmin	KAEEEHLGILGPQLHADVGDK
751080252	565.049	22.09	2.3	0.666	0.441	Tax_Id=9606 Gene_Symbol=CP Ceruloplasmin	KAEEEHLGILGPQLHADVGDK
751080253	565.299	22.091	3.5	0.535	0.606	Tax_Id=9606 Gene_Symbol=CP Ceruloplasmin	KAEEEHLGILGPQLHADVGDK
751080254	565.55	22.083	2.9	0.627	0.502	Tax_Id=9606 Gene_Symbol=CP Ceruloplasmin	KAEEEHLGILGPQLHADVGDK
751080255	522.283	15.628	6.9	0.385	0.561		
751080256	522.784	15.627	7.1	0.431	0.578		
751080257	523.286	15.628	10.4	0.604	0.711		
751080258	841.429	50.334	2.4	0.503	0.089		
751080259	841.639	50.334	4.6	0.27	0.128		
751080260	841.832	50.258	7.4	0.103	0.662		
751080261	842.031	50.259	8.1	0.116	0.784		
751080262	842.232	50.265	4.8	0.67	0.407		
751080263	842.432	50.26	5.6	0.23	0.939		
751080264	842.631	50.249	-1.8	0.73	0.247		
751080265	656.669	32.337	4	0.291	0.843		
751080266	657.003	32.323	3.8	0.342	0.867		
751080267	657.339	32.091	5.8	0.172	0.978		
751080268	785.274	50.4	1	0.856	0.298		
751080269	785.401	50.401	1.4	0.812	0.363		
751080270	785.524	50.409	0.6	0.924	0.437		

751080271	785.649	50.412	0.7	0.87	0.106		
751080272	785.774	50.434	0.5	0.957	0.093		
751080273	551.328	30.142	-6.4	0.122	0.014		
751080274	551.663	30.144	-6.4	0.157	0.021		
751080275	551.997	30.144	-3.9	0.356	0.035		
751080276	552.332	30.157	-3.2	0.804	0.167		
751080277	832.947	23.553	-2.8	0.76	0.123		
751080278	833.09	23.573	-0.7	0.917	0.12		
751080279	833.234	23.562	-1.5	0.839	0.135		
751080280	833.377	23.568	-0.3	0.963	0.163		
751080281	833.52	23.57	-1.5	0.831	0.126		
751080282	833.663	23.571	-1.4	0.874	0.15		
751080283	833.807	23.572	-1	0.908	0.162		
751080284	833.95	23.573	-3.8	0.728	0.149		
751080285	834.094	23.571	-21.3	0.389	0.297		
751080286	639.707	26.872	1.7	0.851	0.194		
751080287	639.907	26.871	2.4	0.723	0.686		
751080288	640.108	26.878	2.8	0.673	0.629		
751080289	640.306	26.966	2.3	0.714	0.867		
751080290	640.509	26.867	0.7	0.913	0.533		
751080291	640.709	26.853	9.2	0.469	0.933		
751080292	554.285	24.281	-6.6	0.156	0.067		
751080293	554.619	24.271	-6.1	0.168	0.059		
751080294	554.953	24.27	-6.6	0.158	0.072		
751080295	996.99	45.773	24.6	0.057	0.673	Tax_Id=9606 Gene_Symbol=C3 Complement C3 (Fragment)	HLIVTPSGC[160.0302]GEQNMIGMTPTVIAVHYL
751080296	997.24	45.859	23.6	0.047	0.444	Tax_Id=9606 Gene_Symbol=C3 Complement C3 (Fragment)	HLIVTPSGC[160.0302]GEQNMIGMTPTVIAVHYL
751080297	997.491	45.914	17.7	0.099	0.674	Tax_Id=9606 Gene_Symbol=C3 Complement C3 (Fragment)	HLIVTPSGC[160.0302]GEQNMIGMTPTVIAVHYL
751080298	997.744	45.697	17.4	0.316	0.853	Tax_Id=9606 Gene_Symbol=C3 Complement C3 (Fragment)	HLIVTPSGC[160.0302]GEQNMIGMTPTVIAVHYL
751080299	997.996	45.699	18.9	0.437	0.706	Tax_Id=9606 Gene_Symbol=C3 Complement C3 (Fragment)	HLIVTPSGC[160.0302]GEQNMIGMTPTVIAVHYL
751080300	998.247	45.716	14.8	0.518	0.644	Tax_Id=9606 Gene_Symbol=C3 Complement C3 (Fragment)	HLIVTPSGC[160.0302]GEQNMIGMTPTVIAVHYL
751080301	998.495	45.978	2.8	0.626	0.59	Tax_Id=9606 Gene_Symbol=C3 Complement C3 (Fragment)	HLIVTPSGC[160.0302]GEQNMIGMTPTVIAVHYL
751080302	619.332	31.426	3.6	0.526	0.388		
751080303	619.833	31.448	1.7	0.808	0.424		
751080304	620.343	31.38	-4.1	0.83	0.294		
751080305	1081.181	49.211	5.6	0.564	0.857		
751080306	868.954	51.334	5.8	0.064	0.334		
751080307	869.078	51.333	7.3	0.047	0.537		
751080308	869.203	51.356	7.8	0.011	0.713		
751080309	869.326	51.349	7.2	0.018	0.52		
751080310	869.451	51.33	6.7	0.111	0.668		
751080311	869.575	51.301	0.4	0.959	0.178		
751080312	658.342	35.287	1.9	0.636	0.173		
751080313	658.676	35.292	1.7	0.7	0.23		
751080314	659.01	35.296	1.8	0.697	0.196		
751080315	659.343	35.307	0.3	0.982	0.347		
751080316	659.679	35.32	-18.4	0.429	0.057		
751080317	641.987	32.125	3.3	0.497	0.729		
751080318	406.73	15.611	0.4	0.954	0.369		
751080319	407.232	15.591	-0.3	0.963	0.217		
751080320	407.733	15.582	-29.2	0.187	0.074		
751080321	740.83	14.127	-5.8	0.091	0.103	Tax_Id=9606 Gene_Symbol=DKK3 cDNA FLJ52545, highly similar to Dickko	SAVEEM[147.0355]EAEEAAAK
751080322	741.332	14.128	-5.9	0.086	0.088	Tax_Id=9606 Gene_Symbol=DKK3 cDNA FLJ52545, highly similar to Dickko	SAVEEM[147.0355]EAEEAAAK
751080323	741.833	14.126	-6.7	0.287	0.396	Tax_Id=9606 Gene_Symbol=DKK3 cDNA FLJ52545, highly similar to Dickko	SAVEEM[147.0355]EAEEAAAK
751080324	742.334	14.129	-28.8	0.185	0.676	Tax_Id=9606 Gene_Symbol=DKK3 cDNA FLJ52545, highly similar to Dickko	SAVEEM[147.0355]EAEEAAAK
751080325	1047.274	50.102	5.9	0.33	0.532		
751080326	1047.525	50.116	5.7	0.39	0.463		
751080327	1047.779	50.1	3.8	0.516	0.312		
751080328	1048.032	50.153	0.7	0.854	0.073		
751080329	1048.207	50.219	-8.9	0.109	0.018		
751080330	975.482	26.779	1.5	0.667	0.413	Tax_Id=9606 Gene_Symbol=PEBP1 Phosphatidylethanolamine-binding prote	GNDISSGTVLSDYVGSGPPK
751080331	975.989	26.739	1.3	0.685	0.235	Tax_Id=9606 Gene_Symbol=PEBP1 Phosphatidylethanolamine-binding prote	GNDISSGTVLSDYVGSGPPK
751080332	976.484	26.74	1.8	0.597	0.48	Tax_Id=9606 Gene_Symbol=PEBP1 Phosphatidylethanolamine-binding prote	GNDISSGTVLSDYVGSGPPK
751080333	976.991	26.743	0.2	0.962	0.379	Tax_Id=9606 Gene_Symbol=PEBP1 Phosphatidylethanolamine-binding prote	GNDISSGTVLSDYVGSGPPK
751080334	619.313	35.361	7.7	0.259	0.817		
751080335	619.813	35.371	7.9	0.251	0.743		
751080336	620.314	35.38	1.5	0.939	0.222		
751080337	620.807	35.41	-7	0.674	0.785		
751080338	962.48	32.106	2.6	0.604	0.629		
751080339	962.981	32.154	3.7	0.446	0.811		
751080340	704.362	18.18	0.6	0.907	0.315	Tax_Id=9606 Gene_Symbol=APOA4 apolipoprotein A-IV precursor	LGEVNTYAGDLQK
751080341	704.864	18.178	0.9	0.876	0.345	Tax_Id=9606 Gene_Symbol=APOA4 apolipoprotein A-IV precursor	LGEVNTYAGDLQK
751080342	705.366	18.184	4.5	0.738	0.636	Tax_Id=9606 Gene_Symbol=APOA4 apolipoprotein A-IV precursor	LGEVNTYAGDLQK
751080343	844.388	28.527	4.2	0.511	0.932		
751080344	844.889	28.567	4.3	0.485	0.993		
751080345	845.391	28.503	5.9	0.37	0.926		
751080346	670.275	46.344	10.7	0.095	0.874		
751080347	977.541	31.234	9.5	0.191	0.607		
751080348	978.541	31.222	6.6	0.288	0.367		
751080349	411.564	17.787	-2	0.669	0.12		
751080350	411.898	17.79	-1.2	0.807	0.162		
751080351	412.233	17.776	0	0.998	0.106		
751080352	811.589	45.505	7.9	0.356	0.429		
751080353	811.755	45.502	4.4	0.438	0.815		
751080354	811.923	45.509	5.3	0.33	0.869		
751080355	812.09	45.504	4.5	0.428	0.767		
751080356	812.257	45.503	5.2	0.341	0.834		
751080357	812.423	45.507	4.5	0.425	0.696		
751080358	812.592	45.512	8.1	0.36	0.489		
751080359	812.758	45.5	8.5	0.221	0.799		
751080360	575.55	22.293	8.3	0.088	0.733		
751080361	575.801	22.28	8.2	0.117	0.769		
751080362	576.051	22.288	8	0.109	0.86		
751080363	576.301	22.301	9.8	0.079	0.585		

751080364	576.552	22.349	6	0.713	0.847		
751080365	695.792	14.458	-3.5	0.641	0.839		
751080366	696.292	14.476	-2.7	0.704	0.886		
751080367	696.793	14.491	-3.5	0.777	0.553		
751080368	647.99	32.141	3.5	0.451	0.736		
751080369	648.325	32.106	3	0.41	0.6		
751080370	648.677	32.03	3.7	0.428	0.724		
751080371	649.001	32.116	2.7	0.621	0.843		
751080372	649.333	32.155	-11.6	0.475	0.378		
751080373	800.54	66.046	1.1	0.888	0.364		
751080374	801.042	66.045	0.6	0.941	0.389		
751080375	801.544	66.041	1.4	0.853	0.353		
751080376	802.046	66.044	2.1	0.773	0.241		
751080377	828.44	25.08	5.1	0.279	0.952		
751080378	828.941	25.097	5	0.266	0.968		
751080379	829.442	25.086	3.9	0.376	0.79		
751080380	829.943	25.112	-27	0.217	0.222		
751080381	754.998	35.045	-1.9	0.73	0.401		
751080382	755.336	34.971	0.2	0.975	0.577		
751080383	755.666	35.042	-1.6	0.783	0.45		
751080384	756	35.042	-4.4	0.732	0.334		
751080385	869.016	51.343	5.3	0.056	0.398		
751080386	869.141	51.348	9.2	0.013	0.743		
751080387	869.264	51.357	8.5	0.005	0.977		
751080388	869.39	51.337	4.9	0.147	0.707		
751080389	869.514	51.334	6.8	0.071	0.574		
751080390	893.912	36.33	-2	0.63	0.288		
751080391	894.162	36.322	-2	0.617	0.284		
751080392	894.413	36.323	-1.4	0.716	0.272		
751080393	894.662	36.319	-1.3	0.742	0.362		
751080394	894.915	36.32	-2.6	0.523	0.274		
751080395	895.171	36.315	0.7	0.86	0.578		
751080396	657.391	30.553	3.5	0.51	0.516	Tax_Id=9606 Gene_Symbol=CLEC3B Putative uncharacterized protein DKFZ	LDTLAQEVALLK
751080397	657.893	30.551	3.4	0.534	0.562	Tax_Id=9606 Gene_Symbol=CLEC3B Putative uncharacterized protein DKFZ	LDTLAQEVALLK
751080398	658.395	30.565	6.1	0.353	0.758	Tax_Id=9606 Gene_Symbol=CLEC3B Putative uncharacterized protein DKFZ	LDTLAQEVALLK
751080399	966.945	24.006	4.6	0.5	0.744		
751080400	967.446	24.005	5.5	0.433	0.691		
751080401	967.947	24.005	6.3	0.396	0.609		
751080402	968.45	23.87	-2.7	0.649	0.166		
751080403	699.349	23.219	7.6	0.38	0.333		
751080404	699.679	23.32	12.6	0.222	0.664		
751080405	700.011	23.351	6.1	0.443	0.316		
751080406	700.343	23.391	6.6	0.602	0.516		
751080407	971.485	32.103	3.5	0.486	0.722		
751080408	971.986	32.119	2.7	0.554	0.557		
751080409	972.489	32.107	2.7	0.592	0.647		
751080410	972.992	32.135	-1.3	0.763	0.235		
751080411	738.712	22.576	12.9	0.079	0.391		
751080412	739.046	22.577	12.3	0.081	0.415		
751080413	739.38	22.58	13.3	0.065	0.407		
751080414	739.714	22.587	15.5	0.067	0.441		
751080415	740.049	22.596	22.3	0.373	0.711		
751080416	558.967	18.766	10.5	0.114	0.464		
751080417	559.302	18.772	10.2	0.1	0.514		
751080418	559.636	18.769	9.6	0.16	0.588		
751080419	559.97	18.743	54.7	0.084	0.27		
751080420	742.854	37.102	3.5	0.415	0.174		
751080421	743.356	37.091	3.8	0.367	0.155		
751080422	743.857	37.115	3.5	0.716	0.228		
751080423	403.245	16.328	2.8	0.532	0.595		
751080424	403.747	16.328	10.8	0.222	0.923		
751080425	404.249	16.326	-25	0.348	0.22		
751080426	684.737	34.642	4	0.695	0.978		
751080427	684.938	34.641	-0.7	0.914	0.612		
751080428	685.138	34.642	-2.8	0.812	0.96		
751080429	685.337	34.635	-6.8	0.409	0.263		
751080430	685.539	34.639	-1.7	0.803	0.321		
751080431	685.739	34.638	15.3	0.288	0.31		
751080432	554.268	17.377	-4	0.301	0.675		
751080433	554.602	17.368	-4.7	0.222	0.478		
751080434	554.936	17.373	-4.3	0.305	0.954		
751080435	555.27	17.401	2.9	0.873	0.372		
751080436	742.692	16.405	-14.4	0.189	0.013	Tax_Id=9606 Gene_Symbol=CHGA Chromogranin-A	SEALAVDGAGKPGAEEAQDPEGK
751080437	743.026	16.405	-9	0.49	0.211	Tax_Id=9606 Gene_Symbol=CHGA Chromogranin-A	SEALAVDGAGKPGAEEAQDPEGK
751080438	743.361	16.417	-17.9	0.578	0.248	Tax_Id=9606 Gene_Symbol=CHGA Chromogranin-A	SEALAVDGAGKPGAEEAQDPEGK
751080439	743.695	16.423	-4.4	0.861	0.625	Tax_Id=9606 Gene_Symbol=CHGA Chromogranin-A	SEALAVDGAGKPGAEEAQDPEGK
751080440	642.812	21.688	-6	0.176	0.035	Tax_Id=9606 Gene_Symbol=CHL1 Isoform 1 of Neural cell adhesion molecul	GDLYFANVEEK
751080441	643.318	21.751	-5	0.339	0.121	Tax_Id=9606 Gene_Symbol=CHL1 Isoform 1 of Neural cell adhesion molecul	GDLYFANVEEK
751080442	643.814	21.686	-6.5	0.184	0.069	Tax_Id=9606 Gene_Symbol=CHL1 Isoform 1 of Neural cell adhesion molecul	GDLYFANVEEK
751080443	636.337	15.566	5.4	0.542	0.904		
751080444	636.839	15.571	4.8	0.593	0.852		
751080445	637.34	15.578	-33.5	0.255	0.277		
751080446	477.266	23.901	1.5	0.782	0.377		
751080447	477.6	23.906	3	0.563	0.44		
751080448	477.935	23.913	1.5	0.781	0.33		
751080449	602.846	46.294	10.5	0.146	0.97		
751080450	603.047	46.272	10.7	0.159	0.887		
751080451	603.248	46.314	11.3	0.181	0.839		
751080452	603.448	46.252	20.1	0.369	0.83		
751080453	977.015	41.878	8.2	0.143	0.492		
751080454	977.518	41.813	11.7	0.131	0.94		
751080455	978.016	41.891	11.9	0.064	0.985		
751080456	978.521	41.856	5.3	0.428	0.248		

751080457	549.315	16.245	-5.2	0.506	0.158	Tax_Id=9606 Gene_Symbol=CLSTN1 Isoform 2 of Calsyntenin-1 (Fragment)	IPDGVVSVSPK
751080458	549.816	16.248	-3.9	0.781	0.286	Tax_Id=9606 Gene_Symbol=CLSTN1 Isoform 2 of Calsyntenin-1 (Fragment)	IPDGVVSVSPK
751080459	550.318	16.234	-32.9	0.011	0.008	Tax_Id=9606 Gene_Symbol=CLSTN1 Isoform 2 of Calsyntenin-1 (Fragment)	IPDGVVSVSPK
751080460	1172.542	36.257	0.7	0.88	0.563		
751080461	1172.877	36.265	0.8	0.861	0.607		
751080462	1173.211	36.264	1.5	0.716	0.631		
751080463	1173.545	36.264	0.4	0.921	0.527		
751080464	1173.879	36.258	0.6	0.877	0.555		
751080465	1174.215	36.263	-0.9	0.857	0.612		
751080466	620.354	21.938	-5.9	0.503	0.285	Tax_Id=9606 Gene_Symbol=CNDP1 Beta-Ala-His dipeptidase	ALEQDLPVNIK
751080467	620.855	21.937	-7.9	0.463	0.279	Tax_Id=9606 Gene_Symbol=CNDP1 Beta-Ala-His dipeptidase	ALEQDLPVNIK
751080468	621.357	21.941	-16.4	0.345	0.65	Tax_Id=9606 Gene_Symbol=CNDP1 Beta-Ala-His dipeptidase	ALEQDLPVNIK
751080469	763.037	30.966	-0.2	0.968	0.354	Tax_Id=9606 Gene_Symbol=CRTAC1 Isoform 1 of Cartilage acidic protein 1	EHGDPLIEELNPGDALEPEGR
751080470	763.372	30.937	-0.3	0.952	0.416	Tax_Id=9606 Gene_Symbol=CRTAC1 Isoform 1 of Cartilage acidic protein 1	EHGDPLIEELNPGDALEPEGR
751080471	763.706	30.966	-0.9	0.854	0.332	Tax_Id=9606 Gene_Symbol=CRTAC1 Isoform 1 of Cartilage acidic protein 1	EHGDPLIEELNPGDALEPEGR
751080472	764.041	30.946	-17.6	0.056	0.069	Tax_Id=9606 Gene_Symbol=CRTAC1 Isoform 1 of Cartilage acidic protein 1	EHGDPLIEELNPGDALEPEGR
751080473	621.807	23.461	8.6	0.181	0.324		
751080474	622.309	23.463	7.7	0.221	0.351		
751080475	622.81	23.469	15.4	0.435	0.731		
751080476	1103.587	20.743	-4.7	0.279	0.187		
751080477	1104.59	20.747	-5.5	0.367	0.223		
751080478	1105.592	20.758	-10.3	0.705	0.653		
751080479	699.844	27.23	6.4	0.48	0.557		
751080480	700.347	27.227	5.5	0.53	0.504		
751080481	656.336	32.069	3.9	0.369	0.831		
751080482	924.418	44.572	2.2	0.774	0.577		
751080483	924.753	44.601	2.4	0.763	0.497		
751080484	925.086	44.599	2	0.8	0.497		
751080485	925.42	44.586	1.8	0.818	0.506		
751080486	925.754	44.558	0.6	0.934	0.431		
751080487	926.088	44.535	1.2	0.913	0.237		
751080488	1288.923	35.808	-0.1	0.989	0.298		
751080489	1289.258	35.815	0.3	0.932	0.352		
751080490	1289.592	35.822	0.3	0.938	0.29		
751080491	1289.926	35.811	0.4	0.928	0.306		
751080492	1290.261	35.767	-0.5	0.894	0.247		
751080493	1290.591	35.755	0.2	0.95	0.24		
751080494	554.258	25.331	9.9	0.392	0.517		
751080495	554.759	25.331	1.9	0.854	0.315		
751080496	555.264	25.357	13.1	0.44	0.864		
751080497	570.961	31.716	1.9	0.759	0.905		
751080498	571.294	31.717	1.8	0.793	0.976		
751080499	571.63	31.693	-3.9	0.65	0.36		
751080500	700.391	15.722	9.4	0.336	0.841		
751080501	701.394	15.748	10.9	0.532	0.591		
751080502	853.835	47.905	8.8	0.058	0.296		
751080503	854.036	47.879	9.2	0.042	0.319		
751080504	854.237	47.679	8.9	0.046	0.266		
751080505	854.439	47.681	10.3	0.028	0.442		
751080506	854.638	47.721	12.6	0.007	0.59		
751080507	647.319	15.281	-4	0.862	0.164		
751080508	647.653	15.29	-5.7	0.708	0.1		
751080509	647.987	15.294	-25.9	0.376	0.256		
751080510	648.322	15.28	-7.7	0.738	0.62		
751080511	736.616	30.655	-1	0.862	0.245		
751080512	736.866	30.665	-2.3	0.65	0.187		
751080513	737.117	30.693	-2.4	0.635	0.178		
751080514	737.365	30.703	-2	0.717	0.227		
751080515	737.614	30.7	-3.6	0.76	0.337		
751080516	967.172	28.312	3.7	0.673	0.666		
751080517	967.422	28.305	3.8	0.601	0.691		
751080518	967.673	28.334	4	0.609	0.765		
751080519	967.925	28.289	5	0.326	0.974		
751080520	968.174	28.339	1	0.889	0.419		
751080521	968.421	28.281	-5.4	0.458	0.065		
751080522	428.767	15.028	3.1	0.373	0.841		
751080523	429.269	15.03	5.8	0.334	0.657		
751080524	429.771	15.069	19.1	0.511	0.208		
751080525	966.688	40.588	1.6	0.634	0.646		
751080526	966.938	40.563	0.7	0.842	0.394		
751080527	967.191	40.544	1	0.762	0.434		
751080528	769.084	37.542	1.3	0.742	0.179		
751080529	769.335	37.562	1.3	0.752	0.196		
751080530	769.585	37.56	1.2	0.762	0.192		
751080531	769.836	37.554	1.3	0.743	0.176		
751080532	770.088	37.501	1.1	0.804	0.103		
751080533	770.34	37.418	-3.3	0.435	0.013		
751080534	788.667	42.726	4.4	0.5	0.808		
751080535	788.918	42.749	3.9	0.48	0.709		
751080536	789.169	42.756	3.3	0.501	0.709		
751080537	789.419	42.763	3.2	0.534	0.831		
751080538	789.67	42.771	8.2	0.612	0.571		
751080539	789.922	42.781	4.6	0.861	0.691		
751080540	597.807	23.83	6.1	0.157	0.885	Tax_Id=9606 Gene_Symbol=F2 Prothrombin (Fragment)	ELLESYIDGR
751080541	598.308	23.84	6.5	0.174	0.912	Tax_Id=9606 Gene_Symbol=F2 Prothrombin (Fragment)	ELLESYIDGR
751080542	598.81	23.846	8.6	0.17	0.945	Tax_Id=9606 Gene_Symbol=F2 Prothrombin (Fragment)	ELLESYIDGR
751080543	567.248	29.792	8.2	0.572	0.501		
751080544	567.582	29.792	8.3	0.586	0.346		
751080545	567.916	29.793	10.6	0.796	0.848		
751080546	568.25	29.783	7.1	0.733	0.246		
751080547	476.261	17.41	4.8	0.443	0.966		
751080548	476.595	17.414	5.8	0.337	0.906		
751080549	476.93	17.429	11.3	0.462	0.76		

751080550	477.264	17.456	20.6	0.595	0.494		
751080551	710.32	28.403	6	0.666	0.467		
751080552	710.57	28.403	7.2	0.507	0.343		
751080553	710.82	28.399	2.7	0.745	0.414		
751080554	711.071	28.398	6.5	0.547	0.325		
751080555	711.32	28.414	3.9	0.533	0.544		
751080556	711.572	28.413	5.5	0.648	0.26		
751080557	722.626	43.857	12.9	0.182	0.658	Tax_Id=9606 Gene_Symbol=APOA1 Apolipoprotein A-I	LREQLGPVTQEFWDNLEKETEGLR
751080558	722.876	43.859	11.2	0.185	0.608	Tax_Id=9606 Gene_Symbol=APOA1 Apolipoprotein A-I	LREQLGPVTQEFWDNLEKETEGLR
751080559	723.127	43.859	12.2	0.157	0.642	Tax_Id=9606 Gene_Symbol=APOA1 Apolipoprotein A-I	LREQLGPVTQEFWDNLEKETEGLR
751080560	723.377	43.858	12.9	0.087	0.862	Tax_Id=9606 Gene_Symbol=APOA1 Apolipoprotein A-I	LREQLGPVTQEFWDNLEKETEGLR
751080561	723.628	43.863	17.8	0.502	0.616	Tax_Id=9606 Gene_Symbol=APOA1 Apolipoprotein A-I	LREQLGPVTQEFWDNLEKETEGLR
751080562	776.397	33.858	12.6	0.053	0.651		
751080563	776.898	33.829	12.9	0.188	0.369		
751080564	777.398	33.844	9.7	0.348	0.393		
751080565	777.9	33.85	-0.7	0.972	0.085		
751080566	446.233	16.902	-4.4	0.441	0.13		
751080567	446.735	16.909	-41.7	0.135	0.158		
751080568	447.227	16.893	-24.2	0.093	0.023		
751080569	508.245	21.322	5.9	0.413	0.396		
751080570	508.58	21.313	9.1	0.519	0.473		
751080571	508.914	21.329	5.6	0.601	0.34		
751080572	509.247	21.333	-2.7	0.871	0.105		
751080573	497.473	21.915	5.9	0.169	0.7	Tax_Id=9606 Gene_Symbol=CP Ceruloplasmin	KAEEEHLGILGPQLHADVGDKVK
751080574	497.673	21.917	6.1	0.231	0.851	Tax_Id=9606 Gene_Symbol=CP Ceruloplasmin	KAEEEHLGILGPQLHADVGDKVK
751080575	497.874	21.915	5.7	0.12	0.748	Tax_Id=9606 Gene_Symbol=CP Ceruloplasmin	KAEEEHLGILGPQLHADVGDKVK
751080576	498.074	21.907	8.3	0.132	0.941	Tax_Id=9606 Gene_Symbol=CP Ceruloplasmin	KAEEEHLGILGPQLHADVGDKVK
751080577	498.275	21.902	12.4	0.322	0.978	Tax_Id=9606 Gene_Symbol=CP Ceruloplasmin	KAEEEHLGILGPQLHADVGDKVK
751080578	810.386	28.374	-5.3	0.138	0.01	Tax_Id=9606 Gene_Symbol=SCG2 cDNA FLJ51276, highly similar to Secret	VPGQGSSEDDLQEEEQIEQAIK
751080579	810.722	28.495	-11	0.064	0.037	Tax_Id=9606 Gene_Symbol=SCG2 cDNA FLJ51276, highly similar to Secret	VPGQGSSEDDLQEEEQIEQAIK
751080580	811.057	28.503	-10	0.077	0.034	Tax_Id=9606 Gene_Symbol=SCG2 cDNA FLJ51276, highly similar to Secret	VPGQGSSEDDLQEEEQIEQAIK
751080581	811.386	28.68	-20.9	0.002	0.006	Tax_Id=9606 Gene_Symbol=SCG2 cDNA FLJ51276, highly similar to Secret	VPGQGSSEDDLQEEEQIEQAIK
751080582	1043.024	43.393	10.4	0.074	0.572		
751080583	1043.526	43.393	11.2	0.286	0.416		
751080584	1044.027	43.396	11.1	0.205	0.349		
751080585	1044.53	43.37	10.5	0.175	0.581		
751080586	1045.038	43.331	6.2	0.509	0.389		
751080587	635.839	22.906	-4.5	0.381	0.173		
751080588	636.089	22.917	-3.2	0.531	0.234		
751080589	636.341	22.939	-5	0.322	0.116		
751080590	636.592	22.921	-7.3	0.162	0.054		
751080591	636.842	22.911	-31.7	0.003	0.003		
751080592	739.018	16.156	-32.4	0	0.006	Tax_Id=9606 Gene_Symbol=CHGA Chromogranin-A	SGEATDGARPQALPEPM[147.0355]QESK
751080593	739.352	16.18	-15.2	0	0.001	Tax_Id=9606 Gene_Symbol=CHGA Chromogranin-A	SGEATDGARPQALPEPM[147.0355]QESK
751080594	739.686	16.209	-17.3	0.067	0.165	Tax_Id=9606 Gene_Symbol=CHGA Chromogranin-A	SGEATDGARPQALPEPM[147.0355]QESK
751080595	740.019	16.248	-46	0.022	0.11	Tax_Id=9606 Gene_Symbol=CHGA Chromogranin-A	SGEATDGARPQALPEPM[147.0355]QESK
751080596	488.89	19.485	1.1	0.842	0.157		
751080597	489.225	19.526	0.9	0.862	0.195		
751080598	489.559	19.5	3.6	0.823	0.102		
751080599	489.893	19.521	-13.1	0.635	0.121		
751080600	989.524	25.722	15.2	0.108	0.202		
751080601	990.527	25.728	14	0.144	0.462		
751080602	956.742	32.051	5.5	0.353	0.852		
751080603	555.289	24.374	1.3	0.848	0.345		
751080604	555.79	24.393	2.9	0.757	0.104		
751080605	556.293	24.392	13.6	0.563	0.496		
751080606	472.273	15.679	-1.8	0.715	0.453		
751080607	472.607	15.685	-1.7	0.788	0.551		
751080608	472.942	15.696	1.1	0.933	0.589		
751080609	473.276	15.722	-21.9	0.414	0.73		
751080610	595.295	28.135	5.3	0.566	0.356		
751080611	595.546	28.126	8.8	0.328	0.783		
751080612	595.797	28.138	11.3	0.33	0.597		
751080613	596.047	28.132	18.1	0.271	0.612		
751080614	580.283	40.096	3.5	0.596	0.756		
751080615	580.786	40.084	2.9	0.692	0.66		
751080616	581.286	40.115	2.9	0.78	0.309		
751080617	608.337	33.109	0.4	0.927	0.15		
751080618	608.84	33.054	1.3	0.746	0.278		
751080619	609.341	33.081	-1.2	0.844	0.23		
751080620	450.265	25.018	4.2	0.381	0.163		
751080621	450.767	25.02	5.9	0.784	0.418		
751080622	967.117	40.521	-1.7	0.791	0.094		
751080623	967.443	40.296	-1.8	0.66	0.325		
751080624	967.787	40.503	5.1	0.515	0.331		
751080625	968.12	40.504	2	0.787	0.208		
751080626	968.453	40.527	0.7	0.931	0.253		
751080627	968.789	40.521	5.9	0.619	0.174		
751080628	969.128	40.881	2.7	0.487	0.156		
751080629	487.281	37.028	38	0.115	0.918		
751080630	487.783	37.032	18.4	0.591	0.42		
751080631	671.005	33.029	6	0.484	0.761		
751080632	671.34	33.022	7.2	0.43	0.696		
751080633	671.673	33.031	4.3	0.661	0.974		
751080634	672.008	33.045	10.1	0.562	0.826		
751080635	877.457	16.477	-0.8	0.924	0.557		
751080636	877.958	16.389	2.8	0.726	0.436		
751080637	878.459	16.463	-0.2	0.986	0.613		
751080638	878.961	16.4	5.1	0.884	0.822		
751080639	637.031	30.344	0.8	0.911	0.852		
751080640	637.365	30.201	0.5	0.941	0.958		
751080641	637.7	30.272	-0.9	0.896	0.739		
751080642	638.035	30.283	10.5	0.311	0.604		

751080643	806.752	31.047	7.2	0.418	0.539		
751080644	893.698	18.752	2.5	0.815	0.371		
751080645	894.199	18.768	5.9	0.71	0.504		
751080646	776.11	41.657	-1.7	0.824	0.274		
751080647	776.36	41.675	-2.8	0.726	0.23		
751080648	776.611	41.648	-4.7	0.577	0.199		
751080649	776.861	41.687	-2.6	0.757	0.305		
751080650	777.111	41.683	-3.9	0.567	0.163		
751080651	777.366	41.738	-4.9	0.484	0.204		
751080652	508.777	33.569	8.9	0.398	0.174		
751080653	509.027	33.565	9.6	0.497	0.387		
751080654	509.278	33.572	10.7	0.658	0.248		
751080655	509.528	33.592	20.3	0.4	0.353		
751080656	509.779	33.616	36.5	0.141	0.657		
751080657	687.065	45.079	9.5	0.081	0.832		
751080658	687.286	45.227	11.2	0.014	0.923		
751080659	687.509	45.225	10.4	0.034	0.807		
751080660	687.73	45.252	10.2	0.025	0.841		
751080661	754.365	14.44	8.2	0.458	0.222		
751080662	755.368	14.439	9	0.577	0.174		
751080663	756.369	14.437	11.5	0.695	0.366		
751080664	693.161	46.057	6.9	0.231	0.586	Tax_Id=9606 Gene_Symbol=C4A;C4B complement component 4B preproprc	LLLFSPSVVHLGVPLSVGVQLQDVPR
751080665	693.412	46.049	5.7	0.304	0.429	Tax_Id=9606 Gene_Symbol=C4A;C4B complement component 4B preproprc	LLLFSPSVVHLGVPLSVGVQLQDVPR
751080666	693.663	46.052	5.1	0.357	0.42	Tax_Id=9606 Gene_Symbol=C4A;C4B complement component 4B preproprc	LLLFSPSVVHLGVPLSVGVQLQDVPR
751080667	693.914	46.057	5.4	0.328	0.434	Tax_Id=9606 Gene_Symbol=C4A;C4B complement component 4B preproprc	LLLFSPSVVHLGVPLSVGVQLQDVPR
751080668	694.164	46.056	6.3	0.284	0.449	Tax_Id=9606 Gene_Symbol=C4A;C4B complement component 4B preproprc	LLLFSPSVVHLGVPLSVGVQLQDVPR
751080669	784.827	27.325	-8.1	0.049	0.023		
751080670	785.077	27.331	-6.3	0.149	0.107		
751080671	785.328	27.332	-6.2	0.141	0.051		
751080672	785.58	27.31	-6.8	0.122	0.085		
751080673	785.837	27.239	-4.3	0.335	0.156		
751080674	595.325	27.731	11	0.49	0.631		
751080675	595.658	27.747	8.6	0.361	0.711		
751080676	595.993	27.745	13.5	0.302	0.75		
751080677	596.326	27.737	3.8	0.333	0.967		
751080678	602.947	46.278	11.8	0.146	0.844		
751080679	603.148	46.316	11.3	0.103	0.838		
751080680	603.347	46.308	10.4	0.11	0.885		
751080681	603.549	46.223	11.5	0.576	0.528		
751080682	579.302	21.502	6.6	0.123	0.731		
751080683	579.553	21.514	7.1	0.097	0.869		
751080684	579.793	21.677	12.5	0.155	0.731		
751080685	580.054	21.484	7.9	0.1	0.649		
751080686	1200.484	34.017	10.4	0.398	0.932		
751080687	1200.817	34.009	15.3	0.316	0.704		
751080688	1201.151	34.012	13	0.357	0.911		
751080689	1201.484	34.02	10.5	0.402	0.912		
751080690	1201.818	34.018	-1.2	0.872	0.051		
751080691	1202.152	34.02	-9.2	0.538	0.135		
751080692	1202.491	34.019	-15.4	0.155	0.034		
751080693	838.696	50.2	-6.8	0.211	0.06		
751080694	838.827	50.173	-7	0.175	0.061		
751080695	838.961	50.207	-6.8	0.253	0.065		
751080696	839.093	50.204	-7.4	0.208	0.065		
751080697	521.794	14.759	11.5	0.624	0.417		
751080698	522.295	14.758	10.5	0.631	0.365		
751080699	522.797	14.747	10.8	0.734	0.706		
751080700	1202.538	31.226	4.6	0.106	0.85		
751080701	1202.873	31.25	4.4	0.116	0.895		
751080702	1203.196	31.228	4.3	0.151	0.863		
751080703	1203.54	31.241	10	0.027	0.423		
751080704	1203.877	31.206	3.4	0.222	0.754		
751080705	1204.191	31.177	7.3	0.252	0.303		
751080706	487.733	32.22	2.5	0.375	0.574		
751080707	487.984	32.227	3.1	0.291	0.708		
751080708	488.234	32.208	2.9	0.309	0.477		
751080709	488.485	32.187	-11.3	0.328	0.379		
751080710	468.27	18.05	11	0.119	0.353	Tax_Id=9606 Gene_Symbol=FBLN1 Isoform C of Fibulin-1	VSPHSGVVALTKPVPEPR
751080711	468.521	18.049	11.3	0.132	0.371	Tax_Id=9606 Gene_Symbol=FBLN1 Isoform C of Fibulin-1	VSPHSGVVALTKPVPEPR
751080712	468.772	18.053	11	0.11	0.483	Tax_Id=9606 Gene_Symbol=FBLN1 Isoform C of Fibulin-1	VSPHSGVVALTKPVPEPR
751080713	469.023	18.064	10.2	0.4	0.591	Tax_Id=9606 Gene_Symbol=FBLN1 Isoform C of Fibulin-1	VSPHSGVVALTKPVPEPR
751080714	535.582	18.274	19.8	0.53	0.249		
751080715	535.917	18.276	6.8	0.478	0.646		
751080716	536.251	18.266	14.5	0.293	0.996		
751080717	879.142	50.491	0.4	0.948	0.698		
751080718	879.474	50.447	0.6	0.909	0.431		
751080719	879.81	50.461	-0.9	0.889	0.296		
751080720	880.145	50.477	-15.1	0.282	0.371		
751080721	900.611	34.013	-1	0.92	0.066		
751080722	900.862	34.014	6.4	0.708	0.466		
751080723	901.112	34.011	6	0.724	0.448		
751080724	901.362	34.014	-0.4	0.96	0.042		
751080725	901.613	34.018	11.2	0.648	0.648		
751080726	901.863	34.024	4.2	0.753	0.166		
751080727	902.115	34.023	-34.1	0.029	0.015		
751080728	482.754	18.249	-4.1	0.553	0.411		
751080729	483.256	18.249	-0.7	0.962	0.488		
751080730	483.758	18.272	-56.4	0.013	0.011		
751080731	600.761	21.263	14.5	0.321	0.634		
751080732	601.01	21.265	14	0.346	0.675		
751080733	601.263	21.253	9.1	0.186	0.758		
751080734	601.513	21.264	-23.9	0.417	0.342		
751080735	601.764	21.233	-10.1	0.588	0.16		

751080736	639.629	24.826	-10.9	0.026	0.023	Tax_Id=9606 Gene_Symbol=VGF Neurosecretory protein VGF	NSEPQDEGELFQGVDPR
751080737	639.963	24.837	-11.4	0.011	0.008	Tax_Id=9606 Gene_Symbol=VGF Neurosecretory protein VGF	NSEPQDEGELFQGVDPR
751080738	640.297	24.841	-10.7	0.02	0.016	Tax_Id=9606 Gene_Symbol=VGF Neurosecretory protein VGF	NSEPQDEGELFQGVDPR
751080739	640.632	24.885	-60.8	0	0.001	Tax_Id=9606 Gene_Symbol=VGF Neurosecretory protein VGF	NSEPQDEGELFQGVDPR
751080740	816.646	36.283	-0.8	0.931	0.842		
751080741	816.897	36.282	-5.1	0.544	0.321		
751080742	817.148	36.278	-6	0.488	0.29		
751080743	817.398	36.273	-0.1	0.991	0.865		
751080744	817.649	36.27	-36.8	0.038	0.02		
751080745	867.074	25.386	9.4	0.127	0.32		
751080746	867.408	25.379	10.4	0.102	0.294		
751080747	867.742	25.434	6.9	0.219	0.475		
751080748	868.077	25.45	7.1	0.202	0.433		
751080749	868.411	25.552	-2.6	0.717	0.531		
751080750	731.222	50.908	9.7	0.033	0.625		
751080751	731.379	50.91	8.3	0.074	0.834		
751080752	731.537	50.903	6.9	0.118	0.576		
751080753	650.316	38.399	6.1	0.402	0.462		
751080754	650.518	38.439	9	0.416	0.426		
751080755	650.718	38.448	7.2	0.518	0.348		
751080756	650.919	38.461	8	0.453	0.365		
751080757	651.12	38.475	7.2	0.606	0.223		
751080758	651.32	38.445	7.2	0.431	0.448		
751080759	1099.833	31.475	3	0.303	0.983		
751080760	1100.168	31.488	2.2	0.466	0.976		
751080761	1100.502	31.465	3.4	0.259	0.858		
751080762	1100.836	31.469	3.4	0.299	0.762		
751080763	1101.172	31.448	2.8	0.412	0.9		
751080764	1101.507	31.401	-8.7	0.538	0.54		
751080765	956.419	40.047	0.4	0.89	0.564		
751080766	956.677	40.308	2.2	0.424	0.826		
751080767	956.929	40.262	2	0.452	0.767		
751080768	957.179	40.326	2.6	0.423	0.723		
751080769	957.436	40.299	2	0.591	0.809		
751080770	957.683	40.19	2.2	0.481	0.913		
751080771	957.936	40.112	2	0.539	0.422		
751080772	488.225	28.729	6.5	0.237	0.357		
751080773	488.56	28.73	8.6	0.477	0.386		
751080774	488.894	28.735	7.9	0.608	0.206		
751080775	509.287	29.788	3.5	0.489	0.354		
751080776	509.788	29.792	7.4	0.73	0.474		
751080777	510.285	29.792	1	0.941	0.14		
751080778	584.587	30.99	6.1	0.757	0.2		
751080779	584.73	30.999	6.6	0.517	0.248		
751080780	584.873	31.006	9.5	0.676	0.459		
751080781	585.016	31.009	7.2	0.314	0.321		
751080782	585.16	31.019	5.9	0.586	0.182		
751080783	585.303	30.977	14.6	0.509	0.541		
751080784	838.763	50.196	-6.6	0.252	0.114		
751080785	838.895	50.205	-4.8	0.372	0.124		
751080786	839.027	50.205	-8.7	0.177	0.065		
751080787	839.161	50.236	13.9	0.575	0.519		
751080788	596.262	17.615	-4.9	0.359	0.344		
751080789	596.597	17.616	-5.6	0.378	0.44		
751080790	596.931	17.62	-6.9	0.406	0.291		
751080791	597.265	17.588	-18.1	0.066	0.169		
751080792	508.247	20.3	-2.4	0.65	0.075		
751080793	508.749	20.298	-1.2	0.865	0.068		
751080794	1107.056	25.025	6.3	0.37	0.955		
751080795	1107.553	25.075	5.3	0.41	0.984		
751080796	1108.058	25.074	2	0.627	0.428		
751080797	1108.553	25.074	2.7	0.405	0.32		
751080798	1109.061	25.045	-16	0.38	0.426		
751080799	520.775	24.093	9.7	0.156	0.827		
751080800	885.555	66.067	-1.6	0.833	0.452		
751080801	886.557	66.07	-2	0.794	0.418		
751080802	682.376	39.054	7.5	0.249	0.472	Tax_Id=9606 Gene_Symbol=A2M Alpha-2-macroglobulin	LLLQQVSLPELPGEYSMK
751080803	682.71	39.048	10.6	0.342	0.346	Tax_Id=9606 Gene_Symbol=A2M Alpha-2-macroglobulin	LLLQQVSLPELPGEYSMK
751080804	683.045	39.054	10.5	0.309	0.393	Tax_Id=9606 Gene_Symbol=A2M Alpha-2-macroglobulin	LLLQQVSLPELPGEYSMK
751080805	683.379	39.065	11.7	0.217	0.584	Tax_Id=9606 Gene_Symbol=A2M Alpha-2-macroglobulin	LLLQQVSLPELPGEYSMK
751080806	779.717	34.993	2.6	0.793	0.944	Tax_Id=9606 Gene_Symbol=SPARCL1 cDNA FLJ52396, highly similar to SF	NILMQLYEANSEHAGYLNEK
751080807	780.051	34.998	2.2	0.848	0.798	Tax_Id=9606 Gene_Symbol=SPARCL1 cDNA FLJ52396, highly similar to SF	NILMQLYEANSEHAGYLNEK
751080808	780.386	34.978	1	0.885	0.617	Tax_Id=9606 Gene_Symbol=SPARCL1 cDNA FLJ52396, highly similar to SF	NILMQLYEANSEHAGYLNEK
751080809	780.72	35.013	2.3	0.794	0.913	Tax_Id=9606 Gene_Symbol=SPARCL1 cDNA FLJ52396, highly similar to SF	NILMQLYEANSEHAGYLNEK
751080810	781.055	34.995	-18.6	0.164	0.339	Tax_Id=9606 Gene_Symbol=SPARCL1 cDNA FLJ52396, highly similar to SF	NILMQLYEANSEHAGYLNEK
751080811	499.8	28.366	-2.2	0.831	0.298		
751080812	500.301	28.375	0.4	0.968	0.268		
751080813	1072.212	47.634	4.3	0.448	0.862		
751080814	1072.325	47.626	5.1	0.36	0.627		
751080815	1072.436	47.62	7.1	0.194	0.892		
751080816	1072.546	47.618	5.6	0.266	0.775		
751080817	1072.656	47.619	6.3	0.209	0.81		
751080818	1072.768	47.619	4.7	0.418	0.693		
751080819	1072.88	47.621	5.2	0.357	0.957		
751080820	1072.993	47.625	3	0.596	0.537		
751080821	1073.101	47.629	6.5	0.182	0.809		
751080822	890.437	49.38	4.6	0.34	0.448	Tax_Id=9606 Gene_Symbol=CP Ceruloplasmin	GVYSSDVFDIFPGTYQTLEMFPR
751080823	890.768	49.39	7.1	0.599	0.662	Tax_Id=9606 Gene_Symbol=CP Ceruloplasmin	GVYSSDVFDIFPGTYQTLEMFPR
751080824	891.101	49.387	4.3	0.563	0.65	Tax_Id=9606 Gene_Symbol=CP Ceruloplasmin	GVYSSDVFDIFPGTYQTLEMFPR
751080825	891.436	49.392	3.6	0.564	0.49	Tax_Id=9606 Gene_Symbol=CP Ceruloplasmin	GVYSSDVFDIFPGTYQTLEMFPR
751080826	891.772	49.403	6	0.627	0.568	Tax_Id=9606 Gene_Symbol=CP Ceruloplasmin	GVYSSDVFDIFPGTYQTLEMFPR
751080827	521.308	15.808	1.7	0.715	0.43		
751080828	521.809	15.814	3	0.604	0.447		

751080829	522.311	15.822	1.8	0.757	0.315		
751080830	574.775	20.856	0.2	0.969	0.351		
751080831	575.276	20.867	0.4	0.925	0.283		
751080832	575.777	20.88	1.8	0.85	0.427		
751080833	595.67	19.925	-1.3	0.843	0.35		
751080834	595.87	19.925	-3.8	0.444	0.107		
751080835	596.071	19.928	-0.7	0.918	0.337		
751080836	415.727	17.443	5.4	0.696	0.377		
751080837	416.228	17.435	3.6	0.437	0.645		
751080838	774.868	18.179	1.5	0.846	0.605		
751080839	775.371	18.114	5.3	0.469	0.912		
751080840	775.874	18.133	-23	0.291	0.42		
751080841	565.3	17.127	5.8	0.273	0.491		
751080842	565.802	17.111	4.9	0.402	0.409		
751080843	566.303	17.13	-7.7	0.675	0.138		
751080844	1094.858	36.23	11.2	0.155	0.626	Tax_Id=9606 Gene_Symbol=HPX Hemopexin	SLGPNSC[160.0302]SANGPGLYLIHGPNLYC[16
751080845	1095.19	36.354	13.1	0.138	0.528	Tax_Id=9606 Gene_Symbol=HPX Hemopexin	SLGPNSC[160.0302]SANGPGLYLIHGPNLYC[16
751080846	1095.523	36.444	9.1	0.215	0.594	Tax_Id=9606 Gene_Symbol=HPX Hemopexin	SLGPNSC[160.0302]SANGPGLYLIHGPNLYC[16
751080847	1095.856	36.516	9.1	0.221	0.583	Tax_Id=9606 Gene_Symbol=HPX Hemopexin	SLGPNSC[160.0302]SANGPGLYLIHGPNLYC[16
751080848	1096.193	36.228	10.9	0.2	0.653	Tax_Id=9606 Gene_Symbol=HPX Hemopexin	SLGPNSC[160.0302]SANGPGLYLIHGPNLYC[16
751080849	473.538	15.709	4	0.412	0.684		
751080850	473.873	15.709	13.5	0.174	0.64		
751080851	474.207	15.716	3.1	0.855	0.347		
751080852	474.541	15.723	-4.8	0.861	0.4		
751080853	539.956	22.247	3.6	0.33	0.483		
751080854	540.291	22.21	3.4	0.395	0.479		
751080855	540.625	22.199	3.3	0.377	0.483		
751080856	540.959	22.191	-3.4	0.777	0.166		
751080857	660.974	31.25	0.3	0.983	0.384		
751080858	661.31	31.272	0.2	0.991	0.402		
751080859	661.643	31.261	-0.1	0.99	0.261		
751080860	661.977	31.269	-12	0.408	0.209		
751080861	687.174	45.232	11.7	0.011	0.845		
751080862	687.397	45.23	10.9	0.023	0.906		
751080863	687.62	45.166	10.9	0.03	0.565		
751080864	639.951	29.484	4	0.705	0.124		
751080865	640.286	29.479	3.6	0.777	0.119		
751080866	640.619	29.491	7.1	0.756	0.215		
751080867	640.953	29.501	2.5	0.889	0.08		
751080868	690.864	18.145	3.9	0.345	0.389		
751080869	691.357	17.984	2.5	0.66	0.182		
751080870	691.865	18.124	9	0.34	0.89		
751080871	623.358	16.898	6.7	0.282	0.753		
751080872	623.86	16.893	2.9	0.662	0.417		
751080873	624.362	16.874	-12.1	0.56	0.366		
751080874	897.918	27.767	0.1	0.984	0.141		
751080875	898.42	27.767	-0.9	0.837	0.139		
751080876	898.92	27.768	-1.4	0.88	0.162		
751080877	899.421	27.781	-23.8	0.115	0.233		
751080878	758.849	28.718	-2.6	0.711	0.705	Tax_Id=9606 Gene_Symbol=SERPINF1 Pigment epithelium-derived factor	TSLEDFYLDEER
751080879	759.352	28.695	-1	0.889	0.859	Tax_Id=9606 Gene_Symbol=SERPINF1 Pigment epithelium-derived factor	TSLEDFYLDEER
751080880	759.853	28.709	-2.4	0.772	0.748	Tax_Id=9606 Gene_Symbol=SERPINF1 Pigment epithelium-derived factor	TSLEDFYLDEER
751080881	507.768	18.159	1.5	0.863	0.831		
751080882	508.27	18.168	-0.4	0.966	0.73		
751080883	508.771	18.182	-29.7	0.39	0.467		
751080884	710.613	31.254	7.6	0.69	0.379		
751080885	710.757	31.258	-4	0.8	0.205		
751080886	710.9	31.258	4.6	0.774	0.405		
751080887	711.043	31.255	-7.3	0.491	0.122		
751080888	711.186	31.24	-0.9	0.953	0.266		
751080889	711.33	31.239	4.4	0.725	0.465		
751080890	711.473	31.202	11	0.449	0.787		
751080891	711.616	31.186	28.3	0.272	0.255		
751080892	904.439	18.851	1.7	0.679	0.381		
751080893	904.94	18.884	0.4	0.878	0.144		
751080894	905.441	18.902	-0.2	0.944	0.365		
751080895	905.945	18.843	-2.9	0.828	0.819		
751080896	644.826	19.414	8	0.047	0.469	Tax_Id=9606 Gene_Symbol=CLU Isoform 2 of Clusterin	ELDESLQVAER
751080897	645.33	19.54	9	0.044	0.435	Tax_Id=9606 Gene_Symbol=CLU Isoform 2 of Clusterin	ELDESLQVAER
751080898	645.828	19.357	6.8	0.226	0.915	Tax_Id=9606 Gene_Symbol=CLU Isoform 2 of Clusterin	ELDESLQVAER
751080899	612.633	18.827	1.7	0.813	0.398		
751080900	612.968	18.838	2.1	0.757	0.403		
751080901	613.302	18.864	0.1	0.98	0.281		
751080902	613.637	18.841	-0.3	0.948	0.137		
751080903	815.718	22.215	13.1	0.216	0.397		
751080904	816.721	22.228	15.3	0.135	0.805		
751080905	741.374	23.501	4.3	0.455	0.818		
751080906	741.698	23.514	4.7	0.441	0.862		
751080907	742.034	23.561	3.1	0.565	0.706		
751080908	742.368	23.356	1.5	0.919	0.603		
751080909	498.253	29.808	7.8	0.28	0.483		
751080910	498.588	29.787	7.9	0.055	0.32		
751080911	498.922	29.789	7.7	0.434	0.28		
751080912	909.443	18.818	2.7	0.668	0.448		
751080913	909.944	18.845	0.3	0.936	0.091		
751080914	910.446	18.852	-1.1	0.876	0.202		
751080915	910.95	18.886	-17.4	0.477	0.489		
751080916	534.771	15.058	2.6	0.889	0.36		
751080917	535.273	15.061	4	0.856	0.452		
751080918	535.774	15.043	5.8	0.715	0.362		
751080919	478.618	28.815	7.6	0.136	0.969		
751080920	478.953	28.816	7.7	0.141	0.906		
751080921	479.288	28.781	4.4	0.608	0.684		

751080922	604.359	24.312	10.7	0.265	0.217		
751080923	604.86	24.313	9.8	0.32	0.213		
751080924	605.362	24.308	-17.1	0.376	0.169		
751080925	738.676	23.508	11.6	0.011	0.181	Tax_Id=9606 Gene_Symbol=- cDNA FLJ51265, moderately similar to Beta-2	KC[160.0302]SYTEDAQC[160.0302]IDGTIEVPK
751080926	739.011	23.503	15.5	0.009	0.109	Tax_Id=9606 Gene_Symbol=- cDNA FLJ51265, moderately similar to Beta-2	KC[160.0302]SYTEDAQC[160.0302]IDGTIEVPK
751080927	739.345	23.511	11.8	0.023	0.222	Tax_Id=9606 Gene_Symbol=- cDNA FLJ51265, moderately similar to Beta-2	KC[160.0302]SYTEDAQC[160.0302]IDGTIEVPK
751080928	739.68	23.499	10.2	0.029	0.593	Tax_Id=9606 Gene_Symbol=- cDNA FLJ51265, moderately similar to Beta-2	KC[160.0302]SYTEDAQC[160.0302]IDGTIEVPK
751080929	761.41	35.588	-5.6	0.581	0.52		
751080930	761.661	35.588	2.6	0.624	0.636		
751080931	761.912	35.59	2.8	0.754	0.812		
751080932	762.163	35.579	-6.1	0.414	0.089		
751080933	762.416	35.665	-3.9	0.759	0.297		
751080934	1255.737	50.734	-9.4	0.328	0.016		
751080935	1255.839	50.752	1.8	0.82	0.557		
751080936	1255.943	50.75	7.1	0.37	0.968		
751080937	1256.045	50.745	8.3	0.3	0.624		
751080938	1256.25	50.771	-1.5	0.808	0.063		
751080939	1256.347	50.763	2.7	0.671	0.447		
751080940	1256.448	50.78	2.4	0.727	0.458		
751080941	1256.552	50.773	-14.8	0.159	0.131		
751080942	1256.651	50.848	2.2	0.664	0.614		
751080943	721.638	25.022	-18	0	0.001	Tax_Id=9606 Gene_Symbol=VGF Neurosecretory protein VGF	VGEEDEEAAEAEAEAEEAER
751080944	721.972	25.022	-18	0	0.001	Tax_Id=9606 Gene_Symbol=VGF Neurosecretory protein VGF	VGEEDEEAAEAEAEAEEAER
751080945	722.306	25.001	-20.6	0	0	Tax_Id=9606 Gene_Symbol=VGF Neurosecretory protein VGF	VGEEDEEAAEAEAEAEEAER
751080946	722.64	24.97	-21.9	0	0	Tax_Id=9606 Gene_Symbol=VGF Neurosecretory protein VGF	VGEEDEEAAEAEAEAEEAER
751080947	773.426	17.552	5.6	0.699	0.432		
751080948	774.429	17.558	1.2	0.925	0.094		
751080949	1337.267	40.087	1.8	0.638	0.487		
751080950	1337.604	40.131	1.7	0.665	0.4		
751080951	1337.938	40.13	2.1	0.595	0.48		
751080952	1338.274	40.133	1.5	0.706	0.385		
751080953	1338.608	40.106	1.5	0.712	0.4		
751080954	1338.941	40.036	0.6	0.892	0.408		
751080955	1339.277	40.01	4.9	0.472	0.285		
751080956	596.274	22.307	8.2	0.336	0.778		
751080957	597.279	22.446	6.4	0.382	0.945		
751080958	787.824	43.386	-1.4	0.817	0.235		
751080959	788.024	43.385	-1.6	0.775	0.216		
751080960	788.225	43.39	-1.2	0.821	0.226		
751080961	788.426	43.385	-1.1	0.845	0.23		
751080962	788.626	43.417	-1.6	0.758	0.247		
751080963	788.827	43.393	-1.2	0.868	0.119		
751080964	619.798	34.723	-4.2	0.839	0.28	Tax_Id=9606 Gene_Symbol=CHGA Chromogranin-A	HSGFEDELSEVLENQSSQAELK
751080965	620.047	34.742	-6.3	0.763	0.248	Tax_Id=9606 Gene_Symbol=CHGA Chromogranin-A	HSGFEDELSEVLENQSSQAELK
751080966	620.299	34.742	-6.2	0.638	0.191	Tax_Id=9606 Gene_Symbol=CHGA Chromogranin-A	HSGFEDELSEVLENQSSQAELK
751080967	620.549	34.76	-1.3	0.89	0.12	Tax_Id=9606 Gene_Symbol=CHGA Chromogranin-A	HSGFEDELSEVLENQSSQAELK
751080968	620.801	34.728	-11	0.394	0.198	Tax_Id=9606 Gene_Symbol=CHGA Chromogranin-A	HSGFEDELSEVLENQSSQAELK
751080969	649.958	23.998	-0.7	0.909	0.59		
751080970	650.294	23.925	0.9	0.871	0.935		
751080971	650.629	23.95	0.4	0.944	0.996		
751080972	650.962	23.932	-0.8	0.871	0.892		
751080973	651.295	23.941	-5.6	0.184	0.204		
751080974	657.345	26.973	9.4	0.062	0.576		
751080975	657.68	26.983	9.5	0.059	0.531		
751080976	658.015	26.985	8.5	0.082	0.75		
751080977	658.349	26.992	3.9	0.659	0.427		
751080978	1084.576	48.608	-6.9	0.348	0.167		
751080979	1084.776	48.668	-6	0.436	0.18		
751080980	1084.976	48.722	-8.4	0.281	0.143		
751080981	1085.177	48.757	-7.1	0.347	0.14		
751080982	1085.379	48.54	-9.2	0.389	0.567		
751080983	701.733	34.858	-16	0.278	0.241		
751080984	702.067	34.858	-1.4	0.727	0.086		
751080985	702.404	34.873	-2.3	0.587	0.114		
751080986	702.736	34.904	-1.9	0.745	0.175		
751080987	405.939	15.84	5.2	0.471	0.782		
751080988	406.19	15.841	2.6	0.629	0.794		
751080989	406.441	15.847	4.5	0.84	0.804		
751080990	406.691	15.823	-7	0.625	0.114		
751080991	1424.727	28.74	8.4	0.367	0.223		
751080992	1425.729	28.737	10	0.481	0.195		
751080993	1426.731	28.753	7	0.643	0.093		
751080994	1111.515	49.174	2.2	0.655	0.655		
751080995	1112.017	49.197	0.1	0.985	0.463		
751080996	1112.524	49.205	2.6	0.542	0.564		
751080997	1113.027	49.178	0.5	0.907	0.42		
751080998	649.309	15.49	-5	0.559	0.185		
751080999	649.819	15.563	-0.7	0.921	0.282		
751081000	650.323	15.62	2.3	0.732	0.437		
751081001	616.843	17.811	-1.7	0.697	0.108		
751081002	617.345	17.797	-1.8	0.701	0.142		
751081003	617.846	17.796	-33.3	0.042	0.009		
751081004	975.269	49.023	-12.7	0.001	0.002		
751081005	975.52	49.034	-13.2	0.001	0.002		
751081006	975.771	49.029	-11.3	0.003	0.004		
751081007	976.02	49.025	-12.7	0.001	0.002		
751081008	976.274	49.029	-13.6	0.002	0.008		
751081009	976.519	48.992	-11.4	0.004	0.02		
751081010	517.766	37.527	1.8	0.867	0.493		
751081011	518.268	37.534	11.3	0.48	0.495		
751081012	518.769	37.547	24.8	0.184	0.794		
751081013	728.96	51.378	5.4	0.072	0.239		
751081014	729.117	51.366	8.5	0.006	0.791		

751081015	729.274	51.365	6.7	0.046	0.321		
751081016	966.644	44.754	17.6	0.201	0.633		
751081017	966.843	44.751	11.9	0.269	0.472		
751081018	967.044	44.751	8.6	0.554	0.44		
751081019	967.245	44.752	7.7	0.607	0.412		
751081020	967.444	44.751	10	0.485	0.543		
751081021	967.646	44.751	7.3	0.186	0.482		
751081022	967.845	44.784	8.2	0.351	0.471		
751081023	703.919	36.144	8	0.726	0.515		
751081024	704.12	36.134	5.2	0.734	0.542		
751081025	704.321	36.144	3.5	0.817	0.487		
751081026	704.521	36.142	4.9	0.734	0.558		
751081027	704.721	36.146	4.3	0.778	0.543		
751081028	704.922	36.144	-10.5	0.404	0.315		
751081029	798.445	22.577	3.9	0.787	0.507		
751081030	799.448	22.58	1.8	0.873	0.299		
751081031	503.789	21.297	10.5	0.051	0.862		
751081032	504.29	21.303	10.9	0.036	0.527		
751081033	504.793	20.964	21.8	0.129	0.871		
751081034	926.8	45.493	1.6	0.821	0.716	Tax_Id=9606 Gene_Symbol=C4A;C4B complement component 4B preproprc	STQDTVIALDALSAYWIASHTTEER
751081035	927.134	45.494	1.8	0.81	0.776	Tax_Id=9606 Gene_Symbol=C4A;C4B complement component 4B preproprc	STQDTVIALDALSAYWIASHTTEER
751081036	927.468	45.493	2	0.791	0.834	Tax_Id=9606 Gene_Symbol=C4A;C4B complement component 4B preproprc	STQDTVIALDALSAYWIASHTTEER
751081037	927.803	45.492	1.4	0.868	0.8	Tax_Id=9606 Gene_Symbol=C4A;C4B complement component 4B preproprc	STQDTVIALDALSAYWIASHTTEER
751081038	928.137	45.494	0.1	0.985	0.622	Tax_Id=9606 Gene_Symbol=C4A;C4B complement component 4B preproprc	STQDTVIALDALSAYWIASHTTEER
751081039	708.368	34.338	0.5	0.964	0.388	Tax_Id=9606 Gene_Symbol=CNDP1 Beta-Ala-His dipeptidase	VFQYIDLHQDEFVQTLK
751081040	708.705	34.301	1.9	0.863	0.559	Tax_Id=9606 Gene_Symbol=CNDP1 Beta-Ala-His dipeptidase	VFQYIDLHQDEFVQTLK
751081041	709.04	34.297	4.1	0.691	0.651	Tax_Id=9606 Gene_Symbol=CNDP1 Beta-Ala-His dipeptidase	VFQYIDLHQDEFVQTLK
751081042	709.372	34.314	-2.4	0.873	0.156	Tax_Id=9606 Gene_Symbol=CNDP1 Beta-Ala-His dipeptidase	VFQYIDLHQDEFVQTLK
751081043	546.294	25.284	14.3	0.553	0.631		
751081044	547.297	25.381	35.3	0.082	0.852		
751081045	661.721	34.587	9.6	0.673	0.525		
751081046	661.921	34.592	10.1	0.534	0.681		
751081047	662.122	34.594	9.3	0.69	0.521		
751081048	458.606	15.233	2.8	0.695	0.194	Tax_Id=9606 Gene_Symbol=CLU Isoform 2 of Clusterin	KTLLSNLEEAKK
751081049	458.94	15.234	3.5	0.836	0.955	Tax_Id=9606 Gene_Symbol=CLU Isoform 2 of Clusterin	KTLLSNLEEAKK
751081050	459.275	15.232	-2.5	0.861	0.157	Tax_Id=9606 Gene_Symbol=CLU Isoform 2 of Clusterin	KTLLSNLEEAKK
751081051	605.399	66.073	2.8	0.715	0.318		
751081052	605.901	66.074	2.4	0.75	0.365		
751081053	606.403	66.071	2.7	0.711	0.335		
751081054	606.905	66.09	-21.2	0.454	0.841		
751081055	631.791	20.886	6.3	0.295	0.574		
751081056	632.293	20.887	5.9	0.339	0.549		
751081057	632.794	20.925	3.2	0.803	0.938		
751081058	955.293	41.41	11.6	0.3	0.803		
751081059	955.445	41.472	1.2	0.858	0.191		
751081060	955.628	41.413	6.7	0.526	0.569		
751081061	955.794	41.486	3	0.701	0.294		
751081062	955.95	41.426	5.6	0.602	0.581		
751081063	956.133	41.637	2.1	0.756	0.214		
751081064	956.295	41.427	-1.3	0.896	0.115		
751081065	559.63	21.047	9.2	0.06	0.933		
751081066	559.964	21.071	9	0.067	0.846		
751081067	733.386	26.737	10.8	0.124	0.994	Tax_Id=9606 Gene_Symbol=C3 Complement C3 (Fragment)	VPVAVQGEDTVQSLTQGDGVAK
751081068	733.721	26.747	13.2	0.077	0.784	Tax_Id=9606 Gene_Symbol=C3 Complement C3 (Fragment)	VPVAVQGEDTVQSLTQGDGVAK
751081069	734.055	26.74	10.3	0.142	0.965	Tax_Id=9606 Gene_Symbol=C3 Complement C3 (Fragment)	VPVAVQGEDTVQSLTQGDGVAK
751081070	734.375	26.686	1.7	0.684	0.374	Tax_Id=9606 Gene_Symbol=C3 Complement C3 (Fragment)	VPVAVQGEDTVQSLTQGDGVAK
751081071	544.77	17.026	0	0.998	0.168	Tax_Id=9606 Gene_Symbol=APLP1 Isoform 2 of Amyloid-like protein 1	DADTPMTLPK
751081072	545.27	17.064	3.9	0.792	0.581	Tax_Id=9606 Gene_Symbol=APLP1 Isoform 2 of Amyloid-like protein 1	DADTPMTLPK
751081073	576.516	44.75	9.7	0.143	0.856		
751081074	576.698	44.763	9.1	0.112	0.752		
751081075	576.88	44.769	8.4	0.129	0.744		
751081076	577.063	44.765	12.5	0.318	0.794		
751081077	467.22	15.751	2.7	0.694	0.299		
751081078	467.723	15.754	6.5	0.577	0.516		
751081079	728.706	29.541	-2.5	0.793	0.158		
751081080	728.906	29.538	1.2	0.9	0.607		
751081081	729.106	29.543	5.9	0.544	0.933		
751081082	729.307	29.55	0.6	0.927	0.416		
751081083	729.507	29.558	-1.3	0.927	0.414		
751081084	729.706	29.614	0.2	0.978	0.511		
751081085	864.387	66.764	1.2	0.886	0.682		
751081086	864.514	66.77	0.1	0.992	0.875		
751081087	864.638	66.748	1.4	0.873	0.633		
751081088	864.76	66.763	1.7	0.844	0.751		
751081089	864.887	66.775	-1	0.907	0.966		
751081090	578.806	23.776	7.1	0.431	0.956		
751081091	741.057	25.098	-21.4	0.256	0.405		
751081092	741.309	25.097	2.8	0.678	0.944		
751081093	741.559	25.113	0.8	0.926	0.733		
751081094	741.81	25.122	0.6	0.931	0.809		
751081095	742.062	25.135	11.9	0.265	0.596		
751081096	806.396	19.359	-2.4	0.631	0.354		
751081097	797.075	35.894	3.4	0.74	0.359	Tax_Id=9606 Gene_Symbol=GALNT10 Isoform 3 of Polypeptide N-acetylgala	SPPELVAEIVLVDDFS[166.998]DREHLKKPLEDY
751081098	797.242	35.912	6.4	0.674	0.58	Tax_Id=9606 Gene_Symbol=GALNT10 Isoform 3 of Polypeptide N-acetylgala	SPPELVAEIVLVDDFS[166.998]DREHLKKPLEDY
751081099	797.409	35.919	7.2	0.754	0.542	Tax_Id=9606 Gene_Symbol=GALNT10 Isoform 3 of Polypeptide N-acetylgala	SPPELVAEIVLVDDFS[166.998]DREHLKKPLEDY
751081100	797.575	35.931	2.9	0.611	0.403	Tax_Id=9606 Gene_Symbol=GALNT10 Isoform 3 of Polypeptide N-acetylgala	SPPELVAEIVLVDDFS[166.998]DREHLKKPLEDY
751081101	797.741	35.873	4.8	0.738	0.55	Tax_Id=9606 Gene_Symbol=GALNT10 Isoform 3 of Polypeptide N-acetylgala	SPPELVAEIVLVDDFS[166.998]DREHLKKPLEDY
751081102	797.908	35.938	1.4	0.786	0.308	Tax_Id=9606 Gene_Symbol=GALNT10 Isoform 3 of Polypeptide N-acetylgala	SPPELVAEIVLVDDFS[166.998]DREHLKKPLEDY
751081103	674.727	35.159	-4.1	0.363	0.076	Tax_Id=9606 Gene_Symbol=APP Isoform L-APP733 of Amyloid beta A4 prot	LALENYITALQAVPPRPR
751081104	675.061	35.175	-3.7	0.435	0.08	Tax_Id=9606 Gene_Symbol=APP Isoform L-APP733 of Amyloid beta A4 prot	LALENYITALQAVPPRPR
751081105	675.395	35.147	-4.2	0.428	0.114	Tax_Id=9606 Gene_Symbol=APP Isoform L-APP733 of Amyloid beta A4 prot	LALENYITALQAVPPRPR
751081106	675.732	35.081	-23.6	0.152	0.173	Tax_Id=9606 Gene_Symbol=APP Isoform L-APP733 of Amyloid beta A4 prot	LALENYITALQAVPPRPR
751081107	1137.006	48.295	2.2	0.739	0.841		

751081108	1137.256	48.292	3.7	0.54	0.515		
751081109	1137.506	48.292	2.8	0.653	0.573		
751081110	1137.757	48.291	2.5	0.698	0.33		
751081111	1138.007	48.294	1.7	0.78	0.499		
751081112	1138.256	48.299	4.3	0.465	0.756		
751081113	1138.507	48.298	5.3	0.351	0.745		
751081114	747.863	29.046	2.3	0.645	0.358		
751081115	793.031	28.703	-12.1	0.393	0.268		
751081116	793.367	28.694	-13.9	0.034	0.011		
751081117	793.701	28.7	-13	0.229	0.088		
751081118	794.035	28.707	-13	0.372	0.102		
751081119	385.873	19.684	0.3	0.951	0.394		
751081120	386.207	19.703	0.6	0.9	0.49		
751081121	386.542	19.725	-5.9	0.717	0.209		
751081122	1166.257	32.98	7.2	0.574	0.243		
751081123	1166.588	33.005	6	0.602	0.181		
751081124	1166.926	32.98	6.6	0.615	0.162		
751081125	1167.26	32.978	5.6	0.571	0.242		
751081126	1167.589	33.014	5.6	0.606	0.279		
751081127	1167.93	32.994	5.5	0.666	0.777		
751081128	900.316	49.917	3.2	0.645	0.645		
751081129	900.457	49.921	3.8	0.598	0.73		
751081130	900.601	49.928	5.5	0.453	0.72		
751081131	900.743	49.941	2.9	0.665	0.793		
751081132	900.887	49.974	0.4	0.948	0.113		
751081133	901.03	50.018	-3	0.713	0.103		
751081134	522.982	43.254	-33.2	0.216	0.298		
751081135	523.316	43.252	-32.8	0.225	0.298		
751081136	523.65	43.246	4.4	0.697	0.323		
751081137	523.985	43.249	23.5	0.367	0.964		
751081138	586.631	26.971	3.5	0.629	0.833		
751081139	586.965	26.972	2.9	0.629	0.889		
751081140	587.298	27.091	2.9	0.481	0.692		
751081141	810.122	28.858	-11.9	0.259	0.277		
751081142	810.372	28.964	-1.7	0.606	0.489		
751081143	810.623	28.917	-3.5	0.404	0.363		
751081144	810.873	28.919	-0.3	0.943	0.879		
751081145	811.124	28.984	-6.3	0.273	0.179		
751081146	798.908	44.887	8.6	0.29	0.502		
751081147	799.41	44.888	9.2	0.286	0.52		
751081148	799.911	44.889	9.8	0.169	0.52		
751081149	800.409	44.805	9	0.184	0.747		
751081150	1001.721	49.621	2	0.829	0.485		
751081151	1001.92	49.622	-0.3	0.973	0.425		
751081152	1002.122	49.627	1	0.919	0.373		
751081153	1002.322	49.623	0.9	0.924	0.322		
751081154	1002.522	49.619	-0.5	0.947	0.212		
751081155	830.897	21.327	1.7	0.756	0.656	Tax_Id=9606 Gene_Symbol=CST3 Cystatin-C	LVGGPM[147.0355]DASVEEEGVR
751081156	831.388	21.296	0.3	0.947	0.615	Tax_Id=9606 Gene_Symbol=CST3 Cystatin-C	LVGGPM[147.0355]DASVEEEGVR
751081157	831.903	21.394	-2.5	0.623	0.168	Tax_Id=9606 Gene_Symbol=CST3 Cystatin-C	LVGGPM[147.0355]DASVEEEGVR
751081158	832.4	21.424	-38.6	0.017	0.038	Tax_Id=9606 Gene_Symbol=CST3 Cystatin-C	LVGGPM[147.0355]DASVEEEGVR
751081159	548.939	26.649	5	0.337	0.854		
751081160	549.273	26.648	3	0.586	0.72		
751081161	549.607	26.648	4.5	0.448	0.88		
751081162	549.941	26.623	7.8	0.649	0.858		
751081163	593.33	17.621	-1.9	0.716	0.237		
751081164	593.831	17.62	-3.9	0.671	0.091		
751081165	594.335	17.675	-1.8	0.92	0.249		
751081166	611.768	24.895	9.7	0.686	0.238		
751081167	612.27	24.931	8.6	0.531	0.238		
751081168	612.776	25.004	4.1	0.589	0.264		
751081169	787.425	39.413	1.8	0.847	0.388	Tax_Id=9606 Gene_Symbol=TTR Transthyretin	YTIAALLSPYSYSTTAVVTNPK
751081170	787.759	39.403	5.7	0.393	0.555	Tax_Id=9606 Gene_Symbol=TTR Transthyretin	YTIAALLSPYSYSTTAVVTNPK
751081171	788.093	39.39	3.1	0.722	0.392	Tax_Id=9606 Gene_Symbol=TTR Transthyretin	YTIAALLSPYSYSTTAVVTNPK
751081172	788.417	39.384	4.9	0.286	0.557	Tax_Id=9606 Gene_Symbol=TTR Transthyretin	YTIAALLSPYSYSTTAVVTNPK
751081173	506.003	23.152	10.4	0.181	0.457		
751081174	506.254	23.152	10.3	0.304	0.415		
751081175	506.505	23.157	11.7	0.436	0.348		
751081176	506.755	23.149	31.4	0.058	0.806		
751081177	729.577	44.496	5.6	0.49	0.813		
751081178	729.778	44.493	5.5	0.519	0.755		
751081179	729.978	44.494	5.7	0.493	0.732		
751081180	730.179	44.493	5.2	0.522	0.861		
751081181	730.379	44.495	5	0.559	0.856		
751081182	730.58	44.496	5.8	0.516	0.677		
751081183	567.796	15.897	7.2	0.423	0.316		
751081184	568.303	15.948	-0.4	0.978	0.543		
751081185	712.956	21.58	7.4	0.245	0.678		
751081186	713.29	21.579	8.7	0.143	0.545		
751081187	713.624	21.588	9.9	0.133	0.506		
751081188	713.958	21.599	-2	0.808	0.22		
751081189	660.502	31.593	-6.3	0.648	0.061		
751081190	660.669	31.625	11.6	0.445	0.512		
751081191	660.836	31.629	14.8	0.53	0.586		
751081192	661.001	31.764	6.7	0.342	0.9		
751081193	661.17	31.588	11.6	0.599	0.451		
751081194	701.423	23.751	-1.3	0.881	0.592		
751081195	702.427	23.759	-6.4	0.298	0.056		
751081196	491.781	20.291	-0.6	0.893	0.092		
751081197	492.032	20.296	0.4	0.935	0.151		
751081198	492.283	20.309	0.1	0.983	0.152		
751081199	492.533	20.342	-3.6	0.586	0.042		
751081200	1062.555	18.606	3.2	0.457	0.724		

751081201	1063.557	18.608	0	0.999	0.547		
751081202	755.46	43.123	2.3	0.811	0.382		
751081203	755.961	43.116	3.4	0.621	0.464		
751081204	756.463	43.111	2.5	0.742	0.414		
751081205	756.965	43.149	-0.7	0.954	0.239		
751081206	673.673	31.986	2.4	0.66	0.582		
751081207	674.007	31.989	2.2	0.68	0.589		
751081208	674.343	31.721	0.6	0.967	0.411		
751081209	674.676	31.721	-12.9	0.471	0.306		
751081210	610.662	29.055	8.4	0.209	0.68	Tax_Id=9606 Gene_Symbol=GSN Isoform 1 of Gelsolin	QTQVSVLPEGGETPLFK
751081211	610.997	29.055	8.3	0.253	0.872	Tax_Id=9606 Gene_Symbol=GSN Isoform 1 of Gelsolin	QTQVSVLPEGGETPLFK
751081212	611.332	29.061	10.5	0.104	0.97	Tax_Id=9606 Gene_Symbol=GSN Isoform 1 of Gelsolin	QTQVSVLPEGGETPLFK
751081213	611.665	29.071	18.2	0.489	0.97	Tax_Id=9606 Gene_Symbol=GSN Isoform 1 of Gelsolin	QTQVSVLPEGGETPLFK
751081214	877.627	47.614	5.9	0.259	0.772		
751081215	877.808	47.612	7.2	0.159	0.886		
751081216	877.99	47.617	6.4	0.207	0.801		
751081217	878.171	47.623	8.6	0.088	0.935		
751081218	729.399	15.793	0.3	0.984	0.091		
751081219	730.403	15.798	-10.2	0.602	0.034		
751081220	1038.005	47.377	-0.7	0.914	0.52		
751081221	1038.256	47.39	-2.7	0.748	0.419		
751081222	1038.507	47.391	-2.4	0.711	0.363		
751081223	1038.757	47.389	-1.5	0.848	0.459		
751081224	1039.007	47.396	-1.1	0.875	0.23		
751081225	1039.261	47.391	-2.2	0.684	0.555		
751081226	984.002	32.246	4.8	0.403	0.923		
751081227	984.504	32.242	5.5	0.325	0.972		
751081228	985.005	32.298	3.4	0.498	0.786		
751081229	985.504	32.218	2.3	0.667	0.873		
751081230	509.251	16.409	-2.4	0.915	0.284		
751081231	509.753	16.415	-8	0.623	0.048		
751081232	533.273	19.315	0.1	0.988	0.386		
751081233	533.606	19.374	-0.2	0.966	0.343		
751081234	533.94	19.388	-10.7	0.282	0.27		
751081235	687.079	46.017	7.6	0.222	0.779		
751081236	687.336	45.951	8.5	0.211	0.523		
751081237	687.586	45.961	8.1	0.25	0.482		
751081238	687.839	45.768	7.6	0.255	0.787		
751081239	688.087	45.959	10	0.207	0.5		
751081240	565.546	26.852	3.7	0.444	0.76		
751081241	565.797	26.854	2.8	0.545	0.617		
751081242	566.047	26.868	0.9	0.818	0.47		
751081243	566.298	26.865	2.9	0.832	0.997		
751081244	619.294	15.742	5.9	0.076	0.723		
751081245	620.297	15.595	4.4	0.401	0.364		
751081246	714.01	28.338	-4	0.27	0.599		
751081247	715.013	28.354	-17.9	0.353	0.838		
751081248	629.02	30.744	-1.6	0.759	0.352	Tax_Id=9606 Gene_Symbol=CHL1 Isoform 1 of Neural cell adhesion molecul	LLLPPTESGSESSITILK
751081249	629.357	30.75	-1.9	0.689	0.336	Tax_Id=9606 Gene_Symbol=CHL1 Isoform 1 of Neural cell adhesion molecul	LLLPPTESGSESSITILK
751081250	629.689	30.698	-2.5	0.675	0.319	Tax_Id=9606 Gene_Symbol=CHL1 Isoform 1 of Neural cell adhesion molecul	LLLPPTESGSESSITILK
751081251	630.025	30.701	-3.1	0.73	0.181	Tax_Id=9606 Gene_Symbol=CHL1 Isoform 1 of Neural cell adhesion molecul	LLLPPTESGSESSITILK
751081252	456.762	15.193	8.8	0.173	0.761		
751081253	457.262	15.245	26	0.067	0.278		
751081254	457.765	15.206	98.2	0.016	0.015		
751081255	698.814	34.127	-3.9	0.477	0.093		
751081256	699.315	34.147	-2.7	0.649	0.131		
751081257	699.816	34.237	-2.1	0.799	0.629		
751081258	991.119	16.855	5.8	0.65	0.224		
751081259	991.453	16.855	7.4	0.588	0.248		
751081260	991.787	16.856	5.2	0.7	0.211		
751081261	992.121	16.857	-6.7	0.873	0.636		
751081262	992.456	16.855	-19.5	0.326	0.066		
751081263	557.301	36.855	-10.1	0.145	0.037		
751081264	557.552	36.848	1.7	0.904	0.432		
751081265	557.803	36.853	0.9	0.951	0.398		
751081266	558.054	36.863	-5.9	0.642	0.165		
751081267	603.294	18.813	4.9	0.497	0.718		
751081268	603.628	18.845	3.2	0.639	0.535		
751081269	603.963	18.842	-0.2	0.975	0.344		
751081270	616.31	39.654	1.6	0.857	0.905		
751081271	616.51	39.653	-0.8	0.903	0.621		
751081272	616.711	39.654	-1.2	0.856	0.595		
751081273	616.911	39.651	-0.9	0.907	0.717		
751081274	617.111	39.654	70.5	0.178	0.416		
751081275	591.913	20.264	1.7	0.748	0.491		
751081276	592.247	20.257	1.1	0.836	0.494		
751081277	592.581	20.267	4.6	0.729	0.534		
751081278	592.915	20.275	-6.1	0.616	0.166		
751081279	715.36	16.211	2.4	0.689	0.452		
751081280	715.862	16.206	4.7	0.48	0.623		
751081281	762.371	21.215	-2.8	0.528	0.046		
751081282	763.375	21.184	-13	0.208	0.035		
751081283	842.08	37.728	0.9	0.778	0.35		
751081284	842.414	37.605	1.4	0.736	0.493		
751081285	842.749	37.575	2.3	0.554	0.538		
751081286	843.083	37.541	1.8	0.691	0.634		
751081287	843.417	37.502	4.9	0.596	0.329		
751081288	816.893	17.156	10.2	0.352	0.633		
751081289	817.398	17.135	13.2	0.383	0.726		
751081290	817.9	17.153	11.1	0.491	0.67		
751081291	818.402	17.178	35.4	0.152	0.858		
751081292	576.607	44.768	10	0.134	0.778		
751081293	576.789	44.773	8.5	0.139	0.763		

751081294	576.971	44.768	12.4	0.354	0.751		
751081295	577.154	44.749	11.4	0.574	0.618		
751081296	465.234	14.589	5.1	0.524	0.531		
751081297	465.485	14.524	11.6	0.561	0.743		
751081298	465.736	14.514	19.4	0.345	0.529		
751081299	625.881	33.74	3.2	0.734	0.85		
751081300	626.38	33.586	7.6	0.361	0.557		
751081301	626.882	33.716	3.2	0.812	0.474		
751081302	834.402	17.143	13.2	0.417	0.687		
751081303	834.903	17.148	11.4	0.467	0.663		
751081304	835.403	17.141	22	0.258	0.845		
751081305	708.355	33.4	10.2	0.051	0.431		
751081306	708.861	33.51	10	0.058	0.435		
751081307	709.363	33.504	11.6	0.037	0.314		
751081308	536.766	29.121	4.8	0.577	0.743		
751081309	537.268	29.129	5.4	0.513	0.777		
751081310	537.769	29.158	-2	0.934	0.396		
751081311	661.011	37.233	8.8	0.584	0.155		
751081312	661.178	37.24	8.8	0.486	0.2		
751081313	661.345	37.242	9.8	0.344	0.741		
751081314	661.512	37.251	10.2	0.274	0.751		
751081315	661.679	37.261	9.1	0.352	0.288		
751081316	661.846	37.264	8.6	0.435	0.266		
751081317	662.013	37.259	3.7	0.561	0.439		
751081318	826.492	30.151	-6.2	0.171	0.02		
751081319	826.993	30.155	-6.1	0.159	0.014		
751081320	827.497	30.152	-6.6	0.115	0.008		
751081321	828.001	30.151	-6.3	0.574	0.215		
751081322	705.023	37.7	3.9	0.592	0.963		
751081323	705.357	37.673	3.3	0.601	1		
751081324	705.691	37.699	3	0.688	0.984		
751081325	864.451	66.757	-0.4	0.966	0.874		
751081326	864.577	66.756	1	0.911	0.701		
751081327	864.698	66.757	2.4	0.782	0.564		
751081328	864.824	66.774	0.9	0.917	0.722		
751081329	864.949	66.778	2	0.81	0.705		
751081330	1082.776	49.419	1.1	0.863	0.449		
751081331	528.292	24.379	1.6	0.912	0.157		
751081332	528.794	24.386	4.1	0.848	0.31		
751081333	561.93	15.666	-1.1	0.887	0.864		
751081334	562.265	15.66	-1.3	0.863	0.878		
751081335	562.599	15.665	-4	0.605	0.335		
751081336	562.933	15.679	-60.8	0.002	0.015		
751081337	587.295	26.485	2.4	0.384	0.11		
751081338	587.632	26.519	1.2	0.676	0.238		
751081339	587.966	26.463	3.5	0.327	0.081		
751081340	588.298	26.465	3.4	0.862	0.132		
751081341	951.227	33.346	6.7	0.665	0.48		
751081342	951.477	33.335	13.2	0.269	0.783		
751081343	951.729	33.359	11.1	0.297	0.829		
751081344	951.979	33.328	11.9	0.307	0.734		
751081345	952.23	33.36	12.2	0.274	0.741		
751081346	952.476	33.336	-14.7	0.062	0.009		
751081347	921.84	49.402	-2.7	0.802	0.058	Tax_Id=9606 Gene_Symbol=SCG3 Secretogranin-3	LLNLGLITESQAHTLEDEVAEVLQK
751081348	922.178	49.398	-7.7	0.602	0.046	Tax_Id=9606 Gene_Symbol=SCG3 Secretogranin-3	LLNLGLITESQAHTLEDEVAEVLQK
751081349	922.512	49.4	-3	0.831	0.086	Tax_Id=9606 Gene_Symbol=SCG3 Secretogranin-3	LLNLGLITESQAHTLEDEVAEVLQK
751081350	922.845	49.405	-3.5	0.726	0.056	Tax_Id=9606 Gene_Symbol=SCG3 Secretogranin-3	LLNLGLITESQAHTLEDEVAEVLQK
751081351	716.383	16.366	10.1	0.226	0.94		
751081352	717.389	16.373	-11.6	0.362	0.015		
751081353	1015.875	49.498	-0.9	0.873	0.114	Tax_Id=9606 Gene_Symbol=B3GNT1 N-acetyllactosaminide beta-1,3-N-acet	TTMDPNDVILATHASVDNLLHLSGLLER
751081354	1016.208	49.497	1	0.86	0.141	Tax_Id=9606 Gene_Symbol=B3GNT1 N-acetyllactosaminide beta-1,3-N-acet	TTMDPNDVILATHASVDNLLHLSGLLER
751081355	1016.542	49.504	0.1	0.982	0.156	Tax_Id=9606 Gene_Symbol=B3GNT1 N-acetyllactosaminide beta-1,3-N-acet	TTMDPNDVILATHASVDNLLHLSGLLER
751081356	1016.876	49.498	-0.5	0.913	0.082	Tax_Id=9606 Gene_Symbol=B3GNT1 N-acetyllactosaminide beta-1,3-N-acet	TTMDPNDVILATHASVDNLLHLSGLLER
751081357	830.439	17.493	0.9	0.924	0.192		
751081358	831.461	17.531	0.9	0.877	0.285		
751081359	1287.666	44.881	1	0.831	0.341		
751081360	1288.167	44.904	1.3	0.791	0.37		
751081361	1288.669	44.904	0.2	0.976	0.314		
751081362	1289.173	44.835	1.9	0.685	0.45		
751081363	1289.669	44.82	12.2	0.203	0.828		
751081364	464.281	25.461	-27.3	0.317	0.306		
751081365	464.783	25.465	5.4	0.647	0.297		
751081366	693.669	27.334	-1.5	0.784	0.532		
751081367	694.004	27.333	0.5	0.925	0.634		
751081368	694.338	27.34	-4.9	0.479	0.702		
751081369	694.669	27.332	-5.6	0.545	0.348		
751081370	813.599	66.727	2.3	0.781	0.665		
751081371	813.718	66.735	-1.2	0.885	0.88		
751081372	813.834	66.725	-0.5	0.952	0.767		
751081373	813.951	66.715	0.1	0.992	0.792		
751081374	814.07	66.707	1.3	0.879	0.704		
751081375	814.185	66.727	3.4	0.708	0.581		
751081376	478.354	66.221	4.4	0.571	0.264		
751081377	479.358	66.203	4.8	0.498	0.206		
751081378	1074.497	30.926	16.1	0.111	0.33		
751081379	1074.833	31.085	22.1	0.207	0.553		
751081380	1075.166	31.076	14.5	0.245	0.906		
751081381	1075.5	31.041	17	0.231	0.616		
751081382	1075.833	31.073	-2.2	0.87	0.1		
751081383	919.452	28.017	11	0.128	0.878	Tax_Id=9606 Gene_Symbol=GSN Isoform 1 of Gelsolin	TPSAAYLWVGTGASEAEK
751081384	919.967	28.028	10.3	0.133	0.996	Tax_Id=9606 Gene_Symbol=GSN Isoform 1 of Gelsolin	TPSAAYLWVGTGASEAEK
751081385	920.459	28.013	10.9	0.15	0.97	Tax_Id=9606 Gene_Symbol=GSN Isoform 1 of Gelsolin	TPSAAYLWVGTGASEAEK
751081386	920.963	27.999	-0.1	0.993	0.202	Tax_Id=9606 Gene_Symbol=GSN Isoform 1 of Gelsolin	TPSAAYLWVGTGASEAEK

751081387	711.832	24.065	3.6	0.545	0.52	Tax_Id=9606 Gene_Symbol=SEBOX cDNA FLJ51266, highly similar to Vitror	FEDGVLDPDYPR
751081388	712.33	24.129	3.5	0.564	0.543	Tax_Id=9606 Gene_Symbol=SEBOX cDNA FLJ51266, highly similar to Vitror	FEDGVLDPDYPR
751081389	712.838	24.093	4.9	0.705	0.838	Tax_Id=9606 Gene_Symbol=SEBOX cDNA FLJ51266, highly similar to Vitror	FEDGVLDPDYPR
751081390	598.487	34.851	7	0.649	0.376		
751081391	598.687	34.853	1	0.912	0.566		
751081392	598.888	34.85	4.8	0.682	0.369		
751081393	599.088	34.85	5.8	0.716	0.542		
751081394	599.289	34.855	4.7	0.832	0.432		
751081395	552.308	15.315	5.5	0.662	0.424	Tax_Id=9606 Gene_Symbol=A2M Alpha-2-macroglobulin	SSGSLLNNAIK
751081396	552.809	15.312	6.4	0.774	0.493	Tax_Id=9606 Gene_Symbol=A2M Alpha-2-macroglobulin	SSGSLLNNAIK
751081397	695.79	45.501	4.2	0.435	0.483		
751081398	695.934	45.502	3.4	0.52	0.494		
751081399	696.077	45.504	3	0.533	0.451		
751081400	696.22	45.503	4	0.453	0.61		
751081401	696.363	45.509	2.3	0.656	0.404		
751081402	696.506	45.502	2.9	0.582	0.483		
751081403	696.65	45.504	4.1	0.476	0.732		
751081404	409.52	17.672	12.6	0.2	0.752		
751081405	409.855	17.664	14.5	0.365	0.47		
751081406	410.189	17.627	21.3	0.248	0.905		
751081407	808.631	34.849	1.4	0.941	0.686		
751081408	808.881	34.867	-14.1	0.185	0.521		
751081409	809.132	34.886	-4.5	0.44	0.507		
751081410	809.382	34.91	-26.6	0.082	0.27		
751081411	809.633	34.928	-17.6	0.354	0.663		
751081412	728.345	33.343	-3.3	0.525	0.296		
751081413	728.679	33.353	-3.7	0.487	0.276		
751081414	729.013	33.35	-3	0.59	0.362		
751081415	729.348	33.368	-11.4	0.028	0.024		
751081416	924.699	37.694	3.1	0.748	0.236	Tax_Id=9606 Gene_Symbol=CNTN1 Isoform 1 of Contactin-1	YGHGVSEEDKGFGPIFEEQPINTIYPEESLEGK
751081417	924.949	37.684	2.5	0.761	0.316	Tax_Id=9606 Gene_Symbol=CNTN1 Isoform 1 of Contactin-1	YGHGVSEEDKGFGPIFEEQPINTIYPEESLEGK
751081418	925.201	37.699	1.6	0.803	0.249	Tax_Id=9606 Gene_Symbol=CNTN1 Isoform 1 of Contactin-1	YGHGVSEEDKGFGPIFEEQPINTIYPEESLEGK
751081419	925.451	37.696	2.3	0.692	0.266	Tax_Id=9606 Gene_Symbol=CNTN1 Isoform 1 of Contactin-1	YGHGVSEEDKGFGPIFEEQPINTIYPEESLEGK
751081420	925.702	37.704	3.6	0.78	0.409	Tax_Id=9606 Gene_Symbol=CNTN1 Isoform 1 of Contactin-1	YGHGVSEEDKGFGPIFEEQPINTIYPEESLEGK
751081421	925.953	37.708	1.7	0.912	0.767	Tax_Id=9606 Gene_Symbol=CNTN1 Isoform 1 of Contactin-1	YGHGVSEEDKGFGPIFEEQPINTIYPEESLEGK
751081422	614.841	18.483	0.8	0.881	0.183		
751081423	615.344	18.459	-1	0.842	0.139		
751081424	615.843	18.511	9.8	0.346	0.84		
751081425	956.685	35.791	2.6	0.591	0.855		
751081426	956.928	35.777	1.9	0.68	0.635		
751081427	957.186	35.797	3.6	0.479	0.919		
751081428	957.434	35.774	2.2	0.634	0.747		
751081429	957.687	35.809	2.7	0.574	0.642		
751081430	957.938	35.762	2.6	0.68	0.444		
751081431	517.266	21.31	8.4	0.291	0.446		
751081432	517.612	21.219	3.5	0.557	0.709		
751081433	517.947	21.219	2.3	0.67	0.814		
751081434	518.281	21.22	0.4	0.978	0.76		
751081435	1217.205	30.348	3.5	0.428	0.831		
751081436	1217.54	30.352	3.1	0.537	0.915		
751081437	1217.874	30.373	3.5	0.513	0.86		
751081438	1218.208	30.399	3.6	0.473	0.966		
751081439	1218.543	30.431	2.4	0.688	0.869		
751081440	1218.877	30.513	-6.4	0.579	0.177		
751081441	714.325	32.197	-4.2	0.35	0.152		
751081442	714.83	32.217	-4.3	0.324	0.105		
751081443	715.333	32.236	-6.2	0.209	0.042		
751081444	508.294	21.798	-4	0.571	0.041		
751081445	508.629	21.805	-1.3	0.882	0.132		
751081446	508.963	21.813	18.2	0.35	0.403		
751081447	984.524	37.431	-1.8	0.701	0.16	Tax_Id=9606 Gene_Symbol=APLP1 Isoform 2 of Amyloid-like protein 1	AALEGFLAALQADPPQAER
751081448	985.025	37.426	-1.6	0.716	0.128	Tax_Id=9606 Gene_Symbol=APLP1 Isoform 2 of Amyloid-like protein 1	AALEGFLAALQADPPQAER
751081449	985.526	37.449	-2.7	0.545	0.059	Tax_Id=9606 Gene_Symbol=APLP1 Isoform 2 of Amyloid-like protein 1	AALEGFLAALQADPPQAER
751081450	986.028	37.446	-5	0.495	0.089	Tax_Id=9606 Gene_Symbol=APLP1 Isoform 2 of Amyloid-like protein 1	AALEGFLAALQADPPQAER
751081451	766.153	46.781	-6	0.2	0.161	Tax_Id=9606 Gene_Symbol=B3GNT1 N-acetyllactosaminide beta-1,3-N-acet	TTM[147.0355]DPNDVILATHASVDNLLHLSGLLE
751081452	766.403	46.787	-5.5	0.233	0.289	Tax_Id=9606 Gene_Symbol=B3GNT1 N-acetyllactosaminide beta-1,3-N-acet	TTM[147.0355]DPNDVILATHASVDNLLHLSGLLE
751081453	766.654	46.787	-5.7	0.275	0.22	Tax_Id=9606 Gene_Symbol=B3GNT1 N-acetyllactosaminide beta-1,3-N-acet	TTM[147.0355]DPNDVILATHASVDNLLHLSGLLE
751081454	766.904	46.792	-5.5	0.257	0.241	Tax_Id=9606 Gene_Symbol=B3GNT1 N-acetyllactosaminide beta-1,3-N-acet	TTM[147.0355]DPNDVILATHASVDNLLHLSGLLE
751081455	767.155	46.793	-6.6	0.125	0.172	Tax_Id=9606 Gene_Symbol=B3GNT1 N-acetyllactosaminide beta-1,3-N-acet	TTM[147.0355]DPNDVILATHASVDNLLHLSGLLE
751081456	813.659	66.737	-1.3	0.88	0.785		
751081457	813.777	66.706	0.6	0.941	0.77		
751081458	813.891	66.721	-1.5	0.861	0.996		
751081459	814.01	66.706	0.3	0.971	0.876		
751081460	814.128	66.773	1.2	0.887	0.665		
751081461	662.106	36.979	2.7	0.424	0.345	Tax_Id=9606 Gene_Symbol=TTR Transthyretin	RYTIAALLSPYSYSTTAVVTNPKE
751081462	662.357	37.054	2.6	0.447	0.48	Tax_Id=9606 Gene_Symbol=TTR Transthyretin	RYTIAALLSPYSYSTTAVVTNPKE
751081463	662.607	37.004	3	0.326	0.484	Tax_Id=9606 Gene_Symbol=TTR Transthyretin	RYTIAALLSPYSYSTTAVVTNPKE
751081464	662.858	36.992	3.3	0.58	0.714	Tax_Id=9606 Gene_Symbol=TTR Transthyretin	RYTIAALLSPYSYSTTAVVTNPKE
751081465	663.109	36.974	0.7	0.935	0.278	Tax_Id=9606 Gene_Symbol=TTR Transthyretin	RYTIAALLSPYSYSTTAVVTNPKE
751081466	445.754	16.126	-13.6	0.711	0.818		
751081467	446.256	16.146	1.4	0.917	0.64		
751081468	637.301	23.334	8.9	0.054	0.673	Tax_Id=9606 Gene_Symbol=C4A;C4B complement component 4B preproprc	LLATLC[160.0302]SAEVC[160.0302]QC[160.0302
751081469	637.635	23.346	12.1	0.017	0.454	Tax_Id=9606 Gene_Symbol=C4A;C4B complement component 4B preproprc	LLATLC[160.0302]SAEVC[160.0302]QC[160.0302
751081470	637.967	23.325	11.4	0.307	0.318	Tax_Id=9606 Gene_Symbol=C4A;C4B complement component 4B preproprc	LLATLC[160.0302]SAEVC[160.0302]QC[160.0302
751081471	638.301	23.3	12.8	0.038	0.197	Tax_Id=9606 Gene_Symbol=C4A;C4B complement component 4B preproprc	LLATLC[160.0302]SAEVC[160.0302]QC[160.0302
751081472	549.271	18.781	4.8	0.358	0.852		
751081473	549.605	18.778	4.8	0.35	0.81		
751081474	549.939	18.769	5.3	0.381	0.86		
751081475	1055.206	40.574	5.4	0.692	0.513	Tax_Id=9606 Gene_Symbol=A2M Alpha-2-macroglobulin	YNILPEKEEFPFALGVQTLPQTC[160.0302]DEPK
751081476	1055.54	40.571	4.3	0.525	0.565	Tax_Id=9606 Gene_Symbol=A2M Alpha-2-macroglobulin	YNILPEKEEFPFALGVQTLPQTC[160.0302]DEPK
751081477	1055.875	40.58	2.3	0.687	0.407	Tax_Id=9606 Gene_Symbol=A2M Alpha-2-macroglobulin	YNILPEKEEFPFALGVQTLPQTC[160.0302]DEPK
751081478	1056.209	40.582	2.4	0.635	0.5	Tax_Id=9606 Gene_Symbol=A2M Alpha-2-macroglobulin	YNILPEKEEFPFALGVQTLPQTC[160.0302]DEPK
751081479	1056.545	40.589	-16.1	0.252	0.194	Tax_Id=9606 Gene_Symbol=A2M Alpha-2-macroglobulin	YNILPEKEEFPFALGVQTLPQTC[160.0302]DEPK

751081480	1056.881	40.582	-1.2	0.931	0.105	Tax_Id=9606 Gene_Symbol=A2M Alpha-2-macroglobulin	YNILPEKEEFPFALGVQTLPQTC[160.0302]DEPK
751081481	1046.227	34.699	5	0.185	0.543		
751081482	1046.477	34.707	4	0.253	0.777		
751081483	1046.728	34.697	2.5	0.441	0.929		
751081484	1046.978	34.701	3.8	0.309	0.966		
751081485	1047.229	34.697	1.9	0.619	0.744		
751081486	722.45	37.532	-1.4	0.817	0.447		
751081487	723.454	37.554	-0.9	0.915	0.267		
751081488	426.988	5.656	185.3	0.219	0.582		
751081489	545.621	35.262	2.6	0.606	0.424		
751081490	545.955	35.271	3.1	0.551	0.498		
751081491	546.289	35.278	-2.6	0.655	0.201		
751081492	584.768	17.176	-14.6	0.154	0.235		
751081493	585.018	17.181	-6.3	0.067	0.128		
751081494	585.269	17.152	-6.8	0.112	0.287		
751081495	585.52	17.17	-21.1	0.145	0.192		
751081496	709.36	15.927	14.4	0.468	0.498	Tax_Id=9606 Gene_Symbol=YIPF3 Protein YIPF3	QVADQMWQAGKR
751081497	709.861	15.955	9.7	0.664	0.454	Tax_Id=9606 Gene_Symbol=YIPF3 Protein YIPF3	QVADQMWQAGKR
751081498	710.361	15.973	-13.4	0.608	0.177	Tax_Id=9606 Gene_Symbol=YIPF3 Protein YIPF3	QVADQMWQAGKR
751081499	540.775	17.764	15.3	0.088	0.414		
751081500	541.275	17.798	17.6	0.409	0.753		
751081501	666.357	37.936	-5.4	0.45	0.152		
751081502	666.559	37.918	-1.1	0.946	0.291		
751081503	666.76	37.919	-1.8	0.844	0.201		
751081504	666.96	37.922	-7.7	0.513	0.064		
751081505	806.898	19.384	-2.5	0.587	0.302		
751081506	807.402	19.361	-2.1	0.667	0.327		
751081507	807.904	19.37	-6.3	0.321	0.138		
751081508	808.404	19.411	-11.9	0.447	0.431		
751081509	1178.764	41.331	21.4	0.316	0.884		
751081510	1178.965	41.327	6	0.513	0.46		
751081511	1179.166	41.332	6.9	0.48	0.481		
751081512	1179.365	41.345	4.6	0.615	0.364		
751081513	1179.561	41.422	4.5	0.528	0.359		
751081514	1179.77	41.328	13.6	0.382	0.47		
751081515	1058.812	16.706	-0.1	0.996	0.125		
751081516	1059.147	16.705	0	0.999	0.062		
751081517	1059.484	16.707	-0.5	0.962	0.123		
751081518	1059.815	16.707	-8.5	0.708	0.104		
751081519	1060.149	16.71	-31.4	0.08	0.024		
751081520	794.36	16.713	-0.5	0.982	0.245		
751081521	794.61	16.704	1	0.956	0.174		
751081522	794.861	16.708	1	0.952	0.157		
751081523	795.112	16.706	-0.2	0.993	0.192		
751081524	795.365	16.74	-17.4	0.255	0.033		
751081525	916.704	37.953	8.7	0.211	0.742		
751081526	916.955	37.948	10.5	0.155	0.641		
751081527	917.205	37.953	10.1	0.213	0.739		
751081528	917.456	37.965	8.8	0.26	0.797		
751081529	917.706	37.974	5.4	0.454	0.679		
751081530	917.957	37.996	12.9	0.398	0.246		
751081531	578.29	21.325	0.6	0.917	0.555		
751081532	578.792	21.387	-0.5	0.931	0.511		
751081533	1232.584	31.105	7.5	0.207	0.619		
751081534	1233.086	31.105	7.3	0.205	0.707		
751081535	1233.587	31.109	11.5	0.121	0.928		
751081536	1234.09	31.127	21.5	0.158	0.828		
751081537	753.683	47.199	8.6	0.129	0.884		
751081538	520.525	24.114	11	0.404	0.508		
751081539	801.386	33.072	3.6	0.679	0.375		
751081540	801.888	33.073	5.8	0.653	0.858		
751081541	894.091	29.874	-2.4	0.82	0.164		
751081542	894.424	29.88	-4.4	0.622	0.044		
751081543	894.759	29.884	-3	0.845	0.188		
751081544	895.094	29.883	-27.2	0.084	0.021		
751081545	648.339	36.062	2.7	0.622	0.774		
751081546	648.503	36.092	5.9	0.325	0.953		
751081547	648.67	36.093	7.9	0.236	0.795		
751081548	648.838	36.114	4.5	0.46	0.635		
751081549	649.003	36.118	18.1	0.083	0.516		
751081550	649.17	36.127	14.5	0.128	0.834		
751081551	910.648	47.313	-0.3	0.939	0.227		
751081552	910.815	47.36	5.3	0.42	0.71		
751081553	910.982	47.365	4.9	0.499	0.783		
751081554	911.149	47.37	5	0.445	0.852		
751081555	911.316	47.365	4.5	0.479	0.669		
751081556	911.486	47.379	2.5	0.649	0.515		
751081557	911.65	47.372	2.9	0.448	0.511		
751081558	911.819	47.378	17.4	0.048	0.331		
751081559	717.532	35.624	10.8	0.409	0.344		
751081560	717.698	35.627	14.1	0.537	0.481		
751081561	717.866	35.628	15.8	0.502	0.555		
751081562	718.033	35.628	8.6	0.214	0.588		
751081563	718.2	35.628	10.6	0.405	0.351		
751081564	718.367	35.625	10.1	0.431	0.382		
751081565	633.318	22.735	3.1	0.817	0.389	Tax_Id=9606 Gene_Symbol=CNDP1 Beta-Ala-His dipeptidase	TVFGTEPDMIR
751081566	633.818	22.861	8.5	0.442	0.338	Tax_Id=9606 Gene_Symbol=CNDP1 Beta-Ala-His dipeptidase	TVFGTEPDMIR
751081567	816.387	22.195	12	0.247	0.333		
751081568	366.201	14.842	6.7	0.652	0.326		
751081569	366.703	14.837	-0.1	0.993	0.153		
751081570	590.228	16.891	1.7	0.889	0.949		
751081571	590.73	16.895	-23.4	0.037	0.018		
751081572	948.085	46.997	3.6	0.541	0.724		

751081573	948.287	46.993	4.4	0.425	0.932		
751081574	948.487	47.015	4.4	0.419	0.914		
751081575	948.688	47.007	5.1	0.355	0.937		
751081576	948.889	47.068	4.8	0.366	0.89		
751081577	949.089	46.985	4	0.448	0.956		
751081578	949.289	46.97	10.9	0.126	0.406		
751081579	849.909	30.691	5.8	0.605	0.551		
751081580	850.41	30.77	-3.9	0.707	0.052		
751081581	850.91	30.726	0	1	0.201		
751081582	788.352	28.652	9.8	0.353	0.96		
751081583	788.605	28.643	-15.6	0.404	0.438		
751081584	788.855	28.639	3.1	0.654	0.316		
751081585	789.106	28.657	4.2	0.614	0.299		
751081586	789.355	28.663	8.1	0.425	0.996		
751081587	569.944	36.408	12.3	0.378	0.727		
751081588	570.279	36.405	6.4	0.362	0.767		
751081589	570.612	36.407	12.1	0.488	0.482		
751081590	570.947	36.405	-6.6	0.839	0.808		
751081591	600.973	23.335	5.6	0.121	0.498		
751081592	695.683	43.395	11	0.163	0.487		
751081593	696.017	43.394	10.7	0.177	0.456		
751081594	696.352	43.401	10	0.201	0.509		
751081595	696.69	43.393	11	0.06	0.934		
751081596	846.097	38.43	3.9	0.562	0.371	Tax_Id=9606 Gene_Symbol=LUM Lumican	NIPTVNENLENYYLEVNQLEK
751081597	846.431	38.426	3.3	0.59	0.524	Tax_Id=9606 Gene_Symbol=LUM Lumican	NIPTVNENLENYYLEVNQLEK
751081598	846.766	38.433	4.8	0.726	0.475	Tax_Id=9606 Gene_Symbol=LUM Lumican	NIPTVNENLENYYLEVNQLEK
751081599	847.1	38.447	2.6	0.784	0.311	Tax_Id=9606 Gene_Symbol=LUM Lumican	NIPTVNENLENYYLEVNQLEK
751081600	661.552	25.172	2.4	0.783	0.654		
751081601	661.804	25.169	2.5	0.639	0.694		
751081602	662.054	25.164	-20.4	0.188	0.233		
751081603	662.305	25.161	6.2	0.39	0.805		
751081604	662.555	25.185	6.9	0.782	0.608		
751081605	616.8	25.168	11.2	0.015	0.966		
751081606	617.302	25.166	8.1	0.07	0.884		
751081607	594.011	28.332	1.9	0.636	0.357	Tax_Id=9606 Gene_Symbol=A2M Alpha-2-macroglobulin	DTVIKPLLVEPEGLEK
751081608	594.343	28.342	1.1	0.821	0.354	Tax_Id=9606 Gene_Symbol=A2M Alpha-2-macroglobulin	DTVIKPLLVEPEGLEK
751081609	594.68	28.334	1	0.82	0.322	Tax_Id=9606 Gene_Symbol=A2M Alpha-2-macroglobulin	DTVIKPLLVEPEGLEK
751081610	595.015	28.313	-26.5	0.137	0.196	Tax_Id=9606 Gene_Symbol=A2M Alpha-2-macroglobulin	DTVIKPLLVEPEGLEK
751081611	868.695	35.202	4.4	0.45	0.62	Tax_Id=9606 Gene_Symbol=PEBP1 Phosphatidylethanolamine-binding prote	WSGPLSLQEVDEQPQHPLHVTYAGAAVDELGK
751081612	868.943	35.213	4.2	0.382	0.62	Tax_Id=9606 Gene_Symbol=PEBP1 Phosphatidylethanolamine-binding prote	WSGPLSLQEVDEQPQHPLHVTYAGAAVDELGK
751081613	869.192	35.225	4.1	0.407	0.727	Tax_Id=9606 Gene_Symbol=PEBP1 Phosphatidylethanolamine-binding prote	WSGPLSLQEVDEQPQHPLHVTYAGAAVDELGK
751081614	869.441	35.24	2.4	0.585	0.351	Tax_Id=9606 Gene_Symbol=PEBP1 Phosphatidylethanolamine-binding prote	WSGPLSLQEVDEQPQHPLHVTYAGAAVDELGK
751081615	869.691	35.243	1.1	0.838	0.148	Tax_Id=9606 Gene_Symbol=PEBP1 Phosphatidylethanolamine-binding prote	WSGPLSLQEVDEQPQHPLHVTYAGAAVDELGK
751081616	869.941	35.252	-4.1	0.856	0.911	Tax_Id=9606 Gene_Symbol=PEBP1 Phosphatidylethanolamine-binding prote	WSGPLSLQEVDEQPQHPLHVTYAGAAVDELGK
751081617	736.507	29.144	-7	0.105	0.036		
751081618	736.674	29.131	-4.6	0.526	0.377		
751081619	736.844	29.065	-3.9	0.575	0.379		
751081620	737.008	29.148	-5.7	0.391	0.267		
751081621	737.175	29.155	-6.6	0.329	0.212		
751081622	737.342	29.233	-8.6	0.487	0.237		
751081623	737.51	29.149	-21.1	0.074	0.108		
751081624	613.306	28.005	5.2	0.377	0.506		
751081625	613.641	28.008	6.2	0.28	0.376		
751081626	613.975	28.009	7.6	0.425	0.249		
751081627	558.292	33.776	4.9	0.456	0.74		
751081628	558.627	33.776	5.2	0.423	0.857		
751081629	558.961	33.783	8	0.295	0.976		
751081630	535.737	14.888	6.4	0.705	0.261		
751081631	536.239	14.886	-26.7	0.294	0.232		
751081632	536.74	14.892	6.3	0.774	0.885		
751081633	632.032	47.362	5.6	0.429	0.92		
751081634	632.366	47.366	1	0.778	0.722		
751081635	632.701	47.329	0.5	0.897	0.823		
751081636	633.036	47.278	5.5	0.333	0.795		
751081637	666.459	37.913	-1.4	0.928	0.211		
751081638	666.659	37.91	0	0.999	0.116		
751081639	666.86	37.926	-3.6	0.735	0.065		
751081640	667.061	37.922	-10.1	0.45	0.029		
751081641	681.028	28.318	6.2	0.201	0.79		
751081642	681.362	28.326	5.4	0.223	0.617		
751081643	681.696	28.324	6.2	0.193	0.88		
751081644	685.324	14.225	14.7	0.045	0.638		
751081645	686.327	14.219	13.1	0.056	0.775		
751081646	821.394	36.228	8.5	0.572	0.752	Tax_Id=9606 Gene_Symbol=HPX Hemopexin	SLGPNSC[160.0302]SANGPGLYLIHGPNLYC[16
751081647	821.643	36.3	9.7	0.255	0.723	Tax_Id=9606 Gene_Symbol=HPX Hemopexin	SLGPNSC[160.0302]SANGPGLYLIHGPNLYC[16
751081648	821.893	36.362	7	0.382	0.986	Tax_Id=9606 Gene_Symbol=HPX Hemopexin	SLGPNSC[160.0302]SANGPGLYLIHGPNLYC[16
751081649	822.145	36.442	6.4	0.33	0.766	Tax_Id=9606 Gene_Symbol=HPX Hemopexin	SLGPNSC[160.0302]SANGPGLYLIHGPNLYC[16
751081650	822.407	36.179	4.1	0.482	0.765	Tax_Id=9606 Gene_Symbol=HPX Hemopexin	SLGPNSC[160.0302]SANGPGLYLIHGPNLYC[16
751081651	822.646	36.271	13.2	0.677	0.656	Tax_Id=9606 Gene_Symbol=HPX Hemopexin	SLGPNSC[160.0302]SANGPGLYLIHGPNLYC[16
751081652	619.643	28.405	2.1	0.683	0.371		
751081653	619.977	28.393	0.2	0.977	0.131		
751081654	811.912	32.976	14.6	0.058	0.955		
751081655	812.166	32.976	12.8	0.095	0.841		
751081656	812.413	32.981	14.8	0.066	0.834		
751081657	812.667	32.983	-4.8	0.648	0.142		
751081658	812.916	32.986	2	0.799	0.175		
751081659	763.055	34.287	11.6	0.22	0.902		
751081660	763.39	34.295	12.3	0.182	0.97		
751081661	763.724	34.291	15.6	0.509	0.63		
751081662	764.058	34.293	2.5	0.826	0.452		
751081663	818.022	30.991	-9.3	0.277	0.051		
751081664	818.222	30.986	3.5	0.523	0.126		
751081665	818.422	30.973	8	0.59	0.444		

751081666	818.623	31.006	3.8	0.601	0.21		
751081667	818.824	31.006	5.9	0.771	0.304		
751081668	819.024	30.999	-26.9	0.248	0.062		
751081669	414.907	15.048	5.5	0.275	0.508		
751081670	415.242	15.048	6.8	0.282	0.547		
751081671	415.576	15.061	22.7	0.39	0.862		
751081672	486.914	18.121	10.3	0.038	0.521	Tax_Id=9606 Gene_Symbol=TPI1;TPI1P1 triosephosphate isomerase isoforr	HVFGESDELIGQK
751081673	487.248	18.132	7.9	0.101	0.881	Tax_Id=9606 Gene_Symbol=TPI1;TPI1P1 triosephosphate isomerase isoforr	HVFGESDELIGQK
751081674	487.582	18.132	7.5	0.222	0.856	Tax_Id=9606 Gene_Symbol=TPI1;TPI1P1 triosephosphate isomerase isoforr	HVFGESDELIGQK
751081675	719.325	30.025	2.5	0.753	0.443	Tax_Id=9606 Gene_Symbol=AGT Angiotensinogen	SLDFTELDVAAEK
751081676	719.827	30.026	3.6	0.685	0.492	Tax_Id=9606 Gene_Symbol=AGT Angiotensinogen	SLDFTELDVAAEK
751081677	720.329	30.029	1.2	0.862	0.518	Tax_Id=9606 Gene_Symbol=AGT Angiotensinogen	SLDFTELDVAAEK
751081678	626.782	23.86	20.3	0.051	0.543		
751081679	627.282	23.871	29.6	0.07	0.716		
751081680	627.785	23.872	0.1	0.996	0.314		
751081681	846.405	18.886	7.6	0.583	0.77		
751081682	847.409	18.902	7.5	0.832	0.89		
751081683	1127.819	37.398	1.5	0.753	0.181		
751081684	1128.152	37.183	2.2	0.587	0.361		
751081685	1128.486	37.199	2.1	0.611	0.38		
751081686	1128.82	37.209	2.4	0.55	0.383		
751081687	1129.155	37.165	3.1	0.393	0.593		
751081688	1129.493	36.887	-2.1	0.517	0.156		
751081689	799.384	26.89	0	0.999	0.116	Tax_Id=9606 Gene_Symbol=APLP1 Isoform 2 of Amyloid-like protein 1	RHQEAQEAC[160.0302]SSQGLILHGSGMLLPC[1
751081690	799.634	26.873	5.4	0.441	0.945	Tax_Id=9606 Gene_Symbol=APLP1 Isoform 2 of Amyloid-like protein 1	RHQEAQEAC[160.0302]SSQGLILHGSGMLLPC[1
751081691	799.885	26.872	2	0.759	0.72	Tax_Id=9606 Gene_Symbol=APLP1 Isoform 2 of Amyloid-like protein 1	RHQEAQEAC[160.0302]SSQGLILHGSGMLLPC[1
751081692	800.135	26.872	0	0.998	0.14	Tax_Id=9606 Gene_Symbol=APLP1 Isoform 2 of Amyloid-like protein 1	RHQEAQEAC[160.0302]SSQGLILHGSGMLLPC[1
751081693	800.386	26.842	-12.2	0.318	0.266	Tax_Id=9606 Gene_Symbol=APLP1 Isoform 2 of Amyloid-like protein 1	RHQEAQEAC[160.0302]SSQGLILHGSGMLLPC[1
751081694	800.637	26.835	0.9	0.963	0.48	Tax_Id=9606 Gene_Symbol=APLP1 Isoform 2 of Amyloid-like protein 1	RHQEAQEAC[160.0302]SSQGLILHGSGMLLPC[1
751081695	872.787	45.146	7.1	0.212	0.712		
751081696	873.107	45.38	6.9	0.254	0.624		
751081697	873.462	45.193	7	0.281	0.848		
751081698	873.797	45.191	5	0.598	0.296		
751081699	877.535	47.609	4.4	0.356	0.753		
751081700	877.718	47.614	6.6	0.201	0.989		
751081701	877.899	47.618	8	0.118	0.875		
751081702	878.083	47.616	5.3	0.265	0.688		
751081703	652.809	16.096	2.9	0.841	0.146		
751081704	653.309	16.106	3.7	0.866	0.414		
751081705	653.811	16.118	-19.4	0.553	0.213		
751081706	604.37	42.714	6.4	0.545	0.373		
751081707	604.704	42.713	13.3	0.568	0.598		
751081708	605.038	42.712	13.2	0.294	0.749		
751081709	748.017	37.018	7.9	0.52	0.266		
751081710	748.351	37.017	2.4	0.807	0.39		
751081711	748.685	37.02	3.3	0.709	0.328		
751081712	749.018	37.018	5	0.591	0.451		
751081713	719.608	39.835	3.9	0.792	0.177		
751081714	719.858	39.848	6.5	0.598	0.155		
751081715	720.11	39.844	5.7	0.632	0.152		
751081716	720.359	39.849	2.3	0.794	0.219		
751081717	720.611	39.844	5.5	0.66	0.256		
751081718	641.339	37.408	10.9	0.01	0.825		
751081719	641.675	37.461	10.7	0.025	0.789		
751081720	642.008	37.496	12.1	0.01	0.771		
751081721	642.343	37.521	1.9	0.853	0.399		
751081722	1027.48	30.806	2.3	0.852	0.953		
751081723	1027.728	30.788	5	0.283	0.476		
751081724	1027.978	30.798	6.8	0.17	0.362		
751081725	1028.229	30.805	2.2	0.663	0.998		
751081726	1028.479	30.8	-18.9	0.092	0.182		
751081727	715.865	28.311	-2.1	0.629	0.278	Tax_Id=9606 Gene_Symbol=CLSTN1 Isoform 2 of Calsyntenin-1 (Fragment)	EGLDLQVLEDSGR
751081728	716.366	28.308	-1.9	0.666	0.365	Tax_Id=9606 Gene_Symbol=CLSTN1 Isoform 2 of Calsyntenin-1 (Fragment)	EGLDLQVLEDSGR
751081729	716.867	28.369	-4.9	0.367	0.221	Tax_Id=9606 Gene_Symbol=CLSTN1 Isoform 2 of Calsyntenin-1 (Fragment)	EGLDLQVLEDSGR
751081730	526.988	15.573	0.9	0.967	0.216		
751081731	527.241	15.553	-1.1	0.94	0.203		
751081732	527.489	15.58	-2.8	0.888	0.171		
751081733	527.74	15.581	-4.7	0.628	0.132		
751081734	1091.494	26.974	1.1	0.861	0.486		
751081735	1091.744	26.982	2.1	0.719	0.571		
751081736	1091.996	26.988	1.9	0.75	0.588		
751081737	1092.246	27.003	1.1	0.826	0.409		
751081738	1092.497	26.988	-0.1	0.983	0.328		
751081739	1092.749	26.987	-1.9	0.782	0.282		
751081740	650.654	26.708	1.8	0.608	0.876		
751081741	650.988	26.702	3.6	0.329	0.961		
751081742	651.321	26.691	1.9	0.501	0.548		
751081743	651.651	26.742	-2.7	0.545	0.195		
751081744	747.335	17.98	7.6	0.326	0.579		
751081745	748.337	18.028	5.6	0.491	0.786		
751081746	837.721	24.597	-5.5	0.412	0.021		
751081747	838.055	24.603	-2	0.845	0.095		
751081748	838.39	24.613	-3.5	0.71	0.043		
751081749	838.724	24.616	-32.3	0.023	0.001		
751081750	481.254	18.109	6	0.16	0.874		
751081751	481.756	18.111	45.1	0.309	0.509		
751081752	650.316	14.282	10.5	0.253	0.632		
751081753	651.319	14.282	14.1	0.571	0.715		
751081754	652.322	14.283	-41.6	0.15	0.177		
751081755	555.265	28.043	5.2	0.737	0.135		
751081756	555.767	28.042	21.9	0.345	0.392		
751081757	459.715	16.904	-37.4	0.184	0.408		
751081758	460.217	16.918	2.4	0.901	0.936		

751081759	766.114	30.121	-1.1	0.877	0.436		
751081760	766.364	30.185	-2.2	0.668	0.157		
751081761	766.615	30.118	-0.2	0.966	0.275		
751081762	766.865	30.196	-2	0.773	0.478		
751081763	767.116	30.214	1.2	0.887	0.769		
751081764	767.366	30.306	-19.7	0.143	0.614		
751081765	506.933	14.938	8.6	0.664	0.345	Tax_Id=9606 Gene_Symbol=B2M Beta-2-microglobulin	IEKVEHSDLSFSK
751081766	507.267	14.938	10.1	0.638	0.429	Tax_Id=9606 Gene_Symbol=B2M Beta-2-microglobulin	IEKVEHSDLSFSK
751081767	507.601	14.939	1.6	0.921	0.369	Tax_Id=9606 Gene_Symbol=B2M Beta-2-microglobulin	IEKVEHSDLSFSK
751081768	744.594	43.038	5.8	0.37	0.943		
751081769	744.777	43.121	0	0.998	0.45		
751081770	744.959	43.069	-0.6	0.889	0.17		
751081771	1258.637	42.22	4.1	0.351	0.321		
751081772	1259.64	42.218	3.8	0.395	0.295		
751081773	1260.643	42.215	-0.1	0.988	0.202		
751081774	881.423	34.014	5.5	0.698	0.221		
751081775	881.758	34.01	9.4	0.678	0.445		
751081776	882.092	34.013	7.1	0.678	0.236		
751081777	882.425	34.01	6	0.61	0.196		
751081778	882.76	34.025	-1.6	0.938	0.26		
751081779	711.362	27.506	4.1	0.346	0.507		
751081780	711.861	27.491	4.5	0.43	0.657		
751081781	712.36	27.496	4.2	0.541	0.629		
751081782	632.337	21.845	1.1	0.776	0.277		
751081783	632.831	21.689	2.4	0.499	0.375		
751081784	633.33	21.714	3.5	0.735	0.466		
751081785	615.308	26.245	4.7	0.427	0.653		
751081786	615.642	26.238	4.1	0.449	0.618		
751081787	615.976	26.232	2.8	0.576	0.524		
751081788	616.308	26.225	5.3	0.334	0.733		
751081789	1013.443	14.712	3.8	0.568	0.711		
751081790	1013.944	14.732	-0.4	0.937	0.693		
751081791	1014.445	14.783	1.3	0.921	0.686		
751081792	1014.944	14.83	-0.8	0.974	0.587		
751081793	1042.017	31.241	4.5	0.293	0.964		
751081794	1042.52	31.305	6.9	0.113	0.78		
751081795	1043.022	31.27	1.4	0.585	0.49		
751081796	1043.522	31.291	-27.5	0.105	0.112		
751081797	426.464	21.451	-28.2	0.244	0.41		
751081798	426.715	21.46	4.7	0.397	0.898		
751081799	426.965	21.454	0.4	0.975	0.579		
751081800	427.216	21.448	-8.3	0.696	0.134		
751081801	885.936	40.35	0	0.994	0.261		
751081802	886.437	40.343	-0.1	0.987	0.245		
751081803	886.938	40.428	-2.5	0.716	0.033		
751081804	887.439	40.649	-0.9	0.896	0.163		
751081805	958.943	24.843	-14.1	0.001	0.001	Tax_Id=9606 Gene_Symbol=VGF Neurosecretory protein VGF	NSEPQDEGELFQGVDPR
751081806	959.444	24.961	-9.3	0.046	0.037	Tax_Id=9606 Gene_Symbol=VGF Neurosecretory protein VGF	NSEPQDEGELFQGVDPR
751081807	959.945	24.873	-42.5	0.002	0.006	Tax_Id=9606 Gene_Symbol=VGF Neurosecretory protein VGF	NSEPQDEGELFQGVDPR
751081808	910.809	48.217	2.8	0.532	0.334		
751081809	910.979	48.177	3.7	0.55	0.598		
751081810	911.147	48.176	3.1	0.493	0.507		
751081811	911.314	48.172	3.6	0.415	0.499		
751081812	911.482	48.168	3	0.493	0.388		
751081813	911.648	48.179	2.8	0.499	0.477		
751081814	911.815	48.174	6.1	0.288	0.612		
751081815	911.983	48.186	15.7	0.262	0.632		
751081816	577.276	66.095	-0.2	0.975	0.453		
751081817	578.28	66.092	-7.6	0.719	0.556		
751081818	552.627	25.065	5	0.281	0.918		
751081819	552.962	25.065	4.6	0.34	0.989		
751081820	553.295	25.047	3.2	0.593	0.485		
751081821	553.626	25.146	-1.9	0.925	0.754		
751081822	676.347	21.243	12.3	0.233	0.232		
751081823	676.85	21.248	4.5	0.611	0.285		
751081824	677.353	21.2	23.5	0.137	0.083		
751081825	563.271	24.528	2.3	0.655	0.401		
751081826	563.773	24.464	3.6	0.68	0.262		
751081827	399.777	20.969	9.5	0.514	0.695		
751081828	400.279	20.976	40.4	0.195	0.835		
751081829	625.355	36.655	8.4	0.356	0.735		
751081830	625.69	36.658	6.6	0.431	0.764		
751081831	626.024	36.655	8.5	0.263	0.627		
751081832	549.276	66.225	2.8	0.708	0.349		
751081833	550.28	66.203	2.6	0.709	0.261		
751081834	912.64	39.326	2.3	0.855	0.198		
751081835	912.841	39.325	-4.6	0.572	0.218		
751081836	913.043	39.326	-4	0.642	0.118		
751081837	913.242	39.32	-7.5	0.403	0.169		
751081838	913.443	39.369	-2.6	0.702	0.217		
751081839	913.644	39.326	-0.8	0.929	0.156		
751081840	714.354	22.065	-4.9	0.351	0.106		
751081841	714.69	22.097	0.3	0.969	0.408		
751081842	715.024	22.1	-2.8	0.849	0.8		
751081843	715.358	22.099	-3.8	0.786	0.581		
751081844	308.09	5.791	21.8	0.068	0.445		
751081845	309.093	5.792	5.9	0.795	0.357		
751081846	897.144	37.847	-2	0.754	0.266	Tax_Id=9606 Gene_Symbol=CLSTN1 Isoform 2 of Calsyntenin-1 (Fragment)	GVQIQAHPSQLVLTLEGEDLGELDK
751081847	897.48	37.865	-1.8	0.724	0.195	Tax_Id=9606 Gene_Symbol=CLSTN1 Isoform 2 of Calsyntenin-1 (Fragment)	GVQIQAHPSQLVLTLEGEDLGELDK
751081848	897.817	37.854	-2.1	0.684	0.145	Tax_Id=9606 Gene_Symbol=CLSTN1 Isoform 2 of Calsyntenin-1 (Fragment)	GVQIQAHPSQLVLTLEGEDLGELDK
751081849	898.153	37.821	-0.1	0.989	0.213	Tax_Id=9606 Gene_Symbol=CLSTN1 Isoform 2 of Calsyntenin-1 (Fragment)	GVQIQAHPSQLVLTLEGEDLGELDK
751081850	898.489	37.817	-3.9	0.321	0.075	Tax_Id=9606 Gene_Symbol=CLSTN1 Isoform 2 of Calsyntenin-1 (Fragment)	GVQIQAHPSQLVLTLEGEDLGELDK
751081851	699.36	26.882	-2.3	0.587	0.155		

751081852	699.694	26.896	-3.2	0.428	0.083		
751081853	700.029	26.899	-3.1	0.519	0.142		
751081854	471.739	20.226	13	0.091	0.978		
751081855	472.241	20.221	14.1	0.186	0.773		
751081856	848.147	28.917	2	0.689	0.35		
751081857	848.397	29.024	3.1	0.521	0.492		
751081858	848.647	29.156	2.3	0.646	0.404		
751081859	848.898	29.238	2.4	0.642	0.524		
751081860	849.148	29.221	1.7	0.87	0.777		
751081861	676.314	24.637	-10	0.294	0.628		
751081862	676.649	24.629	2.4	0.725	0.445		
751081863	676.984	24.594	-8.1	0.39	0.774		
751081864	688.881	44.005	8.4	0.177	0.988	Tax_Id=9606 Gene_Symbol=OMD Osteomodulin	LPNLLQLHLEHNNLEEFPFPLPK
751081865	689.132	44.001	6.4	0.249	0.806	Tax_Id=9606 Gene_Symbol=OMD Osteomodulin	LPNLLQLHLEHNNLEEFPFPLPK
751081866	689.382	44.012	8.3	0.204	0.998	Tax_Id=9606 Gene_Symbol=OMD Osteomodulin	LPNLLQLHLEHNNLEEFPFPLPK
751081867	689.633	44.024	9.3	0.501	0.652	Tax_Id=9606 Gene_Symbol=OMD Osteomodulin	LPNLLQLHLEHNNLEEFPFPLPK
751081868	689.884	44.037	6.1	0.111	0.648	Tax_Id=9606 Gene_Symbol=OMD Osteomodulin	LPNLLQLHLEHNNLEEFPFPLPK
751081869	645.998	22.808	8.1	0.436	0.44	Tax_Id=9606 Gene_Symbol=C4A;C4B complement component 4B preproprc	YLDKTEQWSTLPPETK
751081870	646.333	22.861	5.9	0.334	0.632	Tax_Id=9606 Gene_Symbol=C4A;C4B complement component 4B preproprc	YLDKTEQWSTLPPETK
751081871	646.667	22.835	4.6	0.614	0.403	Tax_Id=9606 Gene_Symbol=C4A;C4B complement component 4B preproprc	YLDKTEQWSTLPPETK
751081872	647.001	22.853	3.3	0.876	0.804	Tax_Id=9606 Gene_Symbol=C4A;C4B complement component 4B preproprc	YLDKTEQWSTLPPETK
751081873	560.805	18.519	1.4	0.879	0.468		
751081874	561.307	18.506	7.6	0.725	0.454		
751081875	797.731	36.485	5.8	0.519	0.612		
751081876	798.067	36.332	5.7	0.435	0.606		
751081877	798.401	36.422	6	0.624	0.357		
751081878	798.736	36.397	5.2	0.428	0.598		
751081879	378.238	15.543	4.1	0.852	0.483		
751081880	378.739	15.614	-3.3	0.802	0.103		
751081881	696.71	39.136	-2.9	0.826	0.226		
751081882	697.045	39.155	-1.8	0.81	0.076		
751081883	697.379	39.229	-3.5	0.789	0.24		
751081884	697.713	39.235	-3.5	0.826	0.559		
751081885	600.793	25.556	9.5	0.309	0.594		
751081886	601.294	25.694	7.9	0.298	0.755		
751081887	988.194	42.735	7.2	0.55	0.328		
751081888	988.445	42.731	8.9	0.28	0.803		
751081889	988.695	42.735	8.4	0.301	0.851		
751081890	988.945	42.731	8.5	0.278	0.878		
751081891	989.196	42.74	9.3	0.299	0.541		
751081892	989.448	42.739	7	0.445	0.508		
751081893	1185.519	45.058	2.5	0.664	0.92		
751081894	1185.665	45.101	3.6	0.59	0.971		
751081895	1185.807	45.127	4.1	0.52	0.881		
751081896	1185.949	45.064	3.6	0.561	0.869		
751081897	586.869	21.123	8.5	0.628	0.197		
751081898	587.07	21.123	8.3	0.425	0.297		
751081899	587.27	21.119	9.2	0.496	0.238		
751081900	587.47	21.126	7.8	0.647	0.139		
751081901	587.671	21.125	19.2	0.257	0.483		
751081902	702.873	30.675	1.1	0.844	0.621		
751081903	703.375	30.668	-0.5	0.92	0.198		
751081904	703.875	30.824	0.7	0.912	0.231		
751081905	753.899	20.172	-26.5	0.005	0.014		
751081906	754.149	20.182	-12	0.004	0.008		
751081907	754.4	20.183	-12.3	0.005	0.01		
751081908	754.651	20.197	-45	0	0.003		
751081909	754.902	20.213	-56	0.002	0.008		
751081910	416.22	20.295	9	0.324	0.606		
751081911	416.471	20.297	11.2	0.287	0.422		
751081912	416.722	20.298	5.8	0.71	0.584		
751081913	416.972	20.326	19.3	0.727	0.52		
751081914	1092.778	49.188	7.2	0.366	0.944		
751081915	1092.979	49.237	6	0.52	0.847		
751081916	1093.18	49.261	3.5	0.644	0.715		
751081917	1093.38	49.287	3	0.644	0.625		
751081918	1093.58	49.31	2.6	0.664	0.651		
751081919	1093.78	49.336	1.9	0.732	0.626		
751081920	744.855	42.849	11.7	0.123	0.585		
751081921	745.348	42.875	10.9	0.203	0.913		
751081922	604.814	21.588	1.1	0.8	0.29	Tax_Id=9606 Gene_Symbol=NCAM1 Isoform 2 of Neural cell adhesion mole(	GLGEISAASEFK
751081923	605.318	21.623	1.4	0.751	0.467	Tax_Id=9606 Gene_Symbol=NCAM1 Isoform 2 of Neural cell adhesion mole(	GLGEISAASEFK
751081924	605.817	21.619	-7.1	0.589	0.243	Tax_Id=9606 Gene_Symbol=NCAM1 Isoform 2 of Neural cell adhesion mole(	GLGEISAASEFK
751081925	605.827	15.42	5	0.667	0.689	Tax_Id=9606 Gene_Symbol=A2M Alpha-2-macroglobulin	LPPNVVEESAR
751081926	606.329	15.43	11.3	0.435	0.661	Tax_Id=9606 Gene_Symbol=A2M Alpha-2-macroglobulin	LPPNVVEESAR
751081927	675.516	38.951	-17.9	0.281	0.351		
751081928	675.716	38.98	1.1	0.859	0.733		
751081929	675.917	38.952	2	0.73	0.632		
751081930	676.118	38.943	2.5	0.709	0.824		
751081931	676.319	38.937	1.6	0.807	0.634		
751081932	676.52	38.936	5.9	0.589	0.264		
751081933	378.219	15.51	-2.7	0.852	0.284		
751081934	378.721	15.508	6	0.759	0.529		
751081935	1020.47	14.91	4.7	0.716	0.265		
751081936	1021.472	14.895	3.1	0.839	0.211		
751081937	1022.475	14.896	-36.5	0.193	0.036		
751081938	816.39	17.153	16	0.326	0.802		
751081939	594.075	24.599	29.3	0.041	0.482		
751081940	594.277	24.596	12.7	0.243	0.626		
751081941	594.476	24.595	28.1	0.124	0.926		
751081942	594.676	24.595	12.1	0.45	0.412		
751081943	594.876	24.599	55.5	0.134	0.584		
751081944	651.831	15.965	3.7	0.543	0.768		

751081945	949.48	42.26	-3.2	0.761	0.756		
751081946	949.814	42.26	-4.6	0.671	0.709		
751081947	950.149	42.278	-5.9	0.584	0.624		
751081948	950.482	42.286	-6.3	0.495	0.265		
751081949	950.816	42.307	-7.2	0.435	0.962		
751081950	901.387	50.755	-2.2	0.668	0.173		
751081951	901.459	50.768	1.4	0.832	0.651		
751081952	901.602	50.801	2.9	0.578	0.702		
751081953	901.743	50.775	2.7	0.619	0.66		
751081954	901.886	50.754	4.1	0.446	0.596		
751081955	687.662	20.2	-2.4	0.688	0.175		
751081956	687.996	20.205	-0.9	0.876	0.237		
751081957	688.331	20.225	-3.2	0.573	0.092		
751081958	715.865	29.677	6.4	0.415	0.305		
751081959	716.365	29.652	4.8	0.445	0.235		
751081960	716.866	29.669	2.5	0.823	0.459		
751081961	371.317	65.979	4.2	0.597	0.301		
751081962	372.32	65.938	6.2	0.4	0.182		
751081963	779.892	35.809	11.1	0.192	0.965		
751081964	780.141	35.797	7.5	0.599	0.543		
751081965	780.392	35.815	6.8	0.207	0.585		
751081966	780.642	35.803	9.6	0.38	0.672		
751081967	780.893	35.827	15.9	0.193	0.892		
751081968	653.379	35.171	-19.8	0.238	0.302		
751081969	653.881	35.179	1.1	0.881	0.299		
751081970	654.382	35.161	-10.3	0.528	0.156		
751081971	984.473	29.464	-3.5	0.422	0.102		
751081972	985.478	29.38	-4.4	0.371	0.067		
751081973	497.907	29.315	-2	0.827	0.268		
751081974	498.241	29.15	-0.8	0.927	0.302		
751081975	498.575	29.145	-25.7	0.331	0.254		
751081976	906.981	33.454	1.9	0.652	0.433		
751081977	907.482	33.463	2.4	0.558	0.44		
751081978	907.984	33.49	2.6	0.56	0.573		
751081979	908.486	33.526	-41.1	0.025	0.023		
751081980	730.343	14.989	5.4	0.713	0.276		
751081981	731.346	14.993	4.9	0.723	0.297		
751081982	544.308	31.413	6	0.438	0.919		
751081983	544.81	31.411	5	0.727	0.751		
751081984	664.343	17.236	-5.1	0.473	0.097		
751081985	664.845	17.245	-8.3	0.454	0.167		
751081986	880.445	26.461	2.3	0.818	0.565	Tax_Id=9606 Gene_Symbol=PTGDS Prostaglandin-H2 D-isomerase	TM[147.0355]LLQPAGSLGSYSYR
751081987	880.947	26.459	1.8	0.858	0.552	Tax_Id=9606 Gene_Symbol=PTGDS Prostaglandin-H2 D-isomerase	TM[147.0355]LLQPAGSLGSYSYR
751081988	881.446	26.479	9.5	0.606	0.316	Tax_Id=9606 Gene_Symbol=PTGDS Prostaglandin-H2 D-isomerase	TM[147.0355]LLQPAGSLGSYSYR
751081989	433.231	15.752	5.6	0.293	0.64		
751081990	433.559	15.702	5.8	0.155	0.931		
751081991	433.892	15.695	4.5	0.349	0.885		
751081992	708.717	34.973	-2	0.702	0.217		
751081993	709.05	34.96	-2	0.677	0.161		
751081994	709.384	34.946	-2.5	0.647	0.187		
751081995	709.718	34.923	-31.8	0.031	0.02		
751081996	971.604	23.552	-2.4	0.848	0.319		
751081997	971.772	23.557	-0.4	0.965	0.161		
751081998	971.939	23.557	-0.9	0.916	0.177		
751081999	972.107	23.564	-1.1	0.878	0.113		
751082000	972.273	23.566	-1.5	0.862	0.185		
751082001	972.441	23.575	-1.2	0.795	0.109		
751082002	972.608	23.572	-2.9	0.759	0.154		
751082003	972.776	23.556	-31.4	0.086	0.026		
751082004	631.795	16.803	8.5	0.699	0.559		
751082005	632.045	16.806	9	0.535	0.698		
751082006	632.296	16.806	5.1	0.524	0.647		
751082007	632.545	16.822	7	0.76	0.604		
751082008	632.792	16.846	-27.3	0.271	0.207		
751082009	875.457	28.775	-11.5	0.401	0.265		
751082010	621.328	43.516	1.6	0.733	0.513		
751082011	621.528	43.522	0.5	0.92	0.369		
751082012	621.729	43.525	2.1	0.664	0.646		
751082013	621.929	43.525	0.1	0.981	0.356		
751082014	622.13	43.513	0.1	0.978	0.393		
751082015	622.33	43.499	1.8	0.887	0.889		
751082016	1180.605	39.542	2.9	0.626	0.325	Tax_Id=9606 Gene_Symbol=TTR Transthyretin	YTIAALLSPYSYSTTAVVTNPK
751082017	1181.139	39.41	5	0.556	0.554	Tax_Id=9606 Gene_Symbol=TTR Transthyretin	YTIAALLSPYSYSTTAVVTNPK
751082018	1181.634	39.422	6.4	0.379	0.511	Tax_Id=9606 Gene_Symbol=TTR Transthyretin	YTIAALLSPYSYSTTAVVTNPK
751082019	1182.14	39.404	6.2	0.349	0.669	Tax_Id=9606 Gene_Symbol=TTR Transthyretin	YTIAALLSPYSYSTTAVVTNPK
751082020	1213.624	35.923	0	0.996	0.84		
751082021	1214.626	35.913	2.4	0.597	0.853		
751082022	645.381	34.222	-2.1	0.749	0.086		
751082023	645.883	34.25	-0.8	0.909	0.281		
751082024	646.385	34.268	-12.4	0.339	0.184		
751082025	456.713	18.827	1.9	0.759	0.392		
751082026	456.964	18.843	1.7	0.84	0.537		
751082027	457.214	18.787	6.5	0.652	0.687		
751082028	519.27	24.881	-1.3	0.769	0.499		
751082029	519.605	24.884	-2.9	0.521	0.366		
751082030	519.939	24.886	-0.3	0.971	0.354		
751082031	1019.448	26.939	-30	0.047	0.087	Tax_Id=9606 Gene_Symbol=CHGB Secretogranin-1	SAEFPDFYDSEEPVSTHQEAENEKDR
751082032	1019.783	26.952	-6.6	0.091	0.042	Tax_Id=9606 Gene_Symbol=CHGB Secretogranin-1	SAEFPDFYDSEEPVSTHQEAENEKDR
751082033	1020.117	26.961	-10.7	0.021	0.015	Tax_Id=9606 Gene_Symbol=CHGB Secretogranin-1	SAEFPDFYDSEEPVSTHQEAENEKDR
751082034	1020.452	26.974	-26.6	0.022	0.041	Tax_Id=9606 Gene_Symbol=CHGB Secretogranin-1	SAEFPDFYDSEEPVSTHQEAENEKDR
751082035	1020.787	26.969	-37.3	0.045	0.256	Tax_Id=9606 Gene_Symbol=CHGB Secretogranin-1	SAEFPDFYDSEEPVSTHQEAENEKDR
751082036	631.345	29.727	-1.7	0.905	0.264		
751082037	632.352	29.774	-13.2	0.523	0.231		

751082038	484.284	17.546	4.7	0.536	0.4		
751082039	484.785	17.543	7.3	0.422	0.14		
751082040	485.287	17.477	18.1	0.643	0.418		
751082041	743.342	33.545	10.9	0.442	0.508		
751082042	743.844	33.564	8.5	0.229	0.613		
751082043	744.346	33.558	10.2	0.379	0.344		
751082044	444.228	16.499	6.1	0.333	0.478		
751082045	444.479	16.503	6.1	0.545	0.585		
751082046	444.73	16.508	3.2	0.868	0.317		
751082047	805.391	33.158	3.8	0.446	0.198		
751082048	805.888	33.085	4.7	0.583	0.386		
751082049	806.389	33.076	2.5	0.717	0.394		
751082050	441.56	34.681	9.6	0.591	0.803		
751082051	441.894	34.683	8.1	0.717	0.732		
751082052	442.228	34.707	5.4	0.866	0.849		
751082053	601.971	21.161	10.1	0.172	0.408		
751082054	602.305	21.054	11.6	0.149	0.292		
751082055	602.639	21.306	11.3	0.228	0.364		
751082056	752.184	39.177	2.8	0.744	0.267		
751082057	752.383	39.211	1.8	0.804	0.694		
751082058	752.583	39.266	3.3	0.664	0.789		
751082059	752.783	39.293	2.7	0.705	0.665		
751082060	752.984	39.307	2.4	0.751	0.708		
751082061	753.184	39.288	4	0.557	0.864		
751082062	753.382	39.256	8.3	0.447	0.553		
751082063	655.91	43.186	2.3	0.87	0.296		
751082064	656.412	43.184	-32.5	0.119	0.112		
751082065	656.914	43.207	2	0.759	0.225		
751082066	773.369	22.168	5.1	0.444	0.763	Tax_Id=9606 Gene_Symbol=CLEC3B Putative uncharacterized protein DKF[illegible]	NWETEITAQPDGGK
751082067	773.869	22.169	7.4	0.592	0.265	Tax_Id=9606 Gene_Symbol=CLEC3B Putative uncharacterized protein DKF[illegible]	NWETEITAQPDGGK
751082068	774.37	22.193	8.3	0.402	0.533	Tax_Id=9606 Gene_Symbol=CLEC3B Putative uncharacterized protein DKF[illegible]	NWETEITAQPDGGK
751082069	798.476	17.997	11.9	0.586	0.766		
751082070	799.48	18.013	20.7	0.488	0.794		
751082071	800.352	21.516	6.1	0.515	0.73		
751082072	801.356	21.351	3.6	0.877	0.633		
751082073	1149.989	14.683	21	0.251	0.436		
751082074	1150.491	14.683	14.2	0.514	0.186		
751082075	1150.992	14.682	30.6	0.161	0.532		
751082076	1151.493	14.682	27.6	0.27	0.399		
751082077	1034.969	29.773	3.7	0.443	0.959		
751082078	1035.471	29.769	3.5	0.445	0.907		
751082079	1035.972	29.778	-1.5	0.862	0.749		
751082080	1036.476	29.768	23	0.318	0.822		
751082081	551.832	31.6	31.8	0.317	0.925		
751082082	552.333	31.583	27	0.395	0.219		
751082083	670.387	46.339	10	0.077	0.889		
751082084	913.153	30.348	3.7	0.519	0.947		
751082085	913.404	30.352	3.2	0.53	0.824		
751082086	913.654	30.385	2.9	0.644	0.741		
751082087	913.905	30.401	3.6	0.543	0.788		
751082088	914.156	30.459	0.2	0.98	0.426		
751082089	914.407	30.45	-19.2	0.31	0.396		
751082090	966.939	35.769	-1.3	0.816	0.139		
751082091	967.193	35.789	-0.9	0.823	0.379		
751082092	967.441	35.779	-0.5	0.939	0.248		
751082093	967.694	35.794	-1.4	0.739	0.342		
751082094	967.944	35.774	-3	0.456	0.104		
751082095	968.196	35.756	-1.8	0.813	0.164		
751082096	1016.467	45.152	8.5	0.59	0.833		
751082097	1016.716	45.151	1.7	0.923	0.477		
751082098	1016.968	45.147	0.4	0.977	0.479		
751082099	1017.219	45.152	1.6	0.905	0.427		
751082100	1017.469	45.152	-0.4	0.959	0.351		
751082101	1017.72	45.155	-3.8	0.808	0.245		
751082102	724.033	42.776	6.3	0.074	0.95	Tax_Id=9606 Gene_Symbol=OGN Osteoglycin	RLDFTGNLIEDIEDGTFSK
751082103	724.367	42.775	7	0.039	0.903	Tax_Id=9606 Gene_Symbol=OGN Osteoglycin	RLDFTGNLIEDIEDGTFSK
751082104	724.702	42.771	6.6	0.061	0.944	Tax_Id=9606 Gene_Symbol=OGN Osteoglycin	RLDFTGNLIEDIEDGTFSK
751082105	725.036	42.778	8.6	0.022	0.68	Tax_Id=9606 Gene_Symbol=OGN Osteoglycin	RLDFTGNLIEDIEDGTFSK
751082106	606.292	18.714	1.8	0.736	0.475		
751082107	997.573	48.132	6.5	0.276	0.702		
751082108	997.638	48.136	8.1	0.127	0.382		
751082109	997.766	48.113	8.9	0.157	0.911		
751082110	997.889	48.121	6	0.286	0.794		
751082111	998.017	48.086	5.7	0.314	0.375		
751082112	998.14	48.068	5	0.328	0.497		
751082113	586.487	34.369	10.3	0.427	0.586		
751082114	586.687	34.362	8	0.246	0.218		
751082115	586.888	34.364	13.5	0.072	0.982		
751082116	587.088	34.365	12.2	0.172	0.503		
751082117	995.11	42.742	3.7	0.693	0.353		
751082118	995.444	42.746	11.6	0.298	1		
751082119	995.778	42.749	10.7	0.292	0.893		
751082120	996.112	42.75	3.6	0.641	0.179		
751082121	1141.598	18.97	5.6	0.61	0.526	Tax_Id=9606 Gene_Symbol=HPX Hemopexin	GGYTLVSGYPK
751082122	1142.601	18.973	6.5	0.569	0.594	Tax_Id=9606 Gene_Symbol=HPX Hemopexin	GGYTLVSGYPK
751082123	1143.603	18.967	2.2	0.947	0.965	Tax_Id=9606 Gene_Symbol=HPX Hemopexin	GGYTLVSGYPK
751082124	901.532	50.77	-1.3	0.792	0.231		
751082125	901.671	50.779	0.8	0.872	0.33		
751082126	901.815	50.79	-0.6	0.907	0.316		
751082127	619.278	23.191	6.9	0.215	0.47		
751082128	619.526	23.179	8.4	0.632	0.237		
751082129	619.777	23.176	7.4	0.464	0.305		
751082130	620.027	23.182	8.8	0.527	0.182		

751082131	620.285	23.165	11	0.469	0.59		
751082132	1003.203	40.071	-1	0.748	0.216		
751082133	1003.453	40.113	-0.3	0.933	0.229		
751082134	1003.704	40.146	0.6	0.852	0.47		
751082135	1003.954	40.144	1.1	0.733	0.662		
751082136	1004.206	40.11	0.1	0.986	0.345		
751082137	1004.457	40.051	-1.8	0.567	0.229		
751082138	587.342	15.199	-7.3	0.732	0.221		
751082139	588.346	15.179	-33.7	0.14	0.06		
751082140	509.596	22.391	13	0.23	0.353		
751082141	509.93	22.4	11.3	0.521	0.303		
751082142	510.265	22.497	6.9	0.268	0.196		
751082143	729.427	28.415	-1.9	0.64	0.101	Tax_Id=9606 Gene_Symbol=NEGR1 cDNA FLJ59691, highly similar to Neur	VVVNFAPTIQEIK
751082144	729.929	28.415	-3.1	0.458	0.063	Tax_Id=9606 Gene_Symbol=NEGR1 cDNA FLJ59691, highly similar to Neur	VVVNFAPTIQEIK
751082145	730.431	28.421	-4.4	0.724	0.88	Tax_Id=9606 Gene_Symbol=NEGR1 cDNA FLJ59691, highly similar to Neur	VVVNFAPTIQEIK
751082146	860.408	28.961	-0.8	0.959	0.284	Tax_Id=9606 Gene_Symbol=CHGB Secretogranin-1	ERADEPQWSLYPSDSQVSEEVK
751082147	860.743	28.972	-0.5	0.969	0.174	Tax_Id=9606 Gene_Symbol=CHGB Secretogranin-1	ERADEPQWSLYPSDSQVSEEVK
751082148	861.077	28.982	-2.3	0.885	0.418	Tax_Id=9606 Gene_Symbol=CHGB Secretogranin-1	ERADEPQWSLYPSDSQVSEEVK
751082149	861.411	28.995	5.7	0.816	0.328	Tax_Id=9606 Gene_Symbol=CHGB Secretogranin-1	ERADEPQWSLYPSDSQVSEEVK
751082150	737.836	18.542	4.9	0.5	0.548		
751082151	738.338	18.536	-25.1	0.06	0.008		
751082152	738.839	18.543	-5.2	0.82	0.18		
751082153	684.157	37.939	5	0.376	0.525		
751082154	684.323	37.969	5.7	0.258	0.544		
751082155	684.49	37.987	5.4	0.258	0.486		
751082156	684.657	37.995	8.1	0.075	0.627		
751082157	684.825	37.994	9.7	0.446	0.653		
751082158	684.992	37.999	6.9	0.707	0.978		
751082159	760.871	18.192	4.8	0.39	0.825	Tax_Id=9606 Gene_Symbol=TIMP2 Metalloproteinase inhibitor 2	EVDSGNDIYGNPIK
751082160	761.372	18.2	0.9	0.879	0.33	Tax_Id=9606 Gene_Symbol=TIMP2 Metalloproteinase inhibitor 2	EVDSGNDIYGNPIK
751082161	761.872	18.203	-4.6	0.868	0.82	Tax_Id=9606 Gene_Symbol=TIMP2 Metalloproteinase inhibitor 2	EVDSGNDIYGNPIK
751082162	782.902	23.225	-8.7	0.026	0.009		
751082163	783.402	23.21	-8.2	0.021	0.003		
751082164	783.904	23.231	-34.5	0.018	0.061		
751082165	480.81	21.301	11.8	0.212	0.506		
751082166	481.01	21.3	13.5	0.345	0.596		
751082167	481.211	21.299	13.9	0.333	0.662		
751082168	481.411	21.306	24.4	0.272	0.29		
751082169	481.612	21.303	23.8	0.507	0.213		
751082170	472.949	24.118	9.8	0.489	0.342	Tax_Id=9606 Gene_Symbol=A2M Alpha-2-macroglobulin	MVSGFIPLKPTVK
751082171	473.284	24.116	17.4	0.287	0.517	Tax_Id=9606 Gene_Symbol=A2M Alpha-2-macroglobulin	MVSGFIPLKPTVK
751082172	473.618	24.116	44.4	0.209	0.701	Tax_Id=9606 Gene_Symbol=A2M Alpha-2-macroglobulin	MVSGFIPLKPTVK
751082173	923.883	46.071	10.7	0.116	0.814	Tax_Id=9606 Gene_Symbol=C4A;C4B complement component 4B preproprc	LLLFSPSVVHLGVPLSVGVQLQDVPR
751082174	924.216	46.055	9.5	0.155	0.824	Tax_Id=9606 Gene_Symbol=C4A;C4B complement component 4B preproprc	LLLFSPSVVHLGVPLSVGVQLQDVPR
751082175	924.551	46.062	8.8	0.181	0.744	Tax_Id=9606 Gene_Symbol=C4A;C4B complement component 4B preproprc	LLLFSPSVVHLGVPLSVGVQLQDVPR
751082176	924.886	46.064	9.1	0.195	0.829	Tax_Id=9606 Gene_Symbol=C4A;C4B complement component 4B preproprc	LLLFSPSVVHLGVPLSVGVQLQDVPR
751082177	978.928	35.379	5.8	0.05	0.757		
751082178	979.18	35.449	5.5	0.109	0.731		
751082179	979.429	35.235	7	0.069	0.744		
751082180	979.68	35.395	6.5	0.065	0.701		
751082181	979.93	35.216	5.9	0.162	0.58		
751082182	980.182	35.297	5.3	0.195	0.74		
751082183	542.581	33.06	7.4	0.366	0.392		
751082184	542.915	33.06	10.2	0.283	0.445		
751082185	543.249	33.069	15.8	0.33	0.483		
751082186	604.812	31.049	26.8	0.274	0.893		
751082187	605.063	31.048	20	0.222	0.618		
751082188	605.308	31.079	2	0.787	0.231		
751082189	605.565	31.057	10.6	0.765	0.496		
751082190	452.039	22.1	-28.9	0.201	0.284		
751082191	452.24	22.1	-4	0.698	0.358		
751082192	452.441	22.098	-29.6	0.178	0.252		
751082193	452.641	22.097	21.5	0.555	0.996		
751082194	619.969	18.668	7.6	0.286	0.916		
751082195	620.303	18.511	11.4	0.105	0.75		
751082196	620.638	18.531	6.3	0.447	0.646		
751082197	652.03	24.6	3.4	0.459	0.668		
751082198	652.365	24.599	4	0.404	0.831		
751082199	652.699	24.598	3.7	0.474	0.723		
751082200	653.033	24.612	19	0.357	0.781		
751082201	751.329	15.294	-0.9	0.892	0.239	Tax_Id=9606 Gene_Symbol=SCG5 Isoform 1 of Neuroendocrine protein 7B2	SVPHFSDEDKDPE
751082202	751.831	15.265	0.7	0.958	0.318	Tax_Id=9606 Gene_Symbol=SCG5 Isoform 1 of Neuroendocrine protein 7B2	SVPHFSDEDKDPE
751082203	752.333	15.265	7	0.514	0.695	Tax_Id=9606 Gene_Symbol=SCG5 Isoform 1 of Neuroendocrine protein 7B2	SVPHFSDEDKDPE
751082204	546.264	18.604	-1.8	0.726	0.23		
751082205	546.765	18.597	-6.9	0.253	0.036		
751082206	547.267	18.548	-35.2	0.068	0.127		
751082207	452.582	16.176	10.3	0.481	0.728		
751082208	452.916	16.167	11.6	0.439	0.806		
751082209	453.25	16.169	24.3	0.116	0.708		
751082210	675.81	26.763	11.8	0.337	0.512		
751082211	676.312	26.787	6.3	0.387	0.938		
751082212	676.813	26.832	4.5	0.559	0.896		
751082213	748.366	28.924	4.2	0.303	0.657		
751082214	748.866	28.867	5.4	0.307	0.495		
751082215	749.368	28.855	0.7	0.955	0.342		
751082216	590.691	42.274	-1.3	0.872	0.755		
751082217	590.891	42.257	1.1	0.897	0.976		
751082218	591.09	42.258	-1	0.939	0.683		
751082219	591.291	42.282	-8.4	0.327	0.584		
751082220	591.492	42.258	-16.3	0.355	0.903		
751082221	595.81	20.574	0.1	0.975	0.344		
751082222	663.457	66.432	NA	NA	NA		
751082223	664.461	66.437	5.5	0.461	0.233		

751082224	656.503	27.263	8.8	0.446	0.991		
751082225	656.703	27.269	-15.9	0.382	0.396		
751082226	656.904	27.271	-15	0.401	0.403		
751082227	657.104	27.275	4	0.7	0.858		
751082228	657.305	27.364	4	0.558	0.589		
751082229	657.505	27.256	9.2	0.688	0.751		
751082230	704.877	27.174	-3	0.657	0.251	Tax_Id=9606 Gene_Symbol=MCAM Isoform 1 of Cell surface glycoprotein M	EVTVPVFYPTEK
751082231	705.379	27.172	-3.9	0.5	0.161	Tax_Id=9606 Gene_Symbol=MCAM Isoform 1 of Cell surface glycoprotein M	EVTVPVFYPTEK
751082232	705.88	27.181	-5.3	0.362	0.135	Tax_Id=9606 Gene_Symbol=MCAM Isoform 1 of Cell surface glycoprotein M	EVTVPVFYPTEK
751082233	822.018	24.825	4.2	0.751	0.172		
751082234	822.353	24.819	6.5	0.587	0.26		
751082235	822.686	24.825	4.6	0.594	0.221		
751082236	823.02	24.827	10.6	0.499	0.332		
751082237	823.353	24.808	-1	0.934	0.39		
751082238	772.679	51.343	5.1	0.088	0.774		
751082239	772.847	51.406	7.9	0.021	0.816		
751082240	773.013	51.33	5.8	0.191	0.985		
751082241	1023.995	24.811	-5.5	0.708	0.15	Tax_Id=9606 Gene_Symbol=SPARCL1 cDNA FLJ52396, highly similar to SF	TVSEALLMEPTDDGNTTPR
751082242	1024.497	24.807	-10.1	0.373	0.113	Tax_Id=9606 Gene_Symbol=SPARCL1 cDNA FLJ52396, highly similar to SF	TVSEALLMEPTDDGNTTPR
751082243	1025.001	24.816	-4.1	0.639	0.166	Tax_Id=9606 Gene_Symbol=SPARCL1 cDNA FLJ52396, highly similar to SF	TVSEALLMEPTDDGNTTPR
751082244	1025.497	24.813	-17.8	0.229	0.082	Tax_Id=9606 Gene_Symbol=SPARCL1 cDNA FLJ52396, highly similar to SF	TVSEALLMEPTDDGNTTPR
751082245	485.731	15.207	2.3	0.694	0.312		
751082246	486.232	15.195	1.3	0.847	0.195		
751082247	635.787	15.488	20.9	0.186	0.73	Tax_Id=9606 Gene_Symbol=FBLN1 Isoform C of Fibulin-1	DC[160.0302]SLPYATESK
751082248	636.287	15.476	10.2	0.198	0.934	Tax_Id=9606 Gene_Symbol=FBLN1 Isoform C of Fibulin-1	DC[160.0302]SLPYATESK
751082249	633.288	22.246	21	0.042	0.23		
751082250	633.622	22.248	10.6	0.481	0.27		
751082251	633.956	22.25	4.1	0.76	0.101		
751082252	634.294	22.308	-3	0.753	0.03		
751082253	734.366	27.791	2.3	0.78	0.237	Tax_Id=9606 Gene_Symbol=TIMP1 Metalloproteinase inhibitor 1	LQSGTHC[160.0302]LWTDQLLQGSEK
751082254	734.7	27.788	5.5	0.595	0.293	Tax_Id=9606 Gene_Symbol=TIMP1 Metalloproteinase inhibitor 1	LQSGTHC[160.0302]LWTDQLLQGSEK
751082255	735.033	27.795	5.3	0.598	0.269	Tax_Id=9606 Gene_Symbol=TIMP1 Metalloproteinase inhibitor 1	LQSGTHC[160.0302]LWTDQLLQGSEK
751082256	735.357	27.765	5.2	0.679	0.608	Tax_Id=9606 Gene_Symbol=TIMP1 Metalloproteinase inhibitor 1	LQSGTHC[160.0302]LWTDQLLQGSEK
751082257	948.471	34.466	4.8	0.685	0.148	Tax_Id=9606 Gene_Symbol=GSN Isoform 1 of Gelsolin	NWRDPDQTDGLGLSYLSSHIANVER
751082258	948.807	34.49	5.5	0.622	0.144	Tax_Id=9606 Gene_Symbol=GSN Isoform 1 of Gelsolin	NWRDPDQTDGLGLSYLSSHIANVER
751082259	949.14	34.463	10.7	0.633	0.459	Tax_Id=9606 Gene_Symbol=GSN Isoform 1 of Gelsolin	NWRDPDQTDGLGLSYLSSHIANVER
751082260	949.473	34.47	11.1	0.6	0.4	Tax_Id=9606 Gene_Symbol=GSN Isoform 1 of Gelsolin	NWRDPDQTDGLGLSYLSSHIANVER
751082261	949.81	34.455	-5.7	0.795	0.151	Tax_Id=9606 Gene_Symbol=GSN Isoform 1 of Gelsolin	NWRDPDQTDGLGLSYLSSHIANVER
751082262	763.12	36.338	1.9	0.892	0.201		
751082263	763.37	36.307	2.9	0.791	0.305		
751082264	763.621	36.34	3.1	0.886	0.382		
751082265	763.872	36.353	5.4	0.81	0.469		
751082266	764.124	36.359	-13	0.369	0.359		
751082267	579.299	18.9	2.4	0.524	0.948		
751082268	579.8	18.965	4.3	0.352	0.883		
751082269	605.325	22.537	11.3	0.159	0.55		
751082270	605.829	22.549	7.6	0.245	0.699		
751082271	606.334	22.413	-1.7	0.92	0.809		
751082272	701.426	31.579	12	0.332	0.934	Tax_Id=9606 Gene_Symbol=C3 Complement C3 (Fragment)	SSLSVPYVIVPLK
751082273	701.929	31.553	19.2	0.12	0.834	Tax_Id=9606 Gene_Symbol=C3 Complement C3 (Fragment)	SSLSVPYVIVPLK
751082274	678.699	39.122	13.7	0.049	0.807		
751082275	679.032	39.132	17.8	0.03	0.445		
751082276	679.368	39.134	15.4	0.073	0.901		
751082277	679.702	39.132	48.3	0.048	0.247		
751082278	674.354	26.194	-3	0.783	0.044		
751082279	675.356	26.224	-17.6	0.122	0.009		
751082280	606.755	25.337	-7.4	0.221	0.155		
751082281	607.256	25.347	-8.4	0.251	0.194		
751082282	607.758	25.37	-38	0.094	0.108		
751082283	725.884	24.654	42.9	0.009	0.13		
751082284	726.385	24.64	29.1	0.014	0.156		
751082285	726.887	24.649	19.5	0.034	0.14		
751082286	1164.117	46.243	3.2	0.673	0.461		
751082287	1164.618	46.262	3.3	0.585	0.458		
751082288	1165.12	46.242	3.8	0.79	0.528		
751082289	1165.614	46.066	22.3	0.298	0.821		
751082290	451.709	16.787	7.1	0.459	0.515		
751082291	452.211	16.816	7.3	0.678	0.607		
751082292	549.451	21.195	3.6	0.687	0.19		
751082293	1207.873	31.159	5.8	0.209	0.322		
751082294	1208.207	31.175	4.2	0.311	0.551		
751082295	1208.543	31.171	4.7	0.261	0.568		
751082296	1208.875	31.183	2.2	0.633	0.666		
751082297	1209.21	31.161	4	0.417	0.668		
751082298	1133.15	37.275	5	0.075	0.81		
751082299	1133.484	37.273	5.3	0.058	0.972		
751082300	1133.819	37.267	4.6	0.158	0.756		
751082301	1134.155	37.252	4.4	0.122	0.748		
751082302	1134.489	37.221	4.2	0.105	0.818		
751082303	1134.823	37.095	3.7	0.183	0.849		
751082304	567.317	31.372	5.7	0.41	0.501		
751082305	567.818	31.353	4.7	0.743	0.745		
751082306	1068.478	22.399	-5.1	0.685	0.118		
751082307	1068.812	22.401	6.3	0.469	0.599		
751082308	1069.147	22.406	-3.9	0.666	0.14		
751082309	1069.492	22.501	-7.3	0.121	0.09		
751082310	1069.815	22.423	-25.8	0.193	0.663		
751082311	660.803	16.757	1.8	0.795	0.376	Tax_Id=9606 Gene_Symbol=GSN Isoform 1 of Gelsolin	SEDC[160.0302]FILDHGK
751082312	661.304	16.756	-2.6	0.876	0.935	Tax_Id=9606 Gene_Symbol=GSN Isoform 1 of Gelsolin	SEDC[160.0302]FILDHGK
751082313	661.806	16.764	19.7	0.577	0.842	Tax_Id=9606 Gene_Symbol=GSN Isoform 1 of Gelsolin	SEDC[160.0302]FILDHGK
751082314	441.308	16.182	-0.3	0.985	0.701		
751082315	442.312	16.179	-13.9	0.635	0.34		
751082316	453.232	33.153	11.1	0.029	0.909		

751082317	453.733	33.139	14.9	0.15	0.69		
751082318	810.341	17.531	1	0.888	0.187		
751082319	811.346	17.513	0	0.998	0.36		
751082320	542.945	28.819	2.4	0.854	0.907	Tax_Id=9606 Gene_Symbol=C4A;C4B complement component 4B preproprc	EELVYELNPLDHR
751082321	543.279	28.818	18.2	0.167	0.945	Tax_Id=9606 Gene_Symbol=C4A;C4B complement component 4B preproprc	EELVYELNPLDHR
751082322	543.613	28.821	5.8	0.816	0.883	Tax_Id=9606 Gene_Symbol=C4A;C4B complement component 4B preproprc	EELVYELNPLDHR
751082323	727.611	44.206	1.3	0.903	0.133		
751082324	727.861	44.202	2.8	0.896	0.308		
751082325	728.113	44.203	4.9	0.822	0.396		
751082326	728.374	44.252	0	0.997	0.171		
751082327	728.613	44.205	-2.3	0.816	0.066		
751082328	589.112	19.773	-32.5	0	0.001		
751082329	589.311	19.764	-15.6	0	0		
751082330	589.512	19.774	-7.3	0.419	0.334		
751082331	589.713	19.788	-38.3	0	0		
751082332	514.278	17.9	6.1	0.655	0.596		
751082333	514.777	17.926	4.2	0.809	0.393		
751082334	359.2	18.895	2.4	0.64	0.211	Tax_Id=9606 Gene_Symbol=CLU Isoform 2 of Clusterin	RPHFFFPK
751082335	359.535	18.891	3.6	0.56	0.222	Tax_Id=9606 Gene_Symbol=CLU Isoform 2 of Clusterin	RPHFFFPK
751082336	359.869	18.875	20.3	0.657	0.944	Tax_Id=9606 Gene_Symbol=CLU Isoform 2 of Clusterin	RPHFFFPK
751082337	1029.205	47.705	4.1	0.322	0.651		
751082338	1029.455	47.905	3.8	0.385	0.56		
751082339	1029.706	47.88	4.2	0.264	0.967		
751082340	1029.957	47.849	2.7	0.489	0.814		
751082341	1030.208	47.774	3.8	0.273	0.814		
751082342	1030.46	47.683	4.7	0.188	0.976		
751082343	924.435	29.424	2.7	0.578	0.946		
751082344	924.697	29.392	2	0.65	0.995		
751082345	924.947	29.393	0.4	0.923	0.781		
751082346	925.198	29.396	-2.6	0.68	0.211		
751082347	925.448	29.4	-7.7	0.255	0.421		
751082348	775.598	34.367	6.9	0.458	0.358		
751082349	775.798	34.375	9.7	0.234	0.854		
751082350	775.999	34.363	10.8	0.217	0.929		
751082351	776.2	34.352	12.9	0.377	0.49		
751082352	845.148	29.519	7.4	0.417	0.292		
751082353	845.397	29.554	9.6	0.5	0.586		
751082354	845.649	29.52	11	0.244	0.692		
751082355	845.899	29.53	11.3	0.361	0.459		
751082356	846.146	29.486	4.2	0.732	0.716		
751082357	846.403	29.53	1.2	0.952	0.852		
751082358	586.869	20.026	9.5	0.359	0.243		
751082359	587.07	20.027	8.7	0.392	0.242		
751082360	587.268	19.998	11.3	0.424	0.538		
751082361	587.47	20.031	9.7	0.441	0.157		
751082362	587.671	20.049	-18.2	0.674	0.49		
751082363	689.017	21.263	2.8	0.601	0.313		
751082364	689.351	21.423	4.2	0.496	0.807		
751082365	450.247	16.151	4	0.773	0.466	Tax_Id=9606 Gene_Symbol=CDH2 Cadherin-2	DVHEGQPLLNVK
751082366	450.581	16.166	-3	0.856	0.895	Tax_Id=9606 Gene_Symbol=CDH2 Cadherin-2	DVHEGQPLLNVK
751082367	450.915	16.172	-23.3	0.279	0.277	Tax_Id=9606 Gene_Symbol=CDH2 Cadherin-2	DVHEGQPLLNVK
751082368	775.892	38.298	-2.9	0.709	0.126		
751082369	776.059	38.297	-2	0.837	0.158		
751082370	776.227	38.306	-1.6	0.866	0.155		
751082371	776.394	38.313	-3.4	0.721	0.151		
751082372	776.561	38.326	-10.2	0.427	0.066		
751082373	907.647	18.039	-1	0.944	0.071		
751082374	907.9	18.043	7.9	0.431	0.695		
751082375	908.149	18.041	2.8	0.879	0.102		
751082376	908.401	18.043	3.2	0.888	0.32		
751082377	908.653	18.048	1	0.955	0.103		
751082378	621.956	20.699	10.3	0.457	0.381	Tax_Id=9606 Gene_Symbol=C1QB Complement C1q subcomponent subunit	FDHVITNMNNNYEPR
751082379	622.29	20.686	6.4	0.386	0.58	Tax_Id=9606 Gene_Symbol=C1QB Complement C1q subcomponent subunit	FDHVITNMNNNYEPR
751082380	622.624	20.706	4.4	0.763	0.172	Tax_Id=9606 Gene_Symbol=C1QB Complement C1q subcomponent subunit	FDHVITNMNNNYEPR
751082381	622.959	20.714	14.9	0.574	0.981	Tax_Id=9606 Gene_Symbol=C1QB Complement C1q subcomponent subunit	FDHVITNMNNNYEPR
751082382	501.744	22.265	10.4	0.083	0.288		
751082383	502.246	22.223	11.8	0.061	0.193		
751082384	571.318	21.204	3.6	0.439	0.861		
751082385	571.819	21.188	2.5	0.676	0.694		
751082386	597.869	31.715	3.4	0.754	0.339	Tax_Id=9606 Gene_Symbol=CNTN1 Isoform 1 of Contactin-1	FIPLIPIPER
751082387	598.371	31.708	2.2	0.902	0.48	Tax_Id=9606 Gene_Symbol=CNTN1 Isoform 1 of Contactin-1	FIPLIPIPER
751082388	735.865	31.799	0.4	0.942	0.732		
751082389	736.366	31.819	0.2	0.979	0.613		
751082390	736.868	31.827	-6.9	0.69	0.961		
751082391	452.215	16.172	0.3	0.977	0.205		
751082392	452.554	16.23	2.3	0.915	0.385		
751082393	784.394	34.514	-1.8	0.897	0.395		
751082394	784.731	34.542	-4.4	0.382	0.068		
751082395	785.066	34.551	-6.5	0.156	0.036		
751082396	785.4	34.547	-6.8	0.15	0.082		
751082397	670.335	27.416	11.1	0.204	0.898		
751082398	670.846	27.424	7.5	0.567	0.239		
751082399	671.352	27.469	4.7	0.584	0.218		
751082400	1192.631	22.557	13.7	0.355	0.824		
751082401	1193.634	22.564	8.3	0.189	0.658		
751082402	1194.636	22.594	25.2	0.354	0.612		
751082403	465.489	18.044	5.6	0.518	0.907		
751082404	465.74	18.045	8.8	0.095	0.86		
751082405	465.991	18.052	15.9	0.102	0.767		
751082406	723.079	25.453	16.7	0.103	0.703		
751082407	723.33	25.442	9.5	0.205	0.842		
751082408	723.58	25.45	5	0.592	0.245		
751082409	723.831	25.452	9	0.527	0.532		

751082410	724.082	25.451	-19.9	0.117	0.008		
751082411	592.277	28.393	14.4	0.079	0.724		
751082412	592.779	28.408	31.2	0.147	0.856		
751082413	467.247	16.41	4.6	0.615	0.356		
751082414	467.581	16.418	7.8	0.498	0.308		
751082415	467.915	16.423	24.5	0.352	0.304		
751082416	468.247	16.401	32.6	0.281	0.653		
751082417	959.427	29.502	3.4	0.794	0.138		
751082418	959.928	29.498	5.1	0.792	0.137		
751082419	960.426	29.578	3	0.68	0.134		
751082420	960.929	29.513	-16.2	0.354	0.108		
751082421	548.316	33.319	2.2	0.615	0.308		
751082422	548.651	33.323	3	0.615	0.526		
751082423	548.985	33.334	0.3	0.975	0.141		
751082424	525.013	20.911	1.7	0.506	0.154		
751082425	525.263	20.895	1.3	0.635	0.153		
751082426	525.514	20.884	2.2	0.404	0.132		
751082427	525.764	20.882	-36.1	0.098	0.473		
751082428	420.22	22.153	2.8	0.675	0.514	Tax_Id=9606 Gene_Symbol=CNDP1 Beta-Ala-His dipeptidase	AIHLDLEEYR
751082429	420.555	22.152	-32.2	0.165	0.218	Tax_Id=9606 Gene_Symbol=CNDP1 Beta-Ala-His dipeptidase	AIHLDLEEYR
751082430	420.889	22.155	5.1	0.845	0.7	Tax_Id=9606 Gene_Symbol=CNDP1 Beta-Ala-His dipeptidase	AIHLDLEEYR
751082431	867.075	28.072	6.8	0.224	0.676		
751082432	867.409	28.091	2.5	0.695	0.823		
751082433	867.743	28.004	4.6	0.384	0.718		
751082434	868.078	28.097	-2.6	0.809	0.934		
751082435	868.411	28.284	-9.5	0.557	0.682		
751082436	627.697	45.501	0.7	0.973	0.382		
751082437	628.032	45.532	1.1	0.869	0.494		
751082438	628.367	45.649	2.7	0.851	0.422		
751082439	628.701	45.544	-3.9	0.759	0.397		
751082440	1101.974	40.193	-2.3	0.722	0.121		
751082441	1102.119	40.188	-2.3	0.709	0.126		
751082442	1102.261	40.179	-2.7	0.686	0.15		
751082443	1102.403	40.189	0.6	0.965	0.296		
751082444	1102.546	40.196	-3.9	0.471	0.08		
751082445	1102.668	40.071	0.5	0.928	0.204		
751082446	1102.831	40.226	-3.5	0.449	0.133		
751082447	436.222	15.659	-0.1	0.99	0.313		
751082448	436.724	15.664	0	1	0.08		
751082449	471.661	34.523	9.8	0.65	0.543		
751082450	471.862	34.522	6.1	0.645	0.515		
751082451	472.062	34.527	-6.7	0.332	0.073		
751082452	472.263	34.534	3.8	0.722	0.237		
751082453	532.301	21.194	10	0.101	0.738		
751082454	532.801	21.103	8	0.175	0.613		
751082455	772.38	25.542	8.2	0.207	0.578		
751082456	772.883	25.544	7.9	0.22	0.526		
751082457	813.407	17.632	-1.4	0.832	0.327	Tax_Id=9606 Gene_Symbol=APOE Apolipoprotein E	VEQAVETEPEPELR
751082458	813.91	17.627	-3.6	0.651	0.053	Tax_Id=9606 Gene_Symbol=APOE Apolipoprotein E	VEQAVETEPEPELR
751082459	814.411	17.635	-21.9	0.241	0.174	Tax_Id=9606 Gene_Symbol=APOE Apolipoprotein E	VEQAVETEPEPELR
751082460	674.34	32.554	-0.8	0.873	0.382		
751082461	502.986	24.974	3.4	0.67	0.99		
751082462	503.32	24.971	2.3	0.797	0.99		
751082463	503.654	24.983	12.7	0.489	0.325		
751082464	1131.998	35.05	-2.2	0.714	0.379		
751082465	1132.499	35.046	-1	0.873	0.499		
751082466	1133.001	35.057	-2.6	0.674	0.298		
751082467	1133.5	35.095	-4.4	0.571	0.974		
751082468	544.94	21.496	3.7	0.747	0.237	Tax_Id=9606 Gene_Symbol=PEBP1 Phosphatidylethanolamine-binding prote	NRPTSISWDGLDSGK
751082469	545.275	21.511	3.5	0.797	0.38	Tax_Id=9606 Gene_Symbol=PEBP1 Phosphatidylethanolamine-binding prote	NRPTSISWDGLDSGK
751082470	545.609	21.507	3.1	0.837	0.1	Tax_Id=9606 Gene_Symbol=PEBP1 Phosphatidylethanolamine-binding prote	NRPTSISWDGLDSGK
751082471	677.344	29.855	3.5	0.517	0.129		
751082472	677.849	29.808	3.7	0.788	0.171		
751082473	678.353	29.807	4.1	0.731	0.23		
751082474	728.859	25.639	-2	0.454	0.196		
751082475	862.023	39.444	9.7	0.504	0.544	Tax_Id=9606 Gene_Symbol=AGT Angiotensinogen	VLSALQAVQGLLVAQGR
751082476	862.525	39.451	3.6	0.645	0.487	Tax_Id=9606 Gene_Symbol=AGT Angiotensinogen	VLSALQAVQGLLVAQGR
751082477	863.026	39.446	7.7	0.244	0.738	Tax_Id=9606 Gene_Symbol=AGT Angiotensinogen	VLSALQAVQGLLVAQGR
751082478	757.614	37.893	2	0.79	0.162		
751082479	757.863	37.895	1.7	0.882	0.114		
751082480	758.114	37.893	2.5	0.804	0.116		
751082481	758.363	37.907	2.8	0.743	0.224		
751082482	758.613	37.908	-5.8	0.728	0.072		
751082483	1079.976	47.58	4.6	0.292	0.927		
751082484	1080.227	47.89	4.4	0.26	0.808		
751082485	1080.477	47.862	2.6	0.528	0.741		
751082486	1080.729	47.803	3.6	0.34	0.728		
751082487	1080.98	47.726	2.8	0.469	0.577		
751082488	1081.23	47.364	3.5	0.428	0.744		
751082489	950.146	28.775	8.5	0.507	0.164		
751082490	950.483	28.744	7.9	0.595	0.329		
751082491	950.817	28.734	16.3	0.376	0.203		
751082492	951.155	28.737	8.8	0.581	0.141		
751082493	492.739	29.346	-3.3	0.527	0.084		
751082494	493.241	29.308	-0.6	0.924	0.207		
751082495	1037.987	31.601	-1.3	0.929	0.177		
751082496	1038.239	30.899	3.6	0.581	0.83		
751082497	725.885	26.232	2.2	0.76	0.978		
751082498	726.891	26.119	6.3	0.359	0.619		
751082499	809.599	37.116	2.6	0.473	0.264		
751082500	809.848	37.279	2.3	0.518	0.305		
751082501	810.099	37.277	2.9	0.43	0.428		
751082502	810.349	37.272	1.8	0.633	0.245		

751082503	810.602	37.25	1.8	0.802	0.22		
751082504	800.881	22.072	3.4	0.701	0.999		
751082505	801.382	22.085	-12	0.337	0.042		
751082506	801.884	21.994	-3.4	0.788	0.293		
751082507	687.337	38.657	5.6	0.532	0.98		
751082508	687.672	38.67	3.7	0.591	0.381		
751082509	688.005	38.687	4.7	0.483	0.425		
751082510	688.341	38.667	7	0.348	0.778		
751082511	688.674	38.69	23.9	0.316	0.373		
751082512	439.243	16.87	4.7	0.596	0.882		
751082513	439.745	16.931	2.8	0.844	0.217		
751082514	508.992	5.658	175.2	0.263	0.669		
751082515	635.642	22.272	-7.5	0.034	0.01	Tax_Id=9606 Gene_Symbol=- 52 kDa protein	VAELEHGSSAYSPPDAFK
751082516	635.978	22.267	-6.9	0.039	0.016	Tax_Id=9606 Gene_Symbol=- 52 kDa protein	VAELEHGSSAYSPPDAFK
751082517	636.31	22.327	-10.5	0.002	0.002	Tax_Id=9606 Gene_Symbol=- 52 kDa protein	VAELEHGSSAYSPPDAFK
751082518	636.645	22.309	-26.9	0.195	0.347	Tax_Id=9606 Gene_Symbol=- 52 kDa protein	VAELEHGSSAYSPPDAFK
751082519	768.676	66.71	2.6	0.767	0.615		
751082520	768.787	66.69	-1	0.91	0.874		
751082521	768.954	66.773	0.3	0.975	0.923		
751082522	731.836	28.744	8.1	0.331	0.33		
751082523	732.338	28.751	5.4	0.269	0.387		
751082524	732.839	28.775	16.8	0.349	0.523		
751082525	881.965	38.721	0.5	0.899	0.674		
751082526	882.469	38.693	0.5	0.898	0.644		
751082527	882.972	38.469	0.5	0.92	0.939		
751082528	1072.53	29.146	4.7	0.593	0.929		
751082529	1073.533	29.148	3.7	0.868	0.551		
751082530	750.651	20.907	-16.2	0.415	0.294		
751082531	750.985	20.905	4.8	0.65	0.47		
751082532	751.319	20.911	6.2	0.522	0.639		
751082533	751.653	20.921	2.7	0.879	0.956		
751082534	751.987	20.937	2.7	0.906	0.736		
751082535	740.892	23.477	-0.9	0.867	0.137		
751082536	741.894	23.485	-5.8	0.194	0.076		
751082537	514.792	15.338	11.8	0.417	0.701	Tax_Id=9606 Gene_Symbol=SERPIND1 Heparin cofactor 2	TLEAQLTPR
751082538	515.294	15.341	12.3	0.567	0.609	Tax_Id=9606 Gene_Symbol=SERPIND1 Heparin cofactor 2	TLEAQLTPR
751082539	623.339	43.567	14.4	0.02	0.657		
751082540	623.841	43.566	14.5	0.022	0.663		
751082541	624.342	43.573	55.4	0.064	0.198		
751082542	571.758	27.474	8.6	0.404	0.232		
751082543	572.26	27.483	9.9	0.562	0.355		
751082544	473.742	17.429	1.5	0.82	0.395		
751082545	474.243	17.414	2.9	0.696	0.398		
751082546	1202.912	38.795	0.8	0.872	0.26		
751082547	1203.246	38.798	1.2	0.839	0.289		
751082548	1203.581	38.799	5.8	0.404	0.43		
751082549	1203.914	38.799	1.8	0.782	0.408		
751082550	1204.249	38.831	-0.1	0.985	0.211		
751082551	467.563	27.413	24.7	0.028	0.3		
751082552	467.897	27.415	-2.1	0.804	0.334		
751082553	492.791	23.046	7.2	0.27	0.655		
751082554	493.293	23.126	9.7	0.381	0.617		
751082555	1034.531	37.59	8.3	0.572	0.624		
751082556	1035.53	37.653	6.1	0.318	0.834		
751082557	1036.54	37.667	11.5	0.333	0.5		
751082558	640.315	21.718	1.5	0.794	0.736		
751082559	640.817	21.735	1.7	0.773	0.773		
751082560	641.318	21.759	6.2	0.712	0.797		
751082561	470.262	21.279	4.4	0.622	0.357		
751082562	686.391	27.861	5.4	0.224	0.774	Tax_Id=9606 Gene_Symbol=CP Ceruloplasmin	GAYPLSIEPIGVR
751082563	686.892	27.875	3.5	0.452	0.803	Tax_Id=9606 Gene_Symbol=CP Ceruloplasmin	GAYPLSIEPIGVR
751082564	649.624	23.995	-0.7	0.914	0.652		
751082565	442.718	19.232	29.6	0.225	0.305		
751082566	443.22	19.235	19.2	0.548	0.376		
751082567	729.696	26.378	-1.8	0.758	0.229		
751082568	730.033	26.438	-2.3	0.737	0.238		
751082569	730.366	26.385	3.2	0.73	0.656		
751082570	526.271	15.169	4.4	0.376	0.759	Tax_Id=9606 Gene_Symbol=CFB Isoform 1 of Complement factor B (Fragme	ALFVSEEEK
751082571	526.772	15.162	5.5	0.56	0.835	Tax_Id=9606 Gene_Symbol=CFB Isoform 1 of Complement factor B (Fragme	ALFVSEEEK
751082572	944.482	39.411	6	0.244	0.756	Tax_Id=9606 Gene_Symbol=AFM Afamin	SDVGFLPPFPTLDPEEK
751082573	944.985	39.474	7.1	0.183	0.98	Tax_Id=9606 Gene_Symbol=AFM Afamin	SDVGFLPPFPTLDPEEK
751082574	945.477	39.412	6.2	0.116	0.957	Tax_Id=9606 Gene_Symbol=AFM Afamin	SDVGFLPPFPTLDPEEK
751082575	436.519	14.403	-45.3	0.057	0.106		
751082576	436.853	14.405	-53.4	0.099	0.083		
751082577	437.187	14.404	-1.4	0.901	0.433		
751082578	730.036	34.371	8.2	0.37	0.726		
751082579	731.037	34.445	8.3	0.628	0.922		
751082580	821.974	33.329	0.6	0.902	0.349	Tax_Id=9606 Gene_Symbol=DAG1 Dystroglycan	VTIPTDLIASSGDIIK
751082581	822.476	33.33	2.7	0.639	0.571	Tax_Id=9606 Gene_Symbol=DAG1 Dystroglycan	VTIPTDLIASSGDIIK
751082582	822.979	33.341	-2.8	0.888	0.339	Tax_Id=9606 Gene_Symbol=DAG1 Dystroglycan	VTIPTDLIASSGDIIK
751082583	419.317	64.778	6	0.468	0.284		
751082584	420.321	64.881	5.4	0.483	0.257		
751082585	585.316	25.013	6	0.612	0.496		
751082586	585.818	24.978	15.6	0.12	0.883		
751082587	806.899	21.429	-4	0.73	0.074		
751082588	807.4	21.437	-3.1	0.645	0.046		
751082589	807.901	21.455	-7.6	0.613	0.069		
751082590	808.403	21.444	-64.9	0.001	0.006		
751082591	474.739	20.058	0.8	0.87	0.216		
751082592	475.241	20.075	2.1	0.746	0.296		
751082593	475.75	20.125	0.8	0.951	0.141		
751082594	659.393	29.953	3.1	0.733	0.332		
751082595	659.897	29.969	3.4	0.807	0.3		

751082596	1190.615	18.367	17.3	0.267	0.86		
751082597	911.131	46.005	6.7	0.326	0.374		
751082598	911.466	45.999	15.6	0.165	0.845		
751082599	911.801	45.999	7	0.37	0.317		
751082600	912.135	45.994	4.3	0.634	0.239		
751082601	912.47	46.005	-3.7	0.744	0.061		
751082602	626.819	25.443	-0.2	0.962	0.98		
751082603	627.321	25.446	-1.8	0.651	0.688		
751082604	627.823	25.467	3.8	0.885	0.412		
751082605	810.392	33.069	5.8	0.449	0.518		
751082606	810.892	33.081	4.7	0.657	0.209		
751082607	811.391	33.02	-0.9	0.954	0.218		
751082608	616.323	16.815	8.6	0.206	0.909		
751082609	616.826	16.825	3.1	0.66	0.7		
751082610	1186.093	45.594	-0.5	0.935	0.593		
751082611	409.541	22.555	1.6	0.81	0.464		
751082612	409.875	22.537	0.5	0.924	0.39		
751082613	410.211	22.542	-22.3	0.219	0.103		
751082614	761.181	33.355	3.6	0.803	0.15		
751082615	761.381	33.358	4.1	0.405	0.339		
751082616	761.582	33.363	11.1	0.323	0.987		
751082617	761.783	33.358	10.9	0.281	0.991		
751082618	761.983	33.364	7.6	0.349	0.64		
751082619	985.52	26.994	7.5	0.123	0.955		
751082620	986.02	26.995	7.1	0.147	0.877		
751082621	986.521	26.992	1.1	0.905	0.155		
751082622	1039.514	35.727	-3.6	0.756	0.177		
751082623	1039.855	35.802	8.2	0.583	0.573		
751082624	1040.192	35.772	11.9	0.157	0.9		
751082625	1040.518	35.752	9.5	0.213	0.698		
751082626	1040.858	35.816	-1.3	0.911	0.251		
751082627	695.045	23.376	7.2	0.257	0.595		
751082628	695.296	23.526	4.9	0.356	0.866		
751082629	695.546	23.521	7.4	0.377	0.621		
751082630	695.797	23.518	1.7	0.757	0.513		
751082631	696.045	23.492	18.6	0.123	0.868		
751082632	496.757	18.246	15.5	0.295	0.927		
751082633	497.259	18.253	16	0.469	0.919		
751082634	1017.162	36.344	-12	0.338	0.096		
751082635	1017.492	36.337	7.9	0.434	0.974		
751082636	1017.83	36.36	-0.8	0.924	0.212		
751082637	1018.166	36.369	-2.4	0.826	0.151		
751082638	1018.497	36.383	-18.1	0.294	0.18		
751082639	575.321	15.407	11	0.195	0.671		
751082640	575.656	15.421	91.8	0.02	0.186		
751082641	575.99	15.424	208.3	0.032	0.15		
751082642	987.935	14.916	2.3	0.888	0.11		
751082643	988.437	14.916	2.3	0.884	0.084		
751082644	988.938	14.917	5.7	0.775	0.179		
751082645	989.44	14.891	-37.8	0.118	0.257		
751082646	933.096	33.999	7.2	0.457	0.197		
751082647	933.43	33.994	6.9	0.372	0.289		
751082648	933.765	33.998	4.8	0.418	0.206		
751082649	934.098	34.001	6.1	0.579	0.184		
751082650	934.432	33.999	2.4	0.929	0.514		
751082651	431.233	21.058	-7.8	0.218	0.163		
751082652	431.567	21.061	7.7	0.632	0.895		
751082653	431.901	21.062	-45.3	0.044	0.02		
751082654	465.262	23.928	6.8	0.679	0.191		
751082655	465.763	23.938	8.2	0.663	0.222		
751082656	1167.315	35.135	5.5	0.644	0.296		
751082657	1167.566	35.141	4.7	0.679	0.342		
751082658	1167.817	35.146	3.4	0.727	0.321		
751082659	1168.068	35.148	2.5	0.807	0.183		
751082660	1168.318	35.151	4.3	0.674	0.764		
751082661	871.428	44.538	2.3	0.536	0.535		
751082662	871.76	44.666	2.2	0.584	0.553		
751082663	872.093	44.757	2.2	0.568	0.375		
751082664	872.427	44.758	1.7	0.632	0.387		
751082665	458.889	15.393	-2.1	0.926	0.233	Tax_Id=9606 Gene_Symbol=APP Isoform L-APP733 of Amyloid beta A4 prot	VESLEQEAANER
751082666	459.223	15.408	2.2	0.872	0.604	Tax_Id=9606 Gene_Symbol=APP Isoform L-APP733 of Amyloid beta A4 prot	VESLEQEAANER
751082667	1083.195	38.417	11	0.416	0.615		
751082668	1083.53	38.408	4.4	0.584	0.678		
751082669	1083.864	38.427	7.3	0.474	0.939		
751082670	1084.197	38.415	5.3	0.604	0.682		
751082671	503.783	18.119	17.4	0.163	0.783		
751082672	504.282	18.066	0.5	0.94	0.638		
751082673	713.357	24.122	3.2	0.507	0.62		
751082674	713.69	24.098	2.2	0.666	0.517		
751082675	714.025	24.086	2.7	0.811	0.721		
751082676	714.361	24.059	20.5	0.353	0.978		
751082677	520.249	18.086	5	0.903	0.565		
751082678	520.751	18.081	-9.5	0.51	0.061		
751082679	693.646	31.065	2.4	0.727	0.21		
751082680	693.982	31.061	3	0.667	0.272		
751082681	694.314	31.05	0	0.994	0.069		
751082682	694.649	31.051	-3.9	0.717	0.046		
751082683	592.831	17.714	6.5	0.216	0.859		
751082684	1001.475	29.215	-0.7	0.948	0.894		
751082685	1001.727	29.214	1.8	0.694	0.991		
751082686	1001.977	29.217	0.8	0.852	0.999		
751082687	1002.228	29.216	-6.2	0.449	0.462		
751082688	1002.479	29.216	-5.7	0.48	0.38		

751082689	1177.875	36.11	-1	0.874	0.995		
751082690	1178.212	36.099	-6.1	0.35	0.621		
751082691	1178.546	36.112	-5.1	0.442	0.611		
751082692	1178.88	36.116	-5	0.434	0.663		
751082693	1179.23	36.088	3.6	0.568	0.574		
751082694	389.562	18.223	1.1	0.923	0.114		
751082695	389.896	18.225	-1.9	0.92	0.244		
751082696	390.231	18.224	-3.9	0.918	0.545		
751082697	800.044	35.548	4.7	0.197	0.939	Tax_Id=9606 Gene_Symbol=CP Ceruloplasmin	HYYIGIIETTWDYASDHGEK
751082698	800.379	35.581	4.7	0.205	0.859	Tax_Id=9606 Gene_Symbol=CP Ceruloplasmin	HYYIGIIETTWDYASDHGEK
751082699	800.713	35.593	4.7	0.184	0.93	Tax_Id=9606 Gene_Symbol=CP Ceruloplasmin	HYYIGIIETTWDYASDHGEK
751082700	801.047	35.572	7.2	0.439	0.327	Tax_Id=9606 Gene_Symbol=CP Ceruloplasmin	HYYIGIIETTWDYASDHGEK
751082701	908.436	25.832	4.3	0.336	0.639	Tax_Id=9606 Gene_Symbol=APOA1 Apolipoprotein A-I	DSGRDYVSQFEGSALGK
751082702	908.938	25.832	4	0.419	0.506	Tax_Id=9606 Gene_Symbol=APOA1 Apolipoprotein A-I	DSGRDYVSQFEGSALGK
751082703	909.437	25.81	5.3	0.519	0.945	Tax_Id=9606 Gene_Symbol=APOA1 Apolipoprotein A-I	DSGRDYVSQFEGSALGK
751082704	619.975	39.506	0	0.999	0.635	Tax_Id=9606 Gene_Symbol=HRG Histidine-rich glycoprotein	SGFPQVSMFFTHTFPK
751082705	620.31	39.521	3.1	0.883	0.303	Tax_Id=9606 Gene_Symbol=HRG Histidine-rich glycoprotein	SGFPQVSMFFTHTFPK
751082706	620.643	39.506	-2.5	0.926	0.916	Tax_Id=9606 Gene_Symbol=HRG Histidine-rich glycoprotein	SGFPQVSMFFTHTFPK
751082707	620.977	39.507	-25.3	0.224	0.026	Tax_Id=9606 Gene_Symbol=HRG Histidine-rich glycoprotein	SGFPQVSMFFTHTFPK
751082708	1153.756	29.556	3.4	0.794	0.538		
751082709	1154.008	29.554	10.1	0.505	0.598		
751082710	1154.26	29.55	7.6	0.605	0.541		
751082711	1154.51	29.546	8.2	0.367	0.831		
751082712	1154.761	29.556	6.4	0.659	0.507		
751082713	1155.011	29.566	12.1	0.474	0.668		
751082714	682.545	45.349	5.2	0.504	0.681		
751082715	682.713	45.316	2.7	0.733	0.639		
751082716	682.88	45.286	3.5	0.6	0.879		
751082717	683.047	45.287	1.4	0.82	0.694		
751082718	717.872	38.142	8.4	0.112	0.848		
751082719	718.372	38.109	6.5	0.066	0.355		
751082720	718.873	38.271	4.8	0.215	0.585		
751082721	682.462	45.292	4	0.535	0.885		
751082722	682.629	45.32	3.4	0.637	0.763		
751082723	682.796	45.353	2.9	0.674	0.725		
751082724	682.963	45.306	0.8	0.889	0.477		
751082725	683.131	45.303	-1.7	0.761	0.079		
751082726	402.566	18.22	0.9	0.961	0.079		
751082727	402.901	18.22	-3.2	0.864	0.034		
751082728	604.814	22.528	6.8	0.293	0.796		
751082729	970.981	32.087	4.5	0.438	0.841		
751082730	666.556	40.023	10.9	0.632	0.497		
751082731	666.756	40.02	8.9	0.478	0.325		
751082732	666.957	40.022	7.6	0.437	0.251		
751082733	667.157	40.024	8.2	0.402	0.301		
751082734	667.357	40.029	7.2	0.665	0.262		
751082735	667.559	40.023	-1.2	0.957	0.621		
751082736	768.454	66.72	0.7	0.929	0.673		
751082737	768.621	66.675	-0.1	0.988	0.953		
751082738	768.731	66.735	2	0.817	0.597		
751082739	768.843	66.699	-0.4	0.96	0.82		
751082740	542.787	18.657	6.4	0.328	0.97		
751082741	543.29	18.664	3.6	0.849	0.682		
751082742	708.609	42.206	14.3	0.361	0.784		
751082743	708.86	42.208	7.4	0.498	0.393		
751082744	709.111	42.205	17	0.221	0.753		
751082745	709.36	42.245	6.1	0.585	0.369		
751082746	709.612	42.173	-18.1	0.351	0.218		
751082747	563.259	28.493	5.3	0.127	0.923		
751082748	563.594	28.5	3.7	0.295	0.655		
751082749	563.929	28.492	2.8	0.529	0.578		
751082750	732.106	36.985	-0.5	0.974	0.926		
751082751	732.357	37.036	0.6	0.938	0.378		
751082752	732.607	36.985	4.6	0.669	0.332		
751082753	732.854	37.002	3.9	0.742	0.799		
751082754	679.327	25.588	5.7	0.4	0.434		
751082755	679.661	25.579	6.4	0.344	0.418		
751082756	679.995	25.584	4.9	0.482	0.375		
751082757	483.973	23.987	0.7	0.935	0.516		
751082758	484.224	23.998	0.9	0.871	0.445		
751082759	484.475	23.976	-9	0.407	0.301		
751082760	455.767	17.267	-7.7	0.03	0.002		
751082761	456.269	17.278	-9.4	0.581	0.226		
751082762	627.048	26.571	3	0.634	0.31		
751082763	627.299	26.578	0.6	0.907	0.19		
751082764	627.549	26.572	3.3	0.748	0.392		
751082765	627.801	26.573	1.5	0.869	0.27		
751082766	1165.566	40.815	0.8	0.911	0.288		
751082767	1165.817	40.835	6	0.462	0.634		
751082768	1166.068	40.847	3.9	0.609	0.453		
751082769	1166.318	40.885	3.2	0.641	0.46		
751082770	1166.568	41.005	0	0.996	0.209		
751082771	1166.816	41.022	-0.6	0.919	0.193		
751082772	1167.073	40.997	-0.7	0.913	0.323		
751082773	398.7	15.159	17.3	0.237	0.65		
751082774	399.202	15.159	24.6	0.205	0.752		
751082775	724.89	21.048	-1	0.866	0.258	Tax_Id=9606 Gene_Symbol=LSAMP Limbic system-associated membrane p	VTVNYPPTITESK
751082776	725.391	21.044	-1.2	0.783	0.142	Tax_Id=9606 Gene_Symbol=LSAMP Limbic system-associated membrane p	VTVNYPPTITESK
751082777	485.598	21.117	14.8	0.351	0.957		
751082778	485.932	21.101	14.2	0.39	0.953		
751082779	486.267	21.082	16.5	0.318	0.818		
751082780	606.329	17.163	15.7	0.166	0.593		
751082781	607.333	17.226	6	0.68	0.64		

751082782	412.904	21.347	-32.1	0.142	0.238	Tax_Id=9606 Gene_Symbol=ENPP2 Isoform 1 of Ectonucleotide pyrophosph	IEDIHLLVER
751082783	413.239	21.346	-6.1	0.76	0.833	Tax_Id=9606 Gene_Symbol=ENPP2 Isoform 1 of Ectonucleotide pyrophosph	IEDIHLLVER
751082784	413.573	21.339	14.2	0.67	0.848	Tax_Id=9606 Gene_Symbol=ENPP2 Isoform 1 of Ectonucleotide pyrophosph	IEDIHLLVER
751082785	748.384	36.209	-10.1	0.021	0.036	Tax_Id=9606 Gene_Symbol=CHGB Secretogranin-1	ADQTVLTEDEKKELENLAAM[147.0355]DLELQK
751082786	748.634	36.259	-10.2	0.123	0.073	Tax_Id=9606 Gene_Symbol=CHGB Secretogranin-1	ADQTVLTEDEKKELENLAAM[147.0355]DLELQK
751082787	748.886	36.235	-7.1	0.094	0.088	Tax_Id=9606 Gene_Symbol=CHGB Secretogranin-1	ADQTVLTEDEKKELENLAAM[147.0355]DLELQK
751082788	749.137	36.233	-6.2	0.623	0.274	Tax_Id=9606 Gene_Symbol=CHGB Secretogranin-1	ADQTVLTEDEKKELENLAAM[147.0355]DLELQK
751082789	585.296	19.683	1	0.846	0.629		
751082790	585.797	19.699	2.2	0.694	0.694		
751082791	1145.992	40.06	-0.2	0.978	0.102		
751082792	1146.136	40.057	-4.4	0.552	0.137		
751082793	1146.28	40.052	-3.9	0.551	0.118		
751082794	1146.421	40.061	-2.3	0.858	0.29		
751082795	1146.564	40.073	-0.4	0.978	0.352		
751082796	1146.706	40.062	-1.6	0.924	0.281		
751082797	899.601	29.509	-33.6	0.018	0.008		
751082798	899.799	29.556	-5.1	0.806	0.293		
751082799	900.001	29.562	-5.3	0.8	0.303		
751082800	900.2	29.583	-6.7	0.304	0.046		
751082801	900.404	29.629	-24.3	0.126	0.102		
751082802	900.604	29.561	-39.6	0.007	0.007		
751082803	558.099	20.642	-2.1	0.667	0.231		
751082804	558.265	20.62	0.4	0.943	0.453		
751082805	558.433	20.641	-1.9	0.66	0.115		
751082806	558.599	20.631	-0.4	0.944	0.358		
751082807	558.767	20.655	-4.2	0.602	0.247		
751082808	558.932	20.643	-0.6	0.94	0.205		
751082809	505.637	16.806	8.4	0.679	0.521	Tax_Id=9606 Gene_Symbol=CP Ceruloplasmin	TYSDHPEKVNKDDEEFIESNK
751082810	505.837	16.8	10.5	0.474	0.707	Tax_Id=9606 Gene_Symbol=CP Ceruloplasmin	TYSDHPEKVNKDDEEFIESNK
751082811	506.038	16.803	6.1	0.428	0.677	Tax_Id=9606 Gene_Symbol=CP Ceruloplasmin	TYSDHPEKVNKDDEEFIESNK
751082812	695.734	37.407	5.7	0.075	0.69	Tax_Id=9606 Gene_Symbol=C4A;C4B complement component 4B preproprc	ASAGLLGAHAAAITAYALTLTK
751082813	696.068	37.41	5.7	0.087	0.819	Tax_Id=9606 Gene_Symbol=C4A;C4B complement component 4B preproprc	ASAGLLGAHAAAITAYALTLTK
751082814	696.402	37.406	4.4	0.2	0.913	Tax_Id=9606 Gene_Symbol=C4A;C4B complement component 4B preproprc	ASAGLLGAHAAAITAYALTLTK
751082815	1117.02	27.061	-7.3	0.378	0.113		
751082816	1117.509	27.1	-11.3	0.062	0.045		
751082817	1118.003	27.123	-20	0.005	0.013		
751082818	724.67	31.153	15.6	0.345	0.846		
751082819	724.836	31.158	14.5	0.335	0.485		
751082820	725.004	31.15	7.9	0.118	0.614		
751082821	725.17	31.144	13	0.273	0.552		
751082822	725.336	31.143	9.5	0.262	0.495		
751082823	725.503	31.151	-1.4	0.914	0.106		
751082824	725.671	31.149	-29.2	0.135	0.033		
751082825	720.396	20.676	-1.4	0.938	0.56		
751082826	721.401	20.675	-3	0.919	0.443		
751082827	512.232	23.639	5.6	0.419	0.181		
751082828	512.734	23.648	12.3	0.478	0.199		
751082829	480.768	20.195	8.1	0.465	0.323		
751082830	481.269	20.205	8.9	0.546	0.281		
751082831	492.248	18.397	5.4	0.746	0.556	Tax_Id=9606 Gene_Symbol=C4A;C4B complement component 4B preproprc	TYNVLDMK
751082832	492.749	18.499	5.2	0.834	0.423	Tax_Id=9606 Gene_Symbol=C4A;C4B complement component 4B preproprc	TYNVLDMK
751082833	672.377	15.834	5	0.798	0.144		
751082834	673.381	15.843	-34.9	0.093	0.004		
751082835	429.558	24.903	11.6	0.486	0.23		
751082836	429.893	24.903	12.1	0.486	0.176		
751082837	430.227	24.823	9	0.504	0.616		
751082838	505.268	22.186	-2.7	0.687	0.247		
751082839	505.77	22.143	-14.9	0.21	0.024		
751082840	731.934	27.397	3.2	0.355	0.348	Tax_Id=9606 Gene_Symbol=APOA1 Apolipoprotein A-I	VKDLATVYVDVLK
751082841	732.436	27.397	3	0.396	0.355	Tax_Id=9606 Gene_Symbol=APOA1 Apolipoprotein A-I	VKDLATVYVDVLK
751082842	732.936	27.383	-0.8	0.952	0.746	Tax_Id=9606 Gene_Symbol=APOA1 Apolipoprotein A-I	VKDLATVYVDVLK
751082843	535.268	17.831	9.2	0.019	0.213		
751082844	535.519	17.829	10.1	0.015	0.171		
751082845	535.769	17.82	9.9	0.022	0.19		
751082846	536.02	17.831	25.9	0.081	0.291		
751082847	562.769	24.837	5.2	0.321	0.516		
751082848	710.361	24.624	-8.3	0.06	0.043		
751082849	710.694	24.635	-12.7	0.037	0.024		
751082850	711.028	24.62	-25.1	0.001	0.003		
751082851	478.268	26.964	4.2	0.439	0.338		
751082852	478.768	27.062	0.5	0.958	0.334		
751082853	607.803	33.619	9.5	0.053	0.623		
751082854	608.302	33.611	4.6	0.358	0.081		
751082855	987.017	35.32	4.5	0.716	0.414	Tax_Id=9606 Gene_Symbol=CD14 Putative uncharacterized protein CD14	AFPALTSLDLSDNPGLGER
751082856	987.518	35.316	3.2	0.513	0.369	Tax_Id=9606 Gene_Symbol=CD14 Putative uncharacterized protein CD14	AFPALTSLDLSDNPGLGER
751082857	988.016	35.318	2	0.694	0.285	Tax_Id=9606 Gene_Symbol=CD14 Putative uncharacterized protein CD14	AFPALTSLDLSDNPGLGER
751082858	663.458	65.066	5.1	0.515	0.274		
751082859	664.461	65.175	4.5	0.545	0.305		
751082860	665.465	65.361	8	0.62	0.207		
751082861	432.215	22.743	6.5	0.645	0.775		
751082862	432.55	22.748	6.8	0.746	0.611		
751082863	432.884	22.764	40.8	0.249	0.49		
751082864	465.596	26.014	5.6	0.578	0.383		
751082865	465.93	26.018	6.5	0.764	0.492		
751082866	466.264	26.035	7.2	0.606	0.411		
751082867	908.463	40.858	2.7	0.7	0.529		
751082868	908.97	40.853	4.3	0.581	0.423		
751082869	909.467	40.884	-1.7	0.794	0.211		
751082870	589.566	24.5	2.4	0.762	0.852		
751082871	589.817	24.515	4	0.621	0.974		
751082872	590.067	24.513	3.6	0.61	0.592		
751082873	479.73	15.094	2.8	0.899	0.497		
751082874	480.232	15.122	-3.9	0.797	0.162		

751082875	772.389	22.827	-0.1	0.979	0.073	Tax_Id=9606 Gene_Symbol=FN1 263 kDa protein	SYTITGLQPGTDYK
751082876	772.892	22.779	1.7	0.694	0.224	Tax_Id=9606 Gene_Symbol=FN1 263 kDa protein	SYTITGLQPGTDYK
751082877	773.395	22.819	0.1	0.988	0.141	Tax_Id=9606 Gene_Symbol=FN1 263 kDa protein	SYTITGLQPGTDYK
751082878	816.756	35.327	4.6	0.24	0.904		
751082879	817.09	35.28	3.8	0.308	0.998		
751082880	817.424	35.261	5.6	0.138	0.645		
751082881	817.76	35.295	17	0.09	0.296		
751082882	399.741	17.996	12.5	0.568	0.791		
751082883	400.242	18.01	5.6	0.865	0.661		
751082884	833.389	30.968	-3.4	0.392	0.075		
751082885	833.586	30.972	-4.1	0.402	0.122		
751082886	833.786	30.97	-2	0.725	0.414		
751082887	833.986	30.972	-0.6	0.892	0.311		
751082888	834.187	30.981	13.1	0.42	0.34		
751082889	834.389	31.001	-11.9	0.513	0.364		
751082890	930.434	37.865	4.8	0.464	0.906		
751082891	930.769	37.863	18.1	0.053	0.338		
751082892	931.094	37.942	7.7	0.259	0.987		
751082893	931.433	38.146	13.1	0.131	0.562		
751082894	931.751	37.862	-6.9	0.66	0.534		
751082895	711.87	48.28	4.4	0.519	0.816		
751082896	712.015	48.263	1.7	0.776	0.644		
751082897	712.157	48.299	3.1	0.604	0.887		
751082898	712.3	48.3	1.6	0.779	0.225		
751082899	490.775	16.68	3.6	0.868	0.431		
751082900	491.278	16.745	0.7	0.874	0.206		
751082901	922.426	33.495	-6.9	0.375	0.18		
751082902	922.678	33.447	-0.1	0.989	0.104		
751082903	922.929	33.473	-0.6	0.931	0.106		
751082904	923.176	33.421	-4.8	0.694	0.235		
751082905	923.43	33.522	-20.7	0.034	0.017		
751082906	414.746	22.013	3.9	0.709	0.436		
751082907	415.248	22.02	-18.6	0.546	0.288		
751082908	799.514	42.176	8.4	0.258	0.497		
751082909	799.656	42.184	7	0.355	0.316		
751082910	799.799	42.174	7.1	0.389	0.783		
751082911	799.942	42.179	8.2	0.586	0.582		
751082912	800.086	42.185	6.1	0.459	0.604		
751082913	800.229	42.176	5.9	0.553	0.18		
751082914	800.373	42.157	4.3	0.67	0.523		
751082915	1304.906	35.394	7.5	0.033	0.543		
751082916	1305.241	35.442	6.6	0.033	0.829		
751082917	1305.574	35.427	6.5	0.038	0.696		
751082918	1305.909	35.405	7.2	0.024	0.807		
751082919	1306.245	35.361	7.3	0.051	0.682		
751082920	1306.582	35.169	5.8	0.142	0.591		
751082921	530.289	14.496	5.5	0.785	0.373		
751082922	530.791	14.513	6.9	0.494	0.364		
751082923	531.292	14.511	-13.4	0.715	0.858		
751082924	615.841	18.021	6.2	0.377	0.387	Tax_Id=9606 Gene_Symbol=APOD Putative uncharacterized protein APOD	NILTSNNIDVK
751082925	616.343	18.06	8.9	0.211	0.637	Tax_Id=9606 Gene_Symbol=APOD Putative uncharacterized protein APOD	NILTSNNIDVK
751082926	708.335	39.372	1.2	0.79	0.777		
751082927	708.669	39.381	-0.2	0.951	0.327		
751082928	708.996	39.495	0.5	0.891	0.37		
751082929	709.337	39.379	0.6	0.92	0.51		
751082930	1070.301	45.856	3.6	0.81	0.813		
751082931	1070.549	45.961	5.4	0.326	0.635		
751082932	1070.799	46.053	4.6	0.225	0.348		
751082933	1071.049	46.118	5.9	0.136	0.426		
751082934	462.924	25.211	7.7	0.03	0.672	Tax_Id=9606 Gene_Symbol=CD44 Isoform 12 of CD44 antigen	YGFIEGHVVIPR
751082935	463.259	25.208	8.4	0.038	0.806	Tax_Id=9606 Gene_Symbol=CD44 Isoform 12 of CD44 antigen	YGFIEGHVVIPR
751082936	463.593	25.212	21.8	0.173	0.924	Tax_Id=9606 Gene_Symbol=CD44 Isoform 12 of CD44 antigen	YGFIEGHVVIPR
751082937	1055.653	17.424	3.5	0.741	0.366		
751082938	1056.656	17.426	-5.2	0.79	0.296		
751082939	427.888	16.372	69.4	0.061	0.351		
751082940	428.223	16.345	4.8	0.554	0.445		
751082941	428.557	16.331	8.3	0.655	0.183		
751082942	817.582	23.946	1.6	0.837	0.321		
751082943	817.833	23.944	15.7	0.115	0.563		
751082944	818.083	23.949	13.5	0.172	0.636		
751082945	818.334	23.951	-5.4	0.458	0.151		
751082946	818.584	23.968	23.7	0.387	0.788		
751082947	618.921	39.986	5.4	0.625	0.165		
751082948	619.046	40.047	3.7	0.647	0.137		
751082949	619.171	40.064	4.8	0.469	0.252		
751082950	619.296	39.975	3.9	0.537	0.21		
751082951	619.421	40.042	4.7	0.678	0.185		
751082952	619.547	39.931	25.5	0.122	0.931		
751082953	680.293	17.238	6.8	0.783	0.333		
751082954	680.795	17.257	1.5	0.934	0.142		
751082955	605.626	18.931	-4.1	0.447	0.241		
751082956	605.959	18.83	-1.9	0.736	0.445		
751082957	772.423	15.041	7.9	0.663	0.807		
751082958	606.315	20.195	-12.5	0.006	0.014		
751082959	606.517	20.148	-14	0.001	0.003		
751082960	606.72	20.151	-5.7	0.318	0.342		
751082961	606.919	20.182	-18.1	0	0.001		
751082962	607.12	20.136	-13.6	0.002	0.003		
751082963	661.341	23.337	-6.5	0.414	0.36		
751082964	661.538	23.263	8.7	0.27	0.407		
751082965	661.738	23.261	0	0.995	0.713		
751082966	661.94	23.261	-10	0.282	0.207		
751082967	662.139	23.284	7.5	0.349	0.878		

751082968	492.282	16.479	3.2	0.882	0.41		
751082969	839.459	36.716	5.7	0.706	0.457	Tax_Id=9606 Gene_Symbol=TTR Transthyretin	RYTIAALLSPYSYSTTAVVTNPK
751082970	839.793	36.7	2.1	0.847	0.385	Tax_Id=9606 Gene_Symbol=TTR Transthyretin	RYTIAALLSPYSYSTTAVVTNPK
751082971	840.128	36.711	7.2	0.693	0.87	Tax_Id=9606 Gene_Symbol=TTR Transthyretin	RYTIAALLSPYSYSTTAVVTNPK
751082972	840.461	36.689	9.7	0.578	0.87	Tax_Id=9606 Gene_Symbol=TTR Transthyretin	RYTIAALLSPYSYSTTAVVTNPK
751082973	561.919	17.997	13.8	0.045	0.859		
751082974	562.252	18.003	10.9	0.128	0.662		
751082975	562.587	18.019	16.7	0.539	0.547		
751082976	372.954	14.15	5.5	0.664	0.463		
751082977	373.205	14.148	4.1	0.837	0.375		
751082978	373.455	14.145	1.7	0.955	0.673		
751082979	643.063	32.023	1.2	0.793	0.505	Tax_Id=9606 Gene_Symbol=CADM3 Isoform 2 of Cell adhesion molecule 3	SLVTVLGIPQKPIITGYK
751082980	643.398	32.019	-0.3	0.951	0.351	Tax_Id=9606 Gene_Symbol=CADM3 Isoform 2 of Cell adhesion molecule 3	SLVTVLGIPQKPIITGYK
751082981	643.732	31.999	-3.5	0.437	0.16	Tax_Id=9606 Gene_Symbol=CADM3 Isoform 2 of Cell adhesion molecule 3	SLVTVLGIPQKPIITGYK
751082982	539.313	27.523	1.9	0.736	0.453		
751082983	539.647	27.523	2.9	0.66	0.695		
751082984	539.981	27.528	-4.3	0.689	0.117		
751082985	1059.806	35.668	5.8	0.65	0.26		
751082986	1059.973	35.668	4.5	0.657	0.262		
751082987	1060.139	35.664	20.5	0.158	0.352		
751082988	1060.306	35.671	3.7	0.703	0.36		
751082989	1060.474	35.666	15.1	0.279	0.427		
751082990	1060.642	35.684	-6.1	0.747	0.15		
751082991	598.946	27.781	-2.6	0.798	0.17		
751082992	599.282	27.803	7.2	0.312	0.951		
751082993	599.615	27.795	0	0.998	0.176		
751082994	552.585	17.142	16.4	0.096	0.643		
751082995	552.919	17.155	11	0.447	0.662		
751082996	553.254	17.175	-0.4	0.985	0.881		
751082997	724.889	18.772	-4.6	0.177	0.031	Tax_Id=9606 Gene_Symbol=LSAMP Limbic system-associated membrane p	VTVNYPPTITESK
751082998	725.39	18.766	-3.8	0.292	0.09	Tax_Id=9606 Gene_Symbol=LSAMP Limbic system-associated membrane p	VTVNYPPTITESK
751082999	725.894	18.717	-48.9	0.029	0.131	Tax_Id=9606 Gene_Symbol=LSAMP Limbic system-associated membrane p	VTVNYPPTITESK
751083000	820.397	41.32	6.9	0.405	0.674		
751083001	821.401	41.293	6.3	0.392	0.701		
751083002	791.654	40.576	5.9	0.769	0.439		
751083003	791.904	40.572	5.8	0.556	0.394		
751083004	792.155	40.586	2.7	0.651	0.533		
751083005	792.406	40.647	2.9	0.628	0.487		
751083006	792.658	40.589	4.4	0.655	0.491		
751083007	674.828	18.821	-3.7	0.745	0.1		
751083008	675.329	18.801	-5.1	0.645	0.089		
751083009	886.435	32.109	1.4	0.706	0.995		
751083010	886.939	32.123	0.9	0.792	0.75		
751083011	887.438	32.231	-6.2	0.266	0.233		
751083012	744.504	45.299	-0.3	0.963	0.32		
751083013	744.686	45.335	1.6	0.793	0.492		
751083014	744.868	45.339	1.2	0.822	0.43		
751083015	745.051	45.346	1.6	0.801	0.53		
751083016	776.411	46.234	4.2	0.573	0.446		
751083017	776.745	46.236	4.6	0.507	0.659		
751083018	777.079	46.238	4	0.579	0.444		
751083019	807.392	15.438	8.9	0.2	0.923		
751083020	808.396	15.453	16.3	0.53	0.975		
751083021	430.738	31.261	38.4	0.133	0.799		
751083022	431.24	31.275	15.1	0.671	0.734		
751083023	753.362	14.369	21	0.283	0.477		
751083024	746.396	14.852	-7.2	0.593	0.119		
751083025	747.399	14.842	-29.9	0.122	0.035		
751083026	1143.205	36.506	-0.7	0.836	0.179		
751083027	1143.541	36.516	-1.7	0.57	0.091		
751083028	1143.875	36.517	-0.9	0.816	0.405		
751083029	1144.209	36.531	-4.6	0.171	0.055		
751083030	1144.543	36.528	-11.8	0.16	0.199		
751083031	744.594	45.344	0.9	0.882	0.435	Tax_Id=9606 Gene_Symbol=SETD7 Putative uncharacterized protein SETD7	HGVC[160.0302]WIY[243.0293]Y[243.0293]PDG
751083032	744.777	45.305	-0.1	0.987	0.288	Tax_Id=9606 Gene_Symbol=SETD7 Putative uncharacterized protein SETD7	HGVC[160.0302]WIY[243.0293]Y[243.0293]PDG
751083033	744.959	45.31	-1.6	0.755	0.142	Tax_Id=9606 Gene_Symbol=SETD7 Putative uncharacterized protein SETD7	HGVC[160.0302]WIY[243.0293]Y[243.0293]PDG
751083034	623.763	24.543	-2.2	0.683	0.031		
751083035	624.264	24.548	-6.3	0.524	0.03		
751083036	569.268	25.152	6.5	0.298	0.747		
751083037	569.46	24.96	18.1	0.368	0.511		
751083038	569.66	24.982	6.9	0.635	0.28		
751083039	569.86	25.003	9.1	0.596	0.302		
751083040	570.061	24.987	16.2	0.451	0.328		
751083041	811.772	27.207	-9.8	0.555	0.098		
751083042	811.973	27.204	-0.1	0.991	0.275		
751083043	812.173	27.205	1	0.908	0.523		
751083044	812.374	27.173	1.5	0.804	0.366		
751083045	812.575	27.214	0.1	0.994	0.587		
751083046	812.775	27.213	-9.4	0.634	0.254		
751083047	549.652	21.197	4.8	0.724	0.408		
751083048	1023.066	39.067	3.5	0.571	0.344	Tax_Id=9606 Gene_Symbol=A2M Alpha-2-macroglobulin	LLLQQVSLPELPGEYSMK
751083049	1023.565	39.06	8.6	0.49	0.456	Tax_Id=9606 Gene_Symbol=A2M Alpha-2-macroglobulin	LLLQQVSLPELPGEYSMK
751083050	1024.073	39.063	6.6	0.656	0.472	Tax_Id=9606 Gene_Symbol=A2M Alpha-2-macroglobulin	LLLQQVSLPELPGEYSMK
751083051	1024.574	39.042	8.5	0.498	0.28	Tax_Id=9606 Gene_Symbol=A2M Alpha-2-macroglobulin	LLLQQVSLPELPGEYSMK
751083052	915.493	29.046	8.1	0.257	0.783	Tax_Id=9606 Gene_Symbol=GSN Isoform 1 of Gelsolin	QTQVSVLPEGGETPLFK
751083053	915.995	29.05	9.2	0.166	0.851	Tax_Id=9606 Gene_Symbol=GSN Isoform 1 of Gelsolin	QTQVSVLPEGGETPLFK
751083054	916.496	29.064	9.4	0.447	0.931	Tax_Id=9606 Gene_Symbol=GSN Isoform 1 of Gelsolin	QTQVSVLPEGGETPLFK
751083055	1064.075	48.099	4.4	0.519	0.572		
751083056	1064.215	48.103	5.1	0.371	0.314		
751083057	1064.418	48.092	3.9	0.505	0.557		
751083058	1064.554	48.077	7.4	0.183	0.584		
751083059	1186.22	36.244	-1.4	0.755	0.267		
751083060	1186.552	36.244	-2.1	0.598	0.193		

751083061	1186.886	36.276	-1.6	0.673	0.212		
751083062	1187.219	36.299	-0.6	0.885	0.383		
751083063	1187.548	36.343	-3.5	0.32	0.08		
751083064	610.323	21.984	10.8	0.169	0.43		
751083065	610.574	21.999	3.1	0.659	0.985		
751083066	610.824	22.014	-4	0.586	0.452		
751083067	611.075	22.008	8.3	0.689	0.574		
751083068	655.627	23.992	-0.6	0.922	0.524		
751083069	953.759	41	7.5	0.213	0.887		
751083070	954.092	41.022	7.2	0.237	0.874		
751083071	954.427	40.883	8.5	0.237	0.902		
751083072	954.755	40.874	7.8	0.206	0.811		
751083073	955.094	40.921	6.2	0.47	0.573		
751083074	827.628	38.441	1.2	0.898	0.148		
751083075	827.874	38.423	2.6	0.72	0.27		
751083076	828.123	38.425	1.4	0.946	0.282		
751083077	828.373	38.432	1.5	0.913	0.809		
751083078	828.623	38.436	-4.4	0.732	0.223		
751083079	828.874	38.426	-11.9	0.5	0.16		
751083080	432.252	24.918	5.6	0.348	0.559		
751083081	432.586	24.923	13	0.566	0.733		
751083082	432.921	24.912	24.2	0.305	1		
751083083	826.178	32.713	4.8	0.403	0.265	Tax_Id=9606 Gene_Symbol=GSN Isoform 1 of Gelsolin	VHVSEEGTEPEAMLQVLGPKPALPAGTEDTAK
751083084	826.427	32.732	4.8	0.475	0.384	Tax_Id=9606 Gene_Symbol=GSN Isoform 1 of Gelsolin	VHVSEEGTEPEAMLQVLGPKPALPAGTEDTAK
751083085	826.679	32.716	5.3	0.461	0.502	Tax_Id=9606 Gene_Symbol=GSN Isoform 1 of Gelsolin	VHVSEEGTEPEAMLQVLGPKPALPAGTEDTAK
751083086	826.93	32.715	5.2	0.435	0.417	Tax_Id=9606 Gene_Symbol=GSN Isoform 1 of Gelsolin	VHVSEEGTEPEAMLQVLGPKPALPAGTEDTAK
751083087	827.182	32.733	8.4	0.345	0.633	Tax_Id=9606 Gene_Symbol=GSN Isoform 1 of Gelsolin	VHVSEEGTEPEAMLQVLGPKPALPAGTEDTAK
751083088	653.477	31.259	7.2	0.674	0.133		
751083089	653.644	31.259	7.1	0.255	0.521		
751083090	653.811	31.267	10.8	0.547	0.343		
751083091	653.979	31.194	8.1	0.54	0.318		
751083092	654.146	31.28	-15	0.515	0.369		
751083093	500.749	30.14	6.1	0.221	0.379		
751083094	501	30.141	6.1	0.19	0.537		
751083095	501.251	30.129	-2.3	0.837	0.099		
751083096	501.502	30.109	-15.5	0.64	0.232		
751083097	726.388	25.992	2.2	0.733	0.885		
751083098	479.472	15.883	-0.9	0.888	0.266		
751083099	479.723	15.881	0.7	0.917	0.375		
751083100	479.974	15.892	-11.4	0.548	0.387		
751083101	1021.136	33.897	1	0.87	0.562	Tax_Id=9606 Gene_Symbol=NCAM1 Isoform 2 of Neural cell adhesion mole	DGEQIEQEEDDEKYIFSDDSSQLTIK
751083102	1021.476	33.968	-0.1	0.993	0.587	Tax_Id=9606 Gene_Symbol=NCAM1 Isoform 2 of Neural cell adhesion mole	DGEQIEQEEDDEKYIFSDDSSQLTIK
751083103	1021.806	34.011	-1.7	0.764	0.411	Tax_Id=9606 Gene_Symbol=NCAM1 Isoform 2 of Neural cell adhesion mole	DGEQIEQEEDDEKYIFSDDSSQLTIK
751083104	1022.141	33.953	-4.4	0.415	0.259	Tax_Id=9606 Gene_Symbol=NCAM1 Isoform 2 of Neural cell adhesion mole	DGEQIEQEEDDEKYIFSDDSSQLTIK
751083105	658.361	17.509	-18.5	0.125	0.134	Tax_Id=9606 Gene_Symbol=SCG3 Secretogranin-3	GILDKEEAEAIK
751083106	658.862	17.515	-9.6	0.364	0.137	Tax_Id=9606 Gene_Symbol=SCG3 Secretogranin-3	GILDKEEAEAIK
751083107	659.364	17.531	-45.4	0.02	0.004	Tax_Id=9606 Gene_Symbol=SCG3 Secretogranin-3	GILDKEEAEAIK
751083108	559.586	21.973	1.3	0.769	0.261	Tax_Id=9606 Gene_Symbol=IGFBP4 Insulin-like growth factor-binding protei	GELDC[160.0302]HQLADSFRE
751083109	559.92	21.975	1.4	0.751	0.247	Tax_Id=9606 Gene_Symbol=IGFBP4 Insulin-like growth factor-binding protei	GELDC[160.0302]HQLADSFRE
751083110	560.254	21.983	-6.7	0.514	0.127	Tax_Id=9606 Gene_Symbol=IGFBP4 Insulin-like growth factor-binding protei	GELDC[160.0302]HQLADSFRE
751083111	681.302	33.337	4.2	0.694	0.249		
751083112	681.502	33.32	10.6	0.415	0.494		
751083113	681.702	33.295	5.6	0.422	0.313		
751083114	681.903	33.321	7.6	0.614	0.39		
751083115	682.103	33.33	-6.7	0.653	0.047		
751083116	1098.523	28.253	-0.7	0.914	0.875		
751083117	1099.526	28.227	0.4	0.953	0.907		
751083118	1337.692	29.819	5.2	0.633	0.157		
751083119	1338.695	29.823	6.8	0.517	0.151		
751083120	1339.696	29.839	6.1	0.668	0.661		
751083121	1100.255	33.668	6	0.321	0.708		
751083122	1100.504	33.648	9.2	0.082	0.365		
751083123	1100.756	33.67	12.2	0.036	0.284		
751083124	1101.008	33.676	11.9	0.036	0.33		
751083125	1101.258	33.675	14.9	0.036	0.206		
751083126	633.53	18.266	5.8	0.684	0.842		
751083127	633.781	18.27	9.1	0.528	0.445		
751083128	634.032	18.265	5.1	0.754	0.436		
751083129	634.282	18.272	0.2	0.994	0.663		
751083130	566.116	34.44	17.2	0.326	0.464		
751083131	566.283	34.433	-7.8	0.757	0.379		
751083132	566.45	34.446	-27.4	0.398	0.25		
751083133	566.617	34.455	7.4	0.615	0.27		
751083134	566.784	34.447	6	0.673	0.348		
751083135	566.951	34.45	-6.5	0.815	0.615		
751083136	730.37	34.499	3.1	0.636	0.499		
751083137	730.704	34.453	6.4	0.396	0.69		
751083138	379.696	23.868	9.3	0.201	0.447		
751083139	380.198	23.844	38.3	0.121	0.98		
751083140	709.805	15.713	2.5	0.806	0.404	Tax_Id=9606 Gene_Symbol=A2M Alpha-2-macroglobulin	HYDGSYSTFGER
751083141	710.308	15.732	0.5	0.977	0.35	Tax_Id=9606 Gene_Symbol=A2M Alpha-2-macroglobulin	HYDGSYSTFGER
751083142	710.808	15.722	6	0.806	0.718	Tax_Id=9606 Gene_Symbol=A2M Alpha-2-macroglobulin	HYDGSYSTFGER
751083143	455.773	18.578	5.2	0.821	0.544		
751083144	456.275	18.565	-2.6	0.913	0.782		
751083145	774.024	19.887	-0.7	0.929	0.11	Tax_Id=9606 Gene_Symbol=SPP1 Isoform A of Osteopontin	EFHSHEFHSHEDMLVVDPK
751083146	774.358	19.901	0.3	0.951	0.124	Tax_Id=9606 Gene_Symbol=SPP1 Isoform A of Osteopontin	EFHSHEFHSHEDMLVVDPK
751083147	774.693	19.883	-0.6	0.962	0.178	Tax_Id=9606 Gene_Symbol=SPP1 Isoform A of Osteopontin	EFHSHEFHSHEDMLVVDPK
751083148	775.027	19.864	-0.1	0.996	0.065	Tax_Id=9606 Gene_Symbol=SPP1 Isoform A of Osteopontin	EFHSHEFHSHEDMLVVDPK
751083149	544.306	34.485	3.2	0.196	0.191		
751083150	545.311	34.504	-0.2	0.984	0.308		
751083151	570.833	27.415	3.6	0.515	0.889		
751083152	571.335	27.42	4.3	0.565	0.772		
751083153	598.785	15.326	9.4	0.201	0.795		

751083154	599.286	15.383	16.5	0.165	0.928		
751083155	945.459	35.371	-4.8	0.707	0.193		
751083156	945.961	35.378	-13.3	0.529	0.199		
751083157	946.46	35.419	-31.6	0.061	0.01		
751083158	650.225	43.524	5.8	0.347	0.643		
751083159	650.367	43.587	7.3	0.244	0.525		
751083160	650.511	43.538	6.1	0.326	0.61		
751083161	650.655	43.55	-4.1	0.57	0.574		
751083162	650.798	43.522	1.9	0.827	0.687		
751083163	830.698	21.221	-5.3	0.249	0.747	Tax_Id=9606 Gene_Symbol=CP Ceruloplasmin	M[147.0355]FTTAPDQVDKEDEDFQESNK
751083164	831.032	21.222	-1.8	0.712	0.993	Tax_Id=9606 Gene_Symbol=CP Ceruloplasmin	M[147.0355]FTTAPDQVDKEDEDFQESNK
751083165	461.232	17.496	11.2	0.112	0.729		
751083166	461.567	17.504	11.1	0.08	0.88		
751083167	461.901	17.521	9.5	0.673	0.811		
751083168	815.057	43.365	-1.2	0.829	0.425		
751083169	815.389	43.359	-1.2	0.84	0.353		
751083170	815.722	43.364	-1.1	0.822	0.273		
751083171	816.056	43.375	-6.8	0.371	0.069		
751083172	816.394	43.455	-5.8	0.304	0.144		
751083173	433.911	14.324	15.5	0.049	0.937		
751083174	434.245	14.316	24.2	0.02	0.749		
751083175	434.579	14.324	14.5	0.456	0.945		
751083176	633.003	35.363	1	0.893	0.993		
751083177	633.338	35.361	2.7	0.734	0.856		
751083178	633.672	35.371	-9.8	0.503	0.064		
751083179	825.395	17.143	10.1	0.25	0.874		
751083180	825.898	17.161	10.2	0.521	0.882		
751083181	826.401	17.165	34.3	0.236	0.565		
751083182	602.555	28.646	7.4	0.401	0.74		
751083183	602.806	28.652	3.8	0.565	0.681		
751083184	603.057	28.642	9.4	0.362	0.691		
751083185	603.307	28.642	-7.1	0.568	0.125		
751083186	559.536	17.574	-2.6	0.85	0.126		
751083187	559.786	17.575	0.4	0.976	0.167		
751083188	560.037	17.582	9.4	0.495	0.256		
751083189	411.243	18.539	5.2	0.411	0.996		
751083190	411.746	18.496	1.2	0.941	0.682		
751083191	888.915	25.409	-37.2	0	0.002		
751083192	889.416	25.419	-25.7	0.005	0.017		
751083193	889.917	25.432	-22.9	0.204	0.152		
751083194	421.725	20.839	5.8	0.388	0.508		
751083195	589.326	34.5	-8.6	0.603	0.159	Tax_Id=9606 Gene_Symbol=HRG Histidine-rich glycoprotein	KGEVLPLPEANFPSFPLPHHK
751083196	589.576	34.495	-3.9	0.714	0.174	Tax_Id=9606 Gene_Symbol=HRG Histidine-rich glycoprotein	KGEVLPLPEANFPSFPLPHHK
751083197	589.827	34.492	-3.7	0.734	0.157	Tax_Id=9606 Gene_Symbol=HRG Histidine-rich glycoprotein	KGEVLPLPEANFPSFPLPHHK
751083198	590.078	34.489	-16.4	0.263	0.16	Tax_Id=9606 Gene_Symbol=HRG Histidine-rich glycoprotein	KGEVLPLPEANFPSFPLPHHK
751083199	1198.056	39.813	4.3	0.465	0.303		
751083200	1199.057	39.823	5.4	0.476	0.237		
751083201	990.982	31.649	-1.7	0.665	0.32	Tax_Id=9606 Gene_Symbol=APP Isoform L-APP733 of Amyloid beta A4 prot	EQNYSDDVLANMISEPR
751083202	991.467	31.261	1.5	0.947	0.441	Tax_Id=9606 Gene_Symbol=APP Isoform L-APP733 of Amyloid beta A4 prot	EQNYSDDVLANMISEPR
751083203	991.966	31.255	-16.3	0.331	0.165	Tax_Id=9606 Gene_Symbol=APP Isoform L-APP733 of Amyloid beta A4 prot	EQNYSDDVLANMISEPR
751083204	751.695	40.239	5.6	0.265	0.769		
751083205	752.039	40.113	3.6	0.616	0.326		
751083206	752.379	40.241	4.4	0.393	0.623		
751083207	752.706	40.15	5.8	0.658	0.816		
751083208	608.972	39.038	2.3	0.605	0.903	Tax_Id=9606 Gene_Symbol=HRG Histidine-rich glycoprotein	ADLFYDVEALDLESPK
751083209	609.306	39.042	2.2	0.593	0.808	Tax_Id=9606 Gene_Symbol=HRG Histidine-rich glycoprotein	ADLFYDVEALDLESPK
751083210	609.641	39.023	1.9	0.82	0.697	Tax_Id=9606 Gene_Symbol=HRG Histidine-rich glycoprotein	ADLFYDVEALDLESPK
751083211	609.975	39.027	1.1	0.948	0.712	Tax_Id=9606 Gene_Symbol=HRG Histidine-rich glycoprotein	ADLFYDVEALDLESPK
751083212	815.401	19.368	-16.7	0.224	0.252		
751083213	815.903	19.375	-6.8	0.246	0.112		
751083214	816.406	19.394	-36.3	0.062	0.069		
751083215	670.454	29.327	8.6	0.344	0.935		
751083216	671.457	29.349	-2.5	0.926	0.732		
751083217	690.312	29.778	2.6	0.566	0.905		
751083218	690.647	29.775	1.9	0.639	0.933		
751083219	690.981	29.777	-1	0.823	0.916		
751083220	562.289	16.714	3.7	0.87	0.286		
751083221	447.517	15.348	11.6	0.493	0.208		
751083222	447.851	15.351	0.9	0.966	0.205		
751083223	448.184	15.347	19.2	0.508	0.756		
751083224	701.004	29.436	2.2	0.61	0.2		
751083225	701.339	29.378	3.2	0.45	0.657		
751083226	716.332	22.389	-2.5	0.749	0.503		
751083227	717.337	22.489	-4	0.743	0.101		
751083228	545.919	19.69	5.5	0.229	0.965		
751083229	546.254	19.542	2.9	0.439	0.654		
751083230	545.584	19.827	12.5	0.072	0.61		
751083231	661.631	23.988	-0.4	0.948	0.61		
751083232	661.965	24.006	-1.5	0.803	0.504		
751083233	946.486	39.294	9	0.218	0.466		
751083234	946.987	39.297	11.7	0.16	0.359		
751083235	947.488	39.337	1.6	0.864	0.437		
751083236	835.426	24.949	-10.8	0.165	0.061		
751083237	836.429	24.964	-30.9	0.037	0.024		
751083238	701.318	17.189	-1.8	0.865	0.425		
751083239	735.345	28.835	2.5	0.538	0.267		
751083240	735.679	28.867	1.1	0.902	0.231		
751083241	736.013	28.865	3.3	0.804	0.285		
751083242	580.816	28.779	7.2	0.604	0.678	Tax_Id=9606 Gene_Symbol=ITIH1 Inter-alpha-trypsin inhibitor heavy chain H	EVAFDLEIPK
751083243	581.318	28.773	7.8	0.712	0.635	Tax_Id=9606 Gene_Symbol=ITIH1 Inter-alpha-trypsin inhibitor heavy chain H	EVAFDLEIPK
751083244	1017.944	28.257	-36.9	0.113	0.127		
751083245	1018.194	28.251	4.3	0.747	0.491		
751083246	1018.444	28.253	3	0.716	0.643		

751083247	1018.693	28.247	-4.2	0.622	0.162		
751083248	1018.946	28.266	-27.1	0.138	0.068		
751083249	1153.853	31.361	5.4	0.359	0.917		
751083250	1154.188	31.378	2.7	0.561	0.844		
751083251	1154.523	31.356	4.7	0.262	0.763		
751083252	1154.858	31.368	3.9	0.459	0.668		
751083253	665.338	19.807	-0.3	0.959	0.248		
751083254	665.674	19.711	2	0.725	0.484		
751083255	666.008	19.727	0	1	0.644		
751083256	1251.266	32.159	6.4	0.284	0.815		
751083257	1251.514	32.171	11.8	0.106	0.735		
751083258	1251.773	32.199	4.9	0.322	0.658		
751083259	1252.027	32.207	7.9	0.179	0.942		
751083260	1252.275	32.155	2.1	0.791	0.177		
751083261	1277.62	46.46	-3	0.595	0.221		
751083262	1277.883	46.559	-0.9	0.895	0.426		
751083263	1278.134	46.608	-0.3	0.953	0.287		
751083264	1278.382	46.644	-1.5	0.745	0.126		
751083265	697.374	35.529	5.9	0.415	0.69		
751083266	697.878	35.511	4.1	0.672	0.558		
751083267	967.691	40.547	0.9	0.787	0.528		
751083268	423.564	17.762	6.5	0.439	0.872		
751083269	423.899	17.797	7.8	0.698	0.55		
751083270	424.237	17.827	-3.1	0.855	0.512		
751083271	649.366	25.346	4.3	0.583	0.941		
751083272	649.866	25.41	0.4	0.975	0.473		
751083273	650.366	25.434	-0.7	0.968	0.888		
751083274	954.482	32.13	4.2	0.395	0.796		
751083275	954.984	32.147	3.6	0.441	0.771		
751083276	556.805	18.366	11.7	0.375	0.625		
751083277	557.305	18.356	14.1	0.394	0.895		
751083278	628.64	27.193	4.5	0.465	0.838	Tax_Id=9606 Gene_Symbol=F2 Prothrombin (Fragment)	TFGSGEADC[160.0302]GLRPLFEK
751083279	628.975	27.185	6.5	0.125	0.308	Tax_Id=9606 Gene_Symbol=F2 Prothrombin (Fragment)	TFGSGEADC[160.0302]GLRPLFEK
751083280	629.309	27.198	5.3	0.415	0.797	Tax_Id=9606 Gene_Symbol=F2 Prothrombin (Fragment)	TFGSGEADC[160.0302]GLRPLFEK
751083281	593.047	25.11	7.2	0.614	0.857		
751083282	593.247	25.1	8.5	0.22	0.966		
751083283	593.448	25.067	8	0.579	0.792		
751083284	593.649	25.122	-29.8	0.269	0.234		
751083285	619.421	66.209	3.9	0.613	0.175		
751083286	620.424	66.219	4.1	0.597	0.175		
751083287	591.292	25.957	4	0.497	0.945		
751083288	591.627	25.941	14.7	0.333	0.302		
751083289	591.963	25.968	12.7	0.314	0.937		
751083290	355.202	16.696	-30.3	0.345	0.221	Tax_Id=9606 Gene_Symbol=CFH Isoform 1 of Complement factor H	IDVHLVPDR
751083291	355.537	16.701	-4.2	0.765	0.197	Tax_Id=9606 Gene_Symbol=CFH Isoform 1 of Complement factor H	IDVHLVPDR
751083292	743.589	16.85	3.2	0.885	0.157		
751083293	743.84	16.851	6.4	0.529	0.236		
751083294	744.091	16.856	4.8	0.784	0.169		
751083295	744.344	16.815	6.8	0.672	0.514		
751083296	686.661	28.829	3.1	0.631	0.968		
751083297	686.849	29.007	16.8	0.277	0.894		
751083298	686.995	28.835	1.6	0.8	0.972		
751083299	707.077	21.787	14.3	0.181	0.408		
751083300	707.328	21.784	39.1	0.034	0.202		
751083301	707.578	21.792	17.8	0.049	0.421		
751083302	707.831	21.785	5.4	0.694	0.553		
751083303	854.505	23.471	5.1	0.428	0.905		
751083304	855.509	23.477	14	0.589	0.572		
751083305	507.319	17.394	9.5	0.566	0.322		
751083306	508.323	17.386	9.7	0.729	0.407		
751083307	477.804	16.149	8.6	0.584	0.42		
751083308	478.306	16.153	0	0.999	0.55		
751083309	568.788	37.748	7.1	0.327	0.112		
751083310	569.29	37.779	10.8	0.54	0.313		
751083311	569.791	37.815	25.9	0.368	0.195		
751083312	427.449	27.882	26.9	0.295	0.64		
751083313	427.65	27.877	24.5	0.39	0.445		
751083314	427.851	27.872	35.7	0.19	0.742		
751083315	428.051	27.817	31.5	0.224	0.596		
751083316	972.453	37.158	-22.2	0.009	0.01		
751083317	972.786	37.18	-8.9	0.163	0.08		
751083318	973.12	37.193	-5.5	0.381	0.217		
751083319	973.454	37.196	-21.1	0.004	0.003		
751083320	735.87	16.618	-11.3	0.572	0.461	Tax_Id=9606 Gene_Symbol=IGFBP7 Insulin-like growth factor-binding protei	GTC[160.0302]EQGPSIVTPPK
751083321	736.376	16.61	9.7	0.666	0.436	Tax_Id=9606 Gene_Symbol=IGFBP7 Insulin-like growth factor-binding protei	GTC[160.0302]EQGPSIVTPPK
751083322	736.878	16.634	3.3	0.914	0.45	Tax_Id=9606 Gene_Symbol=IGFBP7 Insulin-like growth factor-binding protei	GTC[160.0302]EQGPSIVTPPK
751083323	759.362	22.842	1.8	0.896	0.427		
751083324	760.365	22.741	5.4	0.796	0.831		
751083325	651.677	41.873	-6.9	0.515	0.057		
751083326	652.011	41.884	2.4	0.758	0.143		
751083327	652.345	41.96	5.2	0.458	0.402		
751083328	636.655	32.179	3.2	0.487	0.743		
751083329	556.293	25.92	-1.1	0.819	0.15	Tax_Id=9606 Gene_Symbol=CDH13 cDNA FLJ52398, highly similar to Cadh	TPHAEDMAELVIVGGK
751083330	556.624	26.008	-4.3	0.406	0.122	Tax_Id=9606 Gene_Symbol=CDH13 cDNA FLJ52398, highly similar to Cadh	TPHAEDMAELVIVGGK
751083331	556.958	25.968	-13.1	0.077	0.027	Tax_Id=9606 Gene_Symbol=CDH13 cDNA FLJ52398, highly similar to Cadh	TPHAEDMAELVIVGGK
751083332	1270.144	48.53	-4.5	0.365	0.159		
751083333	575.395	66.302	1.2	0.872	0.325		
751083334	576.398	66.375	0.3	0.967	0.354		
751083335	918.418	31.431	3.4	0.547	0.397		
751083336	918.919	31.438	3.9	0.647	0.314		
751083337	696.859	25.768	-1.6	0.606	0.203		
751083338	697.361	25.791	-2.1	0.502	0.19		
751083339	694.846	14.534	-7.2	0.106	0.128		

751083340	695.342	14.464	-8.5	0.103	0.107		
751083341	695.851	14.584	2	0.606	0.653		
751083342	923.206	29.547	-1.4	0.946	0.309		
751083343	923.406	29.546	6.6	0.523	0.717		
751083344	923.606	29.557	7.7	0.607	0.561		
751083345	923.807	29.545	8.1	0.719	0.482		
751083346	924.007	29.547	19.3	0.519	0.523		
751083347	924.208	29.544	-3.8	0.806	0.162		
751083348	706.645	16.476	-5.3	0.641	0.292		
751083349	706.979	16.462	-8.4	0.694	0.18		
751083350	707.316	16.434	7.5	0.388	0.579		
751083351	397.018	15.785	7.1	0.296	0.716		
751083352	397.185	15.778	4.6	0.48	0.944		
751083353	397.352	15.784	1.2	0.896	0.308		
751083354	397.519	15.787	-6.5	0.699	0.04		
751083355	724.695	24.886	4.1	0.355	0.765		
751083356	725.031	24.858	4	0.335	0.97		
751083357	725.368	24.877	5.3	0.383	0.738		
751083358	725.697	24.89	3.6	0.75	0.526		
751083359	436.267	16.089	9.7	0.007	0.853		
751083360	436.769	16.119	11.3	0.106	0.924		
751083361	685.373	22.388	1.1	0.827	0.701		
751083362	685.875	22.41	-1.5	0.759	0.461		
751083363	686.378	22.499	-5.8	0.595	0.245		
751083364	959.015	43.553	4.1	0.596	0.316		
751083365	959.517	43.564	3.5	0.671	0.196		
751083366	960.019	43.571	4	0.68	0.177		
751083367	632.987	22.591	-0.7	0.952	0.094		
751083368	663.563	30.202	4.3	0.757	0.247		
751083369	663.814	30.201	3.5	0.634	0.314		
751083370	664.064	30.2	4.8	0.703	0.228		
751083371	664.315	30.219	4.4	0.533	0.61		
751083372	664.565	30.198	26.8	0.349	0.957		
751083373	672.687	36.027	8.2	0.362	0.427	Tax_Id=9606 Gene_Symbol=AFM Afamin	KSDVGFLPPFPTLDPEEK
751083374	673.022	36.024	6.6	0.145	0.687	Tax_Id=9606 Gene_Symbol=AFM Afamin	KSDVGFLPPFPTLDPEEK
751083375	673.358	36.026	11.8	0.343	0.908	Tax_Id=9606 Gene_Symbol=AFM Afamin	KSDVGFLPPFPTLDPEEK
751083376	673.111	37.82	-1.8	0.718	0.156		
751083377	673.361	37.892	-1.1	0.808	0.209		
751083378	673.612	37.835	-0.7	0.891	0.172		
751083379	673.863	37.774	-3.2	0.544	0.092		
751083380	674.115	37.783	-13	0.184	0.074		
751083381	655.961	24	0.5	0.93	0.559		
751083382	656.298	23.991	-0.7	0.908	0.548		
751083383	656.631	23.967	1.8	0.791	0.661		
751083384	501.77	22.649	3.4	0.765	0.266		
751083385	502.274	22.647	2.9	0.843	0.144		
751083386	558.29	15.549	5.2	0.826	0.285		
751083387	558.773	15.512	7	0.661	0.408		
751083388	521.299	22.85	5.8	0.656	0.978		
751083389	1030.568	25.811	3.1	0.793	0.231		
751083390	1031.572	25.815	-2.4	0.858	0.117		
751083391	1252.89	36.197	3.1	0.312	0.828		
751083392	1253.225	36.215	-0.5	0.897	0.55		
751083393	1253.561	36.196	1.2	0.774	0.834		
751083394	1253.896	36.215	0	0.999	0.616		
751083395	1254.247	36.215	0.7	0.867	0.638		
751083396	579.278	15.351	-1.7	0.908	0.328		
751083397	580.279	15.261	-24.7	0.276	0.282		
751083398	430.773	66.118	2	0.785	0.402		
751083399	431.274	66.114	2.6	0.704	0.299		
751083400	520.792	17.759	12.9	0.382	0.913		
751083401	521.293	17.77	14.5	0.545	0.926		
751083402	749.371	21.828	6.3	0.295	0.823		
751083403	749.872	21.828	2.6	0.773	0.257		
751083404	750.373	21.871	6.5	0.639	0.867		
751083405	1234.566	40.662	1.1	0.794	0.561		
751083406	1234.9	40.659	-0.6	0.877	0.325		
751083407	1235.234	40.647	0	0.992	0.339		
751083408	1235.568	40.608	-0.2	0.965	0.365		
751083409	1235.902	40.541	-0.6	0.886	0.272		
751083410	671.673	37.181	-3.5	0.761	0.109		
751083411	672.007	37.177	-2.5	0.812	0.075		
751083412	672.343	37.176	-3.2	0.671	0.057		
751083413	672.676	37.197	-16.3	0.176	0.027		
751083414	731.849	25.633	-0.4	0.899	0.593		
751083415	732.352	25.624	1.6	0.652	0.713		
751083416	732.856	25.641	-1.2	0.942	0.57		
751083417	634.352	29.719	6	0.159	0.731		
751083418	634.854	29.7	7.6	0.115	0.5		
751083419	925.95	28.209	7.1	0.325	0.722		
751083420	926.452	28.208	8.6	0.513	0.642		
751083421	926.953	28.214	15.1	0.273	0.812		
751083422	927.451	28.167	-13.4	0.37	0.063		
751083423	708.388	37.808	-2.1	0.51	0.208	Tax_Id=9606 Gene_Symbol=CDH2 Cadherin-2	LSLKPTLTEESVKESAEVEEIVFPR
751083424	708.639	37.799	-2.5	0.339	0.155	Tax_Id=9606 Gene_Symbol=CDH2 Cadherin-2	LSLKPTLTEESVKESAEVEEIVFPR
751083425	708.891	37.866	-2.8	0.407	0.175	Tax_Id=9606 Gene_Symbol=CDH2 Cadherin-2	LSLKPTLTEESVKESAEVEEIVFPR
751083426	709.137	37.912	-4.8	0.193	0.055	Tax_Id=9606 Gene_Symbol=CDH2 Cadherin-2	LSLKPTLTEESVKESAEVEEIVFPR
751083427	565.264	19.604	10.1	0.239	0.307		
751083428	565.765	19.614	-7	0.838	0.839		
751083429	538.28	17.147	-1.8	0.609	0.054		
751083430	538.615	17.139	-0.7	0.864	0.175		
751083431	538.949	17.068	0.9	0.88	0.346		
751083432	820.392	17.166	11.7	0.256	0.918		

751083433	820.894	17.173	9	0.334	0.831		
751083434	821.395	17.2	13.3	0.713	0.858		
751083435	487.917	17.748	3.4	0.467	0.467		
751083436	488.252	17.724	4	0.484	0.521		
751083437	488.586	17.737	22	0.356	0.992		
751083438	998.728	43.764	14.1	0.245	0.796		
751083439	998.981	43.603	15.3	0.495	0.651		
751083440	999.232	43.643	9.4	0.201	0.989		
751083441	999.481	43.714	6.4	0.33	0.914		
751083442	999.733	43.727	12.5	0.217	0.902		
751083443	585.864	42.356	0.1	0.992	0.297		
751083444	586.366	42.362	NA	NA	NA		
751083445	586.868	42.374	-40.1	0.013	0.002		
751083446	908.435	18.81	-2.4	0.853	0.348		
751083447	908.938	18.806	2.1	0.893	0.269		
751083448	924.847	30.269	4.6	0.276	1		
751083449	925.349	30.276	4.9	0.212	0.948		
751083450	925.849	30.27	3.3	0.417	0.88		
751083451	926.354	30.302	-3.5	0.796	0.453		
751083452	546.821	23.982	11.2	0.397	0.896	Tax_Id=9606 Gene_Symbol=C3 Complement C3 (Fragment)	NTLIIYLDK
751083453	547.323	23.957	-21.1	0.295	0.326	Tax_Id=9606 Gene_Symbol=C3 Complement C3 (Fragment)	NTLIIYLDK
751083454	565.061	23.203	10.7	0.12	0.471		
751083455	565.312	23.197	12.2	0.091	0.303		
751083456	565.564	23.215	5.7	0.319	0.81		
751083457	565.815	23.258	-7.7	0.535	0.223		
751083458	1064.515	30.147	7.4	0.408	0.916		
751083459	1065.518	30.13	9.5	0.64	0.669		
751083460	599.677	37.709	8.4	0.217	0.362		
751083461	599.843	37.667	6.5	0.359	0.202		
751083462	600.01	37.667	13.8	0.395	0.715		
751083463	600.178	37.639	6.2	0.389	0.212		
751083464	600.343	37.656	7.8	0.324	0.248		
751083465	670.352	35.438	-2.9	0.629	0.085		
751083466	670.55	35.409	-34.8	0.187	0.21		
751083467	670.749	35.407	-37.9	0.215	0.173		
751083468	670.951	35.421	-1.3	0.816	0.188		
751083469	671.152	35.374	-19.9	0.141	0.098		
751083470	799.037	39.788	4.9	0.59	0.243		
751083471	800.041	39.802	4.2	0.561	0.358		
751083472	551.286	24.367	2.5	0.906	0.236		
751083473	551.788	24.36	-4.7	0.83	0.039		
751083474	362.201	16.329	34.3	0.304	0.439	Tax_Id=9606 Gene_Symbol=HPX Hemopexin	GDKVWVYPPEKK
751083475	362.452	16.328	12.4	0.639	0.863	Tax_Id=9606 Gene_Symbol=HPX Hemopexin	GDKVWVYPPEKK
751083476	362.702	16.315	38.8	0.325	0.656	Tax_Id=9606 Gene_Symbol=HPX Hemopexin	GDKVWVYPPEKK
751083477	728.971	16.56	-17.7	0.381	0.066		
751083478	729.306	16.559	-11	0.624	0.384		
751083479	729.64	16.566	-9.6	0.686	0.152		
751083480	775.372	26.942	-1.4	0.824	0.324		
751083481	775.623	27.015	3.6	0.467	0.905		
751083482	775.873	27.064	1.2	0.811	0.468		
751083483	776.123	27.19	3.1	0.592	0.78		
751083484	655.313	32.855	7.4	0.164	0.514		
751083485	655.51	32.852	7.8	0.365	0.855		
751083486	655.711	32.87	6.8	0.407	0.8		
751083487	655.91	32.888	11.2	0.217	0.667		
751083488	656.111	32.856	4.1	0.741	0.822		
751083489	714.912	35.647	-0.2	0.987	0.451		
751083490	715.413	35.644	1.5	0.876	0.176		
751083491	776.418	35.036	-2.3	0.704	0.268		
751083492	640.306	17.129	-3.8	0.457	0.315		
751083493	1150.177	36.431	-0.4	0.908	0.338		
751083494	1150.515	36.48	-0.3	0.942	0.405		
751083495	1150.85	36.481	0.9	0.826	0.643		
751083496	1151.186	36.484	-0.6	0.872	0.548		
751083497	1151.522	36.501	0.6	0.886	0.716		
751083498	791.069	41.041	2	0.805	0.229		
751083499	791.403	41.037	1.9	0.789	0.184		
751083500	791.738	41.029	2.1	0.772	0.24		
751083501	792.072	41.034	1.3	0.861	0.305		
751083502	382.258	15.066	3.4	0.82	0.246	Tax_Id=9606 Gene_Symbol=OGN Osteoglycin	LPVLPPK
751083503	382.76	15.031	9.7	0.562	0.387	Tax_Id=9606 Gene_Symbol=OGN Osteoglycin	LPVLPPK
751083504	699.682	20.857	0.1	0.973	0.74	Tax_Id=9606 Gene_Symbol=AHSG Alpha-2-HS-glycoprotein	HTFM[147.0355]GVVSLGSPSGEVSHPR
751083505	700.016	20.858	0.7	0.933	0.349	Tax_Id=9606 Gene_Symbol=AHSG Alpha-2-HS-glycoprotein	HTFM[147.0355]GVVSLGSPSGEVSHPR
751083506	700.352	20.801	-4	0.533	0.373	Tax_Id=9606 Gene_Symbol=AHSG Alpha-2-HS-glycoprotein	HTFM[147.0355]GVVSLGSPSGEVSHPR
751083507	700.684	20.831	-16.4	0.247	0.5	Tax_Id=9606 Gene_Symbol=AHSG Alpha-2-HS-glycoprotein	HTFM[147.0355]GVVSLGSPSGEVSHPR
751083508	518.734	14.88	6.3	0.692	0.277		
751083509	519.238	14.881	12	0.473	0.457		
751083510	527.266	17.868	7.1	0.279	0.565		
751083511	527.769	17.869	-2.9	0.91	0.803		
751083512	1159.568	40.105	1.3	0.855	0.285		
751083513	1160.574	40.106	4.2	0.722	0.844		
751083514	1062.004	39.41	-0.6	0.921	0.85		
751083515	1062.504	39.365	-0.1	0.986	0.366		
751083516	1063.006	39.378	0.3	0.941	0.355		
751083517	1063.507	39.385	-1.8	0.699	0.581		
751083518	570.838	29.85	2.4	0.724	0.956		
751083519	571.339	29.775	4.9	0.686	0.531		
751083520	627.302	22.571	11.3	0.191	0.461		
751083521	627.801	22.573	9.5	0.516	0.956		
751083522	963.212	29.091	-29.8	0.077	0.125		
751083523	963.458	29.08	-4.7	0.21	0.033		
751083524	963.709	29.11	-8.3	0.038	0.008		
751083525	963.96	29.097	-8.3	0.308	0.095		

751083526	964.214	29.09	-26.2	0.037	0.042		
751083527	641.269	20.655	10.2	0.427	0.954		
751083528	641.772	20.671	28	0.471	0.757		
751083529	757.45	18.178	-11.4	0.428	0.126		
751083530	758.454	18.157	-47.6	0.075	0.072		
751083531	874.37	19.572	7.7	0.328	0.638		
751083532	874.871	19.584	8.8	0.69	0.484		
751083533	529.326	23.017	0.6	0.946	0.446		
751083534	529.828	23.024	5.6	0.728	0.405		
751083535	501.896	15.665	3.1	0.757	0.302		
751083536	502.23	15.617	5.1	0.65	0.315		
751083537	502.563	15.537	14.1	0.407	0.565		
751083538	679.519	27.578	-2.9	0.803	0.986		
751083539	679.719	27.591	NA	NA	NA		
751083540	679.919	27.6	-1.2	0.889	0.937		
751083541	680.12	27.623	3	0.831	0.589		
751083542	482.282	18.52	-6.9	0.346	0.091	Tax_Id=9606 Gene_Symbol=CDH2 Cadherin-2	LSLKPTLTEESVK
751083543	482.616	18.505	-13.9	0.369	0.21	Tax_Id=9606 Gene_Symbol=CDH2 Cadherin-2	LSLKPTLTEESVK
751083544	615.688	37.749	6.7	0.234	0.653		
751083545	616.022	37.752	5.2	0.375	0.547		
751083546	616.356	37.752	9.1	0.141	0.782		
751083547	772.819	45.256	6.4	0.242	0.862		
751083548	772.945	45.28	10.9	0.04	0.835		
751083549	773.072	45.305	10.4	0.054	0.997		
751083550	773.196	45.297	9.8	0.057	0.946		
751083551	773.322	45.294	10.1	0.038	0.92		
751083552	773.448	45.275	10.5	0.019	0.868		
751083553	773.573	45.261	6.1	0.351	0.441		
751083554	568.774	25.334	3.4	0.45	0.707		
751083555	580.69	39.513	13	0.564	0.586		
751083556	581.026	39.454	14.9	0.514	0.654		
751083557	581.36	39.454	15.5	0.395	0.823		
751083558	1220.887	40.615	-1.9	0.653	0.277		
751083559	1221.224	40.676	-0.9	0.824	0.36		
751083560	1221.559	40.656	-1.1	0.782	0.285		
751083561	1221.893	40.613	-1.2	0.765	0.34		
751083562	1222.23	40.602	-1.3	0.738	0.325		
751083563	783.068	40.639	14.6	0.489	0.663	Tax_Id=9606 Gene_Symbol=CP Ceruloplasmin	MYYSAVDPTKDIFTGLIGPMK
751083564	783.402	40.656	8.5	0.381	0.645	Tax_Id=9606 Gene_Symbol=CP Ceruloplasmin	MYYSAVDPTKDIFTGLIGPMK
751083565	783.734	40.622	5	0.545	0.642	Tax_Id=9606 Gene_Symbol=CP Ceruloplasmin	MYYSAVDPTKDIFTGLIGPMK
751083566	638.656	18.154	-1	0.899	0.601		
751083567	638.991	18.125	2.9	0.697	0.622		
751083568	639.324	18.134	5.8	0.547	0.838		
751083569	669.607	46.292	15.2	0.323	0.802		
751083570	670.609	46.296	15.3	0.019	0.283		
751083571	647.654	32.108	4.1	0.408	0.832		
751083572	650.62	20.915	5.5	0.385	0.58		
751083573	650.954	20.909	6.2	0.648	0.59		
751083574	651.293	20.895	4.9	0.564	0.77		
751083575	651.622	20.916	30.1	0.277	0.734		
751083576	631.659	28.374	1.7	0.796	0.32		
751083577	631.993	28.378	-20.1	0.14	0.083		
751083578	632.327	28.391	3.9	0.723	0.826		
751083579	581.302	17.072	10.7	0.623	0.674		
751083580	581.804	17.067	9.8	0.673	0.495		
751083581	954.037	29.468	-6.7	0.25	0.038	Tax_Id=9606 Gene_Symbol=NRCAM Isoform 1 of Neuronal cell adhesion mc	LLEDLVQPPTITQQSPK
751083582	954.537	29.481	-5.6	0.342	0.047	Tax_Id=9606 Gene_Symbol=NRCAM Isoform 1 of Neuronal cell adhesion mc	LLEDLVQPPTITQQSPK
751083583	955.042	29.631	-21.6	0.058	0.034	Tax_Id=9606 Gene_Symbol=NRCAM Isoform 1 of Neuronal cell adhesion mc	LLEDLVQPPTITQQSPK
751083584	1402.367	45.625	6.4	0.196	0.983		
751083585	1402.619	45.621	5.8	0.211	0.912		
751083586	1402.872	45.649	5.3	0.238	0.77		
751083587	1403.121	45.634	4.5	0.352	0.772		
751083588	1403.369	45.624	6.7	0.105	0.987		
751083589	543.301	49.986	1.2	0.891	0.3		
751083590	543.803	49.986	-1.3	0.921	0.318		
751083591	405.214	18.334	23.5	0.073	0.321		
751083592	405.716	18.33	4.5	0.863	0.664		
751083593	459.26	16.181	-2.6	0.725	0.034		
751083594	459.761	16.194	-6.9	0.607	0.052		
751083595	1089.776	23.939	13.4	0.398	0.939		
751083596	1090.11	23.944	12.7	0.178	0.553		
751083597	1090.444	23.951	14.5	0.135	0.379		
751083598	1090.778	23.945	3.6	0.8	0.383		
751083599	1091.112	23.963	18.9	0.458	0.762		
751083600	662.01	25.536	10.4	0.084	0.984		
751083601	662.343	25.588	7.2	0.192	0.409		
751083602	662.676	25.55	1.8	0.785	0.3		
751083603	681.679	18.28	5	0.821	0.639		
751083604	682.013	18.27	3.6	0.83	0.504		
751083605	682.349	18.267	6.1	0.381	0.878		
751083606	1068.962	14.731	24.9	0.235	0.401		
751083607	1069.464	14.732	9.5	0.573	0.116		
751083608	1069.965	14.728	6.9	0.776	0.226		
751083609	1070.468	14.777	-22	0.252	0.018		
751083610	681.343	33.858	4	0.724	0.134		
751083611	681.679	33.869	2.6	0.78	0.156		
751083612	682.011	33.855	-5	0.67	0.057		
751083613	519.641	30.763	9	0.66	0.509		
751083614	519.975	30.762	7.3	0.688	0.405		
751083615	520.31	30.775	-6.7	0.833	0.788		
751083616	487.772	18.415	2.3	0.889	0.063		
751083617	679.369	18.385	-5.1	0.633	0.248		
751083618	1087.617	31.463	1.1	0.871	0.558		

751083619	1088.62	31.459	13	0.642	0.569		
751083620	696.871	21.659	-9.7	0.052	0.016		
751083621	697.374	21.656	-9.5	0.15	0.185		
751083622	860.78	46.2	8.8	0.16	0.668		
751083623	860.925	46.373	8.8	0.122	0.984		
751083624	861.067	46.184	10	0.084	0.943		
751083625	861.21	46.38	6.9	0.187	0.915		
751083626	861.353	46.162	10.4	0.077	0.789		
751083627	861.496	46.183	7.8	0.238	0.512		
751083628	861.641	46.162	1.5	0.93	0.762		
751083629	800.333	27.639	-5.1	0.413	0.249		
751083630	800.834	27.646	-7.2	0.551	0.169		
751083631	801.334	27.566	2.1	0.933	0.702		
751083632	621.806	22.484	-1	0.834	0.285		
751083633	622.309	22.451	1.5	0.781	0.672		
751083634	634.844	16.061	5.2	0.806	0.86		
751083635	635.346	16.039	5.2	0.801	0.529		
751083636	827.917	19.599	0.5	0.939	0.644		
751083637	828.419	19.6	-2.4	0.687	0.256		
751083638	828.922	19.594	3	0.847	0.806		
751083639	595.294	26.297	14.6	0.54	0.58		
751083640	595.629	26.306	-1.1	0.965	0.395		
751083641	595.962	26.267	-2.9	0.681	0.095		
751083642	771.18	36.049	-0.9	0.924	0.139		
751083643	771.377	36.092	3.7	0.704	0.183		
751083644	771.582	36.054	2.1	0.858	0.17		
751083645	771.782	36.061	3.3	0.77	0.211		
751083646	771.985	36.073	5.1	0.66	0.344		
751083647	744.919	20.899	-2.2	0.885	0.698		
751083648	745.119	20.899	4.2	0.564	0.324		
751083649	745.327	20.995	5.2	0.692	0.533		
751083650	745.52	20.902	3.6	0.772	0.244		
751083651	745.721	20.9	2.7	0.868	0.956		
751083652	726.616	36.691	-2.1	0.849	0.283		
751083653	726.868	36.7	-5.3	0.501	0.107		
751083654	727.118	36.689	-3.8	0.715	0.182		
751083655	727.371	36.707	-16.7	0.245	0.105		
751083656	686.714	26.027	6.3	0.612	0.269	Tax_Id=9606 Gene_Symbol=CNTN1 Isoform 1 of Contactin-1	KVLEPMPSTAEISTSGAVLK
751083657	687.048	26.025	4.6	0.537	0.399	Tax_Id=9606 Gene_Symbol=CNTN1 Isoform 1 of Contactin-1	KVLEPMPSTAEISTSGAVLK
751083658	687.383	26.021	3.7	0.488	0.416	Tax_Id=9606 Gene_Symbol=CNTN1 Isoform 1 of Contactin-1	KVLEPMPSTAEISTSGAVLK
751083659	687.721	26.005	10.5	0.332	0.288	Tax_Id=9606 Gene_Symbol=CNTN1 Isoform 1 of Contactin-1	KVLEPMPSTAEISTSGAVLK
751083660	459.243	17.482	6.2	0.506	0.223		
751083661	459.745	17.49	2.7	0.817	0.166		
751083662	546.015	26.859	0.4	0.933	0.578		
751083663	546.266	26.887	0.1	0.978	0.518		
751083664	546.516	26.892	1.9	0.735	0.409		
751083665	546.769	26.967	13	0.647	0.591		
751083666	607.112	34.349	16.8	0.424	0.724		
751083667	607.313	34.369	-1	0.863	0.417		
751083668	607.513	34.371	2.9	0.716	0.357		
751083669	607.714	34.364	2.3	0.819	0.566		
751083670	641.276	16.875	6.6	0.571	0.525		
751083671	641.777	16.876	4.5	0.767	0.557		
751083672	468.015	19.879	-0.9	0.828	0.142		
751083673	468.215	19.894	-0.5	0.902	0.12		
751083674	468.416	19.872	-3	0.5	0.081		
751083675	468.617	19.88	-3.7	0.702	0.083		
751083676	468.817	19.873	-9.9	0.678	0.451		
751083677	958.441	25.446	12.1	0.408	0.365	Tax_Id=9606 Gene_Symbol=GSN Isoform 1 of Gelsolin	VPFDAATLHTSTAMAAQHGMDDDGTGQK
751083678	958.775	25.44	5.9	0.504	0.193	Tax_Id=9606 Gene_Symbol=GSN Isoform 1 of Gelsolin	VPFDAATLHTSTAMAAQHGMDDDGTGQK
751083679	959.109	25.443	12	0.65	0.309	Tax_Id=9606 Gene_Symbol=GSN Isoform 1 of Gelsolin	VPFDAATLHTSTAMAAQHGMDDDGTGQK
751083680	634.663	34.152	7.1	0.276	0.886		
751083681	634.997	34.151	7.1	0.305	0.616		
751083682	635.331	34.144	2.8	0.833	0.375		
751083683	792.334	44.726	6.2	0.342	0.266		
751083684	792.46	44.762	6.2	0.372	0.298		
751083685	792.584	44.75	7.6	0.163	0.725		
751083686	792.71	44.755	7.5	0.155	0.896		
751083687	792.835	44.747	7.6	0.174	0.871		
751083688	792.962	44.748	6.9	0.195	0.905		
751083689	1022.507	46.752	-2.2	0.559	0.235		
751083690	1022.709	46.605	-0.9	0.88	0.373		
751083691	1022.907	46.634	0	0.999	0.255		
751083692	522.911	16.408	-18.8	0.511	0.21		
751083693	523.246	16.46	8.5	0.7	0.469		
751083694	523.579	16.383	-0.3	0.985	0.519		
751083695	400.436	16.58	12.1	0.071	0.46		
751083696	400.637	16.578	12.5	0.071	0.334		
751083697	400.838	16.58	16.1	0.29	0.853		
751083698	401.038	16.568	116.8	0.029	0.112		
751083699	744.686	43.078	2.2	0.684	0.627		
751083700	585.294	18.863	6.3	0.285	0.605		
751083701	585.797	18.944	-1.3	0.864	0.671		
751083702	929.752	40.141	-8.5	0.395	0.688		
751083703	930.078	40.087	-6.8	0.223	0.112		
751083704	930.429	40.07	-6.1	0.287	0.111		
751083705	930.753	40.207	-11.9	0.178	0.167		
751083706	533.276	27.366	6.6	0.637	0.469		
751083707	533.611	27.381	1.1	0.937	0.192		
751083708	533.946	27.396	6.9	0.788	0.559		
751083709	530.289	14.979	0.7	0.973	0.312		
751083710	530.789	14.973	-2.5	0.849	0.112		
751083711	631.337	22.346	-1.1	0.837	0.435	Tax_Id=9606 Gene_Symbol=NFASC Isoform 7 of Neurofascin	VIAINEVGSSHPSLPSER

751083712	631.672	22.338	-5.3	0.206	0.056	Tax_Id=9606 Gene_Symbol=NFASC Isoform 7 of Neurofascin	VIAINEVGSSHPSLPSER
751083713	632.006	22.343	-20.1	0.104	0.034	Tax_Id=9606 Gene_Symbol=NFASC Isoform 7 of Neurofascin	VIAINEVGSSHPSLPSER
751083714	739.844	34.859	0.8	0.937	0.188		
751083715	740.095	34.864	1.7	0.837	0.229		
751083716	740.346	34.866	2.1	0.793	0.355		
751083717	740.596	34.865	1.7	0.88	0.149		
751083718	740.847	34.849	-4.2	0.759	0.194		
751083719	527.803	27.317	-12.6	0.441	0.465		
751083720	528.305	27.331	-31.1	0.112	0.086		
751083721	813.91	26.46	6.6	0.285	0.942	Tax_Id=9606 Gene_Symbol=TIMP2 Metalloproteinase inhibitor 2	GAAPPKQEFLDIEDP
751083722	814.408	26.378	3.2	0.473	0.894	Tax_Id=9606 Gene_Symbol=TIMP2 Metalloproteinase inhibitor 2	GAAPPKQEFLDIEDP
751083723	814.911	26.459	-21.6	0.257	0.198	Tax_Id=9606 Gene_Symbol=TIMP2 Metalloproteinase inhibitor 2	GAAPPKQEFLDIEDP
751083724	444.746	17.168	8.3	0.404	0.365		
751083725	445.247	17.172	8.9	0.375	0.276		
751083726	1044.566	39.15	-4.4	0.543	0.034		
751083727	1045.067	39.152	-4.8	0.505	0.03		
751083728	1045.569	39.156	-17.6	0.102	0.01		
751083729	683.991	37.932	2.9	0.587	0.475		
751083730	506.299	16.521	8.1	0.713	0.431		
751083731	934.253	35.144	7	0.515	0.622		
751083732	656.66	23.986	4	0.234	0.721		
751083733	656.986	24.056	2.3	0.497	0.629		
751083734	657.322	24.047	3.4	0.407	0.635		
751083735	620.98	20.077	-32.9	0.017	0.058		
751083736	621.32	20.071	-0.4	0.949	0.452		
751083737	450.246	15.573	5.1	0.259	0.481	Tax_Id=9606 Gene_Symbol=SERPIND1 Heparin cofactor 2	GPLDQLEK
751083738	450.748	15.626	4.5	0.683	0.836	Tax_Id=9606 Gene_Symbol=SERPIND1 Heparin cofactor 2	GPLDQLEK
751083739	609.298	66.233	3.3	0.675	0.384		
751083740	610.301	66.216	-12.5	0.513	0.444		
751083741	755.845	26.096	9.5	0.544	0.688		
751083742	756.346	26.108	10.2	0.247	0.536		
751083743	455.267	25.25	22.4	0.107	0.779		
751083744	455.602	25.234	57.1	0.043	0.445		
751083745	674.339	23.108	8.8	0.655	0.308	Tax_Id=9606 Gene_Symbol=APOA1 Apolipoprotein A-I	ETEGLRQEMSKDLEEVK
751083746	674.672	23.104	22.2	0.26	0.753	Tax_Id=9606 Gene_Symbol=APOA1 Apolipoprotein A-I	ETEGLRQEMSKDLEEVK
751083747	675.006	23.066	11.1	0.386	0.881	Tax_Id=9606 Gene_Symbol=APOA1 Apolipoprotein A-I	ETEGLRQEMSKDLEEVK
751083748	782.364	28.753	6.7	0.543	0.881		
751083749	782.865	28.753	7	0.699	0.852		
751083750	783.365	28.768	-3.8	0.854	0.456		
751083751	822.444	35.303	4.6	0.302	0.862	Tax_Id=9606 Gene_Symbol=PKM2 Isoform M1 of Pyruvate kinase isozymes	TATESFASDPILYRPVAVALDTK
751083752	822.778	35.286	3.1	0.442	0.881	Tax_Id=9606 Gene_Symbol=PKM2 Isoform M1 of Pyruvate kinase isozymes	TATESFASDPILYRPVAVALDTK
751083753	823.111	35.367	3.3	0.47	0.746	Tax_Id=9606 Gene_Symbol=PKM2 Isoform M1 of Pyruvate kinase isozymes	TATESFASDPILYRPVAVALDTK
751083754	823.445	35.329	-17.7	0.199	0.244	Tax_Id=9606 Gene_Symbol=PKM2 Isoform M1 of Pyruvate kinase isozymes	TATESFASDPILYRPVAVALDTK
751083755	492.245	15.516	1.8	0.871	0.427		
751083756	492.746	15.494	-8.6	0.706	0.358		
751083757	968.241	32.021	-0.9	0.901	0.306		
751083758	968.492	32.107	0.2	0.964	0.373		
751083759	968.744	31.984	-11.4	0.251	0.059		
751083760	756.416	17.626	-1.3	0.912	0.119		
751083761	757.423	17.595	-7.8	0.669	0.539		
751083762	727.724	44.305	3	0.74	0.365		
751083763	728.058	44.316	8.6	0.256	0.927		
751083764	420.972	5.647	14.2	0.725	0.879		
751083765	729.877	25.674	-0.5	0.867	0.655		
751083766	759.164	39.376	-2.3	0.898	0.856		
751083767	759.367	39.346	-10.3	0.101	0.233		
751083768	759.568	39.375	-3.9	0.68	0.21		
751083769	759.768	39.388	-3.9	0.691	0.223		
751083770	759.969	39.41	-13.2	0.246	0.058		
751083771	760.169	39.407	-4.5	0.72	0.162		
751083772	512.762	16.791	5.2	0.763	0.302		
751083773	513.265	16.808	-3.1	0.902	0.813		
751083774	730.4	14.263	5.3	0.774	0.46		
751083775	731.404	14.184	14.4	0.388	0.509		
751083776	599.366	20.673	5.6	0.754	0.249		
751083777	599.868	20.657	4.2	0.841	0.402		
751083778	509.767	22.657	3.9	0.843	0.37		
751083779	491.577	15.67	1.7	0.667	0.267	Tax_Id=9606 Gene_Symbol=CSF1 Isoform 1 of Macrophage colony-stimulati	FNSVPLTDTGHER
751083780	491.911	15.648	1.6	0.869	0.312	Tax_Id=9606 Gene_Symbol=CSF1 Isoform 1 of Macrophage colony-stimulati	FNSVPLTDTGHER
751083781	592.752	17.92	5.6	0.293	0.625		
751083782	593.254	17.951	1.7	0.884	0.413		
751083783	1142.522	26.993	1.6	0.776	0.611		
751083784	1142.768	26.815	2.7	0.683	0.586		
751083785	1143.018	26.809	-0.3	0.988	0.526		
751083786	1143.244	26.861	1.2	0.926	0.423		
751083787	1143.525	27.01	-18.2	0.162	0.092		
751083788	671.104	33.701	6.3	0.516	0.836	Tax_Id=9606 Gene_Symbol=CFD Complement factor D preproprotein	AVPHPDSQPDTIDHDLLLLQLSEK
751083789	671.355	33.792	0.7	0.929	0.976	Tax_Id=9606 Gene_Symbol=CFD Complement factor D preproprotein	AVPHPDSQPDTIDHDLLLLQLSEK
751083790	671.606	33.697	4.8	0.584	0.765	Tax_Id=9606 Gene_Symbol=CFD Complement factor D preproprotein	AVPHPDSQPDTIDHDLLLLQLSEK
751083791	671.856	33.617	-11.4	0.119	0.238	Tax_Id=9606 Gene_Symbol=CFD Complement factor D preproprotein	AVPHPDSQPDTIDHDLLLLQLSEK
751083792	991.56	47.817	3	0.606	0.226		
751083793	992.061	47.803	1.4	0.814	0.182		
751083794	992.563	47.828	4.1	0.526	0.147		
751083795	899.458	47.889	7	0.188	0.995		
751083796	899.709	47.887	7.8	0.193	0.857		
751083797	899.96	47.891	6.7	0.231	0.732		
751083798	900.21	47.892	4.7	0.442	0.623		
751083799	900.46	47.855	6.5	0.233	0.859		
751083800	780.697	47.344	12.7	0.328	0.613		
751083801	780.841	47.357	3.6	0.589	0.559		
751083802	780.985	47.366	3.1	0.612	0.526		
751083803	781.128	47.366	3.7	0.557	0.582		
751083804	781.271	47.368	5.2	0.278	0.682		

751083805	781.414	47.378	3	0.447	0.544		
751083806	781.557	47.37	3.6	0.559	0.441		
751083807	781.701	47.241	-1	0.818	0.253		
751083808	607.808	22.538	5.5	0.509	0.628		
751083809	608.309	22.547	8.7	0.379	0.452		
751083810	1201.484	22.917	-4	0.595	0.074	Tax_Id=9606 Gene_Symbol=SPARCL1 cDNA FLJ52396, highly similar to SF	MQEDEFDQGNQEQEDNSNAEMEEENASNVNK
751083811	1201.817	22.959	-0.6	0.921	0.121	Tax_Id=9606 Gene_Symbol=SPARCL1 cDNA FLJ52396, highly similar to SF	MQEDEFDQGNQEQEDNSNAEMEEENASNVNK
751083812	1202.152	22.966	-1.6	0.81	0.088	Tax_Id=9606 Gene_Symbol=SPARCL1 cDNA FLJ52396, highly similar to SF	MQEDEFDQGNQEQEDNSNAEMEEENASNVNK
751083813	1202.488	22.961	1.5	0.893	0.748	Tax_Id=9606 Gene_Symbol=SPARCL1 cDNA FLJ52396, highly similar to SF	MQEDEFDQGNQEQEDNSNAEMEEENASNVNK
751083814	1202.823	22.937	-1.9	0.829	0.113	Tax_Id=9606 Gene_Symbol=SPARCL1 cDNA FLJ52396, highly similar to SF	MQEDEFDQGNQEQEDNSNAEMEEENASNVNK
751083815	528.293	16.357	7.7	0.225	0.897		
751083816	528.793	16.345	6.6	0.481	0.683		
751083817	675.963	19.773	9.6	0.232	0.637		
751083818	676.297	19.774	5.2	0.59	0.182		
751083819	676.632	19.789	5.3	0.712	0.175		
751083820	968.426	21.43	3.1	0.837	0.488		
751083821	968.761	21.434	3.8	0.823	0.402		
751083822	969.096	21.435	2.2	0.898	0.426		
751083823	969.43	21.437	21	0.446	0.732		
751083824	946.478	17.491	-13.8	0.219	0.039		
751083825	947.482	17.489	-17.9	0.386	0.233		
751083826	713.851	42.945	4.4	0.402	0.656	Tax_Id=9606 Gene_Symbol=CP Ceruloplasmin	WYLFGMGNEVDVHAAFFHGQALTNK
751083827	714.102	43.088	4.7	0.394	0.63	Tax_Id=9606 Gene_Symbol=CP Ceruloplasmin	WYLFGMGNEVDVHAAFFHGQALTNK
751083828	714.352	42.966	4.4	0.4	0.402	Tax_Id=9606 Gene_Symbol=CP Ceruloplasmin	WYLFGMGNEVDVHAAFFHGQALTNK
751083829	714.603	42.937	4.8	0.459	0.645	Tax_Id=9606 Gene_Symbol=CP Ceruloplasmin	WYLFGMGNEVDVHAAFFHGQALTNK
751083830	714.855	42.864	5.7	0.666	0.971	Tax_Id=9606 Gene_Symbol=CP Ceruloplasmin	WYLFGMGNEVDVHAAFFHGQALTNK
751083831	507.742	20.488	-4.2	0.49	0.43		
751083832	1015.488	20.432	0.4	0.977	0.306		
751083833	1016.49	20.465	-33.5	0.034	0.009		
751083834	943.028	30.719	1.9	0.753	0.615		
751083835	944.038	30.828	-7.9	0.246	0.055		
751083836	674.39	17.194	23.1	0.247	0.9		
751083837	675.394	17.185	29.8	0.246	0.786		
751083838	507.502	24.016	4.2	0.493	0.643		
751083839	507.752	24.055	4.6	0.349	0.767		
751083840	508.003	24.008	-11.1	0.517	0.553		
751083841	508.255	23.993	-13.7	0.295	0.059		
751083842	1169.633	29.716	2.7	0.773	0.285		
751083843	1170.636	29.739	7.7	0.566	0.439		
751083844	682.789	22.011	-8.6	0.213	0.058		
751083845	683.291	22.001	0.8	0.907	0.368		
751083846	675.67	28.674	-6.8	0.091	0.028		
751083847	676.004	28.642	-6.4	0.116	0.013		
751083848	943.536	30.856	-4.8	0.406	0.184		
751083849	1081.955	24.989	-22.4	0	0	Tax_Id=9606 Gene_Symbol=VGF Neurosecretory protein VGF	VGEEDEEAAEAEAEAEEAER
751083850	1082.458	25.007	-22.2	0	0.001	Tax_Id=9606 Gene_Symbol=VGF Neurosecretory protein VGF	VGEEDEEAAEAEAEAEEAER
751083851	1082.959	24.992	-32	0	0.002	Tax_Id=9606 Gene_Symbol=VGF Neurosecretory protein VGF	VGEEDEEAAEAEAEAEEAER
751083852	1083.464	24.972	-36.7	0.007	0.038	Tax_Id=9606 Gene_Symbol=VGF Neurosecretory protein VGF	VGEEDEEAAEAEAEAEEAER
751083853	398.556	15.792	0.1	0.991	0.417	Tax_Id=9606 Gene_Symbol=SCG3 Secretogranin-3	TEAYLEAIRK
751083854	398.891	15.798	12.8	0.355	0.877	Tax_Id=9606 Gene_Symbol=SCG3 Secretogranin-3	TEAYLEAIRK
751083855	980.517	39.911	1.2	0.858	0.865		
751083856	981.017	39.88	2	0.769	0.913		
751083857	608.631	23.128	3.8	0.521	0.877	Tax_Id=9606 Gene_Symbol=CTSB Cathepsin B	GQDHC[160.0302]GIESEVVAGIPR
751083858	608.965	23.128	2.5	0.583	0.464	Tax_Id=9606 Gene_Symbol=CTSB Cathepsin B	GQDHC[160.0302]GIESEVVAGIPR
751083859	609.301	23.114	7.1	0.522	0.481	Tax_Id=9606 Gene_Symbol=CTSB Cathepsin B	GQDHC[160.0302]GIESEVVAGIPR
751083860	499.284	29.054	7.5	0.6	0.503		
751083861	499.788	29.136	-4.4	0.726	0.212		
751083862	400.225	22.101	13.4	0.039	0.935		
751083863	400.56	22.032	165.5	0.024	0.153		
751083864	758.341	16.538	-0.3	0.983	0.682		
751083865	758.843	16.537	25.4	0.196	0.3		
751083866	759.35	16.576	-23.6	0.371	0.069		
751083867	881.465	38.76	0.5	0.925	0.486		
751083868	1080.983	27.989	-2.1	0.853	0.198		
751083869	1081.234	27.991	-8.6	0.281	0.12		
751083870	1081.485	28.008	-4.8	0.587	0.145		
751083871	1081.735	28.004	-5.3	0.513	0.063		
751083872	1081.988	28	-11	0.381	0.16		
751083873	578.357	18.27	-6.2	0.37	0.031		
751083874	921.703	37.274	4.4	0.655	0.637	Tax_Id=9606 Gene_Symbol=ITIH2 Inter-alpha (Globulin) inhibitor H2, isoform	NVQFNYPHTSVTDVTQNNFHNYFGGSEIVVAGK
751083875	921.953	37.264	11.6	0.122	0.518	Tax_Id=9606 Gene_Symbol=ITIH2 Inter-alpha (Globulin) inhibitor H2, isoform	NVQFNYPHTSVTDVTQNNFHNYFGGSEIVVAGK
751083876	922.204	37.287	6.5	0.295	0.875	Tax_Id=9606 Gene_Symbol=ITIH2 Inter-alpha (Globulin) inhibitor H2, isoform	NVQFNYPHTSVTDVTQNNFHNYFGGSEIVVAGK
751083877	922.453	37.286	1.9	0.665	0.976	Tax_Id=9606 Gene_Symbol=ITIH2 Inter-alpha (Globulin) inhibitor H2, isoform	NVQFNYPHTSVTDVTQNNFHNYFGGSEIVVAGK
751083878	922.706	37.277	-1	0.905	0.439	Tax_Id=9606 Gene_Symbol=ITIH2 Inter-alpha (Globulin) inhibitor H2, isoform	NVQFNYPHTSVTDVTQNNFHNYFGGSEIVVAGK
751083879	584.948	31.455	-18.2	0.376	0.178		
751083880	585.115	31.462	-0.3	0.987	0.192		
751083881	585.283	31.484	3.4	0.818	0.487		
751083882	585.448	31.348	-5.8	0.625	0.076		
751083883	585.616	31.461	-27.7	0.277	0.022		
751083884	736.016	43.602	-6.4	0.294	0.15		
751083885	736.353	43.637	-4.1	0.518	0.319		
751083886	736.684	43.591	-6	0.366	0.206		
751083887	737.018	43.571	-24	0.083	0.079		
751083888	1040.005	27.352	-2.6	0.818	0.724		
751083889	1040.506	27.355	-31.1	0.033	0.091		
751083890	1040.997	27.23	-13	0.339	0.399		
751083891	741.373	29.327	7.4	0.207	0.534		
751083892	741.871	29.237	12.7	0.358	0.457		
751083893	680.03	35.412	-16.3	0.264	0.642		
751083894	680.364	35.422	-12.8	0.059	0.067		
751083895	680.699	35.435	-9.6	0.648	0.261		
751083896	605.828	26.958	-12.5	0.276	0.085	Tax_Id=9606 Gene_Symbol=AGRN Agrin	HPLHLLEDAVTKPELRPC[160.0302]PTP
751083897	606.078	26.953	-0.5	0.928	0.247	Tax_Id=9606 Gene_Symbol=AGRN Agrin	HPLHLLEDAVTKPELRPC[160.0302]PTP

751083898	606.329	26.966	-0.6	0.961	0.314	Tax_Id=9606 Gene_Symbol=AGRN Agrin	HPLHLLEDAVTKPELRPC[160.0302]PTP
751083899	606.58	26.973	-14.7	0.397	0.18	Tax_Id=9606 Gene_Symbol=AGRN Agrin	HPLHLLEDAVTKPELRPC[160.0302]PTP
751083900	576.808	23.395	14.6	0.187	0.834	Tax_Id=9606 Gene_Symbol=C3 Complement C3 (Fragment)	QPSSAFAAFVK
751083901	577.309	23.395	13.1	0.482	0.543	Tax_Id=9606 Gene_Symbol=C3 Complement C3 (Fragment)	QPSSAFAAFVK
751083902	861.151	23.753	-3.7	0.729	0.627		
751083903	861.653	23.768	-3.2	0.775	0.901		
751083904	702.158	43.028	-5.8	0.688	0.292		
751083905	702.358	43.074	3	0.624	0.371		
751083906	702.559	43.05	3	0.825	0.44		
751083907	702.759	43.084	-0.7	0.918	0.296		
751083908	702.959	43.057	-6.6	0.611	0.374		
751083909	964.462	22.327	2.1	0.79	0.504	Tax_Id=9606 Gene_Symbol=C1QC Complement C1q subcomponent subunit	FNAVLTNPQGDYDTSTGK
751083910	964.964	22.348	-0.8	0.904	0.328	Tax_Id=9606 Gene_Symbol=C1QC Complement C1q subcomponent subunit	FNAVLTNPQGDYDTSTGK
751083911	965.466	22.35	-7.6	0.522	0.378	Tax_Id=9606 Gene_Symbol=C1QC Complement C1q subcomponent subunit	FNAVLTNPQGDYDTSTGK
751083912	669.667	31.47	-16	0.017	0.032		
751083913	670.001	31.468	-15.1	0.028	0.026		
751083914	670.334	31.452	-19.9	0.026	0.073		
751083915	1232.264	29.4	-20.6	0.077	0.096		
751083916	1232.598	29.396	4.5	0.66	0.887		
751083917	1232.932	29.396	-8	0.486	0.273		
751083918	1233.266	29.399	-21.5	0.125	0.353		
751083919	414.728	21.929	7	0.43	0.892		
751083920	414.895	21.926	8.2	0.37	0.859		
751083921	415.062	21.923	3.7	0.744	0.435		
751083922	686.346	27.645	2.8	0.652	0.967		
751083923	686.854	27.66	4.1	0.476	0.906		
751083924	523.783	21.929	8.2	0.35	0.285		
751083925	595.708	39.496	48.4	0.118	0.996	Tax_Id=9606 Gene_Symbol=CHGB Secretogranin-1	ADQTVLTEDEKKELENLAAMDLELQK
751083926	595.908	39.49	6.7	0.77	0.216	Tax_Id=9606 Gene_Symbol=CHGB Secretogranin-1	ADQTVLTEDEKKELENLAAMDLELQK
751083927	596.109	39.493	10.7	0.618	0.212	Tax_Id=9606 Gene_Symbol=CHGB Secretogranin-1	ADQTVLTEDEKKELENLAAMDLELQK
751083928	596.311	39.489	3.9	0.798	0.195	Tax_Id=9606 Gene_Symbol=CHGB Secretogranin-1	ADQTVLTEDEKKELENLAAMDLELQK
751083929	408.248	15.259	1.4	0.886	0.165		
751083930	408.75	15.262	-8.5	0.639	0.074		
751083931	720.368	29.87	-0.7	0.915	0.265		
751083932	721.34	29.895	2	0.775	0.125		
751083933	536.301	25.403	7.1	0.488	0.576	Tax_Id=9606 Gene_Symbol=CNTN1 Isoform 1 of Contactin-1	TTKPYPADIVVQFK
751083934	536.636	25.389	6.6	0.516	0.534	Tax_Id=9606 Gene_Symbol=CNTN1 Isoform 1 of Contactin-1	TTKPYPADIVVQFK
751083935	536.97	25.397	16.6	0.535	0.892	Tax_Id=9606 Gene_Symbol=CNTN1 Isoform 1 of Contactin-1	TTKPYPADIVVQFK
751083936	719.039	38.394	-5.7	0.635	0.048		
751083937	719.376	38.388	0.1	0.986	0.153		
751083938	719.708	38.385	2.1	0.825	0.185		
751083939	720.043	38.464	-25.6	0.109	0.019		
751083940	487.725	16.97	4.7	0.797	0.422		
751083941	488.227	16.99	-0.3	0.983	0.249		
751083942	672.957	19.907	2.5	0.795	0.153		
751083943	673.291	19.882	-3.7	0.665	0.229		
751083944	673.628	19.895	-3.3	0.771	0.193		
751083945	463.906	15.94	4.5	0.83	0.443		
751083946	783.442	31.736	10.3	0.019	0.864		
751083947	1103.585	24.401	4.1	0.797	0.169		
751083948	1104.587	24.401	-3.8	0.772	0.074		
751083949	727.85	28.712	7.7	0.335	0.656		
751083950	728.352	28.745	7	0.21	0.356		
751083951	737.949	42.184	5.3	0.593	0.758		
751083952	738.45	42.175	4.7	0.593	0.584		
751083953	620.317	33.708	-5.7	0.34	0.481		
751083954	620.567	33.693	-4.2	0.476	0.77		
751083955	620.817	33.676	-6.4	0.245	0.372		
751083956	621.07	33.705	-21.9	0.188	0.177		
751083957	596.771	21.095	2.1	0.831	0.181		
751083958	597.284	21.207	0	0.999	0.769		
751083959	820.374	29.198	-0.6	0.976	0.35	Tax_Id=9606 Gene_Symbol=PIK3IP1 Isoform 1 of Phosphoinositide-3-kinase	NPDEDPRGPWC[160.0302]YVSGEAGVPEK
751083960	820.71	29.289	-2.2	0.809	0.107	Tax_Id=9606 Gene_Symbol=PIK3IP1 Isoform 1 of Phosphoinositide-3-kinase	NPDEDPRGPWC[160.0302]YVSGEAGVPEK
751083961	821.044	29.3	-2.3	0.811	0.15	Tax_Id=9606 Gene_Symbol=PIK3IP1 Isoform 1 of Phosphoinositide-3-kinase	NPDEDPRGPWC[160.0302]YVSGEAGVPEK
751083962	490.586	21.791	-9.6	0.233	0.097		
751083963	490.92	21.784	-0.3	0.976	0.477		
751083964	491.254	21.764	-0.1	0.993	0.321		
751083965	436.519	15.404	-52.3	0.071	0.108		
751083966	436.853	15.406	6.6	0.755	0.396		
751083967	636.385	20.622	-0.3	0.945	0.045		
751083968	636.887	20.616	-0.6	0.956	0.145		
751083969	780.926	27.345	1.6	0.742	0.305	Tax_Id=9606 Gene_Symbol=PEBP1 Phosphatidylethanolamine-binding prote	LYTLVLTDPDAPSR
751083970	781.423	27.352	1.5	0.916	0.984	Tax_Id=9606 Gene_Symbol=PEBP1 Phosphatidylethanolamine-binding prote	LYTLVLTDPDAPSR
751083971	781.926	27.356	-13.1	0.573	0.751	Tax_Id=9606 Gene_Symbol=PEBP1 Phosphatidylethanolamine-binding prote	LYTLVLTDPDAPSR
751083972	847.639	22.039	4.9	0.85	0.959		
751083973	847.889	22.036	-4.5	0.716	0.681		
751083974	848.139	22.042	-7.3	0.662	0.418		
751083975	848.393	22.034	-12.8	0.353	0.411		
751083976	633.967	44.77	8.7	0.193	0.65		
751083977	588.838	28.27	11.8	0.52	0.582		
751083978	589.34	28.269	-0.5	0.981	0.151		
751083979	828.693	41.546	6.5	0.621	0.411		
751083980	828.943	41.539	3.2	0.475	0.135		
751083981	829.194	41.542	2	0.884	0.132		
751083982	655.344	31.216	8.5	0.251	0.899		
751083983	655.677	31.217	3.8	0.423	0.681		
751083984	656.007	31.222	2.7	0.723	0.527		
751083985	615.17	35.625	30.9	0.173	0.455		
751083986	615.313	35.659	11.8	0.29	0.617		
751083987	615.456	35.627	21.2	0.113	0.873		
751083988	615.6	35.631	20.9	0.274	0.515		
751083989	615.743	35.629	21.7	0.314	0.969		
751083990	841.412	38.205	5.9	0.623	0.252		

751083991	841.745	38.201	4.9	0.432	0.262		
751083992	1008.934	19.921	-25	0.303	0.423		
751083993	1009.435	19.924	-2.9	0.868	0.401		
751083994	1009.936	19.908	-11.4	0.408	0.215		
751083995	662.523	34.58	8.2	0.427	0.575		
751083996	473.99	26.855	5.1	0.563	0.653		
751083997	474.241	26.857	7.2	0.715	0.461		
751083998	474.492	26.86	6.1	0.682	0.328		
751083999	474.743	26.852	8.4	0.763	0.348		
751084000	648.654	32.194	0.9	0.826	0.406		
751084001	932.653	40.747	4.5	0.678	0.457		
751084002	932.853	40.762	2.3	0.713	0.325		
751084003	933.054	40.809	1.8	0.787	0.265		
751084004	933.253	40.835	0.5	0.916	0.072		
751084005	933.454	40.876	-0.6	0.895	0.098		
751084006	933.653	40.901	-0.5	0.952	0.156		
751084007	933.856	40.89	-8	0.638	0.528		
751084008	829.895	21.447	0.7	0.858	0.212		
751084009	830.396	21.481	-4.9	0.243	0.014		
751084010	754.357	29.299	-1.3	0.714	0.087		
751084011	754.856	29.122	-1.2	0.841	0.094		
751084012	577.814	17.193	-3.4	0.708	0.175		
751084013	578.318	17.181	-16.4	0.424	0.475		
751084014	550.519	18.526	-14.4	0.01	0		
751084015	550.766	18.453	-7.5	0.127	0.01		
751084016	551.017	18.445	-55.9	0	0.001		
751084017	527.898	15.683	8.5	0.37	0.309		
751084018	528.232	15.683	9.1	0.416	0.318		
751084019	528.566	15.685	13	0.633	0.737		
751084020	668.786	22.86	3.4	0.643	0.475		
751084021	669.288	22.769	5.5	0.408	0.237		
751084022	486.619	28.43	-0.7	0.902	0.188		
751084023	486.953	28.429	-0.9	0.954	0.148		
751084024	371.101	74.432	2.1	0.804	0.29		
751084025	372.101	74.428	2.3	0.774	0.287		
751084026	1117.254	34.201	-28.5	0.002	0.002		
751084027	1117.505	34.204	-10.7	0.199	0.431		
751084028	1117.756	34.229	-18	0.092	0.424		
751084029	1118.006	34.217	-17	0.125	0.493		
751084030	1118.258	34.207	-22.1	0.178	0.379		
751084031	975.43	20.917	10.8	0.242	0.766		
751084032	975.931	20.917	5.5	0.39	0.512		
751084033	976.435	20.923	3.5	0.68	0.558		
751084034	638.339	23.218	6.3	0.336	0.841	Tax_Id=9606 Gene_Symbol=CFB Isoform 1 of Complement factor B (Fragme	YGLVTYATYPK
751084035	638.84	23.239	1.9	0.935	0.579	Tax_Id=9606 Gene_Symbol=CFB Isoform 1 of Complement factor B (Fragme	YGLVTYATYPK
751084036	864.092	42.435	-7.5	0.168	0.118		
751084037	864.427	42.439	-5	0.367	0.236		
751084038	864.76	42.436	-5.8	0.385	0.205		
751084039	865.095	42.465	-13.3	0.325	0.299		
751084040	606.353	23.546	-3.2	0.679	0.077		
751084041	983.463	29.698	0.9	0.83	0.3		
751084042	983.965	29.802	-0.7	0.855	0.099		
751084043	775.334	21.545	4	0.634	0.636	Tax_Id=9606 Gene_Symbol=EFEMP1 Isoform 4 of EGF-containing fibulin-lik	DIDEC[160.0302]DIVPDAC[160.0302]K
751084044	775.835	21.543	2.4	0.72	0.485	Tax_Id=9606 Gene_Symbol=EFEMP1 Isoform 4 of EGF-containing fibulin-lik	DIDEC[160.0302]DIVPDAC[160.0302]K
751084045	776.334	21.575	5.5	0.652	0.609	Tax_Id=9606 Gene_Symbol=EFEMP1 Isoform 4 of EGF-containing fibulin-lik	DIDEC[160.0302]DIVPDAC[160.0302]K
751084046	579.795	16.846	7.5	0.339	0.838		
751084047	580.299	16.773	5.9	0.485	0.774		
751084048	493.271	23.515	6.3	0.753	0.387	Tax_Id=9606 Gene_Symbol=FN1 263 kDa protein	HRPRPYPPNVGEEIQIGHIPR
751084049	493.471	23.538	2.4	0.696	0.38	Tax_Id=9606 Gene_Symbol=FN1 263 kDa protein	HRPRPYPPNVGEEIQIGHIPR
751084050	493.672	23.505	4.9	0.768	0.27	Tax_Id=9606 Gene_Symbol=FN1 263 kDa protein	HRPRPYPPNVGEEIQIGHIPR
751084051	493.873	23.498	-7.3	0.644	0.031	Tax_Id=9606 Gene_Symbol=FN1 263 kDa protein	HRPRPYPPNVGEEIQIGHIPR
751084052	943.427	37.453	-0.3	0.976	0.28		
751084053	943.76	37.45	3.9	0.742	0.783		
751084054	944.094	37.456	-1.2	0.917	0.198		
751084055	944.427	37.464	-14.5	0.213	0.054		
751084056	595.292	18.968	3.5	0.533	0.606		
751084057	968.448	24.518	2.9	0.495	0.672		
751084058	944.511	20.548	-3.7	0.73	0.108		
751084059	945.511	20.606	-8.9	0.588	0.382		
751084060	540.917	15.844	-2.3	0.882	0.248		
751084061	541.246	15.78	-18.3	0.106	0.087		
751084062	541.586	15.865	-13.9	0.524	0.105		
751084063	702.531	39.134	9	0.321	0.374		
751084064	702.73	39.284	7.1	0.171	0.967		
751084065	702.931	39.262	6.2	0.246	0.69		
751084066	703.132	39.295	6.5	0.1	0.912		
751084067	703.332	39.101	-0.5	0.93	0.347		
751084068	589.247	15.563	2.4	0.889	0.164		
751084069	589.749	15.576	-6.7	0.751	0.19		
751084070	553.325	34.603	9.1	0.546	0.449		
751084071	553.659	34.6	3.9	0.804	0.795		
751084072	553.995	34.598	2.9	0.816	0.351		
751084073	669.011	34.237	7.2	0.498	0.279		
751084074	669.345	34.228	2.9	0.668	0.281		
751084075	669.68	34.235	8.5	0.538	0.204		
751084076	922.461	26.255	5	0.383	0.864		
751084077	922.963	26.254	3.1	0.605	0.225		
751084078	923.458	26.26	-3.4	0.571	0.104		
751084079	829.4	22.486	2.3	0.76	0.524		
751084080	829.902	22.548	3.5	0.733	0.706		
751084081	830.402	22.568	-8.1	0.388	0.109		
751084082	647.333	21.234	6.4	0.647	0.697		
751084083	647.834	21.23	12.3	0.39	0.795		

751084084	892.684	45.431	4.7	0.581	0.83		
751084085	892.934	45.551	4.2	0.549	0.83		
751084086	893.187	45.197	6.4	0.553	0.73		
751084087	670.335	25.473	4.6	0.348	0.458	Tax_Id=9606 Gene_Symbol=C4A;C4B complement component 4B preproprc	GSFEFPVGDAVSK
751084088	670.833	25.486	4.2	0.749	0.941	Tax_Id=9606 Gene_Symbol=C4A;C4B complement component 4B preproprc	GSFEFPVGDAVSK
751084089	865.474	30.425	6.6	0.333	0.687		
751084090	866.478	30.426	3.8	0.89	0.538		
751084091	634.815	20.746	0.5	0.94	0.226		
751084092	635.314	20.753	13.5	0.333	0.508		
751084093	989.878	46.527	3.3	0.834	0.811		
751084094	990.222	46.652	5.6	0.411	0.888		
751084095	990.544	46.555	3.3	0.586	0.855		
751084096	990.881	46.546	0.5	0.941	0.25		
751084097	612.766	19.631	-2.3	0.858	0.073		
751084098	613.269	19.639	-11.3	0.036	0.025		
751084099	639.023	38.583	-0.2	0.967	0.338		
751084100	639.356	38.581	0.2	0.975	0.342		
751084101	639.691	38.576	3.1	0.802	0.501		
751084102	640.028	38.637	-21.7	0.285	0.086		
751084103	793.011	23.012	1.4	0.899	0.76		
751084104	793.346	22.983	9.4	0.174	0.383		
751084105	793.678	23.027	2.9	0.76	0.567		
751084106	794.014	23.011	22.2	0.262	0.543		
751084107	508.042	18.23	-33.3	0.093	0.285		
751084108	508.242	18.229	-0.5	0.934	0.297		
751084109	508.443	18.232	-26.7	0.073	0.093		
751084110	508.643	18.219	-30.2	0.327	0.236		
751084111	758.384	27.688	-1.9	0.518	0.056	Tax_Id=9606 Gene_Symbol=ALDOC Fructose-bisphosphate aldolase	GVVPLAGTDGETTTQGLDGLSER
751084112	758.719	27.737	-2.8	0.423	0.176	Tax_Id=9606 Gene_Symbol=ALDOC Fructose-bisphosphate aldolase	GVVPLAGTDGETTTQGLDGLSER
751084113	759.054	27.712	-2.4	0.655	0.13	Tax_Id=9606 Gene_Symbol=ALDOC Fructose-bisphosphate aldolase	GVVPLAGTDGETTTQGLDGLSER
751084114	920.748	31.109	-2.3	0.699	0.19		
751084115	921.082	31.102	-2.9	0.577	0.071		
751084116	921.415	31.03	-1.1	0.867	0.15		
751084117	921.75	31.056	3.9	0.799	0.268		
751084118	568.456	28.408	-42.3	0.143	0.052		
751084119	568.657	28.41	6.3	0.622	0.263		
751084120	568.857	28.403	1	0.919	0.409		
751084121	569.059	28.388	-15.9	0.378	0.445		
751084122	619.635	18.811	2.1	0.754	0.432		
751084123	679.139	34.433	1.4	0.886	0.313		
751084124	679.336	34.439	6.7	0.489	0.448		
751084125	679.54	34.443	5.9	0.648	0.295		
751084126	679.74	34.468	7.2	0.517	0.597		
751084127	679.941	34.445	-7.2	0.642	0.286		
751084128	456.265	21.177	5.3	0.589	0.362		
751084129	456.767	21.195	15.4	0.397	0.555		
751084130	979.485	32.056	2.7	0.608	0.65		
751084131	979.986	32.081	3.6	0.549	0.854		
751084132	705.321	27.517	2.1	0.718	0.523		
751084133	705.522	27.52	2.1	0.709	0.642		
751084134	705.722	27.516	1.9	0.713	0.475		
751084135	705.924	27.538	7.9	0.57	0.707		
751084136	706.124	27.516	4	0.772	0.584		
751084137	738.112	42.393	-6.5	0.368	0.329		
751084138	738.362	42.295	-12.4	0.204	0.273		
751084139	738.612	42.286	-29.7	0.059	0.167		
751084140	738.863	42.261	-5.5	0.65	0.936		
751084141	903.421	16.202	-13.5	0.39	0.064		
751084142	904.427	16.21	-54.6	0.017	0.048		
751084143	899.527	25.033	-2	0.865	0.037		
751084144	900.531	25.045	-18.9	0.368	0.06		
751084145	644.679	28.423	-0.6	0.885	0.288		
751084146	645.013	28.404	0.4	0.933	0.375		
751084147	645.351	28.364	-2.9	0.458	0.107		
751084148	960.331	42.895	16.5	0.167	0.68		
751084149	960.478	42.913	9.4	0.499	0.726		
751084150	960.618	42.913	3.2	0.636	0.85		
751084151	960.761	42.943	1.1	0.871	0.578		
751084152	960.904	42.929	2.7	0.716	0.807		
751084153	961.048	42.928	0.6	0.94	0.737		
751084154	961.192	42.886	1.8	0.807	0.789		
751084155	725.039	34.87	7.4	0.595	0.609	Tax_Id=9606 Gene_Symbol=C3 Complement C3 (Fragment)	ILLQGTPVAQM[147.0355]TEDAVDAER
751084156	725.379	34.978	3	0.658	0.425	Tax_Id=9606 Gene_Symbol=C3 Complement C3 (Fragment)	ILLQGTPVAQM[147.0355]TEDAVDAER
751084157	725.706	34.829	8	0.532	0.753	Tax_Id=9606 Gene_Symbol=C3 Complement C3 (Fragment)	ILLQGTPVAQM[147.0355]TEDAVDAER
751084158	705.358	42.119	10	0.022	0.518		
751084159	705.692	42.138	11	0.015	0.455		
751084160	706.026	42.104	8.7	0.058	0.722		
751084161	744.337	19.921	-3.6	0.864	0.991		
751084162	744.587	19.933	4.5	0.719	0.804		
751084163	744.837	19.935	-5.5	0.764	0.479		
751084164	745.089	19.94	-20.6	0.214	0.676		
751084165	662.82	21.771	-6.8	0.232	0.07		
751084166	663.322	21.77	-30.4	0.003	0.003		
751084167	698.31	22.881	15.6	0.04	0.283		
751084168	698.811	22.913	11.5	0.115	0.519		
751084169	1152.149	20.327	-9	0.651	0.073		
751084170	1152.483	20.326	6.8	0.748	0.229		
751084171	1152.817	20.326	10.3	0.517	0.392		
751084172	1153.152	20.331	9.1	0.562	0.572		
751084173	1153.487	20.335	-9.2	0.719	0.376		
751084174	1055.583	24.407	-0.1	0.994	0.061		
751084175	1056.586	24.425	-43.2	0.016	0.003		
751084176	955.452	23.337	9.1	0.504	0.426	Tax_Id=9606 Gene_Symbol=C4A;C4B complement component 4B preproprc	LLATLC[160.0302]SAEVC[160.0302]QC[160.0302

751084177	955.945	23.288	8.5	0.292	0.795	Tax_Id=9606 Gene_Symbol=C4A;C4B complement component 4B preproprc	LLATLC[160.0302]SAEVC[160.0302]QC[160.0302
751084178	956.443	23.253	10.7	0.595	0.588	Tax_Id=9606 Gene_Symbol=C4A;C4B complement component 4B preproprc	LLATLC[160.0302]SAEVC[160.0302]QC[160.0302
751084179	527.585	22.389	6	0.35	0.821	Tax_Id=9606 Gene_Symbol=EFEMP1 Isoform 4 of EGF-containing fibulin-lik	C[160.0302]VNHYGGYLC[160.0302]LPK
751084180	527.919	22.392	-0.1	0.988	0.634	Tax_Id=9606 Gene_Symbol=EFEMP1 Isoform 4 of EGF-containing fibulin-lik	C[160.0302]VNHYGGYLC[160.0302]LPK
751084181	528.254	22.435	2.8	0.8	0.281	Tax_Id=9606 Gene_Symbol=EFEMP1 Isoform 4 of EGF-containing fibulin-lik	C[160.0302]VNHYGGYLC[160.0302]LPK
751084182	458.839	27.487	12.7	0.181	0.676		
751084183	459.04	27.488	17.1	0.04	0.536		
751084184	459.24	27.497	79.5	0.1	0.518		
751084185	459.441	27.496	41.8	0.136	0.807		
751084186	809.384	20.093	-3.9	0.519	0.558		
751084187	809.707	20.034	-3.6	0.575	0.391		
751084188	810.042	20.117	-2.3	0.71	0.139		
751084189	684.685	41.679	5.1	0.473	0.533		
751084190	684.852	41.615	3.4	0.611	0.312		
751084191	685.033	41.571	8.6	0.24	0.965		
751084192	685.182	41.547	6.3	0.332	0.942		
751084193	686.413	30.105	3	0.886	0.441		
751084194	975.711	44.526	-9.7	0.222	0.057		
751084195	975.95	44.572	2.7	0.821	0.258		
751084196	976.201	44.571	2.9	0.693	0.574		
751084197	976.451	44.562	4.7	0.496	0.954		
751084198	976.703	44.572	4	0.617	0.462		
751084199	976.952	44.577	-6.1	0.762	0.816		
751084200	716.943	25.666	-5.1	0.748	0.368		
751084201	717.143	25.663	3.5	0.69	0.156		
751084202	717.344	25.661	4.2	0.648	0.243		
751084203	717.544	25.667	1.5	0.915	0.222		
751084204	879.654	36.221	-4	0.346	0.159		
751084205	879.905	36.214	0.4	0.924	0.463		
751084206	880.15	36.253	1.2	0.802	0.673		
751084207	880.41	36.311	-1.3	0.804	0.235		
751084208	880.66	36.209	-20.6	0.178	0.569		
751084209	561.28	27.771	2.3	0.678	0.683		
751084210	561.781	27.785	2.4	0.859	0.574		
751084211	696.858	28.749	5.9	0.49	0.168		
751084212	697.359	28.762	9.9	0.453	0.513		
751084213	505.244	16.614	11.6	0.445	0.512		
751084214	752.412	37.967	-0.9	0.891	0.225		
751084215	752.662	37.955	-1.4	0.783	0.231		
751084216	752.912	37.956	0.2	0.975	0.225		
751084217	753.163	37.961	-0.9	0.931	0.181		
751084218	680.026	29.661	-4.2	0.754	0.942		
751084219	680.344	29.752	-0.5	0.93	0.169		
751084220	817.131	66.747	-0.4	0.956	0.981		
751084221	817.305	66.722	-0.9	0.918	0.972		
751084222	817.423	66.741	-0.9	0.907	0.932		
751084223	1209.864	18.052	-25.5	0.136	0.004		
751084224	1210.199	18.046	-15.1	0.374	0.062		
751084225	1210.534	18.05	-19.4	0.211	0.031		
751084226	1210.868	18.053	-14.1	0.412	0.027		
751084227	567.233	16.179	2.3	0.888	0.342		
751084228	567.734	16.18	-5.6	0.63	0.236		
751084229	542.948	23.145	11.8	0.396	0.307		
751084230	543.283	23.122	9.5	0.462	0.328		
751084231	543.617	23.138	20	0.412	0.66		
751084232	495.513	28.449	17.6	0.244	0.892		
751084233	495.764	28.449	4.8	0.266	0.672		
751084234	496.015	28.422	29.1	0.119	0.683		
751084235	760.397	24.443	-1.9	0.845	0.358		
751084236	760.896	24.455	-2.4	0.679	0.204		
751084237	478.024	30.242	-2.6	0.716	0.493		
751084238	478.275	30.24	0.5	0.971	0.494		
751084239	478.525	30.223	-8.4	0.711	0.438		
751084240	493.557	25.638	3	0.274	0.9		
751084241	493.892	25.646	-18.3	0.358	0.666		
751084242	494.226	25.654	10.2	0.644	0.772		
751084243	992.989	32.117	0.7	0.884	0.429		
751084244	993.49	32.111	-0.3	0.92	0.232		
751084245	993.993	32.103	-7.1	0.363	0.487		
751084246	1250.893	36.219	-2.5	0.624	0.166		
751084247	1251.232	36.251	2	0.637	0.684		
751084248	1251.565	35.879	3.2	0.415	0.809		
751084249	1251.91	36.207	1.4	0.712	0.77		
751084250	1252.249	36.188	-0.4	0.925	0.5		
751084251	1252.586	36.166	-2	0.601	0.469		
751084252	740.723	27.584	9.9	0.205	0.938		
751084253	741.057	27.572	-11	0.328	0.162		
751084254	741.393	27.57	6.8	0.619	0.943		
751084255	825.125	31.392	-14.8	0.118	0.117		
751084256	825.375	31.378	-11.5	0.364	0.607		
751084257	825.626	31.385	0.6	0.947	0.596		
751084258	825.877	31.291	0.1	0.993	0.569		
751084259	540.292	15.169	23	0.346	0.941		
751084260	540.794	15.175	16.9	0.549	0.931		
751084261	583.292	17.118	2.4	0.676	0.561		
751084262	583.794	17.1	-10.1	0.391	0.208		
751084263	909.461	22.964	-6.6	0.548	0.111		
751084264	910.466	22.989	-28.4	0.22	0.061		
751084265	517.744	23.166	7.6	0.105	0.992		
751084266	517.995	23.17	5.7	0.208	0.663		
751084267	518.246	23.172	4.9	0.442	0.487		
751084268	518.496	23.163	20.5	0.525	0.942		
751084269	796.176	41.359	0	0.998	0.847		

751084270	796.426	41.365	-2.4	0.873	0.794		
751084271	796.678	41.368	0	0.996	0.602		
751084272	796.929	41.371	-2.6	0.881	0.88		
751084273	797.18	41.372	-3.7	0.799	0.384		
751084274	395.959	15.759	16.7	0.103	0.448		
751084275	396.209	15.768	18.5	0.096	0.356		
751084276	396.46	15.753	13.5	0.643	0.734		
751084277	531.962	39.134	0.2	0.984	0.885		
751084278	532.297	39.098	5.1	0.667	0.373		
751084279	532.631	39.05	27.8	0.369	0.786		
751084280	736.719	39.487	1.3	0.808	0.239		
751084281	737.052	39.51	1.6	0.781	0.361		
751084282	737.385	39.464	1.7	0.783	0.389		
751084283	737.722	39.472	1.5	0.861	0.228		
751084284	802.922	33.102	8.1	0.143	0.678		
751084285	803.918	33.091	7	0.588	0.576		
751084286	1202.655	31.156	5.2	0.353	0.466		
751084287	1203.658	31.154	2.8	0.611	0.371		
751084288	590.931	23.239	7.8	0.036	0.927		
751084289	591.265	23.245	8.5	0.049	0.816		
751084290	591.599	23.247	6.5	0.638	0.732		
751084291	996.311	40.831	6.3	0.274	0.538		
751084292	996.825	40.752	5.3	0.693	0.49		
751084293	651.652	25.134	-4.2	0.521	0.277		
751084294	651.987	25.125	-1.4	0.804	0.557		
751084295	652.322	25.12	1.1	0.86	0.675		
751084296	560.291	24.404	1.3	0.884	0.16		
751084297	560.794	24.416	0.3	0.987	0.23		
751084298	530.32	16.484	5.7	0.675	0.166		
751084299	1014.477	20.442	-3.6	0.671	0.155		
751084300	621.59	21.938	8.3	0.176	0.8	Tax_Id=9606 Gene_Symbol=CP Ceruloplasmin	KAEEEHLGILGPQLHADVGDKVK
751084301	621.842	21.906	8.5	0.132	0.883	Tax_Id=9606 Gene_Symbol=CP Ceruloplasmin	KAEEEHLGILGPQLHADVGDKVK
751084302	622.091	21.9	5.7	0.732	0.84	Tax_Id=9606 Gene_Symbol=CP Ceruloplasmin	KAEEEHLGILGPQLHADVGDKVK
751084303	593.342	36.534	0.9	0.892	0.667		
751084304	593.845	36.963	-22.4	0.074	0.068		
751084305	992.18	39.473	0.6	0.951	0.179	Tax_Id=9606 Gene_Symbol=CHGB Secretogranin-1	ADQTVLTEDEKKELENLAAMDLELQK
751084306	992.514	39.481	0.6	0.942	0.138	Tax_Id=9606 Gene_Symbol=CHGB Secretogranin-1	ADQTVLTEDEKKELENLAAMDLELQK
751084307	992.849	39.481	2.7	0.723	0.258	Tax_Id=9606 Gene_Symbol=CHGB Secretogranin-1	ADQTVLTEDEKKELENLAAMDLELQK
751084308	993.192	39.786	-0.6	0.876	0.562	Tax_Id=9606 Gene_Symbol=CHGB Secretogranin-1	ADQTVLTEDEKKELENLAAMDLELQK
751084309	537.289	22.492	12.6	0.545	0.497		
751084310	537.791	22.523	7	0.715	0.09		
751084311	773.384	25.809	1.5	0.782	0.866		
751084312	773.884	25.888	-10.5	0.311	0.188		
751084313	541.305	18.867	6.8	0.087	0.621		
751084314	541.638	18.924	18	0.057	0.449		
751084315	505.59	29.37	14	0.548	0.261		
751084316	505.924	29.375	12.4	0.654	0.175		
751084317	1157.602	21.507	7.3	0.456	0.642	Tax_Id=9606 Gene_Symbol=CLU Isoform 2 of Clusterin	VTTVASHTSDSDVPSGVTEVVVK
751084318	1158.105	21.503	6.5	0.512	0.884	Tax_Id=9606 Gene_Symbol=CLU Isoform 2 of Clusterin	VTTVASHTSDSDVPSGVTEVVVK
751084319	1158.588	21.564	8.2	0.659	0.436	Tax_Id=9606 Gene_Symbol=CLU Isoform 2 of Clusterin	VTTVASHTSDSDVPSGVTEVVVK
751084320	557.306	43.669	3.4	0.713	0.763		
751084321	557.64	43.662	4.3	0.672	0.772		
751084322	557.975	43.663	14.8	0.651	0.909		
751084323	821.059	37.493	-0.6	0.945	0.296		
751084324	821.392	37.643	1	0.871	0.527		
751084325	821.729	37.49	4.6	0.562	0.949		
751084326	822.077	37.753	3.1	0.483	0.494		
751084327	572.59	44.01	10.5	0.636	0.481		
751084328	572.841	44.011	3.4	0.702	0.192		
751084329	573.092	44.01	8.1	0.704	0.409		
751084330	573.343	43.981	23.6	0.172	0.848		
751084331	686.313	31.837	7.3	0.276	0.852		
751084332	686.813	31.84	8.3	0.207	0.987		
751084333	610.622	18.842	-1.6	0.795	0.216		
751084334	610.955	18.839	0.4	0.958	0.652		
751084335	611.29	18.823	-3.3	0.47	0.115		
751084336	611.624	18.75	-0.3	0.972	0.198		
751084337	696.858	26.854	1	0.838	0.387		
751084338	697.36	26.834	-2.2	0.837	0.906		
751084339	848.785	31.241	2.7	0.912	0.561		
751084340	849.126	31.275	3.6	0.691	0.338		
751084341	849.459	31.277	7.6	0.495	0.176		
751084342	496.283	16.141	23.3	0.315	0.786		
751084343	496.785	16.142	-6.8	0.735	0.112		
751084344	483.567	20.545	2.3	0.732	0.582		
751084345	483.901	20.534	-8.1	0.503	0.162		
751084346	624.836	21.292	4.2	0.47	0.442		
751084347	625.336	21.304	6.2	0.583	0.598		
751084348	645.058	48.537	-0.2	0.991	0.338		
751084349	645.311	48.516	-7.4	0.483	0.159		
751084350	645.56	48.536	-18.1	0.156	0.051		
751084351	645.813	48.533	-33	0.052	0.052		
751084352	675.358	14.118	1.4	0.921	0.147		
751084353	676.365	14.015	-2.2	0.855	0.086		
751084354	524.482	21.48	-18.7	0.124	0.145	Tax_Id=9606 Gene_Symbol=GSN Isoform 1 of Gelsolin	VPEARPNSM[147.0355]VVEHPEFLK
751084355	524.733	21.461	-2.4	0.538	0.175	Tax_Id=9606 Gene_Symbol=GSN Isoform 1 of Gelsolin	VPEARPNSM[147.0355]VVEHPEFLK
751084356	524.984	21.413	-3.6	0.379	0.468	Tax_Id=9606 Gene_Symbol=GSN Isoform 1 of Gelsolin	VPEARPNSM[147.0355]VVEHPEFLK
751084357	559.6	21.374	1.5	0.811	0.293		
751084358	559.934	21.376	0.5	0.95	0.404		
751084359	374.722	18.266	1.1	0.938	0.865		
751084360	515.913	22.174	-31.1	0.296	0.301	Tax_Id=9606 Gene_Symbol=CLEC3B Putative uncharacterized protein DKFZ	NWETEITAQPDGGK
751084361	516.247	22.156	2.9	0.87	0.466	Tax_Id=9606 Gene_Symbol=CLEC3B Putative uncharacterized protein DKFZ	NWETEITAQPDGGK
751084362	516.581	22.188	22.4	0.396	0.638	Tax_Id=9606 Gene_Symbol=CLEC3B Putative uncharacterized protein DKFZ	NWETEITAQPDGGK

751084363	1050.494	38.858	9.5	0.441	0.658		
751084364	1050.829	38.862	7.3	0.305	0.694		
751084365	1051.163	38.865	9	0.2	0.982		
751084366	1051.498	38.87	8.6	0.313	0.701		
751084367	1051.832	38.867	6.4	0.553	0.843		
751084368	726.026	30.894	9.9	0.644	0.666		
751084369	726.363	30.869	2.6	0.726	0.919		
751084370	546.322	37.585	0.4	0.855	0.794		
751084371	547.325	37.604	13.3	0.468	0.648		
751084372	973.706	45.484	6.9	0.205	0.967		
751084373	973.908	45.504	4.1	0.479	0.718		
751084374	974.109	45.504	5.3	0.4	0.443		
751084375	974.309	45.503	5.3	0.35	0.806		
751084376	974.51	45.503	1.6	0.81	0.421		
751084377	974.707	45.499	5.9	0.415	0.665		
751084378	506.295	35.057	-4.7	0.314	0.047	Tax_Id=9606 Gene_Symbol=APP Isoform L-APP733 of Amyloid beta A4 prot	LALENYITALQAVPPRPR
751084379	506.546	35.056	-7.2	0.247	0.035	Tax_Id=9606 Gene_Symbol=APP Isoform L-APP733 of Amyloid beta A4 prot	LALENYITALQAVPPRPR
751084380	506.797	35.036	-25.6	0.054	0.025	Tax_Id=9606 Gene_Symbol=APP Isoform L-APP733 of Amyloid beta A4 prot	LALENYITALQAVPPRPR
751084381	692.074	30.096	17.6	0.304	0.887		
751084382	692.325	30.094	6.5	0.363	0.619		
751084383	692.575	30.095	7.8	0.621	0.395		
751084384	692.827	30.072	3.4	0.639	0.532		
751084385	702.004	19.757	-2.9	0.558	0.26		
751084386	427.744	25.425	6.8	0.257	0.662		
751084387	428.245	25.436	17.6	0.046	0.386		
751084388	427.754	23.47	7.5	0.174	0.876		
751084389	428.256	23.486	10.6	0.614	0.504		
751084390	1030.504	38.684	6.9	0.643	0.794		
751084391	1031.008	38.693	5.6	0.612	0.26		
751084392	1031.499	38.702	4.5	0.644	0.323		
751084393	397.227	16.045	4.7	0.586	0.799		
751084394	397.729	16.052	53.9	0.06	0.471		
751084395	1237.214	35.446	5.5	0.177	0.607		
751084396	1237.548	35.385	4.6	0.186	0.828		
751084397	1237.88	35.364	4.5	0.257	0.775		
751084398	1238.215	35.333	5.2	0.234	0.727		
751084399	1238.557	35.076	1.1	0.668	0.788		
751084400	755.336	15.783	-2.2	0.713	0.158		
751084401	755.839	15.806	4.1	0.808	0.945		
751084402	611.96	18.799	2.7	0.84	0.429		
751084403	612.295	18.841	3.9	0.765	0.531		
751084404	544.336	21.633	8.2	0.673	0.548		
751084405	768.583	26.946	-15	0.283	0.132		
751084406	768.834	26.941	-25.8	0.041	0.053		
751084407	769.086	26.949	-8.8	0.088	0.037		
751084408	769.337	26.941	-13.2	0.013	0.008		
751084409	769.588	26.943	-3.9	0.807	0.486		
751084410	572.768	26.752	7.2	0.73	0.434		
751084411	573.019	26.728	-8.5	0.566	0.103		
751084412	573.27	26.689	-1.4	0.884	0.19		
751084413	573.521	26.689	7.6	0.821	0.511		
751084414	670.498	46.33	12.5	0.357	0.768		
751084415	497.268	17.531	6.7	0.499	0.424	Tax_Id=9606 Gene_Symbol=TIMP1 Metalloproteinase inhibitor 1	SEEFLIAGK
751084416	497.77	17.558	4.5	0.819	0.663	Tax_Id=9606 Gene_Symbol=TIMP1 Metalloproteinase inhibitor 1	SEEFLIAGK
751084417	544.766	14.994	6.7	0.673	0.207		
751084418	545.268	14.983	-13.7	0.52	0.181		
751084419	635.781	27.04	5.9	0.249	0.051		
751084420	636.283	27.057	-2.7	0.751	0.06		
751084421	589.003	25.099	3.9	0.517	0.697	Tax_Id=9606 Gene_Symbol=PKM2 Isoform M1 of Pyruvate kinase isozymes	KGVNLPGAAVDLPAVSEK
751084422	589.337	25.095	2.8	0.83	0.467	Tax_Id=9606 Gene_Symbol=PKM2 Isoform M1 of Pyruvate kinase isozymes	KGVNLPGAAVDLPAVSEK
751084423	589.671	25.092	8.5	0.784	0.7	Tax_Id=9606 Gene_Symbol=PKM2 Isoform M1 of Pyruvate kinase isozymes	KGVNLPGAAVDLPAVSEK
751084424	947.43	42.074	8.3	0.257	0.501		
751084425	947.677	42.16	11.6	0.099	0.873		
751084426	947.929	42.098	11.6	0.11	0.962		
751084427	948.179	42.068	9.5	0.471	0.387		
751084428	948.432	42.058	2.9	0.725	0.49		
751084429	467.767	20.668	7.4	0.095	0.652		
751084430	468.269	20.733	11.4	0.313	0.872		
751084431	660.792	19.607	1	0.844	0.72	Tax_Id=9606 Gene_Symbol=SDF4 45 kDa calcium-binding protein isoform 1	DLGGFDEDAEPR
751084432	661.296	19.616	-1.3	0.817	0.212	Tax_Id=9606 Gene_Symbol=SDF4 45 kDa calcium-binding protein isoform 1	DLGGFDEDAEPR
751084433	474.243	21.398	10.1	0.03	0.473		
751084434	474.576	21.373	19.7	0.153	0.8		
751084435	828.486	22.018	2.3	0.85	0.203		
751084436	1008.447	28.004	-1.7	0.783	0.273		
751084437	1008.782	27.931	-1.9	0.776	0.127		
751084438	1009.116	27.928	-1.8	0.822	0.16		
751084439	1009.45	27.917	-18.7	0.305	0.326		
751084440	729.638	36.7	-1.3	0.792	0.129		
751084441	730.141	36.695	2.3	0.623	0.341		
751084442	764.333	14.3	14.6	0.112	0.819		
751084443	1039.225	35.046	6.8	0.698	0.458		
751084444	1039.476	34.98	-21.5	0.176	0.184		
751084445	1039.726	35.009	4	0.507	0.311		
751084446	1039.976	34.997	5.7	0.546	0.391		
751084447	1040.226	34.914	-4.7	0.814	0.269		
751084448	1105.599	23.4	7.9	0.653	0.609		
751084449	1106.602	23.408	20.7	0.408	0.444		
751084450	780.983	48.179	2.5	0.569	0.636		
751084451	781.125	48.17	3.4	0.545	0.341		
751084452	781.268	48.168	2.5	0.585	0.45		
751084453	781.412	48.174	1.7	0.891	0.331		
751084454	781.555	48.173	1.1	0.887	0.595		
751084455	515.261	22.804	2.4	0.657	0.498		

751084456	515.595	22.811	6.4	0.754	0.48		
751084457	710.03	31.691	-9.1	0.427	0.032		
751084458	710.367	31.659	-3.8	0.718	0.024		
751084459	500.261	16.655	6.4	0.32	0.466		
751084460	504.764	22.655	3.8	0.796	0.141		
751084461	605.38	42.251	6.9	0.203	0.593		
751084462	605.882	42.299	2.4	0.786	0.216		
751084463	807.405	47.749	-4.8	0.24	0.144		
751084464	807.607	47.75	-3.2	0.469	0.117		
751084465	807.807	47.748	-3.6	0.506	0.356		
751084466	808.008	47.745	-2.9	0.482	0.174		
751084467	808.207	47.723	-7.3	0.124	0.105		
751084468	1305.243	30.811	5.7	0.201	0.486		
751084469	1305.579	30.843	5.5	0.133	0.839		
751084470	1305.913	30.837	6	0.099	0.651		
751084471	1306.248	30.828	7.4	0.202	0.821		
751084472	1306.583	30.833	5.4	0.265	0.561		
751084473	679.591	18.207	14.4	0.779	0.437		
751084474	679.84	18.188	25.7	0.329	0.137		
751084475	680.091	18.191	13.2	0.689	0.413		
751084476	390.936	14.937	-3	0.562	0.686		
751084477	864.788	44.935	-0.7	0.883	0.277		
751084478	883.609	29.147	-32.3	0.084	0.077		
751084479	883.809	29.152	-9.7	0.224	0.05		
751084480	884.01	29.153	-20.1	0.053	0.028		
751084481	884.21	29.155	-24.6	0.134	0.062		
751084482	884.411	29.153	-27.1	0.107	0.103		
751084483	884.612	29.185	-29.3	0.186	0.148		
751084484	830.903	26.639	2.1	0.823	0.385		
751084485	831.404	26.647	5.9	0.672	0.459		
751084486	831.907	26.638	-26.3	0.167	0.036		
751084487	717.675	17.81	40.7	0.297	0.748		
751084488	718.009	17.811	22.7	0.413	0.947		
751084489	718.343	17.865	7.7	0.416	0.346		
751084490	576.047	23.095	5.1	0.146	0.674		
751084491	576.3	23.131	4.2	0.238	0.745		
751084492	698.376	14.815	-4.3	0.756	0.09		
751084493	755.914	26.14	11.3	0.159	0.39	Tax_Id=9606 Gene_Symbol=PCOLCE Procollagen C-endopeptidase enhanc	TGGLDLPSPPTGASLK
751084494	756.416	26.135	5.1	0.476	0.739	Tax_Id=9606 Gene_Symbol=PCOLCE Procollagen C-endopeptidase enhanc	TGGLDLPSPPTGASLK
751084495	599.378	16.151	-0.2	0.985	0.189		
751084496	566.305	16.081	-11.3	0.285	0.065		
751084497	566.806	16.067	-11.1	0.56	0.17		
751084498	582.343	39.468	1.7	0.906	0.359		
751084499	582.677	39.464	8.9	0.449	0.454		
751084500	583.012	39.471	-12.1	0.612	0.557		
751084501	542.864	16.234	1.3	0.932	0.584		
751084502	543.064	16.242	-18.2	0.35	0.445		
751084503	543.267	16.261	-3.4	0.884	0.459		
751084504	543.465	16.232	-19.3	0.475	0.31		
751084505	766.1	33.788	-3.3	0.356	0.109		
751084506	766.353	33.831	-0.5	0.917	0.291		
751084507	766.604	33.87	-0.6	0.905	0.33		
751084508	766.854	33.77	-13.5	0.38	0.221		
751084509	767.089	33.919	-1.3	0.883	0.703		
751084510	593.031	36.872	0.1	0.982	0.065		
751084511	593.37	36.812	2.7	0.841	0.297		
751084512	593.7	36.88	-8.7	0.531	0.035		
751084513	469.566	16.47	-2	0.881	0.204		
751084514	469.901	16.47	-24.3	0.442	0.196		
751084515	1129.835	34.01	7	0.636	0.582		
751084516	1130.172	34.016	28.1	0.059	0.172		
751084517	1130.505	34.028	11.4	0.144	0.192		
751084518	1130.839	34.019	5.2	0.611	0.956		
751084519	673.329	27.431	6.6	0.501	0.331		
751084520	673.83	27.373	5	0.64	0.28		
751084521	693.34	26.203	1.1	0.836	0.434		
751084522	693.675	26.18	2	0.728	0.712		
751084523	694.009	26.191	0.4	0.932	0.267		
751084524	990.144	41.083	4.1	0.634	0.389	Tax_Id=9606 Gene_Symbol=ITIH4 Isoform 2 of Inter-alpha-trypsin inhibitor h	FSSHVGGTLGQFYQEVLWGSPAASDDGR
751084525	990.475	41.075	9.5	0.28	0.846	Tax_Id=9606 Gene_Symbol=ITIH4 Isoform 2 of Inter-alpha-trypsin inhibitor h	FSSHVGGTLGQFYQEVLWGSPAASDDGR
751084526	990.813	41.06	4.9	0.626	0.511	Tax_Id=9606 Gene_Symbol=ITIH4 Isoform 2 of Inter-alpha-trypsin inhibitor h	FSSHVGGTLGQFYQEVLWGSPAASDDGR
751084527	991.146	41.089	7.2	0.386	0.953	Tax_Id=9606 Gene_Symbol=ITIH4 Isoform 2 of Inter-alpha-trypsin inhibitor h	FSSHVGGTLGQFYQEVLWGSPAASDDGR
751084528	671.396	33.569	3.6	0.813	0.718		
751084529	671.898	33.584	-3.9	0.816	0.87		
751084530	681.295	19.848	3.3	0.717	0.223		
751084531	681.629	19.799	9	0.637	0.253		
751084532	681.964	19.812	-23.4	0.123	0.087		
751084533	861.398	23.853	-1.2	0.715	0.574	Tax_Id=9606 Gene_Symbol=PENK 25 kDa protein	DAEEDDSLANSSDLLK
751084534	861.898	23.988	1	0.775	0.431	Tax_Id=9606 Gene_Symbol=PENK 25 kDa protein	DAEEDDSLANSSDLLK
751084535	862.399	23.979	-4.5	0.556	0.315	Tax_Id=9606 Gene_Symbol=PENK 25 kDa protein	DAEEDDSLANSSDLLK
751084536	626.659	22.263	4.3	0.415	0.444		
751084537	628.793	15.254	-11.6	0.504	0.11		
751084538	629.294	15.248	-17.1	0.397	0.108		
751084539	425.241	17.536	10.3	0.109	0.619		
751084540	528.293	20.865	-5.4	0.295	0.296		
751084541	528.794	20.876	-15.2	0.161	0.106		
751084542	533.262	16.018	-22.3	0.127	0.008		
751084543	533.763	16.021	-40.3	0.08	0.024		
751084544	1204.539	31.271	-1.5	0.865	0.314		
751084545	482.239	29.835	1.6	0.865	0.298		
751084546	482.438	29.827	-0.1	0.996	0.398		
751084547	482.638	29.821	-4.7	0.81	0.363		
751084548	482.839	29.823	9	0.719	0.592		

751084549	551.775	27.218	-3.1	0.822	0.245		
751084550	552.277	27.22	-13.6	0.337	0.043		
751084551	527.62	25.718	2.3	0.794	0.35	Tax_Id=9606 Gene_Symbol=COL6A1 Collagen alpha-1(VI) chain	VFSVAITPDHLEPR
751084552	527.955	25.675	2.2	0.593	0.501	Tax_Id=9606 Gene_Symbol=COL6A1 Collagen alpha-1(VI) chain	VFSVAITPDHLEPR
751084553	528.289	25.668	-0.8	0.958	0.469	Tax_Id=9606 Gene_Symbol=COL6A1 Collagen alpha-1(VI) chain	VFSVAITPDHLEPR
751084554	740.839	19.484	1	0.931	0.133	Tax_Id=9606 Gene_Symbol=DKK3 cDNA FLJ52545, highly similar to Dickko	SAVEEM[147.0355]EAEEAAAK
751084555	741.337	19.488	0.2	0.972	0.107	Tax_Id=9606 Gene_Symbol=DKK3 cDNA FLJ52545, highly similar to Dickko	SAVEEM[147.0355]EAEEAAAK
751084556	822.895	24.928	2.2	0.686	0.616	Tax_Id=9606 Gene_Symbol=SERPINF1 Pigment epithelium-derived factor	KTSLEDFYLDEER
751084557	823.4	24.961	3	0.82	0.812	Tax_Id=9606 Gene_Symbol=SERPINF1 Pigment epithelium-derived factor	KTSLEDFYLDEER
751084558	711.839	20.246	17.5	0.171	0.67		
751084559	712.34	20.263	0.4	0.958	0.306		
751084560	612.235	14.37	7.8	0.619	0.129		
751084561	613.238	14.363	6.3	0.799	0.18		
751084562	1400.695	20.268	20.8	0.431	0.905		
751084563	1401.697	20.295	5	0.681	0.249		
751084564	828.433	41.865	3.7	0.254	0.543		
751084565	829.444	41.645	4.5	0.697	0.451		
751084566	1051.009	29.468	-4.5	0.721	0.262		
751084567	1051.509	29.509	1.8	0.76	0.193		
751084568	481.237	24.592	0.3	0.983	0.107		
751084569	481.739	24.607	11.4	0.667	0.796		
751084570	639.375	26.297	-0.7	0.938	0.231		
751084571	1176.507	36.624	-4.1	0.285	0.049		
751084572	1176.841	36.664	1.1	0.744	0.278		
751084573	1177.175	36.704	0.4	0.9	0.131		
751084574	1177.512	36.678	-0.9	0.784	0.115		
751084575	923.033	37.754	4.3	0.378	0.49	Tax_Id=9606 Gene_Symbol=A2M Alpha-2-macroglobulin	LLIYAVLPTGDVIGDSAK
751084576	923.533	37.772	5.3	0.273	0.683	Tax_Id=9606 Gene_Symbol=A2M Alpha-2-macroglobulin	LLIYAVLPTGDVIGDSAK
751084577	924.034	37.794	6.7	0.258	0.621	Tax_Id=9606 Gene_Symbol=A2M Alpha-2-macroglobulin	LLIYAVLPTGDVIGDSAK
751084578	471.768	16.894	9.9	0.159	0.64		
751084579	472.27	16.889	14.4	0.331	0.962		
751084580	525.264	30.531	6	0.322	0.761	Tax_Id=9606 Gene_Symbol=CFB Isoform 1 of Complement factor B (Fragme	LQDEDLGFL
751084581	525.768	30.495	1.7	0.924	0.421	Tax_Id=9606 Gene_Symbol=CFB Isoform 1 of Complement factor B (Fragme	LQDEDLGFL
751084582	758.059	26.786	-16.3	0.041	0.038	Tax_Id=9606 Gene_Symbol=ALDOA 45 kDa protein	GVVPLAGTNGETTTQGLDGLSER
751084583	758.391	26.796	-2.3	0.594	0.206	Tax_Id=9606 Gene_Symbol=ALDOA 45 kDa protein	GVVPLAGTNGETTTQGLDGLSER
751084584	758.725	26.812	-1.6	0.72	0.4	Tax_Id=9606 Gene_Symbol=ALDOA 45 kDa protein	GVVPLAGTNGETTTQGLDGLSER
751084585	759.059	26.841	-24.9	0.023	0.036	Tax_Id=9606 Gene_Symbol=ALDOA 45 kDa protein	GVVPLAGTNGETTTQGLDGLSER
751084586	760.112	25.779	-19.9	0.093	0.017		
751084587	760.36	25.77	-3.6	0.507	0.14		
751084588	760.614	25.773	-12.2	0.191	0.045		
751084589	760.86	25.873	-24.2	0.185	0.107		
751084590	820.588	37.923	3.7	0.571	0.543		
751084591	820.789	37.954	5.8	0.319	0.614		
751084592	820.985	37.963	4.5	0.327	0.583		
751084593	821.189	37.972	9.3	0.374	0.571		
751084594	476.543	26.673	10.7	0.61	0.547		
751084595	476.877	26.675	13.5	0.414	0.288		
751084596	554.269	21.41	-1.1	0.841	0.286	Tax_Id=9606 Gene_Symbol=CST3 Cystatin-C	LVGGPM[147.0355]DASVEEEGVR
751084597	554.603	21.408	-0.2	0.986	0.161	Tax_Id=9606 Gene_Symbol=CST3 Cystatin-C	LVGGPM[147.0355]DASVEEEGVR
751084598	1144.862	36.478	-0.2	0.953	0.517	Tax_Id=9606 Gene_Symbol=CHGA Chromogranin-A	HSGFEDELSEVLENQSSQAELKEAVEEPSSK
751084599	1145.194	36.475	-6.6	0.337	0.263	Tax_Id=9606 Gene_Symbol=CHGA Chromogranin-A	HSGFEDELSEVLENQSSQAELKEAVEEPSSK
751084600	1145.528	36.478	-2.2	0.61	0.262	Tax_Id=9606 Gene_Symbol=CHGA Chromogranin-A	HSGFEDELSEVLENQSSQAELKEAVEEPSSK
751084601	1145.86	36.493	-1.2	0.754	0.338	Tax_Id=9606 Gene_Symbol=CHGA Chromogranin-A	HSGFEDELSEVLENQSSQAELKEAVEEPSSK
751084602	1146.192	36.532	-5.9	0.512	0.384	Tax_Id=9606 Gene_Symbol=CHGA Chromogranin-A	HSGFEDELSEVLENQSSQAELKEAVEEPSSK
751084603	456.872	22.781	-5.7	0.295	0.107		
751084604	457.206	22.781	1.1	0.935	0.294		
751084605	556.269	27.903	5.2	0.58	0.215		
751084606	556.605	27.822	8.8	0.637	0.191		
751084607	728.219	66.732	-2.6	0.751	0.879		
751084608	423.736	17.923	-4.1	0.681	0.111		
751084609	633.684	44.344	-8.4	0.079	0.028	Tax_Id=9606 Gene_Symbol=- 52 kDa protein	MDQLEGQLLAQVLALEK
751084610	634.018	44.305	-5.8	0.344	0.093	Tax_Id=9606 Gene_Symbol=- 52 kDa protein	MDQLEGQLLAQVLALEK
751084611	697.663	23.673	-9.1	0.082	0.092		
751084612	1168.523	26.769	-32.5	0.02	0.008		
751084613	1168.773	26.771	-0.8	0.967	0.602		
751084614	1169.025	26.776	0.4	0.985	0.613		
751084615	1169.276	26.777	1.9	0.888	0.468		
751084616	1169.528	26.779	1	0.913	0.382		
751084617	743.078	26.917	-24.9	0.005	0.01		
751084618	743.329	26.916	-8.2	0.168	0.056		
751084619	743.579	26.927	-10.7	0.067	0.04		
751084620	743.83	26.933	-3.8	0.763	0.347		
751084621	514.242	26.921	2.8	0.667	0.776		
751084622	514.744	26.851	5.5	0.707	0.898		
751084623	614.723	20.285	-14.7	0.326	0.158		
751084624	588.838	30.116	2	0.884	0.526		
751084625	589.34	30.117	-7.6	0.425	0.232		
751084626	1022.121	39.788	1.7	0.728	0.763		
751084627	1022.453	39.788	2.8	0.543	0.837		
751084628	1022.787	39.777	3	0.539	0.845		
751084629	1023.12	39.83	-2	0.775	0.249		
751084630	423.592	23.723	-0.1	0.996	0.141	Tax_Id=9606 Gene_Symbol=AGT Angiotensinogen	ALQDQLVLVAAK
751084631	423.926	23.721	21.4	0.24	0.913	Tax_Id=9606 Gene_Symbol=AGT Angiotensinogen	ALQDQLVLVAAK
751084632	801.888	16.425	-31.3	0.039	0.085		
751084633	802.388	16.433	-9.5	0.548	0.562		
751084634	643.644	22.343	0.9	0.926	0.544		
751084635	436.343	66.175	9.4	0.223	0.175		
751084636	633.57	31.832	-1.8	0.882	0.162		
751084637	633.821	31.81	13.9	0.157	0.565		
751084638	634.068	31.903	1.5	0.835	0.743		
751084639	662.327	32.048	1.5	0.689	0.639		
751084640	662.661	32.131	-0.3	0.916	0.128		
751084641	662.996	32.109	-4.3	0.558	0.104		

751084642	1016.69	40.216	2.5	0.443	0.812		
751084643	1016.94	40.287	4.2	0.173	0.953		
751084644	1017.19	40.173	1.1	0.732	0.488		
751084645	1017.44	40.229	3.2	0.252	0.664		
751084646	1017.692	40.293	5.2	0.407	0.49		
751084647	737.406	28.145	-6.2	0.557	0.325		
751084648	737.908	28.157	-3.1	0.644	0.357		
751084649	912.422	18.888	8.1	0.318	0.998		
751084650	912.923	18.911	2.8	0.843	0.789		
751084651	913.425	18.984	-9.3	0.682	0.497		
751084652	809.391	45.61	0.2	0.984	0.435		
751084653	809.724	45.606	1.2	0.88	0.782		
751084654	810.058	45.604	-1.4	0.834	0.395		
751084655	666.997	32.111	0.5	0.913	0.603		
751084656	667.332	32.087	1.5	0.664	0.321		
751084657	667.666	32.122	1.5	0.674	0.552		
751084658	623.582	39.672	14	0.288	0.747		
751084659	719.322	16.71	1.1	0.929	0.324		
751084660	719.824	16.693	-50.1	0.003	0.001		
751084661	440.924	22.039	-20.7	0.462	0.322		
751084662	441.258	22.046	17.7	0.26	0.872		
751084663	450.966	16.643	4.3	0.842	0.179	Tax_Id=9606 Gene_Symbol=CALR Calreticulin	IKDPDASKPEDWDER
751084664	451.217	16.64	6.6	0.768	0.236	Tax_Id=9606 Gene_Symbol=CALR Calreticulin	IKDPDASKPEDWDER
751084665	451.468	16.65	-25.6	0.182	0.018	Tax_Id=9606 Gene_Symbol=CALR Calreticulin	IKDPDASKPEDWDER
751084666	1032.159	45.11	-3.7	0.492	0.324		
751084667	1032.491	45.134	-3.6	0.496	0.515		
751084668	1032.825	45.175	-2.9	0.597	0.468		
751084669	1033.161	45.199	-5.6	0.322	0.472		
751084670	1033.494	45.192	-5.1	0.445	0.645		
751084671	702.315	22.618	0.7	0.909	0.636		
751084672	702.645	22.616	1.8	0.816	0.506		
751084673	702.979	22.618	1.9	0.901	0.713		
751084674	1383.681	47.393	-0.8	0.914	0.279		
751084675	1384.011	47.384	0.5	0.939	0.242		
751084676	1384.344	47.387	-1.9	0.707	0.304		
751084677	1384.677	47.393	-2.2	0.693	0.312		
751084678	1384.985	47.414	1.2	0.849	0.326		
751084679	549.852	21.201	4.9	0.714	0.206		
751084680	768.376	23.133	-1	0.926	0.125		
751084681	768.879	23.148	3	0.828	0.547		
751084682	769.38	23.144	-13.7	0.601	0.863		
751084683	527.74	14.883	4.8	0.748	0.248		
751084684	528.241	14.913	-0.1	0.995	0.329		
751084685	947.177	48.817	-6.5	0.673	0.777		
751084686	947.509	48.767	0.8	0.899	0.539		
751084687	947.847	48.805	-0.3	0.968	0.557		
751084688	948.184	48.812	-0.7	0.92	0.194		
751084689	399.725	22.585	8.9	0.549	0.307		
751084690	472.758	20.549	-2.9	0.77	0.14		
751084691	473.259	20.538	-3.4	0.838	0.398		
751084692	473.76	20.524	1	0.906	0.291		
751084693	642.039	17.862	-3.8	0.855	0.642		
751084694	642.289	17.919	2.9	0.817	0.362		
751084695	642.54	17.865	-6.2	0.763	0.325		
751084696	642.79	17.902	-4.4	0.851	0.725		
751084697	687.872	27.738	2.9	0.648	0.577		
751084698	715.919	26.582	4.1	0.845	0.814		
751084699	716.12	26.587	17	0.176	0.841		
751084700	716.319	26.597	8.7	0.288	0.597		
751084701	716.52	26.585	11	0.447	0.777		
751084702	716.721	26.582	-1.6	0.944	0.483		
751084703	784.105	38.473	3.6	0.63	0.753		
751084704	784.605	38.475	7.2	0.681	0.913		
751084705	524.262	16.663	2.4	0.899	0.372		
751084706	532.295	27.36	0.1	0.996	0.372		
751084707	532.797	27.358	2.3	0.906	0.969		
751084708	537.789	15.884	-0.4	0.976	0.375		
751084709	538.293	15.888	1.6	0.938	0.485		
751084710	558.304	20.892	8.2	0.261	0.799		
751084711	558.805	20.865	2.5	0.798	0.312		
751084712	871.802	43.676	-7.7	0.231	0.456		
751084713	872.005	43.731	6.1	0.646	0.579		
751084714	872.207	43.686	-1.4	0.916	0.33		
751084715	872.406	43.69	-0.1	0.982	0.521		
751084716	419.317	66.495	7.1	0.376	0.254		
751084717	420.321	66.493	5.8	0.691	0.293		
751084718	555.819	16.018	10.4	0.581	0.997	Tax_Id=9606 Gene_Symbol=C3 Complement C3 (Fragment)	VLLDGVQNPR
751084719	556.319	16.04	21.9	0.505	0.628	Tax_Id=9606 Gene_Symbol=C3 Complement C3 (Fragment)	VLLDGVQNPR
751084720	556.27	23.538	3.8	0.501	0.552		
751084721	556.524	23.516	6.3	0.46	0.879		
751084722	556.773	23.53	10.1	0.401	0.783		
751084723	526.732	14.891	0.9	0.915	0.503		
751084724	527.235	14.917	6.5	0.668	0.591		
751084725	546.926	14.868	2.3	0.903	0.543		
751084726	547.262	14.78	10.9	0.382	0.841		
751084727	547.596	14.871	-2	0.951	0.871		
751084728	452.236	26.303	0.2	0.988	0.122		
751084729	452.738	26.327	-16.1	0.204	0.014		
751084730	645.972	18.819	1.5	0.912	0.15		
751084731	646.307	18.858	1.3	0.747	0.207		
751084732	646.641	18.814	-17.7	0.535	0.349		
751084733	964.417	26.292	2.1	0.837	0.106		
751084734	435.242	16.889	-1.4	0.891	0.345		

751084735	435.743	16.847	4.2	0.729	0.378		
751084736	691.001	24.726	5.6	0.446	0.487		
751084737	691.334	24.725	4	0.474	0.346		
751084738	691.674	24.647	0.7	0.94	0.981		
751084739	452.72	16.234	2.5	0.794	0.324		
751084740	452.888	16.232	1.2	0.937	0.361		
751084741	453.055	16.224	-3.4	0.896	0.992		
751084742	676.649	26.834	3.5	0.777	0.347		
751084743	617.319	21.79	43.6	0.124	0.366	Tax_Id=9606 Gene_Symbol=TIMP1 Metalloproteinase inhibitor 1	GFQALGDAADIR
751084744	617.819	21.824	0.4	0.983	0.407	Tax_Id=9606 Gene_Symbol=TIMP1 Metalloproteinase inhibitor 1	GFQALGDAADIR
751084745	761.867	21.248	8.8	0.525	0.854		
751084746	762.87	21.294	-13.5	0.691	0.165		
751084747	558.352	21.272	3.7	0.858	0.315		
751084748	660.835	22.444	-3.4	0.446	0.108	Tax_Id=9606 Gene_Symbol=GOLM1 Golgi membrane protein 1	NIDVFNVEDQK
751084749	661.335	22.406	-4.8	0.362	0.334	Tax_Id=9606 Gene_Symbol=GOLM1 Golgi membrane protein 1	NIDVFNVEDQK
751084750	387.216	17.563	4.9	0.736	0.123		
751084751	387.718	17.57	-19.3	0.543	0.23		
751084752	728.352	25.646	-4.4	0.13	0.212		
751084753	925.457	19.94	0.2	0.991	0.464		
751084754	660.034	17.79	35.4	0.149	0.798		
751084755	660.284	17.78	9.7	0.657	0.607		
751084756	660.535	17.794	-5	0.775	0.318		
751084757	1092.806	37.255	-0.3	0.946	0.198		
751084758	1093.145	37.372	-0.6	0.869	0.088		
751084759	1093.478	37.345	0.2	0.953	0.124		
751084760	1093.812	37.226	1.3	0.733	0.122		
751084761	554.303	45.422	1.2	0.856	0.224		
751084762	1013.446	19.798	5.9	0.687	0.204		
751084763	1013.946	19.806	86.2	0.127	0.486		
751084764	596.472	19.927	-13.9	0.136	0.114		
751084765	448.244	18.098	1.8	0.881	0.431		
751084766	448.578	18.107	-1.4	0.914	0.119		
751084767	640.809	42.228	-3	0.784	0.128		
751084768	641.311	42.234	3.3	0.809	0.298		
751084769	641.813	42.239	24.9	0.525	0.487		
751084770	466.243	18.089	7.8	0.551	0.516		
751084771	466.578	18.114	21.1	0.276	0.82		
751084772	628.692	34.213	6.9	0.742	0.629	Tax_Id=9606 Gene_Symbol=A2M Alpha-2-macroglobulin	VSVQLEASPAFLAVPVEK
751084773	629.026	34.19	-34	0.175	0.228	Tax_Id=9606 Gene_Symbol=A2M Alpha-2-macroglobulin	VSVQLEASPAFLAVPVEK
751084774	629.361	34.171	5.6	0.626	0.96	Tax_Id=9606 Gene_Symbol=A2M Alpha-2-macroglobulin	VSVQLEASPAFLAVPVEK
751084775	669.323	27.46	4.3	0.445	0.812		
751084776	669.827	27.347	1.2	0.87	0.448		
751084777	905.08	27.925	-5	0.77	0.923		
751084778	905.415	27.763	1.6	0.714	0.421		
751084779	905.747	27.943	-10.1	0.579	0.419		
751084780	896.425	36.75	-10.6	0.015	0.003		
751084781	896.76	36.806	-7.9	0.11	0.044		
751084782	897.095	36.578	-10.7	0.014	0.006		
751084783	897.428	36.548	-30.6	0.007	0.012		
751084784	582.291	28.198	2.8	0.606	0.688		
751084785	582.625	28.172	3.1	0.872	0.422		
751084786	582.959	28.132	5.8	0.711	0.561		
751084787	408.569	21.128	12.7	0.345	0.692		
751084788	408.904	21.13	16.4	0.329	0.522		
751084789	687.986	22.153	6.8	0.289	0.946		
751084790	447.349	65.118	9	0.301	0.219		
751084791	695.978	26.275	7.2	0.101	0.542		
751084792	696.313	26.314	4.7	0.213	0.865		
751084793	696.65	26.3	18.1	0.008	0.108		
751084794	565.801	21.992	0.2	0.987	0.252		
751084795	923.969	33.032	-0.4	0.965	0.544		
751084796	924.472	32.87	-8	0.305	0.13		
751084797	924.976	32.869	-17.1	0.308	0.208		
751084798	560.79	38.021	2.1	0.882	0.14		
751084799	561.293	38.035	-8.3	0.409	0.068		
751084800	838.926	26.628	-3.5	0.884	0.69		
751084801	839.425	26.615	-6.1	0.711	0.369		
751084802	839.931	26.64	-57.4	0.007	0.026		
751084803	622.328	26.979	1.8	0.884	0.348	Tax_Id=9606 Gene_Symbol=PRRT3 Isoform 1 of Proline-rich transmembran	HAPAEEMPEKPVASPLGPALYGPK
751084804	622.579	26.995	-6.4	0.49	0.055	Tax_Id=9606 Gene_Symbol=PRRT3 Isoform 1 of Proline-rich transmembran	HAPAEEMPEKPVASPLGPALYGPK
751084805	622.829	26.991	-6.8	0.449	0.035	Tax_Id=9606 Gene_Symbol=PRRT3 Isoform 1 of Proline-rich transmembran	HAPAEEMPEKPVASPLGPALYGPK
751084806	611.293	25.782	3.6	0.429	0.587		
751084807	611.624	25.845	3.2	0.795	0.375		
751084808	776.363	36.673	0.8	0.951	0.58		
751084809	776.698	36.767	-1.5	0.747	0.544		
751084810	777.032	36.737	-0.1	0.994	0.828		
751084811	803.88	21.474	5.4	0.555	0.339		
751084812	804.382	21.481	-10.2	0.597	0.179		
751084813	911.526	21.211	6.7	0.644	0.315		
751084814	912.53	21.238	-16.5	0.568	0.09		
751084815	631.291	26.409	7.7	0.568	0.721		
751084816	631.63	26.413	8.4	0.695	0.482		
751084817	631.966	26.41	7.9	0.702	0.463		
751084818	656.401	18.906	7.8	0.326	0.878		
751084819	575.443	36.085	18.8	0.636	0.971		
751084820	575.586	36.09	13.8	0.455	0.444		
751084821	575.729	36.095	18.4	0.322	0.705		
751084822	575.872	36.096	1.5	0.932	0.205		
751084823	576.016	36.088	11.5	0.572	0.659		
751084824	576.159	36.085	-1.8	0.964	0.789		
751084825	479.926	16.043	-17.4	0.115	0.023		
751084826	480.26	16.042	-31	0.026	0.009		
751084827	702.858	26.231	-2.6	0.651	0.136		

751084828	902.155	31.152	6	0.632	0.409		
751084829	902.407	31.207	4.1	0.28	0.769		
751084830	902.656	31.181	4.8	0.223	0.597		
751084831	902.909	31.176	4	0.666	0.424		
751084832	559.347	14.898	-2	0.849	0.127		
751084833	1117.617	20.848	3.2	0.74	0.328	Tax_Id=9606 Gene_Symbol=CLU Isoform 2 of Clusterin	TLLSNLEEAK
751084834	1118.617	20.841	8.2	0.67	0.537	Tax_Id=9606 Gene_Symbol=CLU Isoform 2 of Clusterin	TLLSNLEEAK
751084835	526.564	15.913	-12	0.403	0.094		
751084836	526.899	15.91	-0.4	0.962	0.197		
751084837	496.936	14.147	-20.5	0.524	0.293		
751084838	497.271	14.146	-2	0.877	0.32		
751084839	497.605	14.149	50.8	0.104	0.564		
751084840	654.276	14.41	-19.7	0.398	0.127		
751084841	654.776	14.424	17.8	0.341	0.641		
751084842	495.58	23.299	9.1	0.687	0.191		
751084843	495.914	23.299	2.7	0.882	0.795		
751084844	676.322	16.007	4.3	0.487	0.596	Tax_Id=9606 Gene_Symbol=IGFBP2 insulin-like growth factor-binding protei	LEGEAC[160.0302]GVYTPR
751084845	676.823	16.001	3	0.859	0.597	Tax_Id=9606 Gene_Symbol=IGFBP2 insulin-like growth factor-binding protei	LEGEAC[160.0302]GVYTPR
751084846	547.913	30.138	4.9	0.453	0.764		
751084847	548.247	30.14	2.4	0.798	0.898		
751084848	548.582	30.134	4.3	0.872	0.839		
751084849	891.443	37.578	2.3	0.478	0.798		
751084850	891.944	37.581	4.5	0.241	0.67		
751084851	892.445	37.531	4.2	0.719	0.733		
751084852	878.976	46.023	9.1	0.346	0.625		
751084853	879.475	46.071	13.4	0.162	0.449		
751084854	879.979	46.042	9.2	0.565	0.511		
751084855	938.488	16.391	-2.4	0.916	0.848		
751084856	939.491	16.387	8	0.789	0.693		
751084857	829.398	32.781	2.7	0.863	0.492		
751084858	829.9	32.852	2	0.847	0.207		
751084859	830.406	32.923	-0.9	0.962	0.374		
751084860	729.356	39.66	7.6	0.578	0.537		
751084861	729.858	39.682	1.9	0.679	0.184		
751084862	729.889	36.696	-1.3	0.822	0.177		
751084863	461.578	15.881	-3.6	0.54	0.069	Tax_Id=9606 Gene_Symbol=SULF2 Isoform 1 of Extracellular sulfatase Sulf-	VYHVGLGDAAQPR
751084864	461.913	15.879	-10.2	0.533	0.38	Tax_Id=9606 Gene_Symbol=SULF2 Isoform 1 of Extracellular sulfatase Sulf-	VYHVGLGDAAQPR
751084865	645.574	24.154	3.6	0.536	0.576		
751084866	645.824	24.07	0.2	0.939	0.136		
751084867	646.074	24.187	-1.5	0.657	0.165		
751084868	491.324	21.27	-2	0.844	0.166		
751084869	595.3	21.821	6.7	0.635	0.731		
751084870	595.8	21.829	2.8	0.754	0.303		
751084871	592.688	23.643	-6.2	0.74	0.023	Tax_Id=9606 Gene_Symbol=CALR Calreticulin	AKIDDPTDSKPEDWDKPEHIPDPDAK
751084872	592.888	23.655	12.7	0.575	0.279	Tax_Id=9606 Gene_Symbol=CALR Calreticulin	AKIDDPTDSKPEDWDKPEHIPDPDAK
751084873	593.089	23.652	-0.4	0.969	0.096	Tax_Id=9606 Gene_Symbol=CALR Calreticulin	AKIDDPTDSKPEDWDKPEHIPDPDAK
751084874	593.292	23.637	-3.4	0.745	0.187	Tax_Id=9606 Gene_Symbol=CALR Calreticulin	AKIDDPTDSKPEDWDKPEHIPDPDAK
751084875	609.717	20.216	-30	0.008	0.015		
751084876	658.879	29.859	12.9	0.254	0.739		
751084877	646.001	33.952	4.3	0.831	0.447		
751084878	646.333	33.997	1.4	0.787	0.536		
751084879	646.666	34.089	1.6	0.786	0.286		
751084880	714.328	17.408	-4.3	0.488	0.297		
751084881	714.831	17.395	-7	0.565	0.268		
751084882	553.949	19.671	17.7	0.055	0.667		
751084883	554.283	19.67	19.5	0.396	0.613		
751084884	746.571	37.094	3.9	0.672	0.469	Tax_Id=9606 Gene_Symbol=SCRG1 Putative uncharacterized protein SCRG	ILKDHNC[160.0302]HNLPEGVADLTQIDVNVQDH
751084885	746.771	37.093	6.6	0.345	0.677	Tax_Id=9606 Gene_Symbol=SCRG1 Putative uncharacterized protein SCRG	ILKDHNC[160.0302]HNLPEGVADLTQIDVNVQDH
751084886	746.971	37.116	6	0.502	0.565	Tax_Id=9606 Gene_Symbol=SCRG1 Putative uncharacterized protein SCRG	ILKDHNC[160.0302]HNLPEGVADLTQIDVNVQDH
751084887	747.172	37.08	6.4	0.368	0.343	Tax_Id=9606 Gene_Symbol=SCRG1 Putative uncharacterized protein SCRG	ILKDHNC[160.0302]HNLPEGVADLTQIDVNVQDH
751084888	984.985	24.089	2.6	0.681	0.498		
751084889	778.623	66.046	6.7	0.4	0.237		
751084890	779.627	66.028	4.2	0.613	0.298		
751084891	561.845	34.164	45.1	0.131	0.608		
751084892	562.348	34.146	-1.5	0.807	0.08		
751084893	1348.281	40.297	8.7	0.012	0.579		
751084894	1348.615	40.345	5.4	0.134	0.938		
751084895	1348.949	40.347	6	0.094	0.908		
751084896	1349.284	40.332	5.1	0.165	0.899		
751084897	1349.62	40.353	5.6	0.111	0.854		
751084898	1349.936	40.25	5.5	0.108	0.733		
751084899	571.259	66.137	-1.3	0.855	0.429		
751084900	603.333	30.59	7.7	0.073	0.686		
751084901	603.666	30.566	5.8	0.158	0.776		
751084902	479.766	26.989	9	0.679	0.454		
751084903	842.933	40.969	2.2	0.644	0.751		
751084904	843.435	40.936	2.7	0.582	0.684		
751084905	1197.223	36.284	-1.2	0.757	0.229		
751084906	1197.558	36.345	-2.7	0.544	0.348		
751084907	1197.892	36.368	-4.3	0.382	0.298		
751084908	1198.23	36.411	-21.1	0.018	0.031		
751084909	533.949	20.462	0.8	0.939	0.245	Tax_Id=9606 Gene_Symbol=MCAM Isoform 1 of Cell surface glycoprotein M	GATLALTQVTPQDER
751084910	534.285	20.45	-29.7	0.366	0.257	Tax_Id=9606 Gene_Symbol=MCAM Isoform 1 of Cell surface glycoprotein M	GATLALTQVTPQDER
751084911	457.244	17.534	-6	0.535	0.055		
751084912	457.746	17.547	-8.6	0.632	0.075		
751084913	681.348	16.205	-8.4	0.521	0.035		
751084914	1300.114	25.404	-0.8	0.88	0.392		
751084915	1300.614	25.427	7	0.257	0.777		
751084916	1301.115	25.484	-0.7	0.908	0.53		
751084917	736.856	25.662	-3.8	0.238	0.202		
751084918	737.349	25.613	-10	0.096	0.186		
751084919	1027.497	43.459	-2.7	0.783	0.765	Tax_Id=9606 Gene_Symbol=CLSTN1 Isoform 2 of Calsyntenin-1 (Fragment)	VEAVDADC[160.0302]SPQFSQIC[160.0302]SYE
751084920	1027.749	43.494	0.1	0.985	0.802	Tax_Id=9606 Gene_Symbol=CLSTN1 Isoform 2 of Calsyntenin-1 (Fragment)	VEAVDADC[160.0302]SPQFSQIC[160.0302]SYE

751084921	1027.999	43.507	-1.2	0.784	0.508	Tax_Id=9606 Gene_Symbol=CLSTN1 Isoform 2 of Calsyntenin-1 (Fragment)	VEAVDADC[160.0302]SPQFSQIC[160.0302]SYE
751084922	1028.25	43.53	-3.3	0.479	0.431	Tax_Id=9606 Gene_Symbol=CLSTN1 Isoform 2 of Calsyntenin-1 (Fragment)	VEAVDADC[160.0302]SPQFSQIC[160.0302]SYE
751084923	1028.498	43.42	-5.2	0.103	0.046	Tax_Id=9606 Gene_Symbol=CLSTN1 Isoform 2 of Calsyntenin-1 (Fragment)	VEAVDADC[160.0302]SPQFSQIC[160.0302]SYE
751084924	1089.856	68.169	-1	0.887	0.949	Tax_Id=9606 Gene_Symbol=PTGDS Prostaglandin-H2 D-isomerase	SPHWGSTYSVSVVETDYDQYALLYSQGSK
751084925	1090.189	68.175	-2.6	0.73	0.998	Tax_Id=9606 Gene_Symbol=PTGDS Prostaglandin-H2 D-isomerase	SPHWGSTYSVSVVETDYDQYALLYSQGSK
751084926	1090.523	68.165	-1.1	0.879	0.898	Tax_Id=9606 Gene_Symbol=PTGDS Prostaglandin-H2 D-isomerase	SPHWGSTYSVSVVETDYDQYALLYSQGSK
751084927	1090.858	68.172	-0.7	0.927	0.819	Tax_Id=9606 Gene_Symbol=PTGDS Prostaglandin-H2 D-isomerase	SPHWGSTYSVSVVETDYDQYALLYSQGSK
751084928	1091.192	68.305	-2	0.781	0.818	Tax_Id=9606 Gene_Symbol=PTGDS Prostaglandin-H2 D-isomerase	SPHWGSTYSVSVVETDYDQYALLYSQGSK
751084929	1091.524	68.462	0.7	0.915	0.718	Tax_Id=9606 Gene_Symbol=PTGDS Prostaglandin-H2 D-isomerase	SPHWGSTYSVSVVETDYDQYALLYSQGSK
751084930	473.239	24.569	2.9	0.872	0.235		
751084931	568.283	21.5	2.8	0.781	0.282		
751084932	568.618	21.449	14.3	0.347	0.821		
751084933	568.952	21.441	-14.8	0.555	0.084		
751084934	401.243	21.486	5.4	0.657	0.429		
751084935	401.575	21.37	-14	0.188	0.025		
751084936	391.24	25.407	4.7	0.49	0.713		
751084937	391.49	25.414	13.4	0.407	0.548		
751084938	391.741	25.423	40.8	0.224	0.371		
751084939	437.908	21.23	9.8	0.43	0.487		
751084940	438.243	21.238	-12.1	0.407	0.386		
751084941	563.786	20.801	4.1	0.755	0.716		
751084942	564.284	20.833	4	0.821	0.911		
751084943	445.12	74.587	-1.6	0.821	0.545		
751084944	446.119	74.597	-1.6	0.815	0.554		
751084945	602.647	24.664	-0.1	0.987	0.42	Tax_Id=9606 Gene_Symbol=APOA4 apolipoprotein A-IV precursor	LGPHAGDVEGHLSFLEK
751084946	602.982	24.659	7.3	0.26	0.79	Tax_Id=9606 Gene_Symbol=APOA4 apolipoprotein A-IV precursor	LGPHAGDVEGHLSFLEK
751084947	603.315	24.685	13	0.423	0.816	Tax_Id=9606 Gene_Symbol=APOA4 apolipoprotein A-IV precursor	LGPHAGDVEGHLSFLEK
751084948	813.368	33.096	5.7	0.474	0.247		
751084949	813.868	33.096	13.3	0.278	0.771		
751084950	814.374	33.079	-19.4	0.389	0.103		
751084951	873.396	26.969	3.1	0.849	0.709		
751084952	873.596	26.979	-0.9	0.932	0.281		
751084953	873.796	26.97	2.9	0.748	0.442		
751084954	873.997	26.98	-13.9	0.334	0.185		
751084955	874.198	26.977	-23	0.159	0.098		
751084956	874.4	26.989	-5.4	0.782	0.636		
751084957	539.266	23.596	7.7	0.163	0.784		
751084958	539.6	23.601	0.1	0.995	0.188		
751084959	539.934	23.593	3.5	0.822	0.202		
751084960	693.606	41.956	3.2	0.75	0.224		
751084961	830.449	34.697	7.3	0.368	0.825		
751084962	830.95	34.697	0.9	0.938	0.659		
751084963	543.758	14.762	2.6	0.865	0.304		
751084964	544.26	14.764	-31.8	0.056	0.004		
751084965	669.519	20.643	-6.3	0.78	0.506		
751084966	669.719	20.628	3	0.689	0.683		
751084967	669.92	20.631	-6.3	0.544	0.132		
751084968	670.12	20.633	-7	0.591	0.253		
751084969	368.709	16.739	-1.5	0.937	0.652		
751084970	693.634	36.943	4.5	0.58	0.812		
751084971	734.866	26.656	-2.3	0.692	0.166		
751084972	672.274	28.148	11	0.19	0.722		
751084973	672.775	28.151	6.8	0.516	0.179		
751084974	586.358	17.702	-10.4	0.193	0.069		
751084975	440.258	27.995	3.3	0.868	0.308	Tax_Id=9606 Gene_Symbol=CFD Complement factor D preproprotein	RPDSLQHVLLPVLDR
751084976	440.508	27.996	-4.5	0.698	0.059	Tax_Id=9606 Gene_Symbol=CFD Complement factor D preproprotein	RPDSLQHVLLPVLDR
751084977	440.759	27.987	26.3	0.352	0.937	Tax_Id=9606 Gene_Symbol=CFD Complement factor D preproprotein	RPDSLQHVLLPVLDR
751084978	794.963	43.278	0.3	0.964	0.219		
751084979	795.465	43.265	1.9	0.765	0.199		
751084980	520.578	14.936	-11.7	0.264	0.223		
751084981	520.912	15.008	-32.2	0.032	0.069		
751084982	496.776	27.033	-29.9	0.319	0.199		
751084983	497.278	27.031	-33.1	0.078	0.081		
751084984	535.319	28.996	0.7	0.959	0.304		
751084985	535.821	28.902	-20.2	0.121	0.023		
751084986	632.567	39.683	-3.4	0.853	0.34		
751084987	632.818	39.667	10	0.636	0.47		
751084988	633.069	39.669	3.1	0.699	0.81		
751084989	633.314	39.517	5.1	0.791	0.48		
751084990	798.872	24.919	-5.4	0.619	0.169		
751084991	799.373	24.903	-3.2	0.745	0.208		
751084992	566.301	20.895	3.3	0.611	0.372		
751084993	566.802	20.877	-1	0.948	0.506		
751084994	658.958	23.056	8.8	0.623	0.348		
751084995	659.293	23.058	1.3	0.843	0.243		
751084996	659.626	23.072	0.9	0.953	0.181		
751084997	753.379	14.808	10.3	0.49	0.426		
751084998	541.268	22.083	1.8	0.909	0.252		
751084999	671.336	36.071	5.1	0.254	0.575		
751085000	671.517	36.113	-2.2	0.848	0.448		
751085001	485.893	15.427	-0.2	0.989	0.083		
751085002	486.225	15.467	4.8	0.573	0.617		
751085003	465.762	22.295	4	0.696	0.386		
751085004	466.264	22.304	5.4	0.825	0.55		
751085005	654.011	39.868	2	0.786	0.894		
751085006	654.345	39.857	-1.3	0.848	0.631		
751085007	654.68	39.857	-7.8	0.228	0.329		
751085008	589.32	13.899	8.9	0.397	0.343		
751085009	590.323	13.903	12.9	0.488	0.672		
751085010	444.227	14.77	5.1	0.735	0.892	Tax_Id=9606 Gene_Symbol=CFHR2 Isoform Long of Complement factor H-r	INHGILYDEEK
751085011	444.562	14.922	9.8	0.62	0.479	Tax_Id=9606 Gene_Symbol=CFHR2 Isoform Long of Complement factor H-r	INHGILYDEEK
751085012	796.424	42.764	0.2	0.989	0.234		
751085013	796.673	42.735	4.1	0.547	0.514		

751085014	796.924	42.744	4.8	0.487	0.629		
751085015	797.175	42.76	-6	0.71	0.903		
751085016	984.478	24.219	4.1	0.415	0.763		
751085017	985.481	24.12	0	0.998	0.879		
751085018	485.086	27.319	-5.4	0.651	0.132		
751085019	485.253	27.333	-6.9	0.705	0.197		
751085020	485.421	27.325	-3.6	0.815	0.218		
751085021	485.588	27.319	-22	0.168	0.019		
751085022	554.394	22.548	2	0.89	0.19		
751085023	879.539	66.085	-0.1	0.987	0.268		
751085024	880.544	66.087	-1.3	0.86	0.429		
751085025	1010.545	43.911	-4	0.762	0.715		
751085026	1010.877	43.909	8.9	0.451	0.507		
751085027	1011.213	43.882	2.5	0.612	0.571		
751085028	1011.551	43.915	3.3	0.832	0.774		
751085029	540.329	17.505	-5.2	0.593	0.13		
751085030	540.83	17.498	-6.6	0.441	0.102		
751085031	555.334	23.097	-1	0.919	0.202		
751085032	555.836	23.112	9.9	0.607	0.6		
751085033	452.726	31.784	6.7	0.246	0.812		
751085034	479.25	14.482	3.4	0.736	0.398	Tax_Id=9606 Gene_Symbol=APLP2 Isoform 1 of Amyloid-like protein 2	HYQHVLAVDPEK
751085035	479.584	14.42	10.3	0.523	0.493	Tax_Id=9606 Gene_Symbol=APLP2 Isoform 1 of Amyloid-like protein 2	HYQHVLAVDPEK
751085036	479.919	14.423	14.6	0.455	0.492	Tax_Id=9606 Gene_Symbol=APLP2 Isoform 1 of Amyloid-like protein 2	HYQHVLAVDPEK
751085037	525.76	15.644	7.2	0.478	0.501		
751085038	598.28	18.513	-1.2	0.831	0.219		
751085039	598.783	18.369	-24.8	0.101	0.074		
751085040	841.38	18.606	-57.6	0	0	Tax_Id=9606 Gene_Symbol=SCG3 Secretogranin-3	EANNYEEDPNKPTSWTENQAGK
751085041	841.713	18.612	-22.8	0.25	0.019	Tax_Id=9606 Gene_Symbol=SCG3 Secretogranin-3	EANNYEEDPNKPTSWTENQAGK
751085042	842.047	18.623	-48.2	0.029	0.034	Tax_Id=9606 Gene_Symbol=SCG3 Secretogranin-3	EANNYEEDPNKPTSWTENQAGK
751085043	566.278	24.47	-2.8	0.741	0.058		
751085044	864.452	44.889	-2.1	0.619	0.188		
751085045	1052.966	22.637	-0.9	0.944	0.632		
751085046	1053.469	22.633	-1.4	0.881	0.379		
751085047	1053.968	22.631	-19.3	0.349	0.106		
751085048	543.526	24.001	5.5	0.245	0.888	Tax_Id=9606 Gene_Symbol=CP Ceruloplasmin	LISVDTEHSNIYLQNGPDR
751085049	543.776	23.815	18.4	0.09	0.683	Tax_Id=9606 Gene_Symbol=CP Ceruloplasmin	LISVDTEHSNIYLQNGPDR
751085050	544.027	23.962	1.7	0.823	0.539	Tax_Id=9606 Gene_Symbol=CP Ceruloplasmin	LISVDTEHSNIYLQNGPDR
751085051	414.764	15.096	10.1	0.329	0.713		
751085052	587.33	18.123	16.6	0.21	0.493		
751085053	587.83	18.136	-46.1	0.002	0.004		
751085054	559.624	26.612	-1.1	0.933	0.229		
751085055	559.958	26.609	1	0.945	0.329		
751085056	560.291	26.619	7.2	0.772	0.84		
751085057	649.912	37.115	-19.2	0.157	0.022		
751085058	650.414	37.118	-20.9	0.205	0.027		
751085059	902.437	38.781	5.2	0.53	0.671		
751085060	902.683	38.785	6	0.38	0.521		
751085061	902.939	38.841	1.1	0.848	0.278		
751085062	903.185	38.779	4.2	0.634	0.366		
751085063	903.438	38.779	2.3	0.764	0.369		
751085064	685.721	26.86	4.3	0.492	0.584		
751085065	685.922	26.859	41.6	0.088	0.551		
751085066	686.123	26.869	6.4	0.523	0.229		
751085067	686.322	26.783	5.7	0.314	0.872		
751085068	697.125	21.89	6.2	0.756	0.899		
751085069	697.326	21.852	11.3	0.155	0.566		
751085070	697.526	21.862	7.8	0.344	0.984		
751085071	697.727	21.892	7.6	0.596	0.496		
751085072	1350.265	40.225	4.5	0.209	0.841		
751085073	1350.597	40.237	3.9	0.232	0.672		
751085074	1350.934	40.184	4	0.258	0.695		
751085075	1351.272	40.107	1.9	0.628	0.456		
751085076	545.774	15.002	1.9	0.879	0.251		
751085077	546.276	14.989	-0.9	0.955	0.424		
751085078	590.267	25.395	5.7	0.617	0.714		
751085079	866.106	44.842	-0.7	0.885	0.285		
751085080	866.438	44.855	-1.1	0.797	0.198		
751085081	866.773	44.875	1	0.836	0.56		
751085082	867.109	44.876	-2.5	0.543	0.14		
751085083	496.759	21.227	11.5	0.505	0.224		
751085084	497.26	21.205	62.4	0.106	0.723		
751085085	838.896	21.489	-8.1	0.533	0.045		
751085086	839.392	21.339	-29	0.06	0.002		
751085087	810.997	40.976	8	0.351	0.445		
751085088	811.498	40.973	14.1	0.442	0.725		
751085089	812.003	40.982	-6.4	0.751	0.671		
751085090	708.843	20.271	2.1	0.845	0.383		
751085091	709.358	20.249	6.6	0.526	0.511		
751085092	709.862	20.273	4.7	0.833	0.986		
751085093	555.282	37.805	3.3	0.72	0.209		
751085094	555.784	37.838	2.1	0.918	0.281		
751085095	646.309	18.069	-2.5	0.691	0.382		
751085096	646.811	18.066	-10.6	0.683	0.255		
751085097	471.853	24.535	0.1	0.996	0.345		
751085098	472.054	24.539	0.7	0.942	0.525		
751085099	472.255	24.542	7.7	0.744	0.99		
751085100	1355.587	40.226	5.4	0.1	0.889		
751085101	1355.925	40.238	4.7	0.176	0.904		
751085102	1356.263	40.282	4.7	0.201	0.739		
751085103	1356.6	40.302	5.7	0.088	0.991		
751085104	1356.939	40.286	4.8	0.198	0.733		
751085105	1357.269	40.26	3.1	0.41	0.718		
751085106	545.303	20.571	4.1	0.649	0.556	Tax_Id=9606 Gene_Symbol=ITIH1 Inter-alpha-trypsin inhibitor heavy chain H	LDAQASFLPK

751085107	545.804	20.549	-13.5	0.472	0.244	Tax_Id=9606 Gene_Symbol=ITIH1 Inter-alpha-trypsin inhibitor heavy chain H	LDAQASFLPK
751085108	721.56	40.732	-0.7	0.908	0.103		
751085109	721.716	40.897	-0.6	0.955	0.157		
751085110	721.893	40.763	-3.1	0.709	0.106		
751085111	722.051	40.894	-0.8	0.918	0.194		
751085112	722.228	40.762	-27.5	0.086	0.055		
751085113	372.593	18.054	19.3	0.199	0.7		
751085114	372.793	18.056	9.1	0.499	0.958		
751085115	372.994	18.052	59.8	0.118	0.476		
751085116	647.82	22.806	6.7	0.61	0.728		
751085117	648.321	22.775	19.4	0.361	0.58		
751085118	544.336	16.066	0.5	0.969	0.1		
751085119	669.555	27.754	-20.1	0.084	0.079		
751085120	669.806	27.753	-3	0.527	0.16		
751085121	670.055	27.774	-11.7	0.361	0.564		
751085122	1254.577	36.229	1	0.754	0.705		
751085123	1254.905	36.187	-2.5	0.523	0.493		
751085124	596.665	30.798	6	0.703	0.848		
751085125	596.999	30.803	12	0.12	0.927		
751085126	942.538	16.893	-9.4	0.612	0.458		
751085127	943.537	16.953	-18.8	0.537	0.237		
751085128	666.341	17.527	-5.9	0.39	0.092	Tax_Id=9606 Gene_Symbol=CSPG5 Isoform 2 of Chondroitin sulfate proteog	EAGSAVEAEELVK
751085129	666.843	17.525	-29.2	0.121	0.328	Tax_Id=9606 Gene_Symbol=CSPG5 Isoform 2 of Chondroitin sulfate proteog	EAGSAVEAEELVK
751085130	709.279	30.584	4.8	0.822	0.349		
751085131	709.78	30.583	-5.6	0.736	0.04		
751085132	443.258	18.136	10.5	0.419	0.626		
751085133	443.765	18.132	-4.7	0.745	0.237		
751085134	474.77	23.662	9.9	0.572	0.357		
751085135	475.272	23.667	7.3	0.804	0.554		
751085136	400.213	16.746	-0.5	0.964	0.564		
751085137	400.548	16.751	6.9	0.815	0.898		
751085138	1098.487	39.685	4.6	0.192	0.513		
751085139	1098.737	39.739	3.8	0.261	0.801		
751085140	1098.988	39.727	4	0.246	0.98		
751085141	1099.238	39.705	2.6	0.484	0.714		
751085142	1099.49	39.679	3.9	0.269	0.955		
751085143	458.262	14.531	9.4	0.108	0.327		
751085144	646.86	41.601	-8.5	0.514	0.059		
751085145	647.362	41.605	-7.6	0.483	0.052		
751085146	647.864	41.608	37	0.269	0.94		
751085147	669.371	35.545	3.8	0.528	0.314		
751085148	669.704	35.558	-0.5	0.977	0.331		
751085149	670.039	35.571	9.6	0.548	0.986		
751085150	653.876	30.565	-2.5	0.858	0.873		
751085151	654.373	30.515	-18.3	0.366	0.307		
751085152	587.295	18.991	6.3	0.272	0.789		
751085153	587.797	18.981	6.9	0.581	0.58		
751085154	833.894	21.435	-22.9	0.077	0.014		
751085155	834.395	21.446	-12.6	0.332	0.239		
751085156	711.856	28.752	3	0.404	0.254		
751085157	400.72	23.818	17.2	0.369	0.705		
751085158	517.727	14.891	-0.6	0.97	0.138		
751085159	518.229	14.878	10.4	0.706	0.985		
751085160	844.143	38.919	-12.2	0.466	0.296	Tax_Id=9606 Gene_Symbol=SCRG1 Putative uncharacterized protein SCRG	DHNC[160.0302]HNLPEGVADLTQIDVNVQDHFW
751085161	844.397	38.944	6.2	0.445	0.965	Tax_Id=9606 Gene_Symbol=SCRG1 Putative uncharacterized protein SCRG	DHNC[160.0302]HNLPEGVADLTQIDVNVQDHFW
751085162	844.647	38.94	18.7	0.229	0.952	Tax_Id=9606 Gene_Symbol=SCRG1 Putative uncharacterized protein SCRG	DHNC[160.0302]HNLPEGVADLTQIDVNVQDHFW
751085163	844.897	38.924	-3.1	0.828	0.166	Tax_Id=9606 Gene_Symbol=SCRG1 Putative uncharacterized protein SCRG	DHNC[160.0302]HNLPEGVADLTQIDVNVQDHFW
751085164	693.873	47.309	5.9	0.577	0.342		
751085165	694.375	47.314	4	0.536	0.409		
751085166	636.822	16.686	-12.5	0.35	0.034		
751085167	637.324	16.678	-16.2	0.453	0.119		
751085168	1222.083	43.362	-1.7	0.737	0.206		
751085169	1222.584	43.349	-2.2	0.662	0.109		
751085170	1223.085	43.356	-1.7	0.789	0.289		
751085171	1223.583	43.201	-2.1	0.801	0.487		
751085172	433.22	27.864	8.6	0.574	0.159		
751085173	591.784	15.028	15.5	0.586	0.99		
751085174	692.365	15.851	1.3	0.854	0.345		
751085175	602.81	20.99	-1.8	0.84	0.175		
751085176	556.803	16.971	-18.5	0.194	0.191		
751085177	557.305	16.971	-36.9	0.135	0.228		
751085178	838.71	21.729	-14.5	0.401	0.271		
751085179	839.044	21.742	-4.7	0.654	0.702		
751085180	839.379	21.844	-6.9	0.717	0.334		
751085181	385.264	17.691	19.9	0.197	0.942		
751085182	385.766	17.711	18.8	0.649	0.925		
751085183	746.311	19.65	-5.6	0.793	0.431		
751085184	746.813	19.659	-13.2	0.441	0.321		
751085185	1164.05	36.73	6.8	0.272	0.785		
751085186	1164.547	36.765	8.4	0.173	0.799		
751085187	1165.049	36.647	2.7	0.648	0.774		
751085188	529.335	12.102	2.3	0.88	0.167		
751085189	530.339	12.106	6.8	0.821	0.822		
751085190	718.851	26.053	-9.7	0.056	0.058		
751085191	1037.761	37.568	1.7	0.759	0.488		
751085192	1038.097	37.637	-0.4	0.907	0.116		
751085193	1038.43	37.561	2.6	0.487	0.57		
751085194	1038.765	37.597	-10.1	0.111	0.089		
751085195	862.722	20.684	-1.6	0.933	0.442		
751085196	863.056	20.684	1.1	0.94	0.206		
751085197	863.399	20.763	4.7	0.785	0.365		
751085198	378.222	17.333	-2.7	0.765	0.189		
751085199	378.556	17.352	-14.8	0.355	0.064		

751085200	967.941	40.493	0.4	0.922	0.542		
751085201	447.246	13.213	13.3	0.503	0.539		
751085202	448.249	13.194	19.7	0.227	0.682		
751085203	776.41	43.507	1.5	0.809	0.742		
751085204	776.656	43.508	1.7	0.731	0.453		
751085205	776.908	43.525	-1.3	0.794	0.181		
751085206	777.159	43.512	-1.8	0.842	0.11		
751085207	453.864	17.242	6.9	0.723	0.11		
751085208	454.198	17.27	1.7	0.91	0.402		
751085209	945.831	43.967	2.9	0.667	0.354		
751085210	946.033	43.954	4.4	0.486	0.273		
751085211	946.232	43.961	2.6	0.765	0.082		
751085212	946.434	43.963	-1	0.897	0.068		
751085213	515.932	22.848	1.2	0.932	0.391		
751085214	1017.572	29.825	-6.5	0.64	0.113		
751085215	1018.576	29.852	-9.9	0.692	0.308		
751085216	730.364	39.433	7.5	0.568	0.561		
751085217	730.863	39.226	1.2	0.868	0.234		
751085218	558.796	15.533	0.5	0.968	0.117		
751085219	522.771	27.69	10.4	0.55	0.41		
751085220	522.937	27.694	5.6	0.733	0.216		
751085221	523.105	27.694	7.5	0.684	0.167		
751085222	523.276	27.703	-1.2	0.933	0.093		
751085223	523.439	27.68	27.7	0.428	0.473		
751085224	653.263	66.115	-11.8	0.296	0.807		
751085225	565.332	33.162	26.8	0.16	0.933	Tax_Id=9606 Gene_Symbol=HPX Hemopexin	RLWWLDLK
751085226	565.834	33.181	37.2	0.206	0.949	Tax_Id=9606 Gene_Symbol=HPX Hemopexin	RLWWLDLK
751085227	438.716	16.132	6	0.764	0.617		
751085228	567.308	20.859	5	0.662	0.346		
751085229	567.81	20.86	13.9	0.575	0.624		
751085230	643.95	25.639	-21.5	0.2	0.322	Tax_Id=9606 Gene_Symbol=NELL2 Protein kinase C-binding protein NELL2	NGHIC[160.0302]C[160.0302]SVDPQC[160.0302
751085231	644.284	25.626	-9	0.13	0.03	Tax_Id=9606 Gene_Symbol=NELL2 Protein kinase C-binding protein NELL2	NGHIC[160.0302]C[160.0302]SVDPQC[160.0302
751085232	644.618	25.62	-45.2	0	0.001	Tax_Id=9606 Gene_Symbol=NELL2 Protein kinase C-binding protein NELL2	NGHIC[160.0302]C[160.0302]SVDPQC[160.0302
751085233	813.348	16.209	6.1	0.627	0.211		
751085234	1122.84	29.043	-10.4	0.603	0.39		
751085235	1123.17	29.046	-0.9	0.876	0.119		
751085236	1123.504	29.03	-16.6	0.123	0.075		
751085237	1123.838	29.055	-9.9	0.559	0.928		
751085238	491.291	25.905	-30.8	0.193	0.179	Tax_Id=9606 Gene_Symbol=LSAMP Limbic system-associated membrane p	TSQVYLIVQVPPK
751085239	491.625	25.835	2.5	0.801	0.325	Tax_Id=9606 Gene_Symbol=LSAMP Limbic system-associated membrane p	TSQVYLIVQVPPK
751085240	550.682	28.626	-16.8	0.191	0.031		
751085241	550.883	28.638	9.2	0.702	0.828		
751085242	551.084	28.637	-24.1	0.086	0.029		
751085243	463.567	14.469	-26.6	0.08	0.212		
751085244	463.901	14.494	-19.7	0.286	0.303		
751085245	595.024	15.793	-9.4	0.585	0.402		
751085246	595.275	15.788	-12.2	0.197	0.051		
751085247	595.526	15.789	-22.7	0.086	0.022		
751085248	595.775	15.778	-10	0.711	0.351		
751085249	636.984	28.252	-0.5	0.948	0.308		
751085250	725.847	20.273	2.1	0.849	0.169		
751085251	726.349	20.29	0.1	0.988	0.244		
751085252	544.299	17.706	2.7	0.783	0.684		
751085253	544.798	17.706	-8.2	0.765	0.856		
751085254	471.773	14.215	42.5	0.296	0.709		
751085255	472.271	14.205	1.5	0.911	0.281		
751085256	1258.557	36.172	1.4	0.709	0.925		
751085257	1258.891	36.158	0.6	0.865	0.672		
751085258	1259.234	36.186	-0.3	0.94	0.521		
751085259	1259.576	36.31	-1.1	0.805	0.676		
751085260	808.396	23.575	-2.1	0.927	0.549		
751085261	808.898	23.578	-8.7	0.644	0.517		
751085262	809.403	23.578	17.3	0.426	0.817		
751085263	436.249	16.913	2.6	0.902	0.518		
751085264	531.76	31.221	-25.2	0.41	0.363		
751085265	683.285	19.809	9.5	0.655	0.412		
751085266	683.616	19.807	10	0.609	0.382		
751085267	856.441	26.417	-2.7	0.904	0.281	Tax_Id=9606 Gene_Symbol=CNDP1 Beta-Ala-His dipeptidase	EWVAIESDSVQPVPR
751085268	856.949	26.215	-2	0.899	0.144	Tax_Id=9606 Gene_Symbol=CNDP1 Beta-Ala-His dipeptidase	EWVAIESDSVQPVPR
751085269	480.216	20.23	12.7	0.262	0.78		
751085270	480.55	20.246	15.3	0.35	0.395		
751085271	547.774	18.459	-14.7	0.328	0.27		
751085272	954.02	33.941	8.1	0.629	0.935		
751085273	954.513	33.912	5.1	0.29	0.473		
751085274	955.006	33.93	1.4	0.735	0.258		
751085275	512.346	17.903	5.5	0.685	0.255		
751085276	584.768	19.851	-5	0.313	0.074		
751085277	585.018	19.826	-2.1	0.655	0.392		
751085278	585.269	19.882	-1.5	0.749	0.251		
751085279	606.817	20.436	-0.4	0.961	0.337		
751085280	741.866	39.382	-0.7	0.956	0.255		
751085281	742.114	39.377	0.8	0.938	0.35		
751085282	742.368	39.414	1.9	0.783	0.569		
751085283	742.618	39.399	1	0.941	0.721		
751085284	670.659	23.378	1.1	0.882	0.689		
751085285	670.994	23.373	-4	0.649	0.426		
751085286	1095.469	23.454	-3.5	0.686	0.136		
751085287	613.352	27.915	2.4	0.809	0.798		
751085288	613.686	27.889	0	0.995	0.96		
751085289	614.02	27.831	1.5	0.951	0.894		
751085290	1023.447	33.927	11.5	0.086	0.207		
751085291	1024.45	33.904	15.1	0.335	0.895		
751085292	546.247	20.263	-5.7	0.719	0.054		

751085293	546.749	20.224	13.7	0.675	0.719		
751085294	485.773	24.135	22.6	0.288	0.664		
751085295	596.767	26.588	-17.8	0.506	0.279		
751085296	596.933	26.574	7	0.741	0.458		
751085297	597.1	26.588	29.1	0.11	0.716		
751085298	597.268	26.582	3.2	0.877	0.571		
751085299	597.434	26.582	-4.5	0.886	0.884		
751085300	476.895	17.694	0.4	0.967	0.492		
751085301	477.229	17.705	-12.4	0.344	0.146		
751085302	487.314	16.674	2.7	0.848	0.377		
751085303	637.771	17.643	7.8	0.683	0.348		
751085304	626.786	41.364	5.6	0.684	0.15		
751085305	627.289	41.358	6.2	0.622	0.286		
751085306	473.926	20.066	21.4	0.07	0.352		
751085307	474.26	20.147	6.6	0.488	0.485		
751085308	808.079	38.98	10.3	0.186	0.872		
751085309	808.404	39.021	7.8	0.223	0.659		
751085310	808.751	38.975	6.9	0.579	0.94		
751085311	703.336	41.306	0.6	0.889	0.647	Tax_Id=9606 Gene_Symbol=CNTN1 Isoform 1 of Contactin-1	EYHYGNNFGYIVAFKPFDGEEWK
751085312	703.583	41.346	2.7	0.556	0.498	Tax_Id=9606 Gene_Symbol=CNTN1 Isoform 1 of Contactin-1	EYHYGNNFGYIVAFKPFDGEEWK
751085313	703.834	41.348	1	0.834	0.241	Tax_Id=9606 Gene_Symbol=CNTN1 Isoform 1 of Contactin-1	EYHYGNNFGYIVAFKPFDGEEWK
751085314	704.085	41.355	3	0.864	0.938	Tax_Id=9606 Gene_Symbol=CNTN1 Isoform 1 of Contactin-1	EYHYGNNFGYIVAFKPFDGEEWK
751085315	759.302	23.984	-8.4	0.067	0.007		
751085316	759.803	23.999	-16.5	0.077	0.023		
751085317	904.447	31.848	1.3	0.915	0.591		
751085318	653.658	32.132	-1.5	0.714	0.257		
751085319	958.033	38.598	-1.6	0.764	0.139	Tax_Id=9606 Gene_Symbol=ATP6AP1 V-type proton ATPase subunit S1	EVLTGNDEVIGQVLSTLK
751085320	958.513	38.532	-2.4	0.709	0.169	Tax_Id=9606 Gene_Symbol=ATP6AP1 V-type proton ATPase subunit S1	EVLTGNDEVIGQVLSTLK
751085321	959.035	38.608	-4.5	0.736	0.18	Tax_Id=9606 Gene_Symbol=ATP6AP1 V-type proton ATPase subunit S1	EVLTGNDEVIGQVLSTLK
751085322	941.697	39.205	1.8	0.89	0.626	Tax_Id=9606 Gene_Symbol=FBLN1 Isoform C of Fibulin-1	SQETGDLDVGGLQETDKIIEVEEEQEDPYLNDR
751085323	941.948	39.206	9.1	0.06	0.444	Tax_Id=9606 Gene_Symbol=FBLN1 Isoform C of Fibulin-1	SQETGDLDVGGLQETDKIIEVEEEQEDPYLNDR
751085324	942.199	39.207	10.6	0.053	0.179	Tax_Id=9606 Gene_Symbol=FBLN1 Isoform C of Fibulin-1	SQETGDLDVGGLQETDKIIEVEEEQEDPYLNDR
751085325	942.449	39.223	12.6	0.046	0.156	Tax_Id=9606 Gene_Symbol=FBLN1 Isoform C of Fibulin-1	SQETGDLDVGGLQETDKIIEVEEEQEDPYLNDR
751085326	653.656	26.675	-4.4	0.404	0.055		
751085327	653.992	26.668	-9.3	0.476	0.535		
751085328	830.946	45.746	8	0.117	0.931		
751085329	831.445	45.748	9.8	0.128	0.81		
751085330	831.948	45.8	9.4	0.071	0.773		
751085331	493.78	14.828	-0.1	0.996	0.21		
751085332	494.283	14.829	-24.2	0.287	0.135		
751085333	926.174	40.538	-2.6	0.552	0.405		
751085334	926.424	40.537	1.2	0.708	0.603		
751085335	926.674	40.57	1.1	0.762	0.8		
751085336	926.925	40.552	0.1	0.986	0.429		
751085337	927.174	40.477	-21	0.058	0.069		
751085338	702.673	19.77	-26	0.005	0.014		
751085339	543.907	5.997	8	0.475	0.825		
751085340	544.908	5.992	1.5	0.825	0.994		
751085341	545.906	5.964	17.9	0.571	0.624		
751085342	939.917	36.165	-1.2	0.9	0.633		
751085343	940.167	36.168	0.2	0.956	0.477		
751085344	940.417	36.183	-0.9	0.814	0.46		
751085345	940.669	36.179	2.1	0.779	0.85		
751085346	940.927	36.161	-8.1	0.59	0.866		
751085347	444.586	14.847	5.9	0.703	0.206		
751085348	444.921	14.85	-11.9	0.664	0.2		
751085349	602.809	16.242	1.4	0.943	0.365		
751085350	613.559	69.146	3.6	0.618	0.581		
751085351	613.81	69.147	3.4	0.636	0.556		
751085352	614.06	69.148	3	0.663	0.597		
751085353	614.311	69.183	2.7	0.687	0.609		
751085354	614.562	69.172	4.7	0.634	0.678		
751085355	986.481	38.321	10.3	0.25	0.923		
751085356	986.983	38.331	15.3	0.257	0.873		
751085357	987.485	38.379	24.6	0.244	0.329		
751085358	677.836	37.771	9.7	0.12	0.651		
751085359	588.313	38.639	0.4	0.926	0.283		
751085360	588.648	38.653	1.2	0.743	0.44		
751085361	747.723	29.104	-2.3	0.509	0.965		
751085362	948.476	20.107	-7.2	0.75	0.535		
751085363	349.52	19.253	2.9	0.883	0.45		
751085364	349.854	19.237	-1.8	0.898	0.122		
751085365	429.231	23.939	10.8	0.531	0.375		
751085366	591.969	16.514	-5.1	0.775	0.553		
751085367	592.304	16.521	5.7	0.763	0.676		
751085368	592.637	16.501	11.3	0.721	0.874		
751085369	445.266	12.783	16.2	0.449	0.811		
751085370	446.27	12.807	39.2	0.158	0.347		
751085371	803.399	40.405	-5.8	0.625	0.385		
751085372	803.735	40.38	-7.8	0.472	0.217		
751085373	804.069	40.389	-2.1	0.895	0.992		
751085374	803.357	35.38	-19.1	0.183	0.086		
751085375	803.612	35.386	0.4	0.938	0.265		
751085376	803.861	35.389	1.8	0.756	0.305		
751085377	804.108	35.292	-21.1	0.152	0.185		
751085378	579.293	38.67	13.6	0.139	0.965		
751085379	579.795	38.657	9.9	0.685	0.694		
751085380	1053.739	66.062	-10.9	0.268	0.87		
751085381	1054.743	66.065	-1.1	0.91	0.386		
751085382	1323.675	27.466	41.8	0.116	0.254		
751085383	1324.677	27.47	19.7	0.428	0.874		
751085384	543.761	21.075	2.8	0.794	0.537		
751085385	530.781	15.713	-0.3	0.98	0.177		

751085386	432.281	14.575	-19.6	0.193	0.759		
751085387	529.818	21.468	25.3	0.24	0.914		
751085388	530.32	21.468	-16.9	0.463	0.072		
751085389	349.871	16.587	-23.3	0.142	0.062		
751085390	350.205	16.597	-59.5	0.007	0.014		
751085391	596.065	33.714	-13.3	0.494	0.131		
751085392	629.989	39.404	19.9	0.185	0.839		
751085393	630.323	39.394	4.4	0.701	0.599		
751085394	585.745	14.893	16.4	0.367	0.456		
751085395	483.568	16.884	-31.7	0.145	0.151		
751085396	483.902	16.828	-42.7	0.032	0.1		
751085397	612.807	22.464	13.1	0.316	0.872		
751085398	384.875	15.068	-1.6	0.927	0.307		
751085399	781.724	38.442	6.7	0.148	0.971		
751085400	1324.265	40.119	4.7	0.294	0.929		
751085401	1324.598	40.11	3.5	0.375	0.636		
751085402	448.714	20.521	-8.3	0.567	0.11		
751085403	381.48	16.117	7.2	0.542	0.657		
751085404	486.942	23.289	2	0.751	0.614		
751085405	487.275	23.274	13.7	0.468	0.843		
751085406	605.791	16.21	4.4	0.681	0.629		
751085407	974.977	44.44	1.9	0.857	0.47		
751085408	975.229	44.431	4.4	0.675	0.691		
751085409	975.475	44.423	0.7	0.916	0.527		
751085410	876.421	22.595	-11.1	0.611	0.372		
751085411	876.757	22.503	4.8	0.69	0.978		
751085412	877.091	22.512	-9.4	0.667	0.87		
751085413	602.475	34.972	-14.6	0.525	0.222		
751085414	602.675	34.984	-14	0.351	0.07		
751085415	602.876	34.985	-14.4	0.522	0.298		
751085416	603.076	34.977	-19	0.381	0.317		
751085417	579.818	37.264	3.7	0.794	0.443		
751085418	580.32	37.358	2.4	0.887	0.402		
751085419	373.902	16.534	13.7	0.344	0.419		
751085420	374.237	16.55	16.9	0.614	0.374		
751085421	634.87	44.778	9.8	0.16	0.54		
751085422	612.924	21.958	-8	0.51	0.136		
751085423	613.259	21.961	5.3	0.572	0.254		
751085424	613.595	22.036	-2.8	0.704	0.114		
751085425	580.751	16.801	3.9	0.664	0.559		
751085426	889.966	39.035	3.8	0.436	0.655		
751085427	890.467	39.034	3	0.561	0.598		
751085428	890.973	39.058	21	0.185	0.25		
751085429	608.388	20.401	-5.5	0.442	0.287		
751085430	781.876	26.391	21.8	0.269	0.458		
751085431	782.377	26.355	-9.3	0.27	0.121		
751085432	602.746	46.262	16.2	0.461	0.657		
751085433	352.341	65.167	7.7	0.354	0.231		
751085434	757.368	21.83	7.6	0.759	0.849		
751085435	757.87	21.824	8.4	0.799	0.783		
751085436	758.395	21.921	10.9	0.766	0.942		
751085437	648.814	30.356	3.4	0.827	0.279		
751085438	649.064	30.376	1.3	0.942	0.165		
751085439	649.314	30.484	3.7	0.795	0.971		
751085440	421.216	14.576	1.5	0.933	0.299		
751085441	596.316	25.538	2	0.724	0.68		
751085442	596.653	25.545	0.4	0.951	0.424		
751085443	542.941	26.454	25.4	0.289	0.989		
751085444	543.275	26.349	10.2	0.603	0.583		
751085445	543.611	26.283	53	0.215	0.464		
751085446	765.708	26.503	-5.6	0.338	0.058		
751085447	413.267	5.676	1.9	0.498	0.687		
751085448	414.27	5.699	-7	0.76	0.183		
751085449	833.928	36.944	-8.7	0.319	0.151		
751085450	834.427	36.959	-3.2	0.565	0.318		
751085451	729.869	18.125	0.5	0.985	0.946		
751085452	730.372	18.128	-18.1	0.542	0.516		
751085453	615.791	22.537	20.2	0.085	0.207		
751085454	616.293	22.613	19.1	0.412	0.998		
751085455	597.333	15.793	-27.7	0.118	0.055	Tax_Id=9606 Gene_Symbol=SCG3 Secretogranin-3	TEAYLEAIRK
751085456	597.834	15.801	-39.4	0.054	0.014	Tax_Id=9606 Gene_Symbol=SCG3 Secretogranin-3	TEAYLEAIRK
751085457	588.647	37.534	5.9	0.082	0.823	Tax_Id=9606 Gene_Symbol=C1S Complement C1s subcomponent	SNALDIIFQTDLTGQK
751085458	588.982	37.676	6	0.076	0.401	Tax_Id=9606 Gene_Symbol=C1S Complement C1s subcomponent	SNALDIIFQTDLTGQK
751085459	589.313	37.764	19.2	0.332	0.762	Tax_Id=9606 Gene_Symbol=C1S Complement C1s subcomponent	SNALDIIFQTDLTGQK
751085460	423.965	21.622	7.7	0.409	0.923		
751085461	470.938	16.445	-13.6	0.331	0.049		
751085462	394.198	14.292	10.2	0.467	0.388		
751085463	550.937	15.61	-4.5	0.766	0.561		
751085464	551.27	15.612	-1.5	0.923	0.231		
751085465	777.341	26.973	-28.1	0.007	0.006		
751085466	777.588	26.942	-12.3	0.263	0.139		
751085467	777.839	26.946	-8.4	0.237	0.209		
751085468	778.088	26.926	-55.6	0.009	0.085		
751085469	408.214	13.514	5.7	0.771	0.595		
751085470	409.217	13.527	26.3	0.522	0.564		
751085471	504.774	26.774	6.1	0.619	0.334		
751085472	674.971	24.115	-17.1	0.013	0.005		
751085473	675.305	24.048	-19	0.021	0.06		
751085474	675.638	24.117	-16.9	0.485	0.815		
751085475	441.3	64.838	4.2	0.575	0.311		
751085476	1077.804	35.677	19.9	0.356	0.367		
751085477	1078.137	35.677	6.8	0.592	0.597		
751085478	1078.47	35.662	19	0.329	0.319		

751085479	1078.804	35.658	6.9	0.37	0.422		
751085480	592.876	24.431	9.1	0.404	0.388		
751085481	593.02	24.411	5.1	0.508	0.433		
751085482	593.162	24.408	6.4	0.398	0.686		
751085483	521.728	14.876	2.3	0.875	0.208		
751085484	522.231	14.797	12	0.48	0.444		
751085485	719.82	20.249	3	0.851	0.272		
751085486	720.322	20.438	-5.3	0.552	0.122		
751085487	1294.259	40.538	1.5	0.688	0.409		
751085488	1294.594	40.639	2.2	0.587	0.623		
751085489	1294.927	40.631	0.8	0.83	0.323		
751085490	1295.267	40.595	-2.2	0.568	0.201		
751085491	613.357	14.289	38.2	0.07	0.907		
751085492	438.899	16.011	20	0.215	0.701		
751085493	570.302	20.911	5.5	0.551	0.857		
751085494	570.804	20.911	3.6	0.86	0.634		
751085495	548.837	22.874	7.3	0.678	0.549		
751085496	527.253	22.715	24	0.325	0.869		
751085497	626.994	22.247	4.4	0.667	0.294		
751085498	693.316	17.737	0	0.999	0.627		
751085499	693.796	17.623	-12.4	0.176	0.081		
751085500	638.332	42.188	4.3	0.52	0.536		
751085501	638.834	42.241	1.1	0.938	0.322		
751085502	980.782	41.547	-11.1	0.226	0.065		
751085503	981.115	41.538	0.9	0.885	0.501		
751085504	981.45	41.536	0.6	0.95	0.164		
751085505	981.788	41.553	-11.6	0.281	0.084		
751085506	451.226	25.935	7.1	0.727	0.462		
751085507	451.728	26.001	-3.4	0.879	0.227		
751085508	637.379	39.24	13	0.099	0.792		
751085509	637.88	39.236	10.4	0.235	0.497		
751085510	817.639	68.49	-10.6	0.28	0.485	Tax_Id=9606 Gene_Symbol=PTGDS Prostaglandin-H2 D-isomerase	SPHWGSTYSVSVVETDYDQYALLYSQGSK
751085511	817.889	68.525	-43.4	0.068	0.204	Tax_Id=9606 Gene_Symbol=PTGDS Prostaglandin-H2 D-isomerase	SPHWGSTYSVSVVETDYDQYALLYSQGSK
751085512	818.142	68.455	-1.5	0.831	0.947	Tax_Id=9606 Gene_Symbol=PTGDS Prostaglandin-H2 D-isomerase	SPHWGSTYSVSVVETDYDQYALLYSQGSK
751085513	818.391	68.318	-0.1	0.991	0.782	Tax_Id=9606 Gene_Symbol=PTGDS Prostaglandin-H2 D-isomerase	SPHWGSTYSVSVVETDYDQYALLYSQGSK
751085514	818.641	68.524	3.7	0.736	0.655	Tax_Id=9606 Gene_Symbol=PTGDS Prostaglandin-H2 D-isomerase	SPHWGSTYSVSVVETDYDQYALLYSQGSK
751085515	599.293	26.823	-12.2	0.203	0.296		
751085516	599.544	26.865	-1	0.908	0.383		
751085517	599.795	26.854	-2	0.881	0.563		
751085518	452.848	15.339	14.8	0.342	0.378		
751085519	453.183	15.33	11.9	0.684	0.69		
751085520	329.15	8.826	4	0.768	0.235		
751085521	330.153	8.829	22.7	0.426	0.662		
751085522	662.315	20.034	-10.9	0.416	0.961		
751085523	518.965	21.461	-3	0.83	0.347		
751085524	519.299	21.455	-30.3	0.029	0.048		
751085525	561.586	21.42	14	0.458	0.667		
751085526	561.92	21.406	-0.4	0.97	0.97		
751085527	671.823	22.499	-0.4	0.974	0.666		
751085528	672.324	22.471	-4.2	0.803	0.918		
751085529	488.272	10.76	5.5	0.692	0.344		
751085530	489.276	10.762	18.6	0.437	0.758		
751085531	787.387	35.388	-24.8	0.112	0.15		
751085532	787.722	35.405	-1.3	0.94	0.995		
751085533	788.054	35.432	-20.6	0.211	0.44		
751085534	377.587	15.445	14.5	0.511	0.612		
751085535	377.788	15.442	-7.8	0.606	0.137		
751085536	377.989	15.434	-5.4	0.902	0.787		
751085537	432.863	42.213	4.2	0.817	0.61		
751085538	433.197	42.215	11.4	0.677	0.728		
751085539	575.307	20.87	8.8	0.625	0.619		
751085540	637.326	68.792	4.2	0.55	0.712		
751085541	637.66	68.789	2.8	0.695	0.773		
751085542	637.994	68.835	4.3	0.594	0.588		
751085543	638.329	68.871	2.7	0.798	0.386		
751085544	546.322	39.216	-0.9	0.88	0.777		
751085545	632.845	27.595	4.6	0.781	0.852		
751085546	633.346	27.613	-14.3	0.397	0.281		
751085547	1111.246	46.529	4.4	0.266	0.868		
751085548	1111.587	46.467	4.8	0.222	0.878		
751085549	466.735	21.83	4.9	0.659	0.42		
751085550	466.986	21.812	7	0.61	0.482		
751085551	467.237	21.848	-11.6	0.488	0.221		
751085552	1064.143	13.78	-18	0.297	0.475		
751085553	1064.478	13.778	-10.9	0.35	0.287		
751085554	1064.812	13.778	-19.3	0.24	0.239		
751085555	1065.146	13.781	-7.5	0.709	0.78		
751085556	1065.48	13.787	-1.2	0.972	0.758		
751085557	449.74	25.977	12.5	0.425	0.32		
751085558	409.909	16.108	7.9	0.636	0.323		
751085559	410.243	16.14	21.9	0.508	0.942		
751085560	1012.946	40.258	1.1	0.76	0.426		
751085561	1013.195	40.248	2.1	0.718	0.532		
751085562	1013.448	40.269	2.9	0.381	0.794		
751085563	915.916	40.54	-19.3	0.011	0.043		
751085564	916.167	40.601	-3.9	0.324	0.198		
751085565	916.418	40.566	1.4	0.743	0.998		
751085566	916.669	40.584	-1.6	0.732	0.376		
751085567	494.074	23.612	2.3	0.896	0.162		
751085568	494.408	23.652	3.3	0.856	0.047		
751085569	561.599	17.61	-14.7	0.332	0.237		
751085570	561.933	17.499	-13	0.224	0.13		
751085571	493.507	23.507	-12.4	0.314	0.065		

751085572	493.758	23.572	5.3	0.781	0.597		
751085573	494.008	23.539	-18.1	0.318	0.034		
751085574	479.217	13.814	11.2	0.474	0.529		
751085575	479.551	13.837	12.2	0.516	0.669		
751085576	479.886	13.85	30.2	0.354	0.576		
751085577	727.89	38.682	-7.6	0.522	0.361		
751085578	728.386	38.828	6.7	0.55	0.579		
751085579	681.662	25.425	0.4	0.968	0.599		
751085580	681.994	25.418	-5	0.248	0.11		
751085581	381.731	16.118	9.1	0.657	0.706		
751085582	798.398	19.395	-4.7	0.396	0.042		
751085583	798.901	19.388	-0.9	0.861	0.707		
751085584	1021.99	25.467	-18.5	0.162	0.048		
751085585	1022.993	25.448	-34.6	0.123	0.044		
751085586	553.782	16.039	-39.8	0.076	0.226		
751085587	640.351	45.55	-2.5	0.816	0.292		
751085588	640.681	45.536	-9.9	0.497	0.371		
751085589	641.016	45.538	-28.2	0.322	0.501		
751085590	588.982	38.871	-0.3	0.956	0.323		
751085591	1351.61	40.104	2.3	0.582	0.792		
751085592	463.752	16.941	-28	0.165	0.09		
751085593	531.778	15.958	-3	0.841	0.273		
751085594	628.344	40.409	18.2	0.359	0.721	Tax_Id=9606 Gene_Symbol=GSTM4 Isoform 1 of Glutathione S-transferase	LGLDFPNLPYLIDGAHK
751085595	628.679	40.391	7.5	0.548	0.636	Tax_Id=9606 Gene_Symbol=GSTM4 Isoform 1 of Glutathione S-transferase	LGLDFPNLPYLIDGAHK
751085596	460.277	10.687	21.4	0.398	0.919		
751085597	461.281	10.702	79.9	0.132	0.723		
751085598	843.935	41.07	-8.8	0.282	0.127		
751085599	355.07	74.56	5.4	0.503	0.197		
751085600	966.42	40.169	3.5	0.172	0.936		
751085601	1074.806	41.625	2.2	0.653	0.82		
751085602	996.45	13.932	1.5	0.941	0.855		
751085603	997.453	13.93	-27.1	0.217	0.22		
751085604	387.187	14.15	10.1	0.655	0.917		
751085605	387.689	14.169	64.8	0.11	0.003		
751085606	1094.422	18.397	-2.9	0.867	0.725		
751085607	1094.924	18.408	-4.9	0.826	0.655		
751085608	393.881	14.08	6.7	0.762	0.854		
751085609	394.215	14.01	15.9	0.47	0.865		
751085610	464.923	24.882	0.1	0.994	0.325		
751085611	427.396	65.14	10.3	0.246	0.288		
751085612	488.899	23.888	3.5	0.893	0.734		
751085613	489.233	23.92	10.4	0.684	0.79		
751085614	502.288	10.365	8.5	0.563	0.279		
751085615	503.291	10.388	35.8	0.444	0.756		
751085616	785.891	68.433	2.2	0.748	0.524		
751085617	786.142	68.364	1.8	0.794	0.461		
751085618	786.392	68.384	2.3	0.741	0.623		
751085619	786.644	68.427	3.2	0.638	0.337		
751085620	786.894	68.488	-1.4	0.942	0.559		
751085621	717.38	13.649	7.4	0.621	0.31		
751085622	718.38	13.737	-4.9	0.868	0.288		
751085623	399.261	12.106	-6.4	0.587	0.058		
751085624	400.264	12.129	-14.1	0.694	0.173		
751085625	688.791	13.585	-4.5	0.839	0.9		
751085626	397.532	13.924	2.1	0.91	0.674	Tax_Id=9606 Gene_Symbol=NELL2 Protein kinase C-binding protein NELL2	YLELESSGHR
751085627	397.867	13.954	-51	0.015	0.039	Tax_Id=9606 Gene_Symbol=NELL2 Protein kinase C-binding protein NELL2	YLELESSGHR
751085628	450.214	12.969	-1.7	0.867	0.204		
751085629	451.217	12.967	-14.4	0.619	0.244		
751085630	421.216	13.681	13.7	0.319	0.517		
751085631	519.139	74.6	-2.8	0.659	0.687		
751085632	416.182	12.576	-6.4	0.756	0.3		
751085633	614.286	12.214	0.5	0.97	0.257		
751085634	368.161	11.898	12.4	0.508	0.286		
751085635	410.193	8.596	17.5	0.464	0.23		
751085636	955.485	68.848	1	0.876	0.904	Tax_Id=9606 Gene_Symbol=PTGDS Prostaglandin-H2 D-isomerase	AQGFTEDTIVFLPQTDK
751085637	955.986	68.846	1.3	0.865	0.66	Tax_Id=9606 Gene_Symbol=PTGDS Prostaglandin-H2 D-isomerase	AQGFTEDTIVFLPQTDK
751085638	956.487	68.859	1.7	0.854	0.46	Tax_Id=9606 Gene_Symbol=PTGDS Prostaglandin-H2 D-isomerase	AQGFTEDTIVFLPQTDK
751085639	465.236	13.335	6.7	0.518	0.541		
751085640	477.235	12.497	-15.7	0.598	0.414		
751085641	644.825	69.372	1.7	0.791	0.711		
751085642	644.824	71.158	1.5	0.815	0.778	Tax_Id=9606 Gene_Symbol=CLU Isoform 2 of Clusterin	ELDESLQVAER
751085643	645.326	69.799	6.3	0.519	0.348	Tax_Id=9606 Gene_Symbol=CLU Isoform 2 of Clusterin	ELDESLQVAER
751085644	447.117	74.546	-1.1	0.867	0.522		
751085645	461.261	12.793	1.9	0.879	0.291		
751085646	464.817	68.383	-2.5	0.771	0.208		
751085647	465.018	68.345	-1	0.914	0.236		
751085648	465.219	68.369	-1.6	0.861	0.563		
751085649	529.891	5.868	22.7	0.489	0.416		
751085650	454.25	13.765	11.2	0.37	0.479		
751085651	454.584	13.85	-6.3	0.74	0.091		
751085652	393.298	5.686	31.8	0.169	0.688		
751085653	318.239	7.083	-11.5	0.746	0.887		
751085654	413.267	12.5	1	0.577	0.497		
751085655	414.27	12.552	-6.6	0.709	0.73		
751085656	301.04	10.264	8.5	0.499	0.865		
751085657	374.229	10.584	5.8	0.762	0.628		
751085658	465.016	70.763	-3.1	0.863	0.294		
751085659	451.256	12.962	18	0.511	0.957		
751085660	478.353	71.487	-5.6	0.408	0.806		
751085661	479.356	71.508	-8	0.624	0.868		
751085662	403.256	12.54	24.5	0.269	0.649		
751085663	369.648	6.42	18.5	0.506	0.968		
751085664	495.246	11.854	23	0.27	0.835		

751085665	340.939	13.817	47.7	0.197	0.207
751085666	543.906	70.74	-1.4	0.837	0.758
751085667	544.907	70.966	4.4	0.592	0.458
751085668	475.739	13.84	17.8	0.505	0.557
751085669	359.229	8.576	8.7	0.696	0.944
751085670	429.271	12.385	-23.2	0.133	0.018
751085671	371.316	12.912	-1.6	0.541	0.716
751085672	594.289	12.576	26.8	0.198	0.642
751085673	464.815	70.585	-4.9	0.761	0.155
751085674	645.325	71.613	2.6	0.802	0.758
751085675	483.293	7.745	20.9	0.52	0.806
751085676	593.158	74.634	-3.3	0.599	0.835
751085677	483.245	6.582	13.8	0.558	0.847
751085678	445.266	10.272	0	0.999	0.393
751085679	520.139	74.441	-12.3	0.241	0.553
751085680	536.166	5.505	14	0.421	0.537
751085681	521.135	74.45	-14.4	0.153	0.516
751085682	610.185	5.493	-16.9	0.238	0.379

Supplementary Table 5. SME1 and SME 2 AUCs in Cohort-1.

OPP	Dx	MS Data Name	Property8	Property1	File Name	Run ID	SME1 (AUC) ID#722474226	SME2 (AUC) ID#722476351
1	CTL	OPP 1-1	47	AE111	157753	34777	110,210	61,208
1	CTL	OPP 1-2	47	AE111	157802	34873	74,316	41,737
2	AD	OPP 2-1	56	DC01	157754	34778	21,802	1,315
2	AD	OPP 2-2	56	DC01	157801	34872	32,359	17,678
3	CTL	OPP 3-1	4	IB05	157755	34779	74,014	70,570
3	CTL	OPP 3-2	4	IB05	157800	34871	115,177	96,473
4	AD	OPP 4-1	62	AC070	157756	34780	77,166	30,856
4	AD	OPP 4-2	62	AC070	157799	34869	111,627	33,029
5	CTL	OPP 5-1	79	LB07	157757	34781	115,531	92,829
5	CTL	OPP 5-2	79	LB07	157798	34867	159,732	128,506
6	AD	OPP 6-1	83	AD106	157759	34783	21,504	35,333
6	AD	OPP 6-2	83	AD106	157796	34863	33,087	41,954
7	CTL	OPP 7-1	119	FF061	157760	34784	83,995	75,726
7	CTL	OPP 7-2	119	FF061	157795	34861	98,658	83,988
8	AD	OPP 8-1	146	AE087	157761	34787	51,449	37,524
8	AD	OPP 8-2	146	AE087	157794	34859	59,761	45,654
9	CTL	OPP 9-1	157	KA080	157762	34794	186,144	132,997
9	CTL	OPP 9-2	157	KA080	157793	34857	137,345	96,770
10	AD	OPP 10-1	200	KC013	157763	34801	38,695	35,310
10	AD	OPP 10-2	200	KC013	157792	34955	56,243	48,542
11	CTL	OPP 11-1	198	KF055	157765	34805	91,433	65,512
11	CTL	OPP 11-2	198	KF055	157790	34852	135,815	102,256
12	AD	OPP 12-1	56	EA070	157766	34806	22,916	22,120
12	AD	OPP 12-2	230	EA070	157789	34850	22,622	22,960
13	CTL	OPP 13-1	236	GD090	157767	34807	115,250	74,676
13	CTL	OPP 13-2	236	GD090	157788	34848	157,955	112,783
14	AD	OPP 14-1	248	BB045	157768	34808	24,199	31,317
14	AD	OPP 14-2	248	BB045	157787	34954	21,891	29,401
15	CTL	OPP 15-1	253	GA089	157769	34809	104,111	75,149
15	CTL	OPP 15-2	253	GA089	157786	34844	111,785	78,799
16	AD	OPP 16-1	269	CC090	157771	34811	16,814	20,278
16	AD	OPP 16-2	269	CC090	157784	34839	21,559	21,045
17	CTL	OPP 17-1	282	FE057	157772	35598	198,900	2,225
17	CTL	OPP 17-2	282	FE057	157783	35613	266,129	2,264
18	AD	OPP 18-1	286	IC06	157773	34813	17,501	14,939
18	AD	OPP 18-2	286	IC06	157782	34834	50,749	18,873
19	CTL	OPP 19-1	284	IE046	157774	34945	77,327	60,232
19	CTL	OPP 19-2	284	IE046	157781	34826	91,766	73,952
20	AD	OPP 20-1	302	BB080	157775	35607	30,303	2,977
20	AD	OPP 20-2	302	BB080	157780	35612	26,437	1,535

Supplementary Table 6. SME1 and SME2 AUCs in Cohort-2.

	Feature ID	751080736	751082516				
	Peak Centroid m/z	639.629	635.978				
	Peak Centroid Time	24.826	22.267	File Name	Description	Sample Name	Dx
1	Intensity [809175]	47366.461	18475.074	320173	OPTIMA =229, EPNo =11, Years =5.04	M31133	pMCI
2	Intensity [809281]	54222.992	22390.539	320175	OPTIMA =229, EPNo =13, Years =5.94	M31134	pMCI
3	Intensity [809409]	56005.656	16554.812	320177	OPTIMA =229, EPNo =3, Years =1.07	M31129	pMCI
4	Intensity [809479]	65052.219	24273.898	320179	OPTIMA =229, EPNo =5, Years =2.03	M31130	pMCI
5	Intensity [809549]	52395.516	19215.512	320181	OPTIMA =229, EPNo =1, Years =0	M31128	pMCI
6	Intensity [809693]	65127.734	11600.41	320185	OPTIMA =229, EPNo =7, Years =3.02	M31131	pMCI
7	Intensity [809768]	40193.133	19256.371	320187	OPTIMA =229, EPNo =9, Years =4.06	M31132	pMCI
8	Intensity [809869]	17918.395	8498.25	320190	OPTIMA =74, EPNo =5, Years =2.09	M31032	sCTL
9	Intensity [809938]	13761.064	8837.122	320192	OPTIMA =74, EPNo =3, Years =1	M31031	sCTL
10	Intensity [810019]	22608.303	12226.818	320194	OPTIMA =74, EPNo =1, Years =0	M31030	sCTL
11	Intensity [810211]	18004.604	18074.896	320198	OPTIMA =102, EPNo =5, Years =1.94	M31046	AD
12	Intensity [810316]	17153.541	16146.535	320200	OPTIMA =102, EPNo =1, Years =0	M31045	AD
13	Intensity [810391]	20298.779	11915.255	320202	OPTIMA =102, EPNo =7, Years =2.93	M31047	AD
14	Intensity [810753]	11597.91	10452.768	320210	OPTIMA =66, EPNo =5, Years =2.12	M31029	sCTL
15	Intensity [810874]	16416.027	15527.081	320212	OPTIMA =66, EPNo =1, Years =0	M31028	sCTL
16	Intensity [811158]	27120.297	10593.011	320215	OPTIMA =84, EPNo =1, Years =0	M31037	sCTL
17	Intensity [811407]	38481.664	11470.803	320218	OPTIMA =84, EPNo =3, Years =1.06	M31038	sCTL
18	Intensity [811648]	5727.94	4361.952	320222	OPTIMA =91, EPNo =3, Years =1.12	M31039	sCTL
19	Intensity [811864]	6538.816	4336.864	320225	OPTIMA =91, EPNo =5, Years =2.16	M31040	sCTL
20	Intensity [813481]	82353.477	15730.792	320240	OPTIMA =105, EPNo =3, Years =1.16	M31049	AD
21	Intensity [813601]	73996.484	18187.684	320242	OPTIMA =105, EPNo =7, Years =3.12	M31051	AD
22	Intensity [813732]	55878.031	20162.215	320244	OPTIMA =105, EPNo =5, Years =2.13	M31050	AD
23	Intensity [814245]	24379.41	9646.006	320252	OPTIMA =252, EPNo =1, Years =0	M31145	sCTL
24	Intensity [814369]	14400.014	4737.787	320254	OPTIMA =252, EPNo =5, Years =2.19	M31147	sCTL
25	Intensity [814471]	12369.334	6514.544	320256	OPTIMA =252, EPNo =3, Years =1.1	M31146	sCTL
26	Intensity [814816]	34831.645	18844.479	320261	OPTIMA =147, EPNo =3, Years =1.09	M31074	AD
27	Intensity [814941]	22873.555	22943.445	320263	OPTIMA =147, EPNo =1, Years =0	M31073	AD
28	Intensity [815090]	33896.258	20817.301	320265	OPTIMA =147, EPNo =5, Years =1.97	M31075	AD
29	Intensity [815228]	25562.098	17296.297	320267	OPTIMA =37, EPNo =1, Years =0	M31006	sCTL
30	Intensity [815375]	26307.348	15607.936	320269	OPTIMA =37, EPNo =3, Years =1.15	M31007	sCTL
31	Intensity [815639]	17306.492	7056.44	320273	OPTIMA =95, EPNo =3, Years =1.01	M31042	sCTL
32	Intensity [815785]	29360.604	15941.396	320275	OPTIMA =95, EPNo =1, Years =0	M31041	sCTL
33	Intensity [815916]	12387.319	8625.85	320277	OPTIMA =100, EPNo =1, Years =0	M31043	AD
34	Intensity [816134]	10668.58	5720.723	320280	OPTIMA =100, EPNo =3, Years =1.17	M31044	AD
35	Intensity [819347]	17674.662	16103.648	320310	OPTIMA =33, EPNo =5, Years =2.98	M31003	sCTL
36	Intensity [819472]	11230.998	11644.568	320312	OPTIMA =33, EPNo =7, Years =3.98	M31004	sCTL
37	Intensity [819611]	24008.521	24394.957	320314	OPTIMA =33, EPNo =3, Years =1.97	M31002	sCTL
38	Intensity [819762]	8637.624	7334.342	320316	OPTIMA =33, EPNo =9, Years =4.96	M31005	sCTL
39	Intensity [820406]	13164.601	12460.148	320326	OPTIMA =156, EPNo =3, Years =1.12	M31081	AD
40	Intensity [820551]	12935.473	7311.68	320328	OPTIMA =156, EPNo =1, Years =0	M31080	AD
41	Intensity [820683]	13383.623	4402.771	320330	OPTIMA =156, EPNo =5, Years =2.12	M31082	AD
42	Intensity [820922]	10988.572	5448.142	320333	OPTIMA =51, EPNo =3, Years =0.96	M31012	sCTL
43	Intensity [821060]	17282.932	5455.5	320335	OPTIMA =51, EPNo =1, Years =0	M31011	sCTL
44	Intensity [821185]	8928.305	11354.578	320337	OPTIMA =107, EPNo =3, Years =1.11	M31054	AD
45	Intensity [821308]	15884.217	20019.637	320339	OPTIMA =107, EPNo =1, Years =0	M31053	AD
46	Intensity [821435]	28072.789	21105.371	320341	OPTIMA =118, EPNo =1, Years =0	M31059	AD
47	Intensity [821665]	30304.48	15436.188	320345	OPTIMA =118, EPNo =3, Years =1.12	M31060	AD
48	Intensity [821791]	9390.327	16774.227	320347	OPTIMA =121, EPNo =3, Years =1.12	M31062	AD
49	Intensity [821991]	13101.674	20667.617	320350	OPTIMA =121, EPNo =1, Years =0	M31061	AD
50	Intensity [822149]	16413.719	12665.179	320353	OPTIMA =188, EPNo =1, Years =0	M31098	ODS
51	Intensity [822440]	30570.98	10351.364	320358	OPTIMA =188, EPNo =3, Years =1.04	M31099	pCTL.AD
52	Intensity [824039]	23862.412	15291.174	320373	OPTIMA =114, EPNo =7, Years =3.1	M31058	AD
53	Intensity [824299]	35379.582	15008.818	320375	OPTIMA =114, EPNo =3, Years =1.16	M31056	AD
54	Intensity [824393]	40735.52	21162.756	320376	OPTIMA =114, EPNo =1, Years =0	M31055	AD
55	Intensity [824419]	23396.314	9349.729	320378	OPTIMA =114, EPNo =5, Years =2.14	M31057	AD
56	Intensity [824424]	15709.824	11704.861	320379	OPTIMA =178, EPNo =7, Years =3.14	M31095	ODS
57	Intensity [824434]	21634.543	17167.205	320381	OPTIMA =178, EPNo =1, Years =0	M31092	ODS
58	Intensity [824572]	15298.939	10044.918	320385	OPTIMA =178, EPNo =5, Years =2.13	M31094	ODS
59	Intensity [824691]	13034.486	14184.887	320387	OPTIMA =178, EPNo =3, Years =1.11	M31093	ODS
60	Intensity [824803]	7400.756	5793.404	320389	OPTIMA =158, EPNo =3, Years =1.16	M31084	AD
61	Intensity [824859]	9948.737	7020.818	320390	OPTIMA =158, EPNo =5, Years =2.14	M31085	AD
62	Intensity [824982]	17075.73	8517.562	320392	OPTIMA =158, EPNo =1, Years =0	M31083	AD
63	Intensity [825069]	29571.67	14877.532	320393	OPTIMA =271, EPNo =3, Years =1.09	M31155	sCTL
64	Intensity [825377]	34021.375	16760.557	320398	OPTIMA =271, EPNo =1, Years =0	M31154	sCTL
65	Intensity [826169]	17604.535	7599.067	320410	OPTIMA =301, EPNo =3, Years =0.98	M31166	sCTL
66	Intensity [826282]	15889.811	9933.112	320412	OPTIMA =301, EPNo =1, Years =0	M31165	sCTL
67	Intensity [828645]	36044.805	19500.145	320437	OPTIMA =229, EPNo =11, Years =5.04	M31133	pMCI
68	Intensity [828758]	56116.891	23848.605	320439	OPTIMA =229, EPNo =13, Years =5.94	M31134	pMCI

69	Intensity [828869]	43795.227	20892.049	320441	OPTIMA =229, EPNo =3, Years =1.07	M31129	pMCI
70	Intensity [829005]	62685.891	32320.695	320443	OPTIMA =229, EPNo =5, Years =2.03	M31130	pMCI
71	Intensity [829171]	50952.895	25798.342	320445	OPTIMA =229, EPNo =1, Years =0	M31128	pMCI
72	Intensity [829504]	49917.469	18075.812	320449	OPTIMA =229, EPNo =7, Years =3.02	M31131	pMCI
73	Intensity [829669]	38058.805	20424.459	320451	OPTIMA =229, EPNo =9, Years =4.06	M31132	pMCI
74	Intensity [830676]	18777.398	11071.506	320462	OPTIMA =74, EPNo =5, Years =2.09	M31032	sCTL
75	Intensity [830838]	11960.18	12586.79	320464	OPTIMA =74, EPNo =3, Years =1	M31031	sCTL
76	Intensity [831014]	17251.9	13438.599	320466	OPTIMA =74, EPNo =1, Years =0	M31030	sCTL
77	Intensity [831338]	15136.217	16399.102	320470	OPTIMA =102, EPNo =5, Years =1.94	M31046	AD
78	Intensity [831497]	13904.746	19613.176	320472	OPTIMA =102, EPNo =1, Years =0	M31045	AD
79	Intensity [831667]	12592.551	14494.191	320474	OPTIMA =102, EPNo =7, Years =2.93	M31047	AD
80	Intensity [832325]	11733.346	13410.118	320482	OPTIMA =66, EPNo =5, Years =2.12	M31029	sCTL
81	Intensity [832489]	19227.568	18507.486	320484	OPTIMA =66, EPNo =1, Years =0	M31028	sCTL
82	Intensity [832740]	20701.086	11758.547	320487	OPTIMA =84, EPNo =1, Years =0	M31037	sCTL
83	Intensity [832981]	24535.988	9567.77	320490	OPTIMA =84, EPNo =3, Years =1.06	M31038	sCTL
84	Intensity [833401]	4672.511	3175.845	320494	OPTIMA =91, EPNo =3, Years =1.12	M31039	sCTL
85	Intensity [833745]	5838.401	4495.859	320497	OPTIMA =91, EPNo =5, Years =2.16	M31040	sCTL
86	Intensity [835230]	55690	19121.961	320510	OPTIMA =105, EPNo =13, Years =6.12	M31052	AD
87	Intensity [835659]	25326.582	12459.332	320524	OPTIMA =252, EPNo =1, Years =0	M31145	sCTL
88	Intensity [835718]	16205.523	5557.428	320526	OPTIMA =252, EPNo =5, Years =2.19	M31147	sCTL
89	Intensity [835779]	16757.789	7169.875	320528	OPTIMA =252, EPNo =3, Years =1.1	M31146	sCTL
90	Intensity [835949]	34947.141	25349.48	320533	OPTIMA =147, EPNo =3, Years =1.09	M31074	AD
91	Intensity [836026]	25849.152	26796.977	320535	OPTIMA =147, EPNo =1, Years =0	M31073	AD
92	Intensity [836090]	31312.633	32848.406	320537	OPTIMA =147, EPNo =5, Years =1.97	M31075	AD
93	Intensity [836149]	26582.359	19985.373	320539	OPTIMA =37, EPNo =1, Years =0	M31006	sCTL
94	Intensity [836233]	31490.633	19939.605	320541	OPTIMA =37, EPNo =3, Years =1.15	M31007	sCTL
95	Intensity [836413]	20789.656	12291.176	320545	OPTIMA =95, EPNo =3, Years =1.01	M31042	sCTL
96	Intensity [836492]	33799.219	19815.064	320547	OPTIMA =95, EPNo =1, Years =0	M31041	sCTL
97	Intensity [836542]	13998.232	12283.918	320549	OPTIMA =100, EPNo =1, Years =0	M31043	AD
98	Intensity [836612]	11317.936	7962.537	320552	OPTIMA =100, EPNo =3, Years =1.17	M31044	AD
99	Intensity [838918]	14109.621	15131.911	320594	OPTIMA =33, EPNo =5, Years =2.98	M31003	sCTL
100	Intensity [838956]	8676.066	11089.956	320596	OPTIMA =33, EPNo =7, Years =3.98	M31004	sCTL
101	Intensity [838984]	18726.314	29273.668	320598	OPTIMA =33, EPNo =3, Years =1.97	M31002	sCTL
102	Intensity [839011]	7872.565	7583.321	320600	OPTIMA =33, EPNo =9, Years =4.96	M31005	sCTL
103	Intensity [839184]	8072.375	11423.941	320610	OPTIMA =156, EPNo =3, Years =1.12	M31081	AD
104	Intensity [839219]	9021.639	5146.816	320612	OPTIMA =156, EPNo =1, Years =0	M31080	AD
105	Intensity [839257]	9478.207	3849.471	320614	OPTIMA =156, EPNo =5, Years =2.12	M31082	AD
106	Intensity [839308]	7936.91	4903.26	320617	OPTIMA =51, EPNo =3, Years =0.96	M31012	sCTL
107	Intensity [839343]	13791.634	4801.814	320619	OPTIMA =51, EPNo =1, Years =0	M31011	sCTL
108	Intensity [839378]	7357.368	9364.831	320621	OPTIMA =107, EPNo =3, Years =1.11	M31054	AD
109	Intensity [839413]	11150.439	14542.863	320623	OPTIMA =107, EPNo =1, Years =0	M31053	AD
110	Intensity [839450]	20606.359	18700.309	320625	OPTIMA =118, EPNo =1, Years =0	M31059	AD
111	Intensity [839519]	19768.895	11739.901	320629	OPTIMA =118, EPNo =3, Years =1.12	M31060	AD
112	Intensity [839560]	6932.368	16918.223	320631	OPTIMA =121, EPNo =3, Years =1.12	M31062	AD
113	Intensity [839640]	10197.193	17785.133	320634	OPTIMA =121, EPNo =1, Years =0	M31061	AD
114	Intensity [839707]	11005.159	9212.961	320637	OPTIMA =188, EPNo =1, Years =0	M31098	ODS
115	Intensity [839829]	16652.141	6394.959	320642	OPTIMA =188, EPNo =3, Years =1.04	M31099	pCTL.AD
116	Intensity [840387]	7138.538	10050.993	320658	OPTIMA =114, EPNo =7, Years =3.1	M31058	AD
117	Intensity [840454]	23177.355	10978.377	320660	OPTIMA =114, EPNo =3, Years =1.16	M31056	AD
118	Intensity [840498]	27947.738	13939.293	320661	OPTIMA =114, EPNo =1, Years =0	M31055	AD
119	Intensity [840573]	9054.778	5635.565	320663	OPTIMA =114, EPNo =5, Years =2.14	M31057	AD
120	Intensity [840710]	10396.062	7800.37	320664	OPTIMA =178, EPNo =7, Years =3.14	M31095	ODS
121	Intensity [840891]	14636.365	11070.732	320666	OPTIMA =178, EPNo =1, Years =0	M31092	ODS
122	Intensity [841050]	9495.2	6377.734	320670	OPTIMA =178, EPNo =5, Years =2.13	M31094	ODS
123	Intensity [841147]	9949.982	12465.832	320672	OPTIMA =178, EPNo =3, Years =1.11	M31093	ODS
124	Intensity [841242]	5719.258	4439.874	320674	OPTIMA =158, EPNo =3, Years =1.16	M31084	AD
125	Intensity [841290]	8334.401	6385.896	320675	OPTIMA =158, EPNo =5, Years =2.14	M31085	AD
126	Intensity [841400]	9527.421	6296.457	320677	OPTIMA =158, EPNo =1, Years =0	M31083	AD
127	Intensity [841453]	21848.117	12584.837	320678	OPTIMA =271, EPNo =3, Years =1.09	M31155	sCTL
128	Intensity [841761]	25723.408	13737.104	320683	OPTIMA =271, EPNo =1, Years =0	M31154	sCTL
129	Intensity [842155]	15926.613	8578.912	320695	OPTIMA =301, EPNo =3, Years =0.98	M31166	sCTL
130	Intensity [842216]	7166.391	7029.471	320697	OPTIMA =301, EPNo =1, Years =0	M31165	sCTL
131	Intensity [809230]	15688.6	9431.209	320174	OPTIMA =16, EPNo =5, Years =2.01	M30996	AD
132	Intensity [809367]	27019.195	11975.903	320176	OPTIMA =16, EPNo =7, Years =2.98	M30997	AD
133	Intensity [809444]	10283.305	5855.12	320178	OPTIMA =16, EPNo =15, Years =6.96	M31001	AD
134	Intensity [809513]	10948.164	7782.336	320180	OPTIMA =16, EPNo =11, Years =5	M30999	AD
135	Intensity [809586]	13317.098	10537.309	320182	OPTIMA =16, EPNo =13, Years =5.97	M31000	AD
136	Intensity [809727]	25476.41	14580.656	320186	OPTIMA =16, EPNo =3, Years =0.99	M30995	AD
137	Intensity [809802]	12520.501	13337.653	320188	OPTIMA =16, EPNo =9, Years =3.98	M30998	AD
138	Intensity [809835]	118906.125	17156.432	320189	OPTIMA =150, EPNo =3, Years =1.5	M31077	AD
139	Intensity [809903]	111535.266	18317.273	320191	OPTIMA =150, EPNo =7, Years =3.32	M31079	AD

Supplementary Table 6. SME1 and SME2 AUCs in Cohort-2.

140	Intensity [809984]	119597.406	20553.746	320193	OPTIMA =150, EPNo =5, Years =2.37	M31078	AD
141	Intensity [810153]	101708.906	19386.131	320197	OPTIMA =150, EPNo =1, Years =0	M31076	AD
142	Intensity [810261]	44870.969	17036.488	320199	OPTIMA =159, EPNo =5, Years =2.05	M31088	AD
143	Intensity [810354]	38966.68	15081.756	320201	OPTIMA =159, EPNo =3, Years =1	M31087	AD
144	Intensity [810429]	40641.031	14774.625	320203	OPTIMA =159, EPNo =1, Years =0	M31086	AD
145	Intensity [810473]	49474.328	22772.781	320204	OPTIMA =57, EPNo =3, Years =1.08	M31018	sCTL
146	Intensity [810521]	49558.473	13727.971	320205	OPTIMA =159, EPNo =7, Years =2.99	M31089	AD
147	Intensity [810560]	43226.82	13356.912	320206	OPTIMA =57, EPNo =1, Years =0	M31017	sCTL
148	Intensity [810715]	34755.766	22518.521	320209	OPTIMA =214, EPNo =3, Years =0.97	M31107	pCTL.MCI
149	Intensity [810803]	36729.102	21853.027	320211	OPTIMA =214, EPNo =1, Years =0	M31106	pCTL.MCI
150	Intensity [810914]	22395.535	12953.955	320213	OPTIMA =214, EPNo =5, Years =2.1	M31108	pCTL.MCI
151	Intensity [811047]	51306.555	33552.137	320214	OPTIMA =137, EPNo =3, Years =0.93	M31069	AD
152	Intensity [811228]	50056.305	28253.535	320216	OPTIMA =137, EPNo =1, Years =0	M31068	AD
153	Intensity [811325]	39310.25	19112.559	320217	OPTIMA =222, EPNo =5, Years =1.97	M31122	pMCI
154	Intensity [811574]	64097.754	31540.213	320221	OPTIMA =222, EPNo =1, Years =0	M31120	pMCI
155	Intensity [811723]	46450.523	22249.982	320223	OPTIMA =222, EPNo =3, Years =0.96	M31121	pMCI
156	Intensity [811796]	11617.707	8986.884	320224	OPTIMA =300, EPNo =1, Years =0	M31163	sCTL
157	Intensity [811920]	12884.386	8270.049	320226	OPTIMA =300, EPNo =3, Years =1.03	M31164	sCTL
158	Intensity [813234]	40410.215	13114.565	320237	OPTIMA =105, EPNo =1, Years =0	M31048	AD
159	Intensity [813323]	75278.977	15287.799	320238	OPTIMA =105, EPNo =13, Years =6.12	M31052	AD
160	Intensity [813397]	29479.994	14511.562	320239	OPTIMA =59, EPNo =7, Years =3.24	M31022	sCTL
161	Intensity [813537]	36255.262	13634.637	320241	OPTIMA =59, EPNo =5, Years =2.2	M31021	sCTL
162	Intensity [813661]	40755.953	13045.866	320243	OPTIMA =59, EPNo =1, Years =0	M31019	sCTL
163	Intensity [813791]	20596.613	6328.015	320245	OPTIMA =59, EPNo =9, Years =4.19	M31023	sCTL
164	Intensity [813848]	18907.289	6179.393	320246	OPTIMA =59, EPNo =11, Years =5.19	M31024	sCTL
165	Intensity [814050]	20636.492	19726.168	320249	OPTIMA =82, EPNo =3, Years =1.03	M31033	sCTL
166	Intensity [814118]	47699.383	17411.779	320250	OPTIMA =59, EPNo =3, Years =1.1	M31020	sCTL
167	Intensity [814173]	23665.426	21344.014	320251	OPTIMA =82, EPNo =7, Years =3.07	M31035	sCTL
168	Intensity [814299]	19742.566	17190.191	320253	OPTIMA =82, EPNo =5, Years =1.96	M31034	sCTL
169	Intensity [814420]	18663.91	19846.129	320255	OPTIMA =82, EPNo =9, Years =4.01	M31036	sCTL
170	Intensity [814535]	17340.367	14304.59	320257	OPTIMA =207, EPNo =5, Years =2.09	M31102	pCTL.AD
171	Intensity [814607]	20718.389	13801.277	320258	OPTIMA =207, EPNo =1, Years =0	M31100	pCTL.AD
172	Intensity [814883]	20803.301	16291.553	320262	OPTIMA =207, EPNo =7, Years =3.1	M31103	pCTL.AD
173	Intensity [815029]	17296.184	11564.561	320264	OPTIMA =207, EPNo =3, Years =1.07	M31101	pCTL.AD
174	Intensity [815151]	53565.109	26489.062	320266	OPTIMA =145, EPNo =3, Years =1.14	M31071	AD
175	Intensity [815297]	42198.031	18156.805	320268	OPTIMA =145, EPNo =7, Years =3.09	M31072	AD
176	Intensity [815448]	37927.68	21457.039	320270	OPTIMA =145, EPNo =1, Years =0	M31070	AD
177	Intensity [815699]	57853.008	39504.871	320274	OPTIMA =226, EPNo =1, Years =0	M31125	pMCI
178	Intensity [815851]	65832.031	42249.258	320276	OPTIMA =226, EPNo =3, Years =1.06	M31126	pMCI
179	Intensity [815990]	57875.699	41544.598	320278	OPTIMA =226, EPNo =7, Years =3.06	M31127	pMCI
180	Intensity [816071]	50078.574	8004.773	320279	OPTIMA =293, EPNo =1, Years =0	M31161	sCTL
181	Intensity [816199]	10526.518	6491.799	320281	OPTIMA =293, EPNo =3, Years =1.01	M31162	sCTL
182	Intensity [816259]	77633.312	16986.824	320282	OPTIMA =180, EPNo =1, Years =0	M31096	ODS
183	Intensity [816458]	25355.887	17915.539	320285	OPTIMA =215, EPNo =1, Years =0	M31109	pCTL.MCI
184	Intensity [816523]	72851.477	19421.277	320286	OPTIMA =180, EPNo =3, Years =1.29	M31097	ODS
185	Intensity [816584]	105063.156	19453.141	320287	OPTIMA =245, EPNo =1, Years =0	M31140	sCTL
186	Intensity [819288]	29822.393	22160.42	320309	OPTIMA =127, EPNo =9, Years =3.98	M31066	AD
187	Intensity [819409]	1243.15	1293.552	320311	OPTIMA =127, EPNo =1, Years =0	M31063	AD
188	Intensity [819541]	39526.438	21263.803	320313	OPTIMA =127, EPNo =7, Years =3.02	M31065	AD
189	Intensity [819698]	30217.207	27976.963	320315	OPTIMA =127, EPNo =3, Years =1.01	M31064	AD
190	Intensity [819837]	30630.457	20395.523	320317	OPTIMA =127, EPNo =11, Years =4.92	M31067	AD
191	Intensity [819915]	21301.363	22575.566	320318	OPTIMA =331, EPNo =3, Years =1.08	M31168	sCTL
192	Intensity [820092]	65932.789	26930.004	320321	OPTIMA =54, EPNo =1, Years =0	M31013	sCTL
193	Intensity [820153]	79450.461	25595.004	320322	OPTIMA =54, EPNo =5, Years =2.14	M31014	sCTL
194	Intensity [820215]	46751.113	24928.871	320323	OPTIMA =331, EPNo =1, Years =0	M31167	sCTL
195	Intensity [820273]	55031.402	22632.928	320324	OPTIMA =54, EPNo =9, Years =3.98	M31015	sCTL
196	Intensity [820346]	55633.195	16275.031	320325	OPTIMA =54, EPNo =11, Years =5.21	M31016	sCTL
197	Intensity [820472]	58999.645	26316.398	320327	OPTIMA =216, EPNo =3, Years =1.05	M31111	pCTL.MCI
198	Intensity [820986]	49596.254	21832.406	320334	OPTIMA =216, EPNo =1, Years =0	M31110	pCTL.MCI
199	Intensity [821120]	32022.223	17992.482	320336	OPTIMA =63, EPNo =3, Years =0.99	M31026	sCTL
200	Intensity [821248]	29731.086	16918.373	320338	OPTIMA =63, EPNo =1, Years =0	M31025	sCTL
201	Intensity [821372]	32727.059	16691.828	320340	OPTIMA =63, EPNo =9, Years =3.99	M31027	sCTL
202	Intensity [821498]	23906.898	10408.16	320342	OPTIMA =241, EPNo =5, Years =2.01	M31139	sCTL
203	Intensity [821732]	1110.512	694.65	320346	OPTIMA =241, EPNo =1, Years =0	M31137	pMCI
204	Intensity [821860]	15281.621	10931.395	320348	OPTIMA =241, EPNo =3, Years =1.02	M31138	sCTL
205	Intensity [821921]	64220.711	24873.02	320349	OPTIMA =173, EPNo =3, Years =0.99	M31091	AD
206	Intensity [822043]	68763.164	23242.98	320351	OPTIMA =173, EPNo =1, Years =0	M31090	AD
207	Intensity [822096]	29002.42	13421.753	320352	OPTIMA =281, EPNo =5, Years =2.06	M31159	sCTL
208	Intensity [822331]	15357.52	8724.931	320356	OPTIMA =281, EPNo =7, Years =3.03	M31160	sCTL
209	Intensity [822386]	48619.324	29047.186	320357	OPTIMA =225, EPNo =3, Years =1.01	M31124	pMCI
210	Intensity [822492]	27768.445	13513.383	320359	OPTIMA =281, EPNo =1, Years =0	M31158	sCTL

211	Intensity [822546]	55734.207	29975.949	320360	OPTIMA =225, EPNo =1, Years =0	M31123	pMCI
212	Intensity [822600]	65524.613	33499.121	320361	OPTIMA =216, EPNo =5, Years =2.07	M31112	pCTL.MCI
213	Intensity [823985]	51378.184	34915.781	320372	OPTIMA =219, EPNo =1, Years =0	M31113	pCTL.MCI
214	Intensity [824128]	48061.621	20088.816	320374	OPTIMA =219, EPNo =5, Years =1.91	M31115	pMCI
215	Intensity [824427]	71022.312	25134.117	320380	OPTIMA =220, EPNo =5, Years =2.03	M31118	pMCI
216	Intensity [824495]	74268.641	26788.688	320384	OPTIMA =220, EPNo =3, Years =1.07	M31117	pMCI
217	Intensity [824637]	62278.773	24056.48	320386	OPTIMA =220, EPNo =1, Years =0	M31116	pMCI
218	Intensity [824759]	63078.055	26375.031	320388	OPTIMA =220, EPNo =7, Years =2.97	M31119	pMCI
219	Intensity [824922]	23073.01	10991.855	320391	OPTIMA =257, EPNo =9, Years =4	M31153	sCTL
220	Intensity [825246]	30473.965	22096.83	320396	OPTIMA =257, EPNo =1, Years =0	M31151	sCTL
221	Intensity [825455]	25495.33	15256.975	320399	OPTIMA =48, EPNo =7, Years =3.2	M31010	sCTL
222	Intensity [825569]	30198.07	13842.228	320401	OPTIMA =48, EPNo =1, Years =0	M31008	sCTL
223	Intensity [825716]	21613.518	12146.823	320403	OPTIMA =48, EPNo =3, Years =1.21	M31009	sCTL
224	Intensity [825771]	17986.988	6286.771	320404	OPTIMA =279, EPNo =1, Years =0	M31156	sCTL
225	Intensity [825837]	51620.438	30008.328	320405	OPTIMA =246, EPNo =5, Years =1.93	M31144	sCTL
226	Intensity [826054]	35014.125	4882.391	320408	OPTIMA =279, EPNo =3, Years =0.93	M31157	sCTL
227	Intensity [826115]	61607.695	26958.891	320409	OPTIMA =246, EPNo =1, Years =0	M31142	sCTL
228	Intensity [826229]	54095.992	29749.973	320411	OPTIMA =246, EPNo =3, Years =0.95	M31143	sCTL
229	Intensity [826441]	46377.344	11727.834	320415	OPTIMA =443, EPNo =1, Years =0	M31169	sCTL
230	Intensity [826792]	47856.504	16802.568	320420	OPTIMA =443, EPNo =3, Years =1.04	M31170	sCTL
231	Intensity [827022]	68552.266	10784.427	320424	OPTIMA =212, EPNo =3, Years =0.94	M31105	pCTL.MCI
232	Intensity [827087]	169039.781	17037.479	320425	OPTIMA =212, EPNo =1, Years =0	M31104	pCTL.MCI
233	Intensity [828701]	17191.305	15513.914	320438	OPTIMA =16, EPNo =5, Years =2.01	M30996	AD
234	Intensity [828812]	30286.078	21084.52	320440	OPTIMA =16, EPNo =7, Years =2.98	M30997	AD
235	Intensity [828935]	14176.311	13140.918	320442	OPTIMA =16, EPNo =15, Years =6.96	M31001	AD
236	Intensity [829083]	11445.459	10770.268	320444	OPTIMA =16, EPNo =11, Years =5	M30999	AD
237	Intensity [829258]	16660.301	13867.939	320446	OPTIMA =16, EPNo =13, Years =5.97	M31000	AD
238	Intensity [829591]	20801.258	15104.018	320450	OPTIMA =16, EPNo =3, Years =0.99	M30995	AD
239	Intensity [830185]	15177.918	16884.572	320456	OPTIMA =16, EPNo =9, Years =3.98	M30998	AD
240	Intensity [831830]	42584.516	19192.258	320476	OPTIMA =57, EPNo =3, Years =1.08	M31018	sCTL
241	Intensity [832004]	33775.977	19807.176	320478	OPTIMA =57, EPNo =1, Years =0	M31017	sCTL
242	Intensity [835199]	18715.082	10797.506	320509	OPTIMA =105, EPNo =1, Years =0	M31048	AD
243	Intensity [835256]	28266.758	18037.297	320511	OPTIMA =59, EPNo =7, Years =3.24	M31022	sCTL
244	Intensity [835282]	68581.766	24997.633	320512	OPTIMA =105, EPNo =3, Years =1.16	M31049	AD
245	Intensity [835309]	30207.195	13607.68	320513	OPTIMA =59, EPNo =5, Years =2.2	M31021	sCTL
246	Intensity [835335]	64817.336	21743.473	320514	OPTIMA =105, EPNo =7, Years =3.12	M31051	AD
247	Intensity [835360]	49054.984	24664.648	320515	OPTIMA =59, EPNo =1, Years =0	M31019	sCTL
248	Intensity [835386]	34852.109	19709.602	320516	OPTIMA =105, EPNo =5, Years =2.13	M31050	AD
249	Intensity [835411]	19484.801	9668.724	320517	OPTIMA =59, EPNo =9, Years =4.19	M31023	sCTL
250	Intensity [835436]	16965.342	8415.641	320518	OPTIMA =59, EPNo =11, Years =5.19	M31024	sCTL
251	Intensity [835584]	43639.109	17039.482	320522	OPTIMA =59, EPNo =3, Years =1.1	M31020	sCTL
252	Intensity [836763]	23505.307	23963.562	320557	OPTIMA =215, EPNo =1, Years =0	M31109	pCTL.MCI
253	Intensity [839046]	17616.943	16663.828	320602	OPTIMA =331, EPNo =3, Years =1.08	M31168	sCTL
254	Intensity [839097]	34604.289	17504.709	320605	OPTIMA =54, EPNo =1, Years =0	M31013	sCTL
255	Intensity [839115]	44408.707	20096.844	320606	OPTIMA =54, EPNo =5, Years =2.14	M31014	sCTL
256	Intensity [839133]	24605.383	19716.721	320607	OPTIMA =331, EPNo =1, Years =0	M31167	sCTL
257	Intensity [839150]	32887.844	15382.223	320608	OPTIMA =54, EPNo =9, Years =3.98	M31015	sCTL
258	Intensity [839909]	32484.008	12402.23	320646	OPTIMA =54, EPNo =11, Years =5.21	M31016	sCTL
259	Intensity [841795]	18183.551	10810.754	320684	OPTIMA =48, EPNo =7, Years =3.2	M31010	sCTL
260	Intensity [841826]	46965.328	10149.358	320685	OPTIMA =212, EPNo =3, Years =0.94	M31105	pCTL.MCI
261	Intensity [841858]	21122.492	11331.919	320686	OPTIMA =48, EPNo =1, Years =0	M31008	sCTL
262	Intensity [841892]	102505.086	14057.739	320687	OPTIMA =212, EPNo =1, Years =0	M31104	pCTL.MCI
263	Intensity [841923]	15310.918	11192.458	320688	OPTIMA =48, EPNo =3, Years =1.21	M31009	sCTL
264	Intensity [841955]	9309.554	4623.473	320689	OPTIMA =279, EPNo =1, Years =0	M31156	sCTL
265	Intensity [841986]	42365.008	27436.066	320690	OPTIMA =246, EPNo =5, Years =1.93	M31144	sCTL
266	Intensity [842086]	25229.969	4609.71	320693	OPTIMA =279, EPNo =3, Years =0.93	M31157	sCTL
267	Intensity [842119]	50061.828	25825.834	320694	OPTIMA =246, EPNo =1, Years =0	M31142	sCTL
268	Intensity [842185]	43897.578	37110.867	320696	OPTIMA =246, EPNo =3, Years =0.95	M31143	sCTL
269	Intensity [842314]	17730.758	9033.494	320700	OPTIMA =443, EPNo =1, Years =0	M31169	sCTL
270	Intensity [842468]	35056.496	13307.49	320705	OPTIMA =443, EPNo =3, Years =1.04	M31170	sCTL
271	Intensity [825307]	25055.346	13514.977	320397	OPTIMA =257, EPNo =3, Years =1.15	M31152	sCTL
272	Intensity [826391]	15564.623	9871.209	320414	OPTIMA =255, EPNo =3, Years =1.2	M31149	sCTL
273	Intensity [826534]	30457.312	10662.9	320416	OPTIMA =255, EPNo =5, Years =2.2	M31150	sCTL
274	Intensity [826597]	19651.367	13312.865	320417	OPTIMA =237, EPNo =3, Years =1.08	M31136	pMCI
275	Intensity [826852]	21357.914	11280.356	320421	OPTIMA =255, EPNo =1, Years =0	M31148	sCTL
276	Intensity [826906]	48701.641	24642.047	320422	OPTIMA =237, EPNo =1, Years =0	M31135	pMCI
277	Intensity [826960]	64411.664	9950.537	320423	OPTIMA =219, EPNo =3, Years =1.01	M31114	pMCI
278	Intensity [827154]	90355.617	18682.408	320426	OPTIMA =245, EPNo =3, Years =1.13	M31141	sCTL
279	Intensity [830592]	99632.852	24303.668	320461	OPTIMA =150, EPNo =3, Years =1.5	M31077	AD
280	Intensity [830759]	85049.516	23119.797	320463	OPTIMA =150, EPNo =7, Years =3.32	M31079	AD
281	Intensity [830932]	102358.422	19259.59	320465	OPTIMA =150, EPNo =5, Years =2.37	M31078	AD

282	Intensity [831257]	80339.117	22261.203	320469	OPTIMA =150, EPNo =1, Years =0	M31076	AD
283	Intensity [831419]	22815.453	16738.031	320471	OPTIMA =159, EPNo =5, Years =2.05	M31088	AD
284	Intensity [831577]	20288.164	11329.955	320473	OPTIMA =159, EPNo =3, Years =1	M31087	AD
285	Intensity [831755]	28374.637	17758.699	320475	OPTIMA =159, EPNo =1, Years =0	M31086	AD
286	Intensity [831914]	28211.35	13753.094	320477	OPTIMA =159, EPNo =7, Years =2.99	M31089	AD
287	Intensity [832244]	29610.25	27539.16	320481	OPTIMA =214, EPNo =3, Years =0.97	M31107	pCTL.MCI
288	Intensity [832410]	40393.848	26252.969	320483	OPTIMA =214, EPNo =1, Years =0	M31106	pCTL.MCI
289	Intensity [832578]	22482.379	15864.934	320485	OPTIMA =214, EPNo =5, Years =2.1	M31108	pCTL.MCI
290	Intensity [832659]	47366.098	35675.445	320486	OPTIMA =137, EPNo =3, Years =0.93	M31069	AD
291	Intensity [832820]	34621.891	27474.941	320488	OPTIMA =137, EPNo =1, Years =0	M31068	AD
292	Intensity [832900]	35125.094	15319.671	320489	OPTIMA =222, EPNo =5, Years =1.97	M31122	pMCI
293	Intensity [833283]	45681.094	31923.934	320493	OPTIMA =222, EPNo =1, Years =0	M31120	pMCI
294	Intensity [833500]	30852.227	15328.71	320495	OPTIMA =222, EPNo =3, Years =0.96	M31121	pMCI
295	Intensity [833602]	5339.627	7285.993	320496	OPTIMA =300, EPNo =1, Years =0	M31163	sCTL
296	Intensity [833874]	8807.146	6195.71	320498	OPTIMA =300, EPNo =3, Years =1.03	M31164	sCTL
297	Intensity [835542]	15389.113	20133.906	320521	OPTIMA =82, EPNo =3, Years =1.03	M31033	sCTL
298	Intensity [835633]	27714.734	27839.611	320523	OPTIMA =82, EPNo =7, Years =3.07	M31035	sCTL
299	Intensity [835694]	22354.938	22362.23	320525	OPTIMA =82, EPNo =5, Years =1.96	M31034	sCTL
300	Intensity [835751]	23664.059	27887.199	320527	OPTIMA =82, EPNo =9, Years =4.01	M31036	sCTL
301	Intensity [835820]	17854.129	15290.269	320529	OPTIMA =207, EPNo =5, Years =2.09	M31102	pCTL.AD
302	Intensity [835855]	22111.656	16212.458	320530	OPTIMA =207, EPNo =1, Years =0	M31100	pCTL.AD
303	Intensity [835994]	26573.965	23319.305	320534	OPTIMA =207, EPNo =7, Years =3.1	M31103	pCTL.AD
304	Intensity [836054]	18985.836	16816.859	320536	OPTIMA =207, EPNo =3, Years =1.07	M31101	pCTL.AD
305	Intensity [836115]	59949.758	37160.883	320538	OPTIMA =145, EPNo =3, Years =1.14	M31071	AD
306	Intensity [836185]	45814.266	29447.27	320540	OPTIMA =145, EPNo =7, Years =3.09	M31072	AD
307	Intensity [836261]	42030.945	25375.852	320542	OPTIMA =145, EPNo =1, Years =0	M31070	AD
308	Intensity [836453]	66875.891	46196.906	320546	OPTIMA =226, EPNo =1, Years =0	M31125	pMCI
309	Intensity [836566]	74791.75	44601.508	320550	OPTIMA =226, EPNo =7, Years =3.06	M31127	pMCI
310	Intensity [836589]	51865.547	12283.709	320551	OPTIMA =293, EPNo =1, Years =0	M31161	sCTL
311	Intensity [836636]	6922.921	6256.759	320553	OPTIMA =293, EPNo =3, Years =1.01	M31162	sCTL
312	Intensity [836658]	75043.047	19616.281	320554	OPTIMA =180, EPNo =1, Years =0	M31096	ODS
313	Intensity [836797]	76994.523	22763.586	320558	OPTIMA =180, EPNo =3, Years =1.29	M31097	ODS
314	Intensity [836842]	89418.109	18414.684	320559	OPTIMA =245, EPNo =1, Years =0	M31140	sCTL
315	Intensity [836866]	1876.058	233.031	320560	OPTIMA =226, EPNo =3, Years =1.06	M31126	pMCI
316	Intensity [838937]	1602.855	1328.321	320595	OPTIMA =127, EPNo =1, Years =0	M31063	AD
317	Intensity [838974]	23793.859	16770.666	320597	OPTIMA =127, EPNo =7, Years =3.02	M31065	AD
318	Intensity [838993]	22631.133	32763.295	320599	OPTIMA =127, EPNo =3, Years =1.01	M31064	AD
319	Intensity [839029]	20357.102	14585.187	320601	OPTIMA =127, EPNo =11, Years =4.92	M31067	AD
320	Intensity [839202]	35109.238	25330.07	320611	OPTIMA =216, EPNo =3, Years =1.05	M31111	pCTL.MCI
321	Intensity [839239]	40038.758	25861.359	320613	OPTIMA =216, EPNo =5, Years =2.07	M31112	pCTL.MCI
322	Intensity [839325]	31394.199	16078.314	320618	OPTIMA =216, EPNo =1, Years =0	M31110	pCTL.MCI
323	Intensity [839361]	17546.047	14257.319	320620	OPTIMA =63, EPNo =3, Years =0.99	M31026	sCTL
324	Intensity [839396]	19063.621	10022.699	320622	OPTIMA =63, EPNo =1, Years =0	M31025	sCTL
325	Intensity [839431]	26851.676	14275.388	320624	OPTIMA =63, EPNo =9, Years =3.99	M31027	sCTL
326	Intensity [839468]	21699.035	9717.917	320626	OPTIMA =241, EPNo =5, Years =2.01	M31139	sCTL
327	Intensity [839538]	1061.244	615.347	320630	OPTIMA =241, EPNo =1, Years =0	M31137	pMCI
328	Intensity [839594]	11072.477	9119.02	320632	OPTIMA =241, EPNo =3, Years =1.02	M31138	sCTL
329	Intensity [839620]	41395.57	20954.197	320633	OPTIMA =173, EPNo =3, Years =0.99	M31091	AD
330	Intensity [839661]	58007.855	17878.891	320635	OPTIMA =173, EPNo =1, Years =0	M31090	AD
331	Intensity [839684]	18248.332	9538.059	320636	OPTIMA =281, EPNo =5, Years =2.06	M31159	sCTL
332	Intensity [839791]	10889.596	7095.629	320640	OPTIMA =281, EPNo =7, Years =3.03	M31160	sCTL
333	Intensity [839810]	30865.465	24474.539	320641	OPTIMA =225, EPNo =3, Years =1.01	M31124	pMCI
334	Intensity [839849]	19208.145	8645.852	320643	OPTIMA =281, EPNo =1, Years =0	M31158	sCTL
335	Intensity [839870]	34556.148	19421.6	320644	OPTIMA =225, EPNo =1, Years =0	M31123	pMCI
336	Intensity [839890]	23955.453	16059.555	320645	OPTIMA =127, EPNo =9, Years =3.98	M31066	AD
337	Intensity [840361]	39277.859	24161.391	320657	OPTIMA =219, EPNo =1, Years =0	M31113	pCTL.MCI
338	Intensity [840417]	28211.156	16920.057	320659	OPTIMA =219, EPNo =5, Years =1.91	M31115	pMCI
339	Intensity [840537]	51060.316	7453.638	320662	OPTIMA =219, EPNo =3, Years =1.01	M31114	pMCI
340	Intensity [840838]	43825.512	18543.072	320665	OPTIMA =220, EPNo =5, Years =2.03	M31118	pMCI
341	Intensity [841007]	44617.793	18559.492	320669	OPTIMA =220, EPNo =3, Years =1.07	M31117	pMCI
342	Intensity [841097]	40558.996	17090.254	320671	OPTIMA =220, EPNo =1, Years =0	M31116	pMCI
343	Intensity [841195]	41352.68	18970.273	320673	OPTIMA =220, EPNo =7, Years =2.97	M31119	pMCI
344	Intensity [841346]	15109.942	10146.261	320676	OPTIMA =257, EPNo =9, Years =4	M31153	sCTL
345	Intensity [841694]	23896.834	15104.284	320681	OPTIMA =257, EPNo =1, Years =0	M31151	sCTL
346	Intensity [841729]	20583.258	9937.393	320682	OPTIMA =257, EPNo =3, Years =1.15	M31152	sCTL
347	Intensity [842249]	66383.172	25034.145	320698	OPTIMA =245, EPNo =3, Years =1.13	M31141	sCTL
348	Intensity [842281]	9383.795	9799.741	320699	OPTIMA =255, EPNo =3, Years =1.2	M31149	sCTL
349	Intensity [842343]	24148.301	10803.502	320701	OPTIMA =255, EPNo =5, Years =2.2	M31150	sCTL
350	Intensity [842374]	15967.262	12929.273	320702	OPTIMA =237, EPNo =3, Years =1.08	M31136	pMCI
351	Intensity [842498]	7920.779	9119.039	320706	OPTIMA =255, EPNo =1, Years =0	M31148	sCTL
352	Intensity [842528]	37405.305	24399.461	320707	OPTIMA =237, EPNo =1, Years =0	M31135	pMCI
